# Characterization of the late embryogenesis abundant (LEA) proteins family and their role in drought stress tolerance in upland cotton

**DOI:** 10.1186/s12863-017-0596-1

**Published:** 2018-01-15

**Authors:** Richard Odongo Magwanga, Pu Lu, Joy Nyangasi Kirungu, Hejun Lu, Xingxing Wang, Xiaoyan Cai, Zhongli Zhou, Zhenmei Zhang, Haron Salih, Kunbo Wang, Fang Liu

**Affiliations:** 1 Institute of Cotton Research, Chinese Academy of Agricultural Science (ICR, CAAS)/State Key Laboratory of Cotton Biology, Anyang, 455000 China; 2 School of physical and biological sciences (SPBS), Main campus, Jaramogi Oginga Odinga University of Science and Technology (JOOUST), P.O Box 210-40601, Bondo, Kenya

**Keywords:** Cotton (*Gossypium spp*), Identification, LEA proteins, miRNAs, Gene ontology, Gene expression, Genome, Drought

## Abstract

**Background:**

Late embryogenesis abundant (LEA) proteins are large groups of hydrophilic proteins with major role in drought and other abiotic stresses tolerance in plants. In-depth study and characterization of LEA protein families have been carried out in other plants, but not in upland cotton. The main aim of this research work was to characterize the late embryogenesis abundant (LEA) protein families and to carry out gene expression analysis to determine their potential role in drought stress tolerance in upland cotton. Increased cotton production in the face of declining precipitation and availability of fresh water for agriculture use is the focus for breeders, cotton being the backbone of textile industries and a cash crop for many countries globally.

**Results:**

In this work, a total of 242, 136 and 142 *LEA* genes were identified in *G. hirsutum*, *G. arboreum* and *G. raimondii* respectively. The identified genes were classified into eight groups based on their conserved domain and phylogenetic tree analysis. LEA 2 were the most abundant, this could be attributed to their hydrophobic character. Upland cotton *LEA* genes have fewer introns and are distributed in all chromosomes. Majority of the duplicated *LEA* genes were segmental. Syntenic analysis showed that greater percentages of *LEA* genes are conserved. Segmental gene duplication played a key role in the expansion of *LEA* genes. Sixty three miRNAs were found to target 89 genes, such as miR164, ghr-miR394 among others. Gene ontology analysis revealed that *LEA* genes are involved in desiccation and defense responses. Almost all the *LEA* genes in their promoters contained ABRE, MBS, W-Box and TAC-elements, functionally known to be involved in drought stress and other stress responses. Majority of the *LEA* genes were involved in secretory pathways. Expression profile analysis indicated that most of the *LEA* genes were highly expressed in drought tolerant cultivars *Gossypium tomentosum* as opposed to drought susceptible, *G. hirsutum*. The tolerant genotypes have a greater ability to modulate genes under drought stress than the more susceptible upland cotton cultivars.

**Conclusion:**

The finding provides comprehensive information on *LEA* genes in upland cotton, *G. hirsutum* and possible function in plants under drought stress.

**Electronic supplementary material:**

The online version of this article (10.1186/s12863-017-0596-1) contains supplementary material, which is available to authorized users.

## Background

Drought stress has resulted in to massive losses in crop production and also has altered the natural equilibrium of the environment [1]. To save the ecosystem and enhance production, advanced molecular breeding is the recipe for activation and regulation of specific stress-related genes [2]. Water deficit stress do led to a series of changes including biochemical alterations like accumulation of osmolytes and specific proteins involved in stress tolerance [3]. One of the proteins that play a role in the mechanism of drought resistance is the LEA types of protein known as dehydrin [4]. In cotton production, drought is the main abiotic stress responsible for plant growth compromise and severe yield loss. Even though cotton is considered to be relatively tolerant to water deficit, its optimal growth and yield negatively affected when water supply is limited or interrupted [5]. Water is an essential element for biotic component of the biosphere, such that various responses have evolved to withstand water deficit in all plants and animals, to enable them withstand long periods of water deprivation by adopting a type of life condition known as anhydrobiosis [6].

There is great agronomic significance to understand cotton plant responses to water deficit due to the huge economic losses that results from drought [7]. Cotton metabolism and yield are negatively affected under water deficit conditions, especially at flowering stage [8]. Plants have acquired an evolutionary response to withstand the effect of low water availability, a condition that can disadvantage their growth and development. As immobile organisms, plants possess diverse strategies of responses to drought. Among the molecules highly associated with plant responses to water limitation are the late embryogenesis abundant (LEA) proteins [9]. These proteins are widespread in the plant kingdom and highly enriched during the late stages of embryogenesis and in vegetative tissues in response to water deficit [10].

LEA proteins were first discovered more than 30 years ago and were observed to accumulate at late stages of plant seed development [11]. The LEA proteins have been found in various tissues of abiotic stressed plants and non-plant organisms known to be tolerant to desiccation, such as bacteria and some invertebrates [12]. LEA proteins are members of a large group of hydrophilic, glycine-rich proteins present in a wide range of plant species [13]. This class of proteins are known to be intrinsically disordered in their structures and are mainly expressed under water deprivation condition [14]. The *LEA* genes are highly diverse, with wide distribution in the plant kingdom and has pivotal role in various stress tolerance responses [15].

Scientific investigations on LEA protein families have been on-going for more than two decades [16]. Although there has been a strong association of LEA protein families with environmental stress tolerance of significance drought and cold stress [17], LEA protein families for most of that time, their function has been entirely obscure [18]. Considerable evidence gives an indication that *LEA* genes are involved in desiccation, though their precise function is unknown [19]. The bacterial group 1 LEA proteins have the ability to block enzyme inactivation upon freeze–thaw treatments in vitro and it has analogous functions to plant LEA proteins [10]. Therefore, there is need to conduct a genome wide characterization of LEA protein families in cotton. The recent upland cotton genome publications, *G. hirsutum* [20], *G. arboreum* [21] and *Gossypium raimondii* [22], enabled us to carry out the identification and characterization of all cotton *LEA* genes. In this study, we identified 242, 136 and 142 candidate LEA proteins in *G. hirsutum*, *G. arboreum* and *G. raimondii* respectively, analysed their phylogenetic tree relationships, chromosomal positions, duplicated gene events, gene structure, conserved motif compositions and profiling analysis of gene expression from different cotton plant organs. Our results provides a strong platform for better understanding of the roles and evolutionary history of *LEA* genes, and will help in future studies of the molecular and biological functions of LEA protein families in cotton.

## Methods

### Identification of *LEA* gene families

The conserved LEA protein domains were downloaded from Hidden Markov Model (HMM) (PF 03760, PF03168, PF03242, PF02987, PF0477, PF10714, PF04927 and PF00257. In order to identify the LEA proteins in cotton, the HHM profile of LEA protein was subsequently employed as a query to perform a HMMER search (http://hmmer.janelia.org/) [23] against the *G. hirsutum* and *G. arboreum*, which were obtained from cotton genome project (http://www.cgp.genomics.org.cn) and *G. raimondii* genome downloaded from Phytozome (http://www.Phytozome.net/), with E-value <0.01. All redundant sequences were discarded from further analysis based on cluster W^17^ alignment results. SMART and PFAM database were used to verify the presence of the *LEA* gene domains. The isoelectric points and molecular mass of LEA proteins were estimated by ExPASy Server tool (http://web.expasy.org/compute_pi/). In addition, subcellular location prediction of upland cotton, *Gossypium hirsutum* LEA proteins was conducted using the TargetP1.1 (http://www.cbs.dtu.dk/services/TargetP/) server [24] and Protein Prowler Subcellular Localisation Predictor version 1.2 (http://bioinf.scmb.uq.edu.au/pprowler_webapp_1-2/) [25]. Validation and determination of the possible cell compartmentalization of the LEA protein was done by WoLFPSORT (https://wolfpsort.hgc.jp/) [26].

### Chromosomal locations and syntenic analysis

The chromosomal distribution of *LEA* genes were mapped on cotton chromosomes based on gene position, from up down by Circos-0.69 (http://circos.ca/) [27]. Homologous genes of *G. hirsutum*, *G. raimondii* and *G. arboreum* were identified by BLASTP with threshold >80% in similarity and at least in 80% alignment ratio to their protein total lengths. Default parameters were maintained in all of the steps. Tandem duplications were designated as multiple genes of one family located within the same or neighbouring intergenic region [28]. The Ks/Ka value is an important tool in determining selection pressure acting on the protein coding genes. The genes paralogous pair, which has Ka/Ks, ratio greater than 1, denotes activating evolution under beneficial selection, indicating that at least some of the mutations were advantageous. When the ratio is equal to 1, then the mutation is neutral but if the ratio is less than 1, it implies that the mutation is disadvantageous or under purifying selection [29]. In the estimation of Ks and Ka substitution rate, we used an alignment of multiple nucleotide sequences of homologous genes which code for LEA proteins. In this research, paralogous pairs were aligned using MEGA 6.0. synonymous substitution (Ks) and non-synonymous substitution (Ka) rate were obtained by Dnasp [30].

### Phylogenetic analyses, gene structure organization and motif composition of the LEA proteins in cotton

Full-length sequences of *G. hirsutum*, *G. arboreum*, *G. raimondii*, *P. tabuliformis* and *A. thaliana* LEA proteins were first aligned using ClustalW on MEGA 6 software [31] then conducted phylogenetic analyses based on protein sequences, with neighbour joining (NJ) method. Support for each node was tested with 1000 bootstrap replicates. The analysis of phylogenetic tree was carried out on upland cotton, *G. hirsutum*. The gene structures were obtained through comparing the genomic sequences and their predicted coding sequences from the cotton genome project. In addition, MEME (Multiple Expectation Maximization for Motif EliCitation) online program (http:// meme.nbcr.net/meme/cgi-bin/meme.cgi) [32], was used to identify the conserved protein motifs, with maximum number of different motif at 20; the minimum and largest base sequence width of 6 and 50 respectively.

### Prediction of miRNAs targeted *LEA* genes

The miRNA sequences were obtained from miRBase (http://www.mirbase.org/) [33], the Plant miRNA database (http://bioinformatics.cau.edu.cn/PMRD/) [34] and EST database (http://www.ncbi.nlm.nih.gov/ nucest) *LEA* genes targeted by miRNAs were predicted by searching 5′ and 3´ UTRs and the CDS of all *LEA* genes for complementary sequences of the cotton miRNAs using the psRNATarget server with default parameters (http://plantgrn.noble.org/psRNATarget/?function=3) [35].

### Promoter cis-element analysis

Promoter sequences (2 kb upstream of the translation start site) of all *LEA* genes were obtained from the cotton genome project (http://cgp.genomics.org.cn/page/species/index.jsp).Transcriptional response elements of *LEA* genes promoters were predicted using using the PLACE database (http://www.dna.affrc.go.jp/PLACE/signals can.html) [36].

### Gene ontology (GO) annotation

The functional grouping of LEA proteins sequences and the analysis of annotation data were executed using Blast2GO PRO software version 4.1.1 (https://www.blast2go.com). Blast2GO annotation associates genes or transcripts with GO terms using hierarchical terms, cellular component (CC), biological process (BP) and molecular function (MF). Genes were described in three categories of the GO classification terms: molecular function, biological processes and cellular components.

### Plant materials and treatment

One-month-old cotton seedlings of *G. tomentosum*-AD3–00 (P0601211), *G. hirsutum*-CRI-12 (G09091801–2) and their BC_2_F_1_ genotypes, with *G. tomentosum* as the donor and *G. hirsutum* as the recurrent parent were used to examine the expression patterns of the *LEA* genes under drought condition. *G. tomentosum* is drought tolerant genotype while *G. hirsutum* is drought susceptible genotype. The two upland cotton accessions are perennially grown and maintained by our research group, in Sanya Island, Hainan province, China. Plants were grown in boxes, with dimension of 41 × 41 cm, with a depth of 30 cm and with three biological replications in the greenhouse located at the cotton research institute, Chinese Academy of Agricultural Sciences (CAAS), Anyang, Henan province, China. The greenhouse conditions were set with temperature at 23 ± 1 °C and a 14-h light/10-h dark photoperiod. After one month of growth, watering was totally withdrawn from drought treated seedlings but not in control. The samples for RNA extraction were collected at 0, 7 and 14th day of drought stress exposure, for plants under drought and control. Root, stem and leaf were the main organs of target in this study.

### RNA isolation and qRT-PCR verification

RNA extraction kit, EASYspin plus plant RNA kit, obtained from Aid Lab, China was used to extract total RNA from roots, stems and leaves. The quality and concentration of each RNA sample was determined using gel electrophoresis and a NanoDrop 2000 spectrophotometer. Only RNAs which met the criterion 260/280 ratio of 1.8–2.1, 260/230 ratio ≥ 2.0, were used for further analyses and stored at −80 °C. The cotton constitutive *Ghactin7* gene, forward “ATCCTCCGTCTTGACCTTG” and reverse sequence “TGTCCGTCAGGCAACTCAT” was used as a reference gene and specific *LEA* genes primers were used for qRT-PCR validation. The first-strand cDNA synthesis was carried out with TranScript-All-in-One First-Strand cDNA Synthesis SuperMix for qPCR, obtained from TRAN, it was used in accordance with the manufacturer’s instructions. Primer Premier 5 was used to design 43 LEA primers with melting temperatures of 55–60 °C, primer lengths of 18–25 bp, and amplicon lengths of 101–221 bp. Details of the primers are shown in (Additional file 1: Table S1). Fast Start Universal SYBRgreen Master (Rox) (Roche, Mannheim, Germany) was used to perform qRT-PCR in accordance with the manufacturer’s instructions. Reactions were prepared in a total volume of 20 μL, containing 10 μL of SYBR green master mix, 2 μL of cDNA template, 6 μL of ddH2O, and 2 μL of each primer to make a final concentration of 10 μM.

## Results

### Identification of *LEA* genes in cotton

The HMM profile of the Pfam LEA domains (PF3760, PF03168, PF03242, PF02987, PF00477, PF10714, PF00257 and PF 04927) were used as the query to identify *LEA* genes in the cotton genomes. Two hundred and eighty *LEA* genes were identified in upland cotton, *Gossypium hirsutum*, one hundred-seventy *LEA* genes in *G. raimondii* and one hundred-fifty *LEA* genes in *G. arboreum*. All the *LEA* genes were analyzed manually using the SMART and PFAM database (http://pfam.xfam.org/) to verify the presence of the *LEA* gene domain. Finally, 242, 136 and 142 candidate LEA proteins were identified in *G. hirsutum*, *G. arboreum* and *G. raimondii* respectively. All identified *LEA* genes were grouped into eight groups, ranging from LEA 1 to LEA 6, dehydrin and seed maturation protein (SMP). To validate our classification of upland cotton *LEA* genes, we compared the *LEA* genes nomenclature with previous identification adopted by Hundertmark and Hincha [12] and Bies-Etheve et al. [37] (Table 1).

**Table 1:** LEA proteins distribution in upland cotton compared with other plants

*LEA* genes grouping in this study	Pfam	Hundertmark et al. (2008)	Bies-Etheve et al. (2008)	*Arabidopsis*	*G. hirsutum* (AD)	*G. arboreum* (A)	*G. raimondii* (D)	*Pinus tabuliformis*	TOTALS
LEA 1	PF03760	LEA1	LEA4	7	9	4	4	3	27
LEA 2	PF03168	LEA2	LEA7	3	157	85	89	1	335
LEA 3	PF03242	LEA3	LEA6	4	16	6	7	6	39
LEA 4	PF02987	LEA4	LEA3	12	13	8	7	16	56
LEA 5	PF00477	LEA5	LEA1	6	9	7	6	3	31
LEA 6	PF10714	PvLEA18	LEA8	3	4	2	4	0	13
SMP	PF04927	SMP	LEA5	6	10	16	17	0	49
DEHYDRIN	PF00257	Dehydrin	LEA2	10	24	8	8	1	51
TOTALS				51	242	136	142	30	601

The physicochemical parameters of each *LEA* gene were calculated by using ExPASy, an online tool [38]. Most of the LEA proteins in the same family had similar physicochemical parameters. Cotton LEAs of the LEA 4 contained a greater number of amino acid residues as depicted by their protein lengths (aa), followed closely by the dehydrins (Table 2). Dehydrins have been found to contain high number of amino acid residues from the structural analysis of *LEA* genes in *Brassica napus* [39]. Cotton LEA_6 family members all had relatively low molecular masses, ranging from 10.177 to 11.9634 kDa, similar findings was also reported in the analysis of *B. napus LEA* genes, in which all the *LEA 6* genes had lower molecular masses [39]. Approximately two- thirds of the cotton LEA proteins had high isoelectric points Pl ≥ 7.0, including majority of LEA 2 family.

**Table 2:** *LEA* gene in upland cotton, *Gossypium hirsutum* and their sequence characteristics and subcellular location prediction and chromosome position

GENE ID	PROTEIN TYPE	GENE ANNOTATION	LENGTH (aa)	Pl	MM(aa)	Chr NO	Start	End	Position	Sub cellular localization		
										Wolfpsort	Pprowler	TargetP
CotAD_ 04417	DEHYDRIN	DEHYDRIN-4	98	6.33	10,753.6	At_chr08	2,270,863	2,271,159	296	nucl	other	_
CotAD_ 07367	DEHYDRIN	DEHYDRIN-24	1431	6.31	160,755.92	scaffold72.1	2,201,874	2,209,455	7581	plas	other	_
CotAD_ 08352	DEHYDRIN	DEHYDRIN-23	160	5.45	17,797.83	scaffold190.1	910,057	911,647	1590	nucl	other	_
CotAD_ 10,502	DEHYDRIN	DEHYDRIN-8	235	8.18	26,216.65	Dt01_chr15	2,276,230	2,277,176	946	nucl	other	_
CotAD_ 11,398	DEHYDRIN	DEHYDRIN-13	51	6.52	5513.09	Dt06_chr25	776,882	777,140	258	nucl	other	S
CotAD_ 13,947	DEHYDRIN	DEHYDRIN-19	449	4.92	49,643.3	Dt13_chr18	1,193,127	1,196,473	3346	nucl	other	_
CotAD_ 15,928	DEHYDRIN	DEHYDRIN-18	180	7.98	19,341.26	Dt12_chr26	638,305	639,445	1140	nucl	other	_
CotAD_ 16,331	DEHYDRIN	DEHYDRIN-15	128	5.46	14,436.09	Dt08_chr24	877,320	877,836	516	mito	other	_
CotAD_ 19,173	DEHYDRIN	DEHYDRIN-5	180	7.98	19,330.23	At_chr12	883,350	884,512	1162	cyto	other	_
CotAD_ 22,357	DEHYDRIN	DEHYDRIN-2	197	5.48	22,224.51	At_chr02	824,855	825,544	689	chlo	other	_
CotAD_ 27,143	DEHYDRIN	DEHYDRIN-14	135	9.49	14,720.04	Dt07_chr16	115,939	116,435	496	chlo	other	_
CotAD_ 29,610	DEHYDRIN	DEHYDRIN-16	172	5.89	19,224.36	Dt08_chr24	936,161	936,813	652	cyto	other	_
CotAD_ 31,255	DEHYDRIN	DEHYDRIN-10	199	5.5	22,423.72	Dt02_chr14	711,572	712,266	694	chlo	other	_
CotAD_ 35,513	DEHYDRIN	DEHYDRIN-12	161	4.79	17,827.6	Dt05_chr19	393,056	394,685	1629	chlo	other	_
CotAD_ 42,408	DEHYDRIN	DEHYDRIN-20	344	9.01	37,987.42	Dt13_ch18	453,295	454,329	1034	chlo	other	_
CotAD_ 46,550	DEHYDRIN	DEHYDRIN-1	135	9.33	14,717.04	At_chr01	69,396	69,892	496	cyto	other	_
CotAD_ 50,983	DEHYDRIN	DEHYDRIN-9	243	8.82	26,199.73	Dt02_chr14	297,459	298,432	973	cyto	other	_
CotAD_ 53,264	DEHYDRIN	DEHYDRIN-22	211	5.04	23,789.31	scaffold1899.1	95,851	96,576	725	cyto	other	_
CotAD_ 57,587	DEHYDRIN	DEHYDRIN-17	211	5.22	23,654.28	Dt09_chr23	187,778	188,503	725	cyto	other	_
CotAD_ 64,203	DEHYDRIN	DEHYDRIN-6	178	6.48	19,521.14	At_chr13	125,424	126,358	934	chlo	other	_
CotAD_ 65,889	DEHYDRIN	DEHYDRIN-11	608	5.62	67,130.02	Dt03_chr17	396,530	401,988	5458	cyto	other	_
CotAD_ 70,948	DEHYDRIN	DEHYDRIN-21	332	8.46	37,102.53	Dt13_chr18	41,714	42,880	1166	chlo	other	_
CotAD_ 75,267	DEHYDRIN	DEHYDRIN-7	332	8.22	37,096.42	At_chr13	210,365	211,531	1166	nucl	other	_
CotAD_ 75,537	DEHYDRIN	DEHYDRIN-3	533	5.39	58,793.08	At_chr03	254,566	259,519	4953	plas	other	_
CotAD_ 16,594	LEA1	LEA 1–7	115	6.9	13,315.99	Dt13_chr18	1,203,659	1,204,098	439	mito	other	_
CotAD_ 16,595	LEA1	LEA 1–8	115	6.9	13,315.99	Dt13_chr18	1,204,716	1,205,155	439	cyto	other	_
CotAD_ 17,186	LEA1	LEA 1–1	165	5.81	17,459.58	At_chr06	1,273,709	1,274,303	594	plas	other	_
CotAD_ 20,491	LEA1	LEA 1–6	165	6.08	17,343.5	Dt06_chr25	298,147	298,732	585	cyto	other	_
CotAD_ 30,219	LEA1	LEA 1–2	113	6.3	12,106.42	Dt02_chr14	23,536	24,035	499	chlo	other	_
CotAD_ 31,140	LEA1	LEA 1–3	164	9.3	16,871.44	Dt02_chr14	28,053	28,616	563	cyto	other	_
CotAD_ 48,976	LEA1	LEA 1–9	116	8.01	13,437.07	scaffold842.1	284,300	284,742	442	chlo	other	_
CotAD_ 51,667	LEA1	LEA 1–4	164	9.33	16,897.52	Dt02_chr14	443,597	444,162	565	nucl	other	_
CotAD_ 52,203	LEA1	LEA 1–5	420	9.1	45,990.36	Dt02_chr14	444,540	450,345	5805	cyto	other	_
CotAD_ 00275	LEA2	LEA 2–98	274	10.09	29,834.66	Dt09_chr23	2,049,164	2,049,988	824	chlo	other	M
CotAD_ 00465	LEA2	LEA 2–101	304	9.59	33,689.28	Dt09_chr23	3,367,709	3,368,936	1227	chlo	other	M
CotAD_ 00799	LEA2	LEA 2–154	337	8.96	38,982.02	scaffold26.1	2,048,605	2,051,804	3199	golg	other	M
CotAD_ 00808	LEA2	LEA 2–155	226	10.08	26,011.22	scaffold26.1	2,100,130	2,100,810	680	cyto	other	M
CotAD_ 01033	LEA2	LEA 2–105	202	9.06	22,587.14	Dt10_chr20	1,010,984	1,011,592	608	chlo	other	M
CotAD_ 01298	LEA2	LEA 2–107	218	10.22	24,021.4	Dt10_chr20	5,288,414	5,289,070	656	cyto	other	_
CotAD_ 01321	LEA2	LEA 2–108	238	9.54	26,020.28	Dt10_chr20	5,756,514	5,757,230	716	cyto	SP	M
CotAD_ 01385	LEA2	LEA 2–89	247	7	27,497.03	Dt09_chr23	159,994	161,690	1696	cyto	other	_
CotAD_ 01700	LEA2	LEA 2–100	260	9.36	28,399.83	Dt09_chr23	2,815,779	2,816,561	782	cyto	other	_
CotAD_ 02652	LEA2	LEA 2–97	212	10.05	23,764.43	Dt09_chr23	2,032,508	2,033,146	638	mito	other	_
CotAD_ 03037	LEA2	LEA 2–63	262	9.05	28,472.57	Dt05_chr19	1,838,069	1,841,145	3076	cyto	other	_
CotAD_ 03649	LEA2	LEA 2–34	320	9.82	35,345.6	At_chr09	1,775,719	1,776,844	1125	cyto	other	_
CotAD_ 03784	LEA2	LEA 2–75	116	6.82	13,537.66	Dt07_chr16	548,573	548,923	350	chlo	other	_
CotAD_ 05724	LEA2	LEA 2–32	197	10.05	22,442.51	At_chr09	1,755,547	1,756,140	593	chlo	other/SP	_
CotAD_ 05725	LEA2	LEA 2–33	238	9.73	27,552.78	At_chr09	1,759,512	1,760,228	716	nucl	SP	_
CotAD_ 06037	LEA2	LEA 2–115	205	10.07	22,125.81	Dt13_ch18	90,455	91,072	617	chlo	SP	_
CotAD_ 07087	LEA2	LEA2–3	206	9.75	22,853.64	At_chr02	2,101,730	2,102,350	620	plas	other	_
CotAD_ 08181	LEA2	LEA 2–99	202	8.61	22,460.02	Dt09_chr23	2,228,567	2,229,175	608	cyto	SP	_
CotAD_ 08350	LEA2	LEA 2–152	198	5.02	22,266.98	scaffold190.1	904,483	905,252	769	chlo	SP	_
CotAD_ 08837	LEA2	LEA 2–125	245	8.77	26,376.34	scaffold280.1	51,952	53,959	2007	golg	other	_
CotAD_ 09578	LEA2	LEA 2–30	260	9.39	28,406.84	At_chr09	1,173,118	1,173,900	782	chlo	other	_
CotAD_ 09685	LEA2	LEA 2–93	251	10.07	27,153.8	Dt09_chr23	686,730	687,485	755	chlo	SP	C
CotAD_ 09732	LEA2	LEA 2–96	232	9.44	25,906.5	Dt09_chr23	1,174,994	1,175,943	949	chlo	other	_
CotAD_ 10,376	LEA2	LEA 2–48	277	9.92	30,152.74	Dt01_chr15	100,033	100,866	833	chlo	SP	M
CotAD_ 11,658	LEA2	LEA 2–84	263	9.82	29,835.19	Dt08_chr24	199,067	199,858	791	cyto	SP	M
CotAD_ 11,875	LEA2	LEA 2–147	175	6.95	20,070.28	scaffold42.1	647,003	647,530	527	chlo	SP	M
CotAD_ 11,876	LEA2	LEA 2–148	209	10.01	23,563.32	scaffold42.1	677,153	677,782	629	chlo	SP	_
CotAD_ 11,878	LEA2	LEA 2–149	226	9.49	25,841.73	scaffold42.1	688,643	689,323	680	chlo	SP	_
CotAD_ 11,879	LEA2	LEA 2–150	129	9.45	15,037.05	scaffold42.1	690,007	690,396	389	chlo	SP	_
CotAD_ 12,375	LEA2	LEA 2–25	190	8.59	21,328.78	At_chr09	106,696	107,358	662	chlo	SP	_
CotAD_ 13,115	LEA2	LEA 2–86	192	9.42	20,770.35	Dt08_chr24	1,183,052	1,183,630	578	extr	SP	_
CotAD_ 13,584	LEA2	LEA 2–67	250	9.96	28,048.83	Dt06_chr25	364,402	365,154	752	golg	SP	_
CotAD_ 13,827	LEA2	LEA 2–114	360	7.87	40,945.87	Dt12_chr26	971,427	972,748	1321	E.R.	mTP	C
CotAD_ 14,147	LEA2	LEA 2–16	212	9.94	23,855.54	At_chr07	774,151	774,789	638	mito	SP	S
CotAD_ 15,892	LEA2	LEA 2–112	307	7.7	34,741.21	Dt12_chr26	406,661	409,153	2492	chlo	mTP	_
CotAD_ 16,731	LEA2	LEA 2–94	258	10.01	28,519.44	Dt09_chr23	724,944	725,720	776	chlo	other	_
CotAD_ 17,044	LEA2	LEA 2–17	151	4.84	16,422.87	At_chr07	972,098	972,634	536	cyto	SP	S
CotAD_ 17,045	LEA2	LEA 2–18	219	9.79	23,930.18	At_chr07	992,750	993,409	659	cyto	SP	_
CotAD_ 17,062	LEA2	LEA 2–19	244	9.78	27,393.16	At_chr07	1,176,161	1,176,895	734	chlo	mTP	C
CotAD_ 17,101	LEA2	LEA 2–9	222	9.26	25,294.09	At_chr06	94,601	95,269	668	mito	SP	S
CotAD_ 17,102	LEA2	LEA 2–10	209	10.35	23,661.48	At_chr06	122,145	122,774	629	nucl	SP	_
CotAD_ 17,103	LEA2	LEA 2–11	265	6.7	30,299.29	At_chr06	134,827	135,702	875	mito	SP	C
CotAD_ 17,649	LEA2	LEA 2–37	235	8.5	26,726.9	At_chr10	359,189	361,440	2251	chlo	SP	_
CotAD_ 18,210	LEA2	LEA 2–141	203	10.17	22,501.33	scaffold377.1	414,366	414,977	611	cyto	SP	C
CotAD_ 18,233	LEA2	LEA 2–145	203	10.08	22,406.26	scaffold377.1	560,351	560,962	611	chlo	SP	_
CotAD_ 18,546	LEA2	LEA 2–95	173	9.91	19,695.85	Dt09_chr23	893,109	893,715	606	chlo	mTP	M
CotAD_ 18,729	LEA2	LEA 2–142	277	9.92	30,227.97	scaffold336.1	433,013	433,846	833	chlo	SP	_
CotAD_ 19,078	LEA2	LEA 2–42	216	9.83	24,007.7	At_chr12	18,103	18,753	650	nucl	other	_
CotAD_ 19,107	LEA2	LEA 2–43	183	9.04	20,031.24	At_chr12	312,977	313,528	551	chlo	SP	C
CotAD_ 19,205	LEA2	LEA 2–46	297	6.83	33,395.7	At_chr12	1,142,954	1,145,475	2521	chlo	SP	S
CotAD_ 19,213	LEA2	LEA 2–38	100	9.64	11,538.35	At_chr10	410,401	410,703	302	chlo	mTP	_
CotAD_ 19,214	LEA2	LEA 2–39	181	9.32	20,628.72	At_chr10	411,491	412,036	545	nucl	SP	S
CotAD_ 19,375	LEA2	LEA 2–111	225	8.57	25,956.2	Dt11_chr21	1,023,165	1,023,842	677	golg	mTP	C
CotAD_ 20,020	LEA2	LEA 2–13	250	9.89	27,947.68	At_chr06	997,148	997,900	752	mito	SP	_
CotAD_ 20,308	LEA2	LEA 2–72	191	9.56	21,054.44	Dt06_chr25	1,390,037	1,390,612	575	chlo	cTP	C
CotAD_ 21,731	LEA2	LEA 2–62	244	9.83	27,381.21	Dt05_chr19	1,524,633	1,525,367	734	nucl	other	_
CotAD_ 21,924	LEA2	LEA 2–110	262	10.16	28,411.4	Dt11_chr21	855,130	855,918	788	nucl	SP	S
CotAD_ 23,646	LEA2	LEA 2–74	204	9.81	21,921.93	Dt07_chr16	34,227	34,841	614	nucl	SP	_
CotAD_ 24,019	LEA2	LEA 2–71	203	10.04	22,391.06	Dt06_chr25	652,496	653,107	611	mito	SP	S
CotAD_ 24,497	LEA2	LEA 2–106	263	8.64	29,247.79	Dt10_chr20	1,715,959	1,719,093	3134	chlo	other	_
CotAD_ 24,499	LEA2	LEA 2–138	175	7.66	20,026.25	scaffold238.1	343,833	344,360	527	chlo	SP	_
CotAD_ 25,271	LEA2	LEA 2–139	209	10.01	23,559.33	scaffold238.1	356,524	357,153	629	nucl	SP	_
CotAD_ 26,038	LEA2	LEA 2–140	226	9.43	25,852.71	scaffold238.1	383,318	383,998	680	chlo	SP	S
CotAD_ 26,981	LEA2	LEA 2–26	274	10.09	29,936.66	At_chr09	239,371	240,195	824	chlo	other	_
CotAD_ 27,453	LEA2	LEA 2–131	239	9.76	26,994.13	scaffold477.1	176,874	179,526	2652	mito	SP	_
CotAD_ 27,789	LEA2	LEA 2–151	184	9.41	20,135.39	scaffold699.1	759,396	759,950	554	E.R.	SP	M
CotAD_ 28,249	LEA2	LEA 2–27	150	9.24	16,764.6	At_chr09	274,309	275,011	702	nucl	SP	_
CotAD_ 28,252	LEA2	LEA 2–13	222	8.65	24,982.77	At_chr07	296,696	299,063	2367	mito	other	_
CotAD_ 28,872	LEA2	LEA 2–57	257	9.1	26,949.97	Dt03_chr17	1,936,828	1,937,663	835	nucl	SP	_
CotAD_ 29,279	LEA2	LEA 2–116	305	9.66	34,588.47	Dt13_chr18	639,522	641,549	2027	chlo	SP	_
CotAD_ 31,344	LEA2	LEA 2–132	101	5.51	11,711.01	scaffold1346.1	193,028	193,333	305	chlo	other	_
CotAD_ 31,535	LEA2	LEA 2–8	240	7.89	27,649.86	At_chr05	790,866	791,588	722	vacu	other	_
CotAD_ 31,536	LEA2	LEA 2–136	210	9.19	23,875.63	scaffold1346.1	213,521	214,153	632	plas	SP	_
CotAD_ 31,537	LEA2	LEA 2–133	254	10.22	27,558.52	scaffold1841.1	200,526	201,290	764	nucl	cTP	C
CotAD_ 31,780	LEA2	LEA 2–87	310	9.93	34,525.38	Dt08_chr24	1,487,296	1,488,516	1220	chlo	other	_
CotAD_ 31,782	LEA2	LEA 2–90	210	7.72	23,638.39	Dt09_chr23	194,606	195,238	632	chlo	SP	_
CotAD_ 31,860	LEA2	LEA 2–153	206	9.82	22,839.69	scaffold257.1	1,162,406	1,163,026	620	cyto	SP	_
CotAD_ 31,906	LEA2	LEA 2–137	232	9.66	26,256.38	scaffold769.1	292,760	295,431	2671	cyto	mTP	M
CotAD_ 31,936	LEA2	LEA 2–53	152	4.74	16,462.97	Dt01_chr15	598,039	598,839	800	mito	SP	_
CotAD_ 32,487	LEA2	LEA 2–36	305	9.97	33,718.76	At_chr11	169,902	171,217	1315	mito	other	_
CotAD_ 32,645	LEA2	LEA 2–66	199	9.3	22,785.41	Dt06_chr25	246,850	247,449	599	chlo	SP	C
CotAD_ 32,847	LEA2	LEA 2–24	249	9.79	27,707.74	At_chr09	61,155	61,904	749	extr	cTP	C
CotAD_ 33,143	LEA2	LEA 2–54	305	9.63	34,544.43	Dt02_chr14	1,894,174	1,896,197	2023	chlo	SP	_
CotAD_ 33,144	LEA2	LEA 2–60	240	8.49	27,655.92	Dt05_chr19	151,373	152,095	722	chlo	SP	S
CotAD_ 34,476	LEA2	LEA 2–92	320	9.92	35,579.84	Dt09_chr23	448,827	449,952	1125	cyto	SP	_
CotAD_ 34,798	LEA2	LEA 2–68	222	9.23	25,253.03	Dt06_chr25	385,794	386,462	668	nucl	SP	_
CotAD_ 35,069	LEA2	LEA 2–69	209	10.25	23,628.4	Dt06_chr25	396,513	397,142	629	chlo	SP	_
CotAD_ 35,091	LEA2	LEA 2–70	288	7.1	32,755.52	Dt06_chr25	403,729	404,595	866	extr	SP	_
CotAD_ 35,514	LEA2	LEA 2–61	206	5.9	23,420.27	Dt05_chr19	399,904	400,524	620	mito	SP	C
CotAD_ 36,328	LEA2	LEA 2–144	450	4.92	49,131.5	scaffold821.1	548,888	550,240	1352	chlo	other	_
CotAD_ 36,446	LEA2	LEA 2–78	231	9.47	24,949.39	Dt08_chr24	58,782	59,477	695	chlo	other	_
CotAD_ 36,583	LEA2	LEA 2–146	206	8.88	22,761.2	scaffold821.1	625,818	626,438	620	chlo	other	_
CotAD_ 37,776	LEA2	LEA 2–91	202	9.02	22,357.93	Dt09_chr23	337,931	338,539	608	chlo	SP	S
CotAD_ 37,888	LEA2	LEA 2–21	283	10.15	31,410.18	At_chr08	2,313,418	2,314,578	1160	chlo	other	_
CotAD_ 38,978	LEA2	LEA 2–85	210	9.76	22,644.27	Dt08_chr24	376,609	377,241	632	nucl	SP	S
CotAD_ 39,064	LEA2	LEA 2–50	210	9.48	23,699.74	Dt01_chr15	220,837	221,469	632	chlo	other	_
CotAD_ 39,719	LEA2	LEA 2–52	191	6.29	20,961.07	Dt01_chr15	397,156	397,731	575	nucl	mTP	_
CotAD_ 40,324	LEA2	LEA 2–15	204	9.81	21,780.76	At_chr07	720,430	721,044	614	plas	SP	_
CotAD_ 41,569	LEA2	LEA 2–47	208	10.19	22,559.45	At_chr13	343,514	344,140	626	chlo	other	_
CotAD_ 41,571	LEA2	LEA 2–88	270	9.56	30,627.54	Dt09_chr23	64,512	65,324	812	chlo	SP	_
CotAD_ 41,925	LEA2	LEA 2–128	188	9.22	21,941.4	scaffold1231.1	94,270	94,836	566	nucl	other	_
CotAD_ 42,599	LEA2	LEA 2–129	373	9.9	43,118.75	scaffold1231.1	96,297	98,517	2220	cyto	Other	M
CotAD_ 44,357	LEA2	LEA 2–143	210	9.34	23,874.6	scaffold1088.1	451,853	452,485	632	cyto	other	C
CotAD_ 45,324	LEA2	LEA 2–109	256	9.99	28,431.93	Dt11_chr21	55,317	61,829	6512	chlo	other	_
CotAD_ 46,873	LEA2	LEA 2–29	259	10	28,603.52	At_chr09	355,476	356,255	779	vacu	other	_
CotAD_ 47,322	LEA2	LEA2–5	220	9.85	24,666.72	At_chr03	430,461	431,123	662	chlo	SP	_
CotAD_ 47,454	LEA2	LEA 2–130	661	6.14	73,583.12	scaffold1851.1	116,914	132,924	16,010	cysk	SP	C
CotAD_ 47,495	LEA2	LEA 2–76	318	10.09	35,234.15	Dt07_chr16	1,185,327	1,186,479	1152	chlo	SP	S
CotAD_ 47,749	LEA2	LEA 2–77	251	9.41	27,769.63	Dt07_chr16	1,400,323	1,401,078	755	chlo	mTP	M
CotAD_ 48,050	LEA2	LEA 2–103	217	9.28	24,968.87	Dt10_chr20	968,935	969,588	653	mito	other	_
CotAD_ 48,069	LEA2	LEA 2–104	181	9.57	20,577.73	Dt10_chr20	970,347	970,892	545	extr	SP	S
CotAD_ 48,336	LEA2	LEA 2–58	211	9.12	23,479.93	Dt04_chr22	552,418	553,053	635	nucl	SP	_
CotAD_ 48,753	LEA2	LEA 2–12	210	9.28	23,676.69	At_chr06	482,445	483,077	632	mito	SP	_
CotAD_ 48,769	LEA2	LEA 2–28	304	9.56	33,675.21	At_chr09	334,020	335,245	1225	nucl	SP	_
CotAD_ 49,818	LEA2	LEA 2–119	317	4.63	35,274.16	scaffold2616.1	21,219	22,172	953	cyto	SP	_
CotAD_ 53,045	LEA2	LEA 2–102	206	7.58	22,650.27	Dt10_chr20	363,682	364,302	620	cyto	cTP	C
CotAD_ 53,263	LEA2	LEA 2–23	251	10.11	27,168.81	At_chr09	24,397	25,152	755	chlo	SP	_
CotAD_ 53,981	LEA2	LEA 2–123	247	6.59	27,715.29	scaffold3326.1	42,209	43,944	1735	mito	cTP	C
CotAD_ 54,337	LEA2	LEA 2–14	152	4.84	16,453.02	At_chr07	366,521	367,321	800	chlo	SP	S
CotAD_ 55,224	LEA2	LEA 2–55	210	9.66	23,769.83	Dt03_chr17	607,531	608,163	632	mito	SP	M
CotAD_ 56,356	LEA2	LEA 2–22	173	9.96	19,737.98	At_chr09	18,712	19,318	606	chlo	SP	S
CotAD_ 56,696	LEA2	LEA 2–56	213	9.51	23,750.48	Dt03_chr17	634,717	635,358	641	nucl	SP	_
CotAD_ 58,358	LEA2	LEA 2–113	209	10.19	23,626.51	Dt12_ch26	897,133	897,762	629	chlo	SP	S
CotAD_ 59,405	LEA2	LEA 2–61	320	9.9	35,457.72	Dt05_chr19	251,378	252,501	1123	chlo	SP	S
CotAD_ 60,279	LEA2	LEA 2–124	247	8.76	26,619.63	scaffold2414.1	50,037	52,048	2011	chlo	SP	_
CotAD_ 60,435	LEA2	LEA2–1	251	9.57	27,952.81	At_chr01	137,428	138,183	755	chlo	cTP	C
CotAD_ 60,617	LEA2	LEA 2–49	210	9.51	23,780.9	Dt01_chr15	198,189	198,821	632	mito	SP	_
CotAD_ 61,173	LEA2	LEA2–7	215	9.84	24,043	At_chr04	59,864	60,511	647	chlo	cTP	_
CotAD_ 61,391	LEA2	LEA 2–51	191	6.29	20,884.97	Dt01_chr15	284,374	284,949	575	chlo	SP	C
CotAD_ 62,996	LEA2	LEA2–2	318	9.95	35,356.25	At_chr01	176,895	178,045	1150	nucl	SP	_
CotAD_ 63,174	LEA2	LEA 2–117	377	9.77	41,228.93	scaffold3177.1	19,137	21,221	2084	E.R.	SP	C
CotAD_ 64,004	LEA2	LEA 2–73	219	9.65	23,825.02	Dt07_chr16	34,198	34,857	659	chlo	other	_
CotAD_ 64,120	LEA2	LEA 2–41	218	10.14	24,050.43	At_chr12	951	1607	656	chlo	SP	_
CotAD_ 64,346	LEA2	LEA 2–64	210	8.99	23,572.5	Dt06_chr25	59,643	60,275	632	chlo	SP	_
CotAD_ 64,347	LEA2	LEA 2–65	235	9.44	26,111.93	Dt06_chr25	62,524	63,231	707	plas	cTP	C
CotAD_ 64,657	LEA2	LEA 2–40	262	10.22	28,516.58	At_chr11	144,295	145,083	788	vacu	SP	_
CotAD_ 65,119	LEA2	LEA 2–79	206	8.88	22,733.19	Dt08_chr24	59,660	60,280	620	golg	SP	_
CotAD_ 65,370	LEA2	LEA 2–126	326	9.99	36,098.18	scaffold3528.1	84,696	86,784	2088	chlo	other	_
CotAD_ 66,245	LEA2	LEA 2–82	450	4.94	48,836.2	Dt08_chr24	121,249	122,601	1352	chlo	other	_
CotAD_ 66,538	LEA2	LEA2–6	211	9.47	23,424.96	At_chr04	59,282	59,917	635	chlo	SP	_
CotAD_ 66,551	LEA2	LEA 2–118	225	9.28	25,226.24	scaffold3976.1	20,354	21,031	677	cyto	other	_
CotAD_ 66,774	LEA2	LEA 2–80	216	9.92	24,090.84	Dt08_chr24	68,424	69,074	650	chlo	SP	_
CotAD_ 66,775	LEA2	LEA 2–81	225	9.61	25,078.29	Dt08_chr24	72,945	73,622	677	chlo	SP	S
CotAD_ 67,823	LEA2	LEA 2–20	222	9.49	23,928.26	At_chr08	132,953	133,621	668	cyto	SP	S
CotAD_ 68,063	LEA2	LEA2–4	218	9.3	23,245.72	At_chr03	191,498	192,154	656	cyto	SP	_
CotAD_ 68,189	LEA2	LEA 2–35	206	6.71	22,579.21	At_chr10	67,607	68,227	620	chlo	cTP	C
CotAD_ 69,737	LEA2	LEA 2–134	213	9.75	23,867.69	scaffold2095.1	202,243	202,884	641	chlo	SP	S
CotAD_ 69,738	LEA2	LEA 2–135	210	9.88	23,893.04	scaffold2095.1	208,893	209,525	632	chlo	SP	_
CotAD_ 70,003	LEA2	LEA 2–42	191	9.63	20,942.44	At_chr12	171,793	172,368	575	cyto	cTP	C
CotAD_ 70,190	LEA2	LEA 2–120	430	4.81	48,185.02	scaffold4817.1	31,921	37,140	5219	cyto	other	_
CotAD_ 70,192	LEA2	LEA 2–122	130	4.74	14,420.49	scaffold4817.1	38,397	38,789	392	nucl	SP	M
CotAD_ 71,431	LEA2	LEA 2–59	186	9.58	20,579.98	Dt05_chr19	65,530	66,090	560	extr	other	_
CotAD_ 72,458	LEA2	LEA 2–127	192	9.54	20,613.31	scaffold3083.1	91,828	92,406	578	cysk	SP	_
CotAD_ 72,913	LEA2	LEA 2–121	315	4.63	35,071.89	scaffold4398.1	34,689	36,390	1701	cysk	SP	_
CotAD_ 73,966	LEA2	LEA 2–45	320	9.96	35,484.73	At_chr12	365,460	366,583	1123	chlo	other	_
CotAD_ 74,713	LEA2	LEA 2–83	211	9.12	23,479.93	Dt08_chr24	158,342	158,977	635	golg	other	_
CotAD_ 76,129	LEA2	LEA 2–44	209	10.19	23,626.51	At_chr12	317,009	317,638	629	chlo	other	_
CotAD_ 01504	LEA3	LEA 3–13	93	8.82	10,469.06	Dt09_chr23	1,171,108	1,171,516	408	chlo	other	C
CotAD_ 04558	LEA3	LEA 3–1	100	9.75	10,627.15	At_chr04	288,394	288,796	402	chlo	mTP	S
CotAD_ 04559	LEA3	LEA 3–2	100	9.34	10,419.9	At_chr04	291,007	291,400	393	chlo	SP	S
CotAD_ 21,416	LEA3	LEA 3–9	92	9.83	9667.02	Dt04_chr22	127,037	127,390	353	chlo	mTP	C
CotAD_ 22,634	LEA3	LEA 3–11	100	9.18	10,503.02	Dt04_chr22	853,079	853,496	417	mito	SP	S
CotAD_ 23,118	LEA3	LEA 3–12	99	9.56	10,350.8	Dt04_chr22	855,441	855,830	389	cyto	mTP	S
CotAD_ 24,498	LEA3	LEA 3–8	126	6.27	13,502.83	Dt03_chr17	1,079,773	1,082,222	2449	chlo	mTP	M
CotAD_ 26,668	LEA3	LEA 3–16	98	9.34	10,595.99	scaffold141.1	891,415	891,795	380	chlo	mTP	M
CotAD_ 33,003	LEA3	LEA 3–15	120	7.02	13,729.35	scaffold944.1	428,674	429,107	433	chlo	mTP	M
CotAD_ 35,021	LEA3	LEA 3–5	85	9.77	9781.28	At_chr11	537,390	537,732	342	chlo	mTP	M
CotAD_ 36,999	LEA3	LEA 3–4	126	6.27	13,484.86	At_chr08	1,181,217	1,183,629	2412	cyto	mTP	M
CotAD_ 40,972	LEA3	LEA 3–7	124	9.51	14,155.5	At_chr13	122,903	123,887	984	chlo	mTP	M
CotAD_ 41,714	LEA3	LEA 3–14	105	9.3	11,363.76	Dt11_chr21	445,383	445,797	414	chlo	mTP	M
CotAD_ 43,605	LEA3	LEA 3–3	92	9.75	9709.1	At_chr04	475,252	475,623	371	nucl	mTP	C
CotAD_ 46,270	LEA3	LEA 3–6	105	9.51	11,449.87	At_chr11	673,169	673,590	421	golg	mTP	S
CotAD_ 56,728	LEA3	LEA 3–10	98	9.66	10,549.99	Dt04_chr22	520,431	520,811	380	golg	mTP	S
CotAD_ 00667	LEA4	LEA 4–12	239	9.03	26,335.09	scaffold26.1	961,112	961,910	798	chlo	cTP	C
CotAD_ 02872	LEA4	LEA 4–4	569	5.89	62,916.57	Dt05_chr19	496,264	498,058	1794	mito	other	_
CotAD_ 05963	LEA4	LEA 4–5	266	5.2	29,264.99	Dt05_chr19	2,777,842	2,778,935	1093	extr	SP	S
CotAD_ 09404	LEA4	LEA 4–7	127	9.21	13,553.37	Dt07_chr16	2,927,050	2,928,024	974	chlo	cTP	C
CotAD_ 09405	LEA4	LEA 4–8	109	9.96	12,066.81	Dt07_chr16	2,946,007	2,947,047	1040	chlo	mTP	M
CotAD_ 10,044	LEA4	LEA 4–3	634	5.78	68,352.06	At_chr07	513,343	515,454	2111	nucl	mTP	_
CotAD_ 13,989	LEA4	LEA 4–10	109	10.04	12,094.87	Dt13_chr18	1,907,725	1,908,766	1041	chlo	mTP	M
CotAD_ 22,633	LEA4	LEA 4–6	136	6.93	14,614.04	Dt06_chr25	240,527	241,024	497	chlo	other	_
CotAD_ 23,824	LEA4	LEA 4–9	405	5.88	44,549.64	Dt12_chr26	2,459,614	2,460,913	1299	chlo	other	_
CotAD_ 50,359	LEA4	LEA4–1	284	5.13	31,311.17	At_chr03	809,118	810,165	1047	cyto	SP	S
CotAD_ 62,314	LEA4	LEA 4–11	239	9.03	26,258.97	scaffold3310.1	9965	10,763	798	cyto	cTP	C
CotAD_ 62,659	LEA4	LEA4–2	568	5.96	62,738.55	At_chr06	14,807	16,597	1790	cyto	other	_
CotAD_ 74,061	LEA4	LEA 4–4	405	5.69	44,521.59	At_chr12	408,138	409,436	1298	nucl	other	_
CotAD_ 03264	LEA5	LEA 5–4	110	5.55	11,915.92	Dt06_chr25	1,093,153	1,093,598	445	nucl	other	_
CotAD_ 07516	LEA5	LEA 5–2	123	5.78	13,999.94	At_chr09	1,687,267	1,687,988	721	cyto	other	_
CotAD_ 22,539	LEA5	LEA 5–9	102	5.49	11,072.09	scaffold613.1	610,955	611,364	409	chlo	other	_
CotAD_ 31,869	LEA5	LEA 5–8	102	5.49	11,072.09	scaffold1551.1	323,034	323,444	410	nucl	other	_
CotAD_ 33,321	LEA5	LEA 5–7	110	5.55	11,972.03	scaffold1788.1	214,115	214,558	443	chlo	other	_
CotAD_ 46,888	LEA5	LEA 5–1	144	7.76	16,526.85	At_chr08	563,572	564,774	1202	chlo	other	_
CotAD_ 48,469	LEA5	LEA 5–5	171	8.39	19,503.27	Dt08_chr24	159,604	160,805	1201	nucl	other	_
CotAD_ 56,699	LEA5	LEA 5–6	94	8.1	10,073.96	Dt10_chr20	81,020	81,398	378	chlo	other	_
CotAD_ 57,519	LEA5	LEA 5–3	94	8.1	10,073.96	At_chr12	153,745	154,123	378	vacu	other	_
CotAD_ 13,789	LEA6	LEA 6–3	86	7.8	9580.48	Dt12_chr26	651,517	651,777	260	nucl	other	_
CotAD_ 19,623	LEA6	LEA6–1	94	4.76	10,176.98	At_chr01	1,336,340	1,336,624	284	extr	other	_
CotAD_ 44,941	LEA6	LEA 6–4	114	11.83	11,963.43	scaffold3339.1	62,119	62,571	452	cyto	other	_
CotAD_ 53,438	LEA6	LEA 6–2	94	4.75	10,257.11	Dt07_chr16	78,654	78,938	284	chlo	SP	M
CotAD_ 11,594	SMP	SMP-10	264	4.85	26,898.86	scaffold189.1	992,496	993,467	971	cyto	other	_
CotAD_ 12,680	SMP	SMP-3	169	4.63	17,180.76	At_chr07	1,633,754	1,634,391	637	cyto	other	_
CotAD_ 12,681	SMP	SMP-4	144	4.61	14,950.53	At_chr07	1,636,123	1,636,635	512	chlo	other	_
CotAD_ 12,682	SMP	SMP-5	258	4.56	26,168.03	At_chr07	1,639,467	1,640,458	991	cyto	other	_
CotAD_ 39,233	SMP	SMP-7	171	4.49	17,755.88	Dt13_chr18	1,963,715	1,964,463	748	chlo	other	_
CotAD_ 43,455	SMP	SMP-6	261	4.79	26,971.01	Dt12_chr26	1,316,599	1,317,706	1107	chlo	other	_
CotAD_ 45,390	SMP	SMP-1	252	6.35	26,222.21	At_chr01	335,790	337,578	1788	chlo	other	_
CotAD_ 51,205	SMP	SMP-9	253	6.46	26,151.04	scaffold1984.1	277,433	279,262	1829	chlo	other	_
CotAD_ 66,708	SMP	SMP-2	258	6.44	27,923.82	At_chr04	123,169	126,337	3168	cyto	other	_
CotAD_ 67,721	SMP	SMP-8	264	4.93	26,885.82	scaffold4155.1	34,277	35,248	971	cyto	other	_

LEA: late embryogenesis abundant protein; LEA1, 2, 3, 4, 5 and 6 indicates the sub families of LEA proteins while the −1, −2,-3…. Represents the protein annotation number i.e. LEA1–1, the first member of LEA1 sub family; SMP: seed maturation protein; chlo: chloroplast; cyto: cytoplasm; extr: extracellular part of the cell; nucl: nucleus; mito: mitochondrion; cysk: cytoskeleton; golg: golgi body; vacu: vacuole; plas: plasma membrane; E.R: endoplasmic reticulum; SP: Secretory pathway (presence of a signal peptide); mTP: mitochondrial targeting peptide; cTP: chloroplast transit peptide; Other (nucleus, cytoplasmic, or otherwise). C: cytoplasm; S: secretory pathway; M: mitochondrion and -: others/other cell organelles; chr: chromosome; Dt: sub genome D and At: sub-genome A

The only LEA proteins with all its members having Pl < 7, were the SMPs, this results is in agreement to Pl values obtained for SMPs in *Brassica napus*, all had Pl < 7.0 [39]. The grand average of hydropathy (GRAVY) results as obtained from ExPASy indicated that cotton LEA 2 proteins are the most hydrophobic, with all except three with GRAVY values <0. The rest of the LEA proteins were highly hydrophilic, with almost all of the groups had gravy value of less than 0, these results are consistent with those of the LEA proteins in *Arabidopsis thaliana* [40]. Low hydrophobicity and high net charge are the main characteristics of other LEA proteins [38] which enables them to be totally or partially disordered, this unique features is an attribute which gives the LEA proteins the ability to form flexible structural elements such as molecular chaperones which are integral for the protection of plants from desiccation effects [41]. TargetP1.1 (http://www.cbs.dtu.dk/services/TargetP/) server [24] and Protein Pprowler Subcellular Localisation Predictor version 1.2 (http://bioinf.scmb.uq.edu.au/Pprowler_webapp_1–2/) [25], were used to predict the subcellular location of 242 *Gossypium hirsutum* LEA proteins, most of the LEA proteins were predicted to participate in the secretory pathway, same as the *Brassica napus* LEA proteins [39] (Table 2 and Additional file 2: Table S2).

We further used WoLFPSORT [26] to investigate the particular cell compartments in which the LEA proteins were embedded in, 148 *LEA* genes were predicted to be chloroplasts proteins, 47 as cytoplasm proteins, 20 as mitochondrion proteins, 35 as nucleus proteins, 11 as Golgi body proteins, 7 as extracellular proteins, 7 as plasma proteins, 4 as vacuole proteins and 3 as endoplasmic reticulum proteins. The details of other characteristics of the nucleic acid and protein sequences are provided in (Table 2). *LEA* genes have ubiquitous distribution across cell compartments with unique subcellular localization [42]. LEA 4 gene families were found to be widely distributed in cell structures such as cytosol, mitochondria, plastid, ER, and pexophagosome [42]. The unique and wide distribution of *LEA* genes within the various cell structures is to establish interactions with various cellular membranes under stress conditions. The broad subcellular distribution of LEA proteins highlights the requirement for each cellular compartment to be provided with protective mechanisms to cope with drought stress [17]. In Summary, both experimental and prediction data indicates that LEA proteins have wide distribution in subcellular compartments [42].

### Phylogenetic analyses, gene structure and protein motifs of *LEA* genes in upland cotton

**Fig. 1:**
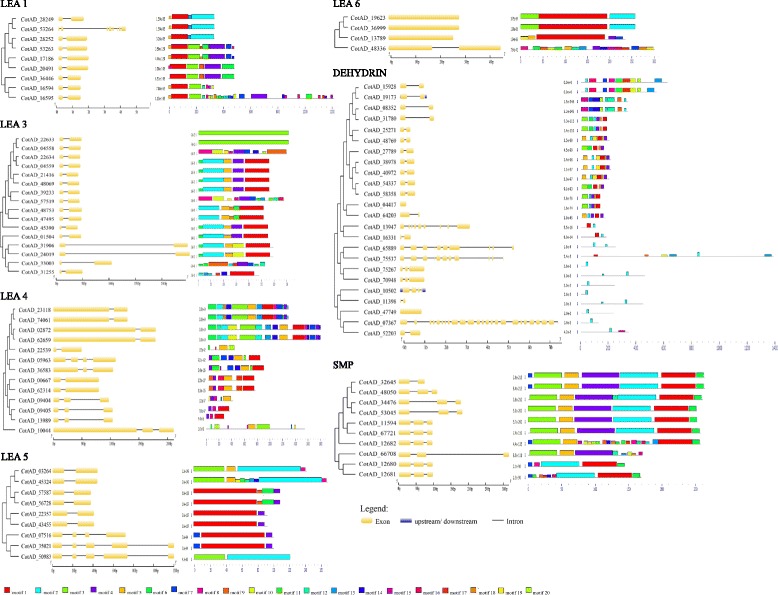
Phylogenetic tree, gene structure and motif compositions of *LEA* genes in upland cotton. The phylogenetic tree was constructed using MEGA 6.0. Exon/intron structures of *LEA* genes in upland cotton, exons introns and up / down-stream were represented by yellow boxes, black lines and blue boxes, respectively. Protein motif analysis represented by different colours, and each motif represented by number

To examine the evolutionary history and relationships of *LEA* protein families, an unrooted phylogenetic tree was constructed from alignments of the full lengths of *LEA* gene sequences with Neighbor-joining method based on similarities of the *LEA* genes in upland cotton, *G. hirsutum*. We constructed phylogenetic tree of all the groups of *LEA* genes separately, which we further combined with intron-exon and motifs to unearth more information about phylogenetic tree and *LEA* genes similarities (Fig. 1). Gene structural diversity and conserved motif divergence are possible mechanisms for the evolution of multigene families [43]. To gain further information into the structural diversity of cotton *LEA* genes, we analyzed the exon / intron organization in the full-length cDNAs with their corresponding genomic DNA sequences of individual *LEA* genes in cotton (Fig. 1). Most closely related *LEA* gene members within the same groups shared similar gene structures in terms of either intron numbers or exon lengths. For example, LEA 1,3,4,5, SMP and dehydrins genes had one to four introns with exception of LEA 2 and 6, which had zero to five introns. This result is in agreement with earlier finding in which dehydrin were found to have introns [44]. By contrast, the gene structure appeared to be more variable in LEA 2 which had the largest number of genes, with sizes of exon/intron structure variants with striking distinctions (Additional file 3: Figure S1). The result suggest the divergence functions of this group of protein family in upland cotton.

Twenty-five distinct motifs were identified. Motifs 1, 2, 3, 4, 5 and 6 were common among all the different groups of *LEA* genes, similar motifs have been previously identified in other plant species, including maize [45], *Arabidopsis thaliana* [40], tomato [46] among other plants. Motif analysis of the *cotton LEA* proteins showed that members of each LEA group possess several group-specific conserved motifs (Table 3). Similar features have been reported for LEA proteins in *Solanum lycopersicum* [46], *Arabidopsis* [40], *Prunus* [47] and poplar [48]. For example, a distinctive and conserved motif in the dehydrin group is the repeated motif, EKKGIMDKIKEKLPG (motif K, richness in lysine residues), in this study, we identified a unique motif among the dehydrin families, GEGREKKGFLEKIKEKLPGHHKKTEEAS, which we named as K1, because of the close similarity with the K- motif. In addition, the commonly known motifs such as EHHEKKGIMDKIKEKLPGHH (K motif) and HSLLEKLHRSNSSSSSSSSDE (S- motif) were also observed. K motif is rich in lysine (K) residues and it is known for protective role of enzymatic activities from the drought effects [49]. The motif pattern formation indicates that cotton LEA proteins are actively involved in various biological processes and are group specific in terms of their activities. The distinct nature of the conserved motifs observed in all the LEA protein families, gives an indication that, the LEA proteins evolved from the gene expansion within their specific gene families. In addition, LEA 4 gene families were found to contain repeats of conserved motif 3, in which in some cases, the repeats were 5, the same attribute was also noted in which they were found to have tendencies of harboring repeat motifs, more so motif 8 [37]. We further did comparison of the common motifs with already identified motif, by the use of Tomtom motif comparison tool, adopting the distance measure of Sandelin-Wasserman function [50]. Motif 1 had 23 matches, with 5 jolma2013, 3 JASPERCORE2014 vertebrates and 15-uniprobe mouse. In motif 2, had 35 matches, 5 jolma2013, 5JASPERCORE2014 vertebrates and 25-uniprobe mouse. With MEME functional tool, we were able to affirm the similarities of our motifs to already published motif in the motif database.

**Table 3:**
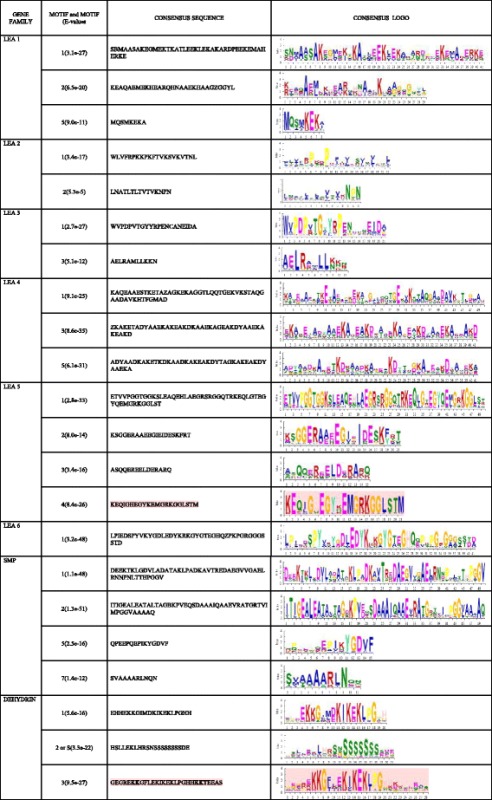
A consensus amino acid sequence of the different motifs features of each upland cotton LEA protein families

The colour scheme of the logo indicates amino acid types. Polar: green = uncharged; blue = +vely charged; red = -vely charged; Non-polar: violet/purple = aliphatic. As described by Dure, 2001

### Phylogenetic analyses of the LEA -proteins in cotton with other plants

**Fig. 2:**
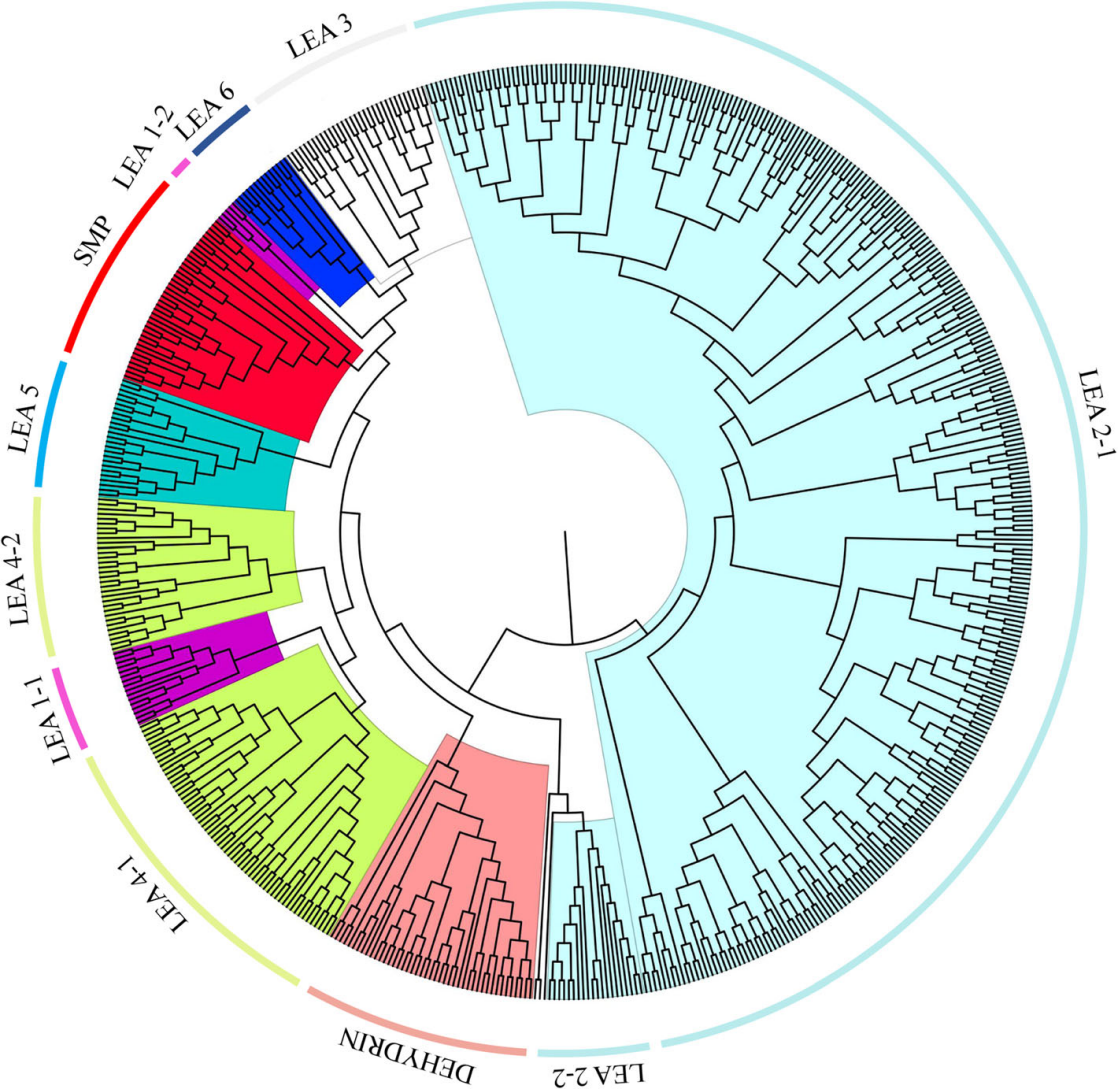
Phylogenetic relationship of *LEA* genes in three cotton species with *Arabidopsis* and *Pinus tabuliformis.* Neighbor-joining phylogeny of 242 genes for *G. hirsutum*, 136 genes for *G. arboreum*, 146 genes for *G. raimondii*, 30 genes for *Pinus tabuliformis* and 51 *Arabidopsis* LEA protein sequences, as constructed by MEGA 6.0. The difference colours mark the various *LEA* gene types

To get a better understanding of the evolutionary history and relationships of *LEA* gene families in cotton to other plants, multiple sequence alignment of 242 genes for *G. hirsutum*, 136 genes for *G. arboreum*, 146 genes for *G. raimondii*, 30 genes for *Pinus tabuliformis* and 51 genes for *Arabidopsis* LEA protein sequences (Fig. 2) were done. The boot strap values for some nodes of the NJ tree were low due to long sequence similarities. The reliability of the phylogenetic tree was done by reconstructing the phylogenetic tree with minimal evolution method. The trees produced by the two methods were identical thus the results were consistent. Based on the Phylogenetic tree analysis, *LEA* genes in cotton were further classified into eight (8) groups. LEA 2 was the largest group with 334 genes from *G. hirsutum* (157), *G. raimondii* (89), *G. arboreum* (85), *A. thaliana* (3) and *P. tabuliformis* (1). All the ortholog genes in LEA 2 were found in upland cotton, *G. hirsutum*, *G. arboreum* and *G. raimondii* genome while no ortholog genes were observed between upland cotton, *G. hirsutum* to either *Arabidopsis thaliana* and or *P. tabuliformis*. The second largest group were LEA 4, with highest number of genes 13 and 16 in *P. tabuliformis* and upland cotton respectively. Upland cotton, *G. hirsutum* contained the highest numbers of *LEA* genes of the following groups, LEA 1, LEA 2, LEA 3, LEA 5, LEA 6, SMP and dehydrin with the exception of LEA 4. Among all the *LEA* gene groups, only LEA 6 had fewer genes, 10 and 3 gene in cotton genome and *Arabidopsis* respectively (Table 1). The total number of ortholog genes between upland cotton, *G. hirsutum*, *G. arboreum* and *G. raimondii* were 201 out of 601 genes mapped on the Phylogenetic tree, which translates to 33%.

In this study, no ortholog genes were detected between upland cotton, *G. hirsutum*, *P. tabuliformis and Arabidopsis*. All the ortholog genes were detected only among the cotton species; this could be due to their evolution. Upland cotton, *G. hirsutum* emerged through hybridization mechanism between A and D genome [51]. Based on the results, there was close relationship between *Arabidopsis* and cotton species as compared to *P. tabuliformis*. The *LEA* genes seems to have a common evolutionary history [37], the aggregation pattern of the genes within the tree showed that LEA 1, LEA3, LEA 4, LEA5, LEA 6 and SMP had a common origin, similar results have also been obtained in the analysis of *LEA* genes in potato, in which SMP, LEA 1, LEA4 and LEA 5 shared a common point of origin [52]. A unique feature on the abundance of cotton *LEA* genes and their distribution was observed in which LEA 2 formed the majority of the cotton *LEA* gene families (Table 1 and Fig. 2). Analysis of the *LEA* genes in monocots and dicots, nearly half of the species containing *LEA* genes, the majority of the genes belong to the LEA 4 and dehydrin families [39]. The analysis of cotton *LEA* genes with other plants revealed that main differences occur in the *LEA 2* genes (Fig. 2). The abundance of LEA 2 genes was lowest in *Pinus tabuliformis* (1) and *Arabidopsis* (3) and higher in *G. hirsutum* (157), *G. arboreum* (85) and *G. raimondii* (89). Similar results were also been observed in which lower proportions of other *LEA* gene families were observed in grapevine but significantly higher number of LEA 2 genes were observed in rice and poplar [53]. It is important to note that, such large number of LEA 2 families have not been described in the previously investigated genomes of poplar [48], rice [11] and *Arabidopsis* [40]. This result may be explained in part by the improved annotation of the higher plant genomes available at Phytozome (v10.2) and also by the fact that LEA 2 is an unusual group composed of ‘a typical’ proteins of hydrophobic nature. This finding suggests that the LEA protein families in higher plants may be larger and much more complex than previously described. On the other hand, minor variations were observed in the other upland cotton *LEA* gene families. Based on this result, the entire *LEA 2* gene families probably were the last to evolve among the *LEA* gene families in higher plants.

### Chromosomal distribution of cotton genes encoding LEA proteins

**Fig. 3:**
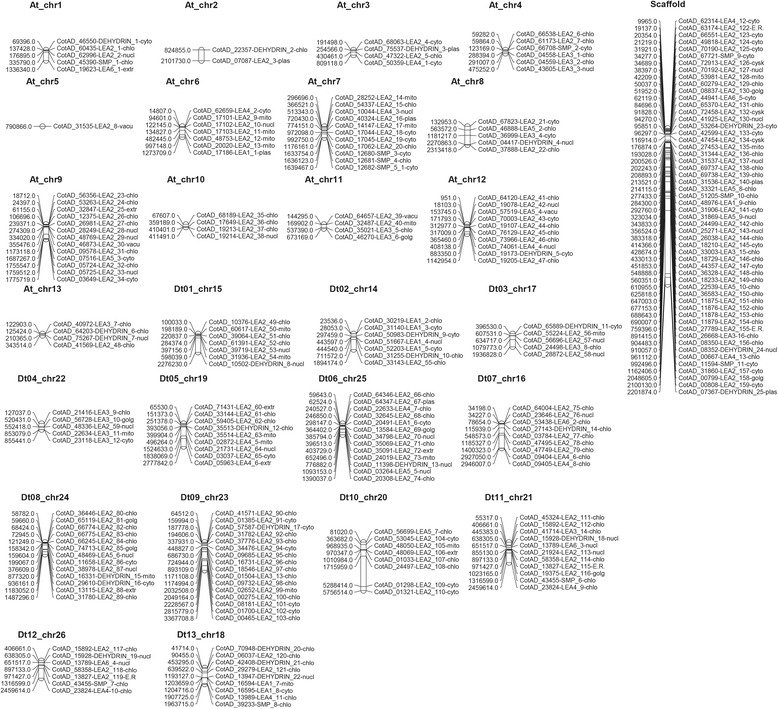
*LEA* genes distribution in tetraploid upland cotton, *Gossypium hirsutum* chromosomes. Chromosomal position of each *LEA* genes was mapped according to the upland cotton genome. The chromosome number is indicated at the top of each chromosome

To determine the chromosomal locations of cotton *LEA* genes based on their positions, data retrieved from the whole cotton genome sequences were used. Chromosome distribution was done by BLASTN search against *G. hirsutum* and *G. arboreum* in cotton genome project and *G.raimondii* genome database in Phytozome (http://www.phytozome.net/cotton.php). One hundred and eighty six (186) upland cotton *LEA* genes were mapped in all chromosomes by Map chart and 56 upland *LEA* genes into unknown chromosomes (scaffold). A plot of *LEA* genes on the cotton genome shows that LEA loci are found on every chromosome (Fig. 3). The distribution of the mapped *LEA* genes were more in Dt with 110 (59%) compared to At, with only 76 (41%) genes. However, the densities of these loci were high on Dt_chr 09, with 9% of all the *LEA* genes. Gene loss was observed on At_chr 05, with a single gene compared to its homolog chromosome Dt_chr 05, which had 9 genes. Similar case was also noted on chromosome At_chr02 and Dt_chr02 with 2 and 7genes respectively. This result indicates an element of gene loss during the hybridization period, as result of crossing over or other internal or external chromosomal phenomenon.

**Fig. 4:**
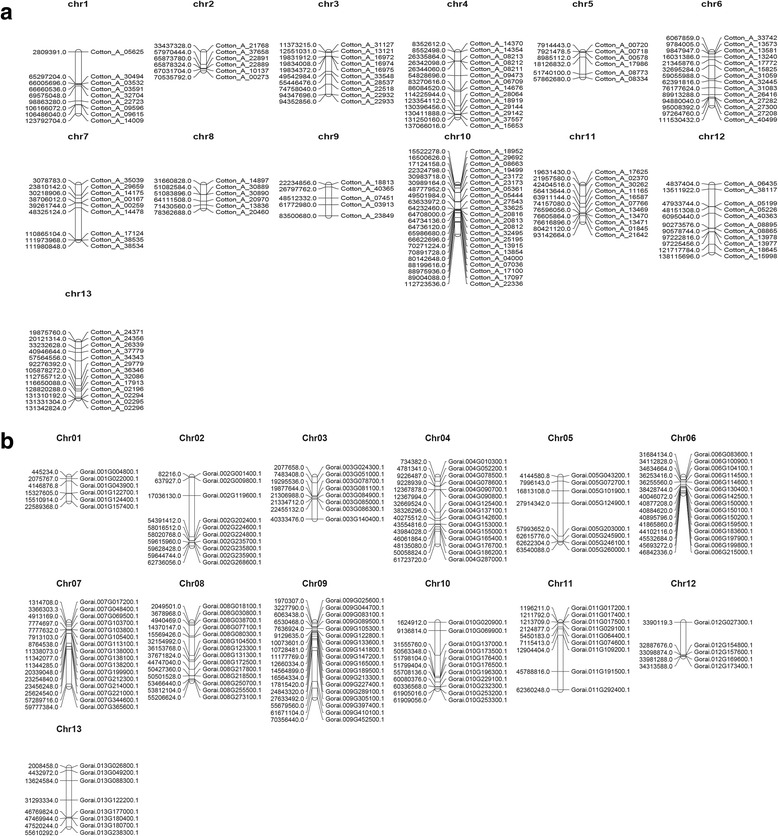
*LEA* genes distribution in A and D cotton chromosomes: Chromosomal position of each *LEA* genes was mapped according to the upland cotton genome. The chromosome number is indicated at the top of each chromosome. **a**: chromosomes of the diploid cotton of A genome, *G. arboreum*; **b**: chromosomes of the diploid cotton of D genome, *G. raimondii*

In the A genome of, *G. arboreum* 136 *LEA* genes were mapped across all the 13 chromosomes, high density of these loci were observed on chromosome 10, which contained 21 genes, translating to 15% of all the *LEA* genes in the genome. The mapping of the gene loci were generally uniform, the lowest loci density was observed on chromosome 9, with 5 genes (4%), followed by chr 2, chr5 and chr 8, with 6 genes each (Fig. 3). In D genome, (*G. raimondii*), 143 *LEA* genes were distributed across all the chromosomes. The highest gene loci density was in chromosome 9 with 18 genes (13%) and the lowest density was in chromosome 12, with only 5 genes (3%). The mapping of the *LEA* genes in both diploid and tetraploid cotton chromosomes, tend to have a unique clustering pattern, high density *LEA* gene clusters were observed in specific chromosomal regions, either at the upper arm, lower arm or the middle region of the chromosomes as shown on chromosomes At_ch01, Dt01_chr15, Dt02_chr14, Dt05_chr19 and Dt10_chr20 within the AD genome, chr02, chr05, chr06 and chr07 in A genome and in D genome, ch07 and chr11 (Fig. 4). The clustering pattern of the *LEA* gene and chromosomal location could be attributed to LEA gene duplication patterns [37].

In general, genes which belong to the same family are distributed across the entire chromosomes in order to ensure their maximum functionalization [54]; this was evident in LEA 2 genes, which was distributed across the entire chromosomes of both tetraploid and diploid cotton (Figs. 3 and 4). It was unique to find some members of *LEA* gene families with restricted distribution, mainly found in some chromosomes but not all like dehydrin despite of their numbers, this implies that dehydrin like genes have the tendency to duplicate and evolve more conservatively within a particular chromosome.

### Gene duplication and syntenic analysis

**Fig. 5:**
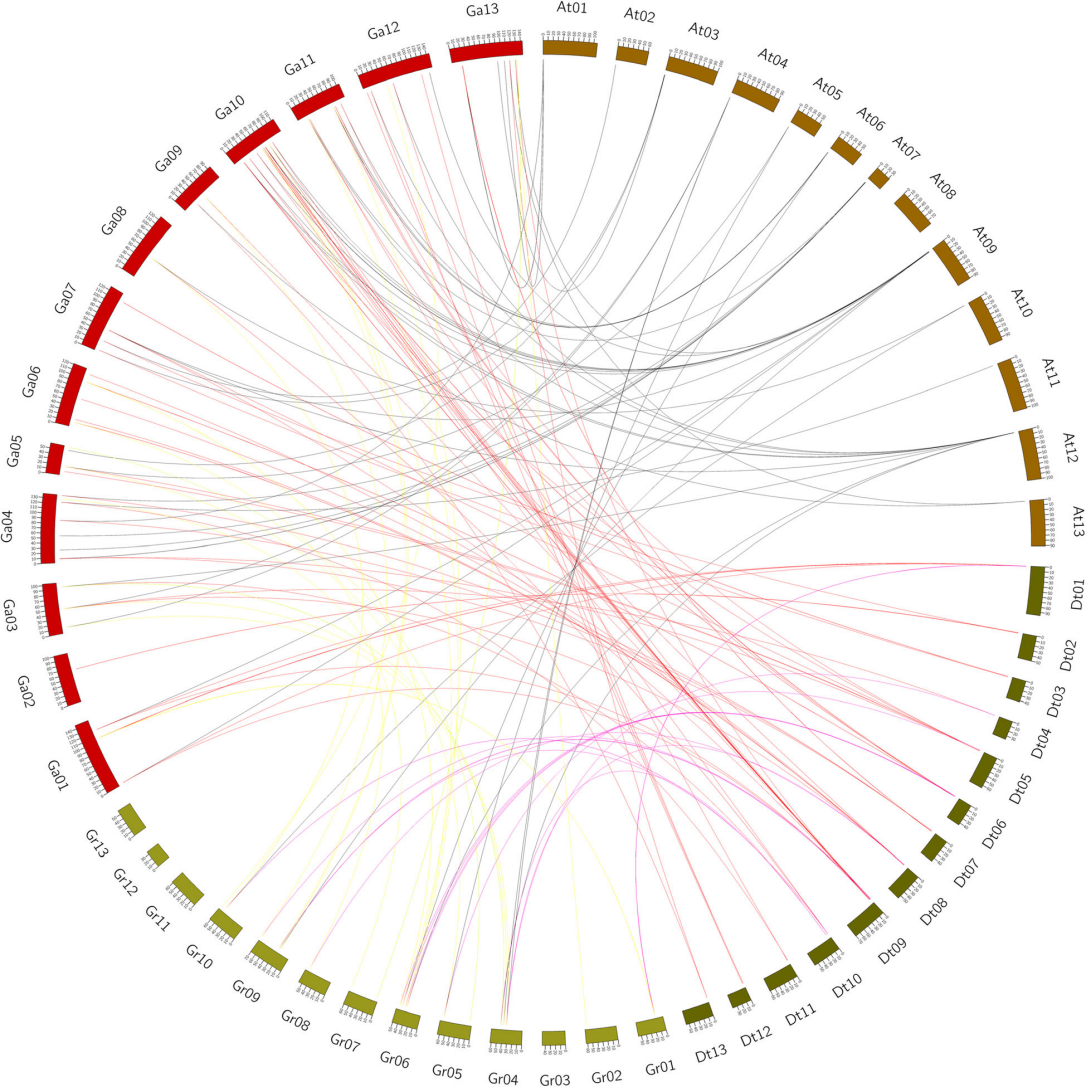
Syntenic relationships among *LEA* genes from *G. hirsutum*, *G. raimondii* and *G. arboretum. G. hirsutum, G. raimondii* and *G. arboretum* chromosomes are indicated in different colours. The putative orthologous *LEA* genes between *G. hirsutum* and *G. raimondii*, *G. hirsutum* and *G. arboretum*, and *G. raimondii* and *G. arboretum* by different colours

Expansion of gene families occurs through three processes namely, segmental duplication, tandem duplication and whole genome duplication [55]. Homologous and orthologous genes are the products of gene duplication events. Duplicated genes function in stress response and development processes in plants [56]. To analyse the relationships between the *LEA* genes and gene duplication events, syntenic blocks of *LEA* genes were combined among *G. hirsutum*, *G. raimondii* and *G. arboreum* (Fig. 5). A total of 241 *LEA* genes were duplicated across the three cotton genome. The most duplicated genes were detected between *G. hirsutum* and its ancestors, *G. arboreum* and *G. raimondii*, this could be due the origin of *G. hirsutum,* as a result of polyploidization of A and D genome (Table 4). Two types of duplication, tandem and segmental duplication event were identified. Majority of the duplicated *LEA* genes, were segmental, this implied that, segmental gene duplication, had a major contributing factor during the evolution time [57]. The Ka/Ks ratio is a pointer to selective pressure acting on a protein-coding gene. It has been observed that some systematic bias in some species do occur more easily in the process of nucleotides substitutions because of species diversity and high mutation rate do accelerates the changes in amino acid proportions [58]. The analysis of the Ka /Ks ratios of the 156 paralogous pairs, were less than 1 and only 20 had ratios of more than 1. Majority of LEA 2, LEA 4 and SMP had very low Ka/Ks ratios; the highest Ka/Ks ratio of 2.59265 was noted in LEA 6. This result is consistent to the previous findings of *Brassica napus LEA* genes, LEA 3 and LEA 6, families recorded higher Ka/Ks ratios, whereas LEA 5 and LEA 2 gene families recorded lowest Ka/Ks ratios [39]. In general Ka/Ks for paralogous gene pair of *LEA* genes had a range of 0 to 2.593 with mean of 0.4717. This result gives an indication that the *LEA* genes have been influenced extensively by purifying selection during the process of their evolution. LEA 2 gene families preferentially do have conserved structure and functions under selective pressure [59].

**Table 4:** Gene duplication, Ks, Ka and Ka/Ks values calculated for paralogous LEA gene pairs in cotton genome

GENE FAMILY	Paralogous gene pairs		Length (aa)	K_a_	K_s_	K_a_/K_s_	Negative/purifying selection	*P*-Value (Fisher)
	A	B						
DEHYDRIN	CotAD_66,538	CotAD_74,713	633	0.01677	0.04094	0.409579	YES	0.0891624
DEHYDRIN	CotAD_70,948	CotAD_75,267	996	0.33429	0.42203	0.792098	YES	0.122937
DEHYDRIN	CotAD_53,981	Cotton_A_05444	753	0.00533	0.01072	0.49715	YES	0.365412
DEHYDRIN	CotAD_65,119	Cotton_A_13573	618	0.01516	0.04762	0.318478	YES	0.0332022
DEHYDRIN	CotAD_66,245	Cotton_A_13581	1350	0.01615	0.03716	0.434571	YES	0.0218162
DEHYDRIN	CotAD_64,004	Cotton_A_14354	657	0.02039	0.07908	0.257869	YES	0.00188419
DEHYDRIN	CotAD_66,774	Cotton_A_23172	648	0.0101	0.05547	0.182079	YES	0.0030156
DEHYDRIN	CotAD_66,775	Cotton_A_23173	675	0.02652	0.06314	0.419977	YES	0.0380326
DEHYDRIN	CotAD_60,435	Cotton_A_24371	699	2.87675	2.00242	1.43664	NO	0.319287
DEHYDRIN	CotAD_58,358	Cotton_A_29692	633	0.01197	0.05683	0.210713	YES	0.00684175
DEHYDRIN	CotAD_66,538	Cotton_A_33548	633	0.00417	0.00659	0.63267	YES	0.549017
DEHYDRIN	CotAD_70,948	Cotton_A_40365	996	0.33435	0.4385	0.762473	YES	0.0864744
DEHYDRIN	CotAD_75,267	Cotton_A_40365	996	0.00385	0.0094	0.409167	YES	0.292983
DEHYDRIN	CotAD_65,119	Gorai.008G218500.1	618	0.00214	0.00666	0.321634	YES	0.36944
DEHYDRIN	Cotton_A_13573	Gorai.008G218500.1	618	0.01297	0.0548	0.236675	YES	0.00834018
LEA1	CotAD_01033	CotAD_08181	606	0.05146	0.15416	0.3338	YES	0.000633352
LEA1	CotAD_00275	CotAD_39,719	822	0.01476	0.03476	0.424567	YES	0.130131
LEA1	CotAD_01033	CotAD_46,550	606	0.0493	0.18716	0.263397	YES	1.42E-05
LEA1	CotAD_01298	CotAD_64,120	654	0.0404	0.13464	0.300053	YES	0.000332152
LEA1	CotAD_00275	Cotton_A_14009	822	0.01475	0.04503	0.327622	YES	0.0184625
LEA1	CotAD_01298	Cotton_A_22932	654	0.03303	0.12281	0.268983	YES	0.000202033
LEA1	CotAD_01033	Cotton_A_27543	606	0.05146	0.1456	0.353443	YES	0.00202823
LEA1	CotAD_01298	Gorai.004G155000.1	630	2.63603	3.65804	0.720612	YES	0.753791
LEA1	CotAD_01033	Gorai.009G305100.1	606	0.05153	0.14499	0.355394	YES	0.00205763
LEA2	CotAD_11,658	_Cotton_A_40499	789	0.02309	0.01751	1.3191	NO	0.985982
LEA2	CotAD_01700	CotAD_09578	780	0.01202	0.02634	0.456421	YES	0.151105
LEA2	CotAD_02652	CotAD_14,147	636	0.00835	0.01974	0.422805	YES	0.226532
LEA2	Cotton_A_13471	CotAD_17,103	180.94	2.32397	1.11323	0.778217	YES	837
LEA2	CotAD_13,584	CotAD_20,020	750	0.00878	0.01711	0.513478	YES	0.287642
LEA2	CotAD_17,062	CotAD_21,731	732	0.00719	0.04161	0.17276	YES	0.00506705
LEA2	CotAD_03649	CotAD_31,344	960	0.01103	0.02214	0.497982	YES	0.177084
LEA2	CotAD_17,101	CotAD_31,535	666	0.01576	0.04026	0.391498	YES	0.077316
LEA2	CotAD_17,102	CotAD_31,536	627	0.00422	0.03365	0.125467	YES	0.0107355
LEA2	Cotton_A_31083	CotAD_35,069	939	2.2748	1.83858	1.23726	NO	0.447623
LEA2	CotAD_19,214	CotAD_35,514	543	0.00955	0.02509	0.380645	YES	0.19023
LEA2	CotAD_19,623	CotAD_36,999	282	0.03296	0.04771	0.69081	YES	0.631725
LEA2	CotAD_18,546	CotAD_37,776	519	0.01016	0.04195	0.242116	YES	0.0375368
LEA2	CotAD_03649	CotAD_37,888	960	0.04522	0.54142	0.08352	YES	9.32E-36
LEA2	CotAD_40,972	CotAD_38,978	591	0.96025	2.04193	0.470264	YES	0.00125135
LEA2	CotAD_32,847	CotAD_39,064	612	0.01106	0.0461	0.239936	YES	0.0153075
LEA2	CotAD_12,375	CotAD_42,408	597	2.42062	1.68288	1.43838	NO	0.288342
LEA2	CotAD_28,872	CotAD_44,941	720	0.0141	0.01369	1.03042	NO	0.900519
LEA2	CotAD_08181	CotAD_46,550	606	0.00654	0.04975	0.131503	YES	0.00250188
LEA2	CotAD_25,271	CotAD_48,769	405	0.00647	0.01063	0.609395	YES	0.539117
LEA2	CotAD_28,252	CotAD_53,263	492	0.01356	0.04285	0.31656	YES	0.069282
LEA2	CotAD_09685	CotAD_53,981	753	0.00711	0.04386	0.162112	YES	0.00252472
LEA2	CotAD_35,091	CotAD_60,435	753	0.03016	0.07689	0.392211	YES	0.0144267
LEA2	CotAD_46,873	CotAD_60,617	630	0.00835	0.03452	0.241852	YES	0.0372109
LEA2	CotAD_46,888	CotAD_61,391	573	0.01387	0.05313	0.261111	YES	0.0175133
LEA2	CotAD_35,069	CotAD_62,996	954	0.00551	0.03643	0.151164	YES	0.0017334
LEA2	CotAD_17,045	CotAD_64,004	657	0.02247	0.06523	0.344452	YES	0.0157104
LEA2	CotAD_36,328	CotAD_64,346	630	0.01777	0.07564	0.23489	YES	0.000973496
LEA2	CotAD_21,924	CotAD_64,657	786	0.01373	0.04693	0.292471	YES	0.0120925
LEA2	CotAD_50,359	CotAD_66,538	633	0.01677	0.04094	0.409579	YES	0.0891624
LEA2	CotAD_19,078	CotAD_66,774	648	0.01009	0.04842	0.208435	YES	0.00834864
LEA2	CotAD_53,438	CotAD_68,189	618	0.02341	0.02898	0.80786	YES	0.519399
LEA2	CotAD_20,308	CotAD_70,003	573	0.00915	0.02291	0.399181	YES	0.206152
LEA2	CotAD_03649	CotAD_73,966	960	0.04597	0.527	0.087231	YES	4.70E-35
LEA2	CotAD_37,888	CotAD_73,966	960	0.01528	0.0442	0.345761	YES	0.0157353
LEA2	CotAD_23,118	CotAD_74,061	1215	0.01611	0.06882	0.234049	YES	5.00E-05
LEA2	CotAD_59,405	CotAD_76,129	627	0	0.00654	0	YES	0
LEA2	CotAD_13,584	Cotton_A_01845	750	0.00878	0.02294	0.382644	YES	0.139381
LEA2	CotAD_20,020	Cotton_A_01845	750	0	0.00568	0	YES	0
LEA2	CotAD_01700	Cotton_A_02196	780	0.09992	0.58986	0.169389	YES	8.68E-22
LEA2	CotAD_09578	Cotton_A_02196	780	0.0903	0.59944	0.150635	YES	7.07E-24
LEA2	Gorai.007G048400.1	Cotton_A_02294	576	2.54671	1.77281	1.43654	NO	0.3084
LEA2	CotAD_02652	Cotton_A_02370	636	0.01256	0.03311	0.379343	YES	0.101339
LEA2	CotAD_14,147	Cotton_A_02370	636	0.00416	0.01312	0.316903	YES	0.244174
LEA2	CotAD_09685	Cotton_A_05444	753	0.0089	0.04387	0.202818	YES	0.00516244
LEA2	CotAD_10,376	Cotton_A_05625	831	0.00645	0.03444	0.187227	YES	0.00723285
LEA2	CotAD_19,375	Cotton_A_06435	675	0.01345	0.05541	0.242679	YES	0.00759106
LEA2	CotAD_01700	Cotton_A_07036	780	0.01551	0.03701	0.419158	YES	0.0752732
LEA2	CotAD_09578	Cotton_A_07036	780	0.00342	0.01037	0.33004	YES	0.256013
LEA2	Cotton_A_02196	Cotton_A_07036	780	0.09428	0.60765	0.155148	YES	1.27E-23
LEA2	CotAD_12,681	Cotton_A_08212	432	0.03121	0.04928	0.633189	YES	0.35887
LEA2	Gorai.005G043200.1	Cotton_A_08334	792	0.00507	0.01526	0.332323	YES	0.16956
LEA2	CotAD_03649	Cotton_A_08663	960	0.00549	0.00437	1.25606	NO	0.744588
LEA2	CotAD_37,888	Cotton_A_08663	960	0.04378	0.55839	0.07841	YES	1.73E-37
LEA2	CotAD_10,044	Cotton_A_09473	1902	0.00274	0.00228	1.20458	NO	0.731531
LEA2	CotAD_46,888	Cotton_A_09596	573	0.00922	0.0453	0.203506	YES	0.0147038
LEA2	CotAD_46,873	Cotton_A_09615	630	0.00835	0.03452	0.241852	YES	0.0372109
LEA2	CotAD_32,487	Cotton_A_13240	630	0.00425	0.01917	0.221854	YES	0.103356
LEA2	CotAD_17,101	Cotton_A_13469	666	0.00195	0.01318	0.148053	YES	0.121749
LEA2	CotAD_31,535	Cotton_A_13469	666	0.01377	0.04718	0.291898	YES	0.0234164
LEA2	CotAD_31,536	Cotton_A_13470	627	0.00211	0.03373	0.062455	YES	0.00360292
LEA2	CotAD_17,103	Cotton_A_13471	837	2.58712	2.32397	1.11323	NO	0.778217
LEA2	CotAD_17,045	Cotton_A_14354	657	0.00201	0.01262	0.159578	YES	0.13409
LEA2	CotAD_17,062	Cotton_A_14370	732	0.0099	0.02648	0.374024	YES	0.0618224
LEA2	CotAD_21,731	Cotton_A_14370	732	0.00899	0.02354	0.381916	YES	0.138838
LEA2	CotAD_03649	Cotton_A_14478	960	0.04592	0.52972	0.086683	YES	3.29E-35
LEA2	CotAD_37,888	Cotton_A_14478	960	0.01247	0.03528	0.353401	YES	0.0321315
LEA2	CotAD_25,271	Cotton_A_14676	405	0.00647	0.03234	0.200226	YES	0.085476
LEA2	CotAD_31,140	Cotton_A_15998	747	0.00174	0.0058	0.300994	YES	0.356655
LEA2	CotAD_20,308	Cotton_A_17625	573	0.01375	0.02296	0.59881	YES	0.347235
LEA2	CotAD_44,941	Cotton_A_17986	720	0.01233	0.01369	0.900555	YES	0.874489
LEA2	CotAD_13,827	Cotton_A_18645	1104	2.12092	1.89653	1.11832	NO	0.642563
LEA2	CotAD_21,924	Cotton_A_18919	786	0.01028	0.05219	0.196967	YES	0.00026749
LEA2	CotAD_19,078	Cotton_A_23172	648	0	0.00672	0	YES	0
LEA2	CotAD_35,069	Cotton_A_24356	954	0.00551	0.03178	0.173291	YES	0.00508945
LEA2	CotAD_35,091	Cotton_A_24371	699	3.50309	1.61186	2.17333	NO	0.036477
LEA2	CotAD_22,539	Cotton_A_25195	408	1.23265	1.24112	0.993172	YES	1
LEA2	CotAD_23,646	Cotton_A_27282	609	0.02587	0.03738	0.692044	YES	0.542393
LEA2	CotAD_23,646	Cotton_A_27300	609	0.04249	0.13135	0.323481	YES	0.000630664
LEA2	Cotton_A_27282	Cotton_A_27300	609	0.04363	0.11818	0.369227	YES	0.00568388
LEA2	CotAD_08181	Cotton_A_27543	606	0	0.00697	0	YES	0
LEA2	CotAD_40,972	Cotton_A_29659	591	0.96659	2.0709	0.466747	YES	0.00123143
LEA2	CotAD_48,976	Cotton_A_29779	660	0	0.00642	0	YES	0
LEA2	CotAD_19,214	Cotton_A_30889	543	0.00237	0.0083	0.285978	YES	0.347253
LEA2	CotAD_35,514	Cotton_A_30889	543	0.00716	0.01659	0.431351	YES	0.312651
LEA2	CotAD_35,513	Cotton_A_30890	651	0.02193	0.05102	0.429783	YES	0.0738291
LEA2	CotAD_13,115	Cotton_A_31059	576	0.0207	0.0379	0.546252	YES	0.312514
LEA2	CotAD_30,219	Cotton_A_32495	597	0.01105	0.03626	0.304817	YES	0.0618481
LEA2	CotAD_50,359	Cotton_A_33548	633	0.01678	0.03388	0.495283	YES	0.175709
LEA2	CotAD_74,713	Cotton_A_33548	633	0.01678	0.03388	0.495283	YES	0.175709
LEA2	CotAD_23,118	Cotton_A_38117	1215	0.01611	0.06077	0.265138	YES	0.000321992
LEA2	CotAD_56,699	Cotton_A_38534	639	0.02021	0.04493	0.449883	YES	0.106618
LEA2	CotAD_56,696	Cotton_A_38535	630	0.01838	0.02269	0.809786	YES	0.670475
LEA2	CotAD_59,405	Cotton_A_40363	627	0.00636	0.04016	0.158424	YES	0.00848415
LEA2	CotAD_46,888	Gorai.001G122700.1	573	0.0046	0.0148	0.310385	YES	0.238274
LEA2	CotAD_46,873	Gorai.001G124400.1	630	0.00208	0.00674	0.30909	YES	0.361889
LEA2	CotAD_28,872	Gorai.005G203000.1	720	0.01233	0.02762	0.446407	YES	0.170613
LEA2	CotAD_44,941	Gorai.005G203000.1	720	0.00175	0.01368	0.127787	YES	0.0998325
LEA2	Cotton_A_17986	Gorai.005G203000.1	720	0.01055	0.02762	0.382183	YES	0.12817
LEA2	CotAD_30,219	Gorai.006G104100.1	597	0.00884	0.00707	1.25015	NO	0.743557
LEA2	Cotton_A_32495	Gorai.006G104100.1	597	0.01106	0.04362	0.25359	YES	0.0255988
LEA2	CotAD_17,101	Gorai.006G150200.1	666	0.01977	0.04018	0.491966	YES	0.209339
LEA2	CotAD_31,535	Gorai.006G150200.1	666	0.00391	0.01981	0.197433	YES	0.082505
LEA2	Cotton_A_13469	Gorai.006G150200.1	666	0.01777	0.04018	0.442182	YES	0.10598
LEA2	CotAD_23,646	Gorai.006G199800.1	609	0.04249	0.11411	0.372373	YES	0.00460089
LEA2	Cotton_A_27282	Gorai.006G199800.1	609	0.04364	0.10127	0.4309	YES	0.0292702
LEA2	Cotton_A_27300	Gorai.006G199800.1	609	0.00852	0.01474	0.578415	YES	0.130872
LEA2	Cotton_A_02294	Gorai.007G048400.1	576	2.54671	1.77281	1.43654	NO	0.3084
LEA2	Gorai.002G235700.1	Gorai.007G048400.1	576	2.49387	1.6786	1.48568	NO	0.261229
LEA2	Cotton_A_29142	Gorai.007G365600.1	630	0.02641	0.05237	0.504231	YES	0.0996897
LEA2	CotAD_08181	Gorai.009G305100.1	606	0.00435	0.02103	0.206723	YES	0.090366
LEA2	CotAD_19,214	Gorai.010G176400.1	543	0.00955	0.01664	0.574182	YES	0.403293
LEA2	CotAD_35,514	Gorai.010G176400.1	543	0	0.00822	0	YES	0
LEA2	Cotton_A_30889	Gorai.010G176400.1	543	0.00716	0.00825	0.8675	YES	0.63691
LEA3	CotAD_31,344	CotAD_37,888	960	0.0445	0.58201	0.076463	YES	1.76E-39
LEA3	CotAD_31,344	CotAD_73,966	960	0.04525	0.56675	0.079847	YES	1.38E-37
LEA3	CotAD_31,344	Cotton_A_08663	960	0.01103	0.02662	0.414477	YES	0.0917464
LEA3	CotAD_31,344	Cotton_A_14478	960	0.0452	0.56976	0.079332	YES	9.48E-38
LEA3	CotAD_68,063	Cotton_A_35039	654	0.0041	0.00611	0.670634	YES	0.565408
LEA3	CotAD_76,129	Cotton_A_40363	627	0.00636	0.03331	0.190971	YES	0.0241426
LEA4	CotAD_73,966	Cotton_A_08663	960	0.04453	0.54363	0.081917	YES	8.99E-37
LEA4	CotAD_73,966	Cotton_A_14478	960	0.00552	0.00865	0.63778	YES	0.447461
LEA4	Cotton_A_08663	Cotton_A_14478	960	0.04448	0.54647	0.081397	YES	6.22E-37
LEA4	CotAD_48,769	Cotton_A_14676	405	0	0.02141	0	YES	0
LEA4	CotAD_64,120	Cotton_A_22932	654	0.00607	0.01278	0.475195	YES	0.348209
LEA4	Gorai.004G155000.1	Cotton_A_22932	630	2.64206	3.59311	0.735312	YES	0.675748
LEA4	CotAD_46,550	Cotton_A_27543	606	0.00654	0.04242	0.154221	YES	0.00764013
LEA4	CotAD_64,120	Gorai.004G155000.1	630	2.60195	2.91277	0.893293	YES	0.834736
LEA4	Cotton_A_22932	Gorai.004G155000.1	630	2.64206	3.59311	0.735312	YES	0.675748
LEA4	CotAD_70,003	Gorai.006G083600.1	573	0.01378	0.02282	0.603673	YES	0.351221
LEA4	Cotton_A_17625	Gorai.006G083600.1	573	0.01841	0.02287	0.804972	YES	0.670413
LEA4	CotAD_46,550	Gorai.009G305100.1	606	0.00655	0.02791	0.234738	YES	0.0613694
LEA4	Cotton_A_27543	Gorai.009G305100.1	606	0.00435	0.01395	0.311662	YES	0.239375
LEA5	CotAD_39,719	Cotton_A_14009	822	0.00325	0.00976	0.333365	YES	0.258994
LEA5	CotAD_53,263	Cotton_A_22889	492	0.01631	0.04279	0.38131	YES	0.103316
LEA5	CotAD_74,061	Cotton_A_38117	1215	0.00426	0.01476	0.28873	YES	0.0817353
LEA6	CotAD_42,408	Cotton_A_13854	642	3.29524	1.27099	2.59265	NO	0.00291028
LEA6	CotAD_60,617	Gorai.001G124400.1	630	0.01046	0.04152	0.251878	YES	0.00495557
LEA6	Cotton_A_09615	Gorai.001G124400.1	630	0.01046	0.04152	0.251878	YES	0.00495557
SMP	Cotton_A_31083	CotAD_62,996	939	2.26658	1.80077	1.25867	NO	0.353457
SMP	CotAD_47,454	Cotton_A_07451	810	0.01141	0.05967	0.191218	YES	0.000658403
SMP	CotAD_61,391	Cotton_A_09596	573	0.0046	0.00736	0.624435	YES	0.545436
SMP	CotAD_64,657	Cotton_A_18919	786	0.00683	0.00508	1.345	NO	0.764969
SMP	CotAD_62,996	Cotton_A_24356	954	0	0.01351	0	YES	0
SMP	Cotton_A_31083	Cotton_A_24356	939	2.26658	1.8524	1.22359	NO	0.3981
SMP	CotAD_61,391	Gorai.001G122700.1	573	0.01855	0.05313	0.349233	YES	0.0424313
SMP	Cotton_A_09596	Gorai.001G122700.1	573	0.01387	0.0453	0.306205	YES	0.042074
SMP	Cotton_A_02294	Gorai.002G235700.1	627	0.00614	0.0377	0.162756	YES	0.0142575
SMP	CotAD_61,173	Gorai.004G137100.1	645	0.01861	0.04646	0.400532	YES	0.0204369
SMP	Cotton_A_31127	Gorai.004G137100.1	645	0.01965	0.0431	0.45597	YES	0.124339
SMP	CotAD_47,454	Gorai.009G452500.1	810	1.79916	1.14125	1.57648	NO	0.0145321
SMP	Cotton_A_07451	Gorai.009G452500.1	810	1.85045	1.14684	1.61353	NO	0.0110846

A B: paralogous gene pair; *aa* amino acids, *K*_*a*_ non-synonymous substitutions per non-synonymous site, *K*_*s*_ synonymous substitutions per synonymous site); *K*_*a*_*/K*_*s*_ the ratio, *SMP* seed maturation protein, *LEA* Late embryogenesis abundance, *Yes* presence of purifying selection while *NO* absence of purifying selection

### Prediction of *LEA* genes (mRNA) targeted by miRNAs in upland cotton

Large groups of small RNAs, known as microRNAs (miRNAs) are reported as the regulators in plant adaptation to abiotic stresses [60]. In transgenic creeping bent grass, *Agrostis stolonifera*, over expression of rice miR319a showed enhanced salt and drought tolerance [61]; over expression of miR396c and miR394 in plants was due to hypersensitive to salinity stress [62]. In cotton, *Gossypium hirsutum*, a group of miRNAs and their targets have been identified, and some of them respond to salt and drought stresses [60]. To get more information on *LEA* genes functions, we carried out the prediction of miRNAs targets on LEA transcripts (mRNA) by the use of psRNATarget, the same as been used for other functional genes in cotton [63]. Out of 242 upland cotton *LEA* genes, 89 genes were found to be targeted by 63 miRNAs, representing 37% of all the *LEA* genes (Additional file 4: Table S3). The highest levels of target were detected on the following genes with more than 6 miRNAs; CotAD_00799 (6 miRNAs), CotAD_06037 (9 miRNAs), CotAD_13,827 (6 miRNAs), CotAD_19,205 (6 miRNAs), CotAD_31,936 (6 miRNAs) CotAD_33,143 (6miRNAs), CotAD_41,925 (8miRNAs) and CotAD_69,738 (7miRNAs) as highlighted in (Additional file 4: Table S3). The rest of the genes were either targeted by one or not more than 5 miRNAs. The high number of miRNAs targeting *LEA* genes could possibly had direct or indirect correlation to their stress tolerance levels to abiotic stress more so drought. Some specific miRNAs had high level of target to various genes such as ghr-miR164 (5 genes), ghr-miR2949a-3p (7 genes), ghr-miR2950 (10 genes), ghr-miR7492a (10 genes), ghr-miR7492b (10 genes), ghr-miR7492c (10 genes), ghr-miR7495a (10 genes), ghr-miR7495b (10 genes), ghr-miR7504a (5 genes), ghr-miR7507 (5 genes), ghr-miR7510a (6 genes), ghr-miR7510b (10 genes), ghr-miR827b (4 genes) and lastly ghr-miR827c (4 genes). It has been found that miRNAs might be playing a role in response to drought and salinity stresses through targeting a series of stress-related genes [60]. Cotton ghr-miR7510b not only involved in drought stress but also highly up regulated in ovule and fibre, thus has an integral role in fibre formation [64]. Deep sequencing of miRNA under drought and salinity, ghr-miR408a, ghr-miR2911, ghr-miR156a/c/d and ghr-miR3954a/b were found to have differential expression in either of the stress factors, drought and salt stress [60].

### Gene ontology (GO) annotation

**Fig. 6:**
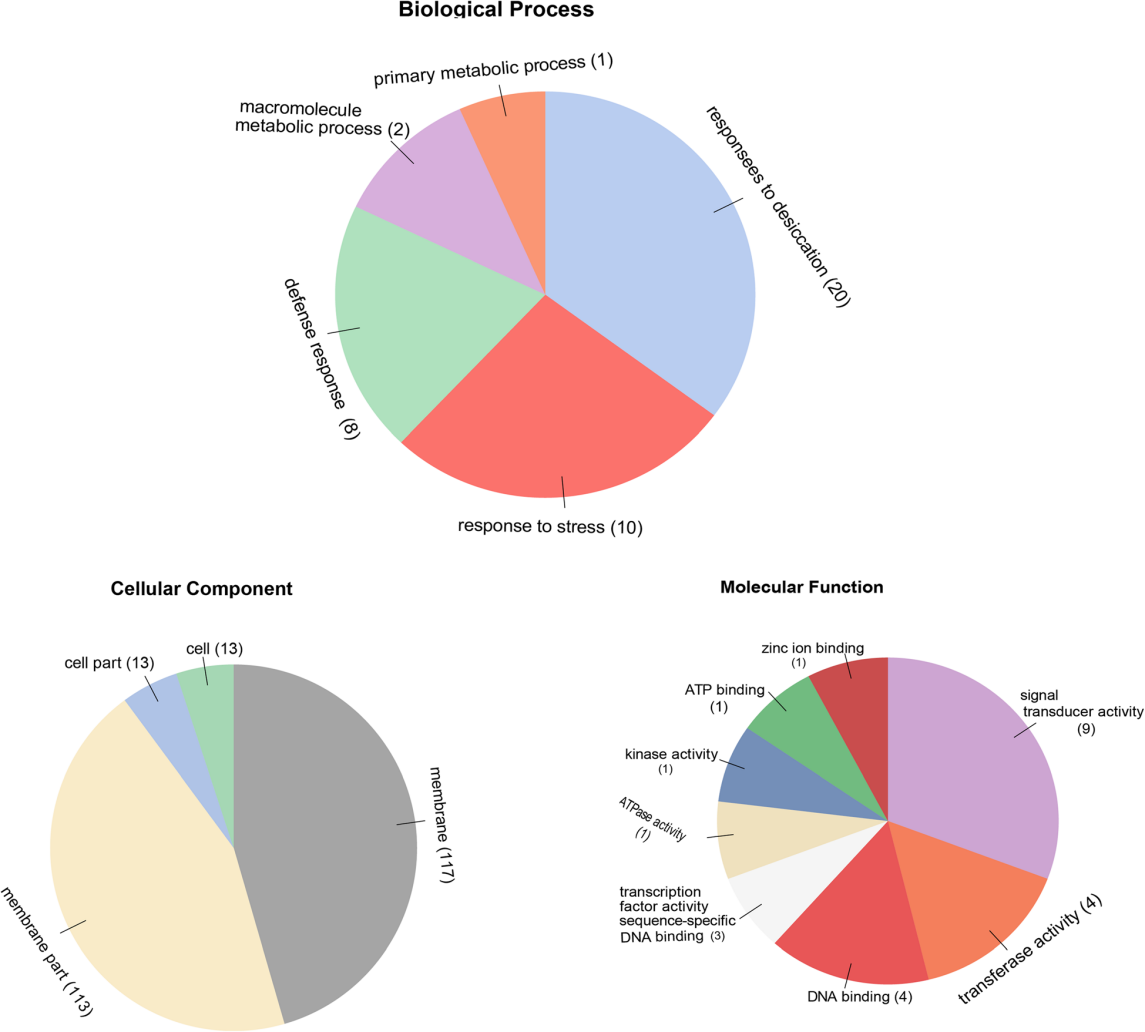
Gene Ontology (GO) annotation results for upland cotton *LEA* genes. GO analysis of 242 LEA protein sequences predicted for their involvement in biological processes (BP), molecular functions (MF) and cellular components (CC). For the results presented as detailed bar diagrams, as illustrated in Additional file 5: Table S4

The biological processes, molecular functions and cellular components of cotton *LEA* genes were examined according Gene Ontology (GO) data base. Blast2GO v4.0 was used to carry out the analysis (Fig. 6 and Additional file 5: Table S4). The results showed that the 242 *LEA* genes were putatively involved in a range of biological processes. Of the 5 terms of biological processes defined by Blast2Go terms, most *LEA* genes were predicted to function in the response to desiccation (~29%), followed by response to stress and response to defense. Molecular function prediction indicated that all 242 *LEA* genes, majority were involved in signal transducer activity, transferase activity and DNA binding. In cellular component prediction of *LEA* genes exhibited to be involved in membrane were 117, membrane parts (113), cell (13) and cell part (13). Higher numbers of upland cotton, *G. hirsutum LEA* genes were mainly involved in cellular component and molecular functions and few were found to be involved in biological processes. In all the LEA groups, molecular functions, biological process and cellular components were noted except in LEA 1 in which only two GO functions, biological process and cellular components were observed.

### Promoter cis-element analysis

**Fig. 7:**
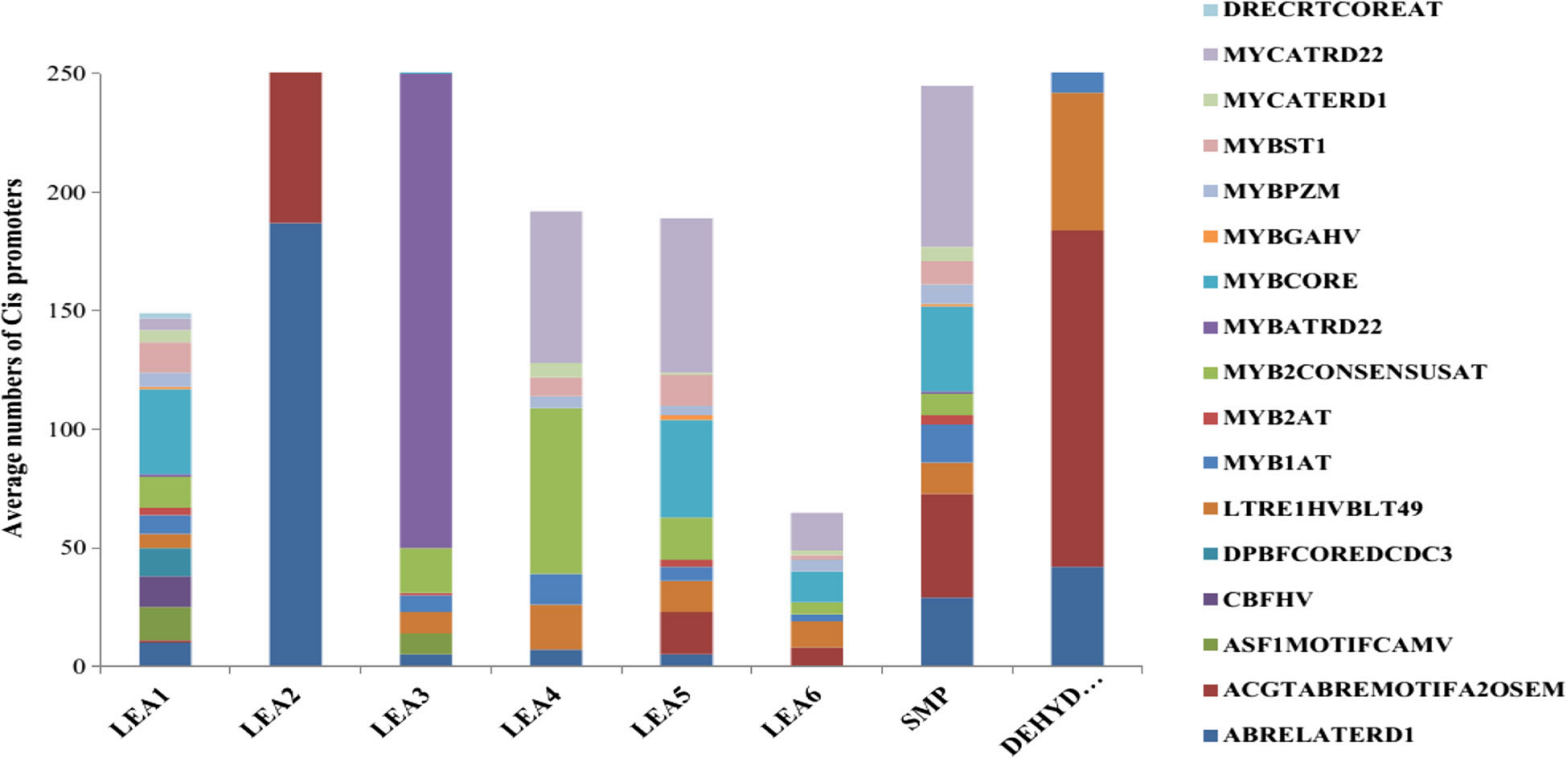
Average number of the cis-promoters ABRELATERD1 (ACGTG), DRECRTCOREAT (G/ACCGAC), MYBCORE (TAACTG), LTRE1HVBLT49 (CCGAC) and others in promoter region of *Gossypium hirsutum LEA* genes from each LEA families. The promoter regions were analyzed in the 1 kb upstream promoter region of translation start site using the PLACE database

Promoter sequences, 2 kb upstream and downstream of the translation start and stop site of all the *LEA* genes were obtained from the cotton genome project. Transcriptional response elements of *LEA* genes promoters were analyzed using the PLACE database (http://www.dna.affrc.go.jp/PLACE/signalscan.html) [36]. In order to determine cis –acting regulator element, we queried a section of the sequence of each gene, but only the start and end codon were used for the selection of cis–promoter elements. Analysis of the promoter region of all upland cotton *LEA* genes identified the presence of various stress responsive cis-acting regulatory elements, including DRE/CRT, ABRE, LTRE and MYBS. These stress-responsive elements were relatively abundant in the promoters of the upland cotton *LEA* genes, more specifically ABREs (Fig. 7 and Additional file 6: Table S5), indicating that LEA proteins may have an important functional role in drought stress response and tolerance in upland cotton, *G. hirsutum.* There were significant differences in the average proportions of the promoter elements detected within the different *LEA* gene families (Fig. 7).

The upland cotton *LEA* genes from LEA 2, LEA3, SMP and dehydrins gene families contained the highest average proportions of stress-responsive elements, while those from LEA 1 and LEA 6 contained the lowest proportions. ABRELATERD1 (ACGTG) was the dominant cis promoter elements, similar findings, with the predominance of ABRE cis-element, have been reported for *LEA* genes in tomato [46], *Arabidopsis* [40] and Chinese plum [47]. ABRE is a cis-acting element majorly involved in abscisic acid signaling in response to abiotic stresses, while DRE/CRT and LTRE are major cis-acting regulatory elements involved in the ABA-independent gene expression in response to water deficit (DRE/CRT) and cold (DRE/CRT and LTRE) [65]. MYBS is well-studied cis-acting promoter element with key role in the abscisic acid-dependent signaling pathway in response to drought, salt and cold [66].

### Upland cotton *LEA* genes expression analysis under drought stress

**Fig. 8:**
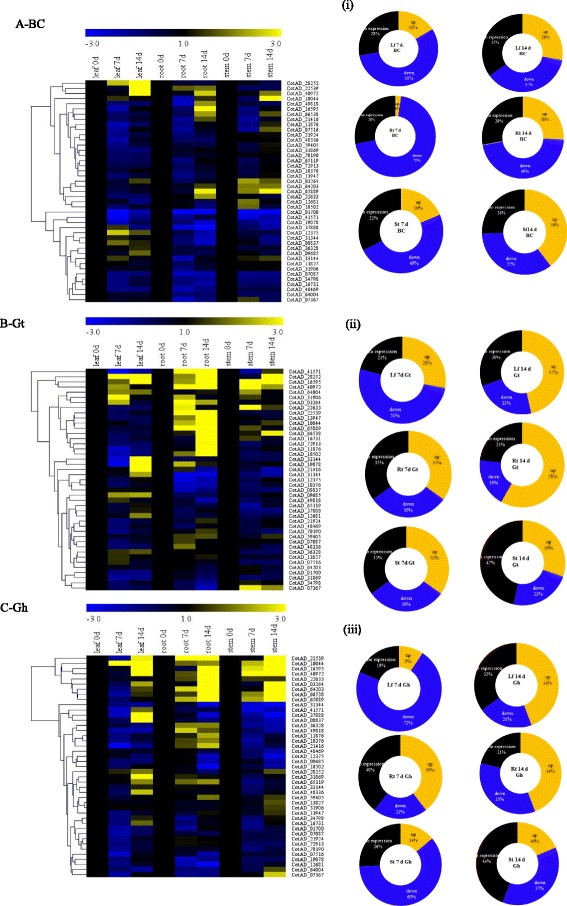
Differential expression of upland cotton *LEA* genes under drought stress. The heat map was visualized using Mev.exe program. (Showed by log_2_ values) in control, and in treated samples 7 and 14 days after drought treatment. **a** – BC_2_F_1_ (offspring), **b** – *Gossypium tomentosum* and **c** – *Gossypium hirsutum*. (i) Yellow – up regulated, blue – down regulated and black- no expression. (i). Percentage of genes exhibiting different responses to dehydration in leaf, root and stem of BC_2_F_1_; (ii). Percentage of genes exhibiting different responses to dehydration in leaf, root and stem of *Gossypium tomentosum* (iii). Percentage of genes exhibiting different responses to dehydration in leaf, root and stem of *Gossypium hirsutum*

To examine the expression profile of LEA proteins family in various tissues under drought stress treatments, we selected 42 *LEA* genes based on phylogenetic tree analysis, intron–exon and protein motif features, for each LEA group. Three cotton genotypes, *G. tomentosum*, a wild type known to be drought resistant, *G. hirsutum*, an elite cultivar, though drought susceptible cultivar and their backcross type BC_2_F_1_ generation were cultivated in the greenhouse under drought simulated and well watered condition. The qRT-PCR analysis was done on the three sets of accessions on different plant organs, roots, stems, and leaves. The results showed that *LEA* genes were differentially expressed under drought treatment across different tissues tested. Based on the cluster analysis, gene expression profiling were categorized into 2 groups, sub group 1, included 15 genes; the majority in this cluster of genes were up regulated in *G. tomentosum* in all tissues after 14 days of stress exposure and down regulation after 7 days of stress except in root, which showed partial expression. In *G. hirsutum*, majority of the genes were up regulated after treatment except in leave tissues in which the genes showed down regulation. In BC_2_F_1_, majority of the genes were down regulated after one week of exposure but expression was high after two weeks under drought stress. This result showed that these genes might be involved either directly or indirectly in drought stress, and their role was majorly concentrated in roots and stem. The second cluster, with 28 genes, the majority of the genes showed down regulation after one week of stress in all the tissues across the three genotypes. Some genes were up regulated across the three genotypes after 2 weeks of drought treatment. The results exhibited differential expression pattern in the 3 genotypes tested (Fig. 8). Some of the *LEA* genes were differentially expressed in the three plant organs and genotypes tested while others showed same expression pattern in different tissues, this could be due to functional divergence of *LEA* genes during plant development, for instance, CotAD_16,595 and CotAD_40,972 were highly expressed in the roots in all the three genotypes (Fig. 1), implying that they could be responsible for enhancing roots traits to drought tolerance. CotAD_13,827 and CotAD_31,906 were highly expressed in the leaves while others such as CotAD_10,044 and CotAD_03264 were highly up regulated in the stem. Further analysis of the expression showed that more than a half the upland cotton *LEA* genes were increased in roots and leaves at 7th and 14th day of stress as opposed to the stem. Roots and leaves tissues are highly sensitive to drought, the roots is the first organ to be affected by water deficit [67]. The leaves wilt or become chlorotic in stress conditions and affects photosynthesis process [68].

## Discussion

LEA proteins family is a large and widely diversified across plant kingdom [15]. The *LEA* gene family has been identified in several crops, such as rice and maize [69], in other organisms such as invertebrates and microorganisms [70]. However, characterization of the LEA protein family and their role in drought stress tolerance in upland cotton has never been reported. In this study, we identified different numbers of *LEA* genes in *G. hirsutum* (242), *G. arboreum* (136), *G. raimondii* (142), *A. thaliana* (51) and *P. tabuliformis* (30). The number of *LEA* genes in cotton genome AD (*G. hirsutum)* were higher than A (*G. arboreum)* and D (*G. raimondii*). The number of *LEA* genes in AD is approximately 1.78 and 1.70 times that in A and D respectively. The high number of *LEA* genes in *G. hirsutum* were more likely caused by gene duplication and the conservation of the *LEA* genes during the polyploidization process, signifying the important role played by these groups of gene families in the process of plant growth and development [40].

Gene duplication, is a major feature of genomic architecture, with cardinal role in the process of plant genomic and organismal evolution, resulting into new raw genetic materials for genetic drift, mutation and selection, which ultimately results into emergence of new gene functions and evolution of gene networks [71]. Gene duplication mechanism not only lead to the expansion of genome content but aids in the diversification of gene function to ensure adequate adaptability and evolution of plants [55]. The tetraploid cotton have undergone whole genome duplication events during their evolution period [72] and *G. hirsutum* emerged through allopolyploidy [73]. In this study, only 46 (17%) tandemly duplicated genes were detected, similarly only 6 genes were found to be tandemly duplicated in *Brassica napus*, perhaps because the evolution of upland cotton and *Brassica napus* are due to whole genome duplication [74, 75]. Majority of the upland *LEA* genes showed a close relationship with respect to the block locations of *G. arboreum* and *G. raimondii LEA* genes.

A phylogenetic analysis provided evidence of the contribution of whole genome duplication contribution to Upland cotton *LEA* genes abundance. *LEA* gene expansion through whole genome duplication have been observed in Arabidopsis [37] and *Brassica napus* [86]. The *Gossypium arboreum* genome contained 136 *LEA* genes and *G. raimondii* genome had 142 *LEA* genes; therefore, a WGD process would be expected to produce more than 242 *LEA* genes in *Gossypium hirsutum*. The *LEA* genes numbers proportions in *G. hirsutum* (tetraploid) implied that a larger number of the duplicated *LEA* genes were lost or became functionless after whole genome duplication. The loss of Upland cotton *LEA* genes could have been due to chromosomes rearrangement, the same mechanism was also observed in the case of *Brassica* [76]. The expansion of *LEA* genes in upland cotton was majorly through segmental duplication, 44% (130/242) of the upland cotton *LEA* genes emerged through segmental duplication. This finding is concurrent to observation made in *Brassica* 72 out of 108 genes occurred through segmental duplication [39] and in *Arabidopsis* in which 24% of its *LEA* genes arose through segmental type of gene duplication [40]. In synteny analysis, we identified 241 pairs with high similarity, implying that most *LEA* gene family members are embedded in highly-conserved syntenic regions, and some genes were either lost or recovered. The loss or gain of genes within the syntenic region have been observed in a number of gene families not only in *LEA* genes [77].

Characterization and structural analysis of genes with major functions on abiotic and biotic stress factors have been found to have fewer introns [48]. The analysis of the upland cotton *LEA* genes, LEA 1, 3, 4, 5, SMP and dehydrins genes had one to four intron with exception of LEA 2 and 6, which had zero to five introns. The reduced intron numbers in stress responsive genes have been recorded, such as trehalose-6-phosphate synthase gene family which plays an important role in abiotic stress and metabolic regulation [78]. The existence of introns in a genome is argued to cause enormous burden on the host [79]. The burden is because the introns requires a spliceosome, which is among the largest molecular complexes in the cell, comprising 5 small nuclear RNAs and more than 150 proteins [79]. It has also been found that intron transcription is costly in terms of time and energy [80]. Moreover, introns can extend the length of the nascent transcript, resulting into an additional expense for transcription [81].

The motif protein analysis and composition of each *LEA* gene family largely varied, although some amino acid-rich regions were detected, similar to previous studies done on *Arabidopsis* [40] and legumes [82]. We found that that genes belonging to the same families exhibited similar gene structure and motif composition. This results is consistent to previous studies which recorded similar exon - intron and protein motif within the same group of the *LEA* genes [23]. LEA proteins have disordered structure along their sequences due to their amino acid compositions [83]. LEA proteins play key roles in the plant cell despite of their disordered structure [41], they have the ability to form chaperons with other elements [84].The structural flexibility of the LEA proteins facilitate interactions with other macromolecules, such as membrane proteins, hence cell membrane stability during drought stress [85]. These results demonstrate that LEA proteins have intrinsic characteristics which enables them to functions as flexible integrators in protecting other molecules under drought stress and other forms of abiotic stress factors [86].

In relation to gene ontology (GO) analysis, biological processes, molecular functions and cellular components are features of genes or gene products that enable us to understand the diverse molecular functions of proteins. Cellular component and molecular activities were highest among the upland cotton LEA proteins, this could be in line with their functions of protecting the membranes and enzymes to maintain cellular activities under drought stress conditions [87]. The finding in this study is concurrent to previous studies which reported that LEA proteins are mainly located in subcellular regions such as chloroplast, nucleus, cytoplasm and mitochondria in *Arabidopsis* [40] and tomato [46]. The subcellular localization and the role of the LEA protein in the cell are positively correlated. Binding to different molecules such as ATP binding (GO: 0005524), sequence-specific DNA binding (GO: 0003700) and zinc ion binding (GO: 0008270) were the major activities for the action of upland LEA proteins as molecular function. Binding of LEA proteins to nucleic acids in order to protect cellular structures by constructing hydrogen network was reported, which is related to the roles of LEA proteins in drought stress tolerance [88]. In addition, LEA protein family groups have been found to enhance membrane stabilization through chaperons formation with phospholipids and other sugar molecules as described in model membranes under drought condition [87]. The molecular function of LEA proteins in drought stress may be through the binding activity.

In addition, biological processes in response to stress factors were dominant, response to desiccation (GO: 0009269); abscisic acid transport (GO: 0080168); response to stress (GO: 0006950); response to water (GO: 0009415); auxin-activated signalling pathway (GO: 0009734); response to water deprivation (GO: 0009414); response to cytokinin (GO: 0009735) and phosphorylation (GO: 0016310). These biological roles detected in cotton LEA proteins were consistent with earlier findings of biological functions of the LEA proteins such as oxidant scavenging activity, enzyme and nucleic acid preservation and membrane maintenance, these biological functions protect cell structures from the deleterious effects of drought and other abiotic stress factors [89]. Our findings is further supported by the highly up regulation of LEA proteins in various studies done in transgenic modal plant, *Arabidopsis* [90] and bacteria [91].

The small RNAs are a diverse class of non-coding regulatory with important function in gene regulation under drought stress conditions by destroying the target gene transcripts in plants [92]. The analysis of upland cotton miRNAs showed that 89 LEA transcripts were targeted by 63 different miRNAs. The NAC gene family are plant-specific transcriptional factors known to play diverse roles in various plant developmental processes, MYB is a transcriptional factor family mainly involved in controlling various processes like responses to biotic and abiotic stresses, development, differentiation, metabolism, defense among other biological processes while mitogen-activated protein kinase (MAPK) gene families also do play an important roles in plant growth, development and defense response. The three plant transcriptional factors, MYB, NAC and MAPK are ranked top under the context of drought and salinity indicating their important roles for the plant to combat drought and salinity stress. Through target prediction, a series of cotton miRNAs were found to be associated with MYB, NAC and MAPK genes including miR164 [60, 93]. In this research work miR164 was found to target four (5) *LEA* genes, CotAD_03784, CotAD_07516, CotAD_19,375, CotAD_24497and CotAD_63,174. The association of these 5 *LEA* genes with miR164, which have been found to be linked to highly ranked plants transcription factors under drought and salt stress, provides a strong evidence of a major role played by *LEA* genes in drought stress. A small RNA like miR827 have been found to confer drought tolerance in transgenic *Arabidopsis,* homologous form of the same miRNA, denoted as Hv-miR827*,* have been proved to confer drought tolerance in barley [94]. The same miRNA, ghr-miR827a/b/c/d, was found to target 12 different *LEA* genes, this implied that these genes targeted by the miRNA had a direct functional role in enhancing drought tolerance in upland cotton.

Gene promoters, also termed as cis-element, play various key roles in the transcriptional regulation of genes controlling a number of abiotic stress and plant hormones responses. Phyto-hormones enhance the ability of plants to adapt to changing environments. Many abiotic stress-related and plant hormones-related cis-elements, including W-Box, MBS, HSE, ABRE and TCA-elements, have been identified [95]. All of these and other cis-elements were detected in our investigation. Therefore, the results obtained is in agreement to the various cis-acting element detected in the analysis of *LEA* genes in various plants such as tomato [46], Chinese plum [47], in brassica [39] and poplar [48]. In each *LEA* gene, contained more than four cis-elements related to abiotic stress signal responsiveness, which provides strong evidence, that these genes might have important functions under different drought stresses.

**Fig. 9:**
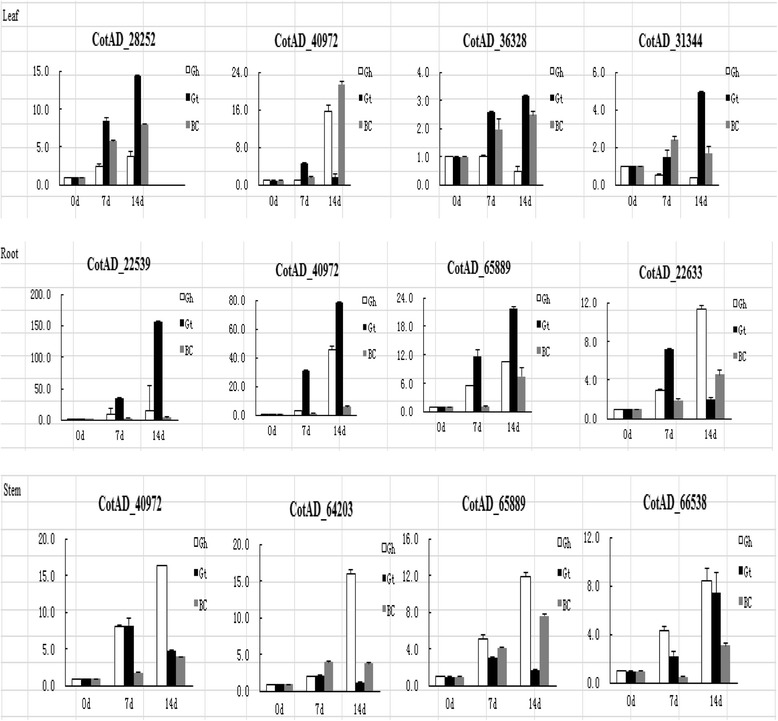
Quantitative PCR analysis of the selected *LEA* genes. Abbreviations: Rt (root), Sm (stem) and Lf (leaf). 0, 7 and 14 days of stress. Gh - *Gossypium hirsutum*, Gt - *Gossypium tomentosum* and BC-BC_2_F_1_ offspring. Y-axis: relative expression (2^−ΔΔCT^)

A number of studies have shown that *LEA* genes do have a significant contribution in drought stress [69]. From the heatmap and expression pattern of cotton *LEA* gene families, high number of *LEA* genes showed higher expression levels across all the plant organs tested. The high expression levels of these genes under drought condition, indicates the maintenance functions during stress conditions, leading to drought tolerance of the plants. A unique observation was made, in which high percentage of the *LEA 2* genes, used in the expression analysis, almost all showed high expression, and it would be of interest to characterize this group of *LEA* gene family in upland cotton. High expression pattern of the *LEA* genes have been observed in *Brassica napus* [39], *Prunus meme* [47], *Arabidopsis* [40] and sweet orange [53]. *LEA* genes have been found to have wide distribution and abundance among the terrestrial plants as opposed to aquatic plants, the abundance of these gene families could be pegged on to their conservative role, aquatic plants do not suffer from drought stress, thus the shrinking number of *LEA* genes. Therefore, the finding of this work and previous publication on function of *LEA* genes may explain why the *LEA* gene family have a wider distribution in terrestrial plants but not moss plants [96]. LEA families with close taxonomic relationships generally exhibited similar scales and distributions. However, the scales of the *LEA* gene family differ in upland cotton *Gossypium hirsutum* and other higher plants such as *Theobroma cacao* L, sweet orange, *Arabidopsis* among others; this could be due to changes in the environmental conditions. The high number of *LEA* genes in upland cotton, suggested that stress adaptation might have initiated the evolution of protein coding sequences which are LEA specific. Similar observation have been reported in maize in which adaptation to abiotic stress led to evolution of protein coding sequences, leading to the variation of *LEA* genes in maize compared to rice [97]. We carried out the expression profiling of the *LEA* genes in drought susceptible upland cotton, *Gossypium hirsutum*, drought tolerant cultivar *Gossypium tomentosum* and their BC_2_F_1_ offspring. High numbers of genes were found to be highly up regulated in *G. tomentosum* than in *G. hirsutum* (Figs. 8 and 9). The results obtained is concurrent to similar findings which have been reported in maize landraces with varying drought stress tolerance levels when compared at the transcriptional level [98]. This result implied that more tolerant cotton genotypes had a greater ability to rapidly adjust more genes under drought stress than the more susceptible cotton cultivars. Moreover, rapid adjustments of greater number of differentially expressed genes and of different transcriptome factor families is considered an important trait of the drought tolerant genotypes [99].

## Conclusions

This research work provides the very first detailed analysis, characterization and expression profile of upland LEA genes under drought condition. A total of 242 *LEA* genes were identified in upland cotton and divided into eight groups. Chromosomal mapping and syntenic analysis showed that all the *LEA* genes were distributed in all the cotton chromosomes with some genes clustering either on the upper arm or the middle region of the chromosomes. Segmental gene duplication was found to have played a major role in the expansion of upland cotton *LEA* genes coupled with whole genome duplication. High numbers of cotton *LEA* genes had few introns. Genes belonging to the same family exhibited similar gene structures and protein motif composition. Expression profiling of the selected *LEA* genes showed differential expression under drought treatment across different plant organs. The outcome of this research provides the most current information thus will increases our understanding of *LEA* genes in cotton and the general role in drought stress tolerance. This work lays the very first foundation for further investigations of the very specific functions of these LEA proteins in cotton in reference to drought stress and other abiotic stress factors.

## Additional files

Additional file 1: Table S1. List of primers used for upland cotton, *Gossypium hirsutum LEA* genes expression analysis under drought stress. (DOCX 19 kb)

Additional file 2: Table S2. LEA gene in upland cotton, *Gossypium hirsutum* and their subcellular location prediction. The colour scheme indicates where the genes are sub-localized. (DOCX 90 kb)

Additional file 3: Figure S1. Phylogenetic tree, gene structure and motif compositions of *LEA 2* genes in upland cotton. The phylogenetic tree was constructed using MEGA 6.0. Exon/intron structures of *LEA* genes in upland cotton, exons introns and up / down-stream were represented by yellow boxes, black lines and blue boxes, respectively. Protein motif analysis represented by different colours, and each motif represented by number. (PDF 3609 kb)

Additional file 4: Table S3. *LEA* genes and mRNA targets. (DOCX 53 kb)

Additional file 5: Table S4. Gene ontology (GO) terms annotation of *LEA* genes in upland cotton. (DOCX 66 kb)

Additional file 6: Table S5. Cis element analysis of putative LEA promoters related to drought stress. (DOCX 719 kb)

Additional file 7: Data. Newick format for the phylogenetic tree. (NWK 19 kb)

## Abbreviations

GO: Gene ontology
GolS: Galactinol synthase
LEA: Late embryogenesis abundance
miRNA: Micro ribonucleic acid
SMP: Seed maturation proteins
