# Supplementary material for: Characterization of the late embryogenesis abundant (LEA) proteins family and their role in drought stress tolerance in upland cotton
Source: BMC Genet. 2018 Jan 15;19:6. doi: 10.1186/s12863-017-0596-1 (PMC5769447; doi:10.1186/s12863-017-0596-1)
Supplement: Supplementary file 1 — List of primers used for upland cotton, Gossypium hirsutum LEA genes expression analysis under drought stress. (DOCX 19 kb) [file 12863_2017_596_MOESM1_ESM.docx]

Supplementary Table 1: List of primers for *LEA* gene expression analysis

| S/NO | PRIMER NAME | Forward | Reverse |
| --- | --- | --- | --- |
| 1 | gRICH_28252 | CAAATTGCCAAGGAAAGGAA | TGATGAGCTCCAGTGGATTG |
| 2 | gRICH_16595 | AACATCGGCAAAGCTCAACT | CCATGGAGATATTGGGGTTG |
| 3 | gRICH_11876 | ACAAATCACCCTCCGAACAG | TCCCAACTTTCCATCTCACC |
| 4 | gRICH_59405 | CTCCACTCCCAATTTCCTCA | CGTAGATGAAAGGGGACCAA |
| 5 | gRICH_41571 | CACTCTCCTCGACACCACTG | TATGGCCTTGGTACGTTGGT |
| 6 | gRICH_12375 | AGCTCCGAACATGCTCACTT | GTTGCCCGTAAACCTTGATG |
| 7 | gRICH_36328 | ACCCTACCAAACCCCATTTC | GGGGATCAATGTCGGTAAAG |
| 8 | gRICH_66538 | CCAATTCACACTGCTCTCCA | GCAGCCCTGTTCCTTGTAAA |
| 9 | gRICH_07087 | AGAGGGTTTTCTGGGTGGAT | AGCAAAGATCCCATCACCAC |
| 10 | gRICH_33144 | AATCCGATCACGGTTCCATA | CCAAAAACGGGTCTCAACAT |
| 11 | gRICH_09685 | CGTGCACGGAACTAAAAACA | CAACCCTTATCCCGACCTTT |
| 12 | gRICH_21924 | TCGATATAGGGGAGGGTTCC | GCTCCCATCTTTGCTTTCAC |
| 13 | gRICH_10376 | TACGACCCATTCACCCTCTC | ACGGGATCCCATTTTTCTTC |
| 14 | gRICH_01700 | ACGTCATCCAAATTCCCAAG | CGGGCTTGAAAACGAAGTAA |
| 15 | gRICH_34798 | AGTGCTCCCTTGGTTCCTCT | ATCCCCAGAATGACGATCAG |
| 16 | gRICH_16731 | AGTGCTCCCTTGGTTCCTCT | ATCCCCAGAATGACGATCAG |
| 17 | gRICH_13827 | CCCAATTACGATCACGACCT | GGACACTTGGGAGGAAAACA |
| 18 | gRICH_31869 | TGGGTCCAGAACAAGCAGAT | TGGCCTTGGAAGTACAGCTT |
| 19 | gRICH_65119 | TTAGAGCTCGGAATGGGAAC | CCTCCACTTGGTCTGCTTGT |
| 20 | gRICH_37888 | GAAGGCCTCCTTGAAGATGA | GAAATCTGAACCCGCTTGAA |
| 21 | gRICH_31344 | AGATGGAGAGCGTGAGAAGG | TGTTGACACCCCCGTAAAAT |
| 22 | gRICH_48469 | TGATAAAGGGTGGCCTGAGT | CACTGCAAAGCTCTTGACCA |
| 23 | gRICH_49818 | ATCGATTTCCGTTTTGGTTG | GATACCGGCTTGAAGCAAGA |
| 24 | gRICH_64004 | TCGCTGCTTTGTTCATCATC | ATTTTTGACGGAGACGTTGG |
| 25 | gRICH_19078 | GTTCCGATCGGTTCAGGTTA | TTGAGCTCTCCCGGTAGGTA |
| 26 | gRICH_08837 | CGCTCTTCCTCTTCTTCTGC | CCATCCAAGGAGGAAGGATT |
| 27 | gRICH_72913 | AAGAACCCAAACCCTGTTCC | GATGCTGCCAGGCTTAATGT |
| 28 | gRICH_70190 | CAGGCTTGATTCCAGATGCT | CAAGTGCATTGAGTCCCAAG |
| 29 | gRICH_22633 | ATGGCTTGCTCTCTCTCCAA | TCTTTAGCTCCGCCAGACAT |
| 30 | gRICH_21416 | ACGCTATCTCCAGACGAGGA | TCTCGTTCCCACAGTTCTCC |
| 31 | gRICH_31906 | GGACAAGTACTGGGCTCCTG | AGTGATGGTGTGGCTTCTCC |
| 32 | gRICH_22539 | CAATGGAGACAGCAGAAGCA | AGGTATGTTTCACCGCATCA |
| 33 | gRICH_10044 | GAGTGCGGCACACTATACGA | GCTGCCTCTTCCTTTTTCCT |
| 34 | gRICH_03264 | AGGCAGGGAGAGACGGTTAT | CATTTCCTGGTACCCCTCAC |
| 35 | gRICH_07516 | GGAGAGCCAGTTGGAAATGA | CAATGCTTGGACCATTTGTG |
| 36 | gRICH_48336 | CATAACCGCATCAGGTCTCC | AACAGTCGGATTCCCCTTG |
| 37 | gRICH_12681 | TGTAACAGCAGCATGGTTGG | TCAGCAACATGTGGAAGCTC |
| 38 | gRICH_40972 | ACAAACTCCCAGGACAGCAT | TTAATGGGGAGCAGTGGTCT |
| 39 | gRICH_64203 | GGGAATCATGCACAAGATCG | CGCCTTCGTGATGAACTTCT |
| 40 | gRICH_13947 | GAATGAGGCAGCAGGAACTT | ACCCTAGGATGTGACCGTTG |
| 41 | gRICH_65889 | TGGTGTTGGCTTCCATCATA | TGCAACACCCCCATACAATA |
| 42 | gRICH_10502 | TGTTTTGGACAAACCAACGA | AAGCCCTACCACCACAGCTA |
| 43 | gRICH_07367 | GCCATATACCGCTGTTCGAT | CCGTTCTTTCAACAGCAACA |
