# Supplementary material for: Characterization of the late embryogenesis abundant (LEA) proteins family and their role in drought stress tolerance in upland cotton
Source: BMC Genet. 2018 Jan 15;19:6. doi: 10.1186/s12863-017-0596-1 (PMC5769447; doi:10.1186/s12863-017-0596-1)
Supplement: Supplementary file 2 — LEA gene in upland cotton, Gossypium hirsutum and their subcellular location prediction. The colour scheme indicates where the genes are sub-localized. (DOCX 90 kb) [file 12863_2017_596_MOESM2_ESM.docx]

Supplementary table 2: *LEA* gene in upland cotton, *Gossypium hirsutum* and their sub cellular location prediction.

| **LEA TYPE** | **Gene id** | **Pprowler** | | | | **TargetP** | | | | | |
| --- | --- | --- | --- | --- | --- | --- | --- | --- | --- | --- | --- |
|  |  | **SP** | **mTP** | **cTP** | **Other** | **Length** | **cTP** | **mTP** | **SP** | **other** | **Location** |
| LEA1 | CotAD_28249 | 0.03 | 0.13 | 0.02 | 0.82 | 113 | 0.333 | 0.07 | 0.162 | 0.67 | _ |
| LEA1 | CotAD_53264 | 0.03 | 0.13 | 0.02 | 0.83 | 420 | 0.283 | 0.066 | 0.13 | 0.774 | _ |
| LEA1 | CotAD_28252 | 0.02 | 0.11 | 0.01 | 0.87 | 164 | 0.175 | 0.085 | 0.146 | 0.831 | _ |
| LEA1 | CotAD_53263 | 0.02 | 0.11 | 0.01 | 0.87 | 164 | 0.181 | 0.084 | 0.14 | 0.826 | _ |
| LEA1 | CotAD_17186 | 0.02 | 0.1 | 0.01 | 0.87 | 165 | 0.384 | 0.059 | 0.149 | 0.694 | _ |
| LEA1 | CotAD_20491 | 0.02 | 0.1 | 0.01 | 0.87 | 165 | 0.384 | 0.059 | 0.149 | 0.694 | _ |
| LEA1 | CotAD_36446 | 0.01 | 0.07 | 0 | 0.92 | 116 | 0.152 | 0.102 | 0.168 | 0.855 | _ |
| LEA1 | CotAD_16594 | 0.01 | 0.07 | 0 | 0.92 | 115 | 0.157 | 0.089 | 0.184 | 0.848 | _ |
| LEA1 | CotAD_16595 | 0.01 | 0.07 | 0 | 0.92 | 115 | 0.157 | 0.089 | 0.184 | 0.848 | _ |
| LEA2 | CotAD_11876 | 0.29 | 0.14 | 0 | 0.57 | 209 | 0.009 | 0.741 | 0.07 | 0.245 | M |
| LEA2 | CotAD_24498 | 0.27 | 0.14 | 0 | 0.59 | 209 | 0.011 | 0.729 | 0.052 | 0.263 | M |
| LEA2 | CotAD_17102 | 0.19 | 0.22 | 0.01 | 0.58 | 209 | 0.025 | 0.814 | 0.022 | 0.277 | M |
| LEA2 | CotAD_31536 | 0.21 | 0.2 | 0.01 | 0.57 | 209 | 0.029 | 0.723 | 0.03 | 0.329 | M |
| LEA2 | CotAD_76129 | 0.12 | 0.22 | 0.01 | 0.65 | 209 | 0.012 | 0.87 | 0.031 | 0.508 | M |
| LEA2 | CotAD_41569 | 0.04 | 0.11 | 0.01 | 0.84 | 101 | 0.156 | 0.167 | 0.06 | 0.587 | _ |
| LEA2 | CotAD_33321 | 0.52 | 0.11 | 0.01 | 0.35 | 210 | 0.059 | 0.43 | 0.069 | 0.355 | M |
| LEA2 | CotAD_41571 | 0.33 | 0.12 | 0.01 | 0.54 | 210 | 0.027 | 0.458 | 0.111 | 0.508 | _ |
| LEA2 | CotAD_46873 | 0.33 | 0.09 | 0 | 0.58 | 210 | 0.009 | 0.223 | 0.317 | 0.7 | _ |
| LEA2 | CotAD_60617 | 0.26 | 0.11 | 0 | 0.63 | 210 | 0.008 | 0.227 | 0.384 | 0.678 | _ |
| LEA2 | CotAD_12375 | 0.07 | 0.11 | 0 | 0.82 | 190 | 0.016 | 0.076 | 0.066 | 0.991 | _ |
| LEA2 | CotAD_42408 | 0.12 | 0.09 | 0 | 0.79 | 210 | 0.028 | 0.298 | 0.206 | 0.899 | _ |
| LEA2 | CotAD_36328 | 0.44 | 0.07 | 0 | 0.5 | 210 | 0.003 | 0.269 | 0.642 | 0.773 | _ |
| LEA2 | CotAD_64346 | 0.47 | 0.06 | 0 | 0.47 | 210 | 0.003 | 0.306 | 0.632 | 0.75 | _ |
| LEA2 | CotAD_02652 | 0.28 | 0.5 | 0.07 | 0.15 | 212 | 0.06 | 0.33 | 0.01 | 0.724 | _ |
| LEA2 | CotAD_14147 | 0.33 | 0.46 | 0.03 | 0.18 | 212 | 0.019 | 0.506 | 0.012 | 0.797 | _ |
| LEA2 | CotAD_66538 | 0.43 | 0.11 | 0.01 | 0.45 | 211 | 0.026 | 0.189 | 0.041 | 0.965 | _ |
| LEA2 | CotAD_50359 | 0.44 | 0.13 | 0.01 | 0.43 | 211 | 0.048 | 0.172 | 0.039 | 0.95 | _ |
| LEA2 | CotAD_74713 | 0.44 | 0.13 | 0.01 | 0.43 | 211 | 0.048 | 0.172 | 0.039 | 0.95 | _ |
| LEA2 | CotAD_32847 | 0.08 | 0.14 | 0.01 | 0.77 | 204 | 0.104 | 0.418 | 0.014 | 0.592 | _ |
| LEA2 | CotAD_39064 | 0.07 | 0.15 | 0.01 | 0.76 | 204 | 0.13 | 0.408 | 0.011 | 0.572 | _ |
| LEA2 | CotAD_46270 | 0.6 | 0.24 | 0.09 | 0.07 | 231 | 0.77 | 0.045 | 0.015 | 0.649 | C |
| LEA2 | CotAD_67823 | 0.31 | 0.16 | 0.03 | 0.51 | 222 | 0.466 | 0.01 | 0.029 | 0.913 | _ |
| LEA2 | CotAD_18546 | 0.75 | 0.13 | 0.06 | 0.06 | 173 | 0.01 | 0.701 | 0.098 | 0.177 | M |
| LEA2 | CotAD_37776 | 0.76 | 0.13 | 0.06 | 0.05 | 173 | 0.009 | 0.68 | 0.102 | 0.219 | M |
| LEA2 | CotAD_30219 | 0.92 | 0.02 | 0 | 0.06 | 199 | 0.013 | 0.372 | 0.318 | 0.212 | M |
| LEA2 | CotAD_17101 | 0.63 | 0.24 | 0.1 | 0.03 | 222 | 0.125 | 0.077 | 0.082 | 0.619 | _ |
| LEA2 | CotAD_31535 | 0.63 | 0.24 | 0.1 | 0.03 | 222 | 0.147 | 0.071 | 0.087 | 0.606 | _ |
| LEA2 | CotAD_01033 | 0.74 | 0.1 | 0 | 0.17 | 202 | 0.007 | 0.616 | 0.121 | 0.658 | _ |
| LEA2 | CotAD_01298 | 0.61 | 0.25 | 0.09 | 0.04 | 218 | 0.029 | 0.07 | 0.079 | 0.959 | _ |
| LEA2 | CotAD_01321 | 0.56 | 0.2 | 0.09 | 0.15 | 238 | 0.545 | 0.015 | 0.015 | 0.631 | _ |
| LEA2 | CotAD_00275 | 0.64 | 0.23 | 0.1 | 0.02 | 274 | 0.404 | 0.053 | 0.025 | 0.511 | _ |
| LEA2 | CotAD_00465 | 0.01 | 0.11 | 0.64 | 0.23 | 304 | 0.626 | 0.063 | 0.025 | 0.523 | C |
| LEA2 | CotAD_07087 | 0.96 | 0 | 0 | 0.04 | 206 | 0.019 | 0.118 | 0.804 | 0.162 | S |
| LEA2 | CotAD_17649 | 0.18 | 0.47 | 0.03 | 0.32 | 235 | 0.033 | 0.308 | 0.013 | 0.647 | _ |
| LEA2 | CotAD_19213 | 0.01 | 0.06 | 0 | 0.92 | 100 | 0.183 | 0.111 | 0.048 | 0.807 | _ |
| LEA2 | CotAD_19214 | 1 | 0 | 0 | 0 | 181 | 0.002 | 0.128 | 0.969 | 0.02 | S |
| LEA2 | CotAD_11658 | 0.64 | 0.23 | 0.1 | 0.02 | 263 | 0.219 | 0.198 | 0.061 | 0.726 | _ |
| LEA2 | CotAD_31936 | 0 | 0.01 | 0.99 | 0 | 283 | 0.974 | 0.019 | 0.019 | 0.131 | C |
| LEA2 | CotAD_00799 | 1 | 0 | 0 | 0 | 337 | 0.004 | 0.117 | 0.971 | 0.017 | S |
| LEA2 | CotAD_00808 | 0.64 | 0.23 | 0.1 | 0.02 | 226 | 0.292 | 0.057 | 0.047 | 0.738 | _ |
| LEA2 | CotAD_10376 | 0.64 | 0.23 | 0.1 | 0.02 | 277 | 0.649 | 0.042 | 0.02 | 0.429 | C |
| LEA2 | CotAD_01385 | 0.46 | 0.29 | 0.09 | 0.16 | 247 | 0.303 | 0.125 | 0.043 | 0.729 | _ |
| LEA2 | CotAD_01700 | 0.64 | 0.24 | 0.1 | 0.02 | 260 | 0.626 | 0.041 | 0.031 | 0.601 | C |
| LEA2 | CotAD_08181 | 0.71 | 0.04 | 0 | 0.25 | 202 | 0.004 | 0.426 | 0.195 | 0.802 | _ |
| LEA2 | CotAD_11875 | 0.24 | 0.44 | 0.18 | 0.14 | 175 | 0.075 | 0.536 | 0.148 | 0.087 | M |
| LEA2 | CotAD_11878 | 0.44 | 0.16 | 0.06 | 0.34 | 226 | 0.043 | 0.07 | 0.111 | 0.898 | _ |
| LEA2 | CotAD_11879 | 0.02 | 0.18 | 0 | 0.79 | 129 | 0.017 | 0.552 | 0.041 | 0.794 | _ |
| LEA2 | CotAD_06037 | 0.32 | 0.33 | 0.14 | 0.21 | 205 | 0.317 | 0.127 | 0.042 | 0.072 | C |
| LEA2 | CotAD_27453 | 0.98 | 0.01 | 0 | 0.01 | 257 | 0.003 | 0.427 | 0.705 | 0.105 | S |
| LEA2 | CotAD_23824 | 0.09 | 0.58 | 0.08 | 0.25 | 263 | 0.121 | 0.189 | 0.013 | 0.432 | _ |
| LEA2 | CotAD_19375 | 0.83 | 0.07 | 0.03 | 0.06 | 225 | 0.035 | 0.048 | 0.583 | 0.199 | S |
| LEA2 | CotAD_37888 | 0 | 0.06 | 0.89 | 0.05 | 320 | 0.871 | 0.03 | 0.006 | 0.159 | C |
| LEA2 | CotAD_03037 | 0.64 | 0.23 | 0.1 | 0.02 | 262 | 0.24 | 0.287 | 0.003 | 0.371 | _ |
| LEA2 | CotAD_03649 | 0 | 0.05 | 0.9 | 0.05 | 320 | 0.839 | 0.045 | 0.009 | 0.161 | C |
| LEA2 | CotAD_44357 | 0.03 | 0.18 | 0.11 | 0.68 | 305 | 0.524 | 0.114 | 0.024 | 0.555 | _ |
| LEA2 | CotAD_05724 | 0.99 | 0 | 0 | 0 | 197 | 0.006 | 0.118 | 0.917 | 0.072 | S |
| LEA2 | CotAD_05725 | 0.55 | 0.31 | 0.08 | 0.05 | 238 | 0.114 | 0.435 | 0.002 | 0.608 | _ |
| LEA2 | CotAD_13115 | 1 | 0 | 0 | 0 | 192 | 0.001 | 0.181 | 0.982 | 0.061 | S |
| LEA2 | CotAD_03784 | 0.3 | 0.18 | 0.01 | 0.51 | 116 | 0.225 | 0.166 | 0.118 | 0.243 | _ |
| LEA2 | CotAD_20020 | 0.64 | 0.23 | 0.1 | 0.02 | 250 | 0.052 | 0.08 | 0.033 | 0.913 | _ |
| LEA2 | CotAD_13584 | 0.64 | 0.23 | 0.1 | 0.02 | 250 | 0.055 | 0.079 | 0.033 | 0.912 | _ |
| LEA2 | CotAD_20308 | 0.99 | 0 | 0 | 0.01 | 191 | 0.001 | 0.122 | 0.945 | 0.101 | S |
| LEA2 | CotAD_17103 | 0.01 | 0.07 | 0 | 0.92 | 265 | 0.283 | 0.123 | 0.085 | 0.829 | _ |
| LEA2 | CotAD_09685 | 0.64 | 0.24 | 0.1 | 0.02 | 251 | 0.144 | 0.351 | 0.003 | 0.58 | _ |
| LEA2 | CotAD_09732 | 0.75 | 0.2 | 0 | 0.05 | 232 | 0.003 | 0.614 | 0.05 | 0.268 | M |
| LEA2 | CotAD_16731 | 0.64 | 0.23 | 0.1 | 0.02 | 258 | 0.554 | 0.039 | 0.011 | 0.572 | _ |
| LEA2 | CotAD_15892 | 0.02 | 0.19 | 0.12 | 0.67 | 307 | 0.289 | 0.099 | 0.036 | 0.742 | _ |
| LEA2 | CotAD_21731 | 0.64 | 0.23 | 0.1 | 0.02 | 244 | 0.426 | 0.038 | 0.009 | 0.727 | _ |
| LEA2 | CotAD_08350 | 0.64 | 0.23 | 0.1 | 0.02 | 198 | 0.099 | 0.173 | 0.074 | 0.434 | _ |
| LEA2 | CotAD_40324 | 0.03 | 0.18 | 0.11 | 0.68 | 305 | 0.531 | 0.112 | 0.024 | 0.556 | _ |
| LEA2 | CotAD_29610 | 0.01 | 0.05 | 0 | 0.93 | 152 | 0.07 | 0.056 | 0.171 | 0.861 | _ |
| LEA2 | CotAD_28872 | 0.61 | 0.24 | 0.09 | 0.05 | 240 | 0.028 | 0.415 | 0.021 | 0.771 | _ |
| LEA2 | CotAD_41925 | 0.01 | 0.07 | 0.86 | 0.07 | 310 | 0.677 | 0.065 | 0.006 | 0.211 | C |
| LEA2 | CotAD_17044 | 0.01 | 0.06 | 0 | 0.93 | 151 | 0.104 | 0.052 | 0.152 | 0.856 | _ |
| LEA2 | CotAD_17045 | 0.64 | 0.23 | 0.1 | 0.02 | 219 | 0.017 | 0.101 | 0.042 | 0.958 | _ |
| LEA2 | CotAD_17062 | 0.64 | 0.23 | 0.1 | 0.02 | 244 | 0.357 | 0.036 | 0.01 | 0.784 | _ |
| LEA2 | CotAD_24497 | 0.14 | 0.51 | 0.23 | 0.12 | 175 | 0.117 | 0.585 | 0.1 | 0.048 | M |
| LEA2 | CotAD_24499 | 0.44 | 0.16 | 0.06 | 0.34 | 226 | 0.039 | 0.071 | 0.101 | 0.901 | _ |
| LEA2 | CotAD_13827 | 0.03 | 0.18 | 0 | 0.79 | 360 | 0.033 | 0.177 | 0.139 | 0.622 | _ |
| LEA2 | CotAD_09578 | 0.64 | 0.24 | 0.1 | 0.02 | 260 | 0.647 | 0.032 | 0.028 | 0.585 | C |
| LEA2 | CotAD_35069 | 0 | 0.02 | 0.97 | 0.01 | 318 | 0.813 | 0.05 | 0.01 | 0.126 | C |
| LEA2 | CotAD_35091 | 0.53 | 0.24 | 0.07 | 0.16 | 251 | 0.203 | 0.16 | 0.057 | 0.857 | _ |
| LEA2 | CotAD_42599 | 0.96 | 0 | 0 | 0.03 | 206 | 0.016 | 0.124 | 0.785 | 0.189 | S |
| LEA2 | CotAD_08837 | 0.64 | 0.23 | 0.1 | 0.02 | 245 | 0.105 | 0.251 | 0.005 | 0.527 | _ |
| LEA2 | CotAD_53438 | 0.63 | 0.1 | 0.03 | 0.24 | 206 | 0.082 | 0.198 | 0.055 | 0.898 | _ |
| LEA2 | CotAD_34798 | 0.64 | 0.23 | 0.1 | 0.02 | 259 | 0.537 | 0.04 | 0.008 | 0.55 | _ |
| LEA2 | CotAD_21924 | 0.64 | 0.23 | 0.1 | 0.02 | 262 | 0.108 | 0.111 | 0.001 | 0.847 | _ |
| LEA2 | CotAD_18729 | 0.64 | 0.23 | 0.1 | 0.02 | 277 | 0.663 | 0.04 | 0.022 | 0.423 | C |
| LEA2 | CotAD_18210 | 0.15 | 0.34 | 0.05 | 0.46 | 203 | 0.045 | 0.306 | 0.021 | 0.881 | _ |
| LEA2 | CotAD_18233 | 0.08 | 0.18 | 0.02 | 0.73 | 203 | 0.057 | 0.185 | 0.028 | 0.923 | _ |
| LEA2 | CotAD_35513 | 0.31 | 0.3 | 0.06 | 0.34 | 217 | 0.046 | 0.253 | 0.037 | 0.858 | _ |
| LEA2 | CotAD_35514 | 1 | 0 | 0 | 0 | 181 | 0.002 | 0.222 | 0.967 | 0.008 | S |
| LEA2 | CotAD_19078 | 0.06 | 0.11 | 0.01 | 0.82 | 216 | 0.039 | 0.047 | 0.024 | 0.968 | _ |
| LEA2 | CotAD_19107 | 0.46 | 0.39 | 0.02 | 0.13 | 183 | 0.011 | 0.239 | 0.472 | 0.064 | S |
| LEA2 | CotAD_19205 | 0.02 | 0.14 | 0.03 | 0.81 | 297 | 0.242 | 0.091 | 0.039 | 0.815 | _ |
| LEA2 | CotAD_27143 | 0.06 | 0.67 | 0.07 | 0.2 | 222 | 0.33 | 0.231 | 0.012 | 0.483 | _ |
| LEA2 | CotAD_48976 | 0.64 | 0.24 | 0.1 | 0.02 | 220 | 0.076 | 0.059 | 0.034 | 0.943 | _ |
| LEA2 | CotAD_26038 | 0.08 | 0.28 | 0.03 | 0.61 | 239 | 0.066 | 0.081 | 0.022 | 0.769 | _ |
| LEA2 | CotAD_31140 | 0.57 | 0.27 | 0.11 | 0.06 | 249 | 0.185 | 0.027 | 0.007 | 0.866 | _ |
| LEA2 | CotAD_56356 | 0.01 | 0.05 | 0 | 0.93 | 152 | 0.061 | 0.046 | 0.183 | 0.864 | _ |
| LEA2 | CotAD_59405 | 0.13 | 0.22 | 0.01 | 0.65 | 209 | 0.012 | 0.87 | 0.031 | 0.508 | M |
| LEA2 | CotAD_46888 | 0.1 | 0.22 | 0.09 | 0.6 | 191 | 0.122 | 0.074 | 0.088 | 0.097 | C |
| LEA2 | CotAD_31537 | 0.18 | 0.1 | 0 | 0.72 | 288 | 0.239 | 0.115 | 0.113 | 0.84 | _ |
| LEA2 | CotAD_23646 | 0.09 | 0.16 | 0.02 | 0.73 | 203 | 0.052 | 0.156 | 0.02 | 0.94 | _ |
| LEA2 | CotAD_68189 | 0.6 | 0.09 | 0.02 | 0.29 | 206 | 0.061 | 0.238 | 0.049 | 0.907 | _ |
| LEA2 | CotAD_47322 | 0.26 | 0.37 | 0.16 | 0.21 | 208 | 0.386 | 0.155 | 0.024 | 0.064 | C |
| LEA2 | CotAD_26668 | 0.41 | 0.42 | 0.01 | 0.15 | 184 | 0.01 | 0.269 | 0.334 | 0.134 | S |
| LEA2 | CotAD_26981 | 0.01 | 0.62 | 0.35 | 0.03 | 150 | 0.211 | 0.744 | 0.014 | 0.103 | M |
| LEA2 | CotAD_29279 | 0.08 | 0.28 | 0.02 | 0.62 | 232 | 0.035 | 0.126 | 0.02 | 0.849 | _ |
| LEA2 | CotAD_31860 | 1 | 0 | 0 | 0 | 450 | 0.012 | 0.022 | 0.982 | 0.024 | S |
| LEA2 | CotAD_31869 | 0.47 | 0.26 | 0.04 | 0.23 | 206 | 0.021 | 0.677 | 0.033 | 0.699 | _ |
| LEA2 | CotAD_44941 | 0.61 | 0.24 | 0.09 | 0.05 | 240 | 0.028 | 0.328 | 0.023 | 0.814 | _ |
| LEA2 | CotAD_56696 | 0.09 | 0.11 | 0.01 | 0.8 | 210 | 0.046 | 0.024 | 0.061 | 0.981 | _ |
| LEA2 | CotAD_56699 | 0.42 | 0.19 | 0.04 | 0.35 | 213 | 0.048 | 0.027 | 0.094 | 0.975 | _ |
| LEA2 | CotAD_43605 | 0 | 0.02 | 0.96 | 0.01 | 305 | 0.919 | 0.026 | 0.009 | 0.126 | C |
| LEA2 | CotAD_31782 | 0.64 | 0.24 | 0.1 | 0.02 | 206 | 0.101 | 0.149 | 0.076 | 0.474 | _ |
| LEA2 | CotAD_31344 | 0 | 0.05 | 0.91 | 0.04 | 320 | 0.81 | 0.032 | 0.005 | 0.214 | C |
| LEA2 | CotAD_68063 | 0.98 | 0.01 | 0 | 0.01 | 218 | 0.004 | 0.416 | 0.652 | 0.11 | S |
| LEA2 | CotAD_46550 | 0.81 | 0.06 | 0 | 0.13 | 202 | 0.004 | 0.604 | 0.186 | 0.597 | M |
| LEA2 | CotAD_33143 | 1 | 0 | 0 | 0 | 188 | 0.003 | 0.085 | 0.976 | 0.021 | S |
| LEA2 | CotAD_33144 | 0.64 | 0.23 | 0.1 | 0.02 | 373 | 0.237 | 0.095 | 0.05 | 0.721 | _ |
| LEA2 | CotAD_32487 | 0.96 | 0.01 | 0 | 0.03 | 210 | 0.004 | 0.258 | 0.6 | 0.173 | S |
| LEA2 | CotAD_70003 | 0.98 | 0 | 0 | 0.02 | 191 | 0.002 | 0.082 | 0.945 | 0.103 | S |
| LEA2 | CotAD_64120 | 0.55 | 0.19 | 0.08 | 0.18 | 218 | 0.035 | 0.066 | 0.078 | 0.963 | _ |
| LEA2 | CotAD_73966 | 0 | 0.06 | 0.89 | 0.05 | 320 | 0.871 | 0.031 | 0.007 | 0.161 | C |
| LEA2 | CotAD_39719 | 0.64 | 0.23 | 0.1 | 0.02 | 274 | 0.345 | 0.056 | 0.037 | 0.572 | _ |
| LEA2 | CotAD_51205 | 0.02 | 0.17 | 0.42 | 0.4 | 304 | 0.539 | 0.073 | 0.023 | 0.594 | _ |
| LEA2 | CotAD_48469 | 0.61 | 0.25 | 0.12 | 0.02 | 256 | 0.647 | 0.236 | 0.017 | 0.179 | C |
| LEA2 | CotAD_61173 | 0.44 | 0.17 | 0.06 | 0.33 | 215 | 0.038 | 0.112 | 0.034 | 0.948 | _ |
| LEA2 | CotAD_47454 | 0.54 | 0.27 | 0.15 | 0.04 | 270 | 0.765 | 0.108 | 0.03 | 0.15 | C |
| LEA2 | CotAD_61391 | 0.12 | 0.19 | 0.04 | 0.66 | 191 | 0.121 | 0.054 | 0.08 | 0.14 | _ |
| LEA2 | CotAD_41714 | 0.64 | 0.24 | 0.1 | 0.02 | 254 | 0.175 | 0.29 | 0 | 0.431 | _ |
| LEA2 | CotAD_49818 | 0.38 | 0.42 | 0.06 | 0.15 | 661 | 0.235 | 0.246 | 0.012 | 0.381 | _ |
| LEA2 | CotAD_62996 | 0 | 0.02 | 0.97 | 0.01 | 318 | 0.813 | 0.05 | 0.01 | 0.126 | C |
| LEA2 | CotAD_64657 | 0.64 | 0.23 | 0.1 | 0.02 | 262 | 0.145 | 0.093 | 0.001 | 0.824 | _ |
| LEA2 | CotAD_69737 | 0.41 | 0.19 | 0.04 | 0.35 | 213 | 0.045 | 0.039 | 0.074 | 0.966 | _ |
| LEA2 | CotAD_69738 | 0.1 | 0.11 | 0.01 | 0.79 | 210 | 0.027 | 0.037 | 0.071 | 0.985 | _ |
| LEA2 | CotAD_60435 | 0.12 | 0.19 | 0.02 | 0.67 | 251 | 0.169 | 0.185 | 0.037 | 0.864 | _ |
| LEA2 | CotAD_60279 | 0.64 | 0.23 | 0.1 | 0.02 | 247 | 0.066 | 0.305 | 0.002 | 0.517 | _ |
| LEA2 | CotAD_51667 | 0.01 | 0.05 | 0 | 0.94 | 317 | 0.086 | 0.093 | 0.1 | 0.937 | _ |
| LEA2 | CotAD_53981 | 0.64 | 0.24 | 0.1 | 0.02 | 251 | 0.119 | 0.409 | 0.002 | 0.548 | _ |
| LEA2 | CotAD_71431 | 1 | 0 | 0 | 0 | 186 | 0.003 | 0.048 | 0.981 | 0.044 | S |
| LEA2 | CotAD_72458 | 1 | 0 | 0 | 0 | 192 | 0.001 | 0.156 | 0.981 | 0.099 | S |
| LEA2 | CotAD_64004 | 0.64 | 0.24 | 0.1 | 0.02 | 219 | 0.02 | 0.092 | 0.036 | 0.954 | _ |
| LEA2 | CotAD_63174 | 0.01 | 0.07 | 0.88 | 0.05 | 377 | 0.82 | 0.044 | 0.004 | 0.189 | C |
| LEA2 | CotAD_66245 | 0.99 | 0 | 0 | 0 | 450 | 0.018 | 0.022 | 0.979 | 0.046 | S |
| LEA2 | CotAD_55224 | 0.45 | 0.29 | 0.09 | 0.17 | 247 | 0.239 | 0.134 | 0.061 | 0.758 | _ |
| LEA2 | CotAD_65370 | 0.01 | 0.09 | 0.84 | 0.07 | 326 | 0.827 | 0.034 | 0.004 | 0.244 | C |
| LEA2 | CotAD_64347 | 0.64 | 0.23 | 0.1 | 0.02 | 235 | 0.053 | 0.187 | 0.006 | 0.858 | _ |
| LEA2 | CotAD_65119 | 0.49 | 0.23 | 0.03 | 0.25 | 206 | 0.023 | 0.675 | 0.039 | 0.662 | M |
| LEA2 | CotAD_66774 | 0.06 | 0.12 | 0.01 | 0.81 | 216 | 0.058 | 0.041 | 0.025 | 0.956 | _ |
| LEA2 | CotAD_66775 | 0.64 | 0.23 | 0.1 | 0.02 | 225 | 0.055 | 0.071 | 0.025 | 0.834 | _ |
| LEA2 | CotAD_66551 | 0.64 | 0.23 | 0.1 | 0.02 | 225 | 0.06 | 0.14 | 0.059 | 0.92 | _ |
| LEA2 | CotAD_72913 | 0.01 | 0.05 | 0 | 0.94 | 315 | 0.086 | 0.093 | 0.1 | 0.937 | _ |
| LEA2 | CotAD_70190 | 0.03 | 0.12 | 0.01 | 0.85 | 430 | 0.148 | 0.145 | 0.095 | 0.611 | _ |
| LEA2 | CotAD_70192 | 0.01 | 0.05 | 0 | 0.94 | 130 | 0.075 | 0.104 | 0.115 | 0.941 | _ |
| LEA3 | CotAD_22633 | 0.47 | 0.2 | 0.2 | 0.12 | 100 | 0.117 | 0.058 | 0.833 | 0.016 | S |
| LEA3 | CotAD_04558 | 0.06 | 0.7 | 0.16 | 0.09 | 100 | 0.221 | 0.192 | 0.307 | 0.027 | S |
| LEA3 | CotAD_22634 | 0.07 | 0.63 | 0.19 | 0.11 | 99 | 0.208 | 0.175 | 0.388 | 0.024 | S |
| LEA3 | CotAD_04559 | 0.4 | 0.22 | 0.2 | 0.18 | 100 | 0.26 | 0.092 | 0.57 | 0.021 | S |
| LEA3 | CotAD_21416 | 0.13 | 0.63 | 0.17 | 0.07 | 92 | 0.747 | 0.424 | 0.102 | 0.006 | C |
| LEA3 | CotAD_48069 | 0.13 | 0.61 | 0.17 | 0.08 | 92 | 0.774 | 0.372 | 0.074 | 0.01 | C |
| LEA3 | CotAD_39233 | 0.05 | 0.84 | 0.01 | 0.09 | 98 | 0.114 | 0.378 | 0.327 | 0.026 | M |
| LEA3 | CotAD_57519 | 0.12 | 0.54 | 0.08 | 0.26 | 98 | 0.182 | 0.151 | 0.501 | 0.029 | S |
| LEA3 | CotAD_48753 | 0.15 | 0.64 | 0.13 | 0.08 | 105 | 0.093 | 0.244 | 0.354 | 0.026 | S |
| LEA3 | CotAD_47495 | 0.04 | 0.74 | 0.14 | 0.08 | 105 | 0.143 | 0.336 | 0.171 | 0.05 | M |
| LEA3 | CotAD_45390 | 0.01 | 0.95 | 0 | 0.04 | 85 | 0.016 | 0.779 | 0.191 | 0.098 | M |
| LEA3 | CotAD_01504 | 0.04 | 0.34 | 0.25 | 0.38 | 93 | 0.437 | 0.309 | 0.091 | 0.242 | C |
| LEA3 | CotAD_31906 | 0 | 0.58 | 0.42 | 0 | 126 | 0.504 | 0.771 | 0.022 | 0.019 | M |
| LEA3 | CotAD_24019 | 0 | 0.58 | 0.42 | 0 | 126 | 0.518 | 0.767 | 0.022 | 0.019 | M |
| LEA3 | CotAD_33003 | 0.02 | 0.82 | 0 | 0.16 | 124 | 0.025 | 0.671 | 0.039 | 0.325 | M |
| LEA3 | CotAD_31255 | 0.02 | 0.75 | 0.01 | 0.22 | 120 | 0.154 | 0.55 | 0.033 | 0.172 | M |
| LEA4 | CotAD_23118 | 0.02 | 0.1 | 0 | 0.88 | 405 | 0.075 | 0.453 | 0.036 | 0.606 | _ |
| LEA4 | CotAD_74061 | 0.02 | 0.1 | 0 | 0.88 | 405 | 0.08 | 0.47 | 0.033 | 0.566 | _ |
| LEA4 | CotAD_02872 | 0.02 | 0.12 | 0 | 0.86 | 569 | 0.059 | 0.455 | 0.02 | 0.646 | _ |
| LEA4 | CotAD_62659 | 0.02 | 0.11 | 0 | 0.87 | 568 | 0.071 | 0.32 | 0.024 | 0.719 | _ |
| LEA4 | CotAD_22539 | 0.02 | 0.1 | 0 | 0.88 | 136 | 0.106 | 0.202 | 0.065 | 0.823 | _ |
| LEA4 | CotAD_05963 | 0.99 | 0 | 0 | 0 | 266 | 0.062 | 0.05 | 0.877 | 0.066 | S |
| LEA4 | CotAD_36583 | 0.99 | 0 | 0 | 0.01 | 284 | 0.096 | 0.054 | 0.78 | 0.078 | S |
| LEA4 | CotAD_00667 | 0 | 0.17 | 0.82 | 0 | 239 | 0.702 | 0.409 | 0.107 | 0.009 | C |
| LEA4 | CotAD_62314 | 0 | 0.23 | 0.76 | 0.01 | 239 | 0.651 | 0.416 | 0.134 | 0.008 | C |
| LEA4 | CotAD_09404 | 0.03 | 0.2 | 0.59 | 0.18 | 127 | 0.707 | 0.287 | 0.08 | 0.032 | C |
| LEA4 | CotAD_09405 | 0.03 | 0.67 | 0.01 | 0.28 | 109 | 0.2 | 0.584 | 0.035 | 0.123 | M |
| LEA4 | CotAD_13989 | 0.03 | 0.72 | 0.02 | 0.23 | 109 | 0.272 | 0.576 | 0.028 | 0.109 | M |
| LEA4 | CotAD_10044 | 0.03 | 0.16 | 0.02 | 0.8 | 634 | 0.233 | 0.162 | 0.044 | 0.703 | _ |
| LEA5 | CotAD_03264 | 0.02 | 0.1 | 0 | 0.88 | 110 | 0.149 | 0.315 | 0.037 | 0.663 | _ |
| LEA5 | CotAD_45324 | 0.02 | 0.1 | 0 | 0.88 | 110 | 0.142 | 0.321 | 0.038 | 0.669 | _ |
| LEA5 | CotAD_57587 | 0.01 | 0.07 | 0 | 0.92 | 94 | 0.192 | 0.207 | 0.032 | 0.747 | _ |
| LEA5 | CotAD_56728 | 0.01 | 0.07 | 0 | 0.92 | 94 | 0.192 | 0.207 | 0.032 | 0.747 | _ |
| LEA5 | CotAD_22357 | 0.01 | 0.06 | 0 | 0.92 | 102 | 0.275 | 0.11 | 0.078 | 0.792 | _ |
| LEA5 | CotAD_43455 | 0.01 | 0.06 | 0 | 0.92 | 102 | 0.275 | 0.11 | 0.078 | 0.792 | _ |
| LEA5 | CotAD_07516 | 0.01 | 0.06 | 0 | 0.93 | 123 | 0.074 | 0.148 | 0.115 | 0.892 | _ |
| LEA5 | CotAD_35021 | 0.01 | 0.05 | 0 | 0.94 | 144 | 0.087 | 0.126 | 0.111 | 0.906 | _ |
| LEA5 | CotAD_50983 | 0.01 | 0.05 | 0 | 0.93 | 171 | 0.068 | 0.111 | 0.08 | 0.912 | _ |
| LEA6 | CotAD_19623 | 0.01 | 0.05 | 0 | 0.94 | 94 | 0.172 | 0.077 | 0.106 | 0.91 | _ |
| LEA6 | CotAD_36999 | 0.01 | 0.05 | 0 | 0.94 | 94 | 0.148 | 0.076 | 0.109 | 0.909 | _ |
| LEA6 | CotAD_13789 | 0.02 | 0.08 | 0 | 0.9 | 86 | 0.17 | 0.147 | 0.087 | 0.788 | _ |
| LEA6 | CotAD_48336 | 0.02 | 0.8 | 0.07 | 0.11 | 114 | 0.185 | 0.566 | 0.003 | 0.186 | M |
| SMP | CotAD_32645 | 0.01 | 0.06 | 0 | 0.93 | 171 | 0.092 | 0.113 | 0.066 | 0.933 | _ |
| SMP | CotAD_12680 | 0.02 | 0.14 | 0 | 0.83 | 169 | 0.029 | 0.432 | 0.068 | 0.795 | _ |
| SMP | CotAD_12681 | 0.01 | 0.06 | 0 | 0.93 | 144 | 0.069 | 0.148 | 0.1 | 0.872 | _ |
| SMP | CotAD_12682 | 0.01 | 0.06 | 0 | 0.92 | 258 | 0.161 | 0.169 | 0.047 | 0.832 | _ |
| SMP | CotAD_11594 | 0.02 | 0.13 | 0 | 0.85 | 264 | 0.113 | 0.457 | 0.017 | 0.616 | _ |
| SMP | CotAD_48050 | 0.01 | 0.06 | 0 | 0.93 | 261 | 0.108 | 0.108 | 0.052 | 0.931 | _ |
| SMP | CotAD_34476 | 0.01 | 0.06 | 0 | 0.93 | 252 | 0.139 | 0.115 | 0.049 | 0.885 | _ |
| SMP | CotAD_53045 | 0.01 | 0.06 | 0 | 0.93 | 253 | 0.144 | 0.144 | 0.033 | 0.845 | _ |
| SMP | CotAD_66708 | 0.01 | 0.06 | 0 | 0.93 | 258 | 0.129 | 0.181 | 0.044 | 0.887 | _ |
| SMP | CotAD_67721 | 0.02 | 0.13 | 0 | 0.85 | 264 | 0.1 | 0.466 | 0.016 | 0.616 | _ |
| DEHYDRIN | CotAD_15928 | 0.01 | 0.05 | 0 | 0.94 | 180 | 0.053 | 0.176 | 0.038 | 0.929 | _ |
| DEHYDRIN | CotAD_19173 | 0.01 | 0.05 | 0 | 0.93 | 180 | 0.052 | 0.149 | 0.032 | 0.942 | _ |
| DEHYDRIN | CotAD_08352 | 0.01 | 0.06 | 0 | 0.93 | 160 | 0.306 | 0.065 | 0.051 | 0.907 | _ |
| DEHYDRIN | CotAD_31780 | 0.01 | 0.05 | 0 | 0.93 | 161 | 0.301 | 0.066 | 0.053 | 0.907 | _ |
| DEHYDRIN | CotAD_25271 | 0.02 | 0.07 | 0 | 0.91 | 135 | 0.185 | 0.139 | 0.053 | 0.767 | _ |
| DEHYDRIN | CotAD_48769 | 0.02 | 0.07 | 0 | 0.91 | 135 | 0.213 | 0.13 | 0.047 | 0.761 | _ |
| DEHYDRIN | CotAD_27789 | 0.01 | 0.05 | 0 | 0.94 | 172 | 0.122 | 0.104 | 0.081 | 0.933 | _ |
| DEHYDRIN | CotAD_38978 | 0.01 | 0.05 | 0 | 0.94 | 197 | 0.154 | 0.081 | 0.083 | 0.924 | _ |
| DEHYDRIN | CotAD_40972 | 0.01 | 0.05 | 0 | 0.94 | 199 | 0.162 | 0.092 | 0.083 | 0.903 | _ |
| DEHYDRIN | CotAD_54337 | 0.01 | 0.05 | 0 | 0.94 | 211 | 0.251 | 0.084 | 0.092 | 0.875 | _ |
| DEHYDRIN | CotAD_58358 | 0.01 | 0.05 | 0 | 0.94 | 211 | 0.225 | 0.088 | 0.098 | 0.879 | _ |
| DEHYDRIN | CotAD_04417 | 0.01 | 0.05 | 0 | 0.94 | 98 | 0.142 | 0.107 | 0.12 | 0.907 | _ |
| DEHYDRIN | CotAD_64203 | 0.01 | 0.05 | 0 | 0.94 | 178 | 0.131 | 0.102 | 0.132 | 0.92 | _ |
| DEHYDRIN | CotAD_13947 | 0.02 | 0.16 | 0 | 0.82 | 449 | 0.007 | 0.686 | 0.029 | 0.844 | _ |
| DEHYDRIN | CotAD_16331 | 0.01 | 0.06 | 0 | 0.93 | 128 | 0.206 | 0.115 | 0.017 | 0.889 | _ |
| DEHYDRIN | CotAD_65889 | 0.01 | 0.05 | 0 | 0.93 | 608 | 0.085 | 0.103 | 0.098 | 0.927 | _ |
| DEHYDRIN | CotAD_75537 | 0.01 | 0.05 | 0 | 0.93 | 533 | 0.087 | 0.105 | 0.1 | 0.925 | _ |
| DEHYDRIN | CotAD_75267 | 0.01 | 0.07 | 0 | 0.91 | 332 | 0.069 | 0.094 | 0.039 | 0.927 | _ |
| DEHYDRIN | CotAD_70948 | 0.01 | 0.07 | 0 | 0.91 | 332 | 0.069 | 0.095 | 0.039 | 0.928 | _ |
| DEHYDRIN | CotAD_10502 | 0.03 | 0.1 | 0.01 | 0.86 | 235 | 0.077 | 0.158 | 0.143 | 0.531 | _ |
| DEHYDRIN | CotAD_11398 | 0.19 | 0.23 | 0.05 | 0.53 | 51 | 0.2 | 0.256 | 0.449 | 0.038 | S |
| DEHYDRIN | CotAD_47749 | 0.02 | 0.07 | 0 | 0.9 | 344 | 0.095 | 0.069 | 0.017 | 0.916 | _ |
| DEHYDRIN | CotAD_07367 | 0.02 | 0.14 | 0.01 | 0.82 | 1431 | 0.274 | 0.142 | 0.123 | 0.636 | _ |
| DEHYDRIN | CotAD_52203 | 0.02 | 0.13 | 0 | 0.85 | 243 | 0.019 | 0.296 | 0.058 | 0.885 | _ |

The color scheme indicates where the genes are sublocalized**.** SP: Secretory pathway (presence of a signal peptide); mTP: mitochondrial targeting peptide; cTP: chloroplast transit peptide; Other (nucleus, cytoplasmic, or otherwise). C: cytoplasm; S: secretory pathway; M: mitochondrion and - : others/other cell organelles.
