# Supplementary figures and images for: Characterization of the late embryogenesis abundant (LEA) proteins family and their role in drought stress tolerance in upland cotton
Source: BMC Genet. 2018 Jan 15;19:6. doi: 10.1186/s12863-017-0596-1 (PMC5769447; doi:10.1186/s12863-017-0596-1)

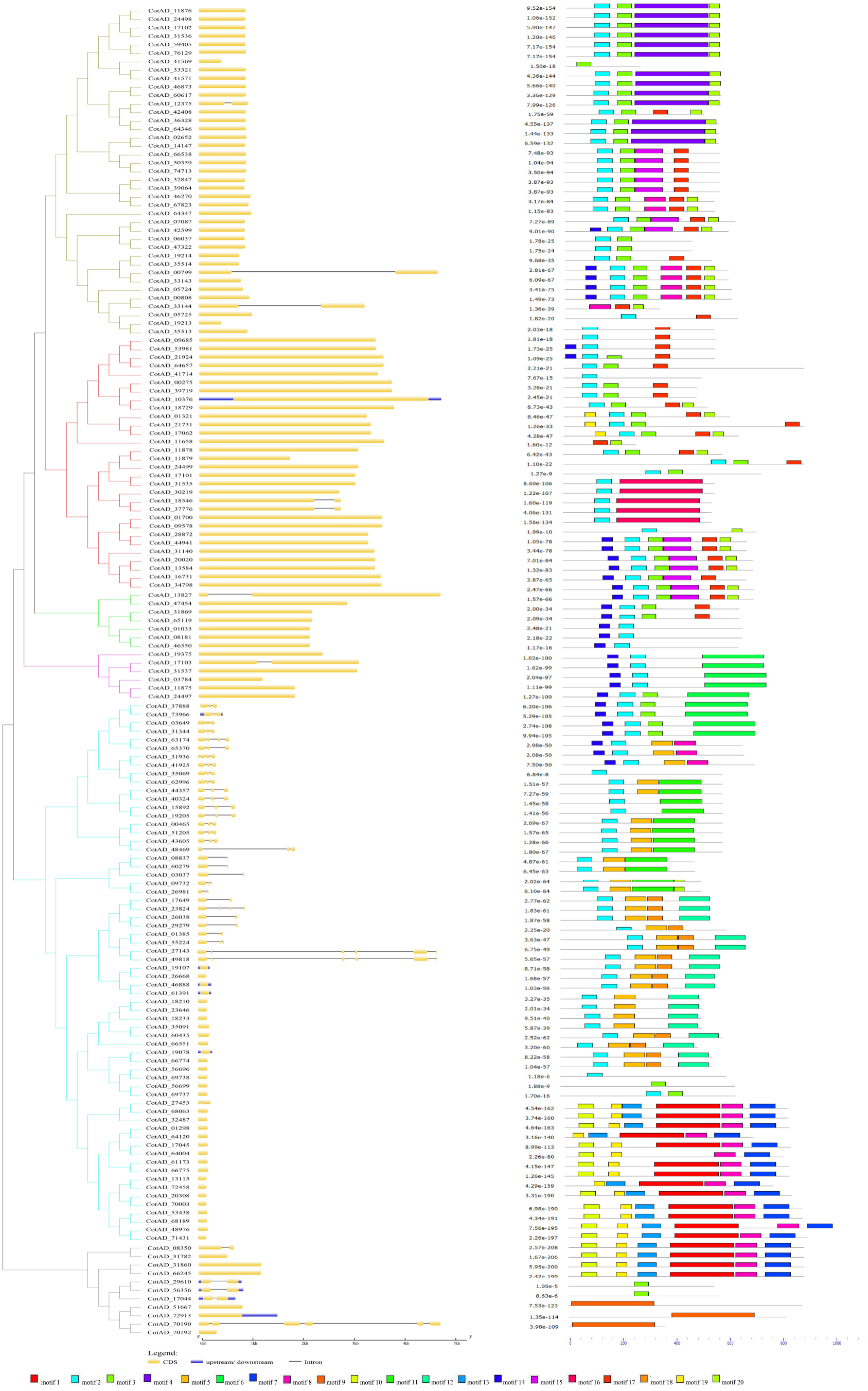

Supplement: Supplementary file 3 — Phylogenetic tree, gene structure and motif compositions of LEA 2 genes in upland cotton. The phylogenetic tree was constructed using MEGA 6.0. Exon/intron structures of LEA genes in upland cotton, exons introns and up / down-stream were represented by yellow boxes, black lines and blue boxes, respectively. Protein motif analysis represented by different colours, and each motif represented by number. (PDF 3609 kb) [file 12863_2017_596_MOESM3_ESM.pdf]
