# Supplementary material for: Characterization of the late embryogenesis abundant (LEA) proteins family and their role in drought stress tolerance in upland cotton
Source: BMC Genet. 2018 Jan 15;19:6. doi: 10.1186/s12863-017-0596-1 (PMC5769447; doi:10.1186/s12863-017-0596-1)
Supplement: Supplementary file 4 — LEA genes and mRNA targets. (DOCX 53 kb) [file 12863_2017_596_MOESM4_ESM.docx]

Supplementary Table 3: *LEA* genes and miRNA targets

| miRNA_targets. | Target genes | Expectation | miRNA start | miRNA end | Target start | Target end | miRNA_aligned_fragment | Target_aligned_fragment | Inhibition |
| --- | --- | --- | --- | --- | --- | --- | --- | --- | --- |
| ghr-miR2949a-3p | CotAD_00275 | 5 | 1 | 21 | 594 | 614 | UGCAAAUCCAGUCAAAAGUUA | CAAUUUGAGACCGGAUUUGAA | Translation |
| ghr-miR7504a | CotAD_00275 | 4 | 1 | 24 | 690 | 713 | UAUGAAACUGUGAUUCCACGUCAU | GAAAGUUGGGAUCAGGGUUUCGUG | Translation |
| ghr-miR7495a | CotAD_00667 | 4 | 1 | 21 | 449 | 469 | UUACUUUAGAUGUCUCCUUCA | UGAAAGAGAAAUCUAAAGAGA | Cleavage |
| ghr-miR7495a | CotAD_00667 | 5 | 1 | 21 | 242 | 262 | UUACUUUAGAUGUCUCCUUCA | CUAAGGAGAAAACGAAAGGAG | Translation |
| ghr-miR7495b | CotAD_00667 | 4 | 1 | 21 | 449 | 469 | UUACUUUAGAUGUCUCCUUCA | UGAAAGAGAAAUCUAAAGAGA | Cleavage |
| ghr-miR7495b | CotAD_00667 | 5 | 1 | 21 | 242 | 262 | UUACUUUAGAUGUCUCCUUCA | CUAAGGAGAAAACGAAAGGAG | Translation |
| ghr-miR2948-5p | CotAD_00799 | 4.5 | 1 | 22 | 475 | 496 | UGUGGGAGAGUUGGGCAAGAAU | UGGCUUGUCCAGCUUGUUUACA | Cleavage |
| ghr-miR7492a | CotAD_00799 | 4 | 1 | 23 | 865 | 887 | CUAUAGAACAUGAUCUUUAGCGG | UCGACAAAGAUCGUGUACUAUAC | Cleavage |
| ghr-miR7492b | CotAD_00799 | 4 | 1 | 23 | 865 | 887 | CUAUAGAACAUGAUCUUUAGCGG | UCGACAAAGAUCGUGUACUAUAC | Cleavage |
| ghr-miR7492c | CotAD_00799 | 4 | 1 | 23 | 865 | 887 | CUAUAGAACAUGAUCUUUAGCGG | UCGACAAAGAUCGUGUACUAUAC | Cleavage |
| ghr-miR7494 | CotAD_00799 | 5 | 1 | 23 | 607 | 629 | AGCUUGUGGACUAGUUUUAACAA | CAUGGAAGAAUAGAGUACAAGCU | Translation |
| ghr-miR7510b | CotAD_00799 | 5 | 1 | 23 | 862 | 884 | AAGAACAUGAUCUUUAGCGGCGU | UUGUCGACAAAGAUCGUGUACUA | Cleavage |
| ghr-miR7499 | CotAD_01298 | 5 | 1 | 24 | 537 | 559 | AUAUAAUUUUCGGUUAAUUCGGUU | GAUC-AAUAUAUUGAACAUUAUAA | Cleavage |
| ghr-miR2950 | CotAD_01385 | 5 | 1 | 21 | 335 | 355 | UGGUGUGCAGGGGGUGGAAUA | ACGUCUACUCCAUGGACAUCA | Translation |
| ghr-miR7495a | CotAD_01700 | 4.5 | 1 | 21 | 692 | 712 | UUACUUUAGAUGUCUCCUUCA | UUAAGGUGACGUGUAAGAUAA | Translation |
| ghr-miR7495b | CotAD_01700 | 4.5 | 1 | 21 | 692 | 712 | UUACUUUAGAUGUCUCCUUCA | UUAAGGUGACGUGUAAGAUAA | Translation |
| ghr-miR7493 | CotAD_02872 | 4.5 | 1 | 24 | 1322 | 1345 | AAUAUUUUAAUAAUUCAAUCGUCA | GUAAGAUUGGAGAAUUGAAAGAUU | Cleavage |
| ghr-miR7511 | CotAD_02872 | 5 | 1 | 24 | 583 | 606 | AGAAGUUUUGCAUG-UGUAGCUGAG | GACA-CUGUAACAGGUAAAGCUUCU | Cleavage |
| ghr-miR394a | CotAD_03264 | 5 | 1 | 20 | 244 | 263 | UUGGCAUUCUGUCCACCUCC | AAAGGUGGACUGAGUACCAU | Translation |
| ghr-miR394b | CotAD_03264 | 5 | 1 | 20 | 244 | 263 | UUGGCAUUCUGUCCACCUCC | AAAGGUGGACUGAGUACCAU | Translation |
| ghr-miR164 | CotAD_03784 | 5 | 1 | 21 | 11 | 31 | UGGAGAAGCAGGGCACGUGCA | UUAACGUAUCCUGUUUCUUAA | Cleavage |
| ghr-miR7510a | CotAD_06037 | 4.5 | 1 | 24 | 417 | 440 | AAGGUCAUGAUCUUUAGCGGCGUU | GACGGCGGUGAAGUUUAAGACCUU | Translation |
| ghr-miR7510a | CotAD_07087 | 5 | 1 | 24 | 579 | 602 | AAGGUCAUGAUCUUUAGCGGCGUU | AUCUUUGCUGAAUUUCUUGGUCUU | Translation |
| ghr-miR156c | CotAD_07367 | 4.5 | 1 | 20 | 3530 | 3549 | UGUCAGAAGAGAGUGAGCAC | CUGUUCGAUUUCUUUUUACA | Cleavage |
| ghr-miR399d | CotAD_07367 | 4.5 | 1 | 21 | 3858 | 3878 | UGCCAAAGGAGAUUUGCCCUG | UGCUGCAAAGUUCUUUUGGUA | Cleavage |
| ghr-miR399e | CotAD_07367 | 4.5 | 1 | 21 | 3858 | 3878 | UGCCAAAGGAGAUUUGCCCCG | UGCUGCAAAGUUCUUUUGGUA | Cleavage |
| ghr-miR7487 | CotAD_07367 | 5 | 1 | 24 | 617 | 649 | AUACUCUUAUAGGACACUUG---------UUAA | UUGAUACUGCUCUCAAGUGUUCUGGGACAGUAA | Translation |
| ghr-miR7495a | CotAD_07367 | 5 | 1 | 21 | 3246 | 3266 | UUACUUUAGAUGUCUCCUUCA | UCAAGGAGUUAGCAAAAUUAA | Translation |
| ghr-miR7495b | CotAD_07367 | 5 | 1 | 21 | 3246 | 3266 | UUACUUUAGAUGUCUCCUUCA | UCAAGGAGUUAGCAAAAUUAA | Translation |
| ghr-miR7497 | CotAD_07367 | 4.5 | 1 | 23 | 3754 | 3776 | ACAUGUGGACUGUCAUAUGGGUU | AUGCCAUAUGCCAUUUCGCAGGU | Translation |
| ghr-miR7500 | CotAD_07367 | 4.5 | 1 | 24 | 2810 | 2833 | AUCGAGUUAUUCGAGUUAAUCGAG | AAACAUUUGCUCGAAUAUCUGGAU | Cleavage |
| ghr-miR164 | CotAD_07516 | 5 | 1 | 21 | 257 | 278 | UGGAGAAGCA-GGGCACGUGCA | UACACGUGCCUGUCCAUCUCCA | Translation |
| ghr-miR7504a | CotAD_08352 | 5 | 1 | 24 | 338 | 361 | UAUGAAACUGUGAUUCCACGUCAU | AUGAGGAGGAGGAGCAAUUUCAUA | Cleavage |
| ghr-miR7504b | CotAD_09404 | 5 | 1 | 24 | 61 | 84 | AGGAGGAAAAAUCUGAUUUGUCAU | GUUAGGAAUAGGGUUCUUCCACCU | Translation |
| ghr-miR7495a | CotAD_09578 | 4.5 | 1 | 21 | 692 | 712 | UUACUUUAGAUGUCUCCUUCA | UUAAGGUGACGUGUAAGAUAA | Translation |
| ghr-miR7495b | CotAD_09578 | 4.5 | 1 | 21 | 692 | 712 | UUACUUUAGAUGUCUCCUUCA | UUAAGGUGACGUGUAAGAUAA | Translation |
| ghr-miR7495a | CotAD_10044 | 4 | 1 | 21 | 1157 | 1177 | UUACUUUAGAUGUCUCCUUCA | GCAAGGAGGCAGCUGAAUUAG | Translation |
| ghr-miR7495b | CotAD_10044 | 4 | 1 | 21 | 1157 | 1177 | UUACUUUAGAUGUCUCCUUCA | GCAAGGAGGCAGCUGAAUUAG | Translation |
| ghr-miR3476-5p | CotAD_11878 | 5 | 1 | 21 | 227 | 247 | UGAACUGGGUUUGUUGGCUGC | ACGUCCAACUCACCCAAUUCA | Translation |
| ghr-miR7491 | CotAD_13827 | 5 | 1 | 24 | 79 | 103 | UGGGAUCUUCGAGAGGAU-UGAGCC | AACCCACAUUUUAUCGAAGAUCCUG | Cleavage |
| ghr-miR7504a | CotAD_13827 | 4 | 1 | 24 | 1013 | 1034 | UAUGAAACUGUGAUUCCACGUCAU | AU--UGUGGCAUCAUAGUUUCAAG | Cleavage |
| ghr-miR7514 | CotAD_13827 | 5 | 1 | 24 | 253 | 276 | AUAAAGUGAUAAGUGAGAUCGUCU | AGCCAACCUCACCUGCCACUUCAU | Translation |
| ghr-miR7486a | CotAD_13947 | 5 | 1 | 24 | 232 | 255 | AAGGAAGCGCUUUGUCCACGUGGA | AGUAUCUGUACAGAGUGUUUUCUG | Cleavage |
| ghr-miR7486b | CotAD_13947 | 5 | 1 | 24 | 232 | 255 | AAGGAAGCGCUUUGUCCACGUGGA | AGUAUCUGUACAGAGUGUUUUCUG | Cleavage |
| ghr-miR7498 | CotAD_13947 | 5 | 1 | 24 | 792 | 820 | AUGGUGACACAUGGUAGUCUC-----ACA | UGUUUCAGGAGAUGGAUAUGUAUUACCAU | Cleavage |
| ghr-miR2950 | CotAD_15892 | 4.5 | 1 | 21 | 563 | 583 | UGGUGUGCAGGGGGUGGAAUA | CUUUCUAUGCCUUCCACGUCA | Cleavage |
| ghr-miR7492a | CotAD_15892 | 4 | 1 | 23 | 820 | 842 | CUAUAGAACAUGAUCUUUAGCGG | AUGGUGAAAACCAAGUUCUAUGG | Translation |
| ghr-miR7492b | CotAD_15892 | 4 | 1 | 23 | 820 | 842 | CUAUAGAACAUGAUCUUUAGCGG | AUGGUGAAAACCAAGUUCUAUGG | Translation |
| ghr-miR7492c | CotAD_15892 | 4 | 1 | 23 | 820 | 842 | CUAUAGAACAUGAUCUUUAGCGG | AUGGUGAAAACCAAGUUCUAUGG | Translation |
| ghr-miR396a | CotAD_16331 | 5 | 1 | 21 | 46 | 66 | UUCCACAGCUUUCUUGAACUG | GAGUUCAAACGGGCUGAGGAG | Cleavage |
| ghr-miR396b | CotAD_16331 | 5 | 1 | 21 | 46 | 66 | UUCCACAGCUUUCUUGAACUG | GAGUUCAAACGGGCUGAGGAG | Cleavage |
| ghr-miR7486a | CotAD_16331 | 5 | 1 | 24 | 94 | 117 | AAGGAAGCGCUUUGUCCACGUGGA | UCAAAGUGGGCAAGGCGUAUUUUG | Cleavage |
| ghr-miR7486b | CotAD_16331 | 5 | 1 | 24 | 94 | 117 | AAGGAAGCGCUUUGUCCACGUGGA | UCAAAGUGGGCAAGGCGUAUUUUG | Cleavage |
| ghr-miR3476-3p | CotAD_16594 | 5 | 1 | 21 | 115 | 135 | AGCCAACAACAUCAGUUCUAA | GAAGAAGAGAAGGAGUUGGCU | Translation |
| ghr-miR3476-3p | CotAD_16595 | 5 | 1 | 21 | 115 | 135 | AGCCAACAACAUCAGUUCUAA | GAAGAAGAGAAGGAGUUGGCU | Translation |
| ghr-miR2949a-5p | CotAD_17045 | 4.5 | 1 | 22 | 616 | 637 | ACUUUUGAACUGGAUUUGCCGA | CAGGCAAUUCAAGAACAGAAGU | Translation |
| ghr-miR2949a-5p | CotAD_17101 | 4 | 1 | 22 | 572 | 593 | ACUUUUGAACUGGAUUUGCCGA | CUGGCAGGUUCAGGCCAAAGGU | Translation |
| ghr-miR2949b | CotAD_17101 | 5 | 1 | 22 | 572 | 593 | UCUUUUGAACUGGAUUUGCCGA | CUGGCAGGUUCAGGCCAAAGGU | Translation |
| ghr-miR2949c | CotAD_17101 | 5 | 1 | 22 | 572 | 593 | UCUUUUGAACUGGAUUUGCCGA | CUGGCAGGUUCAGGCCAAAGGU | Translation |
| ghr-miR482a | CotAD_17186 | 4.5 | 1 | 22 | 112 | 134 | UCUUUCCUACUCCUC-CCAUACC | GAGAUGGCGAGGGAAAGGAAAGA | Translation |
| ghr-miR7507 | CotAD_17649 | 4.5 | 1 | 24 | 113 | 138 | AAGGUAGUGAAGUAGGCAAU--UGGG | ACUACCGUUGCCUCCGCCACUGCCUC | Translation |
| ghr-miR7510a | CotAD_17649 | 4.5 | 1 | 24 | 523 | 546 | AAGGUCAUGAUCUUUAGCGGCGUU | AAGGUUGAUGGAGAUCUUGGUCUU | Cleavage |
| ghr-miR7510b | CotAD_17649 | 5 | 1 | 23 | 524 | 546 | AAGAACAUGAUCUUUAGCGGCGU | AGGUUGAUGGAGAUCUUGGUCUU | Cleavage |
| ghr-miR7504a | CotAD_19107 | 5 | 1 | 24 | 438 | 459 | UAUGAAACUGUGAUUCCACGUCAU | GUG--GUGGGGUCAUAGGUUUAAA | Cleavage |
| ghr-miR390a | CotAD_19205 | 4.5 | 1 | 21 | 553 | 573 | AAGCUCAGGAGGGAUAGCGCC | ACCUCUACCCCUUUAGAGCUU | Cleavage |
| ghr-miR390b | CotAD_19205 | 4.5 | 1 | 21 | 553 | 573 | AAGCUCAGGAGGGAUAGCGCC | ACCUCUACCCCUUUAGAGCUU | Cleavage |
| ghr-miR390c | CotAD_19205 | 4.5 | 1 | 21 | 553 | 573 | AAGCUCAGGAGGGAUAGCGCC | ACCUCUACCCCUUUAGAGCUU | Cleavage |
| ghr-miR7492a | CotAD_19205 | 4 | 1 | 23 | 790 | 812 | CUAUAGAACAUGAUCUUUAGCGG | AUGGUGAAAACCAAGUUCUAUGG | Translation |
| ghr-miR7492b | CotAD_19205 | 4 | 1 | 23 | 790 | 812 | CUAUAGAACAUGAUCUUUAGCGG | AUGGUGAAAACCAAGUUCUAUGG | Translation |
| ghr-miR7492c | CotAD_19205 | 4 | 1 | 23 | 790 | 812 | CUAUAGAACAUGAUCUUUAGCGG | AUGGUGAAAACCAAGUUCUAUGG | Translation |
| ghr-miR482b | CotAD_19214 | 5 | 1 | 22 | 478 | 499 | UCUUGCCUACUCCACCCAUGCC | CCCAUUGGUAAAGAUGGCAAGA | Translation |
| ghr-miR164 | CotAD_19375 | 5 | 1 | 21 | 101 | 121 | UGGAGAAGCAGGGCACGUGCA | UGUCUGUGCCCUUCUUUUUCU | Translation |
| ghr-miR7496a | CotAD_19375 | 4.5 | 1 | 24 | 185 | 208 | AUGACCAAAUUGAUAGAAUGUGUA | CAAAUCUUGUAUCGAUUUGGUUGG | Cleavage |
| ghr-miR7496b | CotAD_19375 | 4.5 | 1 | 24 | 185 | 208 | AUGACCAAAUUGAUAGAAUGUGUA | CAAAUCUUGUAUCGAUUUGGUUGG | Cleavage |
| ghr-miR482a | CotAD_20491 | 4.5 | 1 | 22 | 112 | 134 | UCUUUCCUACUCCUC-CCAUACC | GAGAUGGCGAGGGAAAGGAAAGA | Translation |
| ghr-miR7510b | CotAD_21924 | 5 | 1 | 23 | 165 | 187 | AAGAACAUGAUCUUUAGCGGCGU | CCGCCGCUCAUGCUGUUGUUCUU | Translation |
| ghr-miR482b | CotAD_23118 | 4.5 | 1 | 22 | 717 | 738 | UCUUGCCUACUCCACCCAUGCC | GGAAGCGGCGGGGAAGGCAAGA | Translation |
| ghr-miR7506 | CotAD_23646 | 4.5 | 1 | 24 | 107 | 130 | AUGUCUGGGACAUGGCGUUGGCAC | CAGCUUUCGCCAUUUUCCAGACAA | Translation |
| ghr-miR7507 | CotAD_23824 | 4.5 | 1 | 24 | 113 | 138 | AAGGUAGUGAAGUAGGCAAU--UGGG | ACUACCGUUGCCUCCGCCACUGCCUC | Translation |
| ghr-miR7510a | CotAD_23824 | 4.5 | 1 | 24 | 520 | 543 | AAGGUCAUGAUCUUUAGCGGCGUU | AAGGUUGAUGGAGAUCUUGGUCUU | Cleavage |
| ghr-miR7510b | CotAD_23824 | 5 | 1 | 23 | 521 | 543 | AAGAACAUGAUCUUUAGCGGCGU | AGGUUGAUGGAGAUCUUGGUCUU | Cleavage |
| ghr-miR164 | CotAD_24497 | 5 | 1 | 21 | 77 | 97 | UGGAGAAGCAGGGCACGUGCA | UUAACGUAUCCUGUUUCUUAA | Cleavage |
| ghr-miR3476-5p | CotAD_24499 | 5 | 1 | 21 | 227 | 247 | UGAACUGGGUUUGUUGGCUGC | ACGUCCAACUCACCCAAUUCA | Translation |
| ghr-miR7504a | CotAD_26668 | 4.5 | 1 | 24 | 438 | 459 | UAUGAAACUGUGAUUCCACGUCAU | GUG--GUGGGGUCAUAGGUUCAAA | Cleavage |
| ghr-miR7494 | CotAD_27143 | 5 | 1 | 23 | 536 | 558 | AGCUUGUGGACUAGUUUUAACAA | CCUUUGAUACUGUUACGCAAGUU | Translation |
| ghr-miR7498 | CotAD_27143 | 5 | 1 | 24 | 101 | 124 | AUGGUGACACAUGGUAGUCUCACA | UUCAAAACUGCGUUGUUUCGCCAU | Cleavage |
| ghr-miR7507 | CotAD_27453 | 4.5 | 1 | 24 | 94 | 116 | AAGGUAGUGAAGUAGGCAAUUGGG | CUCA-UUGUUUAUUUCACCGUUUU | Cleavage |
| ghr-miR7491 | CotAD_27789 | 5 | 1 | 24 | 479 | 502 | UGGGAUCUUCGAGAGGAUUGAGCC | GAAACAAACCUGAAGAAGAUCUUA | Translation |
| ghr-miR2949a-5p | CotAD_31140 | 4.5 | 1 | 22 | 725 | 746 | ACUUUUGAACUGGAUUUGCCGA | ACUGCAAAUUCAGAUUGAAGCU | Translation |
| ghr-miR7510a | CotAD_31140 | 5 | 1 | 24 | 636 | 659 | AAGGUCAUGAUCUUUAGCGGCGUU | AAAGAUGAGAAAGGUCAAGAUCUU | Cleavage |
| ghr-miR2950 | CotAD_31860 | 4.5 | 1 | 21 | 1309 | 1329 | UGGUGUGCAGGGGGUGGAAUA | AGAUCUGUCUCCUUCACGCCA | Cleavage |
| ghr-miR7492a | CotAD_31860 | 5 | 1 | 23 | 21 | 43 | CUAUAGAACAUGAUCUUUAGCGG | CUGUUCAGUAUUAUCUUCUGUGG | Translation |
| ghr-miR7492b | CotAD_31860 | 5 | 1 | 23 | 21 | 43 | CUAUAGAACAUGAUCUUUAGCGG | CUGUUCAGUAUUAUCUUCUGUGG | Translation |
| ghr-miR7492c | CotAD_31860 | 5 | 1 | 23 | 21 | 43 | CUAUAGAACAUGAUCUUUAGCGG | CUGUUCAGUAUUAUCUUCUGUGG | Translation |
| ghr-miR2950 | CotAD_31869 | 4.5 | 1 | 21 | 73 | 93 | UGGUGUGCAGGGGGUGGAAUA | GGUGUCAUCACCUGCACACUU | Cleavage |
| ghr-miR7492a | CotAD_31936 | 4.5 | 1 | 23 | 748 | 770 | CUAUAGAACAUGAUCUUUAGCGG | UUGGUGAAGCCCAAGUUCUAUAA | Translation |
| ghr-miR7492b | CotAD_31936 | 4.5 | 1 | 23 | 748 | 770 | CUAUAGAACAUGAUCUUUAGCGG | UUGGUGAAGCCCAAGUUCUAUAA | Translation |
| ghr-miR7492c | CotAD_31936 | 4.5 | 1 | 23 | 748 | 770 | CUAUAGAACAUGAUCUUUAGCGG | UUGGUGAAGCCCAAGUUCUAUAA | Translation |
| ghr-miR827a | CotAD_31936 | 5 | 1 | 21 | 494 | 514 | UUAGAUGACCAUCAACAAACA | CAUUUUUUGGGGUUCAUGUAA | Translation |
| ghr-miR827b | CotAD_31936 | 5 | 1 | 21 | 494 | 514 | UUAGAUGACCAUCAACAAACA | CAUUUUUUGGGGUUCAUGUAA | Translation |
| ghr-miR827c | CotAD_31936 | 5 | 1 | 21 | 494 | 514 | UUAGAUGACCAUCAACAAACA | CAUUUUUUGGGGUUCAUGUAA | Translation |
| ghr-miR156a | CotAD_32487 | 5 | 1 | 20 | 359 | 378 | UGACAGAAGAGAGUGAGCAC | CCGUUCAGUCUUUUCCGUUA | Cleavage |
| ghr-miR156b | CotAD_32487 | 5 | 1 | 20 | 359 | 378 | UGACAGAAGAGAGUGAGCAC | CCGUUCAGUCUUUUCCGUUA | Cleavage |
| ghr-miR156d | CotAD_32487 | 5 | 1 | 20 | 359 | 378 | UGACAGAAGAGAGUGAGCAC | CCGUUCAGUCUUUUCCGUUA | Cleavage |
| ghr-miR7507 | CotAD_32487 | 5 | 1 | 24 | 93 | 116 | AAGGUAGUGAAGUAGGCAAUUGGG | UCUUAUCGUUUACUUCACCGUUUU | Cleavage |
| ghr-miR399a | CotAD_32645 | 5 | 1 | 21 | 395 | 415 | CGCCAAUGGAGAUUUGUCCGG | GUGGUCCAAUCACUAUUGGAG | Translation |
| ghr-miR399b | CotAD_32645 | 5 | 1 | 21 | 395 | 415 | CGCCAAUGGAGAUUUGUCCGG | GUGGUCCAAUCACUAUUGGAG | Translation |
| ghr-miR2948-5p | CotAD_33143 | 4 | 1 | 22 | 28 | 49 | UGUGGGAGAGUUGGGCAAGAAU | UGGCUUGUCCAACUUGUUUACA | Cleavage |
| ghr-miR482a | CotAD_33143 | 4 | 1 | 22 | 461 | 482 | UCUUUCCUACUCCUCCCAUACC | AACAUGGGAUUAAUAGGGAAGG | Translation |
| ghr-miR7492a | CotAD_33143 | 4 | 1 | 23 | 418 | 440 | CUAUAGAACAUGAUCUUUAGCGG | UCGACAAAGAUCGUGUACUAUAC | Cleavage |
| ghr-miR7492b | CotAD_33143 | 4 | 1 | 23 | 418 | 440 | CUAUAGAACAUGAUCUUUAGCGG | UCGACAAAGAUCGUGUACUAUAC | Cleavage |
| ghr-miR7492c | CotAD_33143 | 4 | 1 | 23 | 418 | 440 | CUAUAGAACAUGAUCUUUAGCGG | UCGACAAAGAUCGUGUACUAUAC | Cleavage |
| ghr-miR7510b | CotAD_33143 | 5 | 1 | 23 | 415 | 437 | AAGAACAUGAUCUUUAGCGGCGU | UUGUCGACAAAGAUCGUGUACUA | Cleavage |
| ghr-miR482a | CotAD_33144 | 4 | 1 | 22 | 986 | 1007 | UCUUUCCUACUCCUCCCAUACC | AACAUGGGAUCAAUAGGGAAGG | Translation |
| ghr-miR827a | CotAD_35069 | 5 | 1 | 21 | 599 | 619 | UUAGAUGACCAUCAACAAACA | CAUUUUUUGGGGUUCAUGUAA | Translation |
| ghr-miR827b | CotAD_35069 | 5 | 1 | 21 | 599 | 619 | UUAGAUGACCAUCAACAAACA | CAUUUUUUGGGGUUCAUGUAA | Translation |
| ghr-miR827c | CotAD_35069 | 5 | 1 | 21 | 599 | 619 | UUAGAUGACCAUCAACAAACA | CAUUUUUUGGGGUUCAUGUAA | Translation |
| ghr-miR399a | CotAD_35514 | 4.5 | 1 | 21 | 467 | 487 | CGCCAAUGGAGAUUUGUCCGG | AAGGACAUAUACCCAUUGGUA | Translation |
| ghr-miR399b | CotAD_35514 | 4.5 | 1 | 21 | 467 | 487 | CGCCAAUGGAGAUUUGUCCGG | AAGGACAUAUACCCAUUGGUA | Translation |
| ghr-miR482b | CotAD_35514 | 5 | 1 | 22 | 478 | 499 | UCUUGCCUACUCCACCCAUGCC | CCCAUUGGUAAAGAUGGCAAGA | Translation |
| ghr-miR3476-3p | CotAD_36446 | 4 | 1 | 21 | 112 | 132 | AGCCAACAACAUCAGUUCUAA | GAAGAACAGAAGGAGUUGGCU | Translation |
| ghr-miR156a | CotAD_39719 | 4.5 | 1 | 20 | 362 | 381 | UGACAGAAGAGAGUGAGCAC | CCUCUUCCUCUCAUCUGUCA | Cleavage |
| ghr-miR156b | CotAD_39719 | 4.5 | 1 | 20 | 362 | 381 | UGACAGAAGAGAGUGAGCAC | CCUCUUCCUCUCAUCUGUCA | Cleavage |
| ghr-miR156d | CotAD_39719 | 4.5 | 1 | 20 | 362 | 381 | UGACAGAAGAGAGUGAGCAC | CCUCUUCCUCUCAUCUGUCA | Cleavage |
| ghr-miR7504a | CotAD_39719 | 4 | 1 | 24 | 690 | 713 | UAUGAAACUGUGAUUCCACGUCAU | GAAAGUUGGGAUCAGGGUUUCGUG | Translation |
| ghr-miR7492a | CotAD_40324 | 4 | 1 | 23 | 814 | 836 | CUAUAGAACAUGAUCUUUAGCGG | CUGGUAAAGACAAAGUUUUAUGG | Translation |
| ghr-miR7492b | CotAD_40324 | 4 | 1 | 23 | 814 | 836 | CUAUAGAACAUGAUCUUUAGCGG | CUGGUAAAGACAAAGUUUUAUGG | Translation |
| ghr-miR7492c | CotAD_40324 | 4 | 1 | 23 | 814 | 836 | CUAUAGAACAUGAUCUUUAGCGG | CUGGUAAAGACAAAGUUUUAUGG | Translation |
| ghr-miR396a | CotAD_41925 | 4.5 | 1 | 21 | 469 | 489 | UUCCACAGCUUUCUUGAACUG | UCGUUCAAUGAAUUUGUGGUA | Translation |
| ghr-miR396b | CotAD_41925 | 4.5 | 1 | 21 | 469 | 489 | UUCCACAGCUUUCUUGAACUG | UCGUUCAAUGAAUUUGUGGUA | Translation |
| ghr-miR7492a | CotAD_41925 | 4.5 | 1 | 23 | 829 | 851 | CUAUAGAACAUGAUCUUUAGCGG | UUGGUGAAGCCCAAGUUCUAUAA | Translation |
| ghr-miR7492b | CotAD_41925 | 4.5 | 1 | 23 | 829 | 851 | CUAUAGAACAUGAUCUUUAGCGG | UUGGUGAAGCCCAAGUUCUAUAA | Translation |
| ghr-miR7492c | CotAD_41925 | 4.5 | 1 | 23 | 829 | 851 | CUAUAGAACAUGAUCUUUAGCGG | UUGGUGAAGCCCAAGUUCUAUAA | Translation |
| ghr-miR827a | CotAD_41925 | 5 | 1 | 21 | 575 | 595 | UUAGAUGACCAUCAACAAACA | CAUUUUUUGGGGUUCAUGUAA | Translation |
| ghr-miR827b | CotAD_41925 | 5 | 1 | 21 | 575 | 595 | UUAGAUGACCAUCAACAAACA | CAUUUUUUGGGGUUCAUGUAA | Translation |
| ghr-miR827c | CotAD_41925 | 5 | 1 | 21 | 575 | 595 | UUAGAUGACCAUCAACAAACA | CAUUUUUUGGGGUUCAUGUAA | Translation |
| ghr-miR7501 | CotAD_43605 | 5 | 1 | 24 | 6 | 29 | AUAUCUGAUUCUGACACGAAAAAA | GUCAACGAAGUCGGAAUCGGAUAU | Cleavage |
| ghr-miR7492a | CotAD_44357 | 5 | 1 | 23 | 814 | 836 | CUAUAGAACAUGAUCUUUAGCGG | CUGGUAAAGACAAAGUGCUAUGG | Translation |
| ghr-miR7492b | CotAD_44357 | 5 | 1 | 23 | 814 | 836 | CUAUAGAACAUGAUCUUUAGCGG | CUGGUAAAGACAAAGUGCUAUGG | Translation |
| ghr-miR7492c | CotAD_44357 | 5 | 1 | 23 | 814 | 836 | CUAUAGAACAUGAUCUUUAGCGG | CUGGUAAAGACAAAGUGCUAUGG | Translation |
| ghr-miR7488 | CotAD_46270 | 3.5 | 1 | 21 | 443 | 463 | UUUUGAGUACAGGGGACAAAA | CGUUGUCCCCCGUACUCGGUA | Translation |
| ghr-miR7510b | CotAD_46270 | 5 | 1 | 23 | 529 | 551 | AAGAACAUGAUCUUUAGCGGCGU | GUGGCGUUGAGGGUAGUGUUGUU | Translation |
| ghr-miR2950 | CotAD_46888 | 5 | 1 | 21 | 122 | 142 | UGGUGUGCAGGGGGUGGAAUA | CUUUCCACCUCAUCUCCAUCA | Translation |
| ghr-miR7510a | CotAD_47322 | 4.5 | 1 | 24 | 417 | 440 | AAGGUCAUGAUCUUUAGCGGCGUU | GACGGCGGUGAAGUUUAAGACCUU | Translation |
| ghr-miR399a | CotAD_48050 | 4.5 | 1 | 21 | 395 | 415 | CGCCAAUGGAGAUUUGUCCGG | GUGGUCCAAUCACCAUUGGAG | Translation |
| ghr-miR399b | CotAD_48050 | 4.5 | 1 | 21 | 395 | 415 | CGCCAAUGGAGAUUUGUCCGG | GUGGUCCAAUCACCAUUGGAG | Translation |
| ghr-miR7511 | CotAD_48469 | 5 | 1 | 24 | 298 | 325 | AGAAGUUUUGCA-UGUGUAGC---UGAG | GUCAAGAGCUUUGCAGUGCACAACUUCU | Cleavage |
| ghr-miR7494 | CotAD_49818 | 5 | 1 | 23 | 542 | 564 | AGCUUGUGGACUAGUUUUAACAA | CCUUUGAUACUGUUACGCAAGUU | Translation |
| ghr-miR7510b | CotAD_51667 | 5 | 1 | 23 | 452 | 474 | AAGAACAUGAUCUUUAGCGGCGU | UCAUUGUUGAUGUUCCUGUUUUU | Translation |
| ghr-miR2950 | CotAD_61391 | 5 | 1 | 21 | 122 | 142 | UGGUGUGCAGGGGGUGGAAUA | CUUUCCACCUCAUCUCCAUCA | Translation |
| ghr-miR7495a | CotAD_62314 | 4 | 1 | 21 | 449 | 469 | UUACUUUAGAUGUCUCCUUCA | UGAAAGAGAAAUCUAAAGAGA | Cleavage |
| ghr-miR7495a | CotAD_62314 | 5 | 1 | 21 | 242 | 262 | UUACUUUAGAUGUCUCCUUCA | CUAAGGAGAAAACGAAAGGAG | Translation |
| ghr-miR7495b | CotAD_62314 | 4 | 1 | 21 | 449 | 469 | UUACUUUAGAUGUCUCCUUCA | UGAAAGAGAAAUCUAAAGAGA | Cleavage |
| ghr-miR7495b | CotAD_62314 | 5 | 1 | 21 | 242 | 262 | UUACUUUAGAUGUCUCCUUCA | CUAAGGAGAAAACGAAAGGAG | Translation |
| ghr-miR7511 | CotAD_62659 | 5 | 1 | 24 | 580 | 603 | AGAAGUUUUGCAUG-UGUAGCUGAG | GACA-CUGUAACAGGUAAAGCUUCU | Cleavage |
| ghr-miR827a | CotAD_62996 | 5 | 1 | 21 | 599 | 619 | UUAGAUGACCAUCAACAAACA | CAUUUUUUGGGGUUCAUGUAA | Translation |
| ghr-miR827b | CotAD_62996 | 5 | 1 | 21 | 599 | 619 | UUAGAUGACCAUCAACAAACA | CAUUUUUUGGGGUUCAUGUAA | Translation |
| ghr-miR827c | CotAD_62996 | 5 | 1 | 21 | 599 | 619 | UUAGAUGACCAUCAACAAACA | CAUUUUUUGGGGUUCAUGUAA | Translation |
| ghr-miR164 | CotAD_63174 | 5 | 1 | 21 | 771 | 791 | UGGAGAAGCAGGGCACGUGCA | CGCACAUGGUUUGCUUAUUCA | Cleavage |
| ghr-miR2949a-5p | CotAD_64004 | 4.5 | 1 | 22 | 616 | 637 | ACUUUUGAACUGGAUUUGCCGA | CAGGCAAUUCAAGAACAGAAGU | Translation |
| ghr-miR7499 | CotAD_64120 | 5 | 1 | 24 | 537 | 559 | AUAUAAUUUUCGGUUAAUUCGGUU | GAUC-AAUAUAUUGAACAUUAUAA | Cleavage |
| ghr-miR7484a | CotAD_64203 | 5 | 1 | 24 | 502 | 529 | UUUGUAUAUUAGAUCAAAGA----GCAA | UUGUAUCAUCUUUAUUUUUAUAUGCGGA | Translation |
| ghr-miR7484b | CotAD_64203 | 5 | 1 | 24 | 502 | 529 | UUUGUAUAUUAGAUCAAAGA----GCAA | UUGUAUCAUCUUUAUUUUUAUAUGCGGA | Translation |
| ghr-miR393 | CotAD_64346 | 5 | 1 | 22 | 371 | 392 | UCCAAAGGGAUCGCAUUGAUCU | ACGGCAAUGCGGUGCCUGUGGC | Translation |
| ghr-miR7513 | CotAD_64347 | 5 | 1 | 21 | 686 | 706 | AAUCAGCCAGGAAUCGUUUGA | CCAAAAGUUGCCUCGUUGGUU | Cleavage |
| ghr-miR7510b | CotAD_64657 | 5 | 1 | 23 | 165 | 187 | AAGAACAUGAUCUUUAGCGGCGU | CCGCCGCUCAUGCUGUUGUUCUU | Translation |
| ghr-miR2950 | CotAD_65119 | 5 | 1 | 21 | 73 | 93 | UGGUGUGCAGGGGGUGGAAUA | GGUGUCAUCAUCUGCACACUC | Cleavage |
| ghr-miR3476-5p | CotAD_65119 | 5 | 1 | 21 | 358 | 378 | UGAACUGGGUUUGUUGGCUGC | GAAGUAAAUAAGAACAGUUCG | Translation |
| ghr-miR2950 | CotAD_65889 | 4.5 | 1 | 21 | 160 | 180 | UGGUGUGCAGGGGGUGGAAUA | CCUUCCACCACUUUCACUUCA | Cleavage |
| ghr-miR2950 | CotAD_66245 | 4.5 | 1 | 21 | 1309 | 1329 | UGGUGUGCAGGGGGUGGAAUA | AGAUCUGUCUCCUUCACGCCA | Cleavage |
| ghr-miR7492a | CotAD_66245 | 5 | 1 | 23 | 21 | 43 | CUAUAGAACAUGAUCUUUAGCGG | CUGUUCAGUAUUAUCUUCUGUGG | Translation |
| ghr-miR7492b | CotAD_66245 | 5 | 1 | 23 | 21 | 43 | CUAUAGAACAUGAUCUUUAGCGG | CUGUUCAGUAUUAUCUUCUGUGG | Translation |
| ghr-miR7492c | CotAD_66245 | 5 | 1 | 23 | 21 | 43 | CUAUAGAACAUGAUCUUUAGCGG | CUGUUCAGUAUUAUCUUCUGUGG | Translation |
| ghr-miR3476-3p | CotAD_66708 | 4 | 1 | 21 | 218 | 238 | AGCCAACAACAUCAGUUCUAA | UAAGAGUCGGUGUUGUUGGUC | Cleavage |
| ghr-miR7489 | CotAD_66708 | 5 | 1 | 24 | 426 | 449 | AUUGUUGCCAAUACAGGAGAACGU | ACAAUCCUCUCUGUUGGUGACAAA | Cleavage |
| ghr-miR7488 | CotAD_67823 | 4.5 | 1 | 21 | 416 | 436 | UUUUGAGUACAGGGGACAAAA | CGUUAUCCCCCGUACUCGGUA | Translation |
| ghr-miR7510b | CotAD_67823 | 5 | 1 | 23 | 502 | 524 | AAGAACAUGAUCUUUAGCGGCGU | GUGGCGUUGAGGGUAGUGUUGUU | Translation |
| ghr-miR7507 | CotAD_68063 | 4.5 | 1 | 24 | 94 | 116 | AAGGUAGUGAAGUAGGCAAUUGGG | CUCA-UUGUUUAUUUCACCGUUUU | Cleavage |
| ghr-miR7496a | CotAD_69738 | 5 | 1 | 24 | 116 | 140 | AUGACCAAAUUGAU-AGAAUGUGUA | UUUACAUUCUUGUUAUUAUGGUCAU | Translation |
| ghr-miR7496b | CotAD_69738 | 5 | 1 | 24 | 116 | 140 | AUGACCAAAUUGAU-AGAAUGUGUA | UUUACAUUCUUGUUAUUAUGGUCAU | Translation |
| ghr-miR2949a-5p | CotAD_70948 | 4.5 | 1 | 22 | 200 | 221 | ACUUUUGAACUGGAUUUGCCGA | GAGGAAAAUUCAGUUUACAAGA | Cleavage |
| ghr-miR2949b | CotAD_70948 | 3.5 | 1 | 22 | 200 | 221 | UCUUUUGAACUGGAUUUGCCGA | GAGGAAAAUUCAGUUUACAAGA | Cleavage |
| ghr-miR2949c | CotAD_70948 | 3.5 | 1 | 22 | 200 | 221 | UCUUUUGAACUGGAUUUGCCGA | GAGGAAAAUUCAGUUUACAAGA | Cleavage |
| ghr-miR7495a | CotAD_70948 | 5 | 1 | 21 | 322 | 342 | UUACUUUAGAUGUCUCCUUCA | UGGAAUACACAUCUAAAGAAA | Cleavage |
| ghr-miR7495b | CotAD_70948 | 5 | 1 | 21 | 322 | 342 | UUACUUUAGAUGUCUCCUUCA | UGGAAUACACAUCUAAAGAAA | Cleavage |
| ghr-miR7510b | CotAD_72913 | 5 | 1 | 23 | 452 | 474 | AAGAACAUGAUCUUUAGCGGCGU | UCAUUGUUGAUGUUCCUGUUUUU | Translation |
| ghr-miR482b | CotAD_74061 | 4.5 | 1 | 22 | 717 | 738 | UCUUGCCUACUCCACCCAUGCC | GGAAGCGGCGGGGAAGGCAAGA | Translation |
| ghr-miR7512 | CotAD_74061 | 5 | 1 | 21 | 953 | 973 | UGCUACUUGUAGUUAUGCAUG | CAUCCGAAACUGCAGAUAGCA | Cleavage |
| ghr-miR2949a-5p | CotAD_75267 | 4.5 | 1 | 22 | 200 | 221 | ACUUUUGAACUGGAUUUGCCGA | GAGGAAAAUUCAGUUUACAAGA | Cleavage |
| ghr-miR2949b | CotAD_75267 | 3.5 | 1 | 22 | 200 | 221 | UCUUUUGAACUGGAUUUGCCGA | GAGGAAAAUUCAGUUUACAAGA | Cleavage |
| ghr-miR2949c | CotAD_75267 | 3.5 | 1 | 22 | 200 | 221 | UCUUUUGAACUGGAUUUGCCGA | GAGGAAAAUUCAGUUUACAAGA | Cleavage |
| ghr-miR7495a | CotAD_75267 | 5 | 1 | 21 | 322 | 342 | UUACUUUAGAUGUCUCCUUCA | UGGAAUACACAUCUAAAGAAA | Cleavage |
| ghr-miR7495b | CotAD_75267 | 5 | 1 | 21 | 322 | 342 | UUACUUUAGAUGUCUCCUUCA | UGGAAUACACAUCUAAAGAAA | Cleavage |
| ghr-miR2950 | CotAD_75537 | 4.5 | 1 | 21 | 160 | 180 | UGGUGUGCAGGGGGUGGAAUA | CCUUCCACCACUUUCACUUCA | Cleavage |

The highlighted regions are the *LEA* genes with high target ratio of more than 5 miRNAs
