# Supplementary material for: Characterization of the late embryogenesis abundant (LEA) proteins family and their role in drought stress tolerance in upland cotton
Source: BMC Genet. 2018 Jan 15;19:6. doi: 10.1186/s12863-017-0596-1 (PMC5769447; doi:10.1186/s12863-017-0596-1)
Supplement: Supplementary file 5 — Gene ontology (GO) terms annotation of LEA genes in upland cotton. (DOCX 66 kb) [file 12863_2017_596_MOESM5_ESM.docx]

Supplementary Table 4: Gene ontology (GO) terms annotation of *LEA* genes in upland cotton, *Gossypium hirsutum*

| Gene ID. | Protein type | GO: Molecular function (MF) | GO: Biological process (BP) | GO: Cellular component (CC) |
| --- | --- | --- | --- | --- |
| CotAD_04417 | DEHYDRIN |  |  |  |
| CotAD_07367 | DEHYDRIN | GO:0016887:ATPase activity | GO:0015692:lead ion transport | GO:0016021:intergral component of membrane |
|  |  | GO:0005524:ATP binding | GO:0080168:abscisic acid transport | GO:0005886:plasma membrane |
|  |  | GO:0000166:nucleotide binding | GO:0055085:transmembrane transport | GO:0016020:membrane |
|  |  | GO:0042626:ATPase activity, coupled to transmembrane movement of substance | GO:0006810:transport |  |
| CotAD_08352 | DEHYDRIN |  | GO:0006950:response to stress | GO:0005829:cytosol |
|  |  |  | GO:0009415:response to water |  |
| CotAD_10502 | DEHYDRIN |  | GO:0006351:transcription,DNA -templated | GO:0005634:nucleus |
|  |  |  | GO:0006355:regulations of transcription, DNA-templated |  |
|  |  |  | GO:0009734:auxin-activated signaling pathway |  |
| CotAD_11398 | DEHYDRIN |  | GO:0006950:response to stress | GO:0016021:intergral component of membrane |
| CotAD_13947 | DEHYDRIN | GO:0008270:zinc ion binding |  |  |
|  |  | GO:0016874:ligase activity |  |  |
| CotAD_15928 | DEHYDRIN |  | GO:0006950:response to stress | GO:0005829:cytosol |
|  |  |  | GO:0009415:response to water |  |
|  |  |  | GO:0009737:response to abscisic acid |  |
|  |  |  | GO:0009414:response to water deprivation |  |
|  |  |  | GO:0009631:cold acclimation |  |
| CotAD_16331 | DEHYDRIN |  | GO:0006364:rRNAprocessing | GO:0032040:small subunit processome |
| CotAD_19173 | DEHYDRIN |  | GO:0009737:response to abscisic acid | GO:0005829:cytosol |
|  |  |  | GO:0009631:cold acclimation |  |
|  |  |  | GO:0006950:response to stress |  |
|  |  |  | GO:0009414:response to water deprivation |  |
| CotAD_27143 | DEHYDRIN |  |  | GO:0016021:intergral component of membrane |
| CotAD_29610 | DEHYDRIN |  | GO:0009269:response to desiccation | GO:0016021:intergral component of membrane |
|  |  |  |  | GO:0005829:cytosol |
| CotAD_35513 | DEHYDRIN |  |  | GO:0016021:intergral component of membrane |
| CotAD_22357 | DEHYDRIN |  |  | GO:0016021:intergralcomponent of membrane |
| CotAD_31255 | DEHYDRIN |  | GO:0006950:response to stress |  |
| CotAD_42408 | DEHYDRIN | GO:0004871:signal transducer activity | GO:0007165:signal transduction | GO:0016021:intergral component of membrane |
|  |  |  | GO:0006952:defense response | GO:0005737:cytoplasm |
|  |  |  |  | GO:0009506:plasmodesma |
|  |  |  |  | GO:0016020:membrane |
|  |  |  |  | GO:0046658:anchored component of plasma membrane |
| CotAD_46550 | DEHYDRIN |  |  | GO:0016021:intergral component of membrane |
|  |  |  |  | GO:0016020:membrane |
| CotAD_50983 | DEHYDRIN | GO:0003677:DNA binding | GO:0006351:transcription,DNA-templated | GO:0005634:nucleus |
|  |  | GO:0003700:transcription factor activity, sequence-specific DNA binding | GO:0006355:regulation of transcription, DNA-templated |  |
|  |  | GO:0046983:protein dimerization activity |  |  |
| CotAD_53264 | DEHYDRIN |  |  | GO:0016021:intergral component of membrane |
|  |  |  |  | GO:0016020:membrane |
| CotAD_57587 | DEHYDRIN |  |  |  |
| CotAD_64203 | DEHYDRIN |  |  |  |
| CotAD_65889 | DEHYDRIN |  |  | GO:0016021:intergral component of membrane |
|  |  |  |  | GO:0016020:membrane |
|  |  |  |  | GO:0005774:vacuolar membrane |
| CotAD_70948 | DEHYDRIN | GO:0003677:DNA binding |  |  |
| CotAD_75267 | DEHYDRIN | GO:0003677:DNA binding |  |  |
| CotAD_75537 | DEHYDRIN |  |  | GO:0016021:intergral component of membrane |
|  |  |  |  | GO:0016020:membrane |
|  |  |  |  | GO:0005774:vacuolar membrane |
| CotAD_16594 | LEA1 |  | GO:0009790:embryo development |  |
| CotAD_16595 | LEA1 |  | GO:0009790:embryo development |  |
| CotAD_17186 | LEA1 |  | GO:0009790:embryo development |  |
| CotAD_20491 | LEA1 |  | GO:0009790:embryo development |  |
| CotAD_30219 | LEA1 |  |  | GO:0016021:intergral component of membrane |
|  |  |  |  | GO:0016020:membrane |
| CotAD_31140 | LEA1 |  |  | GO:0016021:intergral component of membrane |
|  |  |  |  | GO:0016020:membrane |
| CotAD_48976 | LEA1 |  |  | GO:0016021:intergral component of membrane |
|  |  |  |  | GO:0009506:plasmodesma |
|  |  |  |  | GO:0046658:anchored component of plasma membrane |
| CotAD_51667 | LEA1 |  | GO:0009269:response to desiccation | GO:0009506:plasmodesma |
|  |  |  | GO:0009735:response to cytokinin | GO:0005829:cytosol |
|  |  |  |  | GO:0005794:golgi apparatus |
|  |  |  |  | GO:0005886:plasma membrane |
| CotAD_00275 | LEA2 |  |  | GO:0016021:intergral component of membrane |
|  |  |  |  | GO:0005886:plasma membrane |
| CotAD_00465 | LEA2 |  |  | GO:0016021:intergral component of membrane |
| CotAD_00799 | LEA2 | GO:0004871:signal transducer activity | GO:0007165:signal transduction | GO:0016021:intergral component of membrane |
|  |  |  | GO:0006952:defense response | GO:0009506:plasmodesma |
|  |  |  |  | GO:0016020:membrane |
|  |  |  |  | GO:0046658:anchored component of plasma membrane |
| CotAD_00808 | LEA2 | GO:0004871:signal transducer activity | GO:0007165:signal transduction | GO:0016021:intergral component of membrane |
|  |  |  | GO:0006952:defense response | GO:0009506:plasmodesma |
|  |  |  |  | GO:0016020:membrane |
|  |  |  |  | GO:0046658:anchored component of plasma membrane |
| CotAD_01033 | LEA2 |  |  | GO:0016021:intergral component of membrane |
|  |  |  |  | GO:0009507:chloroplast |
|  |  |  |  | GO:0016021::integral component of membrane |
| CotAD_01298 | LEA2 |  |  | GO:0016021:intergral component of membrane |
|  |  |  |  | GO:0005886:plasma membrane |
| CotAD_01321 | LEA2 |  |  | GO:0016021:intergral component of membrane |
|  |  |  |  | GO:0016020:membrane |
| CotAD_01385 | LEA2 |  |  | GO:0016021:intergral component of membrane |
|  |  |  |  | GO:0016020:membrane |
| CotAD_01700 | LEA2 | GO:0003674:molecular_function | GO:0008150:biological_process | GO:0016021:intergral component of membrane |
|  |  |  |  | GO:0009506:plasmodesma |
|  |  |  |  | GO:0016020:membrane |
|  |  |  |  | GO:0005886:plasma membrane |
| CotAD_02652 | LEA2 | GO:0004871:signal transducer activity | GO:0007165:signal transduction | GO:0016021:intergral component of membrane |
|  |  |  | GO:0006952:defense response | GO:0009506:plasmodesma |
|  |  |  |  | GO:0016020:membrane |
|  |  |  |  | GO:0005886:plasma membrane |
|  |  |  |  | GO:0046658:anchored component of plasma membrane |
| CotAD_03037 | LEA2 |  | GO:0009269:response to desiccation | GO:0016021:intergralcomponent of membrane |
|  |  |  |  | GO:0016020:membrane |
|  |  |  |  | GO:0005886:plasma membrane |
| CotAD_03649 | LEA2 |  |  | GO:0016021:intergralcomponent of membrane |
|  |  |  |  | GO:0016020:membrane |
| CotAD_03784 | LEA2 |  |  | GO:0016021:intergralcomponent of membrane |
| CotAD_05724 | LEA2 |  |  | GO:0016021:intergralcomponent of membrane |
|  |  |  |  | GO:0016020:membrane |
| CotAD_05725 | LEA2 |  |  | GO:0016021:intergralcomponent of membrane |
|  |  |  |  | GO:0016020:membrane |
| CotAD_06037 | LEA2 |  |  | GO:0016021:intergral component of membrane |
| CotAD_07087 | LEA2 | GO:0004871:signal transducer activity | GO:0006952:defense response | GO:0009506:plasmodesma |
|  |  |  |  | GO:0016021:intergral component of membrane |
|  |  |  |  | GO:0046658:anchored component of plasma membrane |
| CotAD_08181 | LEA2 |  |  | GO:0016021:intergral component of membrane |
|  |  |  |  | GO:0016020:membrane |
| CotAD_08350 | LEA2 |  |  | GO:0016021:intergralcomponent of membrane |
| CotAD_08837 | LEA2 |  |  | GO:0016021:intergral component of membrane |
|  |  |  |  | GO:0016020:membrane |
| CotAD_09578 | LEA2 | GO:0003674:molecular_function | GO:0008150:biological_process | GO:0016021:intergral component of membrane |
|  |  |  |  | GO:0009506:plasmodesma |
|  |  |  |  | GO:0016020:membrane |
|  |  |  |  | GO:0005886:plasma membrane |
| CotAD_09685 | LEA2 |  |  | GO:0009506:plasmodesma |
|  |  |  |  | GO:0016020:membrane |
|  |  |  |  | GO:0016021:intergral component of membrane |
|  |  |  |  | GO:0005886:plasma membrane |
| CotAD_09732 | LEA2 |  |  | GO:0016021:intergral component of membrane |
|  |  |  |  | GO:0016020:membrane |
| CotAD_10376 | LEA2 |  |  | GO:0016021:intergral component of membrane |
|  |  |  |  | GO:0016020:membrane |
|  |  |  |  | GO:0005886:plasma membrane |
| CotAD_11658 | LEA2 |  |  | GO:0016021:intergral component of membrane |
| CotAD_11875 | LEA2 |  |  | GO:0016021:intergral component of membrane |
| CotAD_11876 | LEA2 | GO:0004871:signal transducer activity | GO:0007165:signal transduction | GO:0016021:intergral component of membrane |
|  |  |  | GO:0051607:defense response to virus | GO:0009506:plasmodesma |
|  |  |  | GO:0006952:defense response | GO:0016020:membrane |
|  |  |  |  | GO:0005886:plasma membrane |
|  |  |  |  | GO:0046658:anchored component of plasma membrane |
| CotAD_11878 | LEA2 |  |  | GO:0016021:intergral component of membrane |
|  |  |  |  | GO:0016020:membrane |
| CotAD_11879 | LEA2 |  |  | GO:0016021:intergral component of membrane |
| CotAD_12375 | LEA2 |  | GO:0050896:response to stimulus | GO:0016021:intergral component of membrane |
|  |  |  | GO:0007165:signal transduction | GO:0009506:plasmodesma |
|  |  |  | GO:0006952:defense response | GO:0005737:cytoplasm |
|  |  |  |  | GO:0016020:membrane |
|  |  |  |  | GO:0046658:achored of plasma membrane |
| CotAD_13115 | LEA2 |  |  | GO:0016021:intergralcomponent of membrane |
|  |  |  |  | GO:0016020:membrane |
| CotAD_13584 | LEA2 |  |  | GO:0016021:intergralcomponent of membrane |
|  |  |  |  | GO:0016020:membrane |
| CotAD_13827 | LEA2 | GO:0016301:kinase activity | GO:0016310:phosphorylation | GO:0016021:intergral component of membrane |
|  |  |  |  | GO:0016020:membrane |
| CotAD_14147 | LEA2 | GO:0004871:signal transducer activity | GO:0007165:signal transduction | GO:0016021:intergral component of membrane |
|  |  |  | GO:0006952:defense response | GO:0009506:plasmodesma |
|  |  |  |  | GO:0016020:membrane |
|  |  |  |  | GO:0005886:plasma membrane |
|  |  |  |  | GO:0046658:anchored component of plasma membrane |
| CotAD_15892 | LEA2 |  |  | GO:0016021:intergral component of membrane |
|  |  |  |  | GO:0016020:membrane |
| CotAD_16731 | LEA2 |  |  | GO:0016021:intergral component of membrane |
|  |  |  |  | GO:0016020:membrane |
| CotAD_17044 | LEA2 |  | GO:0009269:response to desiccation | GO:0016021:intergralcomponent of membrane |
|  |  |  |  | GO:0005829:cytosol |
| CotAD_17045 | LEA2 |  | GO:0009269:response to desiccation | GO:0016021:intergralcomponent of membrane |
|  |  |  |  | GO:0016020:membrane |
|  |  |  |  | GO:0005886:plasma membrane |
| CotAD_17062 | LEA2 | GO:0016874:ligase activity |  | GO:0016021:intergralcomponent of membrane |
|  |  |  |  | GO:0016020:membrane |
| CotAD_17101 | LEA2 | GO:0004871:signal transducer activity | GO:0007165:signal transduction | GO:0016021:intergral component of membrane |
|  |  |  | GO:0006952:defense response | GO:0009506:plasmodesma |
|  |  |  |  | GO:0016020:membrane |
|  |  |  |  | GO:0046658:anchored component of plasma membrane |
| CotAD_17102 | LEA2 | GO:0004871:signal transducer activity | GO:0007165:signal transduction | GO:0009506:plasmodesma |
|  |  |  | GO:0006952:defense response | GO:0046658:anchored component of plasma membrane |
|  |  |  |  | GO:0016021:intergral component of membrane |
|  |  |  |  | GO:0016020:membrane |
| CotAD_17103 | LEA2 |  |  | GO:0016021:intergral component of membrane |
|  |  |  |  | GO:0016020:membrane |
| CotAD_17649 | LEA2 | GO:0016740:transferase activity |  | GO:0016021:intergral component of membrane |
|  |  |  |  | GO:0016020:membrane |
| CotAD_18210 | LEA2 |  |  | GO:0016021:intergral component of membrane |
|  |  |  |  | GO:0016020:membrane |
| CotAD_18233 | LEA2 |  |  | GO:0016021:intergral component of membrane |
|  |  |  |  | GO:0016020:membrane |
| CotAD_18546 | LEA2 |  |  | GO:0016021:intergral component of membrane |
| CotAD_18729 | LEA2 |  |  | GO:0016021:intergral component of membrane |
|  |  |  |  | GO:0016020:membrane |
|  |  |  |  | GO:0005886:plasma membrane |
| CotAD_19078 | LEA2 |  |  | GO:0016021:intergral component of membrane |
| CotAD_19107 | LEA2 |  | GO:0009269:response to desiccation | GO:0016021:intergral component of membrane |
| CotAD_19205 | LEA2 |  |  | GO:0016021:intergral component of membrane |
|  |  |  |  | GO:0016020:membrane |
| CotAD_19213 | LEA2 |  |  | GO:0016021:intergral component of membrane |
| CotAD_19214 | LEA2 |  |  | GO:0016021:intergral component of membrane |
| CotAD_19375 | LEA2 |  |  | GO:0016021:intergralcomponent of membrane |
|  |  |  |  | GO:0016020:membrane |
| CotAD_20020 | LEA2 |  |  | GO:0016021:intergralcomponent of membrane |
|  |  |  |  | GO:0016020:membrane |
| CotAD_20308 | LEA2 |  |  | GO:0016021:intergral component of membrane |
|  |  |  |  | GO:0016020:membrane |
| CotAD_21731 | LEA2 | GO:0016874:ligase activity |  | GO:0016021:intergralcomponent of membrane |
|  |  |  |  | GO:0016020:membrane |
| CotAD_21924 | LEA2 |  |  | GO:0016021:intergral component of membrane |
|  |  |  |  | GO:0016020:membrane |
|  |  |  |  | GO:0009506:plasmodema |
|  |  |  |  | GO:0005886:plasma membrane |
| CotAD_24019 | LEA2 |  | GO:0006950:response to stress |  |
| CotAD_24497 | LEA2 |  |  | GO:0016021:intergralcomponent of membrane |
| CotAD_24499 | LEA2 |  |  | GO:0016021:intergralcomponent of membrane |
|  |  |  |  | GO:0016020:membrane |
| CotAD_25271 | LEA2 |  | GO:0006950:response to stress |  |
|  |  |  | GO:0009415:response to water |  |
| CotAD_26038 | LEA2 | GO:0016740:transferase activity |  | GO:0016021:intergral component of membrane |
|  |  |  |  | GO:0016020:membrane |
| CotAD_27453 | LEA2 |  |  | GO:0016021::integral component of membrane |
| CotAD_27789 | LEA2 |  | GO:0006950:response to stress |  |
|  |  |  | GO:0009415:response to water |  |
| CotAD_28249 | LEA2 |  | GO:0009790:embryo development |  |
| CotAD_28252 | LEA2 |  | GO:0009790:embryo development |  |
| CotAD_28872 | LEA2 |  |  | GO:0016021:intergralcomponent of membrane |
|  |  |  |  | GO:0016020:membrane |
| CotAD_31535 | LEA2 | GO:0004871:signal transducer activity | GO:0007165:signal transduction | GO:0016021:intergralcomponent of membrane |
|  |  |  | GO:0006952:defense response | GO:0009506:plasmodesma |
|  |  |  |  | GO:0016020:membrane |
|  |  |  |  | GO:0046658:anchored component of plasma membrane |
| CotAD_31906 | LEA2 | GO:0003674:molecular_function | GO:0006950:response to stress |  |
| CotAD_31936 | LEA2 |  |  | GO:0016021:intergral component of membrane |
| CotAD_32645 | LEA2 |  |  | GO:0005829:cytosol |
| CotAD_32847 | LEA2 | GO:0004871:signal transducer activity | GO:0007165:signal transduction | GO:0009506:plasmodesma |
|  |  |  | GO:0006952:defense response | GO:0016021:intergral component of membrane |
|  |  |  |  | GO:0016020:membrane |
|  |  |  |  | GO:0046658:anchored component of plasma membrane |
| CotAD_34476 | LEA2 |  |  | GO:0005829:cytosol |
| CotAD_34798 | LEA2 |  |  | GO:0016021:intergral component of membrane |
|  |  |  |  | GO:0016020:membrane |
| CotAD_35069 | LEA2 |  |  | GO:0016021:intergral component of membrane |
|  |  |  |  | GO:0016020:membrane |
| CotAD_35091 | LEA2 |  |  | GO:0016020:membrane |
|  |  |  |  | GO:0016021:intergral component of membrane |
| CotAD_35514 | LEA2 |  |  | GO:0016021:intergral component of membrane |
|  |  |  |  | GO:0016020:membrane |
| CotAD_36328 | LEA2 |  |  | GO:0016021:intergral component of membrane |
| CotAD_36583 | LEA2 |  |  | GO:0016021:intergral component of membrane |
|  |  |  |  | GO:0016020:membrane |
| CotAD_37888 | LEA2 |  |  | GO:0016021:intergralcomponent of membrane |
|  |  |  |  | GO:0016020:membrane |
| CotAD_38978 | LEA2 |  | GO:0006950:reponse to stress |  |
|  |  |  | GO:0009415:response to water |  |
| CotAD_40324 | LEA2 |  |  | GO:0016021:intergralcomponent of membrane |
|  |  |  |  | GO:0016020:membrane |
| CotAD_41925 | LEA2 |  |  | GO:0016021:intergralcomponent of membrane |
|  |  |  |  | GO:0016020:membrane |
| CotAD_42599 | LEA2 |  | GO:0050896:response to stimuli | GO:0016021:intergral component of membrane |
| CotAD_44357 | LEA2 |  |  | GO:0016021:intergralcomponent of membrane |
|  |  |  |  | GO:0016020:membrane |
| CotAD_46873 | LEA2 | GO:0004871:signal transducer activity | GO:0007165:signal transduction | GO:0016021:intergralcomponent of membrane |
|  |  |  | GO:0006952:defense response | GO:0009506:plasmodesma |
|  |  |  |  | GO:0005737:cytoplasm |
|  |  |  |  | GO:0046658:anchored component of plasma membrane |
|  |  |  |  | GO:0005737:cytoplasm |
| CotAD_47495 | LEA2 |  | GO:0006950:response to stress | GO:0005739:mitochondrion |
|  |  |  | GO:0009737:response to abscisic acid |  |
| CotAD_47749 | LEA2 |  |  |  |
| CotAD_48050 | LEA2 |  |  | GO:0005829:cytosol |
| CotAD_48069 | LEA2 |  | GO:0006950:response to stress |  |
| CotAD_48753 | LEA2 |  | GO:0006950:response to stress | GO:0005739:mitochondrion |
|  |  |  | GO:0009737:response to abscisic acid |  |
| CotAD_56356 | LEA2 |  | GO:0009269:response to desiccation | GO:0016021:intergral component of membrane |
|  |  |  |  | GO:0005829:cytosol |
| CotAD_59405 | LEA2 | GO:0004871:signal transducer activity | GO:0007165:signal transduction | GO:0016021:intergral component of membrane |
|  |  |  | GO:0006952:defense response | GO:0009506:plasmodesma |
|  |  |  |  | GO:0016020:membrane |
|  |  |  |  | GO:0046658:anchored component of plasma membrane |
| CotAD_23646 | LEA2 |  |  | GO:0016020:membrane |
|  |  |  |  | GO:0016021:integral component of membrane |
| CotAD_26981 | LEA2 |  |  | GO:0016021:intergral component of membrane |
| CotAD_29279 | LEA2 | GO:0016740:transferase activity | GO:0008150:biogical _ process | GO:0016021:intergral component of membrane |
|  |  | GO:0003674:molecular_function |  | GO:0005634:nucleus |
|  |  |  |  | GO:0016020:membrane |
| CotAD_31344 | LEA2 |  |  | GO:0016021:intergralcomponent of membrane |
|  |  |  |  | GO:0016020:membrane |
| CotAD_31536 | LEA2 | GO:0004871:signal transducer activity | GO:0007165:signal transduction | GO:0016021:intergralcomponent of membrane |
|  |  |  | GO:0006952:defense response | GO:0009506:plasmodesma |
|  |  |  |  | GO:0016020:membrane |
|  |  |  |  | GO:0046658:anchored component of plasma membrane |
| CotAD_31537 | LEA2 |  |  | GO:0016021:intergralcomponent of membrane |
| CotAD_31780 | LEA2 |  | GO:0009737:response to abscisic acid | GO:0005829:cytosol |
|  |  |  | GO:0009631:cold acclimation |  |
|  |  |  | GO:0009414:response to water deprivation |  |
| CotAD_31782 | LEA2 |  |  | GO:0016021:intergralcomponent of membrane |
| CotAD_31860 | LEA2 | GO:0004190:aspartic-type endopeptidase activity | GO:0030163:protein catabolic process | GO:0005576:extracellular region |
|  |  | GO:0016787:hydrolase activity | GO:0006508:proteolysis |  |
|  |  | GO:0008233:peptidase activity |  |  |
| CotAD_32487 | LEA2 |  |  | GO:0016021:intergral component of membrane |
| CotAD_33143 | LEA2 |  |  | GO:0016021:intergral component of membrane |
|  |  |  |  | GO:0016020:membrane |
| CotAD_33144 | LEA2 |  |  | GO:0016021:intergral component of membrane |
|  |  |  |  | GO:0016020:membrane |
| CotAD_36446 | LEA2 |  | GO:0009790:embryo development |  |
| CotAD_37776 | LEA2 |  |  | GO:0016021:intergral component of membrane |
| CotAD_39064 | LEA2 | GO:0004871:signal transducer activity | GO:0007165:signal transduction | GO:0009506:plasmodesma |
|  |  |  | GO:0006952:defense response | GO:0016021:intergral component of membrane |
|  |  |  |  | GO:0046658:anchored component of plasma membrane |
|  |  |  |  | GO:0016020:membrane |
| CotAD_39719 | LEA2 |  |  | GO:0016021:intergral component of membrane |
|  |  |  |  | GO:0016020:membrane |
|  |  |  |  | go:0005886:plasma membrane |
| CotAD_41569 | LEA2 | GO:0004871:signal transducer activity | GO:0006952:defense response | GO:0016021:intergral component of membrane |
|  |  |  |  | GO:0009506:plasmodesma |
|  |  |  |  | GO:0046658:anchored component of plasma membrane |
| CotAD_41571 | LEA2 | GO:0004871:signal transducer activity | GO:0007165:signal transduction | GO:0009506:plasmodesma |
|  |  |  | GO:0006952:defense response | GO:0016020:membrane |
|  |  |  |  | GO:0016021:integral component of membrane |
|  |  |  |  | GO:0046658:anchored component of plasma membrane |
| CotAD_45324 | LEA2 |  |  | GO:0016021:intergral component of membrane |
| CotAD_47322 | LEA2 |  |  | GO:0016021:intergral component of membrane |
| CotAD_47454 | LEA2 |  |  | GO:0016021:intergral component of membrane |
|  |  |  |  | GO:0016020:membrane |
| CotAD_48336 | LEA2 |  |  |  |
| CotAD_48769 | LEA2 |  | GO:0006950:response to stress |  |
|  |  |  | GO:0009415:response to water |  |
| CotAD_49818 | LEA2 |  |  | GO:0016021:integral component of membrane |
|  |  |  |  | GO:0016020:membrane |
| CotAD_53045 | LEA2 |  |  | GO:0005829:cytosol |
| CotAD_53263 | LEA2 |  |  | GO:0009790:embryo development |
| CotAD_53981 | LEA2 |  |  | GO:0009506:plasmodesma |
|  |  |  |  | GO:0016021:integral component of membrane |
|  |  |  |  | GO:0016020:membrane |
|  |  |  |  | GO:0005886:plasma membrane |
| CotAD_54337 | LEA2 |  | GO:0006950:response to stress |  |
|  |  |  | GO:0009415:response to water |  |
| CotAD_55224 | LEA2 |  |  | GO:0016021:intergral component of membrane |
|  |  |  |  | GO:0016020:membrane |
| CotAD_56696 | LEA2 |  |  | GO:0016021:intergral component of membrane |
| CotAD_58358 | LEA2 |  | GO:0006950:response to stress |  |
|  |  |  | GO:0009415:response to water |  |
| CotAD_60279 | LEA2 |  |  | GO:0016021:intergral component of membrane |
|  |  |  |  | GO:0016020:membrane |
| CotAD_60435 | LEA2 |  |  | GO:0016021:intergral component of membrane |
|  |  |  |  | GO:0016020:membrane |
| CotAD_60617 | LEA2 | GO:0004871:signal transducer activity | GO:0007165:signal transduction | GO:0016021:intergral component of membrane |
|  |  |  | GO:0006952:defense response | GO:0009506:plasmodesma |
|  |  |  |  | GO:0005737:cytoplasm |
|  |  |  |  | GO:0016020:membrane |
|  |  |  |  | GO:0046658:anchored component of plasma membrane |
| CotAD_61173 | LEA2 |  |  | GO:0016021:intergral component of membrane |
|  |  |  |  | GO:0016020:membrane |
|  |  |  |  | GO:0005886:plasma membrane |
| CotAD_61391 | LEA2 |  | GO:0009269:response to desiccation |  |
| CotAD_62996 | LEA2 |  |  | GO:0016021:intergral component of membrane |
|  |  |  |  | GO:0016020:membrane |
| CotAD_63174 | LEA2 |  |  | GO:0016021:integral component of membrane |
|  |  |  |  | GO:0016020:membrane |
| CotAD_64004 | LEA2 |  | GO:0009269:response to desiccation | GO:0016021:integral component of membrane |
|  |  |  |  | GO:0016020:membrane |
|  |  |  |  | go:0005886:plasma membrane |
| CotAD_64120 | LEA2 |  |  | GO:0016021:intergralcomponent of membrane |
|  |  |  |  | GO:0016020:membrane |
|  |  |  |  | go:0005886:plasma membrane |
| CotAD_64346 | LEA2 | GO:0004871:signal transducer activity | GO:0007165:signal transduction | GO:0009506:plasmodesma |
|  |  |  | GO:0006952:defense response | GO:0016021:integral component of membrane |
|  |  |  |  | GO:0046658:anchored component of plasma membrane |
| CotAD_64347 | LEA2 |  |  | GO:0009506:plasmodesma |
|  |  |  |  | GO:0016020:membrane |
|  |  |  |  | GO:0016021:integral component of membrane |
| CotAD_64657 | LEA2 |  |  | GO:0016021:intergralcomponent of membrane |
|  |  |  |  | GO:0016020:membrane |
|  |  |  |  | GO:0009506:plasmodesma |
|  |  |  |  | GO:0005886:plasma membrane |
| CotAD_65119 | LEA2 |  |  | GO:0016021:intergral component of membrane |
|  |  |  |  | GO:0016020:membrane |
| CotAD_65370 | LEA2 |  |  | GO:0016021:intergral component of membrane |
|  |  |  |  | GO:0016020:membrane |
| CotAD_66245 | LEA2 | GO:0004190:aspartic-type endopeptidase activity | GO:0030163:protein catabolic process | GO:0005576:extracellular region |
|  |  | GO:0016787:hydrolase activity | GO:0006508:proteolysis |  |
|  |  | GO:0008233:peptidase activity |  |  |
| CotAD_66538 | LEA2 | GO:0004871:signal transducer activity | GO:0007165:signal transduction | GO:0016021:intergral component of membrane |
|  |  |  | GO:0006952:defense response | GO:0009506:plasmodesma |
|  |  |  |  | GO:0016020:membrane |
|  |  |  |  | GO:0046658:anchored component of plasma membrane |
| CotAD_66551 | LEA2 |  |  | GO:0016021:intergral component of membrane |
| CotAD_66774 | LEA2 |  |  | GO:0016021:intergral component of membrane |
|  |  |  |  | GO:0016020:membrane |
| CotAD_66775 | LEA2 | GO:0003674:molecular function | GO:0008150:biological process | GO:0016021:intergral component of membrane |
|  |  |  |  | GO:0016020:membrane |
|  |  |  |  | GO:0005886:plasma membrane |
| CotAD_67823 | LEA2 |  | GO:0007165:signal transduction | GO:0009506:plasmodesma |
|  |  |  | GO:0006952:defense response | GO:0016021:integral component of membrane |
|  |  |  |  | GO:0046658:anchored component of plasma membrane |
| CotAD_68063 | LEA2 |  |  | GO:0016021:intergralcomponent of membrane |
|  |  |  |  | GO:0016020:membrane |
| CotAD_68189 | LEA2 |  |  | GO:0016021:intergralcomponent of membrane |
|  |  |  |  | GO:0009506:plasmodesma |
|  |  |  |  | GO:0016020:membrane |
| CotAD_69737 | LEA2 |  |  | GO:0016021:intergral component of membrane |
| CotAD_69738 | LEA2 |  |  | GO:0016021:intergral component of membrane |
| CotAD_70003 | LEA2 |  |  | GO:0016021:intergral component of membrane |
|  |  |  |  | GO:0016020:membrane |
| CotAD_70190 | LEA2 |  | GO:0042221:response to chemicals | GO:0044464:cell part |
|  |  |  |  |  |
| CotAD_70192 | LEA2 |  | GO:0009269:response to desiccation | GO:0009506:plasmodesma |
|  |  |  | GO:0009735:plasma membrane | GO:0005829:cytosol |
|  |  |  |  | GO:0005794:golgi apparatus |
|  |  |  |  | GO:0005886:plasma membrane |
| CotAD_71431 | LEA2 |  |  | GO:0009506:plasmodesma |
|  |  |  |  | GO:0016021:integral component of membrane |
|  |  |  |  | GO:0046658:anchored component of plasma membrane |
| CotAD_72458 | LEA2 |  |  | GO:0016020:membrane |
|  |  |  |  | GO:0016021:integral component of membrane |
| CotAD_72913 | LEA2 |  | GO:0009269:response to desiccation | GO:0009506:plasmodesma |
|  |  |  | GO:0009735:response to cytokinin | GO:0005829:cytosol |
|  |  |  |  | GO:0005794:golgi apparatus |
|  |  |  |  | GO:0005886:plasma membrane |
| CotAD_73966 | LEA2 |  |  | GO:0016021:intergralcomponent of membrane |
|  |  |  |  | GO:0016020:membrane |
| CotAD_74713 | LEA2 | GO:0004871:signal transducer activity | GO:0007165:signal transduction | GO:0016021:intergralcomponent of membrane |
|  |  |  | GO:0006952:defense response | GO:0009506:plasmodesma |
|  |  |  |  | GO:0016020:membrane |
|  |  |  |  | GO:0005886:plasma membrane |
|  |  |  |  | GO:0046658:anchored component of plasma membrane |
| CotAD_76129 | LEA2 | GO:0004871:signal transducer activity | GO:0007165:signal transduction | GO:0016021:intergralcomponent of membrane |
|  |  |  | GO:0006952:defense response | GO:0009506:plasmodesma |
|  |  |  |  | GO:0016020:membrane |
|  |  |  |  | GO:0046658:anchored component of plasma membrane |
| CotAD_01504 | LEA3 | GO:0006979:response to oxidative stress |  |  |
|  |  | GO:0006950:response to stress |  |  |
| CotAD_04558 | LEA3 |  | GO:0006950:response to stress |  |
| CotAD_04559 | LEA3 |  | GO:0006950:response to stress |  |
| CotAD_21416 | LEA3 |  | GO:0006950:response to stress |  |
| CotAD_22634 | LEA3 |  | GO:0006950:response to stress |  |
| CotAD_23118 | LEA3 |  | GO:0009793:embryo development ending seed dormancy | GO:0005829:cytosol |
| CotAD_24498 | LEA3 | GO:0004871:signal transducer activity | GO:0007165:signal transduction | GO:0016021:intergralcomponent of membrane |
|  |  |  | GO:0006952:defense response | GO:0009506:plasmodesma |
|  |  |  |  | GO:0016020:membrane |
|  |  |  |  | GO:0046658:anchored component of plasma membrane |
| CotAD_33003 | LEA3 |  | GO:0006950:response to stress |  |
| CotAD_36999 | LEA3 |  |  |  |
| CotAD_40972 | LEA3 |  | GO:0009737:response to abscisic acid | GO:0016020:membrane |
|  |  |  | GO:0009631:cold acclimation | GO:0005829:cytosol |
|  |  |  | GO:0009414:response to water deprivation |  |
|  |  |  | GO:0009415:response to water |  |
|  |  |  | GO:0006950:response to stress |  |
| CotAD_26668 | LEA3 |  | GO:0009269:response to desiccation | GO:0016021:intergralcomponent of membrane |
| CotAD_35021 | LEA3 | GO:0003677:trascription factor activity, sequence-specific DNA binding | GO: transcription DNA-templated | GO:0005634:nucleus |
|  |  | GO:0003700:transcription factor activity, sequence-specific DNA binding | GO:0006355:regulation of transcription, DNA-templated |  |
|  |  | GO:0046983:protein dimerization activity |  |  |
| CotAD_41714 | LEA3 |  |  | GO:0016021:intergral component of membrane |
|  |  |  |  | GO:0016020:membrane |
|  |  |  |  | GO:0009506:plasmodesma |
|  |  |  |  | go:0005886:plasma membrane |
| CotAD_43605 | LEA3 |  |  | GO:0016021:intergral component of membrane |
|  |  |  |  | GO:0016020:membrane |
| CotAD_46270 | LEA3 | GO:0004871:signal transducer activity | GO:0007165:signal transduction | GO:0009506:plasmodesma |
|  |  |  | GO:0006952:defense response | GO:0016021:integral component of membrane |
|  |  |  |  | GO:0046658:anchored component of plasma membrane |
| CotAD_02872 | LEA4 |  | GO:0009793:embryo development ending seed dormancy | GO:0005737:cytoplasm |
|  |  |  | GO:0010227:floral organ abscission | GO:0005618:cell wall |
|  |  |  |  | GO:0005829:cytosol |
|  |  |  |  | GO:0005576:extracellular region |
| CotAD_05963 | LEA4 |  |  | GO:0016021:integral component of membrane |
|  |  |  |  | GO:0016020:membrane |
| CotAD_22633 | LEA4 |  | GO:0006950:responsee to stress |  |
| CotAD_23824 | LEA4 | GO:0016740:transferase activity |  | GO:0016021:intergralcomponent of membrane |
|  |  |  |  | GO:0016020:membrane |
| CotAD_50359 | LEA4 | GO:0004871:signal transducer activity | GO:0007165:signal transduction | GO:0016021:intergral component of membrane |
|  |  |  | GO:0006952:defense response | GO:0009506:plasmodesma |
|  |  |  |  | GO:0016020:membrane |
|  |  |  |  | GO:0005886:plasma membrane |
|  |  |  |  | GO:0046658:anchored component of plasma membrane |
| CotAD_62659 | LEA4 |  | GO:0009793:embryo development ending seed dormancy | GO:0005829:cytosol |
|  |  |  | GO:0010227:floral organ abscission |  |
| CotAD_74061 | LEA4 |  | GO:0009793:embryo development ending seed dormancy | GO:0005829:cytosol |
| CotAD_03264 | LEA5 |  |  |  |
| CotAD_07516 | LEA5 | GO:0003700:transcription factor activity, sequence-specific DNA binding | GO:0006355:regulation of transcription, DNA-templated | GO:0005634:nucleus |
| CotAD_46888 | LEA5 |  | GO:0009269:response to desiccation |  |
| CotAD_31869 | LEA5 |  |  | GO:0016021:intergral component of membrane |
|  |  |  |  | GO:0016020:membrane |
| CotAD_33321 | LEA5 | GO:0004871:signal transducer activity | GO:0007165:signal transduction | GO:0016021:intergral component of membrane |
|  |  |  | GO:0006952:defense response | GO:0009506:plasmodesma |
|  |  |  |  | GO:0016020:membrane |
|  |  |  |  | GO:0046658:anchored component of plasma membrane |
| CotAD_48469 | LEA5 |  |  | GO:0016021:intergral component of membrane |
| CotAD_56699 | LEA5 |  |  | GO:0016021:intergral component of membrane |
| CotAD_57519 | LEA5 |  | GO:0006950:response to stress |  |
| CotAD_13789 | LEA6 |  |  | GO:0005829:cytosol |
| CotAD_53438 | LEA6 |  |  | GO:0016021:intergral component of membrane |
|  |  |  |  | GO:0009506:plasmodesma |
|  |  |  |  | GO:0016020:membrane |
|  |  |  |  | GO:0046658:anchored component of plasma membrane |
|  |  |  |  | GO:0005886:plasma membrane |
| CotAD_44941 | LEA6 |  |  | GO:0016021:intergral component of membrane |
|  |  |  |  | GO:0016020:membrane |
| CotAD_12680 | SMP |  |  |  |
| CotAD_12681 | SMP |  | GO:0009737:reponse to abscisic acid | GO:0005829:cytosol |
|  |  |  | GO:0006970:response to osmotic stress | GO:0005730:nucleus |
|  |  |  | GO:0010226:response to lithium ion |  |
|  |  |  | GO:0009845:seed germination |  |
|  |  |  | GO:0006873:cellular ion homeostasis |  |
|  |  |  | GO:0009414:response to water deprivation |  |
| CotAD_39233 | SMP |  | GO:0006950:response to stress |  |
| CotAD_45390 | SMP |  | GO:0006950:response to stress |  |
| CotAD_43455 | SMP |  |  | GO:0005634:nucleus |
| CotAD_51205 | SMP |  |  | GO:0016021:intergral component of membrane |
| CotAD_66708 | SMP | GO:0003674:molecular_function | GO:0009737:response to abscisic acid | GO:0005634:nucleus |
|  |  |  | GO:0009793:embryo development ending seed dormancy | GO:0005737:cytoplasm |
|  |  |  | GO:0010226:response to lithium ion | GO:0005730:nucleolus |
|  |  |  | GO:0006970:response to osmotic stress |  |
|  |  |  | GO:0009845:seed germination |  |
|  |  |  | GO:0006873:cellular ion homeostasis |  |
|  |  |  | GO:0009414:response to water deprivation |  |
|  |  |  |  |  |
