# Supplementary material for: Characterization of the late embryogenesis abundant (LEA) proteins family and their role in drought stress tolerance in upland cotton
Source: BMC Genet. 2018 Jan 15;19:6. doi: 10.1186/s12863-017-0596-1 (PMC5769447; doi:10.1186/s12863-017-0596-1)
Supplement: Supplementary file 6 — Cis element analysis of putative LEA promoters related to drought stress. (DOCX 719 kb) [file 12863_2017_596_MOESM6_ESM.docx]

Supplementary table 5: CIS element analysis of putative LEA promoters related to drought stress

| LEA type | Factor or Site Name | Location | strand | Signal Sequence | SITE | Function |
| --- | --- | --- | --- | --- | --- | --- |
| DEHYDRIN | ABRECE1HVA22 | 74 | (-) | TGCCACCGG | S000014 | ABA responsive elements |
| DEHYDRIN | ABRELATERD1 | 189 | (+) | ACGTG | S000414 | ABA responsive elements |
| DEHYDRIN | ABRELATERD1 | 180 | (+) | ACGTG | S000414 | ABA responsive elements |
| DEHYDRIN | ABRELATERD1 | 720 | (-) | ACGTG | S000414 | ABA responsive elements |
| DEHYDRIN | ABRELATERD1 | 222 | (+) | ACGTG | S000414 | ABA responsive elements |
| DEHYDRIN | ABRELATERD1 | 717 | (-) | ACGTG | S000414 | ABA responsive elements |
| DEHYDRIN | ABRELATERD1 | 95 | (-) | ACGTG | S000414 | ABA responsive elements |
| DEHYDRIN | ABRELATERD1 | 96 | (+) | ACGTG | S000414 | ABA responsive elements |
| DEHYDRIN | ABRELATERD1 | 381 | (+) | ACGTG | S000414 | ABA responsive elements |
| DEHYDRIN | ABRELATERD1 | 363 | (-) | ACGTG | S000414 | ABA responsive elements |
| DEHYDRIN | ABRELATERD1 | 364 | (+) | ACGTG | S000414 | ABA responsive elements |
| DEHYDRIN | ABRELATERD1 | 896 | (+) | ACGTG | S000414 | ABA responsive elements |
| DEHYDRIN | ABRELATERD1 | 400 | (-) | ACGTG | S000414 | ABA responsive elements |
| DEHYDRIN | ABRELATERD1 | 401 | (+) | ACGTG | S000414 | ABA responsive elements |
| DEHYDRIN | ABRELATERD1 | 421 | (-) | ACGTG | S000414 | ABA responsive elements |
| DEHYDRIN | ABRELATERD1 | 422 | (+) | ACGTG | S000414 | ABA responsive elements |
| DEHYDRIN | ABRELATERD1 | 74 | (-) | ACGTG | S000414 | ABA responsive elements |
| DEHYDRIN | ABRELATERD1 | 95 | (-) | ACGTG | S000414 | ABA responsive elements |
| DEHYDRIN | ABRELATERD1 | 96 | (+) | ACGTG | S000414 | ABA responsive elements |
| DEHYDRIN | ABRELATERD1 | 381 | (+) | ACGTG | S000414 | ABA responsive elements |
| DEHYDRIN | ABRELATERD1 | 863 | (+) | ACGTG | S000414 | ABA responsive elements |
| DEHYDRIN | ABRELATERD1 | 458 | (+) | ACGTG | S000414 | ABA responsive elements |
| DEHYDRIN | ABRELATERD1 | 581 | (+) | ACGTG | S000414 | ABA responsive elements |
| DEHYDRIN | ABRELATERD1 | 586 | (-) | ACGTG | S000414 | ABA responsive elements |
| DEHYDRIN | ABRELATERD1 | 86 | (+) | ACGTG | S000414 | ABA responsive elements |
| DEHYDRIN | ABRELATERD1 | 16 | (+) | ACGTG | S000414 | ABA responsive elements |
| DEHYDRIN | ABRELATERD1 | 146 | (-) | ACGTG | S000414 | ABA responsive elements |
| DEHYDRIN | ABRELATERD1 | 241 | (+) | ACGTG | S000414 | ABA responsive elements |
| DEHYDRIN | ABREOSRAB21 | 189 | (+) | ACGTSSSC | S000012 | ABA responsive elements |
| DEHYDRIN | ABREOSRAB21 | 180 | (+) | ACGTSSSC | S000012 | ABA responsive elements |
| DEHYDRIN | ABREOSRAB21 | 376 | (-) | ACGTSSSC | S000012 | ABA responsive elements |
| DEHYDRIN | ABREOSRAB21 | 454 | (-) | ACGTSSSC | S000012 | ABA responsive elements |
| DEHYDRIN | ABREOSRAB21 | 577 | (-) | ACGTSSSC | S000012 | ABA responsive elements |
| DEHYDRIN | ABRERATCAL | 719 | (-) | MACGYGB | S000507 | ABA responsive elements |
| DEHYDRIN | ABRERATCAL | 221 | (+) | MACGYGB | S000507 | ABA responsive elements |
| DEHYDRIN | ABRERATCAL | 716 | (-) | MACGYGB | S000507 | ABA responsive elements |
| DEHYDRIN | ABRERATCAL | 363 | (+) | MACGYGB | S000507 | ABA responsive elements |
| DEHYDRIN | ABRERATCAL | 399 | (-) | MACGYGB | S000507 | ABA responsive elements |
| DEHYDRIN | ABRERATCAL | 237 | (-) | MACGYGB | S000507 | ABA responsive elements |
| DEHYDRIN | ABRERATCAL | 306 | (-) | MACGYGB | S000507 | ABA responsive elements |
| DEHYDRIN | ABRERATCAL | 328 | (+) | MACGYGB | S000507 | ABA responsive elements |
| DEHYDRIN | ABRERATCAL | 585 | (-) | MACGYGB | S000507 | ABA responsive elements |
| DEHYDRIN | ACGTABREMOTIFA2OSEM | 398 | (-) | ACGTGKC | S000394 | early responsive to dehydration |
| DEHYDRIN | ACGTABREMOTIFA2OSEM | 581 | (+) | ACGTGKC | S000394 | early responsive to dehydration |
| DEHYDRIN | ACGTATERD1 | 189 | (-) | ACGT | S000415 | early responsive to dehydration |
| DEHYDRIN | ACGTATERD1 | 189 | (+) | ACGT | S000415 | early responsive to dehydration |
| DEHYDRIN | ACGTATERD1 | 180 | (-) | ACGT | S000415 | early responsive to dehydration |
| DEHYDRIN | ACGTATERD1 | 180 | (+) | ACGT | S000415 | early responsive to dehydration |
| DEHYDRIN | ACGTATERD1 | 551 | (-) | ACGT | S000415 | early responsive to dehydration |
| DEHYDRIN | ACGTATERD1 | 551 | (+) | ACGT | S000415 | early responsive to dehydration |
| DEHYDRIN | ACGTATERD1 | 272 | (-) | ACGT | S000415 | early responsive to dehydration |
| DEHYDRIN | ACGTATERD1 | 272 | (+) | ACGT | S000415 | early responsive to dehydration |
| DEHYDRIN | ACGTATERD1 | 542 | (-) | ACGT | S000415 | early responsive to dehydration |
| DEHYDRIN | ACGTATERD1 | 542 | (+) | ACGT | S000415 | early responsive to dehydration |
| DEHYDRIN | ACGTATERD1 | 688 | (-) | ACGT | S000415 | early responsive to dehydration |
| DEHYDRIN | ACGTATERD1 | 688 | (+) | ACGT | S000415 | early responsive to dehydration |
| DEHYDRIN | ACGTATERD1 | 694 | (-) | ACGT | S000415 | early responsive to dehydration |
| DEHYDRIN | ACGTATERD1 | 694 | (+) | ACGT | S000415 | early responsive to dehydration |
| DEHYDRIN | ACGTATERD1 | 721 | (-) | ACGT | S000415 | early responsive to dehydration |
| DEHYDRIN | ACGTATERD1 | 721 | (+) | ACGT | S000415 | early responsive to dehydration |
| DEHYDRIN | ACGTATERD1 | 542 | (-) | ACGT | S000415 | early responsive to dehydration |
| DEHYDRIN | ACGTATERD1 | 542 | (+) | ACGT | S000415 | early responsive to dehydration |
| DEHYDRIN | ACGTATERD1 | 833 | (-) | ACGT | S000415 | early responsive to dehydration |
| DEHYDRIN | ACGTATERD1 | 833 | (+) | ACGT | S000415 | early responsive to dehydration |
| DEHYDRIN | ACGTATERD1 | 222 | (-) | ACGT | S000415 | early responsive to dehydration |
| DEHYDRIN | ACGTATERD1 | 222 | (+) | ACGT | S000415 | early responsive to dehydration |
| DEHYDRIN | ACGTATERD1 | 44 | (-) | ACGT | S000415 | early responsive to dehydration |
| DEHYDRIN | ACGTATERD1 | 44 | (+) | ACGT | S000415 | early responsive to dehydration |
| DEHYDRIN | ACGTATERD1 | 578 | (-) | ACGT | S000415 | early responsive to dehydration |
| DEHYDRIN | ACGTATERD1 | 578 | (+) | ACGT | S000415 | early responsive to dehydration |
| DEHYDRIN | ACGTATERD1 | 685 | (-) | ACGT | S000415 | early responsive to dehydration |
| DEHYDRIN | ACGTATERD1 | 685 | (+) | ACGT | S000415 | early responsive to dehydration |
| DEHYDRIN | ACGTATERD1 | 691 | (-) | ACGT | S000415 | early responsive to dehydration |
| DEHYDRIN | ACGTATERD1 | 691 | (+) | ACGT | S000415 | early responsive to dehydration |
| DEHYDRIN | ACGTATERD1 | 718 | (-) | ACGT | S000415 | early responsive to dehydration |
| DEHYDRIN | ACGTATERD1 | 718 | (+) | ACGT | S000415 | early responsive to dehydration |
| DEHYDRIN | ACGTATERD1 | 227 | (-) | ACGT | S000415 | early responsive to dehydration |
| DEHYDRIN | ACGTATERD1 | 227 | (+) | ACGT | S000415 | early responsive to dehydration |
| DEHYDRIN | ACGTATERD1 | 290 | (-) | ACGT | S000415 | early responsive to dehydration |
| DEHYDRIN | ACGTATERD1 | 290 | (+) | ACGT | S000415 | early responsive to dehydration |
| DEHYDRIN | ACGTATERD1 | 332 | (-) | ACGT | S000415 | early responsive to dehydration |
| DEHYDRIN | ACGTATERD1 | 332 | (+) | ACGT | S000415 | early responsive to dehydration |
| DEHYDRIN | ACGTATERD1 | 185 | (-) | ACGT | S000415 | early responsive to dehydration |
| DEHYDRIN | ACGTATERD1 | 185 | (+) | ACGT | S000415 | early responsive to dehydration |
| DEHYDRIN | ACGTATERD1 | 191 | (-) | ACGT | S000415 | early responsive to dehydration |
| DEHYDRIN | ACGTATERD1 | 191 | (+) | ACGT | S000415 | early responsive to dehydration |
| DEHYDRIN | ACGTATERD1 | 197 | (-) | ACGT | S000415 | early responsive to dehydration |
| DEHYDRIN | ACGTATERD1 | 197 | (+) | ACGT | S000415 | early responsive to dehydration |
| DEHYDRIN | ACGTATERD1 | 293 | (-) | ACGT | S000415 | early responsive to dehydration |
| DEHYDRIN | ACGTATERD1 | 293 | (+) | ACGT | S000415 | early responsive to dehydration |
| DEHYDRIN | ACGTATERD1 | 502 | (-) | ACGT | S000415 | early responsive to dehydration |
| DEHYDRIN | ACGTATERD1 | 502 | (+) | ACGT | S000415 | early responsive to dehydration |
| DEHYDRIN | ACGTATERD1 | 80 | (-) | ACGT | S000415 | early responsive to dehydration |
| DEHYDRIN | ACGTATERD1 | 80 | (+) | ACGT | S000415 | early responsive to dehydration |
| DEHYDRIN | ACGTATERD1 | 96 | (-) | ACGT | S000415 | early responsive to dehydration |
| DEHYDRIN | ACGTATERD1 | 96 | (+) | ACGT | S000415 | early responsive to dehydration |
| DEHYDRIN | ACGTATERD1 | 381 | (-) | ACGT | S000415 | early responsive to dehydration |
| DEHYDRIN | ACGTATERD1 | 381 | (+) | ACGT | S000415 | early responsive to dehydration |
| DEHYDRIN | ACGTATERD1 | 364 | (-) | ACGT | S000415 | early responsive to dehydration |
| DEHYDRIN | ACGTATERD1 | 364 | (+) | ACGT | S000415 | early responsive to dehydration |
| DEHYDRIN | ACGTATERD1 | 886 | (-) | ACGT | S000415 | early responsive to dehydration |
| DEHYDRIN | ACGTATERD1 | 886 | (+) | ACGT | S000415 | early responsive to dehydration |
| DEHYDRIN | ACGTATERD1 | 896 | (-) | ACGT | S000415 | early responsive to dehydration |
| DEHYDRIN | ACGTATERD1 | 896 | (+) | ACGT | S000415 | early responsive to dehydration |
| DEHYDRIN | ACGTATERD1 | 101 | (-) | ACGT | S000415 | early responsive to dehydration |
| DEHYDRIN | ACGTATERD1 | 101 | (+) | ACGT | S000415 | early responsive to dehydration |
| DEHYDRIN | ACGTATERD1 | 230 | (-) | ACGT | S000415 | early responsive to dehydration |
| DEHYDRIN | ACGTATERD1 | 230 | (+) | ACGT | S000415 | early responsive to dehydration |
| DEHYDRIN | ACGTATERD1 | 313 | (-) | ACGT | S000415 | early responsive to dehydration |
| DEHYDRIN | ACGTATERD1 | 313 | (+) | ACGT | S000415 | early responsive to dehydration |
| DEHYDRIN | ACGTATERD1 | 335 | (-) | ACGT | S000415 | early responsive to dehydration |
| DEHYDRIN | ACGTATERD1 | 335 | (+) | ACGT | S000415 | early responsive to dehydration |
| DEHYDRIN | ACGTATERD1 | 365 | (-) | ACGT | S000415 | early responsive to dehydration |
| DEHYDRIN | ACGTATERD1 | 365 | (+) | ACGT | S000415 | early responsive to dehydration |
| DEHYDRIN | ACGTATERD1 | 401 | (-) | ACGT | S000415 | early responsive to dehydration |
| DEHYDRIN | ACGTATERD1 | 401 | (+) | ACGT | S000415 | early responsive to dehydration |
| DEHYDRIN | ACGTATERD1 | 406 | (-) | ACGT | S000415 | early responsive to dehydration |
| DEHYDRIN | ACGTATERD1 | 406 | (+) | ACGT | S000415 | early responsive to dehydration |
| DEHYDRIN | ACGTATERD1 | 422 | (-) | ACGT | S000415 | early responsive to dehydration |
| DEHYDRIN | ACGTATERD1 | 422 | (+) | ACGT | S000415 | early responsive to dehydration |
| DEHYDRIN | ACGTATERD1 | 491 | (-) | ACGT | S000415 | early responsive to dehydration |
| DEHYDRIN | ACGTATERD1 | 491 | (+) | ACGT | S000415 | early responsive to dehydration |
| DEHYDRIN | ACGTATERD1 | 75 | (-) | ACGT | S000415 | early responsive to dehydration |
| DEHYDRIN | ACGTATERD1 | 75 | (+) | ACGT | S000415 | early responsive to dehydration |
| DEHYDRIN | ACGTATERD1 | 80 | (-) | ACGT | S000415 | early responsive to dehydration |
| DEHYDRIN | ACGTATERD1 | 80 | (+) | ACGT | S000415 | early responsive to dehydration |
| DEHYDRIN | ACGTATERD1 | 96 | (-) | ACGT | S000415 | early responsive to dehydration |
| DEHYDRIN | ACGTATERD1 | 96 | (+) | ACGT | S000415 | early responsive to dehydration |
| DEHYDRIN | ACGTATERD1 | 381 | (-) | ACGT | S000415 | early responsive to dehydration |
| DEHYDRIN | ACGTATERD1 | 381 | (+) | ACGT | S000415 | early responsive to dehydration |
| DEHYDRIN | ACGTATERD1 | 863 | (-) | ACGT | S000415 | early responsive to dehydration |
| DEHYDRIN | ACGTATERD1 | 863 | (+) | ACGT | S000415 | early responsive to dehydration |
| DEHYDRIN | ACGTATERD1 | 310 | (-) | ACGT | S000415 | early responsive to dehydration |
| DEHYDRIN | ACGTATERD1 | 310 | (+) | ACGT | S000415 | early responsive to dehydration |
| DEHYDRIN | ACGTATERD1 | 344 | (-) | ACGT | S000415 | early responsive to dehydration |
| DEHYDRIN | ACGTATERD1 | 344 | (+) | ACGT | S000415 | early responsive to dehydration |
| DEHYDRIN | ACGTATERD1 | 449 | (-) | ACGT | S000415 | early responsive to dehydration |
| DEHYDRIN | ACGTATERD1 | 449 | (+) | ACGT | S000415 | early responsive to dehydration |
| DEHYDRIN | ACGTATERD1 | 44 | (-) | ACGT | S000415 | early responsive to dehydration |
| DEHYDRIN | ACGTATERD1 | 44 | (+) | ACGT | S000415 | early responsive to dehydration |
| DEHYDRIN | ACGTATERD1 | 98 | (-) | ACGT | S000415 | early responsive to dehydration |
| DEHYDRIN | ACGTATERD1 | 98 | (+) | ACGT | S000415 | early responsive to dehydration |
| DEHYDRIN | ACGTATERD1 | 150 | (-) | ACGT | S000415 | early responsive to dehydration |
| DEHYDRIN | ACGTATERD1 | 150 | (+) | ACGT | S000415 | early responsive to dehydration |
| DEHYDRIN | ACGTATERD1 | 380 | (-) | ACGT | S000415 | early responsive to dehydration |
| DEHYDRIN | ACGTATERD1 | 380 | (+) | ACGT | S000415 | early responsive to dehydration |
| DEHYDRIN | ACGTATERD1 | 458 | (-) | ACGT | S000415 | early responsive to dehydration |
| DEHYDRIN | ACGTATERD1 | 458 | (+) | ACGT | S000415 | early responsive to dehydration |
| DEHYDRIN | ACGTATERD1 | 466 | (-) | ACGT | S000415 | early responsive to dehydration |
| DEHYDRIN | ACGTATERD1 | 466 | (+) | ACGT | S000415 | early responsive to dehydration |
| DEHYDRIN | ACGTATERD1 | 563 | (-) | ACGT | S000415 | early responsive to dehydration |
| DEHYDRIN | ACGTATERD1 | 563 | (+) | ACGT | S000415 | early responsive to dehydration |
| DEHYDRIN | ACGTATERD1 | 581 | (-) | ACGT | S000415 | early responsive to dehydration |
| DEHYDRIN | ACGTATERD1 | 581 | (+) | ACGT | S000415 | early responsive to dehydration |
| DEHYDRIN | ACGTATERD1 | 644 | (-) | ACGT | S000415 | early responsive to dehydration |
| DEHYDRIN | ACGTATERD1 | 644 | (+) | ACGT | S000415 | early responsive to dehydration |
| DEHYDRIN | ACGTATERD1 | 167 | (-) | ACGT | S000415 | early responsive to dehydration |
| DEHYDRIN | ACGTATERD1 | 167 | (+) | ACGT | S000415 | early responsive to dehydration |
| DEHYDRIN | ACGTATERD1 | 472 | (-) | ACGT | S000415 | early responsive to dehydration |
| DEHYDRIN | ACGTATERD1 | 472 | (+) | ACGT | S000415 | early responsive to dehydration |
| DEHYDRIN | ACGTATERD1 | 587 | (-) | ACGT | S000415 | early responsive to dehydration |
| DEHYDRIN | ACGTATERD1 | 587 | (+) | ACGT | S000415 | early responsive to dehydration |
| DEHYDRIN | ACGTATERD1 | 134 | (-) | ACGT | S000415 | early responsive to dehydration |
| DEHYDRIN | ACGTATERD1 | 134 | (+) | ACGT | S000415 | early responsive to dehydration |
| DEHYDRIN | ACGTATERD1 | 195 | (-) | ACGT | S000415 | early responsive to dehydration |
| DEHYDRIN | ACGTATERD1 | 195 | (+) | ACGT | S000415 | early responsive to dehydration |
| DEHYDRIN | ACGTATERD1 | 251 | (-) | ACGT | S000415 | early responsive to dehydration |
| DEHYDRIN | ACGTATERD1 | 251 | (+) | ACGT | S000415 | early responsive to dehydration |
| DEHYDRIN | ACGTATERD1 | 265 | (-) | ACGT | S000415 | early responsive to dehydration |
| DEHYDRIN | ACGTATERD1 | 265 | (+) | ACGT | S000415 | early responsive to dehydration |
| DEHYDRIN | ACGTATERD1 | 587 | (-) | ACGT | S000415 | early responsive to dehydration |
| DEHYDRIN | ACGTATERD1 | 587 | (+) | ACGT | S000415 | early responsive to dehydration |
| DEHYDRIN | ACGTATERD1 | 86 | (-) | ACGT | S000415 | early responsive to dehydration |
| DEHYDRIN | ACGTATERD1 | 86 | (+) | ACGT | S000415 | early responsive to dehydration |
| DEHYDRIN | ACGTATERD1 | 833 | (-) | ACGT | S000415 | early responsive to dehydration |
| DEHYDRIN | ACGTATERD1 | 833 | (+) | ACGT | S000415 | early responsive to dehydration |
| DEHYDRIN | ACGTATERD1 | 16 | (-) | ACGT | S000415 | early responsive to dehydration |
| DEHYDRIN | ACGTATERD1 | 16 | (+) | ACGT | S000415 | early responsive to dehydration |
| DEHYDRIN | ACGTATERD1 | 9 | (-) | ACGT | S000415 | early responsive to dehydration |
| DEHYDRIN | ACGTATERD1 | 9 | (+) | ACGT | S000415 | early responsive to dehydration |
| DEHYDRIN | ACGTATERD1 | 147 | (-) | ACGT | S000415 | early responsive to dehydration |
| DEHYDRIN | ACGTATERD1 | 147 | (+) | ACGT | S000415 | early responsive to dehydration |
| DEHYDRIN | ACGTATERD1 | 241 | (-) | ACGT | S000415 | early responsive to dehydration |
| DEHYDRIN | ACGTATERD1 | 241 | (+) | ACGT | S000415 | early responsive to dehydration |
| DEHYDRIN | AGCBOXNPGLB | 201 | (+) | AGCCGCC | S000232 | early responsive to dehydration |
| DEHYDRIN | AGCBOXNPGLB | 273 | (-) | AGCCGCC | S000232 | early responsive to dehydration |
| DEHYDRIN | AGCBOXNPGLB | 515 | (-) | AGCCGCC | S000232 | early responsive to dehydration |
| DEHYDRIN | LTRE1HVBLT49 | 312 | (-) | CCGAAA | S000250 | necessary for coldor drought |
| DEHYDRIN | LTRE1HVBLT49 | 303 | (-) | CCGAAA | S000250 | necessary for coldor drought |
| DEHYDRIN | LTRE1HVBLT49 | 210 | (+) | CCGAAA | S000250 | necessary for coldor drought |
| DEHYDRIN | LTRE1HVBLT49 | 203 | (+) | CCGAAA | S000250 | necessary for coldor drought |
| DEHYDRIN | LTRE1HVBLT49 | 765 | (-) | CCGAAA | S000250 | necessary for coldor drought |
| DEHYDRIN | LTRE1HVBLT49 | 380 | (-) | CCGAAA | S000250 | necessary for coldor drought |
| DEHYDRIN | LTRE1HVBLT49 | 12 | (-) | CCGAAA | S000250 | necessary for coldor drought |
| DEHYDRIN | LTRE1HVBLT49 | 119 | (-) | CCGAAA | S000250 | necessary for coldor drought |
| DEHYDRIN | LTRE1HVBLT49 | 234 | (-) | CCGAAA | S000250 | necessary for coldor drought |
| DEHYDRIN | LTRE1HVBLT49 | 66 | (-) | CCGAAA | S000250 | necessary for coldor drought |
| DEHYDRIN | LTRE1HVBLT49 | 311 | (-) | CCGAAA | S000250 | necessary for coldor drought |
| DEHYDRIN | LTRE1HVBLT49 | 548 | (-) | CCGAAA | S000250 | necessary for coldor drought |
| DEHYDRIN | LTRE1HVBLT49 | 366 | (-) | CCGAAA | S000250 | necessary for coldor drought |
| DEHYDRIN | LTRE1HVBLT49 | 765 | (-) | CCGAAA | S000250 | necessary for coldor drought |
| DEHYDRIN | LTREATLTI78 | 706 | (+) | ACCGACA | S000157 | necessary for coldor drought |
| DEHYDRIN | LTREATLTI78 | 553 | (-) | ACCGACA | S000157 | necessary for coldor drought |
| DEHYDRIN | LTRECOREATCOR15 | 588 | (-) | CCGAC | S000153 | necessary for coldor drought |
| DEHYDRIN | LTRECOREATCOR15 | 32 | (+) | CCGAC | S000153 | necessary for coldor drought |
| DEHYDRIN | LTRECOREATCOR15 | 223 | (-) | CCGAC | S000153 | necessary for coldor drought |
| DEHYDRIN | LTRECOREATCOR15 | 391 | (-) | CCGAC | S000153 | necessary for coldor drought |
| DEHYDRIN | LTRECOREATCOR15 | 707 | (+) | CCGAC | S000153 | necessary for coldor drought |
| DEHYDRIN | LTRECOREATCOR15 | 267 | (-) | CCGAC | S000153 | necessary for coldor drought |
| DEHYDRIN | LTRECOREATCOR15 | 685 | (+) | CCGAC | S000153 | necessary for coldor drought |
| DEHYDRIN | LTRECOREATCOR15 | 210 | (-) | CCGAC | S000153 | necessary for coldor drought |
| DEHYDRIN | LTRECOREATCOR15 | 211 | (-) | CCGAC | S000153 | necessary for coldor drought |
| DEHYDRIN | LTRECOREATCOR15 | 453 | (-) | CCGAC | S000153 | necessary for coldor drought |
| DEHYDRIN | LTRECOREATCOR15 | 598 | (-) | CCGAC | S000153 | necessary for coldor drought |
| DEHYDRIN | LTRECOREATCOR15 | 267 | (-) | CCGAC | S000153 | necessary for coldor drought |
| DEHYDRIN | LTRECOREATCOR15 | 682 | (+) | CCGAC | S000153 | necessary for coldor drought |
| DEHYDRIN | LTRECOREATCOR15 | 125 | (+) | CCGAC | S000153 | necessary for coldor drought |
| DEHYDRIN | LTRECOREATCOR15 | 373 | (-) | CCGAC | S000153 | necessary for coldor drought |
| DEHYDRIN | LTRECOREATCOR15 | 14 | (+) | CCGAC | S000153 | necessary for coldor drought |
| DEHYDRIN | LTRECOREATCOR15 | 49 | (+) | CCGAC | S000153 | necessary for coldor drought |
| DEHYDRIN | LTRECOREATCOR15 | 264 | (-) | CCGAC | S000153 | necessary for coldor drought |
| DEHYDRIN | LTRECOREATCOR15 | 344 | (+) | CCGAC | S000153 | necessary for coldor drought |
| DEHYDRIN | LTRECOREATCOR15 | 491 | (+) | CCGAC | S000153 | necessary for coldor drought |
| DEHYDRIN | LTRECOREATCOR15 | 833 | (+) | CCGAC | S000153 | necessary for coldor drought |
| DEHYDRIN | LTRECOREATCOR15 | 492 | (-) | CCGAC | S000153 | necessary for coldor drought |
| DEHYDRIN | LTRECOREATCOR15 | 528 | (-) | CCGAC | S000153 | necessary for coldor drought |
| DEHYDRIN | LTRECOREATCOR15 | 221 | (+) | CCGAC | S000153 | necessary for coldor drought |
| DEHYDRIN | LTRECOREATCOR15 | 227 | (+) | CCGAC | S000153 | necessary for coldor drought |
| DEHYDRIN | LTRECOREATCOR15 | 373 | (-) | CCGAC | S000153 | necessary for coldor drought |
| DEHYDRIN | LTRECOREATCOR15 | 408 | (-) | CCGAC | S000153 | necessary for coldor drought |
| DEHYDRIN | LTRECOREATCOR15 | 486 | (-) | CCGAC | S000153 | necessary for coldor drought |
| DEHYDRIN | LTRECOREATCOR15 | 187 | (-) | CCGAC | S000153 | necessary for coldor drought |
| DEHYDRIN | LTRECOREATCOR15 | 334 | (+) | CCGAC | S000153 | necessary for coldor drought |
| DEHYDRIN | LTRECOREATCOR15 | 292 | (-) | CCGAC | S000153 | necessary for coldor drought |
| DEHYDRIN | LTRECOREATCOR15 | 14 | (+) | CCGAC | S000153 | necessary for coldor drought |
| DEHYDRIN | LTRECOREATCOR15 | 49 | (+) | CCGAC | S000153 | necessary for coldor drought |
| DEHYDRIN | LTRECOREATCOR15 | 307 | (+) | CCGAC | S000153 | necessary for coldor drought |
| DEHYDRIN | LTRECOREATCOR15 | 406 | (-) | CCGAC | S000153 | necessary for coldor drought |
| DEHYDRIN | LTRECOREATCOR15 | 500 | (-) | CCGAC | S000153 | necessary for coldor drought |
| DEHYDRIN | LTRECOREATCOR15 | 565 | (-) | CCGAC | S000153 | necessary for coldor drought |
| DEHYDRIN | LTRECOREATCOR15 | 554 | (-) | CCGAC | S000153 | necessary for coldor drought |
| DEHYDRIN | LTRECOREATCOR15 | 454 | (-) | CCGAC | S000153 | necessary for coldor drought |
| DEHYDRIN | LTRECOREATCOR15 | 308 | (-) | CCGAC | S000153 | necessary for coldor drought |
| DEHYDRIN | LTRECOREATCOR15 | 240 | (-) | CCGAC | S000153 | necessary for coldor drought |
| DEHYDRIN | LTRECOREATCOR15 | 77 | (-) | CCGAC | S000153 | MYB recognition site |
| DEHYDRIN | MYB1AT | 134 | (-) | WAACCA | S000408 | MYB recognition site |
| DEHYDRIN | MYB1AT | 204 | (-) | WAACCA | S000408 | MYB recognition site |
| DEHYDRIN | MYB1AT | 158 | (-) | WAACCA | S000408 | MYB recognition site |
| DEHYDRIN | MYB1AT | 445 | (-) | WAACCA | S000408 | MYB recognition site |
| DEHYDRIN | MYB1AT | 8 | (+) | WAACCA | S000408 | MYB recognition site |
| DEHYDRIN | MYB1AT | 53 | (+) | WAACCA | S000408 | MYB recognition site |
| DEHYDRIN | MYB1AT | 429 | (-) | WAACCA | S000408 | MYB recognition site |
| DEHYDRIN | MYB1AT | 778 | (-) | WAACCA | S000408 | MYB recognition site |
| DEHYDRIN | MYB1AT | 53 | (+) | WAACCA | S000408 | MYB recognition site |
| DEHYDRIN | MYB1AT | 429 | (-) | WAACCA | S000408 | MYB recognition site |
| DEHYDRIN | MYB1AT | 488 | (-) | WAACCA | S000408 | MYB recognition site |
| DEHYDRIN | MYB1AT | 775 | (-) | WAACCA | S000408 | MYB recognition site |
| DEHYDRIN | MYB1AT | 842 | (-) | WAACCA | S000408 | MYB recognition site |
| DEHYDRIN | MYB1AT | 35 | (-) | WAACCA | S000408 | MYB recognition site |
| DEHYDRIN | MYB1AT | 150 | (-) | WAACCA | S000408 | MYB recognition site |
| DEHYDRIN | MYB1AT | 156 | (-) | WAACCA | S000408 | MYB recognition site |
| DEHYDRIN | MYB1AT | 777 | (-) | WAACCA | S000408 | MYB recognition site |
| DEHYDRIN | MYB1AT | 101 | (+) | WAACCA | S000408 | MYB recognition site |
| DEHYDRIN | MYB1AT | 450 | (+) | WAACCA | S000408 | MYB recognition site |
| DEHYDRIN | MYB1AT | 572 | (+) | WAACCA | S000408 | MYB recognition site |
| DEHYDRIN | MYB1AT | 878 | (-) | WAACCA | S000408 | MYB recognition site |
| DEHYDRIN | MYB1AT | 155 | (+) | WAACCA | S000408 | MYB recognition site |
| DEHYDRIN | MYB1AT | 288 | (+) | WAACCA | S000408 | MYB recognition site |
| DEHYDRIN | MYB1AT | 144 | (+) | WAACCA | S000408 | MYB recognition site |
| DEHYDRIN | MYB1AT | 143 | (-) | WAACCA | S000408 | MYB recognition site |
| DEHYDRIN | MYB1AT | 210 | (-) | WAACCA | S000408 | MYB recognition site |
| DEHYDRIN | MYB1AT | 366 | (-) | WAACCA | S000408 | MYB recognition site |
| DEHYDRIN | MYB1AT | 507 | (-) | WAACCA | S000408 | MYB recognition site |
| DEHYDRIN | MYB1AT | 413 | (+) | WAACCA | S000408 | MYB recognition site |
| DEHYDRIN | MYB1AT | 2 | (-) | WAACCA | S000408 | MYB recognition site |
| DEHYDRIN | MYB1AT | 31 | (-) | WAACCA | S000408 | MYB recognition site |
| DEHYDRIN | MYB1AT | 694 | (+) | WAACCA | S000408 | MYB recognition site |
| DEHYDRIN | MYB1LEPR | 439 | (+) | GTTAGTT | S000443 | MYB recognition site |
| DEHYDRIN | MYB1LEPR | 255 | (-) | GTTAGTT | S000443 | MYB recognition site |
| DEHYDRIN | MYB2AT | 710 | (-) | TAACTG | S000177 | MYB recognition site |
| DEHYDRIN | MYB2AT | 546 | (+) | TAACTG | S000177 | MYB recognition site |
| DEHYDRIN | MYB2AT | 546 | (+) | TAACTG | S000177 | MYB recognition site |
| DEHYDRIN | MYB2AT | 365 | (-) | TAACTG | S000177 | MYB recognition site |
| DEHYDRIN | MYB2AT | 726 | (-) | TAACTG | S000177 | MYB recognition site |
| DEHYDRIN | MYB2AT | 369 | (-) | TAACTG | S000177 | MYB recognition site |
| DEHYDRIN | MYB2AT | 516 | (-) | TAACTG | S000177 | MYB recognition site |
| DEHYDRIN | MYB2AT | 599 | (-) | TAACTG | S000177 | MYB recognition site |
| DEHYDRIN | MYB2AT | 519 | (+) | TAACTG | S000177 | MYB recognition site |
| DEHYDRIN | MYB2AT | 254 | (+) | TAACTG | S000177 | MYB recognition site |
| DEHYDRIN | MYB2AT | 365 | (-) | TAACTG | S000177 | MYB recognition site |
| DEHYDRIN | MYB2AT | 726 | (-) | TAACTG | S000177 | MYB recognition site |
| DEHYDRIN | MYB2AT | 182 | (+) | TAACTG | S000177 | MYB recognition site |
| DEHYDRIN | MYB2AT | 365 | (-) | TAACTG | S000177 | MYB recognition site |
| DEHYDRIN | MYB2AT | 290 | (-) | TAACTG | S000177 | MYB recognition site |
| DEHYDRIN | MYB2AT | 313 | (-) | TAACTG | S000177 | MYB recognition site |
| DEHYDRIN | MYB2CONSENSUSAT | 296 | (-) | YAACKG | S000409 | Dehydratio/water stress |
| DEHYDRIN | MYB2CONSENSUSAT | 287 | (-) | YAACKG | S000409 | Dehydratio/water stress |
| DEHYDRIN | MYB2CONSENSUSAT | 404 | (+) | YAACKG | S000409 | Dehydratio/water stress |
| DEHYDRIN | MYB2CONSENSUSAT | 710 | (-) | YAACKG | S000409 | Dehydratio/water stress |
| DEHYDRIN | MYB2CONSENSUSAT | 422 | (-) | YAACKG | S000409 | Dehydratio/water stress |
| DEHYDRIN | MYB2CONSENSUSAT | 546 | (+) | YAACKG | S000409 | Dehydratio/water stress |
| DEHYDRIN | MYB2CONSENSUSAT | 665 | (+) | YAACKG | S000409 | Dehydratio/water stress |
| DEHYDRIN | MYB2CONSENSUSAT | 546 | (+) | YAACKG | S000409 | Dehydratio/water stress |
| DEHYDRIN | MYB2CONSENSUSAT | 365 | (-) | YAACKG | S000409 | Dehydratio/water stress |
| DEHYDRIN | MYB2CONSENSUSAT | 726 | (-) | YAACKG | S000409 | Dehydratio/water stress |
| DEHYDRIN | MYB2CONSENSUSAT | 56 | (-) | YAACKG | S000409 | Dehydratio/water stress |
| DEHYDRIN | MYB2CONSENSUSAT | 369 | (-) | YAACKG | S000409 | Dehydratio/water stress |
| DEHYDRIN | MYB2CONSENSUSAT | 392 | (+) | YAACKG | S000409 | Dehydratio/water stress |
| DEHYDRIN | MYB2CONSENSUSAT | 263 | (+) | YAACKG | S000409 | Dehydratio/water stress |
| DEHYDRIN | MYB2CONSENSUSAT | 437 | (-) | YAACKG | S000409 | Dehydratio/water stress |
| DEHYDRIN | MYB2CONSENSUSAT | 449 | (+) | YAACKG | S000409 | Dehydratio/water stress |
| DEHYDRIN | MYB2CONSENSUSAT | 516 | (-) | YAACKG | S000409 | Dehydratio/water stress |
| DEHYDRIN | MYB2CONSENSUSAT | 599 | (-) | YAACKG | S000409 | Dehydratio/water stress |
| DEHYDRIN | MYB2CONSENSUSAT | 566 | (+) | YAACKG | S000409 | Dehydratio/water stress |
| DEHYDRIN | MYB2CONSENSUSAT | 824 | (-) | YAACKG | S000409 | Dehydratio/water stress |
| DEHYDRIN | MYB2CONSENSUSAT | 840 | (-) | YAACKG | S000409 | Dehydratio/water stress |
| DEHYDRIN | MYB2CONSENSUSAT | 896 | (+) | YAACKG | S000409 | Dehydratio/water stress |
| DEHYDRIN | MYB2CONSENSUSAT | 92 | (-) | YAACKG | S000409 | Dehydratio/water stress |
| DEHYDRIN | MYB2CONSENSUSAT | 512 | (-) | YAACKG | S000409 | Dehydratio/water stress |
| DEHYDRIN | MYB2CONSENSUSAT | 667 | (-) | YAACKG | S000409 | Dehydratio/water stress |
| DEHYDRIN | MYB2CONSENSUSAT | 28 | (+) | YAACKG | S000409 | Dehydratio/water stress |
| DEHYDRIN | MYB2CONSENSUSAT | 191 | (-) | YAACKG | S000409 | Dehydratio/water stress |
| DEHYDRIN | MYB2CONSENSUSAT | 566 | (+) | YAACKG | S000409 | Dehydratio/water stress |
| DEHYDRIN | MYB2CONSENSUSAT | 743 | (+) | YAACKG | S000409 | Dehydratio/water stress |
| DEHYDRIN | MYB2CONSENSUSAT | 519 | (+) | YAACKG | S000409 | Dehydratio/water stress |
| DEHYDRIN | MYB2CONSENSUSAT | 216 | (+) | YAACKG | S000409 | Dehydratio/water stress |
| DEHYDRIN | MYB2CONSENSUSAT | 337 | (-) | YAACKG | S000409 | Dehydratio/water stress |
| DEHYDRIN | MYB2CONSENSUSAT | 603 | (-) | YAACKG | S000409 | Dehydratio/water stress |
| DEHYDRIN | MYB2CONSENSUSAT | 254 | (+) | YAACKG | S000409 | Dehydratio/water stress |
| DEHYDRIN | MYB2CONSENSUSAT | 626 | (+) | YAACKG | S000409 | Dehydratio/water stress |
| DEHYDRIN | MYB2CONSENSUSAT | 635 | (+) | YAACKG | S000409 | Dehydratio/water stress |
| DEHYDRIN | MYB2CONSENSUSAT | 365 | (-) | YAACKG | S000409 | Dehydratio/water stress |
| DEHYDRIN | MYB2CONSENSUSAT | 726 | (-) | YAACKG | S000409 | Dehydratio/water stress |
| DEHYDRIN | MYB2CONSENSUSAT | 182 | (+) | YAACKG | S000409 | Dehydratio/water stress |
| DEHYDRIN | MYB2CONSENSUSAT | 365 | (-) | YAACKG | S000409 | Dehydratio/water stress |
| DEHYDRIN | MYB2CONSENSUSAT | 139 | (-) | YAACKG | S000409 | Dehydratio/water stress |
| DEHYDRIN | MYB2CONSENSUSAT | 290 | (-) | YAACKG | S000409 | Dehydratio/water stress |
| DEHYDRIN | MYB2CONSENSUSAT | 328 | (+) | YAACKG | S000409 | Dehydratio/water stress |
| DEHYDRIN | MYB2CONSENSUSAT | 313 | (-) | YAACKG | S000409 | Dehydratio/water stress |
| DEHYDRIN | MYB2CONSENSUSAT | 604 | (-) | YAACKG | S000409 | Dehydratio/water stress |
| DEHYDRIN | MYBCORE | 296 | (+) | CNGTTR | S000176 | Dehydratio/water stress |
| DEHYDRIN | MYBCORE | 333 | (-) | CNGTTR | S000176 | Dehydratio/water stress |
| DEHYDRIN | MYBCORE | 605 | (-) | CNGTTR | S000176 | Dehydratio/water stress |
| DEHYDRIN | MYBCORE | 287 | (+) | CNGTTR | S000176 | Dehydratio/water stress |
| DEHYDRIN | MYBCORE | 404 | (-) | CNGTTR | S000176 | Dehydratio/water stress |
| DEHYDRIN | MYBCORE | 317 | (+) | CNGTTR | S000176 | Dehydratio/water stress |
| DEHYDRIN | MYBCORE | 323 | (+) | CNGTTR | S000176 | Dehydratio/water stress |
| DEHYDRIN | MYBCORE | 710 | (+) | CNGTTR | S000176 | Dehydratio/water stress |
| DEHYDRIN | MYBCORE | 290 | (-) | CNGTTR | S000176 | Dehydratio/water stress |
| DEHYDRIN | MYBCORE | 436 | (+) | CNGTTR | S000176 | Dehydratio/water stress |
| DEHYDRIN | MYBCORE | 451 | (+) | CNGTTR | S000176 | Dehydratio/water stress |
| DEHYDRIN | MYBCORE | 138 | (+) | CNGTTR | S000176 | Dehydratio/water stress |
| DEHYDRIN | MYBCORE | 212 | (+) | CNGTTR | S000176 | Dehydratio/water stress |
| DEHYDRIN | MYBCORE | 332 | (-) | CNGTTR | S000176 | Dehydratio/water stress |
| DEHYDRIN | MYBCORE | 422 | (+) | CNGTTR | S000176 | Dehydratio/water stress |
| DEHYDRIN | MYBCORE | 379 | (+) | CNGTTR | S000176 | Dehydratio/water stress |
| DEHYDRIN | MYBCORE | 546 | (-) | CNGTTR | S000176 | Dehydratio/water stress |
| DEHYDRIN | MYBCORE | 557 | (+) | CNGTTR | S000176 | Dehydratio/water stress |
| DEHYDRIN | MYBCORE | 584 | (-) | CNGTTR | S000176 | Dehydratio/water stress |
| DEHYDRIN | MYBCORE | 407 | (-) | CNGTTR | S000176 | Dehydratio/water stress |
| DEHYDRIN | MYBCORE | 466 | (+) | CNGTTR | S000176 | Dehydratio/water stress |
| DEHYDRIN | MYBCORE | 665 | (-) | CNGTTR | S000176 | Dehydratio/water stress |
| DEHYDRIN | MYBCORE | 379 | (+) | CNGTTR | S000176 | Dehydratio/water stress |
| DEHYDRIN | MYBCORE | 546 | (-) | CNGTTR | S000176 | Dehydratio/water stress |
| DEHYDRIN | MYBCORE | 557 | (+) | CNGTTR | S000176 | Dehydratio/water stress |
| DEHYDRIN | MYBCORE | 584 | (-) | CNGTTR | S000176 | Dehydratio/water stress |
| DEHYDRIN | MYBCORE | 69 | (-) | CNGTTR | S000176 | Dehydratio/water stress |
| DEHYDRIN | MYBCORE | 365 | (+) | CNGTTR | S000176 | Dehydratio/water stress |
| DEHYDRIN | MYBCORE | 726 | (+) | CNGTTR | S000176 | Dehydratio/water stress |
| DEHYDRIN | MYBCORE | 10 | (-) | CNGTTR | S000176 | Dehydratio/water stress |
| DEHYDRIN | MYBCORE | 56 | (+) | CNGTTR | S000176 | Dehydratio/water stress |
| DEHYDRIN | MYBCORE | 140 | (-) | CNGTTR | S000176 | Dehydratio/water stress |
| DEHYDRIN | MYBCORE | 355 | (-) | CNGTTR | S000176 | Dehydratio/water stress |
| DEHYDRIN | MYBCORE | 407 | (-) | CNGTTR | S000176 | Dehydratio/water stress |
| DEHYDRIN | MYBCORE | 407 | (-) | CNGTTR | S000176 | Dehydratio/water stress |
| DEHYDRIN | MYBCORE | 466 | (+) | CNGTTR | S000176 | Dehydratio/water stress |
| DEHYDRIN | MYBCORE | 369 | (+) | CNGTTR | S000176 | Dehydratio/water stress |
| DEHYDRIN | MYBCORE | 338 | (-) | CNGTTR | S000176 | Dehydratio/water stress |
| DEHYDRIN | MYBCORE | 392 | (-) | CNGTTR | S000176 | Dehydratio/water stress |
| DEHYDRIN | MYBCORE | 228 | (-) | CNGTTR | S000176 | Dehydratio/water stress |
| DEHYDRIN | MYBCORE | 379 | (+) | CNGTTR | S000176 | Dehydratio/water stress |
| DEHYDRIN | MYBCORE | 263 | (-) | CNGTTR | S000176 | Dehydratio/water stress |
| DEHYDRIN | MYBCORE | 437 | (+) | CNGTTR | S000176 | Dehydratio/water stress |
| DEHYDRIN | MYBCORE | 449 | (-) | CNGTTR | S000176 | Dehydratio/water stress |
| DEHYDRIN | MYBCORE | 516 | (+) | CNGTTR | S000176 | Dehydratio/water stress |
| DEHYDRIN | MYBCORE | 599 | (+) | CNGTTR | S000176 | Dehydratio/water stress |
| DEHYDRIN | MYBCORE | 566 | (-) | CNGTTR | S000176 | Dehydratio/water stress |
| DEHYDRIN | MYBCORE | 824 | (+) | CNGTTR | S000176 | Dehydratio/water stress |
| DEHYDRIN | MYBCORE | 840 | (+) | CNGTTR | S000176 | Dehydratio/water stress |
| DEHYDRIN | MYBCORE | 896 | (-) | CNGTTR | S000176 | Dehydratio/water stress |
| DEHYDRIN | MYBCORE | 92 | (+) | CNGTTR | S000176 | Dehydratio/water stress |
| DEHYDRIN | MYBCORE | 207 | (-) | CNGTTR | S000176 | Dehydratio/water stress |
| DEHYDRIN | MYBCORE | 830 | (-) | CNGTTR | S000176 | Dehydratio/water stress |
| DEHYDRIN | MYBCORE | 965 | (-) | CNGTTR | S000176 | Dehydratio/water stress |
| DEHYDRIN | MYBCORE | 512 | (+) | CNGTTR | S000176 | Dehydratio/water stress |
| DEHYDRIN | MYBCORE | 147 | (+) | CNGTTR | S000176 | Dehydratio/water stress |
| DEHYDRIN | MYBCORE | 542 | (+) | CNGTTR | S000176 | Dehydratio/water stress |
| DEHYDRIN | MYBCORE | 667 | (+) | CNGTTR | S000176 | Dehydratio/water stress |
| DEHYDRIN | MYBCORE | 28 | (-) | CNGTTR | S000176 | Dehydratio/water stress |
| DEHYDRIN | MYBCORE | 156 | (-) | CNGTTR | S000176 | Dehydratio/water stress |
| DEHYDRIN | MYBCORE | 75 | (-) | CNGTTR | S000176 | Dehydratio/water stress |
| DEHYDRIN | MYBCORE | 211 | (-) | CNGTTR | S000176 | Dehydratio/water stress |
| DEHYDRIN | MYBCORE | 217 | (-) | CNGTTR | S000176 | Dehydratio/water stress |
| DEHYDRIN | MYBCORE | 587 | (-) | CNGTTR | S000176 | Dehydratio/water stress |
| DEHYDRIN | MYBCORE | 191 | (+) | CNGTTR | S000176 | Dehydratio/water stress |
| DEHYDRIN | MYBCORE | 566 | (-) | CNGTTR | S000176 | Dehydratio/water stress |
| DEHYDRIN | MYBCORE | 743 | (-) | CNGTTR | S000176 | Dehydratio/water stress |
| DEHYDRIN | MYBCORE | 519 | (-) | CNGTTR | S000176 | Dehydratio/water stress |
| DEHYDRIN | MYBCORE | 127 | (-) | CNGTTR | S000176 | Dehydratio/water stress |
| DEHYDRIN | MYBCORE | 216 | (-) | CNGTTR | S000176 | Dehydratio/water stress |
| DEHYDRIN | MYBCORE | 337 | (+) | CNGTTR | S000176 | Dehydratio/water stress |
| DEHYDRIN | MYBCORE | 377 | (+) | CNGTTR | S000176 | Dehydratio/water stress |
| DEHYDRIN | MYBCORE | 556 | (+) | CNGTTR | S000176 | Dehydratio/water stress |
| DEHYDRIN | MYBCORE | 305 | (+) | CNGTTR | S000176 | Dehydratio/water stress |
| DEHYDRIN | MYBCORE | 603 | (+) | CNGTTR | S000176 | Dehydratio/water stress |
| DEHYDRIN | MYBCORE | 617 | (+) | CNGTTR | S000176 | Dehydratio/water stress |
| DEHYDRIN | MYBCORE | 172 | (+) | CNGTTR | S000176 | Dehydratio/water stress |
| DEHYDRIN | MYBCORE | 422 | (+) | CNGTTR | S000176 | Dehydratio/water stress |
| DEHYDRIN | MYBCORE | 254 | (-) | CNGTTR | S000176 | Dehydratio/water stress |
| DEHYDRIN | MYBCORE | 371 | (+) | CNGTTR | S000176 | Dehydratio/water stress |
| DEHYDRIN | MYBCORE | 437 | (-) | CNGTTR | S000176 | Dehydratio/water stress |
| DEHYDRIN | MYBCORE | 626 | (-) | CNGTTR | S000176 | Dehydratio/water stress |
| DEHYDRIN | MYBCORE | 635 | (-) | CNGTTR | S000176 | Dehydratio/water stress |
| DEHYDRIN | MYBCORE | 69 | (-) | CNGTTR | S000176 | Dehydratio/water stress |
| DEHYDRIN | MYBCORE | 365 | (+) | CNGTTR | S000176 | Dehydratio/water stress |
| DEHYDRIN | MYBCORE | 726 | (+) | CNGTTR | S000176 | Dehydratio/water stress |
| DEHYDRIN | MYBCORE | 182 | (-) | CNGTTR | S000176 | Dehydratio/water stress |
| DEHYDRIN | MYBCORE | 474 | (-) | CNGTTR | S000176 | Dehydratio/water stress |
| DEHYDRIN | MYBCORE | 69 | (-) | CNGTTR | S000176 | Dehydratio/water stress |
| DEHYDRIN | MYBCORE | 365 | (+) | CNGTTR | S000176 | Dehydratio/water stress |
| DEHYDRIN | MYBCORE | 32 | (-) | CNGTTR | S000176 | Dehydratio/water stress |
| DEHYDRIN | MYBCORE | 139 | (+) | CNGTTR | S000176 | Dehydratio/water stress |
| DEHYDRIN | MYBCORE | 290 | (+) | CNGTTR | S000176 | Dehydratio/water stress |
| DEHYDRIN | MYBCORE | 328 | (-) | CNGTTR | S000176 | Dehydratio/water stress |
| DEHYDRIN | MYBCORE | 310 | (-) | CNGTTR | S000176 | Dehydratio/water stress |
| DEHYDRIN | MYBCORE | 313 | (+) | CNGTTR | S000176 | Dehydratio/water stress |
| DEHYDRIN | MYBCORE | 604 | (+) | CNGTTR | S000176 | Dehydratio/water stress |
| DEHYDRIN | MYBCOREATCYCB1 | 296 | (-) | AACGG | S000502 | Dehydratio/water stress |
| DEHYDRIN | MYBCOREATCYCB1 | 287 | (-) | AACGG | S000502 | Dehydratio/water stress |
| DEHYDRIN | MYBCOREATCYCB1 | 670 | (+) | AACGG | S000502 | Dehydratio/water stress |
| DEHYDRIN | MYBCOREATCYCB1 | 422 | (-) | AACGG | S000502 | Dehydratio/water stress |
| DEHYDRIN | MYBCOREATCYCB1 | 844 | (-) | AACGG | S000502 | Dehydratio/water stress |
| DEHYDRIN | MYBCOREATCYCB1 | 56 | (-) | AACGG | S000502 | Dehydratio/water stress |
| DEHYDRIN | MYBCOREATCYCB1 | 108 | (-) | AACGG | S000502 | Dehydratio/water stress |
| DEHYDRIN | MYBCOREATCYCB1 | 198 | (-) | AACGG | S000502 | Dehydratio/water stress |
| DEHYDRIN | MYBCOREATCYCB1 | 113 | (-) | AACGG | S000502 | Dehydratio/water stress |
| DEHYDRIN | MYBCOREATCYCB1 | 315 | (+) | AACGG | S000502 | Dehydratio/water stress |
| DEHYDRIN | MYBCOREATCYCB1 | 393 | (+) | AACGG | S000502 | Dehydratio/water stress |
| DEHYDRIN | MYBCOREATCYCB1 | 437 | (-) | AACGG | S000502 | Dehydratio/water stress |
| DEHYDRIN | MYBCOREATCYCB1 | 450 | (+) | AACGG | S000502 | Dehydratio/water stress |
| DEHYDRIN | MYBCOREATCYCB1 | 71 | (-) | AACGG | S000502 | Dehydratio/water stress |
| DEHYDRIN | MYBCOREATCYCB1 | 897 | (+) | AACGG | S000502 | Dehydratio/water stress |
| DEHYDRIN | MYBCOREATCYCB1 | 181 | (-) | AACGG | S000502 | Dehydratio/water stress |
| DEHYDRIN | MYBCOREATCYCB1 | 532 | (-) | AACGG | S000502 | Dehydratio/water stress |
| DEHYDRIN | MYBCOREATCYCB1 | 265 | (+) | AACGG | S000502 | Dehydratio/water stress |
| DEHYDRIN | MYBCOREATCYCB1 | 71 | (-) | AACGG | S000502 | Dehydratio/water stress |
| DEHYDRIN | MYBCOREATCYCB1 | 191 | (-) | AACGG | S000502 | Dehydratio/water stress |
| DEHYDRIN | MYBCOREATCYCB1 | 744 | (+) | AACGG | S000502 | Dehydratio/water stress |
| DEHYDRIN | MYBCOREATCYCB1 | 656 | (-) | AACGG | S000502 | Dehydratio/water stress |
| DEHYDRIN | MYBCOREATCYCB1 | 217 | (+) | AACGG | S000502 | Dehydratio/water stress |
| DEHYDRIN | MYBCOREATCYCB1 | 488 | (-) | AACGG | S000502 | Dehydratio/water stress |
| DEHYDRIN | MYBCOREATCYCB1 | 627 | (+) | AACGG | S000502 | Dehydratio/water stress |
| DEHYDRIN | MYBCOREATCYCB1 | 636 | (+) | AACGG | S000502 | Dehydratio/water stress |
| DEHYDRIN | MYBCOREATCYCB1 | 718 | (+) | AACGG | S000502 | Dehydratio/water stress |
| DEHYDRIN | MYBCOREATCYCB1 | 844 | (-) | AACGG | S000502 | Dehydratio/water stress |
| DEHYDRIN | MYBCOREATCYCB1 | 139 | (-) | AACGG | S000502 | Dehydratio/water stress |
| DEHYDRIN | MYBCOREATCYCB1 | 604 | (-) | AACGG | S000502 | MYB recognition site |
| DEHYDRIN | MYBPLANT | 349 | (-) | MACCWAMC | S000167 | MYB recognition site |
| DEHYDRIN | MYBPLANT | 426 | (-) | MACCWAMC | S000167 | MYB recognition site |
| DEHYDRIN | MYBPLANT | 426 | (-) | MACCWAMC | S000167 | MYB recognition site |
| DEHYDRIN | MYBPLANT | 51 | (+) | MACCWAMC | S000167 | MYB recognition site |
| DEHYDRIN | MYBPLANT | 266 | (+) | MACCWAMC | S000167 | MYB recognition site |
| DEHYDRIN | MYBPLANT | 324 | (+) | MACCWAMC | S000167 | MYB recognition site |
| DEHYDRIN | MYBPLANT | 631 | (-) | MACCWAMC | S000167 | MYB recognition site |
| DEHYDRIN | MYBPZM | 673 | (-) | CCWACC | S000179 | MYB recognition site |
| DEHYDRIN | MYBPZM | 44 | (+) | CCWACC | S000179 | MYB recognition site |
| DEHYDRIN | MYBPZM | 212 | (+) | CCWACC | S000179 | MYB recognition site |
| DEHYDRIN | MYBPZM | 34 | (+) | CCWACC | S000179 | MYB recognition site |
| DEHYDRIN | MYBPZM | 270 | (-) | CCWACC | S000179 | MYB recognition site |
| DEHYDRIN | MYBPZM | 507 | (-) | CCWACC | S000179 | MYB recognition site |
| DEHYDRIN | MYBPZM | 270 | (-) | CCWACC | S000179 | MYB recognition site |
| DEHYDRIN | MYBPZM | 507 | (-) | CCWACC | S000179 | MYB recognition site |
| DEHYDRIN | MYBPZM | 15 | (+) | CCWACC | S000179 | MYB recognition site |
| DEHYDRIN | MYBPZM | 246 | (+) | CCWACC | S000179 | MYB recognition site |
| DEHYDRIN | MYBPZM | 53 | (+) | CCWACC | S000179 | MYB recognition site |
| DEHYDRIN | MYBPZM | 624 | (-) | CCWACC | S000179 | MYB recognition site |
| DEHYDRIN | MYBPZM | 294 | (-) | CCWACC | S000179 | MYB recognition site |
| DEHYDRIN | MYBPZM | 49 | (+) | CCWACC | S000179 | MYB recognition site |
| DEHYDRIN | MYBPZM | 627 | (-) | CCWACC | S000179 | MYB recognition site |
| DEHYDRIN | MYBPZM | 81 | (+) | CCWACC | S000179 | MYB recognition site |
| DEHYDRIN | MYBPZM | 124 | (+) | CCWACC | S000179 | MYB recognition site |
| DEHYDRIN | MYBPZM | 129 | (+) | CCWACC | S000179 | MYB recognition site |
| DEHYDRIN | MYBPZM | 326 | (+) | CCWACC | S000179 | MYB recognition site |
| DEHYDRIN | MYBPZM | 107 | (+) | CCWACC | S000179 | MYB recognition site |
| DEHYDRIN | MYBPZM | 126 | (+) | CCWACC | S000179 | MYB recognition site |
| DEHYDRIN | MYBPZM | 299 | (+) | CCWACC | S000179 | MYB recognition site |
| DEHYDRIN | MYBPZM | 397 | (+) | CCWACC | S000179 | MYB recognition site |
| DEHYDRIN | MYBPZM | 423 | (-) | CCWACC | S000179 | MYB recognition site |
| DEHYDRIN | MYBST1 | 46 | (-) | GGATA | S000180 | MYB responsive |
| DEHYDRIN | MYBST1 | 74 | (+) | GGATA | S000180 | MYB responsive |
| DEHYDRIN | MYBST1 | 386 | (+) | GGATA | S000180 | MYB responsive |
| DEHYDRIN | MYBST1 | 171 | (+) | GGATA | S000180 | MYB responsive |
| DEHYDRIN | MYBST1 | 354 | (-) | GGATA | S000180 | MYB responsive |
| DEHYDRIN | MYBST1 | 231 | (-) | GGATA | S000180 | MYB responsive |
| DEHYDRIN | MYBST1 | 837 | (+) | GGATA | S000180 | MYB responsive |
| DEHYDRIN | MYBST1 | 231 | (-) | GGATA | S000180 | MYB responsive |
| DEHYDRIN | MYBST1 | 419 | (-) | GGATA | S000180 | MYB responsive |
| DEHYDRIN | MYBST1 | 834 | (+) | GGATA | S000180 | MYB responsive |
| DEHYDRIN | MYBST1 | 180 | (-) | GGATA | S000180 | MYB responsive |
| DEHYDRIN | MYBST1 | 629 | (+) | GGATA | S000180 | MYB responsive |
| DEHYDRIN | MYBST1 | 122 | (-) | GGATA | S000180 | MYB responsive |
| DEHYDRIN | MYBST1 | 528 | (-) | GGATA | S000180 | MYB responsive |
| DEHYDRIN | MYBST1 | 890 | (-) | GGATA | S000180 | MYB responsive |
| DEHYDRIN | MYBST1 | 267 | (+) | GGATA | S000180 | MYB responsive |
| DEHYDRIN | MYBST1 | 338 | (+) | GGATA | S000180 | MYB responsive |
| DEHYDRIN | MYBST1 | 483 | (+) | GGATA | S000180 | MYB responsive |
| DEHYDRIN | MYBST1 | 294 | (+) | GGATA | S000180 | MYB responsive |
| DEHYDRIN | MYBST1 | 69 | (+) | GGATA | S000180 | MYB responsive |
| DEHYDRIN | MYBST1 | 480 | (+) | GGATA | S000180 | MYB responsive |
| DEHYDRIN | MYBST1 | 737 | (-) | GGATA | S000180 | MYB responsive |
| DEHYDRIN | MYBST1 | 581 | (-) | GGATA | S000180 | MYB responsive |
| DEHYDRIN | MYBST1 | 52 | (-) | GGATA | S000180 | MYB responsive |
| DEHYDRIN | MYBST1 | 186 | (-) | GGATA | S000180 | MYB responsive |
| DEHYDRIN | MYBST1 | 123 | (-) | GGATA | S000180 | MYB responsive |
| DEHYDRIN | MYBST1 | 252 | (+) | GGATA | S000180 | MYB responsive |
| DEHYDRIN | MYBST1 | 654 | (+) | GGATA | S000180 | MYB responsive |
| DEHYDRIN | MYBST1 | 531 | (+) | GGATA | S000180 | MYB responsive |
| DEHYDRIN | MYBST1 | 107 | (+) | GGATA | S000180 | MYB responsive |
| DEHYDRIN | MYBST1 | 305 | (+) | GGATA | S000180 | Dehydratio/water stress |
| DEHYDRIN | MYCATERD1 | 332 | (-) | CATGTG | S000413 | Dehydratio/water stress |
| DEHYDRIN | MYCATERD1 | 773 | (-) | CATGTG | S000413 | Dehydratio/water stress |
| DEHYDRIN | MYCATERD1 | 250 | (+) | CATGTG | S000413 | Dehydratio/water stress |
| DEHYDRIN | MYCATERD1 | 278 | (-) | CATGTG | S000413 | Dehydratio/water stress |
| DEHYDRIN | MYCATERD1 | 526 | (+) | CATGTG | S000413 | Dehydratio/water stress |
| DEHYDRIN | MYCATERD1 | 283 | (+) | CATGTG | S000413 | Dehydratio/water stress |
| DEHYDRIN | MYCATRD22 | 332 | (+) | CACATG | S000174 | Dehydratio/water stress |
| DEHYDRIN | MYCATRD22 | 773 | (+) | CACATG | S000174 | Dehydratio/water stress |
| DEHYDRIN | MYCATRD22 | 250 | (-) | CACATG | S000174 | Dehydratio/water stress |
| DEHYDRIN | MYCATRD22 | 278 | (+) | CACATG | S000174 | Dehydratio/water stress |
| DEHYDRIN | MYCATRD22 | 526 | (-) | CACATG | S000174 | Dehydratio/water stress |
| DEHYDRIN | MYCATRD22 | 283 | (-) | CACATG | S000174 | Dehydratio/water stress |
| DEHYDRIN | MYCCONSENSUSAT | 114 | (-) | CANNTG | S000407 | Dehydratio/water stress |
| DEHYDRIN | MYCCONSENSUSAT | 114 | (+) | CANNTG | S000407 | Dehydratio/water stress |
| DEHYDRIN | MYCCONSENSUSAT | 413 | (-) | CANNTG | S000407 | Dehydratio/water stress |
| DEHYDRIN | MYCCONSENSUSAT | 413 | (+) | CANNTG | S000407 | Dehydratio/water stress |
| DEHYDRIN | MYCCONSENSUSAT | 439 | (-) | CANNTG | S000407 | Dehydratio/water stress |
| DEHYDRIN | MYCCONSENSUSAT | 439 | (+) | CANNTG | S000407 | Dehydratio/water stress |
| DEHYDRIN | MYCCONSENSUSAT | 105 | (-) | CANNTG | S000407 | Dehydratio/water stress |
| DEHYDRIN | MYCCONSENSUSAT | 105 | (+) | CANNTG | S000407 | Dehydratio/water stress |
| DEHYDRIN | MYCCONSENSUSAT | 191 | (-) | CANNTG | S000407 | Dehydratio/water stress |
| DEHYDRIN | MYCCONSENSUSAT | 191 | (+) | CANNTG | S000407 | Dehydratio/water stress |
| DEHYDRIN | MYCCONSENSUSAT | 404 | (-) | CANNTG | S000407 | Dehydratio/water stress |
| DEHYDRIN | MYCCONSENSUSAT | 404 | (+) | CANNTG | S000407 | Dehydratio/water stress |
| DEHYDRIN | MYCCONSENSUSAT | 293 | (-) | CANNTG | S000407 | Dehydratio/water stress |
| DEHYDRIN | MYCCONSENSUSAT | 293 | (+) | CANNTG | S000407 | Dehydratio/water stress |
| DEHYDRIN | MYCCONSENSUSAT | 4 | (-) | CANNTG | S000407 | Dehydratio/water stress |
| DEHYDRIN | MYCCONSENSUSAT | 4 | (+) | CANNTG | S000407 | Dehydratio/water stress |
| DEHYDRIN | MYCCONSENSUSAT | 358 | (-) | CANNTG | S000407 | Dehydratio/water stress |
| DEHYDRIN | MYCCONSENSUSAT | 358 | (+) | CANNTG | S000407 | Dehydratio/water stress |
| DEHYDRIN | MYCCONSENSUSAT | 548 | (-) | CANNTG | S000407 | Dehydratio/water stress |
| DEHYDRIN | MYCCONSENSUSAT | 548 | (+) | CANNTG | S000407 | Dehydratio/water stress |
| DEHYDRIN | MYCCONSENSUSAT | 132 | (-) | CANNTG | S000407 | Dehydratio/water stress |
| DEHYDRIN | MYCCONSENSUSAT | 132 | (+) | CANNTG | S000407 | Dehydratio/water stress |
| DEHYDRIN | MYCCONSENSUSAT | 498 | (-) | CANNTG | S000407 | Dehydratio/water stress |
| DEHYDRIN | MYCCONSENSUSAT | 498 | (+) | CANNTG | S000407 | Dehydratio/water stress |
| DEHYDRIN | MYCCONSENSUSAT | 261 | (-) | CANNTG | S000407 | Dehydratio/water stress |
| DEHYDRIN | MYCCONSENSUSAT | 261 | (+) | CANNTG | S000407 | Dehydratio/water stress |
| DEHYDRIN | MYCCONSENSUSAT | 442 | (-) | CANNTG | S000407 | Dehydratio/water stress |
| DEHYDRIN | MYCCONSENSUSAT | 442 | (+) | CANNTG | S000407 | Dehydratio/water stress |
| DEHYDRIN | MYCCONSENSUSAT | 665 | (-) | CANNTG | S000407 | Dehydratio/water stress |
| DEHYDRIN | MYCCONSENSUSAT | 665 | (+) | CANNTG | S000407 | Dehydratio/water stress |
| DEHYDRIN | MYCCONSENSUSAT | 893 | (-) | CANNTG | S000407 | Dehydratio/water stress |
| DEHYDRIN | MYCCONSENSUSAT | 893 | (+) | CANNTG | S000407 | Dehydratio/water stress |
| DEHYDRIN | MYCCONSENSUSAT | 132 | (-) | CANNTG | S000407 | Dehydratio/water stress |
| DEHYDRIN | MYCCONSENSUSAT | 132 | (+) | CANNTG | S000407 | Dehydratio/water stress |
| DEHYDRIN | MYCCONSENSUSAT | 498 | (-) | CANNTG | S000407 | Dehydratio/water stress |
| DEHYDRIN | MYCCONSENSUSAT | 498 | (+) | CANNTG | S000407 | Dehydratio/water stress |
| DEHYDRIN | MYCCONSENSUSAT | 158 | (-) | CANNTG | S000407 | Dehydratio/water stress |
| DEHYDRIN | MYCCONSENSUSAT | 158 | (+) | CANNTG | S000407 | Dehydratio/water stress |
| DEHYDRIN | MYCCONSENSUSAT | 317 | (-) | CANNTG | S000407 | Dehydratio/water stress |
| DEHYDRIN | MYCCONSENSUSAT | 317 | (+) | CANNTG | S000407 | Dehydratio/water stress |
| DEHYDRIN | MYCCONSENSUSAT | 398 | (-) | CANNTG | S000407 | Dehydratio/water stress |
| DEHYDRIN | MYCCONSENSUSAT | 398 | (+) | CANNTG | S000407 | Dehydratio/water stress |
| DEHYDRIN | MYCCONSENSUSAT | 595 | (-) | CANNTG | S000407 | Dehydratio/water stress |
| DEHYDRIN | MYCCONSENSUSAT | 595 | (+) | CANNTG | S000407 | Dehydratio/water stress |
| DEHYDRIN | MYCCONSENSUSAT | 521 | (-) | CANNTG | S000407 | Dehydratio/water stress |
| DEHYDRIN | MYCCONSENSUSAT | 521 | (+) | CANNTG | S000407 | Dehydratio/water stress |
| DEHYDRIN | MYCCONSENSUSAT | 611 | (-) | CANNTG | S000407 | Dehydratio/water stress |
| DEHYDRIN | MYCCONSENSUSAT | 611 | (+) | CANNTG | S000407 | Dehydratio/water stress |
| DEHYDRIN | MYCCONSENSUSAT | 442 | (-) | CANNTG | S000407 | Dehydratio/water stress |
| DEHYDRIN | MYCCONSENSUSAT | 442 | (+) | CANNTG | S000407 | Dehydratio/water stress |
| DEHYDRIN | MYCCONSENSUSAT | 890 | (-) | CANNTG | S000407 | Dehydratio/water stress |
| DEHYDRIN | MYCCONSENSUSAT | 890 | (+) | CANNTG | S000407 | Dehydratio/water stress |
| DEHYDRIN | MYCCONSENSUSAT | 32 | (-) | CANNTG | S000407 | Dehydratio/water stress |
| DEHYDRIN | MYCCONSENSUSAT | 32 | (+) | CANNTG | S000407 | Dehydratio/water stress |
| DEHYDRIN | MYCCONSENSUSAT | 518 | (-) | CANNTG | S000407 | Dehydratio/water stress |
| DEHYDRIN | MYCCONSENSUSAT | 518 | (+) | CANNTG | S000407 | Dehydratio/water stress |
| DEHYDRIN | MYCCONSENSUSAT | 247 | (-) | CANNTG | S000407 | Dehydratio/water stress |
| DEHYDRIN | MYCCONSENSUSAT | 247 | (+) | CANNTG | S000407 | Dehydratio/water stress |
| DEHYDRIN | MYCCONSENSUSAT | 332 | (-) | CANNTG | S000407 | Dehydratio/water stress |
| DEHYDRIN | MYCCONSENSUSAT | 332 | (+) | CANNTG | S000407 | Dehydratio/water stress |
| DEHYDRIN | MYCCONSENSUSAT | 507 | (-) | CANNTG | S000407 | Dehydratio/water stress |
| DEHYDRIN | MYCCONSENSUSAT | 507 | (+) | CANNTG | S000407 | Dehydratio/water stress |
| DEHYDRIN | MYCCONSENSUSAT | 263 | (-) | CANNTG | S000407 | Dehydratio/water stress |
| DEHYDRIN | MYCCONSENSUSAT | 263 | (+) | CANNTG | S000407 | Dehydratio/water stress |
| DEHYDRIN | MYCCONSENSUSAT | 424 | (-) | CANNTG | S000407 | Dehydratio/water stress |
| DEHYDRIN | MYCCONSENSUSAT | 424 | (+) | CANNTG | S000407 | Dehydratio/water stress |
| DEHYDRIN | MYCCONSENSUSAT | 95 | (-) | CANNTG | S000407 | Dehydratio/water stress |
| DEHYDRIN | MYCCONSENSUSAT | 95 | (+) | CANNTG | S000407 | Dehydratio/water stress |
| DEHYDRIN | MYCCONSENSUSAT | 566 | (-) | CANNTG | S000407 | Dehydratio/water stress |
| DEHYDRIN | MYCCONSENSUSAT | 566 | (+) | CANNTG | S000407 | Dehydratio/water stress |
| DEHYDRIN | MYCCONSENSUSAT | 759 | (-) | CANNTG | S000407 | Dehydratio/water stress |
| DEHYDRIN | MYCCONSENSUSAT | 759 | (+) | CANNTG | S000407 | Dehydratio/water stress |
| DEHYDRIN | MYCCONSENSUSAT | 773 | (-) | CANNTG | S000407 | Dehydratio/water stress |
| DEHYDRIN | MYCCONSENSUSAT | 773 | (+) | CANNTG | S000407 | Dehydratio/water stress |
| DEHYDRIN | MYCCONSENSUSAT | 824 | (-) | CANNTG | S000407 | Dehydratio/water stress |
| DEHYDRIN | MYCCONSENSUSAT | 824 | (+) | CANNTG | S000407 | Dehydratio/water stress |
| DEHYDRIN | MYCCONSENSUSAT | 840 | (-) | CANNTG | S000407 | Dehydratio/water stress |
| DEHYDRIN | MYCCONSENSUSAT | 840 | (+) | CANNTG | S000407 | Dehydratio/water stress |
| DEHYDRIN | MYCCONSENSUSAT | 60 | (-) | CANNTG | S000407 | Dehydratio/water stress |
| DEHYDRIN | MYCCONSENSUSAT | 60 | (+) | CANNTG | S000407 | Dehydratio/water stress |
| DEHYDRIN | MYCCONSENSUSAT | 92 | (-) | CANNTG | S000407 | Dehydratio/water stress |
| DEHYDRIN | MYCCONSENSUSAT | 92 | (+) | CANNTG | S000407 | Dehydratio/water stress |
| DEHYDRIN | MYCCONSENSUSAT | 110 | (-) | CANNTG | S000407 | Dehydratio/water stress |
| DEHYDRIN | MYCCONSENSUSAT | 110 | (+) | CANNTG | S000407 | Dehydratio/water stress |
| DEHYDRIN | MYCCONSENSUSAT | 363 | (-) | CANNTG | S000407 | Dehydratio/water stress |
| DEHYDRIN | MYCCONSENSUSAT | 363 | (+) | CANNTG | S000407 | Dehydratio/water stress |
| DEHYDRIN | MYCCONSENSUSAT | 519 | (-) | CANNTG | S000407 | Dehydratio/water stress |
| DEHYDRIN | MYCCONSENSUSAT | 519 | (+) | CANNTG | S000407 | Dehydratio/water stress |
| DEHYDRIN | MYCCONSENSUSAT | 677 | (-) | CANNTG | S000407 | Dehydratio/water stress |
| DEHYDRIN | MYCCONSENSUSAT | 677 | (+) | CANNTG | S000407 | Dehydratio/water stress |
| DEHYDRIN | MYCCONSENSUSAT | 739 | (-) | CANNTG | S000407 | Dehydratio/water stress |
| DEHYDRIN | MYCCONSENSUSAT | 739 | (+) | CANNTG | S000407 | Dehydratio/water stress |
| DEHYDRIN | MYCCONSENSUSAT | 512 | (-) | CANNTG | S000407 | Dehydratio/water stress |
| DEHYDRIN | MYCCONSENSUSAT | 512 | (+) | CANNTG | S000407 | Dehydratio/water stress |
| DEHYDRIN | MYCCONSENSUSAT | 250 | (-) | CANNTG | S000407 | Dehydratio/water stress |
| DEHYDRIN | MYCCONSENSUSAT | 250 | (+) | CANNTG | S000407 | Dehydratio/water stress |
| DEHYDRIN | MYCCONSENSUSAT | 400 | (-) | CANNTG | S000407 | Dehydratio/water stress |
| DEHYDRIN | MYCCONSENSUSAT | 400 | (+) | CANNTG | S000407 | Dehydratio/water stress |
| DEHYDRIN | MYCCONSENSUSAT | 421 | (-) | CANNTG | S000407 | Dehydratio/water stress |
| DEHYDRIN | MYCCONSENSUSAT | 421 | (+) | CANNTG | S000407 | Dehydratio/water stress |
| DEHYDRIN | MYCCONSENSUSAT | 667 | (-) | CANNTG | S000407 | Dehydratio/water stress |
| DEHYDRIN | MYCCONSENSUSAT | 667 | (+) | CANNTG | S000407 | Dehydratio/water stress |
| DEHYDRIN | MYCCONSENSUSAT | 28 | (-) | CANNTG | S000407 | Dehydratio/water stress |
| DEHYDRIN | MYCCONSENSUSAT | 28 | (+) | CANNTG | S000407 | Dehydratio/water stress |
| DEHYDRIN | MYCCONSENSUSAT | 437 | (-) | CANNTG | S000407 | Dehydratio/water stress |
| DEHYDRIN | MYCCONSENSUSAT | 437 | (+) | CANNTG | S000407 | Dehydratio/water stress |
| DEHYDRIN | MYCCONSENSUSAT | 475 | (-) | CANNTG | S000407 | Dehydratio/water stress |
| DEHYDRIN | MYCCONSENSUSAT | 475 | (+) | CANNTG | S000407 | Dehydratio/water stress |
| DEHYDRIN | MYCCONSENSUSAT | 95 | (-) | CANNTG | S000407 | Dehydratio/water stress |
| DEHYDRIN | MYCCONSENSUSAT | 95 | (+) | CANNTG | S000407 | Dehydratio/water stress |
| DEHYDRIN | MYCCONSENSUSAT | 566 | (-) | CANNTG | S000407 | Dehydratio/water stress |
| DEHYDRIN | MYCCONSENSUSAT | 566 | (+) | CANNTG | S000407 | Dehydratio/water stress |
| DEHYDRIN | MYCCONSENSUSAT | 466 | (-) | CANNTG | S000407 | Dehydratio/water stress |
| DEHYDRIN | MYCCONSENSUSAT | 466 | (+) | CANNTG | S000407 | Dehydratio/water stress |
| DEHYDRIN | MYCCONSENSUSAT | 318 | (-) | CANNTG | S000407 | Dehydratio/water stress |
| DEHYDRIN | MYCCONSENSUSAT | 318 | (+) | CANNTG | S000407 | Dehydratio/water stress |
| DEHYDRIN | MYCCONSENSUSAT | 81 | (-) | CANNTG | S000407 | Dehydratio/water stress |
| DEHYDRIN | MYCCONSENSUSAT | 81 | (+) | CANNTG | S000407 | Dehydratio/water stress |
| DEHYDRIN | MYCCONSENSUSAT | 278 | (-) | CANNTG | S000407 | Dehydratio/water stress |
| DEHYDRIN | MYCCONSENSUSAT | 278 | (+) | CANNTG | S000407 | Dehydratio/water stress |
| DEHYDRIN | MYCCONSENSUSAT | 337 | (-) | CANNTG | S000407 | Dehydratio/water stress |
| DEHYDRIN | MYCCONSENSUSAT | 337 | (+) | CANNTG | S000407 | Dehydratio/water stress |
| DEHYDRIN | MYCCONSENSUSAT | 439 | (-) | CANNTG | S000407 | Dehydratio/water stress |
| DEHYDRIN | MYCCONSENSUSAT | 439 | (+) | CANNTG | S000407 | Dehydratio/water stress |
| DEHYDRIN | MYCCONSENSUSAT | 615 | (-) | CANNTG | S000407 | Dehydratio/water stress |
| DEHYDRIN | MYCCONSENSUSAT | 615 | (+) | CANNTG | S000407 | Dehydratio/water stress |
| DEHYDRIN | MYCCONSENSUSAT | 32 | (-) | CANNTG | S000407 | Dehydratio/water stress |
| DEHYDRIN | MYCCONSENSUSAT | 32 | (+) | CANNTG | S000407 | Dehydratio/water stress |
| DEHYDRIN | MYCCONSENSUSAT | 38 | (-) | CANNTG | S000407 | Dehydratio/water stress |
| DEHYDRIN | MYCCONSENSUSAT | 38 | (+) | CANNTG | S000407 | Dehydratio/water stress |
| DEHYDRIN | MYCCONSENSUSAT | 206 | (-) | CANNTG | S000407 | Dehydratio/water stress |
| DEHYDRIN | MYCCONSENSUSAT | 206 | (+) | CANNTG | S000407 | Dehydratio/water stress |
| DEHYDRIN | MYCCONSENSUSAT | 526 | (-) | CANNTG | S000407 | Dehydratio/water stress |
| DEHYDRIN | MYCCONSENSUSAT | 526 | (+) | CANNTG | S000407 | Dehydratio/water stress |
| DEHYDRIN | MYCCONSENSUSAT | 103 | (-) | CANNTG | S000407 | Dehydratio/water stress |
| DEHYDRIN | MYCCONSENSUSAT | 103 | (+) | CANNTG | S000407 | Dehydratio/water stress |
| DEHYDRIN | MYCCONSENSUSAT | 283 | (-) | CANNTG | S000407 | Dehydratio/water stress |
| DEHYDRIN | MYCCONSENSUSAT | 283 | (+) | CANNTG | S000407 | Dehydratio/water stress |
| DEHYDRIN | MYCCONSENSUSAT | 603 | (-) | CANNTG | S000407 | Dehydratio/water stress |
| DEHYDRIN | MYCCONSENSUSAT | 603 | (+) | CANNTG | S000407 | Dehydratio/water stress |
| DEHYDRIN | MYCCONSENSUSAT | 29 | (-) | CANNTG | S000407 | Dehydratio/water stress |
| DEHYDRIN | MYCCONSENSUSAT | 29 | (+) | CANNTG | S000407 | Dehydratio/water stress |
| DEHYDRIN | MYCCONSENSUSAT | 160 | (-) | CANNTG | S000407 | Dehydratio/water stress |
| DEHYDRIN | MYCCONSENSUSAT | 160 | (+) | CANNTG | S000407 | Dehydratio/water stress |
| DEHYDRIN | MYCCONSENSUSAT | 503 | (-) | CANNTG | S000407 | Dehydratio/water stress |
| DEHYDRIN | MYCCONSENSUSAT | 503 | (+) | CANNTG | S000407 | Dehydratio/water stress |
| DEHYDRIN | MYCCONSENSUSAT | 557 | (-) | CANNTG | S000407 | Dehydratio/water stress |
| DEHYDRIN | MYCCONSENSUSAT | 557 | (+) | CANNTG | S000407 | Dehydratio/water stress |
| DEHYDRIN | MYCCONSENSUSAT | 640 | (-) | CANNTG | S000407 | Dehydratio/water stress |
| DEHYDRIN | MYCCONSENSUSAT | 640 | (+) | CANNTG | S000407 | Dehydratio/water stress |
| DEHYDRIN | MYCCONSENSUSAT | 146 | (-) | CANNTG | S000407 | Dehydratio/water stress |
| DEHYDRIN | MYCCONSENSUSAT | 146 | (+) | CANNTG | S000407 | Dehydratio/water stress |
| DEHYDRIN | MYCCONSENSUSAT | 157 | (-) | CANNTG | S000407 | Dehydratio/water stress |
| DEHYDRIN | MYCCONSENSUSAT | 157 | (+) | CANNTG | S000407 | Dehydratio/water stress |
| DEHYDRIN | MYCCONSENSUSAT | 385 | (-) | CANNTG | S000407 | Dehydratio/water stress |
| DEHYDRIN | MYCCONSENSUSAT | 385 | (+) | CANNTG | S000407 | Dehydratio/water stress |
| DEHYDRIN | MYCCONSENSUSAT | 716 | (-) | CANNTG | S000407 | Dehydratio/water stress |
| DEHYDRIN | MYCCONSENSUSAT | 716 | (+) | CANNTG | S000407 | Dehydratio/water stress |
| DEHYDRIN | MYCCONSENSUSAT | 761 | (-) | CANNTG | S000407 | Dehydratio/water stress |
| DEHYDRIN | MYCCONSENSUSAT | 761 | (+) | CANNTG | S000407 | Dehydratio/water stress |
| DEHYDRIN | MYCCONSENSUSAT | 158 | (-) | CANNTG | S000407 | Dehydratio/water stress |
| DEHYDRIN | MYCCONSENSUSAT | 158 | (+) | CANNTG | S000407 | Dehydratio/water stress |
| DEHYDRIN | MYCCONSENSUSAT | 317 | (-) | CANNTG | S000407 | Dehydratio/water stress |
| DEHYDRIN | MYCCONSENSUSAT | 317 | (+) | CANNTG | S000407 | Dehydratio/water stress |
| DEHYDRIN | MYCCONSENSUSAT | 398 | (-) | CANNTG | S000407 | Dehydratio/water stress |
| DEHYDRIN | MYCCONSENSUSAT | 398 | (+) | CANNTG | S000407 | Dehydratio/water stress |
| DEHYDRIN | MYCCONSENSUSAT | 595 | (-) | CANNTG | S000407 | Dehydratio/water stress |
| DEHYDRIN | MYCCONSENSUSAT | 595 | (+) | CANNTG | S000407 | Dehydratio/water stress |
| DEHYDRIN | MYCCONSENSUSAT | 196 | (-) | CANNTG | S000407 | Dehydratio/water stress |
| DEHYDRIN | MYCCONSENSUSAT | 196 | (+) | CANNTG | S000407 | Dehydratio/water stress |
| DEHYDRIN | MYCCONSENSUSAT | 284 | (-) | CANNTG | S000407 | Dehydratio/water stress |
| DEHYDRIN | MYCCONSENSUSAT | 284 | (+) | CANNTG | S000407 | Dehydratio/water stress |
| DEHYDRIN | MYCCONSENSUSAT | 563 | (-) | CANNTG | S000407 | Dehydratio/water stress |
| DEHYDRIN | MYCCONSENSUSAT | 563 | (+) | CANNTG | S000407 | Dehydratio/water stress |
| DEHYDRIN | MYCCONSENSUSAT | 722 | (-) | CANNTG | S000407 | Dehydratio/water stress |
| DEHYDRIN | MYCCONSENSUSAT | 722 | (+) | CANNTG | S000407 | Dehydratio/water stress |
| DEHYDRIN | MYCCONSENSUSAT | 856 | (-) | CANNTG | S000407 | Dehydratio/water stress |
| DEHYDRIN | MYCCONSENSUSAT | 856 | (+) | CANNTG | S000407 | Dehydratio/water stress |
| DEHYDRIN | MYCCONSENSUSAT | 902 | (-) | CANNTG | S000407 | Dehydratio/water stress |
| DEHYDRIN | MYCCONSENSUSAT | 902 | (+) | CANNTG | S000407 | Dehydratio/water stress |
| DEHYDRIN | MYCCONSENSUSAT | 134 | (-) | CANNTG | S000407 | Dehydratio/water stress |
| DEHYDRIN | MYCCONSENSUSAT | 134 | (+) | CANNTG | S000407 | Dehydratio/water stress |
| DEHYDRIN | MYCCONSENSUSAT | 158 | (-) | CANNTG | S000407 | Dehydratio/water stress |
| DEHYDRIN | MYCCONSENSUSAT | 158 | (+) | CANNTG | S000407 | Dehydratio/water stress |
| DEHYDRIN | MYCCONSENSUSAT | 317 | (-) | CANNTG | S000407 | Dehydratio/water stress |
| DEHYDRIN | MYCCONSENSUSAT | 317 | (+) | CANNTG | S000407 | Dehydratio/water stress |
| DEHYDRIN | MYCCONSENSUSAT | 213 | (-) | CANNTG | S000407 | Dehydratio/water stress |
| DEHYDRIN | MYCCONSENSUSAT | 213 | (+) | CANNTG | S000407 | Dehydratio/water stress |
| DEHYDRIN | MYCCONSENSUSAT | 273 | (-) | CANNTG | S000407 | Dehydratio/water stress |
| DEHYDRIN | MYCCONSENSUSAT | 273 | (+) | CANNTG | S000407 | Dehydratio/water stress |
| DEHYDRIN | MYCCONSENSUSAT | 328 | (-) | CANNTG | S000407 | Dehydratio/water stress |
| DEHYDRIN | MYCCONSENSUSAT | 328 | (+) | CANNTG | S000407 | Dehydratio/water stress |
| DEHYDRIN | MYCCONSENSUSAT | 49 | (-) | CANNTG | S000407 | Dehydratio/water stress |
| DEHYDRIN | MYCCONSENSUSAT | 49 | (+) | CANNTG | S000407 | Dehydratio/water stress |
| DEHYDRIN | MYCCONSENSUSAT | 82 | (-) | CANNTG | S000407 | Dehydratio/water stress |
| DEHYDRIN | MYCCONSENSUSAT | 82 | (+) | CANNTG | S000407 | Dehydratio/water stress |
| DEHYDRIN | MYCCONSENSUSAT | 214 | (-) | CANNTG | S000407 | Dehydratio/water stress |
| DEHYDRIN | MYCCONSENSUSAT | 214 | (+) | CANNTG | S000407 | Dehydratio/water stress |
| DEHYDRIN | MYCCONSENSUSAT | 247 | (-) | CANNTG | S000407 | Dehydratio/water stress |
| DEHYDRIN | MYCCONSENSUSAT | 247 | (+) | CANNTG | S000407 | Dehydratio/water stress |
| DEHYDRIN | MYCCONSENSUSAT | 343 | (-) | CANNTG | S000407 | Dehydratio/water stress |
| DEHYDRIN | MYCCONSENSUSAT | 343 | (+) | CANNTG | S000407 | Dehydratio/water stress |
| DEHYDRIN | MYCCONSENSUSAT | 625 | (-) | CANNTG | S000407 | Dehydratio/water stress |
| DEHYDRIN | MYCCONSENSUSAT | 625 | (+) | CANNTG | S000407 | Dehydratio/water stress |
| DEHYDRIN | MYCCONSENSUSAT | 121 | (-) | CANNTG | S000407 | Dehydratio/water stress |
| DEHYDRIN | MYCCONSENSUSAT | 121 | (+) | CANNTG | S000407 | Dehydratio/water stress |
| DEHYDRIN | MYCCONSENSUSAT | 264 | (-) | CANNTG | S000407 | Dehydratio/water stress |
| DEHYDRIN | MYCCONSENSUSAT | 264 | (+) | CANNTG | S000407 | Dehydratio/water stress |
| LEA 1 | ABRELATERD1 | 244 | (-) | ACGTG | S000414 | ABA responsive elements |
| LEA 1 | ABRELATERD1 | 447 | (-) | ACGTG | S000414 | ABA responsive elements |
| LEA 1 | ABRELATERD1 | 569 | (+) | ACGTG | S000414 | ABA responsive elements |
| LEA 1 | ABRELATERD1 | 108 | (-) | ACGTG | S000414 | ABA responsive elements |
| LEA 1 | ABRELATERD1 | 41 | (-) | ACGTG | S000414 | ABA responsive elements |
| LEA 1 | ABRELATERD1 | 232 | (-) | ACGTG | S000414 | ABA responsive elements |
| LEA 1 | ABRELATERD1 | 246 | (-) | ACGTG | S000414 | ABA responsive elements |
| LEA 1 | ABRELATERD1 | 419 | (+) | ACGTG | S000414 | ABA responsive elements |
| LEA 1 | ABREOSRAB21 | 247 | (+) | ACGTSSSC | S000012 | ABA responsive elements |
| LEA 1 | ABRERATCAL | 568 | (+) | MACGYGB | S000507 | ABA responsive elements |
| LEA 1 | ACGTABREMOTIFA2OSEM | 569 | (+) | ACGTGKC | S000394 | early responsive to dehydration |
| LEA 1 | ASF1MOTIFCAMV | 159 | (-) | TGACG | S000024 | Abiotic and biotic stress |
| LEA 1 | ASF1MOTIFCAMV | 165 | (-) | TGACG | S000024 | Abiotic and biotic stress |
| LEA 1 | ASF1MOTIFCAMV | 105 | (-) | TGACG | S000024 | Abiotic and biotic stress |
| LEA 1 | ASF1MOTIFCAMV | 288 | (+) | TGACG | S000024 | Abiotic and biotic stress |
| LEA 1 | ASF1MOTIFCAMV | 593 | (+) | TGACG | S000024 | Abiotic and biotic stress |
| LEA 1 | ASF1MOTIFCAMV | 276 | (-) | TGACG | S000024 | Abiotic and biotic stress |
| LEA 1 | ASF1MOTIFCAMV | 320 | (+) | TGACG | S000024 | Abiotic and biotic stress |
| LEA 1 | ASF1MOTIFCAMV | 356 | (+) | TGACG | S000024 | Abiotic and biotic stress |
| LEA 1 | ASF1MOTIFCAMV | 110 | (-) | TGACG | S000024 | Abiotic and biotic stress |
| LEA 1 | ASF1MOTIFCAMV | 190 | (-) | TGACG | S000024 | Abiotic and biotic stress |
| LEA 1 | ASF1MOTIFCAMV | 485 | (+) | TGACG | S000024 | Abiotic and biotic stress |
| LEA 1 | ASF1MOTIFCAMV | 440 | (+) | TGACG | S000024 | Abiotic and biotic stress |
| LEA 1 | ASF1MOTIFCAMV | 150 | (-) | TGACG | S000024 | Abiotic and biotic stress |
| LEA 1 | ASF1MOTIFCAMV | 622 | (-) | TGACG | S000024 | Abiotic and biotic stress |
| LEA 1 | CBFHV | 382 | (-) | RYCGAC | S000497 | dehydration responsive element |
| LEA 1 | CBFHV | 382 | (+) | RYCGAC | S000497 | dehydration responsive element |
| LEA 1 | CBFHV | 296 | (+) | RYCGAC | S000497 | dehydration responsive element |
| LEA 1 | CBFHV | 37 | (-) | RYCGAC | S000497 | dehydration responsive element |
| LEA 1 | CBFHV | 37 | (+) | RYCGAC | S000497 | dehydration responsive element |
| LEA 1 | CBFHV | 120 | (-) | RYCGAC | S000497 | dehydration responsive element |
| LEA 1 | CBFHV | 120 | (+) | RYCGAC | S000497 | dehydration responsive element |
| LEA 1 | CBFHV | 487 | (+) | RYCGAC | S000497 | dehydration responsive element |
| LEA 1 | CBFHV | 26 | (-) | RYCGAC | S000497 | dehydration responsive element |
| LEA 1 | CBFHV | 414 | (-) | RYCGAC | S000497 | dehydration responsive element |
| LEA 1 | CBFHV | 414 | (+) | RYCGAC | S000497 | dehydration responsive element |
| LEA 1 | CBFHV | 81 | (+) | RYCGAC | S000497 | dehydration responsive element |
| LEA 1 | LTRE1HVBLT49 | 35 | (-) | CCGAAA | S000250 | necessary for coldor drought |
| LEA 1 | LTRE1HVBLT49 | 215 | (-) | CCGAAA | S000250 | necessary for coldor drought |
| LEA 1 | LTRE1HVBLT49 | 611 | (+) | CCGAAA | S000250 | necessary for coldor drought |
| LEA 1 | LTRE1HVBLT49 | 287 | (-) | CCGAAA | S000250 | necessary for coldor drought |
| LEA 1 | LTRE1HVBLT49 | 341 | (-) | CCGAAA | S000250 | necessary for coldor drought |
| LEA 1 | LTRE1HVBLT49 | 479 | (-) | CCGAAA | S000250 | necessary for coldor drought |
| LEA 1 | MYB1AT | 227 | (+) | WAACCA | S000408 | MYB recognition site |
| LEA 1 | MYB1AT | 296 | (-) | WAACCA | S000408 | MYB recognition site |
| LEA 1 | MYB1AT | 395 | (+) | WAACCA | S000408 | MYB recognition site |
| LEA 1 | MYB1AT | 851 | (-) | WAACCA | S000408 | MYB recognition site |
| LEA 1 | MYB1AT | 13 | (-) | WAACCA | S000408 | MYB recognition site |
| LEA 1 | MYB1AT | 60 | (+) | WAACCA | S000408 | MYB recognition site |
| LEA 1 | MYB1AT | 606 | (+) | WAACCA | S000408 | MYB recognition site |
| LEA 1 | MYB1AT | 694 | (-) | WAACCA | S000408 | MYB recognition site |
| LEA 1 | MYB2AT | 515 | (-) | TAACTG | S000177 | MYB recognition site |
| LEA 1 | MYB2AT | 687 | (+) | TAACTG | S000177 | MYB recognition site |
| LEA 1 | MYB2AT | 38 | (+) | TAACTG | S000177 | MYB recognition site |
| LEA 1 | MYB2CONSENSUSAT | 515 | (-) | YAACKG | S000409 | Dehydratio/water stress |
| LEA 1 | MYB2CONSENSUSAT | 544 | (-) | YAACKG | S000409 | Dehydratio/water stress |
| LEA 1 | MYB2CONSENSUSAT | 345 | (+) | YAACKG | S000409 | Dehydratio/water stress |
| LEA 1 | MYB2CONSENSUSAT | 635 | (-) | YAACKG | S000409 | Dehydratio/water stress |
| LEA 1 | MYB2CONSENSUSAT | 687 | (+) | YAACKG | S000409 | Dehydratio/water stress |
| LEA 1 | MYB2CONSENSUSAT | 60 | (+) | YAACKG | S000409 | Dehydratio/water stress |
| LEA 1 | MYB2CONSENSUSAT | 99 | (+) | YAACKG | S000409 | Dehydratio/water stress |
| LEA 1 | MYB2CONSENSUSAT | 492 | (+) | YAACKG | S000409 | Dehydratio/water stress |
| LEA 1 | MYB2CONSENSUSAT | 620 | (-) | YAACKG | S000409 | Dehydratio/water stress |
| LEA 1 | MYB2CONSENSUSAT | 634 | (-) | YAACKG | S000409 | Dehydratio/water stress |
| LEA 1 | MYB2CONSENSUSAT | 384 | (+) | YAACKG | S000409 | Dehydratio/water stress |
| LEA 1 | MYB2CONSENSUSAT | 38 | (+) | YAACKG | S000409 | Dehydratio/water stress |
| LEA 1 | MYB2CONSENSUSAT | 117 | (-) | YAACKG | S000409 | Dehydratio/water stress |
| LEA 1 | MYBCORE | 365 | (+) | CNGTTR | S000176 | Dehydratio/water stress |
| LEA 1 | MYBCORE | 515 | (+) | CNGTTR | S000176 | Dehydratio/water stress |
| LEA 1 | MYBCORE | 544 | (+) | CNGTTR | S000176 | Dehydratio/water stress |
| LEA 1 | MYBCORE | 122 | (+) | CNGTTR | S000176 | Dehydratio/water stress |
| LEA 1 | MYBCORE | 221 | (+) | CNGTTR | S000176 | Dehydratio/water stress |
| LEA 1 | MYBCORE | 622 | (-) | CNGTTR | S000176 | Dehydratio/water stress |
| LEA 1 | MYBCORE | 345 | (-) | CNGTTR | S000176 | Dehydratio/water stress |
| LEA 1 | MYBCORE | 635 | (+) | CNGTTR | S000176 | Dehydratio/water stress |
| LEA 1 | MYBCORE | 687 | (-) | CNGTTR | S000176 | Dehydratio/water stress |
| LEA 1 | MYBCORE | 60 | (-) | CNGTTR | S000176 | Dehydratio/water stress |
| LEA 1 | MYBCORE | 92 | (-) | CNGTTR | S000176 | Dehydratio/water stress |
| LEA 1 | MYBCORE | 99 | (-) | CNGTTR | S000176 | Dehydratio/water stress |
| LEA 1 | MYBCORE | 471 | (-) | CNGTTR | S000176 | Dehydratio/water stress |
| LEA 1 | MYBCORE | 492 | (-) | CNGTTR | S000176 | Dehydratio/water stress |
| LEA 1 | MYBCORE | 519 | (-) | CNGTTR | S000176 | Dehydratio/water stress |
| LEA 1 | MYBCORE | 443 | (+) | CNGTTR | S000176 | Dehydratio/water stress |
| LEA 1 | MYBCORE | 620 | (+) | CNGTTR | S000176 | Dehydratio/water stress |
| LEA 1 | MYBCORE | 634 | (+) | CNGTTR | S000176 | Dehydratio/water stress |
| LEA 1 | MYBCORE | 62 | (-) | CNGTTR | S000176 | Dehydratio/water stress |
| LEA 1 | MYBCORE | 113 | (+) | CNGTTR | S000176 | Dehydratio/water stress |
| LEA 1 | MYBCORE | 384 | (-) | CNGTTR | S000176 | Dehydratio/water stress |
| LEA 1 | MYBCORE | 38 | (-) | CNGTTR | S000176 | Dehydratio/water stress |
| LEA 1 | MYBCORE | 41 | (+) | CNGTTR | S000176 | Dehydratio/water stress |
| LEA 1 | MYBCORE | 117 | (+) | CNGTTR | S000176 | Dehydratio/water stress |
| LEA 1 | MYBCORE | 300 | (-) | CNGTTR | S000176 | Dehydratio/water stress |
| LEA 1 | MYBCORE | 344 | (+) | CNGTTR | S000176 | Dehydratio/water stress |
| LEA 1 | MYBCOREATCYCB1 | 211 | (+) | AACGG | S000502 | Dehydratio/water stress |
| LEA 1 | MYBCOREATCYCB1 | 203 | (-) | AACGG | S000502 | Dehydratio/water stress |
| LEA 1 | MYBCOREATCYCB1 | 346 | (+) | AACGG | S000502 | Dehydratio/water stress |
| LEA 1 | MYBCOREATCYCB1 | 635 | (-) | AACGG | S000502 | Dehydratio/water stress |
| LEA 1 | MYBCOREATCYCB1 | 61 | (+) | AACGG | S000502 | Dehydratio/water stress |
| LEA 1 | MYBCOREATCYCB1 | 100 | (+) | AACGG | S000502 | Dehydratio/water stress |
| LEA 1 | MYBCOREATCYCB1 | 493 | (+) | AACGG | S000502 | Dehydratio/water stress |
| LEA 1 | MYBCOREATCYCB1 | 385 | (+) | AACGG | S000502 | Dehydratio/water stress |
| LEA 1 | MYBCOREATCYCB1 | 754 | (+) | AACGG | S000502 | Dehydratio/water stress |
| LEA 1 | MYBCOREATCYCB1 | 117 | (-) | AACGG | S000502 | Dehydratio/water stress |
| LEA 1 | MYBGAHV | 658 | (-) | TAACAAA | S000181 | MYB recognition site |
| LEA 1 | MYBPZM | 299 | (+) | CCWACC | S000179 | MYB recognition site |
| LEA 1 | MYBPZM | 262 | (-) | CCWACC | S000179 | MYB recognition site |
| LEA 1 | MYBPZM | 98 | (+) | CCWACC | S000179 | MYB recognition site |
| LEA 1 | MYBPZM | 333 | (-) | CCWACC | S000179 | MYB recognition site |
| LEA 1 | MYBPZM | 471 | (-) | CCWACC | S000179 | MYB recognition site |
| LEA 1 | MYBPZM | 345 | (-) | CCWACC | S000179 | MYB recognition site |
| LEA 1 | MYBST1 | 127 | (+) | GGATA | S000180 | MYB responsive |
| LEA 1 | MYBST1 | 189 | (-) | GGATA | S000180 | MYB responsive |
| LEA 1 | MYBST1 | 43 | (+) | GGATA | S000180 | MYB responsive |
| LEA 1 | MYBST1 | 510 | (+) | GGATA | S000180 | MYB responsive |
| LEA 1 | MYBST1 | 165 | (+) | GGATA | S000180 | MYB responsive |
| LEA 1 | MYBST1 | 166 | (-) | GGATA | S000180 | MYB responsive |
| LEA 1 | MYBST1 | 222 | (-) | GGATA | S000180 | MYB responsive |
| LEA 1 | MYBST1 | 327 | (-) | GGATA | S000180 | MYB responsive |
| LEA 1 | MYBST1 | 648 | (+) | GGATA | S000180 | MYB responsive |
| LEA 1 | MYBST1 | 344 | (+) | GGATA | S000180 | MYB responsive |
| LEA 1 | MYBST1 | 592 | (+) | GGATA | S000180 | MYB responsive |
| LEA 1 | MYBST1 | 687 | (+) | GGATA | S000180 | MYB responsive |
| LEA 1 | MYBST1 | 597 | (+) | GGATA | S000180 | MYB responsive |
| LEA 1 | MYCATERD1 | 266 | (-) | CATGTG | S000413 | Dehydratio/water stress |
| LEA 1 | MYCATERD1 | 37 | (-) | CATGTG | S000413 | Dehydratio/water stress |
| LEA 1 | MYCATERD1 | 75 | (+) | CATGTG | S000413 | Dehydratio/water stress |
| LEA 1 | MYCATERD1 | 73 | (-) | CATGTG | S000413 | Dehydratio/water stress |
| LEA 1 | MYCATERD1 | 581 | (+) | CATGTG | S000413 | Dehydratio/water stress |
| LEA 1 | MYCATRD22 | 266 | (+) | CACATG | S000174 | Dehydratio/water stress |
| LEA 1 | MYCATRD22 | 37 | (+) | CACATG | S000174 | Dehydratio/water stress |
| LEA 1 | MYCATRD22 | 75 | (-) | CACATG | S000174 | Dehydratio/water stress |
| LEA 1 | MYCATRD22 | 73 | (+) | CACATG | S000174 | Dehydratio/water stress |
| LEA 1 | MYCATRD22 | 581 | (-) | CACATG | S000174 | Dehydratio/water stress |
| LEA 2 | LTRECOREATCOR15 | 728 | (-) | CCGAC | S000153 | necessary for coldor drought |
| LEA 2 | CBFHV | 3 | (-) | RYCGAC | S000497 | dehydration responsive element |
| LEA 2 | LTRECOREATCOR15 | 239 | (-) | CCGAC | S000153 | necessary for coldor drought |
| LEA 2 | MYBAT | 513 | (+) | TAACTG | S000177 | MYB recognition site |
| LEA 2 | ABREATCONSENSUS | 436 | (-) | YACGTGGC | S000406 | ABA responsive elements |
| LEA 2 | ABRECE1HVA | 673 | (+) | TGCCACCGG | S000014 | ABA responsive complex |
| LEA 2 | ABRECE1HVA | 673 | (+) | TGCCACCGG | S000014 | ABA responsive complex |
| LEA 2 | ABRELATERD1 | 110 | (-) | ACGTG | S000414 | ABA responsive complex |
| LEA 2 | ABRELATERD1 | 435 | (+) | ACGTG | S000414 | ABA responsive complex |
| LEA 2 | ABRELATERD1 | 697 | (-) | ACGTG | S000414 | ABA responsive complex |
| LEA 2 | ABRELATERD1 | 903 | (+) | ACGTG | S000414 | ABA responsive complex |
| LEA 2 | ABRELATERD1 | 1006 | (+) | ACGTG | S000414 | ABA responsive complex |
| LEA 2 | ACGTATERD1 | 194 | (+) | ACGT | S000415 | early responsive to dehydration |
| LEA 2 | ACGTATERD1 | 200 | (-) | ACGT | S000415 | early responsive to dehydration |
| LEA 2 | ACGTATERD1 | 200 | (+) | ACGT | S000415 | early responsive to dehydration |
| LEA 2 | ACGTATERD1 | 849 | (-) | ACGT | S000415 | early responsive to dehydration |
| LEA 2 | ACGTATERD1 | 849 | (+) | ACGT | S000415 | early responsive to dehydration |
| LEA 2 | ASF1MOTIFCAMV | 634 | (-) | TGACG | S000024 | Abiotic and biotic stress |
| LEA 2 | ASF1MOTIFCAMV | 110 | (+) | TGACG | S000024 | Abiotic and biotic stress |
| LEA 2 | ASF1MOTIFCAMV | 125 | (+) | TGACG | S000024 | Abiotic and biotic stress |
| LEA 2 | ASF1MOTIFCAMV | 140 | (-) | TGACG | S000024 | Abiotic and biotic stress |
| LEA 2 | ASF1MOTIFCAMV | 462 | (+) | TGACG | S000024 | Abiotic and biotic stress |
| LEA 2 | ASF1MOTIFCAMV | 199 | (-) | TGACG | S000024 | Abiotic and biotic stress |
| LEA 2 | ASF1MOTIFCAMV | 229 | (+) | TGACG | S000024 | Abiotic and biotic stress |
| LEA 2 | LTRECOREATCOR15 | 3 | (-) | CCGAC | S000153 | necessary for coldor drought |
| LEA 2 | LTRECOREATCOR15 | 449 | (-) | CCGAC | S000153 | necessary for coldor drought |
| LEA 2 | LTRECOREATCOR15 | 424 | (+) | CCGAC | S000153 | necessary for coldor drought |
| LEA 2 | LTRECOREATCOR15 | 630 | (-) | CCGAC | S000153 | necessary for coldor drought |
| LEA 2 | LTRECOREATCOR15 | 269 | (-) | CCGAC | S000153 | necessary for coldor drought |
| LEA 2 | LTRECOREATCOR15 | 350 | (+) | CCGAC | S000153 | necessary for coldor drought |
| LEA 2 | LTRECOREATCOR15 | 500 | (+) | CCGAC | S000153 | necessary for coldor drought |
| LEA 2 | LTRECOREATCOR15 | 849 | (+) | CCGAC | S000153 | necessary for coldor drought |
| LEA 2 | LTRECOREATCOR15 | 565 | (-) | CCGAC | S000153 | necessary for coldor drought |
| LEA 2 | LTRECOREATCOR15 | 175 | (-) | CCGAC | S000153 | necessary for coldor drought |
| LEA 2 | LTRECOREATCOR15 | 294 | (-) | CCGAC | S000153 | necessary for coldor drought |
| LEA 2 | LTRECOREATCOR15 | 335 | (-) | CCGAC | S000153 | necessary for coldor drought |
| LEA 2 | LTRECOREATCOR15 | 537 | (-) | CCGAC | S000153 | necessary for coldor drought |
| LEA 2 | MYBAT | 727 | (+) | TAACTG | S000177 | MYB recognition site |
| LEA 2 | MYBAT | 724 | (-) | TAACTG | S000177 | MYB recognition site |
| LEA 2 | MYBAT | 513 | (+) | TAACTG | S000177 | MYB recognition site |
| LEA 2 | MYBAT | 681 | (+) | TAACTG | S000177 | MYB recognition site |
| LEA 2 | MYBAT | 939 | (-) | TAACTG | S000177 | MYB recognition site |
| LEA 2 | MYBAT | 525 | (-) | TAACTG | S000177 | MYB recognition site |
| LEA 2 | MYBAT | 372 | (-) | TAACTG | S000177 | MYB recognition site |
| LEA 2 | MYBAT | 740 | (-) | TAACTG | S000177 | MYB recognition site |
| LEA 2 | MYBAT | 376 | (-) | TAACTG | S000177 | MYB recognition site |
| LEA 2 | MYBAT | 513 | (+) | TAACTG | S000177 | MYB recognition site |
| LEA 2 | MYBAT | 724 | (-) | TAACTG | S000177 | MYB recognition site |
| LEA 2 | MYBAT | 514 | (-) | TAACTG | S000177 | MYB recognition site |
| LEA 2 | MYBAT | 727 | (+) | TAACTG | S000177 | MYB recognition site |
| LEA 2 | MYBAT | 526 | (-) | TAACTG | S000177 | MYB recognition site |
| LEA 2 | MYBAT | 529 | (+) | TAACTG | S000177 | MYB recognition site |
| LEA 2 | MYBAT | 1315 | (-) | TAACTG | S000177 | MYB recognition site |
| LEA 2 | MYBAT | 185 | (+) | TAACTG | S000177 | MYB recognition site |
| LEA 2 | MYBAT | 372 | (-) | TAACTG | S000177 | MYB recognition site |
| LEA 2 | MYBAT | 372 | (-) | TAACTG | S000177 | MYB recognition site |
| LEA 2 | MYBAT | 740 | (-) | TAACTG | S000177 | MYB recognition site |
| LEA 2 | MYBATRD | 60 | (+) | CTAACCA | S000175 | MYB recognition site |
| LEA 2 | MYBATRD | 193 | (+) | CTAACCA | S000175 | MYB recognition site |
| LEA 2 | MYBATRD | 60 | (+) | CTAACCA | S000175 | MYB recognition site |
| LEA 2 | MYBATRD | 60 | (+) | CTAACCA | S000175 | MYB recognition site |
| LEA 2 | MYBATRD | 123 | (-) | CTAACCA | S000175 | MYB recognition site |
| LEA 2 | MYBATRD | 193 | (+) | CTAACCA | S000175 | MYB recognition site |
| LEA 2 | MYBATRD | 207 | (+) | CTAACCA | S000175 | MYB recognition site |
| LEA 2 | ABRELATERD1 | 248 | (-) | ACGTG | S000414 | ABA responsive complex |
| LEA 2 | LTRECOREATCOR15 | 11 | (+) | CCGAC | S000153 | necessary for coldor drought |
| LEA 2 | LTRECOREATCOR15 | 588 | (-) | CCGAC | S000153 | necessary for coldor drought |
| LEA 2 | LTRECOREATCOR15 | 273 | (-) | CCGAC | S000153 | necessary for coldor drought |
| LEA 2 | LTRECOREATCOR15 | 296 | (+) | CCGAC | S000153 | necessary for coldor drought |
| LEA 2 | MYBATRD | 1480 | (+) | CTAACCA | S000175 | MYB recognition site |
| LEA 2 | MYBATRD | 53 | (+) | CTAACCA | S000175 | MYB recognition site |
| LEA 2 | MYBATRD | 574 | (-) | CTAACCA | S000175 | MYB recognition site |
| LEA 2 | MYBATRD | 53 | (+) | CTAACCA | S000175 | MYB recognition site |
| LEA 2 | MYBATRD | 53 | (+) | CTAACCA | S000175 | MYB recognition site |
| LEA 2 | ABRELATERD1 | 580 | (+) | ACGTG | S000414 | ABA responsive complex |
| LEA 2 | CBFHV | 449 | (-) | RYCGAC | S000497 | dehydration responsive element |
| LEA 2 | CBFHV | 227 | (+) | RYCGAC | S000497 | dehydration responsive element |
| LEA 2 | CBFHV | 270 | (+) | RYCGAC | S000497 | dehydration responsive element |
| LEA 2 | CBFHV | 52 | (-) | RYCGAC | S000497 | dehydration responsive element |
| LEA 2 | LTRECOREATCOR15 | 3 | (-) | CCGAC | S000153 | necessary for coldor drought |
| LEA 2 | LTRECOREATCOR15 | 411 | (-) | CCGAC | S000153 | necessary for coldor drought |
| LEA 2 | LTRECOREATCOR15 | 802 | (+) | CCGAC | S000153 | necessary for coldor drought |
| LEA 2 | LTRECOREATCOR15 | 313 | (+) | CCGAC | S000153 | necessary for coldor drought |
| LEA 2 | LTRECOREATCOR15 | 225 | (-) | CCGAC | S000153 | necessary for coldor drought |
| LEA 2 | LTRECOREATCOR15 | 3 | (-) | CCGAC | S000153 | necessary for coldor drought |
| LEA 2 | LTRECOREATCOR15 | 588 | (-) | CCGAC | S000153 | necessary for coldor drought |
| LEA 2 | LTRECOREATCOR15 | 535 | (-) | CCGAC | S000153 | necessary for coldor drought |
| LEA 2 | MYBCONSENSUSAT | 61 | (+) | YAACKG | S000409 | MYB recognition site |
| LEA 2 | MYBCONSENSUSAT | 501 | (+) | YAACKG | S000409 | MYB recognition site |
| LEA 2 | MYBCONSENSUSAT | 632 | (-) | YAACKG | S000409 | MYB recognition site |
| LEA 2 | MYBCONSENSUSAT | 646 | (-) | YAACKG | S000409 | MYB recognition site |
| LEA 2 | MYBCONSENSUSAT | 149 | (+) | YAACKG | S000409 | MYB recognition site |
| LEA 2 | MYBCONSENSUSAT | 387 | (+) | YAACKG | S000409 | MYB recognition site |
| LEA 2 | MYBCONSENSUSAT | 588 | (-) | YAACKG | S000409 | MYB recognition site |
| LEA 2 | MYBCONSENSUSAT | 605 | (+) | YAACKG | S000409 | MYB recognition site |
| LEA 2 | MYBCONSENSUSAT | 626 | (-) | YAACKG | S000409 | MYB recognition site |
| LEA 2 | LTRECOREATCOR15 | 3 | (-) | CCGAC | S000153 | necessary for coldor drought |
| LEA 2 | LTRECOREATCOR15 | 130 | (+) | CCGAC | S000153 | necessary for coldor drought |
| LEA 2 | ABRELATERD1 | 409 | (+) | ACGTG | S000414 | ABA responsive complex |
| LEA 2 | LTRECOREATCOR15 | 513 | (-) | CCGAC | S000153 | necessary for coldor drought |
| LEA 2 | LTRECOREATCOR15 | 3 | (-) | CCGAC | S000153 | necessary for coldor drought |
| LEA 2 | MYBCONSENSUSAT | 688 | (-) | YAACKG | S000409 | MYB recognition site |
| LEA 2 | MYBCONSENSUSAT | 525 | (-) | YAACKG | S000409 | MYB recognition site |
| LEA 2 | ACGTATERD1 | 97 | (-) | ACGT | S000415 | early responsive to dehydration |
| LEA 2 | ACGTATERD1 | 97 | (+) | ACGT | S000415 | early responsive to dehydration |
| LEA 2 | ACGTATERD1 | 370 | (-) | ACGT | S000415 | early responsive to dehydration |
| LEA 2 | ACGTATERD1 | 370 | (+) | ACGT | S000415 | early responsive to dehydration |
| LEA 2 | ACGTATERD1 | 913 | (-) | ACGT | S000415 | early responsive to dehydration |
| LEA 2 | ACGTATERD1 | 913 | (+) | ACGT | S000415 | early responsive to dehydration |
| LEA 2 | ACGTATERD1 | 552 | (-) | ACGT | S000415 | early responsive to dehydration |
| LEA 2 | ACGTATERD1 | 552 | (+) | ACGT | S000415 | early responsive to dehydration |
| LEA 2 | AGCBOXNPGLB | 426 | (+) | AGCCGCC | S000232 | stress signal response factors |
| LEA 2 | AGCBOXNPGLB | 205 | (+) | AGCCGCC | S000232 | stress signal response factors |
| LEA 2 | AGCBOXNPGLB | 27 | (+) | AGCCGCC | S000232 | stress signal response factors |
| LEA 2 | AGCBOXNPGLB | 525 | (-) | AGCCGCC | S000232 | stress signal response factors |
| LEA 2 | AGCBOXNPGLB | 17 | (+) | AGCCGCC | S000232 | stress signal response factors |
| LEA 2 | AGCBOXNPGLB | 226 | (+) | AGCCGCC | S000232 | stress signal response factors |
| LEA 2 | AGCBOXNPGLB | 205 | (+) | AGCCGCC | S000232 | stress signal response factors |
| LEA 2 | AGCBOXNPGLB | 205 | (+) | AGCCGCC | S000232 | stress signal response factors |
| LEA 2 | AGCBOXNPGLB | 525 | (-) | AGCCGCC | S000232 | stress signal response factors |
| LEA 2 | AGMOTIFNTMYB | 525 | (+) | AGATCCAA | S000444 | induced by various stress |
| LEA 2 | AGMOTIFNTMYB | 600 | (-) | AGATCCAA | S000444 | induced by various stress |
| LEA 2 | AGMOTIFNTMYB | 525 | (+) | AGATCCAA | S000444 | induced by various stress |
| LEA 2 | ASF1MOTIFCAMV | 630 | (-) | TGACG | S000024 | Abiotic and biotic stress |
| LEA 2 | ASF1MOTIFCAMV | 267 | (-) | TGACG | S000024 | Abiotic and biotic stress |
| LEA 2 | ASF1MOTIFCAMV | 697 | (-) | TGACG | S000024 | Abiotic and biotic stress |
| LEA 2 | ASF1MOTIFCAMV | 361 | (+) | TGACG | S000024 | Abiotic and biotic stress |
| LEA 2 | ASF1MOTIFCAMV | 1027 | (+) | TGACG | S000024 | Abiotic and biotic stress |
| LEA 2 | ASF1MOTIFCAMV | 241 | (+) | TGACG | S000024 | Abiotic and biotic stress |
| LEA 2 | ASF1MOTIFCAMV | 143 | (-) | TGACG | S000024 | Abiotic and biotic stress |
| LEA 2 | ASF1MOTIFCAMV | 189 | (-) | TGACG | S000024 | Abiotic and biotic stress |
| LEA 2 | ASF1MOTIFCAMV | 214 | (-) | TGACG | S000024 | Abiotic and biotic stress |
| LEA 2 | ASF1MOTIFCAMV | 136 | (+) | TGACG | S000024 | Abiotic and biotic stress |
| LEA 2 | ASF1MOTIFCAMV | 55 | (-) | TGACG | S000024 | Abiotic and biotic stress |
| LEA 2 | ASF1MOTIFCAMV | 664 | (-) | TGACG | S000024 | Abiotic and biotic stress |
| LEA 2 | ASF1MOTIFCAMV | 440 | (+) | TGACG | S000024 | Abiotic and biotic stress |
| LEA 2 | ASF1MOTIFCAMV | 586 | (+) | TGACG | S000024 | Abiotic and biotic stress |
| LEA 2 | ASF1MOTIFCAMV | 614 | (+) | TGACG | S000024 | Abiotic and biotic stress |
| LEA 2 | ASF1MOTIFCAMV | 565 | (+) | TGACG | S000024 | Abiotic and biotic stress |
| LEA 2 | ASF1MOTIFCAMV | 197 | (-) | TGACG | S000024 | Abiotic and biotic stress |
| LEA 2 | ASF1MOTIFCAMV | 617 | (+) | TGACG | S000024 | Abiotic and biotic stress |
| LEA 2 | ASF1MOTIFCAMV | 351 | (-) | TGACG | S000024 | Abiotic and biotic stress |
| LEA 2 | ASF1MOTIFCAMV | 283 | (-) | TGACG | S000024 | Abiotic and biotic stress |
| LEA 2 | ASF1MOTIFCAMV | 911 | (+) | TGACG | S000024 | Abiotic and biotic stress |
| LEA 2 | ASF1MOTIFCAMV | 41 | (-) | TGACG | S000024 | Abiotic and biotic stress |
| LEA 2 | ASF1MOTIFCAMV | 104 | (+) | TGACG | S000024 | Abiotic and biotic stress |
| LEA 2 | ASF1MOTIFCAMV | 284 | (+) | TGACG | S000024 | Abiotic and biotic stress |
| LEA 2 | ASF1MOTIFCAMV | 687 | (+) | TGACG | S000024 | Abiotic and biotic stress |
| LEA 2 | ASF1MOTIFCAMV | 24 | (-) | TGACG | S000024 | Abiotic and biotic stress |
| LEA 2 | ASF1MOTIFCAMV | 33 | (-) | TGACG | S000024 | Abiotic and biotic stress |
| LEA 2 | ASF1MOTIFCAMV | 79 | (+) | TGACG | S000024 | Abiotic and biotic stress |
| LEA 2 | ASF1MOTIFCAMV | 195 | (-) | TGACG | S000024 | Abiotic and biotic stress |
| LEA 2 | ASF1MOTIFCAMV | 311 | (-) | TGACG | S000024 | Abiotic and biotic stress |
| LEA 2 | ASF1MOTIFCAMV | 348 | (-) | TGACG | S000024 | Abiotic and biotic stress |
| LEA 2 | ASF1MOTIFCAMV | 324 | (+) | TGACG | S000024 | Abiotic and biotic stress |
| LEA 2 | ASF1MOTIFCAMV | 30 | (+) | TGACG | S000024 | Abiotic and biotic stress |
| LEA 2 | ASF1MOTIFCAMV | 627 | (+) | TGACG | S000024 | Abiotic and biotic stress |
| LEA 2 | ASF1MOTIFCAMV | 363 | (+) | TGACG | S000024 | Abiotic and biotic stress |
| LEA 2 | ASF1MOTIFCAMV | 494 | (+) | TGACG | S000024 | Abiotic and biotic stress |
| LEA 2 | ASF1MOTIFCAMV | 605 | (-) | TGACG | S000024 | Abiotic and biotic stress |
| LEA 2 | ASF1MOTIFCAMV | 41 | (-) | TGACG | S000024 | Abiotic and biotic stress |
| LEA 2 | ASF1MOTIFCAMV | 104 | (+) | TGACG | S000024 | Abiotic and biotic stress |
| LEA 2 | ASF1MOTIFCAMV | 284 | (+) | TGACG | S000024 | Abiotic and biotic stress |
| LEA 2 | ASF1MOTIFCAMV | 687 | (+) | TGACG | S000024 | Abiotic and biotic stress |
| LEA 2 | ASF1MOTIFCAMV | 162 | (-) | TGACG | S000024 | Abiotic and biotic stress |
| LEA 2 | ASF1MOTIFCAMV | 159 | (-) | TGACG | S000024 | Abiotic and biotic stress |
| LEA 2 | ASF1MOTIFCAMV | 208 | (-) | TGACG | S000024 | Abiotic and biotic stress |
| LEA 2 | ASF1MOTIFCAMV | 244 | (-) | TGACG | S000024 | Abiotic and biotic stress |
| LEA 2 | ASF1MOTIFCAMV | 72 | (-) | TGACG | S000024 | Abiotic and biotic stress |
| LEA 2 | ASF1MOTIFCAMV | 671 | (-) | TGACG | S000024 | Abiotic and biotic stress |
| LEA 2 | ASF1MOTIFCAMV | 193 | (-) | TGACG | S000024 | Abiotic and biotic stress |
| LEA 2 | CBFHV | 324 | (+) | RYCGAC | S000497 | dehydration responsive element |
| LEA 2 | CBFHV | 175 | (-) | RYCGAC | S000497 | dehydration responsive element |
| LEA 2 | CBFHV | 270 | (+) | RYCGAC | S000497 | dehydration responsive element |
| LEA 2 | CBFHV | 410 | (+) | RYCGAC | S000497 | dehydration responsive element |
| LEA 2 | CBFHV | 3 | (-) | RYCGAC | S000497 | dehydration responsive element |
| LEA 2 | CBFHV | 449 | (-) | RYCGAC | S000497 | dehydration responsive element |
| LEA 2 | CBFHV | 423 | (+) | RYCGAC | S000497 | dehydration responsive element |
| LEA 2 | CBFHV | 630 | (-) | RYCGAC | S000497 | dehydration responsive element |
| LEA 2 | CBFHV | 598 | (-) | RYCGAC | S000497 | dehydration responsive element |
| LEA 2 | CBFHV | 349 | (+) | RYCGAC | S000497 | dehydration responsive element |
| LEA 2 | CBFHV | 848 | (+) | RYCGAC | S000497 | dehydration responsive element |
| LEA 2 | CBFHV | 1007 | (+) | RYCGAC | S000497 | dehydration responsive element |
| LEA 2 | CBFHV | 565 | (-) | RYCGAC | S000497 | dehydration responsive element |
| LEA 2 | CBFHV | 75 | (-) | RYCGAC | S000497 | dehydration responsive element |
| LEA 2 | CBFHV | 75 | (+) | RYCGAC | S000497 | dehydration responsive element |
| LEA 2 | CBFHV | 274 | (-) | RYCGAC | S000497 | dehydration responsive element |
| LEA 2 | CBFHV | 274 | (+) | RYCGAC | S000497 | dehydration responsive element |
| LEA 2 | CBFHV | 175 | (-) | RYCGAC | S000497 | dehydration responsive element |
| LEA 2 | LTRECOREATCOR15 | 553 | (-) | CCGAC | S000153 | necessary for coldor drought |
| LEA 2 | LTRECOREATCOR15 | 29 | (-) | CCGAC | S000153 | necessary for coldor drought |
| LEA 2 | LTRECOREATCOR15 | 418 | (-) | CCGAC | S000153 | necessary for coldor drought |
| LEA 2 | LTRECOREATCOR15 | 5 | (+) | CCGAC | S000153 | necessary for coldor drought |
| LEA 2 | LTRECOREATCOR15 | 295 | (+) | CCGAC | S000153 | necessary for coldor drought |
| LEA 2 | LTRECOREATCOR15 | 401 | (-) | CCGAC | S000153 | necessary for coldor drought |
| LEA 2 | LTRECOREATCOR15 | 225 | (+) | CCGAC | S000153 | necessary for coldor drought |
| LEA 2 | LTRECOREATCOR15 | 231 | (+) | CCGAC | S000153 | necessary for coldor drought |
| LEA 2 | LTRECOREATCOR15 | 15 | (-) | CCGAC | S000153 | necessary for coldor drought |
| LEA 2 | LTRECOREATCOR15 | 210 | (-) | CCGAC | S000153 | necessary for coldor drought |
| LEA 2 | LTRECOREATCOR15 | 821 | (-) | CCGAC | S000153 | necessary for coldor drought |
| LEA 2 | LTRECOREATCOR15 | 242 | (-) | CCGAC | S000153 | necessary for coldor drought |
| LEA 2 | LTRECOREATCOR15 | 489 | (-) | CCGAC | S000153 | necessary for coldor drought |
| LEA 2 | LTRECOREATCOR15 | 637 | (-) | CCGAC | S000153 | necessary for coldor drought |
| LEA 2 | LTRECOREATCOR15 | 71 | (-) | CCGAC | S000153 | necessary for coldor drought |
| LEA 2 | LTRECOREATCOR15 | 185 | (+) | CCGAC | S000153 | necessary for coldor drought |
| LEA 2 | LTRECOREATCOR15 | 3 | (-) | CCGAC | S000153 | necessary for coldor drought |
| LEA 2 | LTRECOREATCOR15 | 64 | (-) | CCGAC | S000153 | necessary for coldor drought |
| LEA 2 | LTRECOREATCOR15 | 385 | (-) | CCGAC | S000153 | necessary for coldor drought |
| LEA 2 | LTRECOREATCOR15 | 137 | (+) | CCGAC | S000153 | necessary for coldor drought |
| LEA 2 | LTRECOREATCOR15 | 182 | (+) | CCGAC | S000153 | necessary for coldor drought |
| LEA 2 | LTRECOREATCOR15 | 232 | (+) | CCGAC | S000153 | necessary for coldor drought |
| LEA 2 | LTRECOREATCOR15 | 60 | (-) | CCGAC | S000153 | necessary for coldor drought |
| LEA 2 | LTRECOREATCOR15 | 671 | (-) | CCGAC | S000153 | necessary for coldor drought |
| LEA 2 | LTRECOREATCOR15 | 231 | (+) | CCGAC | S000153 | necessary for coldor drought |
| LEA 2 | LTRECOREATCOR15 | 497 | (+) | CCGAC | S000153 | necessary for coldor drought |
| LEA 2 | LTRECOREATCOR15 | 818 | (-) | CCGAC | S000153 | necessary for coldor drought |
| LEA 2 | LTRECOREATCOR15 | 191 | (+) | CCGAC | S000153 | necessary for coldor drought |
| LEA 2 | LTRECOREATCOR15 | 225 | (+) | CCGAC | S000153 | necessary for coldor drought |
| LEA 2 | LTRECOREATCOR15 | 231 | (+) | CCGAC | S000153 | necessary for coldor drought |
| LEA 2 | LTRECOREATCOR15 | 380 | (-) | CCGAC | S000153 | necessary for coldor drought |
| LEA 2 | LTRECOREATCOR15 | 416 | (-) | CCGAC | S000153 | necessary for coldor drought |
| LEA 2 | LTRECOREATCOR15 | 495 | (-) | CCGAC | S000153 | necessary for coldor drought |
| LEA 2 | LTRECOREATCOR15 | 585 | (-) | CCGAC | S000153 | necessary for coldor drought |
| LEA 2 | LTRECOREATCOR15 | 588 | (-) | CCGAC | S000153 | necessary for coldor drought |
| LEA 2 | LTRECOREATCOR15 | 599 | (-) | CCGAC | S000153 | necessary for coldor drought |
| LEA 2 | LTRECOREATCOR15 | 71 | (-) | CCGAC | S000153 | necessary for coldor drought |
| LEA 2 | LTRECOREATCOR15 | 185 | (+) | CCGAC | S000153 | necessary for coldor drought |
| LEA 2 | LTRECOREATCOR15 | 3 | (-) | CCGAC | S000153 | necessary for coldor drought |
| LEA 2 | LTRECOREATCOR15 | 411 | (-) | CCGAC | S000153 | necessary for coldor drought |
| LEA 2 | LTRECOREATCOR15 | 802 | (+) | CCGAC | S000153 | necessary for coldor drought |
| LEA 2 | LTRECOREATCOR15 | 14 | (+) | CCGAC | S000153 | necessary for coldor drought |
| LEA 2 | LTRECOREATCOR15 | 380 | (-) | CCGAC | S000153 | necessary for coldor drought |
| LEA 2 | LTRECOREATCOR15 | 313 | (+) | CCGAC | S000153 | necessary for coldor drought |
| LEA 2 | LTRECOREATCOR15 | 414 | (-) | CCGAC | S000153 | necessary for coldor drought |
| LEA 2 | LTRECOREATCOR15 | 576 | (-) | CCGAC | S000153 | necessary for coldor drought |
| LEA 2 | LTRECOREATCOR15 | 565 | (-) | CCGAC | S000153 | necessary for coldor drought |
| LEA 2 | LTRECOREATCOR15 | 269 | (-) | CCGAC | S000153 | necessary for coldor drought |
| LEA 2 | MYBCONSENSUSAT | 554 | (-) | YAACKG | S000409 | MYB recognition site |
| LEA 2 | MYBCONSENSUSAT | 509 | (-) | YAACKG | S000409 | MYB recognition site |
| LEA 2 | MYBCONSENSUSAT | 351 | (+) | YAACKG | S000409 | MYB recognition site |
| LEA 2 | MYBCONSENSUSAT | 647 | (-) | YAACKG | S000409 | MYB recognition site |
| LEA 2 | MYBCONSENSUSAT | 700 | (+) | YAACKG | S000409 | MYB recognition site |
| LEA 2 | MYBCONSENSUSAT | 680 | (-) | YAACKG | S000409 | MYB recognition site |
| LEA 2 | MYBCONSENSUSAT | 461 | (-) | YAACKG | S000409 | MYB recognition site |
| LEA 2 | MYBCONSENSUSAT | 495 | (+) | YAACKG | S000409 | MYB recognition site |
| LEA 2 | MYBCONSENSUSAT | 702 | (-) | YAACKG | S000409 | MYB recognition site |
| LEA 2 | MYBCONSENSUSAT | 724 | (+) | YAACKG | S000409 | MYB recognition site |
| LEA 2 | MYBCONSENSUSAT | 739 | (+) | YAACKG | S000409 | MYB recognition site |
| LEA 2 | MYBCONSENSUSAT | 294 | (-) | YAACKG | S000409 | MYB recognition site |
| LEA 2 | MYBCONSENSUSAT | 332 | (+) | YAACKG | S000409 | MYB recognition site |
| LEA 2 | MYBCONSENSUSAT | 538 | (-) | YAACKG | S000409 | MYB recognition site |
| LEA 2 | MYBCONSENSUSAT | 70 | (+) | YAACKG | S000409 | MYB recognition site |
| LEA 2 | MYBCONSENSUSAT | 94 | (+) | YAACKG | S000409 | MYB recognition site |
| LEA 2 | MYBCONSENSUSAT | 262 | (-) | YAACKG | S000409 | MYB recognition site |
| LEA 2 | MYBCONSENSUSAT | 476 | (-) | YAACKG | S000409 | MYB recognition site |
| LEA 2 | MYBCONSENSUSAT | 571 | (-) | YAACKG | S000409 | MYB recognition site |
| LEA 2 | MYBCONSENSUSAT | 733 | (+) | YAACKG | S000409 | MYB recognition site |
| LEA 2 | MYBCONSENSUSAT | 13 | (-) | YAACKG | S000409 | MYB recognition site |
| LEA 2 | MYBCONSENSUSAT | 241 | (+) | YAACKG | S000409 | MYB recognition site |
| LEA 2 | MYBCONSENSUSAT | 119 | (-) | YAACKG | S000409 | MYB recognition site |
| LEA 2 | MYBCONSENSUSAT | 435 | (-) | YAACKG | S000409 | MYB recognition site |
| LEA 2 | MYBCONSENSUSAT | 526 | (-) | YAACKG | S000409 | MYB recognition site |
| LEA 2 | MYBCONSENSUSAT | 725 | (-) | YAACKG | S000409 | MYB recognition site |
| LEA 2 | MYBCONSENSUSAT | 391 | (+) | YAACKG | S000409 | MYB recognition site |
| LEA 2 | MYBCONSENSUSAT | 525 | (-) | YAACKG | S000409 | MYB recognition site |
| LEA 2 | MYBCONSENSUSAT | 135 | (-) | YAACKG | S000409 | MYB recognition site |
| LEA 2 | MYBCONSENSUSAT | 424 | (-) | YAACKG | S000409 | MYB recognition site |
| LEA 2 | MYBCONSENSUSAT | 519 | (-) | YAACKG | S000409 | MYB recognition site |
| LEA 2 | MYBCONSENSUSAT | 461 | (-) | YAACKG | S000409 | MYB recognition site |
| LEA 2 | MYBCONSENSUSAT | 495 | (+) | YAACKG | S000409 | MYB recognition site |
| LEA 2 | MYBCONSENSUSAT | 702 | (-) | YAACKG | S000409 | MYB recognition site |
| LEA 2 | MYBCONSENSUSAT | 724 | (+) | YAACKG | S000409 | MYB recognition site |
| LEA 2 | MYBCONSENSUSAT | 739 | (+) | YAACKG | S000409 | MYB recognition site |
| LEA 2 | MYBCONSENSUSAT | 376 | (-) | YAACKG | S000409 | MYB recognition site |
| LEA 2 | MYBCONSENSUSAT | 61 | (+) | YAACKG | S000409 | MYB recognition site |
| LEA 2 | MYBCONSENSUSAT | 96 | (+) | YAACKG | S000409 | MYB recognition site |
| LEA 2 | MYBCONSENSUSAT | 107 | (+) | YAACKG | S000409 | MYB recognition site |
| LEA 2 | MYBCONSENSUSAT | 511 | (+) | YAACKG | S000409 | MYB recognition site |
| LEA 2 | MYBCONSENSUSAT | 652 | (-) | YAACKG | S000409 | MYB recognition site |
| LEA 2 | MYBCONSENSUSAT | 37 | (-) | YAACKG | S000409 | MYB recognition site |
| LEA 2 | MYBCONSENSUSAT | 92 | (-) | YAACKG | S000409 | MYB recognition site |
| LEA 2 | MYBCONSENSUSAT | 600 | (-) | YAACKG | S000409 | MYB recognition site |
| LEA 2 | MYBCONSENSUSAT | 118 | (+) | YAACKG | S000409 | MYB recognition site |
| LEA 2 | MYBCONSENSUSAT | 134 | (+) | YAACKG | S000409 | MYB recognition site |
| LEA 2 | MYBCONSENSUSAT | 572 | (+) | YAACKG | S000409 | MYB recognition site |
| LEA 2 | MYBCONSENSUSAT | 514 | (+) | YAACKG | S000409 | MYB recognition site |
| LEA 2 | MYBCONSENSUSAT | 217 | (+) | YAACKG | S000409 | MYB recognition site |
| LEA 2 | MYBCONSENSUSAT | 87 | (+) | YAACKG | S000409 | MYB recognition site |
| LEA 2 | MYBCONSENSUSAT | 417 | (+) | YAACKG | S000409 | MYB recognition site |
| LEA 2 | MYBCONSENSUSAT | 268 | (+) | YAACKG | S000409 | MYB recognition site |
| LEA 2 | MYBCONSENSUSAT | 174 | (-) | YAACKG | S000409 | MYB recognition site |
| LEA 2 | MYBCONSENSUSAT | 243 | (-) | YAACKG | S000409 | MYB recognition site |
| LEA 2 | MYBCONSENSUSAT | 654 | (+) | YAACKG | S000409 | MYB recognition site |
| LEA 2 | MYBCONSENSUSAT | 1060 | (+) | YAACKG | S000409 | MYB recognition site |
| LEA 2 | MYBCONSENSUSAT | 7 | (-) | YAACKG | S000409 | MYB recognition site |
| LEA 2 | MYBCONSENSUSAT | 666 | (-) | YAACKG | S000409 | MYB recognition site |
| LEA 2 | MYBCONSENSUSAT | 572 | (+) | YAACKG | S000409 | MYB recognition site |
| LEA 2 | MYBCONSENSUSAT | 149 | (+) | YAACKG | S000409 | MYB recognition site |
| LEA 2 | MYBCONSENSUSAT | 119 | (-) | YAACKG | S000409 | MYB recognition site |
| LEA 2 | MYBCONSENSUSAT | 521 | (-) | YAACKG | S000409 | MYB recognition site |
| LEA 2 | MYBCONSENSUSAT | 521 | (-) | YAACKG | S000409 | MYB recognition site |
| LEA 2 | MYBCONSENSUSAT | 61 | (+) | YAACKG | S000409 | MYB recognition site |
| LEA 2 | MYBCONSENSUSAT | 96 | (+) | YAACKG | S000409 | MYB recognition site |
| LEA 2 | MYBCONSENSUSAT | 107 | (+) | YAACKG | S000409 | MYB recognition site |
| LEA 2 | MYBCONSENSUSAT | 511 | (+) | YAACKG | S000409 | MYB recognition site |
| LEA 2 | MYBCONSENSUSAT | 652 | (-) | YAACKG | S000409 | MYB recognition site |
| LEA 2 | MYBCONSENSUSAT | 291 | (+) | YAACKG | S000409 | MYB recognition site |
| LEA 2 | MYBCONSENSUSAT | 491 | (-) | YAACKG | S000409 | MYB recognition site |
| LEA 2 | MYBCONSENSUSAT | 240 | (-) | YAACKG | S000409 | MYB recognition site |
| LEA 2 | MYBCONSENSUSAT | 300 | (-) | YAACKG | S000409 | MYB recognition site |
| LEA 2 | MYBCONSENSUSAT | 174 | (-) | YAACKG | S000409 | MYB recognition site |
| LEA 2 | MYBCONSENSUSAT | 243 | (-) | YAACKG | S000409 | MYB recognition site |
| LEA 2 | MYBCONSENSUSAT | 203 | (-) | YAACKG | S000409 | MYB recognition site |
| LEA 2 | MYBCONSENSUSAT | 514 | (-) | YAACKG | S000409 | MYB recognition site |
| LEA 2 | MYBCONSENSUSAT | 666 | (-) | YAACKG | S000409 | MYB recognition site |
| LEA 2 | MYBCONSENSUSAT | 162 | (-) | YAACKG | S000409 | MYB recognition site |
| LEA 2 | MYBCONSENSUSAT | 521 | (-) | YAACKG | S000409 | MYB recognition site |
| LEA 2 | MYBCONSENSUSAT | 618 | (-) | YAACKG | S000409 | MYB recognition site |
| LEA 2 | MYBCONSENSUSAT | 119 | (-) | YAACKG | S000409 | MYB recognition site |
| LEA 2 | MYBCONSENSUSAT | 118 | (+) | YAACKG | S000409 | MYB recognition site |
| LEA 2 | MYBCONSENSUSAT | 572 | (+) | YAACKG | S000409 | MYB recognition site |
| LEA 2 | MYBCONSENSUSAT | 291 | (+) | YAACKG | S000409 | MYB recognition site |
| LEA 2 | MYBCONSENSUSAT | 491 | (-) | YAACKG | S000409 | MYB recognition site |
| LEA 2 | MYBCONSENSUSAT | 466 | (-) | YAACKG | S000409 | MYB recognition site |
| LEA 2 | MYBCONSENSUSAT | 219 | (-) | YAACKG | S000409 | MYB recognition site |
| LEA 2 | MYBCONSENSUSAT | 488 | (-) | YAACKG | S000409 | MYB recognition site |
| LEA 2 | MYBCONSENSUSAT | 589 | (-) | YAACKG | S000409 | MYB recognition site |
| LEA 2 | MYBCONSENSUSAT | 35 | (-) | YAACKG | S000409 | MYB recognition site |
| LEA 2 | MYBCONSENSUSAT | 597 | (-) | YAACKG | S000409 | MYB recognition site |
| LEA 2 | MYBCONSENSUSAT | 629 | (-) | YAACKG | S000409 | MYB recognition site |
| LEA 2 | MYBCONSENSUSAT | 737 | (+) | YAACKG | S000409 | MYB recognition site |
| LEA 2 | MYBCONSENSUSAT | 733 | (+) | YAACKG | S000409 | MYB recognition site |
| LEA 2 | MYBCONSENSUSAT | 572 | (+) | YAACKG | S000409 | MYB recognition site |
| LEA 2 | MYBCONSENSUSAT | 93 | (-) | YAACKG | S000409 | MYB recognition site |
| LEA 2 | MYBCONSENSUSAT | 1315 | (-) | YAACKG | S000409 | MYB recognition site |
| LEA 2 | MYBCONSENSUSAT | 1354 | (+) | YAACKG | S000409 | MYB recognition site |
| LEA 2 | MYBCONSENSUSAT | 220 | (+) | YAACKG | S000409 | MYB recognition site |
| LEA 2 | MYBCONSENSUSAT | 343 | (-) | YAACKG | S000409 | MYB recognition site |
| LEA 2 | MYBCONSENSUSAT | 649 | (-) | YAACKG | S000409 | MYB recognition site |
| LEA 2 | MYBCONSENSUSAT | 856 | (-) | YAACKG | S000409 | MYB recognition site |
| LEA 2 | MYBCONSENSUSAT | 60 | (+) | YAACKG | S000409 | MYB recognition site |
| LEA 2 | MYBCONSENSUSAT | 219 | (-) | YAACKG | S000409 | MYB recognition site |
| LEA 2 | MYBCONSENSUSAT | 380 | (-) | YAACKG | S000409 | MYB recognition site |
| LEA 2 | MYBCONSENSUSAT | 132 | (-) | YAACKG | S000409 | MYB recognition site |
| LEA 2 | MYBCONSENSUSAT | 149 | (+) | YAACKG | S000409 | MYB recognition site |
| LEA 2 | MYBCONSENSUSAT | 935 | (+) | YAACKG | S000409 | MYB recognition site |
| LEA 2 | MYBCONSENSUSAT | 513 | (+) | YAACKG | S000409 | MYB recognition site |
| LEA 2 | MYBCONSENSUSAT | 727 | (+) | YAACKG | S000409 | MYB recognition site |
| LEA 2 | MYBCONSENSUSAT | 724 | (-) | YAACKG | S000409 | MYB recognition site |
| LEA 2 | MYBCONSENSUSAT | 68 | (+) | YAACKG | S000409 | MYB recognition site |
| LEA 2 | MYBCONSENSUSAT | 598 | (-) | YAACKG | S000409 | MYB recognition site |
| LEA 2 | MYBCONSENSUSAT | 455 | (+) | YAACKG | S000409 | MYB recognition site |
| LEA 2 | MYBCONSENSUSAT | 529 | (+) | YAACKG | S000409 | MYB recognition site |
| LEA 2 | MYBCONSENSUSAT | 109 | (-) | YAACKG | S000409 | MYB recognition site |
| LEA 2 | MYBCONSENSUSAT | 61 | (+) | YAACKG | S000409 | MYB recognition site |
| LEA 2 | MYBCONSENSUSAT | 501 | (+) | YAACKG | S000409 | MYB recognition site |
| LEA 2 | MYBCONSENSUSAT | 534 | (+) | YAACKG | S000409 | MYB recognition site |
| LEA 2 | MYBCONSENSUSAT | 513 | (+) | YAACKG | S000409 | MYB recognition site |
| LEA 2 | MYBCONSENSUSAT | 140 | (-) | YAACKG | S000409 | MYB recognition site |
| LEA 2 | MYBCONSENSUSAT | 681 | (+) | YAACKG | S000409 | MYB recognition site |
| LEA 2 | MYBCONSENSUSAT | 732 | (-) | YAACKG | S000409 | MYB recognition site |
| LEA 2 | MYBCONSENSUSAT | 939 | (-) | YAACKG | S000409 | MYB recognition site |
| LEA 2 | MYBCONSENSUSAT | 57 | (+) | YAACKG | S000409 | MYB recognition site |
| LEA 2 | MYBCONSENSUSAT | 478 | (+) | YAACKG | S000409 | MYB recognition site |
| LEA 2 | MYBCONSENSUSAT | 391 | (+) | YAACKG | S000409 | MYB recognition site |
| LEA 2 | MYBCONSENSUSAT | 176 | (-) | YAACKG | S000409 | MYB recognition site |
| LEA 2 | MYBCONSENSUSAT | 632 | (-) | YAACKG | S000409 | MYB recognition site |
| LEA 2 | MYBCONSENSUSAT | 534 | (+) | YAACKG | S000409 | MYB recognition site |
| LEA 2 | MYBCONSENSUSAT | 525 | (-) | YAACKG | S000409 | MYB recognition site |
| LEA 2 | MYBCONSENSUSAT | 513 | (-) | YAACKG | S000409 | MYB recognition site |
| LEA 2 | MYBCONSENSUSAT | 785 | (+) | YAACKG | S000409 | MYB recognition site |
| LEA 2 | MYBCONSENSUSAT | 26 | (-) | YAACKG | S000409 | MYB recognition site |
| LEA 2 | MYBCONSENSUSAT | 482 | (-) | YAACKG | S000409 | MYB recognition site |
| LEA 2 | MYBCONSENSUSAT | 503 | (+) | YAACKG | S000409 | MYB recognition site |
| LEA 2 | MYBCONSENSUSAT | 486 | (+) | YAACKG | S000409 | MYB recognition site |
| LEA 2 | MYBCONSENSUSAT | 632 | (-) | YAACKG | S000409 | MYB recognition site |
| LEA 2 | MYBCONSENSUSAT | 646 | (-) | YAACKG | S000409 | MYB recognition site |
| LEA 2 | LTRECOREATCOR15 | 350 | (+) | CCGAC | S000153 | necessary for coldor drought |
| LEA 2 | LTRECOREATCOR15 | 500 | (+) | CCGAC | S000153 | necessary for coldor drought |
| LEA 2 | CBFHV | 425 | (-) | RYCGAC | S000497 | dehydration responsive element |
| LEA 2 | MYBCONSENSUSAT | 372 | (-) | YAACKG | S000409 | MYB recognition site |
| LEA 2 | MYBCONSENSUSAT | 740 | (-) | YAACKG | S000409 | MYB recognition site |
| LEA 2 | MYBCONSENSUSAT | 376 | (-) | YAACKG | S000409 | MYB recognition site |
| LEA 2 | ABRELATERD1 | 429 | (-) | ACGTG | S000414 | ABA responsive complex |
| LEA 2 | ABRELATERD1 | 430 | (+) | ACGTG | S000414 | ABA responsive complex |
| LEA 2 | ABRELATERD1 | 689 | (+) | ACGTG | S000414 | ABA responsive complex |
| LEA 2 | ABRELATERD1 | 452 | (+) | ACGTG | S000414 | ABA responsive complex |
| LEA 2 | ABRELATERD1 | 552 | (+) | ACGTG | S000414 | ABA responsive complex |
| LEA 2 | ABRELATERD1 | 81 | (+) | ACGTG | S000414 | ABA responsive complex |
| LEA 2 | ABRELATERD1 | 96 | (-) | ACGTG | S000414 | ABA responsive complex |
| LEA 2 | ABRELATERD1 | 97 | (+) | ACGTG | S000414 | ABA responsive complex |
| LEA 2 | ABRELATERD1 | 616 | (+) | ACGTG | S000414 | ABA responsive complex |
| LEA 2 | ABRELATERD1 | 689 | (+) | ACGTG | S000414 | ABA responsive complex |
| LEA 2 | ABRELATERD1 | 732 | (+) | ACGTG | S000414 | ABA responsive complex |
| LEA 2 | ABRELATERD1 | 88 | (-) | ACGTG | S000414 | ABA responsive complex |
| LEA 2 | ABRELATERD1 | 159 | (+) | ACGTG | S000414 | ABA responsive complex |
| LEA 2 | ABRELATERD1 | 588 | (-) | ACGTG | S000414 | ABA responsive complex |
| LEA 2 | AGMOTIFNTMYB | 690 | (-) | AGATCCAA | S000444 | induced by various stress |
| LEA 2 | AGMOTIFNTMYB | 543 | (+) | AGATCCAA | S000444 | induced by various stress |
| LEA 2 | AGMOTIFNTMYB | 690 | (-) | AGATCCAA | S000444 | induced by various stress |
| LEA 2 | ARE1 | 707 | (+) | RGTGACNNNGC | S000022 | antioxidant response element |
| LEA 2 | ARE1 | 288 | (+) | RGTGACNNNGC | S000022 | antioxidant response element |
| LEA 2 | ARE1 | 707 | (+) | RGTGACNNNGC | S000022 | antioxidant response element |
| LEA 2 | ARE1 | 592 | (-) | RGTGACNNNGC | S000022 | antioxidant response element |
| LEA 2 | ARE1 | 19 | (-) | RGTGACNNNGC | S000022 | antioxidant response element |
| LEA 2 | ASF1MOTIFCAMV | 112 | (-) | TGACG | S000024 | Abiotic and biotic stress |
| LEA 2 | ABRELATERD1 | 35 | (+) | ACGTG | S000414 | ABA responsive complex |
| LEA 2 | ABRELATERD1 | 277 | (+) | ACGTG | S000414 | ABA responsive complex |
| LEA 2 | ABRELATERD1 | 598 | (+) | ACGTG | S000414 | ABA responsive complex |
| LEA 2 | ABRELATERD1 | 419 | (-) | ACGTG | S000414 | ABA responsive complex |
| LEA 2 | ABRELATERD1 | 476 | (+) | ACGTG | S000414 | ABA responsive complex |
| LEA 2 | ABRELATERD1 | 41 | (-) | ACGTG | S000414 | ABA responsive complex |
| LEA 2 | ABRELATERD1 | 236 | (-) | ACGTG | S000414 | ABA responsive complex |
| LEA 2 | ABRELATERD1 | 250 | (-) | ACGTG | S000414 | ABA responsive complex |
| LEA 2 | ABRELATERD1 | 427 | (+) | ACGTG | S000414 | ABA responsive complex |
| LEA 2 | ABRELATERD1 | 154 | (-) | ACGTG | S000414 | ABA responsive complex |
| LEA 2 | ABRELATERD1 | 155 | (+) | ACGTG | S000414 | ABA responsive complex |
| LEA 2 | ABRELATERD1 | 184 | (-) | ACGTG | S000414 | ABA responsive complex |
| LEA 2 | ABRELATERD1 | 381 | (+) | ACGTG | S000414 | ABA responsive complex |
| LEA 2 | ABRELATERD1 | 472 | (-) | ACGTG | S000414 | ABA responsive complex |
| LEA 2 | ABRELATERD1 | 473 | (+) | ACGTG | S000414 | ABA responsive complex |
| LEA 2 | ABRELATERD1 | 435 | (+) | ACGTG | S000414 | ABA responsive complex |
| LEA 2 | ABRELATERD1 | 88 | (-) | ACGTG | S000414 | ABA responsive complex |
| LEA 2 | ABRELATERD1 | 598 | (+) | ACGTG | S000414 | ABA responsive complex |
| LEA 2 | ABRELATERD1 | 233 | (-) | ACGTG | S000414 | ABA responsive complex |
| LEA 2 | ABRELATERD1 | 247 | (-) | ACGTG | S000414 | ABA responsive complex |
| LEA 2 | ABRELATERD1 | 424 | (+) | ACGTG | S000414 | ABA responsive complex |
| LEA 2 | ABRELATERD1 | 236 | (-) | ACGTG | S000414 | ABA responsive complex |
| LEA 2 | ABRELATERD1 | 427 | (+) | ACGTG | S000414 | ABA responsive complex |
| LEA 2 | ABRELATERD1 | 154 | (-) | ACGTG | S000414 | ABA responsive complex |
| LEA 2 | ABRELATERD1 | 155 | (+) | ACGTG | S000414 | ABA responsive complex |
| LEA 2 | LTRECOREATCOR15 | 380 | (-) | CCGAC | S000153 | necessary for coldor drought |
| LEA 2 | LTRECOREATCOR15 | 416 | (-) | CCGAC | S000153 | necessary for coldor drought |
| LEA 2 | ABRELATERD1 | 184 | (-) | ACGTG | S000414 | ABA responsive complex |
| LEA 2 | ABRELATERD1 | 472 | (-) | ACGTG | S000414 | ABA responsive complex |
| LEA 2 | ASF1MOTIFCAMV | 193 | (-) | TGACG | S000024 | Abiotic and biotic stress |
| LEA 2 | LTRECOREATCOR15 | 495 | (-) | CCGAC | S000153 | necessary for coldor drought |
| LEA 2 | LTRECOREATCOR15 | 518 | (-) | CCGAC | S000153 | necessary for coldor drought |
| LEA 2 | LTRECOREATCOR15 | 668 | (-) | CCGAC | S000153 | necessary for coldor drought |
| LEA 2 | ABRELATERD1 | 473 | (+) | ACGTG | S000414 | ABA responsive complex |
| LEA 2 | ABRELATERD1 | 715 | (+) | ACGTG | S000414 | ABA responsive complex |
| LEA 2 | ABRELATERD1 | 236 | (-) | ACGTG | S000414 | ABA responsive complex |
| LEA 2 | ABRELATERD1 | 427 | (+) | ACGTG | S000414 | ABA responsive complex |
| LEA 2 | ABRELATERD1 | 277 | (+) | ACGTG | S000414 | ABA responsive complex |
| LEA 2 | ABRELATERD1 | 81 | (+) | ACGTG | S000414 | ABA responsive complex |
| LEA 2 | ASF1MOTIFCAMV | 116 | (-) | TGACG | S000024 | Abiotic and biotic stress |
| LEA 2 | ASF1MOTIFCAMV | 316 | (-) | TGACG | S000024 | Abiotic and biotic stress |
| LEA 2 | ASF1MOTIFCAMV | 592 | (+) | TGACG | S000024 | Abiotic and biotic stress |
| LEA 2 | ASF1MOTIFCAMV | 55 | (-) | TGACG | S000024 | Abiotic and biotic stress |
| LEA 2 | ASF1MOTIFCAMV | 503 | (+) | TGACG | S000024 | Abiotic and biotic stress |
| LEA 2 | ASF1MOTIFCAMV | 402 | (+) | TGACG | S000024 | Abiotic and biotic stress |
| LEA 2 | ASF1MOTIFCAMV | 339 | (-) | TGACG | S000024 | Abiotic and biotic stress |
| LEA 2 | ASF1MOTIFCAMV | 520 | (+) | TGACG | S000024 | Abiotic and biotic stress |
| LEA 2 | ASF1MOTIFCAMV | 525 | (-) | TGACG | S000024 | Abiotic and biotic stress |
| LEA 2 | ASF1MOTIFCAMV | 412 | (+) | TGACG | S000024 | Abiotic and biotic stress |
| LEA 2 | CBFHV | 425 | (+) | RYCGAC | S000497 | dehydration responsive element |
| LEA 2 | CBFHV | 952 | (-) | RYCGAC | S000497 | dehydration responsive element |
| LEA 2 | CBFHV | 960 | (-) | RYCGAC | S000497 | dehydration responsive element |
| LEA 2 | LTRECOREATCOR15 | 180 | (-) | CCGAC | S000153 | necessary for coldor drought |
| LEA 2 | LTRECOREATCOR15 | 465 | (-) | CCGAC | S000153 | necessary for coldor drought |
| LEA 2 | LTRECOREATCOR15 | 11 | (+) | CCGAC | S000153 | necessary for coldor drought |
| LEA 2 | LTRECOREATCOR15 | 392 | (-) | CCGAC | S000153 | necessary for coldor drought |
| LEA 2 | LTRECOREATCOR15 | 849 | (+) | CCGAC | S000153 | necessary for coldor drought |
| LEA 2 | LTRECOREATCOR15 | 463 | (-) | CCGAC | S000153 | necessary for coldor drought |
| LEA 2 | MYBCONSENSUSAT | 680 | (-) | YAACKG | S000409 | MYB recognition site |
| LEA 2 | MYBCONSENSUSAT | 292 | (-) | YAACKG | S000409 | MYB recognition site |
| LEA 2 | MYBCONSENSUSAT | 412 | (+) | YAACKG | S000409 | MYB recognition site |
| LEA 2 | MYBCONSENSUSAT | 572 | (+) | YAACKG | S000409 | MYB recognition site |
| LEA 2 | MYBCONSENSUSAT | 513 | (+) | YAACKG | S000409 | MYB recognition site |
| LEA 2 | MYBCONSENSUSAT | 724 | (-) | YAACKG | S000409 | MYB recognition site |
| LEA 2 | MYBCONSENSUSAT | 514 | (-) | YAACKG | S000409 | MYB recognition site |
| LEA 2 | MYBCONSENSUSAT | 513 | (-) | YAACKG | S000409 | MYB recognition site |
| LEA 2 | MYBCONSENSUSAT | 727 | (+) | YAACKG | S000409 | MYB recognition site |
| LEA 2 | MYBCONSENSUSAT | 577 | (+) | YAACKG | S000409 | MYB recognition site |
| LEA 2 | MYBCONSENSUSAT | 840 | (-) | YAACKG | S000409 | MYB recognition site |
| LEA 2 | MYBCONSENSUSAT | 856 | (-) | YAACKG | S000409 | MYB recognition site |
| LEA 2 | MYBCONSENSUSAT | 268 | (+) | YAACKG | S000409 | MYB recognition site |
| LEA 2 | MYBCONSENSUSAT | 445 | (-) | YAACKG | S000409 | MYB recognition site |
| LEA 2 | ASF1MOTIFCAMV | 110 | (+) | TGACG | S000024 | Abiotic and biotic stress |
| LEA 2 | CBFHV | 960 | (+) | RYCGAC | S000497 | dehydration responsive element |
| LEA 2 | LTRECOREATCOR15 | 215 | (-) | CCGAC | S000153 | necessary for coldor drought |
| LEA 2 | MYBCONSENSUSAT | 526 | (-) | YAACKG | S000409 | MYB recognition site |
| LEA 2 | MYBCONSENSUSAT | 124 | (-) | YAACKG | S000409 | MYB recognition site |
| LEA 2 | ASF1MOTIFCAMV | 162 | (-) | TGACG | S000024 | Abiotic and biotic stress |
| LEA 2 | ASF1MOTIFCAMV | 168 | (-) | TGACG | S000024 | Abiotic and biotic stress |
| LEA 2 | ASF1MOTIFCAMV | 107 | (-) | TGACG | S000024 | Abiotic and biotic stress |
| LEA 2 | ASF1MOTIFCAMV | 293 | (+) | TGACG | S000024 | Abiotic and biotic stress |
| LEA 2 | ASF1MOTIFCAMV | 604 | (+) | TGACG | S000024 | Abiotic and biotic stress |
| LEA 2 | ASF1MOTIFCAMV | 140 | (-) | TGACG | S000024 | Abiotic and biotic stress |
| LEA 2 | ASF1MOTIFCAMV | 217 | (-) | TGACG | S000024 | Abiotic and biotic stress |
| LEA 2 | ASF1MOTIFCAMV | 2 | (+) | TGACG | S000024 | Abiotic and biotic stress |
| LEA 2 | ASF1MOTIFCAMV | 278 | (-) | TGACG | S000024 | Abiotic and biotic stress |
| LEA 2 | ASF1MOTIFCAMV | 615 | (-) | TGACG | S000024 | Abiotic and biotic stress |
| LEA 2 | ASF1MOTIFCAMV | 630 | (-) | TGACG | S000024 | Abiotic and biotic stress |
| LEA 2 | ASF1MOTIFCAMV | 195 | (-) | TGACG | S000024 | Abiotic and biotic stress |
| LEA 2 | ASF1MOTIFCAMV | 617 | (+) | TGACG | S000024 | Abiotic and biotic stress |
| LEA 2 | ASF1MOTIFCAMV | 159 | (-) | TGACG | S000024 | Abiotic and biotic stress |
| LEA 2 | ASF1MOTIFCAMV | 430 | (+) | TGACG | S000024 | Abiotic and biotic stress |
| LEA 2 | ASF1MOTIFCAMV | 208 | (-) | TGACG | S000024 | Abiotic and biotic stress |
| LEA 2 | ASF1MOTIFCAMV | 565 | (+) | TGACG | S000024 | Abiotic and biotic stress |
| LEA 2 | CBFHV | 273 | (-) | RYCGAC | S000497 | dehydration responsive element |
| LEA 2 | CBFHV | 322 | (+) | RYCGAC | S000497 | dehydration responsive element |
| LEA 2 | CBFHV | 3 | (-) | RYCGAC | S000497 | dehydration responsive element |
| LEA 2 | CBFHV | 411 | (-) | RYCGAC | S000497 | dehydration responsive element |
| LEA 2 | CBFHV | 801 | (+) | RYCGAC | S000497 | dehydration responsive element |
| LEA 2 | CBFHV | 312 | (+) | RYCGAC | S000497 | dehydration responsive element |
| LEA 2 | CBFHV | 622 | (-) | RYCGAC | S000497 | dehydration responsive element |
| LEA 2 | CBFHV | 622 | (+) | RYCGAC | S000497 | dehydration responsive element |
| LEA 2 | CBFHV | 331 | (-) | RYCGAC | S000497 | dehydration responsive element |
| LEA 2 | LTRECOREATCOR15 | 428 | (-) | CCGAC | S000153 | necessary for coldor drought |
| LEA 2 | LTRECOREATCOR15 | 590 | (-) | CCGAC | S000153 | necessary for coldor drought |
| LEA 2 | LTRECOREATCOR15 | 689 | (+) | CCGAC | S000153 | necessary for coldor drought |
| LEA 2 | LTRECOREATCOR15 | 3 | (-) | CCGAC | S000153 | necessary for coldor drought |
| LEA 2 | LTRECOREATCOR15 | 64 | (-) | CCGAC | S000153 | necessary for coldor drought |
| LEA 2 | LTRECOREATCOR15 | 385 | (-) | CCGAC | S000153 | necessary for coldor drought |
| LEA 2 | LTRECOREATCOR15 | 3 | (-) | CCGAC | S000153 | necessary for coldor drought |
| LEA 2 | LTRECOREATCOR15 | 462 | (-) | CCGAC | S000153 | necessary for coldor drought |
| LEA 2 | LTRECOREATCOR15 | 175 | (-) | CCGAC | S000153 | necessary for coldor drought |
| LEA 2 | LTRECOREATCOR15 | 294 | (-) | CCGAC | S000153 | necessary for coldor drought |
| LEA 2 | LTRECOREATCOR15 | 330 | (-) | CCGAC | S000153 | necessary for coldor drought |
| LEA 2 | LTRECOREATCOR15 | 562 | (-) | CCGAC | S000153 | necessary for coldor drought |
| LEA 2 | LTRECOREATCOR15 | 191 | (+) | CCGAC | S000153 | necessary for coldor drought |
| LEA 2 | LTRECOREATCOR15 | 330 | (+) | CCGAC | S000153 | necessary for coldor drought |
| LEA 2 | LTRECOREATCOR15 | 361 | (-) | CCGAC | S000153 | necessary for coldor drought |
| LEA 2 | LTRECOREATCOR15 | 244 | (-) | CCGAC | S000153 | necessary for coldor drought |
| LEA 2 | LTRECOREATCOR15 | 1098 | (-) | CCGAC | S000153 | necessary for coldor drought |
| LEA 2 | MYBCONSENSUSAT | 455 | (+) | YAACKG | S000409 | MYB recognition site |
| LEA 2 | MYBCONSENSUSAT | 529 | (+) | YAACKG | S000409 | MYB recognition site |
| LEA 2 | MYBCONSENSUSAT | 659 | (+) | YAACKG | S000409 | MYB recognition site |
| LEA 2 | MYBCONSENSUSAT | 162 | (-) | YAACKG | S000409 | MYB recognition site |
| LEA 2 | MYBCONSENSUSAT | 220 | (+) | YAACKG | S000409 | MYB recognition site |
| LEA 2 | MYBCONSENSUSAT | 343 | (-) | YAACKG | S000409 | MYB recognition site |
| LEA 2 | MYBCONSENSUSAT | 93 | (-) | YAACKG | S000409 | MYB recognition site |
| LEA 2 | MYBCONSENSUSAT | 1161 | (-) | YAACKG | S000409 | MYB recognition site |
| LEA 2 | MYBCONSENSUSAT | 1315 | (-) | YAACKG | S000409 | MYB recognition site |
| LEA 2 | MYBCONSENSUSAT | 1354 | (+) | YAACKG | S000409 | MYB recognition site |
| LEA 2 | MYBCONSENSUSAT | 615 | (-) | YAACKG | S000409 | MYB recognition site |
| LEA 2 | MYBCONSENSUSAT | 219 | (-) | YAACKG | S000409 | MYB recognition site |
| LEA 2 | MYBCONSENSUSAT | 470 | (-) | YAACKG | S000409 | MYB recognition site |
| LEA 2 | MYBCONSENSUSAT | 626 | (-) | YAACKG | S000409 | MYB recognition site |
| LEA 2 | MYBCONSENSUSAT | 292 | (-) | YAACKG | S000409 | MYB recognition site |
| LEA 2 | MYBCONSENSUSAT | 412 | (+) | YAACKG | S000409 | MYB recognition site |
| LEA 2 | MYBCONSENSUSAT | 203 | (-) | YAACKG | S000409 | MYB recognition site |
| LEA 2 | MYBCONSENSUSAT | 514 | (-) | YAACKG | S000409 | MYB recognition site |
| LEA 2 | MYBCONSENSUSAT | 185 | (+) | YAACKG | S000409 | MYB recognition site |
| LEA 2 | MYBCONSENSUSAT | 372 | (-) | YAACKG | S000409 | MYB recognition site |
| LEA 2 | MYBCONSENSUSAT | 399 | (+) | YAACKG | S000409 | MYB recognition site |
| LEA 2 | MYBCONSENSUSAT | 372 | (-) | YAACKG | S000409 | MYB recognition site |
| LEA 2 | MYBCONSENSUSAT | 740 | (-) | YAACKG | S000409 | MYB recognition site |
| LEA 2 | MYBCONSENSUSAT | 282 | (-) | YAACKG | S000409 | MYB recognition site |
| LEA 2 | MYBCORE | 61 | (-) | CNGTTR | S000176 | Dehydratio/water stress |
| LEA 2 | MYBCORE | 93 | (-) | CNGTTR | S000176 | Dehydratio/water stress |
| LEA 2 | MYBCORE | 480 | (-) | CNGTTR | S000176 | Dehydratio/water stress |
| LEA 2 | MYBCORE | 501 | (-) | CNGTTR | S000176 | Dehydratio/water stress |
| LEA 2 | MYBCORE | 529 | (-) | CNGTTR | S000176 | Dehydratio/water stress |
| LEA 2 | MYBCORE | 451 | (+) | CNGTTR | S000176 | Dehydratio/water stress |
| LEA 2 | MYBCORE | 632 | (+) | CNGTTR | S000176 | Dehydratio/water stress |
| LEA 2 | MYBCORE | 646 | (+) | CNGTTR | S000176 | Dehydratio/water stress |
| LEA 2 | MYBCORE | 387 | (-) | CNGTTR | S000176 | Dehydratio/water stress |
| LEA 2 | MYBCORE | 588 | (+) | CNGTTR | S000176 | Dehydratio/water stress |
| LEA 2 | MYBCORE | 605 | (-) | CNGTTR | S000176 | Dehydratio/water stress |
| LEA 2 | MYBCORE | 33 | (-) | CNGTTR | S000176 | Dehydratio/water stress |
| LEA 2 | MYBCORE | 433 | (+) | CNGTTR | S000176 | Dehydratio/water stress |
| LEA 2 | MYBCORE | 626 | (+) | CNGTTR | S000176 | Dehydratio/water stress |
| LEA 2 | MYBCORE | 688 | (+) | CNGTTR | S000176 | Dehydratio/water stress |
| LEA 2 | MYBCORE | 372 | (+) | CNGTTR | S000176 | Dehydratio/water stress |
| LEA 2 | MYBCORE | 525 | (+) | CNGTTR | S000176 | Dehydratio/water stress |
| LEA 2 | MYBCORE | 554 | (+) | CNGTTR | S000176 | Dehydratio/water stress |
| LEA 2 | ABRELATERD1 | 96 | (-) | ACGTG | S000414 | ABA responsive complex |
| LEA 2 | ABRELATERD1 | 97 | (+) | ACGTG | S000414 | ABA responsive complex |
| LEA 2 | ABRELATERD1 | 419 | (-) | ACGTG | S000414 | ABA responsive complex |
| LEA 2 | ABRELATERD1 | 389 | (-) | ACGTG | S000414 | ABA responsive complex |
| LEA 2 | ABRELATERD1 | 546 | (+) | ACGTG | S000414 | ABA responsive complex |
| LEA 2 | ABRELATERD1 | 433 | (+) | ACGTG | S000414 | ABA responsive complex |
| LEA 2 | ABRELATERD1 | 913 | (+) | ACGTG | S000414 | ABA responsive complex |
| LEA 2 | ABRELATERD1 | 1168 | (-) | ACGTG | S000414 | ABA responsive complex |
| LEA 2 | ASF1MOTIFCAMV | 281 | (-) | TGACG | S000024 | Abiotic and biotic stress |
| LEA 2 | ASF1MOTIFCAMV | 326 | (+) | TGACG | S000024 | Abiotic and biotic stress |
| LEA 2 | ASF1MOTIFCAMV | 363 | (+) | TGACG | S000024 | Abiotic and biotic stress |
| LEA 2 | ASF1MOTIFCAMV | 412 | (+) | TGACG | S000024 | Abiotic and biotic stress |
| LEA 2 | ASF1MOTIFCAMV | 113 | (-) | TGACG | S000024 | Abiotic and biotic stress |
| LEA 2 | ASF1MOTIFCAMV | 363 | (+) | TGACG | S000024 | Abiotic and biotic stress |
| LEA 2 | ASF1MOTIFCAMV | 366 | (-) | TGACG | S000024 | Abiotic and biotic stress |
| LEA 2 | ASF1MOTIFCAMV | 44 | (-) | TGACG | S000024 | Abiotic and biotic stress |
| LEA 2 | ASF1MOTIFCAMV | 222 | (-) | TGACG | S000024 | Abiotic and biotic stress |
| LEA 2 | ASF1MOTIFCAMV | 920 | (+) | TGACG | S000024 | Abiotic and biotic stress |
| LEA 2 | ASF1MOTIFCAMV | 42 | (+) | TGACG | S000024 | Abiotic and biotic stress |
| LEA 2 | ASF1MOTIFCAMV | 61 | (+) | TGACG | S000024 | Abiotic and biotic stress |
| LEA 2 | ASF1MOTIFCAMV | 286 | (+) | TGACG | S000024 | Abiotic and biotic stress |
| LEA 2 | ASF1MOTIFCAMV | 293 | (+) | TGACG | S000024 | Abiotic and biotic stress |
| LEA 2 | ASF1MOTIFCAMV | 351 | (-) | TGACG | S000024 | Abiotic and biotic stress |
| LEA 2 | ASF1MOTIFCAMV | 396 | (-) | TGACG | S000024 | Abiotic and biotic stress |
| LEA 2 | ASF1MOTIFCAMV | 654 | (+) | TGACG | S000024 | Abiotic and biotic stress |
| LEA 2 | ASF1MOTIFCAMV | 675 | (-) | TGACG | S000024 | Abiotic and biotic stress |
| LEA 2 | ASF1MOTIFCAMV | 370 | (-) | TGACG | S000024 | Abiotic and biotic stress |
| LEA 2 | ASF1MOTIFCAMV | 177 | (-) | TGACG | S000024 | Abiotic and biotic stress |
| LEA 2 | CBFHV | 225 | (-) | RYCGAC | S000497 | dehydration responsive element |
| LEA 2 | CBFHV | 535 | (-) | RYCGAC | S000497 | dehydration responsive element |
| LEA 2 | CBFHV | 75 | (-) | RYCGAC | S000497 | dehydration responsive element |
| LEA 2 | CBFHV | 75 | (+) | RYCGAC | S000497 | dehydration responsive element |
| LEA 2 | CBFHV | 541 | (-) | RYCGAC | S000497 | dehydration responsive element |
| LEA 2 | CBFHV | 3 | (-) | RYCGAC | S000497 | dehydration responsive element |
| LEA 2 | LTRECOREATCOR15 | 554 | (-) | CCGAC | S000153 | necessary for coldor drought |
| LEA 2 | LTRECOREATCOR15 | 392 | (-) | CCGAC | S000153 | necessary for coldor drought |
| LEA 2 | LTRECOREATCOR15 | 715 | (+) | CCGAC | S000153 | necessary for coldor drought |
| LEA 2 | LTRECOREATCOR15 | 127 | (+) | CCGAC | S000153 | necessary for coldor drought |
| LEA 2 | LTRECOREATCOR15 | 225 | (-) | CCGAC | S000153 | necessary for coldor drought |
| LEA 2 | MYB1AT | 402 | (+) | WAACCA | S000408 | MYB recognition site |
| LEA 2 | MYB1AT | 868 | (-) | WAACCA | S000408 | MYB recognition site |
| LEA 2 | MYB1AT | 8 | (+) | WAACCA | S000408 | MYB recognition site |
| LEA 2 | MYB1AT | 464 | (+) | WAACCA | S000408 | MYB recognition site |
| LEA 2 | MYB1AT | 247 | (-) | WAACCA | S000408 | MYB recognition site |
| LEA 2 | MYB1AT | 261 | (+) | WAACCA | S000408 | MYB recognition site |
| LEA 2 | MYB1AT | 231 | (+) | WAACCA | S000408 | MYB recognition site |
| LEA 2 | MYB1AT | 62 | (+) | WAACCA | S000408 | MYB recognition site |
| LEA 2 | MYB1AT | 160 | (+) | WAACCA | S000408 | MYB recognition site |
| LEA 2 | MYB1AT | 24 | (+) | WAACCA | S000408 | MYB recognition site |
| LEA 2 | MYB1AT | 402 | (+) | WAACCA | S000408 | MYB recognition site |
| LEA 2 | MYB1AT | 742 | (-) | WAACCA | S000408 | MYB recognition site |
| LEA 2 | MYB1AT | 165 | (-) | WAACCA | S000408 | MYB recognition site |
| LEA 2 | MYBCORE | 124 | (+) | CNGTTR | S000176 | Dehydratio/water stress |
| LEA 2 | MYBCORE | 225 | (+) | CNGTTR | S000176 | Dehydratio/water stress |
| LEA 2 | MYBCORE | 634 | (-) | CNGTTR | S000176 | Dehydratio/water stress |
| LEA 2 | MYBCORE | 351 | (-) | CNGTTR | S000176 | Dehydratio/water stress |
| LEA 2 | MYBCORE | 647 | (+) | CNGTTR | S000176 | Dehydratio/water stress |
| LEA 2 | MYBCORE | 700 | (-) | CNGTTR | S000176 | Dehydratio/water stress |
| LEA 2 | MYBCORE | 552 | (+) | CNGTTR | S000176 | Dehydratio/water stress |
| LEA 2 | MYBCORE | 680 | (+) | CNGTTR | S000176 | Dehydratio/water stress |
| LEA 2 | MYBCORE | 226 | (+) | CNGTTR | S000176 | Dehydratio/water stress |
| LEA 2 | MYBCORE | 461 | (+) | CNGTTR | S000176 | Dehydratio/water stress |
| LEA 2 | MYBCORE | 495 | (-) | CNGTTR | S000176 | Dehydratio/water stress |
| LEA 2 | MYBCORE | 702 | (+) | CNGTTR | S000176 | Dehydratio/water stress |
| LEA 2 | MYBCORE | 724 | (-) | CNGTTR | S000176 | Dehydratio/water stress |
| LEA 2 | MYBCORE | 739 | (-) | CNGTTR | S000176 | Dehydratio/water stress |
| LEA 2 | MYBCORE | 294 | (+) | CNGTTR | S000176 | Dehydratio/water stress |
| LEA 2 | MYBCORE | 332 | (-) | CNGTTR | S000176 | Dehydratio/water stress |
| LEA 2 | MYBCORE | 538 | (+) | CNGTTR | S000176 | Dehydratio/water stress |
| LEA 2 | MYBCORE | 70 | (-) | CNGTTR | S000176 | Dehydratio/water stress |
| LEA 2 | MYBCORE | 94 | (-) | CNGTTR | S000176 | Dehydratio/water stress |
| LEA 2 | MYBCORE | 183 | (+) | CNGTTR | S000176 | Dehydratio/water stress |
| LEA 2 | MYBCORE | 262 | (+) | CNGTTR | S000176 | Dehydratio/water stress |
| LEA 2 | MYBCORE | 476 | (+) | CNGTTR | S000176 | Dehydratio/water stress |
| LEA 2 | MYBCORE | 571 | (+) | CNGTTR | S000176 | Dehydratio/water stress |
| LEA 2 | MYBCORE | 733 | (-) | CNGTTR | S000176 | Dehydratio/water stress |
| LEA 2 | MYBCORE | 797 | (-) | CNGTTR | S000176 | Dehydratio/water stress |
| LEA 2 | MYBCORE | 29 | (-) | CNGTTR | S000176 | Dehydratio/water stress |
| LEA 2 | MYBCORE | 211 | (-) | CNGTTR | S000176 | Dehydratio/water stress |
| LEA 2 | MYBCORE | 13 | (+) | CNGTTR | S000176 | Dehydratio/water stress |
| LEA 2 | MYBCORE | 175 | (-) | CNGTTR | S000176 | Dehydratio/water stress |
| LEA 2 | MYBCORE | 241 | (-) | CNGTTR | S000176 | Dehydratio/water stress |
| LEA 2 | MYB1AT | 442 | (-) | WAACCA | S000408 | MYB recognition site |
| LEA 2 | MYBCORE | 450 | (+) | CNGTTR | S000176 | Dehydratio/water stress |
| LEA 2 | MYBCORE | 599 | (+) | CNGTTR | S000176 | Dehydratio/water stress |
| LEA 2 | ASF1MOTIFCAMV | 397 | (-) | TGACG | S000024 | Abiotic and biotic stress |
| LEA 2 | MYBCORE | 74 | (-) | CNGTTR | S000176 | Dehydratio/water stress |
| LEA 2 | MYBCORE | 119 | (+) | CNGTTR | S000176 | Dehydratio/water stress |
| LEA 2 | ASF1MOTIFCAMV | 1027 | (+) | TGACG | S000024 | Abiotic and biotic stress |
| LEA 2 | ASF1MOTIFCAMV | 113 | (+) | TGACG | S000024 | Abiotic and biotic stress |
| LEA 2 | ABRELATERD1 | 241 | (-) | ACGTG | S000414 | ABA responsive complex |
| LEA 2 | ABRELATERD1 | 447 | (+) | ACGTG | S000414 | ABA responsive complex |
| LEA 2 | ABRELATERD1 | 983 | (+) | ACGTG | S000414 | ABA responsive complex |
| LEA 2 | ASF1MOTIFCAMV | 599 | (+) | TGACG | S000024 | Abiotic and biotic stress |
| LEA 2 | ASF1MOTIFCAMV | 403 | (-) | TGACG | S000024 | Abiotic and biotic stress |
| LEA 2 | ASF1MOTIFCAMV | 626 | (+) | TGACG | S000024 | Abiotic and biotic stress |
| LEA 2 | ASF1MOTIFCAMV | 36 | (-) | TGACG | S000024 | Abiotic and biotic stress |
| LEA 2 | ASF1MOTIFCAMV | 189 | (-) | TGACG | S000024 | Abiotic and biotic stress |
| LEA 2 | CBFHV | 513 | (-) | RYCGAC | S000497 | dehydration responsive element |
| LEA 2 | CBFHV | 26 | (-) | RYCGAC | S000497 | dehydration responsive element |
| LEA 2 | CBFHV | 422 | (-) | RYCGAC | S000497 | dehydration responsive element |
| LEA 2 | MYB1AT | 15 | (+) | WAACCA | S000408 | MYB recognition site |
| LEA 2 | MYB1AT | 452 | (-) | WAACCA | S000408 | MYB recognition site |
| LEA 2 | MYB1AT | 231 | (+) | WAACCA | S000408 | MYB recognition site |
| LEA 2 | MYB1AT | 11 | (+) | WAACCA | S000408 | MYB recognition site |
| LEA 2 | MYBCORE | 435 | (+) | CNGTTR | S000176 | Dehydratio/water stress |
| LEA 2 | MYBCORE | 523 | (-) | CNGTTR | S000176 | Dehydratio/water stress |
| LEA 2 | MYBCORE | 526 | (+) | CNGTTR | S000176 | Dehydratio/water stress |
| LEA 2 | MYBCORE | 725 | (+) | CNGTTR | S000176 | Dehydratio/water stress |
| LEA 2 | MYBCORE | 63 | (-) | CNGTTR | S000176 | Dehydratio/water stress |
| LEA 2 | MYBCORE | 115 | (+) | CNGTTR | S000176 | Dehydratio/water stress |
| LEA 2 | MYBCORE | 391 | (-) | CNGTTR | S000176 | Dehydratio/water stress |
| LEA 2 | MYBCORE | 372 | (+) | CNGTTR | S000176 | Dehydratio/water stress |
| LEA 2 | MYBCORE | 525 | (+) | CNGTTR | S000176 | Dehydratio/water stress |
| LEA 2 | MYBCORE | 554 | (+) | CNGTTR | S000176 | Dehydratio/water stress |
| LEA 2 | MYBCORE | 135 | (+) | CNGTTR | S000176 | Dehydratio/water stress |
| LEA 2 | MYBCORE | 387 | (+) | CNGTTR | S000176 | Dehydratio/water stress |
| LEA 2 | MYBCORE | 134 | (+) | CNGTTR | S000176 | Dehydratio/water stress |
| LEA 2 | MYBCORE | 210 | (+) | CNGTTR | S000176 | Dehydratio/water stress |
| LEA 2 | MYBCORE | 332 | (-) | CNGTTR | S000176 | Dehydratio/water stress |
| LEA 2 | MYBCORE | 424 | (+) | CNGTTR | S000176 | Dehydratio/water stress |
| LEA 2 | ABRELATERD1 | 35 | (+) | ACGTG | S000414 | ABA responsive complex |
| LEA 2 | ABRELATERD1 | 590 | (-) | ACGTG | S000414 | ABA responsive complex |
| LEA 2 | ABRELATERD1 | 96 | (-) | ACGTG | S000414 | ABA responsive complex |
| LEA 2 | ABRELATERD1 | 97 | (+) | ACGTG | S000414 | ABA responsive complex |
| LEA 2 | ABRELATERD1 | 370 | (+) | ACGTG | S000414 | ABA responsive complex |
| LEA 2 | ABRELATERD1 | 309 | (-) | ACGTG | S000414 | ABA responsive complex |
| LEA 2 | ABRELATERD1 | 206 | (-) | ACGTG | S000414 | ABA responsive complex |
| LEA 2 | ABRELATERD1 | 402 | (+) | ACGTG | S000414 | ABA responsive complex |
| LEA 2 | ABRELATERD1 | 493 | (-) | ACGTG | S000414 | ABA responsive complex |
| LEA 2 | ABRELATERD1 | 494 | (+) | ACGTG | S000414 | ABA responsive complex |
| LEA 2 | ABRELATERD1 | 264 | (-) | ACGTG | S000414 | ABA responsive complex |
| LEA 2 | ABRELATERD1 | 417 | (-) | ACGTG | S000414 | ABA responsive complex |
| LEA 2 | ABRELATERD1 | 418 | (+) | ACGTG | S000414 | ABA responsive complex |
| LEA 2 | ASF1MOTIFCAMV | 664 | (+) | TGACG | S000024 | Abiotic and biotic stress |
| LEA 2 | ASF1MOTIFCAMV | 214 | (-) | TGACG | S000024 | Abiotic and biotic stress |
| LEA 2 | ASF1MOTIFCAMV | 384 | (+) | TGACG | S000024 | Abiotic and biotic stress |
| LEA 2 | ASF1MOTIFCAMV | 244 | (-) | TGACG | S000024 | Abiotic and biotic stress |
| LEA 2 | ASF1MOTIFCAMV | 494 | (+) | TGACG | S000024 | Abiotic and biotic stress |
| LEA 2 | ASF1MOTIFCAMV | 162 | (-) | TGACG | S000024 | Abiotic and biotic stress |
| LEA 2 | ASF1MOTIFCAMV | 56 | (-) | TGACG | S000024 | Abiotic and biotic stress |
| LEA 2 | ASF1MOTIFCAMV | 214 | (-) | TGACG | S000024 | Abiotic and biotic stress |
| LEA 2 | ASF1MOTIFCAMV | 402 | (+) | TGACG | S000024 | Abiotic and biotic stress |
| LEA 2 | ASF1MOTIFCAMV | 217 | (-) | TGACG | S000024 | Abiotic and biotic stress |
| LEA 2 | ASF1MOTIFCAMV | 70 | (-) | TGACG | S000024 | Abiotic and biotic stress |
| LEA 2 | ASF1MOTIFCAMV | 26 | (+) | TGACG | S000024 | Abiotic and biotic stress |
| LEA 2 | ASF1MOTIFCAMV | 143 | (-) | TGACG | S000024 | Abiotic and biotic stress |
| LEA 2 | ASF1MOTIFCAMV | 963 | (+) | TGACG | S000024 | Abiotic and biotic stress |
| LEA 2 | ASF1MOTIFCAMV | 1081 | (-) | TGACG | S000024 | Abiotic and biotic stress |
| LEA 2 | ASF1MOTIFCAMV | 266 | (-) | TGACG | S000024 | Abiotic and biotic stress |
| LEA 2 | ASF1MOTIFCAMV | 97 | (-) | TGACG | S000024 | Abiotic and biotic stress |
| LEA 2 | ASF1MOTIFCAMV | 911 | (+) | TGACG | S000024 | Abiotic and biotic stress |
| LEA 2 | ATHB1ATCONSENSUS | 1531 | (-) | CAATWATTG | S000317 | Abiotic and biotic stress |
| LEA 2 | ATHB1ATCONSENSUS | 1531 | (+) | CAATWATTG | S000317 | Abiotic and biotic stress |
| LEA 2 | BOXLCOREDCPAL | 559 | (-) | ACCWWCC | S000492 | MYB responsive |
| LEA 2 | CBFHV | 422 | (+) | RYCGAC | S000497 | dehydration responsive element |
| LEA 2 | CBFHV | 210 | (-) | RYCGAC | S000497 | dehydration responsive element |
| LEA 2 | CBFHV | 242 | (-) | RYCGAC | S000497 | dehydration responsive element |
| LEA 2 | CBFHV | 489 | (-) | RYCGAC | S000497 | dehydration responsive element |
| LEA 2 | CBFHV | 698 | (-) | RYCGAC | S000497 | dehydration responsive element |
| LEA 2 | CBFHV | 184 | (+) | RYCGAC | S000497 | dehydration responsive element |
| LEA 2 | CBFHV | 3 | (-) | RYCGAC | S000497 | dehydration responsive element |
| LEA 2 | CBFHV | 64 | (-) | RYCGAC | S000497 | dehydration responsive element |
| LEA 2 | CBFHV | 521 | (-) | RYCGAC | S000497 | dehydration responsive element |
| LEA 2 | CBFHV | 181 | (+) | RYCGAC | S000497 | dehydration responsive element |
| LEA 2 | CBFHV | 60 | (-) | RYCGAC | S000497 | dehydration responsive element |
| LEA 2 | CBFHV | 212 | (-) | RYCGAC | S000497 | dehydration responsive element |
| LEA 2 | CBFHV | 212 | (+) | RYCGAC | S000497 | dehydration responsive element |
| LEA 2 | CBFHV | 248 | (-) | RYCGAC | S000497 | dehydration responsive element |
| LEA 2 | CBFHV | 294 | (-) | RYCGAC | S000497 | dehydration responsive element |
| LEA 2 | CBFHV | 37 | (-) | RYCGAC | S000497 | dehydration responsive element |
| LEA 2 | CBFHV | 37 | (+) | RYCGAC | S000497 | dehydration responsive element |
| LEA 2 | CBFHV | 122 | (-) | RYCGAC | S000497 | dehydration responsive element |
| LEA 2 | LTRECOREATCOR15 | 637 | (-) | CCGAC | S000153 | necessary for coldor drought |
| LEA 2 | LTRECOREATCOR15 | 601 | (+) | CCGAC | S000153 | necessary for coldor drought |
| LEA 2 | LTRECOREATCOR15 | 777 | (-) | CCGAC | S000153 | necessary for coldor drought |
| LEA 2 | LTRECOREATCOR15 | 3 | (-) | CCGAC | S000153 | necessary for coldor drought |
| LEA 2 | LTRECOREATCOR15 | 449 | (-) | CCGAC | S000153 | necessary for coldor drought |
| LEA 2 | MYB1AT | 54 | (+) | WAACCA | S000408 | MYB recognition site |
| LEA 2 | MYB1AT | 195 | (-) | WAACCA | S000408 | MYB recognition site |
| LEA 2 | MYB1AT | 62 | (+) | WAACCA | S000408 | MYB recognition site |
| LEA 2 | MYB1AT | 628 | (+) | WAACCA | S000408 | MYB recognition site |
| LEA 2 | MYB1AT | 156 | (+) | WAACCA | S000408 | MYB recognition site |
| LEA 2 | MYB1AT | 293 | (-) | WAACCA | S000408 | MYB recognition site |
| LEA 2 | MYB1AT | 323 | (-) | WAACCA | S000408 | MYB recognition site |
| LEA 2 | MYB1AT | 22 | (+) | WAACCA | S000408 | MYB recognition site |
| LEA 2 | MYB1AT | 666 | (+) | WAACCA | S000408 | MYB recognition site |
| LEA 2 | MYBCORE | 519 | (+) | CNGTTR | S000176 | Dehydratio/water stress |
| LEA 2 | MYBCORE | 226 | (+) | CNGTTR | S000176 | Dehydratio/water stress |
| LEA 2 | MYBCORE | 461 | (+) | CNGTTR | S000176 | Dehydratio/water stress |
| LEA 2 | MYBCORE | 495 | (-) | CNGTTR | S000176 | Dehydratio/water stress |
| LEA 2 | MYBCORE | 702 | (+) | CNGTTR | S000176 | Dehydratio/water stress |
| LEA 2 | MYBCORE | 724 | (-) | CNGTTR | S000176 | Dehydratio/water stress |
| LEA 2 | MYBCORE | 739 | (-) | CNGTTR | S000176 | Dehydratio/water stress |
| LEA 2 | MYBCORE | 376 | (+) | CNGTTR | S000176 | Dehydratio/water stress |
| LEA 2 | MYBCORE | 199 | (+) | CNGTTR | S000176 | Dehydratio/water stress |
| LEA 2 | MYBCORE | 240 | (+) | CNGTTR | S000176 | Dehydratio/water stress |
| LEA 2 | MYBCORE | 341 | (+) | CNGTTR | S000176 | Dehydratio/water stress |
| LEA 2 | MYBCORE | 38 | (-) | CNGTTR | S000176 | Dehydratio/water stress |
| LEA 2 | MYBCORE | 61 | (-) | CNGTTR | S000176 | Dehydratio/water stress |
| LEA 2 | MYBCORE | 96 | (-) | CNGTTR | S000176 | Dehydratio/water stress |
| LEA 2 | MYBCORE | 107 | (-) | CNGTTR | S000176 | Dehydratio/water stress |
| LEA 2 | MYBCORE | 174 | (-) | CNGTTR | S000176 | Dehydratio/water stress |
| LEA 2 | MYBCORE | 511 | (-) | CNGTTR | S000176 | Dehydratio/water stress |
| LEA 2 | MYBCORE | 538 | (-) | CNGTTR | S000176 | Dehydratio/water stress |
| LEA 2 | MYBCORE | 652 | (+) | CNGTTR | S000176 | Dehydratio/water stress |
| LEA 2 | MYBCORE | 37 | (+) | CNGTTR | S000176 | Dehydratio/water stress |
| LEA 2 | MYBCORE | 92 | (+) | CNGTTR | S000176 | Dehydratio/water stress |
| LEA 2 | MYBCORE | 514 | (+) | CNGTTR | S000176 | Dehydratio/water stress |
| LEA 2 | MYBCORE | 600 | (+) | CNGTTR | S000176 | Dehydratio/water stress |
| LEA 2 | MYBCORE | 28 | (-) | CNGTTR | S000176 | Dehydratio/water stress |
| LEA 2 | MYBCORE | 96 | (-) | CNGTTR | S000176 | Dehydratio/water stress |
| LEA 2 | MYBCORE | 118 | (-) | CNGTTR | S000176 | Dehydratio/water stress |
| LEA 2 | MYBCORE | 134 | (-) | CNGTTR | S000176 | Dehydratio/water stress |
| LEA 2 | MYBCORE | 219 | (+) | CNGTTR | S000176 | Dehydratio/water stress |
| LEA 2 | MYBCORE | 278 | (-) | CNGTTR | S000176 | Dehydratio/water stress |
| LEA 2 | MYBCORE | 572 | (-) | CNGTTR | S000176 | Dehydratio/water stress |
| LEA 2 | MYBCORE | 315 | (-) | CNGTTR | S000176 | Dehydratio/water stress |
| LEA 2 | MYBCORE | 514 | (-) | CNGTTR | S000176 | Dehydratio/water stress |
| LEA 2 | MYBCORE | 18 | (-) | CNGTTR | S000176 | Dehydratio/water stress |
| LEA 2 | MYBCORE | 217 | (-) | CNGTTR | S000176 | Dehydratio/water stress |
| LEA 2 | MYBCORE | 87 | (-) | CNGTTR | S000176 | Dehydratio/water stress |
| LEA 2 | MYBCORE | 275 | (-) | CNGTTR | S000176 | Dehydratio/water stress |
| LEA 2 | MYBCORE | 417 | (-) | CNGTTR | S000176 | Dehydratio/water stress |
| LEA 2 | MYBCORE | 562 | (+) | CNGTTR | S000176 | Dehydratio/water stress |
| LEA 2 | MYBCORE | 232 | (-) | CNGTTR | S000176 | Dehydratio/water stress |
| LEA 2 | MYBCORE | 268 | (-) | CNGTTR | S000176 | Dehydratio/water stress |
| LEA 2 | MYBCORE | 386 | (+) | CNGTTR | S000176 | Dehydratio/water stress |
| LEA 2 | MYBCORE | 10 | (-) | CNGTTR | S000176 | Dehydratio/water stress |
| LEA 2 | MYBCORE | 63 | (-) | CNGTTR | S000176 | Dehydratio/water stress |
| LEA 2 | MYBCORE | 174 | (+) | CNGTTR | S000176 | Dehydratio/water stress |
| LEA 2 | MYBCORE | 243 | (+) | CNGTTR | S000176 | Dehydratio/water stress |
| LEA 2 | ABRELATERD1 | 438 | (-) | ACGTG | S000414 | ABA responsive complex |
| LEA 2 | BOXLCOREDCPAL | 689 | (+) | ACCWWCC | S000492 | MYB responsive |
| LEA 2 | MYB1AT | 369 | (+) | WAACCA | S000408 | MYB recognition site |
| LEA 2 | MYBCORE | 579 | (-) | CNGTTR | S000176 | Dehydratio/water stress |
| LEA 2 | MYBCORE | 675 | (+) | CNGTTR | S000176 | Dehydratio/water stress |
| LEA 2 | MYBCORE | 123 | (-) | CNGTTR | S000176 | Dehydratio/water stress |
| LEA 2 | MYB1AT | 327 | (+) | WAACCA | S000408 | MYB recognition site |
| LEA 2 | ABRELATERD1 | 439 | (+) | ACGTG | S000414 | ABA responsive complex |
| LEA 2 | ABRELATERD1 | 464 | (+) | ACGTG | S000414 | ABA responsive complex |
| LEA 2 | ABRELATERD1 | 409 | (+) | ACGTG | S000414 | ABA responsive complex |
| LEA 2 | ABRELATERD1 | 429 | (-) | ACGTG | S000414 | ABA responsive complex |
| LEA 2 | ABRELATERD1 | 430 | (+) | ACGTG | S000414 | ABA responsive complex |
| LEA 2 | ABRELATERD1 | 277 | (+) | ACGTG | S000414 | ABA responsive complex |
| LEA 2 | ABRELATERD1 | 183 | (+) | ACGTG | S000414 | ABA responsive complex |
| LEA 2 | ABRELATERD1 | 192 | (+) | ACGTG | S000414 | ABA responsive complex |
| LEA 2 | ABRELATERD1 | 206 | (-) | ACGTG | S000414 | ABA responsive complex |
| LEA 2 | ABRELATERD1 | 402 | (+) | ACGTG | S000414 | ABA responsive complex |
| LEA 2 | ABRELATERD1 | 493 | (-) | ACGTG | S000414 | ABA responsive complex |
| LEA 2 | ABRELATERD1 | 494 | (+) | ACGTG | S000414 | ABA responsive complex |
| LEA 2 | ABRELATERD1 | 590 | (-) | ACGTG | S000414 | ABA responsive complex |
| LEA 2 | ABRELATERD1 | 96 | (-) | ACGTG | S000414 | ABA responsive complex |
| LEA 2 | ABRELATERD1 | 97 | (+) | ACGTG | S000414 | ABA responsive complex |
| LEA 2 | ABRELATERD1 | 388 | (+) | ACGTG | S000414 | ABA responsive complex |
| LEA 2 | ABRELATERD1 | 1036 | (+) | ACGTG | S000414 | ABA responsive complex |
| LEA 2 | ABRELATERD1 | 467 | (+) | ACGTG | S000414 | ABA responsive complex |
| LEA 2 | ABRELATERD1 | 592 | (+) | ACGTG | S000414 | ABA responsive complex |
| LEA 2 | ABRELATERD1 | 370 | (-) | ACGTG | S000414 | ABA responsive complex |
| LEA 2 | ABRELATERD1 | 371 | (+) | ACGTG | S000414 | ABA responsive complex |
| LEA 2 | ABRELATERD1 | 913 | (+) | ACGTG | S000414 | ABA responsive complex |
| LEA 2 | ABRELATERD1 | 1168 | (-) | ACGTG | S000414 | ABA responsive complex |
| LEA 2 | ABRELATERD1 | 597 | (-) | ACGTG | S000414 | ABA responsive complex |
| LEA 2 | ABRELATERD1 | 192 | (+) | ACGTG | S000414 | ABA responsive complex |
| LEA 2 | ABRELATERD1 | 183 | (+) | ACGTG | S000414 | ABA responsive complex |
| LEA 2 | ASF1MOTIFCAMV | 113 | (-) | TGACG | S000024 | Abiotic and biotic stress |
| LEA 2 | ASF1MOTIFCAMV | 363 | (+) | TGACG | S000024 | Abiotic and biotic stress |
| LEA 2 | ASF1MOTIFCAMV | 366 | (-) | TGACG | S000024 | Abiotic and biotic stress |
| LEA 2 | ASF1MOTIFCAMV | 397 | (-) | TGACG | S000024 | Abiotic and biotic stress |
| LEA 2 | ASF1MOTIFCAMV | 339 | (-) | TGACG | S000024 | Abiotic and biotic stress |
| LEA 2 | ASF1MOTIFCAMV | 520 | (+) | TGACG | S000024 | Abiotic and biotic stress |
| LEA 2 | ASF1MOTIFCAMV | 525 | (-) | TGACG | S000024 | Abiotic and biotic stress |
| LEA 2 | ASF1MOTIFCAMV | 28 | (-) | TGACG | S000024 | Abiotic and biotic stress |
| LEA 2 | ASF1MOTIFCAMV | 163 | (-) | TGACG | S000024 | Abiotic and biotic stress |
| LEA 2 | ASF1MOTIFCAMV | 181 | (-) | TGACG | S000024 | Abiotic and biotic stress |
| LEA 2 | ASF1MOTIFCAMV | 5 | (-) | TGACG | S000024 | Abiotic and biotic stress |
| LEA 2 | ASF1MOTIFCAMV | 379 | (-) | TGACG | S000024 | Abiotic and biotic stress |
| LEA 2 | ASF1MOTIFCAMV | 156 | (-) | TGACG | S000024 | Abiotic and biotic stress |
| LEA 2 | ASF1MOTIFCAMV | 61 | (+) | TGACG | S000024 | Abiotic and biotic stress |
| LEA 2 | ASF1MOTIFCAMV | 566 | (+) | TGACG | S000024 | Abiotic and biotic stress |
| LEA 2 | ASF1MOTIFCAMV | 434 | (-) | TGACG | S000024 | Abiotic and biotic stress |
| LEA 2 | ASF1MOTIFCAMV | 521 | (-) | TGACG | S000024 | Abiotic and biotic stress |
| LEA 2 | ASF1MOTIFCAMV | 107 | (+) | TGACG | S000024 | Abiotic and biotic stress |
| LEA 2 | BOXLCOREDCPAL | 41 | (+) | ACCWWCC | S000492 | MYB responsive |
| LEA 2 | BOXLCOREDCPAL | 76 | (+) | ACCWWCC | S000492 | MYB responsive |
| LEA 2 | BOXLCOREDCPAL | 245 | (+) | ACCWWCC | S000492 | MYB responsive |
| LEA 2 | BOXLCOREDCPAL | 271 | (-) | ACCWWCC | S000492 | MYB responsive |
| LEA 2 | BOXLCOREDCPAL | 436 | (-) | ACCWWCC | S000492 | MYB responsive |
| LEA 2 | BOXLCOREDCPAL | 54 | (+) | ACCWWCC | S000492 | MYB responsive |
| LEA 2 | BOXLCOREDCPAL | 58 | (+) | ACCWWCC | S000492 | MYB responsive |
| LEA 2 | BOXLCOREDCPAL | 339 | (-) | ACCWWCC | S000492 | MYB responsive |
| LEA 2 | BOXLCOREDCPAL | 63 | (+) | ACCWWCC | S000492 | MYB responsive |
| LEA 2 | BOXLCOREDCPAL | 448 | (-) | ACCWWCC | S000492 | MYB responsive |
| LEA 2 | BOXLCOREDCPAL | 47 | (+) | ACCWWCC | S000492 | MYB responsive |
| LEA 2 | BOXLCOREDCPAL | 245 | (+) | ACCWWCC | S000492 | MYB responsive |
| LEA 2 | BOXLCOREDCPAL | 53 | (+) | ACCWWCC | S000492 | MYB responsive |
| LEA 2 | BOXLCOREDCPAL | 431 | (-) | ACCWWCC | S000492 | MYB responsive |
| LEA 2 | BOXLCOREDCPAL | 233 | (+) | ACCWWCC | S000492 | MYB responsive |
| LEA 2 | BOXLCOREDCPAL | 291 | (+) | ACCWWCC | S000492 | MYB responsive |
| LEA 2 | BOXLCOREDCPAL | 585 | (-) | ACCWWCC | S000492 | MYB responsive |
| LEA 2 | BOXLCOREDCPAL | 288 | (-) | ACCWWCC | S000492 | MYB responsive |
| LEA 2 | BOXLCOREDCPAL | 355 | (-) | ACCWWCC | S000492 | MYB responsive |
| LEA 2 | BOXLCOREDCPAL | 427 | (-) | ACCWWCC | S000492 | MYB responsive |
| LEA 2 | BOXLCOREDCPAL | 467 | (-) | ACCWWCC | S000492 | MYB responsive |
| LEA 2 | BOXLCOREDCPAL | 77 | (+) | ACCWWCC | S000492 | MYB responsive |
| LEA 2 | BOXLCOREDCPAL | 322 | (+) | ACCWWCC | S000492 | MYB responsive |
| LEA 2 | BOXLCOREDCPAL | 391 | (-) | ACCWWCC | S000492 | MYB responsive |
| LEA 2 | BOXLCOREDCPAL | 233 | (+) | ACCWWCC | S000492 | MYB responsive |
| LEA 2 | BOXLCOREDCPAL | 145 | (+) | ACCWWCC | S000492 | MYB responsive |
| LEA 2 | BOXLCOREDCPAL | 322 | (+) | ACCWWCC | S000492 | MYB responsive |
| LEA 2 | BOXLCOREDCPAL | 547 | (-) | ACCWWCC | S000492 | MYB responsive |
| LEA 2 | BOXLCOREDCPAL | 388 | (-) | ACCWWCC | S000492 | MYB responsive |
| LEA 2 | BOXLCOREDCPAL | 291 | (+) | ACCWWCC | S000492 | MYB responsive |
| LEA 2 | BOXLCOREDCPAL | 68 | (+) | ACCWWCC | S000492 | MYB responsive |
| LEA 2 | BOXLCOREDCPAL | 585 | (-) | ACCWWCC | S000492 | MYB responsive |
| LEA 2 | BOXLCOREDCPAL | 114 | (+) | ACCWWCC | S000492 | MYB responsive |
| LEA 2 | BOXLCOREDCPAL | 391 | (-) | ACCWWCC | S000492 | MYB responsive |
| LEA 2 | BOXLCOREDCPAL | 145 | (+) | ACCWWCC | S000492 | MYB responsive |
| LEA 2 | CBFHV | 122 | (+) | RYCGAC | S000497 | dehydration responsive element |
| LEA 2 | CBFHV | 496 | (+) | RYCGAC | S000497 | dehydration responsive element |
| LEA 2 | CBFHV | 264 | (-) | RYCGAC | S000497 | dehydration responsive element |
| LEA 2 | CBFHV | 264 | (+) | RYCGAC | S000497 | dehydration responsive element |
| LEA 2 | CBFHV | 172 | (-) | RYCGAC | S000497 | dehydration responsive element |
| LEA 2 | CBFHV | 172 | (+) | RYCGAC | S000497 | dehydration responsive element |
| LEA 2 | CBFHV | 184 | (+) | RYCGAC | S000497 | dehydration responsive element |
| LEA 2 | CBFHV | 3 | (-) | RYCGAC | S000497 | dehydration responsive element |
| LEA 2 | CBFHV | 411 | (-) | RYCGAC | S000497 | dehydration responsive element |
| LEA 2 | CBFHV | 801 | (+) | RYCGAC | S000497 | dehydration responsive element |
| LEA 2 | CBFHV | 13 | (+) | RYCGAC | S000497 | dehydration responsive element |
| LEA 2 | CBFHV | 312 | (+) | RYCGAC | S000497 | dehydration responsive element |
| LEA 2 | CBFHV | 622 | (-) | RYCGAC | S000497 | dehydration responsive element |
| LEA 2 | CBFHV | 622 | (+) | RYCGAC | S000497 | dehydration responsive element |
| LEA 2 | CBFHV | 450 | (+) | RYCGAC | S000497 | dehydration responsive element |
| LEA 2 | CBFHV | 565 | (-) | RYCGAC | S000497 | dehydration responsive element |
| LEA 2 | CBFHV | 349 | (+) | RYCGAC | S000497 | dehydration responsive element |
| LEA 2 | CBFHV | 848 | (+) | RYCGAC | S000497 | dehydration responsive element |
| LEA 2 | CBFHV | 1007 | (+) | RYCGAC | S000497 | dehydration responsive element |
| LEA 2 | CBFHV | 288 | (+) | RYCGAC | S000497 | dehydration responsive element |
| LEA 2 | CBFHV | 474 | (-) | RYCGAC | S000497 | dehydration responsive element |
| LEA 2 | CBFHV | 474 | (+) | RYCGAC | S000497 | dehydration responsive element |
| LEA 2 | CBFHV | 215 | (-) | RYCGAC | S000497 | dehydration responsive element |
| LEA 2 | CBFHV | 462 | (-) | RYCGAC | S000497 | dehydration responsive element |
| LEA 2 | CBFHV | 671 | (-) | RYCGAC | S000497 | dehydration responsive element |
| LEA 2 | CBFHV | 175 | (-) | RYCGAC | S000497 | dehydration responsive element |
| LEA 2 | CBFHV | 172 | (-) | RYCGAC | S000497 | dehydration responsive element |
| LEA 2 | CBFHV | 172 | (+) | RYCGAC | S000497 | dehydration responsive element |
| LEA 2 | CBFHV | 190 | (+) | RYCGAC | S000497 | dehydration responsive element |
| LEA 2 | LTRECOREATCOR15 | 585 | (-) | CCGAC | S000153 | necessary for coldor drought |
| LEA 2 | LTRECOREATCOR15 | 228 | (+) | CCGAC | S000153 | necessary for coldor drought |
| LEA 2 | LTRECOREATCOR15 | 3 | (-) | CCGAC | S000153 | necessary for coldor drought |
| LEA 2 | LTRECOREATCOR15 | 33 | (-) | CCGAC | S000153 | necessary for coldor drought |
| LEA 2 | LTRECOREATCOR15 | 161 | (+) | CCGAC | S000153 | necessary for coldor drought |
| LEA 2 | LTRECOREATCOR15 | 451 | (-) | CCGAC | S000153 | necessary for coldor drought |
| LEA 2 | LTRECOREATCOR15 | 269 | (+) | CCGAC | S000153 | necessary for coldor drought |
| LEA 2 | LTRECOREATCOR15 | 528 | (+) | CCGAC | S000153 | necessary for coldor drought |
| LEA 2 | LTRECOREATCOR15 | 270 | (-) | CCGAC | S000153 | necessary for coldor drought |
| LEA 2 | MYB1AT | 448 | (+) | WAACCA | S000408 | MYB recognition site |
| LEA 2 | MYB1AT | 513 | (-) | WAACCA | S000408 | MYB recognition site |
| LEA 2 | MYB1AT | 75 | (+) | WAACCA | S000408 | MYB recognition site |
| LEA 2 | MYB1AT | 724 | (-) | WAACCA | S000408 | MYB recognition site |
| LEA 2 | MYB1AT | 86 | (+) | WAACCA | S000408 | MYB recognition site |
| LEA 2 | MYB1AT | 192 | (+) | WAACCA | S000408 | MYB recognition site |
| LEA 2 | MYB1AT | 402 | (-) | WAACCA | S000408 | MYB recognition site |
| LEA 2 | MYB1AT | 843 | (+) | WAACCA | S000408 | MYB recognition site |
| LEA 2 | MYB1AT | 464 | (-) | WAACCA | S000408 | MYB recognition site |
| LEA 2 | MYB1AT | 22 | (+) | WAACCA | S000408 | MYB recognition site |
| LEA 2 | MYB1AT | 61 | (+) | WAACCA | S000408 | MYB recognition site |
| LEA 2 | MYB1AT | 618 | (+) | WAACCA | S000408 | MYB recognition site |
| LEA 2 | MYB1AT | 707 | (-) | WAACCA | S000408 | MYB recognition site |
| LEA 2 | MYB1AT | 142 | (-) | WAACCA | S000408 | MYB recognition site |
| LEA 2 | MYB1AT | 194 | (+) | WAACCA | S000408 | MYB recognition site |
| LEA 2 | MYB1AT | 22 | (+) | WAACCA | S000408 | MYB recognition site |
| LEA 2 | MYB1AT | 373 | (-) | WAACCA | S000408 | MYB recognition site |
| LEA 2 | MYB1AT | 192 | (+) | WAACCA | S000408 | MYB recognition site |
| LEA 2 | MYB1AT | 402 | (-) | WAACCA | S000408 | MYB recognition site |
| LEA 2 | MYB1AT | 145 | (-) | WAACCA | S000408 | MYB recognition site |
| LEA 2 | MYB1AT | 161 | (-) | WAACCA | S000408 | MYB recognition site |
| LEA 2 | MYB1AT | 514 | (-) | WAACCA | S000408 | MYB recognition site |
| LEA 2 | MYB1AT | 675 | (-) | WAACCA | S000408 | MYB recognition site |
| LEA 2 | MYBCORE | 478 | (-) | CNGTTR | S000176 | Dehydratio/water stress |
| LEA 2 | MYBCORE | 654 | (-) | CNGTTR | S000176 | Dehydratio/water stress |
| LEA 2 | MYBCORE | 1060 | (-) | CNGTTR | S000176 | Dehydratio/water stress |
| LEA 2 | MYBCORE | 270 | (+) | CNGTTR | S000176 | Dehydratio/water stress |
| LEA 2 | MYBCORE | 516 | (-) | CNGTTR | S000176 | Dehydratio/water stress |
| LEA 2 | MYBCORE | 10 | (-) | CNGTTR | S000176 | Dehydratio/water stress |
| LEA 2 | MYBCORE | 161 | (+) | CNGTTR | S000176 | Dehydratio/water stress |
| LEA 2 | MYBCORE | 198 | (+) | CNGTTR | S000176 | Dehydratio/water stress |
| LEA 2 | MYBCORE | 594 | (-) | CNGTTR | S000176 | Dehydratio/water stress |
| LEA 2 | MYBCORE | 7 | (+) | CNGTTR | S000176 | Dehydratio/water stress |
| LEA 2 | MYBCORE | 102 | (-) | CNGTTR | S000176 | Dehydratio/water stress |
| LEA 2 | MYBCORE | 666 | (+) | CNGTTR | S000176 | Dehydratio/water stress |
| LEA 2 | MYBCORE | 129 | (+) | CNGTTR | S000176 | Dehydratio/water stress |
| LEA 2 | MYBCORE | 572 | (-) | CNGTTR | S000176 | Dehydratio/water stress |
| LEA 2 | MYBCORE | 598 | (+) | CNGTTR | S000176 | Dehydratio/water stress |
| LEA 2 | MYBCORE | 122 | (+) | CNGTTR | S000176 | Dehydratio/water stress |
| LEA 2 | MYBCORE | 119 | (+) | CNGTTR | S000176 | Dehydratio/water stress |
| LEA 2 | MYBCORE | 306 | (-) | CNGTTR | S000176 | Dehydratio/water stress |
| LEA 2 | MYBCORE | 350 | (+) | CNGTTR | S000176 | Dehydratio/water stress |
| LEA 2 | MYBCORE | 521 | (+) | CNGTTR | S000176 | Dehydratio/water stress |
| LEA 2 | MYBCORE | 612 | (-) | CNGTTR | S000176 | Dehydratio/water stress |
| LEA 2 | MYBCORE | 521 | (+) | CNGTTR | S000176 | Dehydratio/water stress |
| LEA 2 | MYBCORE | 612 | (-) | CNGTTR | S000176 | Dehydratio/water stress |
| LEA 2 | MYBCORE | 31 | (-) | CNGTTR | S000176 | Dehydratio/water stress |
| LEA 2 | MYBCORE | 244 | (-) | CNGTTR | S000176 | Dehydratio/water stress |
| LEA 2 | MYBCORE | 38 | (-) | CNGTTR | S000176 | Dehydratio/water stress |
| LEA 2 | MYBCORE | 61 | (-) | CNGTTR | S000176 | Dehydratio/water stress |
| LEA 2 | MYBCORE | 96 | (-) | CNGTTR | S000176 | Dehydratio/water stress |
| LEA 2 | MYBCORE | 107 | (-) | CNGTTR | S000176 | Dehydratio/water stress |
| LEA 2 | MYBCORE | 174 | (-) | CNGTTR | S000176 | Dehydratio/water stress |
| LEA 2 | MYBCORE | 511 | (-) | CNGTTR | S000176 | Dehydratio/water stress |
| LEA 2 | MYBCORE | 538 | (-) | CNGTTR | S000176 | Dehydratio/water stress |
| LEA 2 | MYBCORE | 652 | (+) | CNGTTR | S000176 | Dehydratio/water stress |
| LEA 2 | MYBCORE | 311 | (+) | CNGTTR | S000176 | Dehydratio/water stress |
| LEA 2 | MYBCORE | 451 | (+) | CNGTTR | S000176 | Dehydratio/water stress |
| LEA 2 | MYBCORE | 291 | (-) | CNGTTR | S000176 | Dehydratio/water stress |
| LEA 2 | MYBCORE | 491 | (+) | CNGTTR | S000176 | Dehydratio/water stress |
| LEA 2 | MYBCORE | 485 | (-) | CNGTTR | S000176 | Dehydratio/water stress |
| LEA 2 | MYBCORE | 125 | (-) | CNGTTR | S000176 | Dehydratio/water stress |
| LEA 2 | MYBCORE | 240 | (+) | CNGTTR | S000176 | Dehydratio/water stress |
| LEA 2 | MYBCORE | 387 | (-) | CNGTTR | S000176 | Dehydratio/water stress |
| LEA 2 | MYBCORE | 300 | (+) | CNGTTR | S000176 | Dehydratio/water stress |
| LEA 2 | MYBCORE | 398 | (-) | CNGTTR | S000176 | Dehydratio/water stress |
| LEA 2 | MYBCORE | 10 | (-) | CNGTTR | S000176 | Dehydratio/water stress |
| LEA 2 | MYBCORE | 63 | (-) | CNGTTR | S000176 | Dehydratio/water stress |
| LEA 2 | MYBCORE | 174 | (+) | CNGTTR | S000176 | Dehydratio/water stress |
| LEA 2 | MYBCORE | 243 | (+) | CNGTTR | S000176 | Dehydratio/water stress |
| LEA 2 | MYBCORE | 579 | (-) | CNGTTR | S000176 | Dehydratio/water stress |
| LEA 2 | MYBCORE | 675 | (+) | CNGTTR | S000176 | Dehydratio/water stress |
| LEA 2 | MYBCORE | 39 | (+) | CNGTTR | S000176 | Dehydratio/water stress |
| LEA 2 | MYBCORE | 78 | (+) | CNGTTR | S000176 | Dehydratio/water stress |
| LEA 2 | MYBCORE | 188 | (-) | CNGTTR | S000176 | Dehydratio/water stress |
| LEA 2 | MYBCORE | 222 | (+) | CNGTTR | S000176 | Dehydratio/water stress |
| LEA 2 | MYBCORE | 453 | (+) | CNGTTR | S000176 | Dehydratio/water stress |
| LEA 2 | MYBCORE | 514 | (+) | CNGTTR | S000176 | Dehydratio/water stress |
| LEA 2 | MYBCORE | 102 | (-) | CNGTTR | S000176 | Dehydratio/water stress |
| LEA 2 | MYBCORE | 666 | (+) | CNGTTR | S000176 | Dehydratio/water stress |
| LEA 2 | MYBCORE | 162 | (+) | CNGTTR | S000176 | Dehydratio/water stress |
| LEA 2 | MYBCORE | 180 | (+) | CNGTTR | S000176 | Dehydratio/water stress |
| LEA 2 | MYBCORE | 535 | (-) | CNGTTR | S000176 | Dehydratio/water stress |
| LEA 2 | MYBCORE | 521 | (+) | CNGTTR | S000176 | Dehydratio/water stress |
| LEA 2 | MYBCORE | 612 | (-) | CNGTTR | S000176 | Dehydratio/water stress |
| LEA 2 | MYBCORE | 618 | (+) | CNGTTR | S000176 | Dehydratio/water stress |
| LEA 2 | MYBCORE | 119 | (+) | CNGTTR | S000176 | Dehydratio/water stress |
| LEA 2 | MYBCORE | 347 | (+) | CNGTTR | S000176 | Dehydratio/water stress |
| LEA 2 | MYBCORE | 28 | (-) | CNGTTR | S000176 | Dehydratio/water stress |
| LEA 2 | MYBCORE | 96 | (-) | CNGTTR | S000176 | Dehydratio/water stress |
| LEA 2 | MYBCORE | 118 | (-) | CNGTTR | S000176 | Dehydratio/water stress |
| LEA 2 | MYBCORE | 219 | (+) | CNGTTR | S000176 | Dehydratio/water stress |
| LEA 2 | MYBCORE | 278 | (-) | CNGTTR | S000176 | Dehydratio/water stress |
| LEA 2 | MYBCORE | 572 | (-) | CNGTTR | S000176 | Dehydratio/water stress |
| LEA 2 | MYBCORE | 315 | (-) | CNGTTR | S000176 | Dehydratio/water stress |
| LEA 2 | MYBCORE | 306 | (-) | CNGTTR | S000176 | Dehydratio/water stress |
| LEA 2 | MYBCORE | 350 | (+) | CNGTTR | S000176 | Dehydratio/water stress |
| LEA 2 | MYBCORE | 291 | (-) | CNGTTR | S000176 | Dehydratio/water stress |
| LEA 2 | MYBCORE | 491 | (+) | CNGTTR | S000176 | Dehydratio/water stress |
| LEA 2 | MYBCORE | 90 | (+) | CNGTTR | S000176 | Dehydratio/water stress |
| LEA 2 | MYBCORE | 357 | (-) | CNGTTR | S000176 | Dehydratio/water stress |
| LEA 2 | MYBCORE | 463 | (-) | CNGTTR | S000176 | Dehydratio/water stress |
| LEA 2 | MYBCORE | 466 | (+) | CNGTTR | S000176 | Dehydratio/water stress |
| LEA 2 | MYBCORE | 539 | (+) | CNGTTR | S000176 | Dehydratio/water stress |
| LEA 2 | MYBCORE | 219 | (+) | CNGTTR | S000176 | Dehydratio/water stress |
| LEA 2 | MYBCORE | 232 | (-) | CNGTTR | S000176 | Dehydratio/water stress |
| LEA 2 | MYBCORE | 408 | (-) | CNGTTR | S000176 | Dehydratio/water stress |
| LEA 2 | MYBCORE | 488 | (+) | CNGTTR | S000176 | Dehydratio/water stress |
| LEA 2 | MYBCORE | 589 | (+) | CNGTTR | S000176 | Dehydratio/water stress |
| LEA 2 | MYBCORE | 306 | (-) | CNGTTR | S000176 | Dehydratio/water stress |
| LEA 2 | MYBCORE | 350 | (+) | CNGTTR | S000176 | Dehydratio/water stress |
| LEA 2 | MYBCORE | 28 | (-) | CNGTTR | S000176 | Dehydratio/water stress |
| LEA 2 | MYBCORE | 35 | (+) | CNGTTR | S000176 | Dehydratio/water stress |
| LEA 2 | MYBCORE | 114 | (-) | CNGTTR | S000176 | Dehydratio/water stress |
| LEA 2 | MYBCORE | 177 | (+) | CNGTTR | S000176 | Dehydratio/water stress |
| LEA 2 | MYBCORE | 525 | (-) | CNGTTR | S000176 | Dehydratio/water stress |
| LEA 2 | MYBCORE | 597 | (+) | CNGTTR | S000176 | Dehydratio/water stress |
| LEA 2 | MYBCORE | 629 | (+) | CNGTTR | S000176 | Dehydratio/water stress |
| LEA 2 | MYBCORE | 737 | (-) | CNGTTR | S000176 | Dehydratio/water stress |
| LEA 2 | MYBCORE | 733 | (-) | CNGTTR | S000176 | Dehydratio/water stress |
| LEA 2 | ABRELATERD1 | 96 | (-) | ACGTG | S000414 | ABA responsive complex |
| LEA 2 | ABRELATERD1 | 97 | (+) | ACGTG | S000414 | ABA responsive complex |
| LEA 2 | ABRELATERD1 | 370 | (+) | ACGTG | S000414 | ABA responsive complex |
| LEA 2 | ABREOSRAB1 | 666 | (+) | ACGTSSSC | S000012 | ABA responsive complex |
| LEA 2 | ABREOSRAB1 | 501 | (-) | ACGTSSSC | S000012 | ABA responsive complex |
| LEA 2 | ABREOSRAB1 | 85 | (-) | ACGTSSSC | S000012 | ABA responsive complex |
| LEA 2 | ABREOSRAB1 | 251 | (+) | ACGTSSSC | S000012 | ABA responsive complex |
| LEA 2 | ABREOSRAB1 | 85 | (-) | ACGTSSSC | S000012 | ABA responsive complex |
| LEA 2 | ABREOSRAB1 | 398 | (-) | ACGTSSSC | S000012 | ABA responsive complex |
| LEA 2 | ABREOSRAB1 | 402 | (+) | ACGTSSSC | S000012 | ABA responsive complex |
| LEA 2 | ASF1MOTIFCAMV | 590 | (-) | TGACG | S000024 | Abiotic and biotic stress |
| LEA 2 | ASF1MOTIFCAMV | 250 | (-) | TGACG | S000024 | Abiotic and biotic stress |
| LEA 2 | ASF1MOTIFCAMV | 437 | (+) | TGACG | S000024 | Abiotic and biotic stress |
| LEA 2 | ASF1MOTIFCAMV | 583 | (+) | TGACG | S000024 | Abiotic and biotic stress |
| LEA 2 | ASF1MOTIFCAMV | 299 | (-) | TGACG | S000024 | Abiotic and biotic stress |
| LEA 2 | ASF1MOTIFCAMV | 700 | (+) | TGACG | S000024 | Abiotic and biotic stress |
| LEA 2 | ASF1MOTIFCAMV | 725 | (-) | TGACG | S000024 | Abiotic and biotic stress |
| LEA 2 | BOXLCOREDCPAL | 114 | (+) | ACCWWCC | S000492 | MYB responsive |
| LEA 2 | BOXLCOREDCPAL | 391 | (-) | ACCWWCC | S000492 | MYB responsive |
| LEA 2 | BOXLCOREDCPAL | 144 | (+) | ACCWWCC | S000492 | MYB responsive |
| LEA 2 | BOXLCOREDCPAL | 145 | (-) | ACCWWCC | S000492 | MYB responsive |
| LEA 2 | BOXLCOREDCPAL | 495 | (-) | ACCWWCC | S000492 | MYB responsive |
| LEA 2 | BOXLCOREDCPAL | 63 | (+) | ACCWWCC | S000492 | MYB responsive |
| LEA 2 | BOXLCOREDCPAL | 448 | (-) | ACCWWCC | S000492 | MYB responsive |
| LEA 2 | BOXLCOREDCPAL | 559 | (-) | ACCWWCC | S000492 | MYB responsive |
| LEA 2 | BOXLCOREDCPAL | 105 | (+) | ACCWWCC | S000492 | MYB responsive |
| LEA 2 | BOXLCOREDCPAL | 1260 | (+) | ACCWWCC | S000492 | MYB responsive |
| LEA 2 | BOXLCOREDCPAL | 291 | (+) | ACCWWCC | S000492 | MYB responsive |
| LEA 2 | BOXLCOREDCPAL | 233 | (+) | ACCWWCC | S000492 | MYB responsive |
| LEA 2 | BOXLCOREDCPAL | 331 | (+) | ACCWWCC | S000492 | MYB responsive |
| LEA 2 | BOXLCOREDCPAL | 442 | (-) | ACCWWCC | S000492 | MYB responsive |
| LEA 2 | BOXLCOREDCPAL | 559 | (-) | ACCWWCC | S000492 | MYB responsive |
| LEA 2 | BOXLCOREDCPAL | 146 | (+) | ACCWWCC | S000492 | MYB responsive |
| LEA 2 | BOXLCOREDCPAL | 312 | (-) | ACCWWCC | S000492 | MYB responsive |
| LEA 2 | BOXLCOREDCPAL | 64 | (+) | ACCWWCC | S000492 | MYB responsive |
| LEA 2 | BOXLCOREDCPAL | 488 | (-) | ACCWWCC | S000492 | MYB responsive |
| LEA 2 | BOXLCOREDCPAL | 489 | (-) | ACCWWCC | S000492 | MYB responsive |
| LEA 2 | CBFHV | 361 | (-) | RYCGAC | S000497 | dehydration responsive element |
| LEA 2 | CBFHV | 446 | (-) | RYCGAC | S000497 | dehydration responsive element |
| LEA 2 | CBFHV | 446 | (+) | RYCGAC | S000497 | dehydration responsive element |
| LEA 2 | CBFHV | 76 | (-) | RYCGAC | S000497 | dehydration responsive element |
| LEA 2 | CBFHV | 677 | (-) | RYCGAC | S000497 | dehydration responsive element |
| LEA 2 | CBFHV | 677 | (+) | RYCGAC | S000497 | dehydration responsive element |
| LEA 2 | CBFHV | 1013 | (-) | RYCGAC | S000497 | dehydration responsive element |
| LEA 2 | CBFHV | 108 | (-) | RYCGAC | S000497 | dehydration responsive element |
| LEA 2 | CBFHV | 108 | (+) | RYCGAC | S000497 | dehydration responsive element |
| LEA 2 | CBFHV | 144 | (-) | RYCGAC | S000497 | dehydration responsive element |
| LEA 2 | CBFHV | 190 | (-) | RYCGAC | S000497 | dehydration responsive element |
| LEA 2 | CBFHV | 443 | (-) | RYCGAC | S000497 | dehydration responsive element |
| LEA 2 | LTRECOREATCOR15 | 293 | (+) | CCGAC | S000153 | necessary for coldor drought |
| LEA 2 | LTRECOREATCOR15 | 3 | (-) | CCGAC | S000153 | necessary for coldor drought |
| LEA 2 | LTRECOREATCOR15 | 78 | (-) | CCGAC | S000153 | necessary for coldor drought |
| LEA 2 | LTRECOREATCOR15 | 314 | (+) | CCGAC | S000153 | necessary for coldor drought |
| LEA 2 | MYB1AT | 448 | (+) | WAACCA | S000408 | MYB recognition site |
| LEA 2 | MYB1AT | 513 | (-) | WAACCA | S000408 | MYB recognition site |
| LEA 2 | MYB1AT | 541 | (+) | WAACCA | S000408 | MYB recognition site |
| LEA 2 | MYB1AT | 142 | (-) | WAACCA | S000408 | MYB recognition site |
| LEA 2 | MYB1AT | 61 | (+) | WAACCA | S000408 | MYB recognition site |
| LEA 2 | MYB1AT | 22 | (+) | WAACCA | S000408 | MYB recognition site |
| LEA 2 | MYB1AT | 666 | (+) | WAACCA | S000408 | MYB recognition site |
| LEA 2 | MYB1AT | 61 | (+) | WAACCA | S000408 | MYB recognition site |
| LEA 2 | MYB1AT | 112 | (+) | WAACCA | S000408 | MYB recognition site |
| LEA 2 | MYB1AT | 618 | (+) | WAACCA | S000408 | MYB recognition site |
| LEA 2 | MYB1AT | 377 | (+) | WAACCA | S000408 | MYB recognition site |
| LEA 2 | MYB1AT | 53 | (-) | WAACCA | S000408 | MYB recognition site |
| LEA 2 | MYBCORE | 797 | (-) | CNGTTR | S000176 | Dehydratio/water stress |
| LEA 2 | MYBCORE | 129 | (+) | CNGTTR | S000176 | Dehydratio/water stress |
| LEA 2 | MYBCORE | 315 | (-) | CNGTTR | S000176 | Dehydratio/water stress |
| LEA 2 | MYBCORE | 572 | (-) | CNGTTR | S000176 | Dehydratio/water stress |
| LEA 2 | MYBCORE | 598 | (+) | CNGTTR | S000176 | Dehydratio/water stress |
| LEA 2 | MYBCORE | 387 | (+) | CNGTTR | S000176 | Dehydratio/water stress |
| LEA 2 | MYBCORE | 93 | (+) | CNGTTR | S000176 | Dehydratio/water stress |
| LEA 2 | MYBCORE | 211 | (-) | CNGTTR | S000176 | Dehydratio/water stress |
| LEA 2 | MYBCORE | 846 | (-) | CNGTTR | S000176 | Dehydratio/water stress |
| LEA 2 | MYBCORE | 984 | (-) | CNGTTR | S000176 | Dehydratio/water stress |
| LEA 2 | MYBCORE | 1052 | (-) | CNGTTR | S000176 | Dehydratio/water stress |
| LEA 2 | MYBCORE | 1315 | (+) | CNGTTR | S000176 | Dehydratio/water stress |
| LEA 2 | MYBCORE | 1354 | (-) | CNGTTR | S000176 | Dehydratio/water stress |
| LEA 2 | MYBCORE | 220 | (-) | CNGTTR | S000176 | Dehydratio/water stress |
| LEA 2 | MYBCORE | 343 | (+) | CNGTTR | S000176 | Dehydratio/water stress |
| LEA 2 | MYBCORE | 384 | (+) | CNGTTR | S000176 | Dehydratio/water stress |
| LEA 2 | MYBCORE | 567 | (+) | CNGTTR | S000176 | Dehydratio/water stress |
| LEA 2 | MYBCORE | 649 | (+) | CNGTTR | S000176 | Dehydratio/water stress |
| LEA 2 | MYBCORE | 856 | (+) | CNGTTR | S000176 | Dehydratio/water stress |
| LEA 2 | MYBCORE | 60 | (-) | CNGTTR | S000176 | Dehydratio/water stress |
| LEA 2 | MYBCORE | 219 | (+) | CNGTTR | S000176 | Dehydratio/water stress |
| LEA 2 | MYBCORE | 380 | (+) | CNGTTR | S000176 | Dehydratio/water stress |
| LEA 2 | MYBCORE | 132 | (+) | CNGTTR | S000176 | Dehydratio/water stress |
| LEA 2 | MYBCORE | 306 | (-) | CNGTTR | S000176 | Dehydratio/water stress |
| LEA 2 | MYBCORE | 433 | (+) | CNGTTR | S000176 | Dehydratio/water stress |
| LEA 2 | MYBCORE | 935 | (-) | CNGTTR | S000176 | Dehydratio/water stress |
| LEA 2 | MYBCORE | 484 | (+) | CNGTTR | S000176 | Dehydratio/water stress |
| LEA 2 | MYBCORE | 513 | (-) | CNGTTR | S000176 | Dehydratio/water stress |
| LEA 2 | MYB1AT | 518 | (+) | WAACCA | S000408 | MYB recognition site |
| LEA 2 | MYBCORE | 10 | (-) | CNGTTR | S000176 | Dehydratio/water stress |
| LEA 2 | MYBCORE | 164 | (+) | CNGTTR | S000176 | Dehydratio/water stress |
| LEA 2 | ABREOSRAB1 | 414 | (-) | ACGTSSSC | S000012 | ABA responsive complex |
| LEA 2 | BOXLCOREDCPAL | 146 | (+) | ACCWWCC | S000492 | MYB responsive |
| LEA 2 | CBFHV | 443 | (+) | RYCGAC | S000497 | dehydration responsive element |
| LEA 2 | CBFHV | 264 | (-) | RYCGAC | S000497 | dehydration responsive element |
| LEA 2 | MYB1AT | 61 | (+) | WAACCA | S000408 | MYB recognition site |
| LEA 2 | ASF1MOTIFCAMV | 229 | (+) | TGACG | S000024 | Abiotic and biotic stress |
| LEA 2 | ASF1MOTIFCAMV | 630 | (-) | TGACG | S000024 | Abiotic and biotic stress |
| LEA 2 | ASF1MOTIFCAMV | 276 | (-) | TGACG | S000024 | Abiotic and biotic stress |
| LEA 2 | BOXLCOREDCPAL | 14 | (+) | ACCWWCC | S000492 | MYB responsive |
| LEA 2 | BOXLCOREDCPAL | 476 | (-) | ACCWWCC | S000492 | MYB responsive |
| LEA 2 | CBFHV | 264 | (+) | RYCGAC | S000497 | dehydration responsive element |
| LEA 2 | CBFHV | 225 | (-) | RYCGAC | S000497 | dehydration responsive element |
| LEA 2 | LTRECOREATCOR15 | 655 | (-) | CCGAC | S000153 | necessary for coldor drought |
| LEA 2 | LTRECOREATCOR15 | 3 | (-) | CCGAC | S000153 | necessary for coldor drought |
| LEA 2 | MYBCORE | 597 | (-) | CNGTTR | S000176 | Dehydratio/water stress |
| LEA 2 | MYBCORE | 669 | (+) | CNGTTR | S000176 | Dehydratio/water stress |
| LEA 2 | MYBCORE | 727 | (-) | CNGTTR | S000176 | Dehydratio/water stress |
| LEA 2 | MYBCORE | 323 | (+) | CNGTTR | S000176 | Dehydratio/water stress |
| LEA 2 | MYBCORE | 329 | (+) | CNGTTR | S000176 | Dehydratio/water stress |
| LEA 2 | MYBCORE | 724 | (+) | CNGTTR | S000176 | Dehydratio/water stress |
| LEA 2 | MYBCORE | 68 | (-) | CNGTTR | S000176 | Dehydratio/water stress |
| LEA 2 | ABREOSRAB1 | 435 | (-) | ACGTSSSC | S000012 | ABA responsive complex |
| LEA 2 | ABREOSRAB1 | 183 | (+) | ACGTSSSC | S000012 | ABA responsive complex |
| LEA 2 | ABREOSRAB1 | 192 | (+) | ACGTSSSC | S000012 | ABA responsive complex |
| LEA 2 | ABREOSRAB1 | 398 | (-) | ACGTSSSC | S000012 | ABA responsive complex |
| LEA 2 | ABREOSRAB1 | 402 | (+) | ACGTSSSC | S000012 | ABA responsive complex |
| LEA 2 | ABREOSRAB1 | 508 | (-) | ACGTSSSC | S000012 | ABA responsive complex |
| LEA 2 | ASF1MOTIFCAMV | 627 | (-) | TGACG | S000024 | Abiotic and biotic stress |
| LEA 2 | ASF1MOTIFCAMV | 634 | (-) | TGACG | S000024 | Abiotic and biotic stress |
| LEA 2 | ASF1MOTIFCAMV | 272 | (-) | TGACG | S000024 | Abiotic and biotic stress |
| LEA 2 | BOXLCOREDCPAL | 337 | (+) | ACCWWCC | S000492 | MYB responsive |
| LEA 2 | BOXLCOREDCPAL | 448 | (-) | ACCWWCC | S000492 | MYB responsive |
| LEA 2 | BOXLCOREDCPAL | 212 | (+) | ACCWWCC | S000492 | MYB responsive |
| LEA 2 | BOXLCOREDCPAL | 339 | (-) | ACCWWCC | S000492 | MYB responsive |
| LEA 2 | BOXLCOREDCPAL | 275 | (-) | ACCWWCC | S000492 | MYB responsive |
| LEA 2 | CBFHV | 606 | (-) | RYCGAC | S000497 | dehydration responsive element |
| LEA 2 | LTRE1HVBLT49 | 623 | (+) | CCGAAA | S000250 | low temperature |
| LEA 2 | LTRE1HVBLT49 | 35 | (-) | CCGAAA | S000250 | low temperature |
| LEA 2 | LTRE1HVBLT49 | 219 | (-) | CCGAAA | S000250 | low temperature |
| LEA 2 | LTRECOREATCOR15 | 449 | (-) | CCGAC | S000153 | necessary for coldor drought |
| LEA 2 | LTRECOREATCOR15 | 220 | (+) | CCGAC | S000153 | necessary for coldor drought |
| LEA 2 | LTRECOREATCOR15 | 601 | (+) | CCGAC | S000153 | necessary for coldor drought |
| LEA 2 | LTRECOREATCOR15 | 777 | (-) | CCGAC | S000153 | necessary for coldor drought |
| LEA 2 | LTRECOREATCOR15 | 386 | (-) | CCGAC | S000153 | necessary for coldor drought |
| LEA 2 | MYB1AT | 112 | (+) | WAACCA | S000408 | MYB recognition site |
| LEA 2 | MYB1AT | 618 | (+) | WAACCA | S000408 | MYB recognition site |
| LEA 2 | MYB1AT | 123 | (-) | WAACCA | S000408 | MYB recognition site |
| LEA 2 | MYB1AT | 742 | (-) | WAACCA | S000408 | MYB recognition site |
| LEA 2 | MYB1AT | 464 | (-) | WAACCA | S000408 | MYB recognition site |
| LEA 2 | MYB1AT | 22 | (+) | WAACCA | S000408 | MYB recognition site |
| LEA 2 | MYB1AT | 11 | (+) | WAACCA | S000408 | MYB recognition site |
| LEA 2 | MYBCORE | 483 | (-) | CNGTTR | S000176 | Dehydratio/water stress |
| LEA 2 | MYBCORE | 598 | (+) | CNGTTR | S000176 | Dehydratio/water stress |
| LEA 2 | MYBCORE | 387 | (-) | CNGTTR | S000176 | Dehydratio/water stress |
| LEA 2 | MYBCORE | 343 | (-) | CNGTTR | S000176 | Dehydratio/water stress |
| LEA 2 | MYBCORE | 529 | (-) | CNGTTR | S000176 | Dehydratio/water stress |
| LEA 2 | MYBCORE | 31 | (-) | CNGTTR | S000176 | Dehydratio/water stress |
| LEA 2 | MYBCORE | 244 | (-) | CNGTTR | S000176 | Dehydratio/water stress |
| LEA 2 | MYBCORE | 148 | (+) | CNGTTR | S000176 | Dehydratio/water stress |
| LEA 2 | MYBCORE | 797 | (-) | CNGTTR | S000176 | Dehydratio/water stress |
| LEA 2 | MYBCORE | 109 | (+) | CNGTTR | S000176 | Dehydratio/water stress |
| LEA 2 | MYBCORE | 247 | (-) | CNGTTR | S000176 | Dehydratio/water stress |
| LEA 2 | MYBCORE | 61 | (-) | CNGTTR | S000176 | Dehydratio/water stress |
| LEA 2 | MYBCORE | 93 | (-) | CNGTTR | S000176 | Dehydratio/water stress |
| LEA 2 | MYBCORE | 480 | (-) | CNGTTR | S000176 | Dehydratio/water stress |
| LEA 2 | MYBCORE | 501 | (-) | CNGTTR | S000176 | Dehydratio/water stress |
| LEA 2 | MYBCORE | 529 | (-) | CNGTTR | S000176 | Dehydratio/water stress |
| LEA 2 | MYBCORE | 510 | (-) | CNGTTR | S000176 | Dehydratio/water stress |
| LEA 2 | MYBCORE | 534 | (-) | CNGTTR | S000176 | Dehydratio/water stress |
| LEA 2 | MYBCORE | 484 | (+) | CNGTTR | S000176 | Dehydratio/water stress |
| LEA 2 | MYBCORE | 513 | (-) | CNGTTR | S000176 | Dehydratio/water stress |
| LEA 2 | MYBCORE | 601 | (+) | CNGTTR | S000176 | Dehydratio/water stress |
| LEA 2 | MYBCORE | 140 | (+) | CNGTTR | S000176 | Dehydratio/water stress |
| LEA 2 | MYBCORE | 681 | (-) | CNGTTR | S000176 | Dehydratio/water stress |
| LEA 2 | MYBCORE | 732 | (+) | CNGTTR | S000176 | Dehydratio/water stress |
| LEA 2 | MYB1AT | 54 | (+) | WAACCA | S000408 | MYB recognition site |
| LEA 2 | ABREOSRAB1 | 383 | (-) | ACGTSSSC | S000012 | ABA responsive complex |
| LEA 2 | ABREOSRAB1 | 463 | (-) | ACGTSSSC | S000012 | ABA responsive complex |
| LEA 2 | ABREOSRAB1 | 588 | (-) | ACGTSSSC | S000012 | ABA responsive complex |
| LEA 2 | ABREOSRAB1 | 192 | (+) | ACGTSSSC | S000012 | ABA responsive complex |
| LEA 2 | ABREOSRAB1 | 183 | (+) | ACGTSSSC | S000012 | ABA responsive complex |
| LEA 2 | ABRERATCAL | 579 | (+) | MACGYGB | S000507 | ABA responsive complex |
| LEA 2 | ABRERATCAL | 500 | (-) | MACGYGB | S000507 | ABA responsive complex |
| LEA 2 | ABRERATCAL | 52 | (+) | MACGYGB | S000507 | ABA responsive complex |
| LEA 2 | ABRERATCAL | 53 | (-) | MACGYGB | S000507 | ABA responsive complex |
| LEA 2 | ABRERATCAL | 551 | (+) | MACGYGB | S000507 | ABA responsive complex |
| LEA 2 | ABRERATCAL | 96 | (+) | MACGYGB | S000507 | ABA responsive complex |
| LEA 2 | ABRERATCAL | 168 | (-) | MACGYGB | S000507 | ABA responsive complex |
| LEA 2 | ABRERATCAL | 433 | (-) | MACGYGB | S000507 | ABA responsive complex |
| LEA 2 | ASF1MOTIFCAMV | 163 | (-) | TGACG | S000024 | Abiotic and biotic stress |
| LEA 2 | ASF1MOTIFCAMV | 181 | (-) | TGACG | S000024 | Abiotic and biotic stress |
| LEA 2 | ASF1MOTIFCAMV | 626 | (+) | TGACG | S000024 | Abiotic and biotic stress |
| LEA 2 | ASF1MOTIFCAMV | 186 | (-) | TGACG | S000024 | Abiotic and biotic stress |
| LEA 2 | BOXLCOREDCPAL | 513 | (-) | ACCWWCC | S000492 | MYB responsive |
| LEA 2 | BOXLCOREDCPAL | 334 | (+) | ACCWWCC | S000492 | MYB responsive |
| LEA 2 | BOXLCOREDCPAL | 53 | (+) | ACCWWCC | S000492 | MYB responsive |
| LEA 2 | BOXLCOREDCPAL | 144 | (+) | ACCWWCC | S000492 | MYB responsive |
| LEA 2 | BOXLCOREDCPAL | 337 | (+) | ACCWWCC | S000492 | MYB responsive |
| LEA 2 | BOXLCOREDCPAL | 448 | (-) | ACCWWCC | S000492 | MYB responsive |
| LEA 2 | BOXLCOREDCPAL | 218 | (+) | ACCWWCC | S000492 | MYB responsive |
| LEA 2 | BOXLCOREDCPAL | 41 | (+) | ACCWWCC | S000492 | MYB responsive |
| LEA 2 | LTRE1HVBLT49 | 604 | (-) | CCGAAA | S000250 | low temperature |
| LEA 2 | LTRE1HVBLT49 | 231 | (-) | CCGAAA | S000250 | low temperature |
| LEA 2 | LTRE1HVBLT49 | 596 | (-) | CCGAAA | S000250 | low temperature |
| LEA 2 | LTRE1HVBLT49 | 292 | (-) | CCGAAA | S000250 | low temperature |
| LEA 2 | LTRE1HVBLT49 | 443 | (-) | CCGAAA | S000250 | low temperature |
| LEA 2 | LTRE1HVBLT49 | 604 | (-) | CCGAAA | S000250 | low temperature |
| LEA 2 | LTRE1HVBLT49 | 759 | (+) | CCGAAA | S000250 | low temperature |
| LEA 2 | LTRE1HVBLT49 | 263 | (+) | CCGAAA | S000250 | low temperature |
| LEA 2 | LTRE1HVBLT49 | 445 | (-) | CCGAAA | S000250 | low temperature |
| LEA 2 | LTRE1HVBLT49 | 495 | (+) | CCGAAA | S000250 | low temperature |
| LEA 2 | LTRE1HVBLT49 | 956 | (-) | CCGAAA | S000250 | low temperature |
| LEA 2 | LTRE1HVBLT49 | 256 | (-) | CCGAAA | S000250 | low temperature |
| LEA 2 | LTRE1HVBLT49 | 740 | (-) | CCGAAA | S000250 | low temperature |
| LEA 2 | LTRECOREATCOR15 | 40 | (-) | CCGAC | S000153 | necessary for coldor drought |
| LEA 2 | LTRECOREATCOR15 | 52 | (-) | CCGAC | S000153 | necessary for coldor drought |
| LEA 2 | MYB1AT | 145 | (+) | WAACCA | S000408 | MYB recognition site |
| LEA 2 | MYB1AT | 195 | (-) | WAACCA | S000408 | MYB recognition site |
| LEA 2 | MYB1AT | 103 | (+) | WAACCA | S000408 | MYB recognition site |
| LEA 2 | MYB1AT | 145 | (-) | WAACCA | S000408 | MYB recognition site |
| LEA 2 | MYB1AT | 240 | (-) | WAACCA | S000408 | MYB recognition site |
| LEA 2 | MYB1AT | 87 | (-) | WAACCA | S000408 | MYB recognition site |
| LEA 2 | MYB1AT | 130 | (-) | WAACCA | S000408 | MYB recognition site |
| LEA 2 | MYBCORE | 939 | (+) | CNGTTR | S000176 | Dehydratio/water stress |
| LEA 2 | MYBCORE | 57 | (-) | CNGTTR | S000176 | Dehydratio/water stress |
| LEA 2 | MYBCORE | 478 | (-) | CNGTTR | S000176 | Dehydratio/water stress |
| LEA 2 | MYBCORE | 623 | (+) | CNGTTR | S000176 | Dehydratio/water stress |
| LEA 2 | MYBCORE | 63 | (-) | CNGTTR | S000176 | Dehydratio/water stress |
| LEA 2 | MYBCORE | 391 | (-) | CNGTTR | S000176 | Dehydratio/water stress |
| LEA 2 | MYBCORE | 138 | (-) | CNGTTR | S000176 | Dehydratio/water stress |
| LEA 2 | MYBCORE | 176 | (+) | CNGTTR | S000176 | Dehydratio/water stress |
| LEA 2 | MYBCORE | 632 | (+) | CNGTTR | S000176 | Dehydratio/water stress |
| LEA 2 | MYBCORE | 638 | (-) | CNGTTR | S000176 | Dehydratio/water stress |
| LEA 2 | MYBCORE | 510 | (-) | CNGTTR | S000176 | Dehydratio/water stress |
| LEA 2 | MYBCORE | 534 | (-) | CNGTTR | S000176 | Dehydratio/water stress |
| LEA 2 | MYBCORE | 389 | (-) | CNGTTR | S000176 | Dehydratio/water stress |
| LEA 2 | MYBCORE | 442 | (-) | CNGTTR | S000176 | Dehydratio/water stress |
| LEA 2 | MYBCORE | 372 | (+) | CNGTTR | S000176 | Dehydratio/water stress |
| LEA 2 | MYBCORE | 525 | (+) | CNGTTR | S000176 | Dehydratio/water stress |
| LEA 2 | MYBCORE | 554 | (+) | CNGTTR | S000176 | Dehydratio/water stress |
| LEA 2 | MYBCORE | 313 | (-) | CNGTTR | S000176 | Dehydratio/water stress |
| LEA 2 | MYBCORE | 513 | (+) | CNGTTR | S000176 | Dehydratio/water stress |
| LEA 2 | MYBCORE | 766 | (+) | CNGTTR | S000176 | Dehydratio/water stress |
| LEA 2 | MYBCORE | 785 | (-) | CNGTTR | S000176 | Dehydratio/water stress |
| LEA 2 | MYBCORE | 26 | (+) | CNGTTR | S000176 | Dehydratio/water stress |
| LEA 2 | MYBCORE | 482 | (+) | CNGTTR | S000176 | Dehydratio/water stress |
| LEA 2 | MYBCORE | 488 | (-) | CNGTTR | S000176 | Dehydratio/water stress |
| LEA 2 | MYBCORE | 448 | (-) | CNGTTR | S000176 | Dehydratio/water stress |
| LEA 2 | MYBCORE | 503 | (-) | CNGTTR | S000176 | Dehydratio/water stress |
| LEA 2 | MYBCORE | 258 | (+) | CNGTTR | S000176 | Dehydratio/water stress |
| LEA 2 | MYBCORE | 486 | (-) | CNGTTR | S000176 | Dehydratio/water stress |
| LEA 2 | MYBCORE | 562 | (+) | CNGTTR | S000176 | Dehydratio/water stress |
| LEA 2 | MYB1AT | 8 | (+) | WAACCA | S000408 | MYB recognition site |
| LEA 2 | MYB1AT | 64 | (-) | WAACCA | S000408 | MYB recognition site |
| LEA 2 | MYB1AT | 247 | (-) | WAACCA | S000408 | MYB recognition site |
| LEA 2 | MYBCORE | 918 | (-) | CNGTTR | S000176 | Dehydratio/water stress |
| LEA 2 | MYBCORE | 386 | (+) | CNGTTR | S000176 | Dehydratio/water stress |
| LEA 2 | ABRERATCAL | 158 | (+) | MACGYGB | S000507 | ABA responsive complex |
| LEA 2 | ABRERATCAL | 671 | (+) | MACGYGB | S000507 | ABA responsive complex |
| LEA 2 | ABRERATCAL | 597 | (+) | MACGYGB | S000507 | ABA responsive complex |
| LEA 2 | ABRERATCAL | 149 | (+) | MACGYGB | S000507 | ABA responsive complex |
| LEA 2 | ABRERATCAL | 153 | (-) | MACGYGB | S000507 | ABA responsive complex |
| LEA 2 | ASF1MOTIFCAMV | 379 | (+) | TGACG | S000024 | Abiotic and biotic stress |
| LEA 2 | BOXLCOREDCPAL | 331 | (+) | ACCWWCC | S000492 | MYB responsive |
| LEA 2 | BOXLCOREDCPAL | 442 | (-) | ACCWWCC | S000492 | MYB responsive |
| LEA 2 | BOXLCOREDCPAL | 485 | (-) | ACCWWCC | S000492 | MYB responsive |
| LEA 2 | LTRE1HVBLT49 | 347 | (-) | CCGAAA | S000250 | low temperature |
| LEA 2 | LTRECOREATCOR15 | 325 | (+) | CCGAC | S000153 | necessary for coldor drought |
| LEA 2 | MYB1AT | 261 | (+) | WAACCA | S000408 | MYB recognition site |
| LEA 2 | MYB1AT | 555 | (+) | WAACCA | S000408 | MYB recognition site |
| LEA 2 | MYBCORE | 568 | (+) | CNGTTR | S000176 | Dehydratio/water stress |
| LEA 2 | MYBCORE | 595 | (-) | CNGTTR | S000176 | Dehydratio/water stress |
| LEA 2 | MYBCORE | 451 | (+) | CNGTTR | S000176 | Dehydratio/water stress |
| LEA 2 | MYBCORE | 632 | (+) | CNGTTR | S000176 | Dehydratio/water stress |
| LEA 2 | MYBCORE | 646 | (+) | CNGTTR | S000176 | Dehydratio/water stress |
| LEA 2 | MYBCORE | 70 | (-) | CNGTTR | S000176 | Dehydratio/water stress |
| LEA 2 | MYB1AT | 749 | (+) | WAACCA | S000408 | MYB recognition site |
| LEA 2 | ABRERATCAL | 154 | (+) | MACGYGB | S000507 | ABA responsive complex |
| LEA 2 | ABRERATCAL | 471 | (-) | MACGYGB | S000507 | ABA responsive complex |
| LEA 2 | ABRERATCAL | 472 | (+) | MACGYGB | S000507 | ABA responsive complex |
| LEA 2 | ABRERATCAL | 251 | (+) | MACGYGB | S000507 | ABA responsive complex |
| LEA 2 | ABRERATCAL | 434 | (+) | MACGYGB | S000507 | ABA responsive complex |
| LEA 2 | ASF1MOTIFCAMV | 107 | (+) | TGACG | S000024 | Abiotic and biotic stress |
| LEA 2 | BOXLCOREDCPAL | 127 | (+) | ACCWWCC | S000492 | MYB responsive |
| LEA 2 | BOXLCOREDCPAL | 580 | (-) | ACCWWCC | S000492 | MYB responsive |
| LEA 2 | BOXLCOREDCPAL | 291 | (+) | ACCWWCC | S000492 | MYB responsive |
| LEA 2 | LTRE1HVBLT49 | 488 | (-) | CCGAAA | S000250 | low temperature |
| LEA 2 | LTRECOREATCOR15 | 269 | (+) | CCGAC | S000153 | necessary for coldor drought |
| LEA 2 | MYB1AT | 264 | (+) | WAACCA | S000408 | MYB recognition site |
| LEA 2 | MYB1AT | 8 | (+) | WAACCA | S000408 | MYB recognition site |
| LEA 2 | MYBCORE | 372 | (+) | CNGTTR | S000176 | Dehydratio/water stress |
| LEA 2 | MYBCORE | 510 | (-) | CNGTTR | S000176 | Dehydratio/water stress |
| LEA 2 | MYBCORE | 740 | (+) | CNGTTR | S000176 | Dehydratio/water stress |
| LEA 2 | MYBCORE | 167 | (-) | CNGTTR | S000176 | Dehydratio/water stress |
| LEA 2 | MYBCORE | 376 | (+) | CNGTTR | S000176 | Dehydratio/water stress |
| LEA 2 | MYBCORE | 552 | (+) | CNGTTR | S000176 | Dehydratio/water stress |
| LEA 2 | MYB1AT | 226 | (-) | WAACCA | S000408 | MYB recognition site |
| LEA 2 | ASF1MOTIFCAMV | 290 | (+) | TGACG | S000024 | Abiotic and biotic stress |
| LEA 2 | ASF1MOTIFCAMV | 87 | (+) | TGACG | S000024 | Abiotic and biotic stress |
| LEA 2 | ASF1MOTIFCAMV | 375 | (+) | TGACG | S000024 | Abiotic and biotic stress |
| LEA 2 | ASF1MOTIFCAMV | 722 | (-) | TGACG | S000024 | Abiotic and biotic stress |
| LEA 2 | ASF1MOTIFCAMV | 70 | (-) | TGACG | S000024 | Abiotic and biotic stress |
| LEA 2 | ASF1MOTIFCAMV | 299 | (-) | TGACG | S000024 | Abiotic and biotic stress |
| LEA 2 | ASF1MOTIFCAMV | 700 | (+) | TGACG | S000024 | Abiotic and biotic stress |
| LEA 2 | ASF1MOTIFCAMV | 725 | (-) | TGACG | S000024 | Abiotic and biotic stress |
| LEA 2 | ASF1MOTIFCAMV | 370 | (-) | TGACG | S000024 | Abiotic and biotic stress |
| LEA 2 | ASF1MOTIFCAMV | 272 | (-) | TGACG | S000024 | Abiotic and biotic stress |
| LEA 2 | ASF1MOTIFCAMV | 703 | (-) | TGACG | S000024 | Abiotic and biotic stress |
| LEA 2 | ASF1MOTIFCAMV | 379 | (-) | TGACG | S000024 | Abiotic and biotic stress |
| LEA 2 | ASF1MOTIFCAMV | 156 | (-) | TGACG | S000024 | Abiotic and biotic stress |
| LEA 2 | ASF1MOTIFCAMV | 73 | (-) | TGACG | S000024 | Abiotic and biotic stress |
| LEA 2 | ASF1MOTIFCAMV | 634 | (-) | TGACG | S000024 | Abiotic and biotic stress |
| LEA 2 | ASF1MOTIFCAMV | 186 | (-) | TGACG | S000024 | Abiotic and biotic stress |
| LEA 2 | ASF1MOTIFCAMV | 379 | (+) | TGACG | S000024 | Abiotic and biotic stress |
| LEA 2 | ASF1MOTIFCAMV | 145 | (+) | TGACG | S000024 | Abiotic and biotic stress |
| LEA 2 | ASF1MOTIFCAMV | 37 | (+) | TGACG | S000024 | Abiotic and biotic stress |
| LEA 2 | ASF1MOTIFCAMV | 189 | (-) | TGACG | S000024 | Abiotic and biotic stress |
| LEA 2 | ASF1MOTIFCAMV | 214 | (-) | TGACG | S000024 | Abiotic and biotic stress |
| LEA 2 | ASF1MOTIFCAMV | 574 | (+) | TGACG | S000024 | Abiotic and biotic stress |
| LEA 2 | BOXLCOREDCPAL | 105 | (+) | ACCWWCC | S000492 | MYB responsive |
| LEA 2 | BOXLCOREDCPAL | 1260 | (+) | ACCWWCC | S000492 | MYB responsive |
| LEA 2 | BOXLCOREDCPAL | 275 | (-) | ACCWWCC | S000492 | MYB responsive |
| LEA 2 | BOXLCOREDCPAL | 513 | (-) | ACCWWCC | S000492 | MYB responsive |
| LEA 2 | BOXLCOREDCPAL | 431 | (-) | ACCWWCC | S000492 | MYB responsive |
| LEA 2 | BOXLCOREDCPAL | 14 | (+) | ACCWWCC | S000492 | MYB responsive |
| LEA 2 | BOXLCOREDCPAL | 747 | (+) | ACCWWCC | S000492 | MYB responsive |
| LEA 2 | BOXLCOREDCPAL | 334 | (+) | ACCWWCC | S000492 | MYB responsive |
| LEA 2 | BOXLCOREDCPAL | 467 | (-) | ACCWWCC | S000492 | MYB responsive |
| LEA 2 | BOXLCOREDCPAL | 334 | (+) | ACCWWCC | S000492 | MYB responsive |
| LEA 2 | BOXLCOREDCPAL | 275 | (-) | ACCWWCC | S000492 | MYB responsive |
| LEA 2 | BOXLCOREDCPAL | 513 | (-) | ACCWWCC | S000492 | MYB responsive |
| LEA 2 | CBFHV | 37 | (-) | RYCGAC | S000497 | dehydration responsive element |
| LEA 2 | CBFHV | 37 | (+) | RYCGAC | S000497 | dehydration responsive element |
| LEA 2 | CBFHV | 122 | (-) | RYCGAC | S000497 | dehydration responsive element |
| LEA 2 | CBFHV | 122 | (+) | RYCGAC | S000497 | dehydration responsive element |
| LEA 2 | CBFHV | 496 | (+) | RYCGAC | S000497 | dehydration responsive element |
| LEA 2 | CBFHV | 413 | (-) | RYCGAC | S000497 | dehydration responsive element |
| LEA 2 | CBFHV | 413 | (+) | RYCGAC | S000497 | dehydration responsive element |
| LEA 2 | CBFHV | 881 | (-) | RYCGAC | S000497 | dehydration responsive element |
| LEA 2 | CBFHV | 881 | (+) | RYCGAC | S000497 | dehydration responsive element |
| LEA 2 | CBFHV | 385 | (-) | RYCGAC | S000497 | dehydration responsive element |
| LEA 2 | CBFHV | 385 | (+) | RYCGAC | S000497 | dehydration responsive element |
| LEA 2 | CBFHV | 553 | (-) | RYCGAC | S000497 | dehydration responsive element |
| LEA 2 | CBFHV | 389 | (-) | RYCGAC | S000497 | dehydration responsive element |
| LEA 2 | CBFHV | 389 | (+) | RYCGAC | S000497 | dehydration responsive element |
| LEA 2 | CBFHV | 668 | (-) | RYCGAC | S000497 | dehydration responsive element |
| LEA 2 | CBFHV | 465 | (-) | RYCGAC | S000497 | dehydration responsive element |
| LEA 2 | CBFHV | 428 | (-) | RYCGAC | S000497 | dehydration responsive element |
| LEA 2 | CBFHV | 590 | (-) | RYCGAC | S000497 | dehydration responsive element |
| LEA 2 | CBFHV | 688 | (+) | RYCGAC | S000497 | dehydration responsive element |
| LEA 2 | CBFHV | 3 | (-) | RYCGAC | S000497 | dehydration responsive element |
| LEA 2 | CBFHV | 64 | (-) | RYCGAC | S000497 | dehydration responsive element |
| LEA 2 | CBFHV | 521 | (-) | RYCGAC | S000497 | dehydration responsive element |
| LEA 2 | CBFHV | 26 | (-) | RYCGAC | S000497 | dehydration responsive element |
| LEA 2 | CBFHV | 422 | (-) | RYCGAC | S000497 | dehydration responsive element |
| LEA 2 | CBFHV | 422 | (+) | RYCGAC | S000497 | dehydration responsive element |
| LEA 2 | CBFHV | 392 | (-) | RYCGAC | S000497 | dehydration responsive element |
| LEA 2 | CBFHV | 600 | (+) | RYCGAC | S000497 | dehydration responsive element |
| LEA 2 | CBFHV | 777 | (-) | RYCGAC | S000497 | dehydration responsive element |
| LEA 2 | CBFHV | 3 | (-) | RYCGAC | S000497 | dehydration responsive element |
| LEA 2 | CBFHV | 449 | (-) | RYCGAC | S000497 | dehydration responsive element |
| LEA 2 | CBFHV | 227 | (+) | RYCGAC | S000497 | dehydration responsive element |
| LEA 2 | CBFHV | 3 | (-) | RYCGAC | S000497 | dehydration responsive element |
| LEA 2 | CBFHV | 33 | (-) | RYCGAC | S000497 | dehydration responsive element |
| LEA 2 | CBFHV | 451 | (-) | RYCGAC | S000497 | dehydration responsive element |
| LEA 2 | CBFHV | 443 | (-) | RYCGAC | S000497 | dehydration responsive element |
| LEA 2 | CBFHV | 443 | (+) | RYCGAC | S000497 | dehydration responsive element |
| LEA 2 | CBFHV | 314 | (+) | RYCGAC | S000497 | dehydration responsive element |
| LEA 2 | CBFHV | 527 | (+) | RYCGAC | S000497 | dehydration responsive element |
| LEA 2 | CBFHV | 270 | (-) | RYCGAC | S000497 | dehydration responsive element |
| LEA 2 | CBFHV | 319 | (+) | RYCGAC | S000497 | dehydration responsive element |
| LEA 2 | CBFHV | 78 | (-) | RYCGAC | S000497 | dehydration responsive element |
| LEA 2 | CBFHV | 3 | (-) | RYCGAC | S000497 | dehydration responsive element |
| LEA 2 | CBFHV | 449 | (-) | RYCGAC | S000497 | dehydration responsive element |
| LEA 2 | CBFHV | 331 | (-) | RYCGAC | S000497 | dehydration responsive element |
| LEA 2 | CBFHV | 600 | (+) | RYCGAC | S000497 | dehydration responsive element |
| LEA 2 | CBFHV | 777 | (-) | RYCGAC | S000497 | dehydration responsive element |
| LEA 2 | CBFHV | 52 | (-) | RYCGAC | S000497 | dehydration responsive element |
| LEA 2 | CBFHV | 324 | (+) | RYCGAC | S000497 | dehydration responsive element |
| LEA 2 | CBFHV | 319 | (-) | RYCGAC | S000497 | dehydration responsive element |
| LEA 2 | CBFHV | 319 | (+) | RYCGAC | S000497 | dehydration responsive element |
| LEA 2 | CBFHV | 361 | (-) | RYCGAC | S000497 | dehydration responsive element |
| LEA 2 | CBFHV | 446 | (-) | RYCGAC | S000497 | dehydration responsive element |
| LEA 2 | LTRE1HVBLT49 | 287 | (-) | CCGAAA | S000250 | low temperature |
| LEA 2 | LTRE1HVBLT49 | 377 | (+) | CCGAAA | S000250 | low temperature |
| LEA 2 | LTRE1HVBLT49 | 448 | (-) | CCGAAA | S000250 | low temperature |
| LEA 2 | LTRE1HVBLT49 | 256 | (-) | CCGAAA | S000250 | low temperature |
| LEA 2 | LTRE1HVBLT49 | 124 | (-) | CCGAAA | S000250 | low temperature |
| LEA 2 | LTRE1HVBLT49 | 495 | (+) | CCGAAA | S000250 | low temperature |
| LEA 2 | LTRE1HVBLT49 | 344 | (-) | CCGAAA | S000250 | low temperature |
| LEA 2 | LTRE1HVBLT49 | 485 | (-) | CCGAAA | S000250 | low temperature |
| LEA 2 | LTRE1HVBLT49 | 580 | (-) | CCGAAA | S000250 | low temperature |
| LEA 2 | LTRE1HVBLT49 | 387 | (+) | CCGAAA | S000250 | low temperature |
| LEA 2 | LTRE1HVBLT49 | 347 | (-) | CCGAAA | S000250 | low temperature |
| LEA 2 | LTRE1HVBLT49 | 197 | (+) | CCGAAA | S000250 | low temperature |
| LEA 2 | LTRE1HVBLT49 | 338 | (-) | CCGAAA | S000250 | low temperature |
| LEA 2 | LTRE1HVBLT49 | 347 | (-) | CCGAAA | S000250 | low temperature |
| LEA 2 | LTRE1HVBLT49 | 231 | (-) | CCGAAA | S000250 | low temperature |
| LEA 2 | LTRE1HVBLT49 | 810 | (-) | CCGAAA | S000250 | low temperature |
| LEA 2 | LTRE1HVBLT49 | 443 | (-) | CCGAAA | S000250 | low temperature |
| LEA 2 | LTRE1HVBLT49 | 12 | (-) | CCGAAA | S000250 | low temperature |
| LEA 2 | LTRE1HVBLT49 | 121 | (-) | CCGAAA | S000250 | low temperature |
| LEA 2 | LTRE1HVBLT49 | 555 | (+) | CCGAAA | S000250 | low temperature |
| LEA 2 | LTRE1HVBLT49 | 124 | (-) | CCGAAA | S000250 | low temperature |
| LEA 2 | LTRE1HVBLT49 | 67 | (-) | CCGAAA | S000250 | low temperature |
| LEA 2 | LTRE1HVBLT49 | 377 | (+) | CCGAAA | S000250 | low temperature |
| LEA 2 | LTRE1HVBLT49 | 621 | (-) | CCGAAA | S000250 | low temperature |
| LEA 2 | LTRE1HVBLT49 | 232 | (+) | CCGAAA | S000250 | low temperature |
| LEA 2 | LTRE1HVBLT49 | 216 | (-) | CCGAAA | S000250 | low temperature |
| LEA 2 | LTRE1HVBLT49 | 338 | (-) | CCGAAA | S000250 | low temperature |
| LEA 2 | LTRE1HVBLT49 | 474 | (-) | CCGAAA | S000250 | low temperature |
| LEA 2 | LTRE1HVBLT49 | 66 | (-) | CCGAAA | S000250 | low temperature |
| LEA 2 | LTRE1HVBLT49 | 119 | (-) | CCGAAA | S000250 | low temperature |
| LEA 2 | LTRE1HVBLT49 | 491 | (-) | CCGAAA | S000250 | low temperature |
| LEA 2 | LTRE1HVBLT49 | 623 | (+) | CCGAAA | S000250 | low temperature |
| LEA 2 | LTRE1HVBLT49 | 207 | (+) | CCGAAA | S000250 | low temperature |
| LEA 2 | LTRE1HVBLT49 | 780 | (-) | CCGAAA | S000250 | low temperature |
| LEA 2 | LTRE1HVBLT49 | 461 | (-) | CCGAAA | S000250 | low temperature |
| LEA 2 | LTRE1HVBLT49 | 309 | (-) | CCGAAA | S000250 | low temperature |
| LEA 2 | LTRE1HVBLT49 | 318 | (-) | CCGAAA | S000250 | low temperature |
| LEA 2 | LTRE1HVBLT49 | 214 | (+) | CCGAAA | S000250 | low temperature |
| LEA 2 | LTRE1HVBLT49 | 474 | (-) | CCGAAA | S000250 | low temperature |
| LEA 2 | LTRE1HVBLT49 | 528 | (-) | CCGAAA | S000250 | low temperature |
| LEA 2 | LTRE1HVBLT49 | 555 | (+) | CCGAAA | S000250 | low temperature |
| LEA 2 | LTRE1HVBLT49 | 35 | (-) | CCGAAA | S000250 | low temperature |
| LEA 2 | LTRE1HVBLT49 | 116 | (-) | CCGAAA | S000250 | low temperature |
| LEA 2 | LTRE1HVBLT49 | 298 | (-) | CCGAAA | S000250 | low temperature |
| LEA 2 | LTRE1HVBLT49 | 67 | (-) | CCGAAA | S000250 | low temperature |
| LEA 2 | LTRE1HVBLT49 | 12 | (-) | CCGAAA | S000250 | low temperature |
| LEA 2 | LTRE1HVBLT49 | 121 | (-) | CCGAAA | S000250 | low temperature |
| LEA 2 | LTRE1HVBLT49 | 238 | (-) | CCGAAA | S000250 | low temperature |
| LEA 2 | LTRE1HVBLT49 | 373 | (-) | CCGAAA | S000250 | low temperature |
| LEA 2 | LTRE1HVBLT49 | 317 | (-) | CCGAAA | S000250 | low temperature |
| LEA 2 | LTRE1HVBLT49 | 461 | (-) | CCGAAA | S000250 | low temperature |
| LEA 2 | LTRE1HVBLT49 | 318 | (-) | CCGAAA | S000250 | low temperature |
| LEA 2 | LTRE1HVBLT49 | 309 | (-) | CCGAAA | S000250 | low temperature |
| LEA 2 | LTRE1HVBLT49 | 387 | (-) | CCGAAA | S000250 | low temperature |
| LEA 2 | LTRE1HVBLT49 | 780 | (-) | CCGAAA | S000250 | low temperature |
| LEA 2 | LTRE1HVBLT49 | 621 | (-) | CCGAAA | S000250 | low temperature |
| LEA 2 | LTREATLTI78 | 552 | (-) | ACCGACA | S000157 | low temperature |
| LEA 2 | LTREATLTI78 | 2 | (-) | ACCGACA | S000157 | low temperature |
| LEA 2 | LTREATLTI78 | 2 | (-) | ACCGACA | S000157 | low temperature |
| LEA 2 | LTREATLTI78 | 564 | (-) | ACCGACA | S000157 | low temperature |
| LEA 2 | LTREATLTI78 | 312 | (+) | ACCGACA | S000157 | low temperature |
| LEA 2 | LTREATLTI78 | 181 | (+) | ACCGACA | S000157 | low temperature |
| LEA 2 | LTREATLTI78 | 564 | (-) | ACCGACA | S000157 | low temperature |
| LEA 2 | LTRECOREATCOR15 | 3 | (-) | CCGAC | S000153 | necessary for coldor drought |
| LEA 2 | LTRECOREATCOR15 | 330 | (+) | CCGAC | S000153 | necessary for coldor drought |
| LEA 2 | LTRECOREATCOR15 | 361 | (-) | CCGAC | S000153 | necessary for coldor drought |
| LEA 2 | LTRECOREATCOR15 | 3 | (-) | CCGAC | S000153 | necessary for coldor drought |
| LEA 2 | LTRECOREATCOR15 | 78 | (-) | CCGAC | S000153 | necessary for coldor drought |
| LEA 2 | LTRECOREATCOR15 | 314 | (+) | CCGAC | S000153 | necessary for coldor drought |
| LEA 2 | LTRECOREATCOR15 | 655 | (-) | CCGAC | S000153 | necessary for coldor drought |
| LEA 2 | LTRECOREATCOR15 | 217 | (+) | CCGAC | S000153 | necessary for coldor drought |
| LEA 2 | LTRECOREATCOR15 | 3 | (-) | CCGAC | S000153 | necessary for coldor drought |
| LEA 2 | LTRECOREATCOR15 | 449 | (-) | CCGAC | S000153 | necessary for coldor drought |
| LEA 2 | LTRECOREATCOR15 | 585 | (-) | CCGAC | S000153 | necessary for coldor drought |
| LEA 2 | LTRECOREATCOR15 | 228 | (+) | CCGAC | S000153 | necessary for coldor drought |
| LEA 2 | LTRECOREATCOR15 | 239 | (-) | CCGAC | S000153 | necessary for coldor drought |
| LEA 2 | LTRECOREATCOR15 | 40 | (-) | CCGAC | S000153 | necessary for coldor drought |
| LEA 2 | LTRECOREATCOR15 | 52 | (-) | CCGAC | S000153 | necessary for coldor drought |
| LEA 2 | LTRECOREATCOR15 | 325 | (+) | CCGAC | S000153 | necessary for coldor drought |
| LEA 2 | LTRECOREATCOR15 | 175 | (-) | CCGAC | S000153 | necessary for coldor drought |
| LEA 2 | MYB1AT | 503 | (-) | WAACCA | S000408 | MYB recognition site |
| LEA 2 | MYB1AT | 158 | (+) | WAACCA | S000408 | MYB recognition site |
| LEA 2 | MYB1AT | 293 | (+) | WAACCA | S000408 | MYB recognition site |
| LEA 2 | MYB1AT | 194 | (+) | WAACCA | S000408 | MYB recognition site |
| LEA 2 | MYB1AT | 131 | (+) | WAACCA | S000408 | MYB recognition site |
| LEA 2 | MYB1AT | 296 | (+) | WAACCA | S000408 | MYB recognition site |
| LEA 2 | MYB1AT | 136 | (-) | WAACCA | S000408 | MYB recognition site |
| LEA 2 | MYB1AT | 231 | (+) | WAACCA | S000408 | MYB recognition site |
| LEA 2 | MYB1AT | 299 | (+) | WAACCA | S000408 | MYB recognition site |
| LEA 2 | MYB1AT | 15 | (+) | WAACCA | S000408 | MYB recognition site |
| LEA 2 | MYB1AT | 208 | (+) | WAACCA | S000408 | MYB recognition site |
| LEA 2 | MYB1AT | 452 | (-) | WAACCA | S000408 | MYB recognition site |
| LEA 2 | MYB1AT | 123 | (+) | WAACCA | S000408 | MYB recognition site |
| LEA 2 | MYB1AT | 503 | (-) | WAACCA | S000408 | MYB recognition site |
| LEA 2 | MYB1AT | 744 | (-) | WAACCA | S000408 | MYB recognition site |
| LEA 2 | MYB1AT | 34 | (+) | WAACCA | S000408 | MYB recognition site |
| LEA 2 | MYB1AT | 139 | (-) | WAACCA | S000408 | MYB recognition site |
| LEA 2 | MYB1AT | 269 | (+) | WAACCA | S000408 | MYB recognition site |
| LEA 2 | MYB1AT | 1481 | (+) | WAACCA | S000408 | MYB recognition site |
| LEA 2 | MYB1AT | 54 | (+) | WAACCA | S000408 | MYB recognition site |
| LEA 2 | MYB1AT | 437 | (-) | WAACCA | S000408 | MYB recognition site |
| LEA 2 | MYB1AT | 868 | (-) | WAACCA | S000408 | MYB recognition site |
| LEA 2 | MYB1AT | 161 | (-) | WAACCA | S000408 | MYB recognition site |
| LEA 2 | MYB1AT | 453 | (-) | WAACCA | S000408 | MYB recognition site |
| LEA 2 | MYB1AT | 136 | (-) | WAACCA | S000408 | MYB recognition site |
| LEA 2 | MYB1AT | 208 | (-) | WAACCA | S000408 | MYB recognition site |
| LEA 2 | MYB1AT | 308 | (+) | WAACCA | S000408 | MYB recognition site |
| LEA 2 | MYB1AT | 8 | (+) | WAACCA | S000408 | MYB recognition site |
| LEA 2 | MYB1AT | 299 | (+) | WAACCA | S000408 | MYB recognition site |
| LEA 2 | MYB1AT | 216 | (+) | WAACCA | S000408 | MYB recognition site |
| LEA 2 | MYB1AT | 574 | (-) | WAACCA | S000408 | MYB recognition site |
| LEA 2 | MYB1AT | 264 | (+) | WAACCA | S000408 | MYB recognition site |
| LEA 2 | MYB1AT | 792 | (-) | WAACCA | S000408 | MYB recognition site |
| LEA 2 | MYB1AT | 1051 | (-) | WAACCA | S000408 | MYB recognition site |
| LEA 2 | MYB1AT | 158 | (+) | WAACCA | S000408 | MYB recognition site |
| LEA 2 | MYB1AT | 293 | (+) | WAACCA | S000408 | MYB recognition site |
| LEA 2 | MYB1AT | 146 | (+) | WAACCA | S000408 | MYB recognition site |
| LEA 2 | MYB1AT | 581 | (-) | WAACCA | S000408 | MYB recognition site |
| LEA 2 | MYB1AT | 145 | (-) | WAACCA | S000408 | MYB recognition site |
| LEA 2 | MYB1AT | 103 | (+) | WAACCA | S000408 | MYB recognition site |
| LEA 2 | MYB1AT | 54 | (+) | WAACCA | S000408 | MYB recognition site |
| LEA 2 | MYB1AT | 437 | (-) | WAACCA | S000408 | MYB recognition site |
| LEA 2 | MYB1AT | 517 | (-) | WAACCA | S000408 | MYB recognition site |
| LEA 2 | MYB1AT | 214 | (-) | WAACCA | S000408 | MYB recognition site |
| LEA 2 | MYB1AT | 373 | (-) | WAACCA | S000408 | MYB recognition site |
| LEA 2 | MYB1AT | 136 | (-) | WAACCA | S000408 | MYB recognition site |
| LEA 2 | MYB1AT | 208 | (-) | WAACCA | S000408 | MYB recognition site |
| LEA 2 | MYB1AT | 161 | (-) | WAACCA | S000408 | MYB recognition site |
| LEA 2 | MYB1AT | 453 | (-) | WAACCA | S000408 | MYB recognition site |
| LEA 2 | MYB1AT | 541 | (+) | WAACCA | S000408 | MYB recognition site |
| LEA 2 | MYB1AT | 2 | (-) | WAACCA | S000408 | MYB recognition site |
| LEA 2 | MYB1AT | 35 | (-) | WAACCA | S000408 | MYB recognition site |
| LEA 2 | MYB1AT | 159 | (-) | WAACCA | S000408 | MYB recognition site |
| LEA 2 | MYB1AT | 54 | (+) | WAACCA | S000408 | MYB recognition site |
| LEA 2 | MYB1AT | 437 | (-) | WAACCA | S000408 | MYB recognition site |
| LEA 2 | MYBAT | 525 | (-) | TAACTG | S000177 | MYB recognition site |
| LEA 2 | MYBAT | 700 | (+) | TAACTG | S000177 | MYB recognition site |
| LEA 2 | MYBAT | 294 | (-) | TAACTG | S000177 | MYB recognition site |
| LEA 2 | MYBAT | 332 | (+) | TAACTG | S000177 | MYB recognition site |
| LEA 2 | MYBAT | 733 | (+) | TAACTG | S000177 | MYB recognition site |
| LEA 2 | MYBAT | 13 | (-) | TAACTG | S000177 | MYB recognition site |
| LEA 2 | MYBAT | 525 | (-) | TAACTG | S000177 | MYB recognition site |
| LEA 2 | MYBCORE | 680 | (+) | CNGTTR | S000176 | Dehydratio/water stress |
| LEA 2 | MYBCORE | 292 | (+) | CNGTTR | S000176 | Dehydratio/water stress |
| LEA 2 | MYBCORE | 412 | (-) | CNGTTR | S000176 | Dehydratio/water stress |
| LEA 2 | MYBCORE | 301 | (+) | CNGTTR | S000176 | Dehydratio/water stress |
| LEA 2 | MYBCORE | 339 | (-) | CNGTTR | S000176 | Dehydratio/water stress |
| LEA 2 | MYBCORE | 617 | (-) | CNGTTR | S000176 | Dehydratio/water stress |
| LEA 2 | MYBCORE | 64 | (+) | CNGTTR | S000176 | Dehydratio/water stress |
| LEA 2 | MYBCORE | 572 | (-) | CNGTTR | S000176 | Dehydratio/water stress |
| LEA 2 | MYBCORE | 598 | (+) | CNGTTR | S000176 | Dehydratio/water stress |
| LEA 2 | MYBCORE | 484 | (+) | CNGTTR | S000176 | Dehydratio/water stress |
| LEA 2 | MYBCORE | 513 | (-) | CNGTTR | S000176 | Dehydratio/water stress |
| LEA 2 | MYBCORE | 601 | (+) | CNGTTR | S000176 | Dehydratio/water stress |
| LEA 2 | MYBCORE | 323 | (+) | CNGTTR | S000176 | Dehydratio/water stress |
| LEA 2 | MYBCORE | 329 | (+) | CNGTTR | S000176 | Dehydratio/water stress |
| LEA 2 | MYBCORE | 724 | (+) | CNGTTR | S000176 | Dehydratio/water stress |
| LEA 2 | MYBCORE | 113 | (+) | CNGTTR | S000176 | Dehydratio/water stress |
| LEA 2 | MYBCORE | 330 | (-) | CNGTTR | S000176 | Dehydratio/water stress |
| LEA 2 | MYBCORE | 514 | (+) | CNGTTR | S000176 | Dehydratio/water stress |
| LEA 2 | MYBCORE | 313 | (-) | CNGTTR | S000176 | Dehydratio/water stress |
| LEA 2 | MYBCORE | 513 | (+) | CNGTTR | S000176 | Dehydratio/water stress |
| LEA 2 | MYBCORE | 522 | (+) | CNGTTR | S000176 | Dehydratio/water stress |
| LEA 2 | MYBCORE | 669 | (+) | CNGTTR | S000176 | Dehydratio/water stress |
| LEA 2 | MYBCORE | 727 | (-) | CNGTTR | S000176 | Dehydratio/water stress |
| LEA 2 | MYBCORE | 577 | (-) | CNGTTR | S000176 | Dehydratio/water stress |
| LEA 2 | MYBCORE | 840 | (+) | CNGTTR | S000176 | Dehydratio/water stress |
| LEA 2 | MYBCORE | 856 | (+) | CNGTTR | S000176 | Dehydratio/water stress |
| LEA 2 | MYBCORE | 268 | (-) | CNGTTR | S000176 | Dehydratio/water stress |
| LEA 2 | MYBCORE | 445 | (+) | CNGTTR | S000176 | Dehydratio/water stress |
| LEA 2 | MYBCORE | 526 | (+) | CNGTTR | S000176 | Dehydratio/water stress |
| LEA 2 | MYBCORE | 124 | (+) | CNGTTR | S000176 | Dehydratio/water stress |
| LEA 2 | MYBCORE | 225 | (+) | CNGTTR | S000176 | Dehydratio/water stress |
| LEA 2 | MYBCORE | 301 | (+) | CNGTTR | S000176 | Dehydratio/water stress |
| LEA 2 | MYBCORE | 634 | (-) | CNGTTR | S000176 | Dehydratio/water stress |
| LEA 2 | MYBCORE | 529 | (-) | CNGTTR | S000176 | Dehydratio/water stress |
| LEA 2 | MYBCORE | 129 | (-) | CNGTTR | S000176 | Dehydratio/water stress |
| LEA 2 | MYBCORE | 162 | (+) | CNGTTR | S000176 | Dehydratio/water stress |
| LEA 2 | MYBCORE | 180 | (+) | CNGTTR | S000176 | Dehydratio/water stress |
| LEA 2 | MYBCORE | 535 | (-) | CNGTTR | S000176 | Dehydratio/water stress |
| LEA 2 | MYBCORE | 220 | (-) | CNGTTR | S000176 | Dehydratio/water stress |
| LEA 2 | MYBCORE | 343 | (+) | CNGTTR | S000176 | Dehydratio/water stress |
| LEA 2 | MYBCORE | 384 | (+) | CNGTTR | S000176 | Dehydratio/water stress |
| LEA 2 | MYBCORE | 567 | (+) | CNGTTR | S000176 | Dehydratio/water stress |
| LEA 2 | MYBCORE | 93 | (+) | CNGTTR | S000176 | Dehydratio/water stress |
| LEA 2 | MYBCORE | 211 | (-) | CNGTTR | S000176 | Dehydratio/water stress |
| LEA 2 | MYBCORE | 846 | (-) | CNGTTR | S000176 | Dehydratio/water stress |
| LEA 2 | MYBCORE | 984 | (-) | CNGTTR | S000176 | Dehydratio/water stress |
| LEA 2 | MYBCORE | 1052 | (-) | CNGTTR | S000176 | Dehydratio/water stress |
| LEA 2 | MYBCORE | 1161 | (+) | CNGTTR | S000176 | Dehydratio/water stress |
| LEA 2 | MYBCORE | 1315 | (+) | CNGTTR | S000176 | Dehydratio/water stress |
| LEA 2 | MYBCORE | 1354 | (-) | CNGTTR | S000176 | Dehydratio/water stress |
| LEA 2 | MYBCORE | 386 | (+) | CNGTTR | S000176 | Dehydratio/water stress |
| LEA 2 | MYBCORE | 568 | (+) | CNGTTR | S000176 | Dehydratio/water stress |
| LEA 2 | MYBCORE | 595 | (-) | CNGTTR | S000176 | Dehydratio/water stress |
| LEA 2 | MYBCORE | 175 | (+) | CNGTTR | S000176 | Dehydratio/water stress |
| LEA 2 | MYBCORE | 430 | (+) | CNGTTR | S000176 | Dehydratio/water stress |
| LEA 2 | MYBCORE | 311 | (+) | CNGTTR | S000176 | Dehydratio/water stress |
| LEA 2 | MYBCORE | 615 | (+) | CNGTTR | S000176 | Dehydratio/water stress |
| LEA 2 | MYBCORE | 629 | (+) | CNGTTR | S000176 | Dehydratio/water stress |
| LEA 2 | MYBCORE | 362 | (-) | CNGTTR | S000176 | Dehydratio/water stress |
| LEA 2 | MYBCORE | 415 | (-) | CNGTTR | S000176 | Dehydratio/water stress |
| LEA 2 | MYBCORE | 219 | (+) | CNGTTR | S000176 | Dehydratio/water stress |
| LEA 2 | MYBCORE | 232 | (-) | CNGTTR | S000176 | Dehydratio/water stress |
| LEA 2 | MYBCORE | 389 | (-) | CNGTTR | S000176 | Dehydratio/water stress |
| LEA 2 | MYBCORE | 470 | (+) | CNGTTR | S000176 | Dehydratio/water stress |
| LEA 2 | MYBCORE | 626 | (+) | CNGTTR | S000176 | Dehydratio/water stress |
| LEA 2 | MYBCORE | 136 | (-) | CNGTTR | S000176 | Dehydratio/water stress |
| LEA 2 | MYBCORE | 167 | (-) | CNGTTR | S000176 | Dehydratio/water stress |
| LEA 2 | MYBCORE | 409 | (-) | CNGTTR | S000176 | Dehydratio/water stress |
| LEA 2 | MYBCORE | 301 | (+) | CNGTTR | S000176 | Dehydratio/water stress |
| LEA 2 | MYBCORE | 339 | (-) | CNGTTR | S000176 | Dehydratio/water stress |
| LEA 2 | MYBCORE | 617 | (-) | CNGTTR | S000176 | Dehydratio/water stress |
| LEA 2 | MYBCORE | 292 | (+) | CNGTTR | S000176 | Dehydratio/water stress |
| LEA 2 | MYBCORE | 412 | (-) | CNGTTR | S000176 | Dehydratio/water stress |
| LEA 2 | MYBCORE | 39 | (+) | CNGTTR | S000176 | Dehydratio/water stress |
| LEA 2 | MYBCORE | 78 | (+) | CNGTTR | S000176 | Dehydratio/water stress |
| LEA 2 | MYBCORE | 188 | (-) | CNGTTR | S000176 | Dehydratio/water stress |
| LEA 2 | MYBCORE | 222 | (+) | CNGTTR | S000176 | Dehydratio/water stress |
| LEA 2 | MYBCORE | 453 | (+) | CNGTTR | S000176 | Dehydratio/water stress |
| LEA 2 | MYBCORE | 514 | (+) | CNGTTR | S000176 | Dehydratio/water stress |
| LEA 2 | MYBCORE | 185 | (-) | CNGTTR | S000176 | Dehydratio/water stress |
| LEA 2 | MYBCORE | 483 | (-) | CNGTTR | S000176 | Dehydratio/water stress |
| LEA 2 | MYBCORE | 70 | (-) | CNGTTR | S000176 | Dehydratio/water stress |
| LEA 2 | MYBCORE | 372 | (+) | CNGTTR | S000176 | Dehydratio/water stress |
| LEA 2 | MYBCORE | 344 | (-) | CNGTTR | S000176 | Dehydratio/water stress |
| LEA 2 | MYBCORE | 399 | (-) | CNGTTR | S000176 | Dehydratio/water stress |
| LEA 2 | MYBCORE | 232 | (-) | CNGTTR | S000176 | Dehydratio/water stress |
| LEA 2 | MYBCORE | 386 | (+) | CNGTTR | S000176 | Dehydratio/water stress |
| LEA 2 | MYBCORE | 70 | (-) | CNGTTR | S000176 | Dehydratio/water stress |
| LEA 2 | MYBCORE | 372 | (+) | CNGTTR | S000176 | Dehydratio/water stress |
| LEA 2 | MYBCORE | 510 | (-) | CNGTTR | S000176 | Dehydratio/water stress |
| LEA 2 | MYBCORE | 740 | (+) | CNGTTR | S000176 | Dehydratio/water stress |
| LEA 2 | MYBCORE | 148 | (+) | CNGTTR | S000176 | Dehydratio/water stress |
| LEA 2 | MYBCORE | 282 | (+) | CNGTTR | S000176 | Dehydratio/water stress |
| LEA 2 | MYBCORE | 797 | (-) | CNGTTR | S000176 | Dehydratio/water stress |
| LEA 2 | MYBCORE | 386 | (+) | CNGTTR | S000176 | Dehydratio/water stress |
| LEA 2 | MYBCORE | 568 | (+) | CNGTTR | S000176 | Dehydratio/water stress |
| LEA 2 | MYBCORE | 595 | (-) | CNGTTR | S000176 | Dehydratio/water stress |
| LEA 2 | MYBCORE | 389 | (+) | CNGTTR | S000176 | Dehydratio/water stress |
| LEA 2 | MYBCOREATCYCB1 | 62 | (+) | AACGG | S000502 | Dehydratio/water stress |
| LEA 2 | MYBCOREATCYCB1 | 502 | (+) | AACGG | S000502 | Dehydratio/water stress |
| LEA 2 | MYBCOREATCYCB1 | 348 | (-) | AACGG | S000502 | Dehydratio/water stress |
| LEA 2 | MYBCOREATCYCB1 | 388 | (+) | AACGG | S000502 | Dehydratio/water stress |
| LEA 2 | MYBCOREATCYCB1 | 588 | (-) | AACGG | S000502 | Dehydratio/water stress |
| LEA 2 | MYBCOREATCYCB1 | 606 | (+) | AACGG | S000502 | Dehydratio/water stress |
| LEA 2 | MYBCOREATCYCB1 | 626 | (-) | AACGG | S000502 | Dehydratio/water stress |
| LEA 2 | MYBCOREATCYCB1 | 215 | (+) | AACGG | S000502 | Dehydratio/water stress |
| LEA 2 | MYBCOREATCYCB1 | 207 | (-) | AACGG | S000502 | Dehydratio/water stress |
| LEA 2 | MYBCOREATCYCB1 | 352 | (+) | AACGG | S000502 | Dehydratio/water stress |
| LEA 2 | MYBCOREATCYCB1 | 647 | (-) | AACGG | S000502 | Dehydratio/water stress |
| LEA 2 | MYBCOREATCYCB1 | 106 | (-) | AACGG | S000502 | Dehydratio/water stress |
| LEA 2 | MYBCOREATCYCB1 | 542 | (-) | AACGG | S000502 | Dehydratio/water stress |
| LEA 2 | MYBCOREATCYCB1 | 191 | (+) | AACGG | S000502 | Dehydratio/water stress |
| LEA 2 | MYBCOREATCYCB1 | 461 | (-) | AACGG | S000502 | Dehydratio/water stress |
| LEA 2 | MYBCOREATCYCB1 | 496 | (+) | AACGG | S000502 | Dehydratio/water stress |
| LEA 2 | MYBCOREATCYCB1 | 634 | (-) | AACGG | S000502 | Dehydratio/water stress |
| LEA 2 | MYBCOREATCYCB1 | 702 | (-) | AACGG | S000502 | Dehydratio/water stress |
| LEA 2 | MYBCOREATCYCB1 | 725 | (+) | AACGG | S000502 | Dehydratio/water stress |
| LEA 2 | MYBCOREATCYCB1 | 740 | (+) | AACGG | S000502 | Dehydratio/water stress |
| LEA 2 | MYBCOREATCYCB1 | 410 | (+) | AACGG | S000502 | Dehydratio/water stress |
| LEA 2 | MYBCOREATCYCB1 | 538 | (-) | AACGG | S000502 | Dehydratio/water stress |
| LEA 2 | MYBCOREATCYCB1 | 71 | (+) | AACGG | S000502 | Dehydratio/water stress |
| LEA 2 | MYBCOREATCYCB1 | 95 | (+) | AACGG | S000502 | Dehydratio/water stress |
| LEA 2 | MYBCOREATCYCB1 | 262 | (-) | AACGG | S000502 | Dehydratio/water stress |
| LEA 2 | MYBCOREATCYCB1 | 476 | (-) | AACGG | S000502 | Dehydratio/water stress |
| LEA 2 | MYBCOREATCYCB1 | 571 | (-) | AACGG | S000502 | Dehydratio/water stress |
| LEA 2 | MYBCOREATCYCB1 | 242 | (+) | AACGG | S000502 | Dehydratio/water stress |
| LEA 2 | MYBCOREATCYCB1 | 6 | (-) | AACGG | S000502 | Dehydratio/water stress |
| LEA 2 | MYBCOREATCYCB1 | 119 | (-) | AACGG | S000502 | Dehydratio/water stress |
| LEA 2 | MYBCOREATCYCB1 | 435 | (-) | AACGG | S000502 | Dehydratio/water stress |
| LEA 2 | MYBCOREATCYCB1 | 526 | (-) | AACGG | S000502 | Dehydratio/water stress |
| LEA 2 | MYBCOREATCYCB1 | 392 | (+) | AACGG | S000502 | Dehydratio/water stress |
| LEA 2 | MYBCOREATCYCB1 | 392 | (+) | AACGG | S000502 | Dehydratio/water stress |
| LEA 2 | MYBCOREATCYCB1 | 35 | (+) | AACGG | S000502 | Dehydratio/water stress |
| LEA 2 | MYBCOREATCYCB1 | 313 | (+) | AACGG | S000502 | Dehydratio/water stress |
| LEA 2 | MYBCOREATCYCB1 | 135 | (-) | AACGG | S000502 | Dehydratio/water stress |
| LEA 2 | MYBCOREATCYCB1 | 414 | (+) | AACGG | S000502 | Dehydratio/water stress |
| LEA 2 | MYBCOREATCYCB1 | 424 | (-) | AACGG | S000502 | Dehydratio/water stress |
| LEA 2 | MYBCOREATCYCB1 | 519 | (-) | AACGG | S000502 | Dehydratio/water stress |
| LEA 2 | MYBCOREATCYCB1 | 191 | (+) | AACGG | S000502 | Dehydratio/water stress |
| LEA 2 | MYBCOREATCYCB1 | 461 | (-) | AACGG | S000502 | Dehydratio/water stress |
| LEA 2 | MYBCOREATCYCB1 | 496 | (+) | AACGG | S000502 | Dehydratio/water stress |
| LEA 2 | MYBCOREATCYCB1 | 634 | (-) | AACGG | S000502 | Dehydratio/water stress |
| LEA 2 | MYBCOREATCYCB1 | 702 | (-) | AACGG | S000502 | Dehydratio/water stress |
| LEA 2 | MYBCOREATCYCB1 | 725 | (+) | AACGG | S000502 | Dehydratio/water stress |
| LEA 2 | MYBCOREATCYCB1 | 740 | (+) | AACGG | S000502 | Dehydratio/water stress |
| LEA 2 | MYBCOREATCYCB1 | 62 | (+) | AACGG | S000502 | Dehydratio/water stress |
| LEA 2 | MYBCOREATCYCB1 | 108 | (+) | AACGG | S000502 | Dehydratio/water stress |
| LEA 2 | MYBCOREATCYCB1 | 512 | (+) | AACGG | S000502 | Dehydratio/water stress |
| LEA 2 | MYBCOREATCYCB1 | 652 | (-) | AACGG | S000502 | Dehydratio/water stress |
| LEA 2 | MYBCOREATCYCB1 | 582 | (+) | AACGG | S000502 | Dehydratio/water stress |
| LEA 2 | MYBCOREATCYCB1 | 600 | (-) | AACGG | S000502 | Dehydratio/water stress |
| LEA 2 | MYBCOREATCYCB1 | 119 | (+) | AACGG | S000502 | Dehydratio/water stress |
| LEA 2 | MYBCOREATCYCB1 | 573 | (+) | AACGG | S000502 | Dehydratio/water stress |
| LEA 2 | MYBCOREATCYCB1 | 173 | (-) | AACGG | S000502 | Dehydratio/water stress |
| LEA 2 | MYBCOREATCYCB1 | 401 | (-) | AACGG | S000502 | Dehydratio/water stress |
| LEA 2 | MYBCOREATCYCB1 | 515 | (+) | AACGG | S000502 | Dehydratio/water stress |
| LEA 2 | MYBCOREATCYCB1 | 105 | (-) | AACGG | S000502 | Dehydratio/water stress |
| LEA 2 | MYBCOREATCYCB1 | 218 | (+) | AACGG | S000502 | Dehydratio/water stress |
| LEA 2 | MYBCOREATCYCB1 | 88 | (+) | AACGG | S000502 | Dehydratio/water stress |
| LEA 2 | MYBCOREATCYCB1 | 269 | (+) | AACGG | S000502 | Dehydratio/water stress |
| LEA 2 | MYBCOREATCYCB1 | 174 | (-) | AACGG | S000502 | Dehydratio/water stress |
| LEA 2 | MYBCOREATCYCB1 | 243 | (-) | AACGG | S000502 | Dehydratio/water stress |
| LEA 2 | MYBCOREATCYCB1 | 542 | (+) | AACGG | S000502 | Dehydratio/water stress |
| LEA 2 | MYBCOREATCYCB1 | 655 | (+) | AACGG | S000502 | Dehydratio/water stress |
| LEA 2 | MYBCOREATCYCB1 | 567 | (+) | AACGG | S000502 | Dehydratio/water stress |
| LEA 2 | MYBCOREATCYCB1 | 371 | (+) | AACGG | S000502 | Dehydratio/water stress |
| LEA 2 | MYBCOREATCYCB1 | 666 | (-) | AACGG | S000502 | Dehydratio/water stress |
| LEA 2 | MYBCOREATCYCB1 | 383 | (+) | AACGG | S000502 | Dehydratio/water stress |
| LEA 2 | MYBCOREATCYCB1 | 573 | (+) | AACGG | S000502 | Dehydratio/water stress |
| LEA 2 | MYBCOREATCYCB1 | 119 | (-) | AACGG | S000502 | Dehydratio/water stress |
| LEA 2 | MYBCOREATCYCB1 | 36 | (+) | AACGG | S000502 | Dehydratio/water stress |
| LEA 2 | MYBCOREATCYCB1 | 62 | (+) | AACGG | S000502 | Dehydratio/water stress |
| LEA 2 | MYBCOREATCYCB1 | 108 | (+) | AACGG | S000502 | Dehydratio/water stress |
| LEA 2 | MYBCOREATCYCB1 | 512 | (+) | AACGG | S000502 | Dehydratio/water stress |
| LEA 2 | MYBCOREATCYCB1 | 652 | (-) | AACGG | S000502 | Dehydratio/water stress |
| LEA 2 | MYBCOREATCYCB1 | 292 | (+) | AACGG | S000502 | Dehydratio/water stress |
| LEA 2 | MYBCOREATCYCB1 | 491 | (-) | AACGG | S000502 | Dehydratio/water stress |
| LEA 2 | MYBCOREATCYCB1 | 25 | (-) | AACGG | S000502 | Dehydratio/water stress |
| LEA 2 | MYBCOREATCYCB1 | 300 | (-) | AACGG | S000502 | Dehydratio/water stress |
| LEA 2 | MYBCOREATCYCB1 | 174 | (-) | AACGG | S000502 | Dehydratio/water stress |
| LEA 2 | MYBCOREATCYCB1 | 243 | (-) | AACGG | S000502 | Dehydratio/water stress |
| LEA 2 | MYBCOREATCYCB1 | 542 | (+) | AACGG | S000502 | Dehydratio/water stress |
| LEA 2 | MYBCOREATCYCB1 | 371 | (+) | AACGG | S000502 | Dehydratio/water stress |
| LEA 2 | MYBCOREATCYCB1 | 666 | (-) | AACGG | S000502 | Dehydratio/water stress |
| LEA 2 | MYBCOREATCYCB1 | 162 | (-) | AACGG | S000502 | Dehydratio/water stress |
| LEA 2 | MYBCOREATCYCB1 | 36 | (+) | AACGG | S000502 | Dehydratio/water stress |
| LEA 2 | MYBAT | 376 | (-) | TAACTG | S000177 | MYB recognition site |
| LEA 2 | MYBAT | 134 | (+) | TAACTG | S000177 | MYB recognition site |
| LEA 2 | MYBAT | 466 | (-) | TAACTG | S000177 | MYB recognition site |
| LEA 2 | MYBAT | 35 | (-) | TAACTG | S000177 | MYB recognition site |
| LEA 2 | MYBAT | 629 | (-) | TAACTG | S000177 | MYB recognition site |
| LEA 2 | MYBAT | 737 | (+) | TAACTG | S000177 | MYB recognition site |
| LEA 2 | MYBCOREATCYCB1 | 618 | (-) | AACGG | S000502 | Dehydratio/water stress |
| LEA 2 | MYBCOREATCYCB1 | 119 | (-) | AACGG | S000502 | Dehydratio/water stress |
| LEA 2 | MYBCOREATCYCB1 | 119 | (+) | AACGG | S000502 | Dehydratio/water stress |
| LEA 2 | MYBCOREATCYCB1 | 573 | (+) | AACGG | S000502 | Dehydratio/water stress |
| LEA 2 | MYBCOREATCYCB1 | 173 | (-) | AACGG | S000502 | Dehydratio/water stress |
| LEA 2 | MYBCOREATCYCB1 | 401 | (-) | AACGG | S000502 | Dehydratio/water stress |
| LEA 2 | MYBCOREATCYCB1 | 292 | (+) | AACGG | S000502 | Dehydratio/water stress |
| LEA 2 | MYBCOREATCYCB1 | 491 | (-) | AACGG | S000502 | Dehydratio/water stress |
| LEA 2 | MYBCOREATCYCB1 | 112 | (-) | AACGG | S000502 | Dehydratio/water stress |
| LEA 2 | MYBCOREATCYCB1 | 219 | (-) | AACGG | S000502 | Dehydratio/water stress |
| LEA 2 | MYBCOREATCYCB1 | 488 | (-) | AACGG | S000502 | Dehydratio/water stress |
| LEA 2 | MYBCOREATCYCB1 | 589 | (-) | AACGG | S000502 | Dehydratio/water stress |
| LEA 2 | MYBCOREATCYCB1 | 324 | (+) | AACGG | S000502 | Dehydratio/water stress |
| LEA 2 | MYBCOREATCYCB1 | 69 | (-) | AACGG | S000502 | Dehydratio/water stress |
| LEA 2 | MYBCOREATCYCB1 | 597 | (-) | AACGG | S000502 | Dehydratio/water stress |
| LEA 2 | MYBCOREATCYCB1 | 383 | (+) | AACGG | S000502 | Dehydratio/water stress |
| LEA 2 | MYBCOREATCYCB1 | 573 | (+) | AACGG | S000502 | Dehydratio/water stress |
| LEA 2 | MYBCOREATCYCB1 | 689 | (+) | AACGG | S000502 | Dehydratio/water stress |
| LEA 2 | MYBCOREATCYCB1 | 414 | (+) | AACGG | S000502 | Dehydratio/water stress |
| LEA 2 | MYBCOREATCYCB1 | 184 | (-) | AACGG | S000502 | Dehydratio/water stress |
| LEA 2 | MYBCOREATCYCB1 | 680 | (+) | AACGG | S000502 | Dehydratio/water stress |
| LEA 2 | MYBCOREATCYCB1 | 221 | (+) | AACGG | S000502 | Dehydratio/water stress |
| LEA 2 | ABRERATCAL | 433 | (-) | MACGYGB | S000507 | ABA responsive complex |
| LEA 2 | ABRERATCAL | 597 | (+) | MACGYGB | S000507 | ABA responsive complex |
| LEA 2 | ABRERATCAL | 400 | (-) | MACGYGB | S000507 | ABA responsive complex |
| LEA 2 | ABRERATCAL | 153 | (-) | MACGYGB | S000507 | ABA responsive complex |
| LEA 2 | ABRERATCAL | 154 | (+) | MACGYGB | S000507 | ABA responsive complex |
| LEA 2 | ABRERATCAL | 471 | (-) | MACGYGB | S000507 | ABA responsive complex |
| LEA 2 | ABRERATCAL | 472 | (+) | MACGYGB | S000507 | ABA responsive complex |
| LEA 2 | ABRERATCAL | 557 | (+) | MACGYGB | S000507 | ABA responsive complex |
| LEA 2 | ABRERATCAL | 400 | (-) | MACGYGB | S000507 | ABA responsive complex |
| LEA 2 | ABRERATCAL | 96 | (+) | MACGYGB | S000507 | ABA responsive complex |
| LEA 2 | ABRERATCAL | 710 | (+) | MACGYGB | S000507 | ABA responsive complex |
| LEA 2 | ABRERATCAL | 251 | (+) | MACGYGB | S000507 | ABA responsive complex |
| LEA 2 | ABRERATCAL | 96 | (+) | MACGYGB | S000507 | ABA responsive complex |
| LEA 2 | ABRERATCAL | 557 | (+) | MACGYGB | S000507 | ABA responsive complex |
| LEA 2 | ABRERATCAL | 54 | (-) | MACGYGB | S000507 | ABA responsive complex |
| LEA 2 | ABRERATCAL | 380 | (+) | MACGYGB | S000507 | ABA responsive complex |
| LEA 2 | ABRERATCAL | 492 | (-) | MACGYGB | S000507 | ABA responsive complex |
| LEA 2 | ABRERATCAL | 493 | (+) | MACGYGB | S000507 | ABA responsive complex |
| LEA 2 | ABRERATCAL | 416 | (-) | MACGYGB | S000507 | ABA responsive complex |
| LEA 2 | ABRERATCAL | 437 | (-) | MACGYGB | S000507 | ABA responsive complex |
| LEA 2 | ABRERATCAL | 565 | (+) | MACGYGB | S000507 | ABA responsive complex |
| LEA 2 | ABRERATCAL | 566 | (-) | MACGYGB | S000507 | ABA responsive complex |
| LEA 2 | ABRERATCAL | 54 | (-) | MACGYGB | S000507 | ABA responsive complex |
| LEA 2 | ABRERATCAL | 492 | (-) | MACGYGB | S000507 | ABA responsive complex |
| LEA 2 | ABRERATCAL | 493 | (+) | MACGYGB | S000507 | ABA responsive complex |
| LEA 2 | ABRERATCAL | 96 | (+) | MACGYGB | S000507 | ABA responsive complex |
| LEA 2 | ABRERATCAL | 914 | (+) | MACGYGB | S000507 | ABA responsive complex |
| LEA 2 | ABRERATCAL | 312 | (-) | MACGYGB | S000507 | ABA responsive complex |
| LEA 2 | ABRERATCAL | 370 | (+) | MACGYGB | S000507 | ABA responsive complex |
| LEA 2 | ABRERATCAL | 149 | (+) | MACGYGB | S000507 | ABA responsive complex |
| LEA 2 | ABRERATCAL | 334 | (+) | MACGYGB | S000507 | ABA responsive complex |
| LEA 2 | ABRERATCAL | 596 | (-) | MACGYGB | S000507 | ABA responsive complex |
| LEA 2 | ABRERATCAL | 96 | (+) | MACGYGB | S000507 | ABA responsive complex |
| LEA 2 | ABREZMRAB8 | 471 | (-) | CCACGTGG | S000133 | ABA responsive complex |
| LEA 2 | ABREZMRAB8 | 471 | (+) | CCACGTGG | S000133 | ABA responsive complex |
| LEA 2 | ABREZMRAB8 | 471 | (-) | CCACGTGG | S000133 | ABA responsive complex |
| LEA 2 | ABREZMRAB8 | 471 | (+) | CCACGTGG | S000133 | ABA responsive complex |
| LEA 2 | ABREZMRAB8 | 492 | (-) | CCACGTGG | S000133 | ABA responsive complex |
| LEA 2 | ABREZMRAB8 | 492 | (+) | CCACGTGG | S000133 | ABA responsive complex |
| LEA 2 | ABREZMRAB8 | 492 | (-) | CCACGTGG | S000133 | ABA responsive complex |
| LEA 2 | ABREZMRAB8 | 492 | (+) | CCACGTGG | S000133 | ABA responsive complex |
| LEA 2 | ACGTABREMOTIFAOSEM | 580 | (+) | ACGTGKC | S000394 | interdependent in the ABA responsive expression of the rd29A |
| LEA 2 | ACGTABREMOTIFAOSEM | 381 | (+) | ACGTGKC | S000394 | interdependent in the ABA responsive expression of the rd29A |
| LEA 2 | ACGTABREMOTIFAOSEM | 435 | (+) | ACGTGKC | S000394 | interdependent in the ABA responsive expression of the rd29A |
| LEA 2 | ACGTABREMOTIFAOSEM | 436 | (-) | ACGTGKC | S000394 | interdependent in the ABA responsive expression of the rd29A |
| LEA 2 | ACGTABREMOTIFAOSEM | 592 | (+) | ACGTGKC | S000394 | interdependent in the ABA responsive expression of the rd29A |
| LEA 2 | ACGTATERD1 | 111 | (-) | ACGT | S000415 | early responsive to dehydration |
| LEA 2 | ACGTATERD1 | 111 | (+) | ACGT | S000415 | early responsive to dehydration |
| LEA 2 | ACGTATERD1 | 283 | (-) | ACGT | S000415 | early responsive to dehydration |
| LEA 2 | ACGTATERD1 | 283 | (+) | ACGT | S000415 | early responsive to dehydration |
| LEA 2 | ACGTATERD1 | 81 | (-) | ACGT | S000415 | early responsive to dehydration |
| LEA 2 | ACGTATERD1 | 81 | (+) | ACGT | S000415 | early responsive to dehydration |
| LEA 2 | ACGTATERD1 | 850 | (-) | ACGT | S000415 | early responsive to dehydration |
| LEA 2 | ACGTATERD1 | 850 | (+) | ACGT | S000415 | early responsive to dehydration |
| LEA 2 | ACGTATERD1 | 435 | (-) | ACGT | S000415 | early responsive to dehydration |
| LEA 2 | ACGTATERD1 | 435 | (+) | ACGT | S000415 | early responsive to dehydration |
| LEA 2 | ACGTATERD1 | 698 | (-) | ACGT | S000415 | early responsive to dehydration |
| LEA 2 | ACGTATERD1 | 698 | (+) | ACGT | S000415 | early responsive to dehydration |
| LEA 2 | ACGTATERD1 | 903 | (-) | ACGT | S000415 | early responsive to dehydration |
| LEA 2 | ACGTATERD1 | 903 | (+) | ACGT | S000415 | early responsive to dehydration |
| LEA 2 | ACGTATERD1 | 1006 | (-) | ACGT | S000415 | early responsive to dehydration |
| LEA 2 | ACGTATERD1 | 1006 | (+) | ACGT | S000415 | early responsive to dehydration |
| LEA 2 | ACGTATERD1 | 288 | (-) | ACGT | S000415 | early responsive to dehydration |
| LEA 2 | ACGTATERD1 | 288 | (+) | ACGT | S000415 | early responsive to dehydration |
| LEA 2 | ACGTATERD1 | 161 | (-) | ACGT | S000415 | early responsive to dehydration |
| LEA 2 | ACGTATERD1 | 161 | (+) | ACGT | S000415 | early responsive to dehydration |
| LEA 2 | ACGTATERD1 | 295 | (-) | ACGT | S000415 | early responsive to dehydration |
| LEA 2 | ACGTATERD1 | 295 | (+) | ACGT | S000415 | early responsive to dehydration |
| LEA 2 | ACGTATERD1 | 317 | (-) | ACGT | S000415 | early responsive to dehydration |
| LEA 2 | ACGTATERD1 | 317 | (+) | ACGT | S000415 | early responsive to dehydration |
| LEA 2 | ACGTATERD1 | 249 | (-) | ACGT | S000415 | early responsive to dehydration |
| LEA 2 | ACGTATERD1 | 249 | (+) | ACGT | S000415 | early responsive to dehydration |
| LEA 2 | ACGTATERD1 | 280 | (-) | ACGT | S000415 | early responsive to dehydration |
| LEA 2 | ACGTATERD1 | 280 | (+) | ACGT | S000415 | early responsive to dehydration |
| LEA 2 | ACGTATERD1 | 365 | (-) | ACGT | S000415 | early responsive to dehydration |
| LEA 2 | ACGTATERD1 | 365 | (+) | ACGT | S000415 | early responsive to dehydration |
| LEA 2 | ACGTATERD1 | 527 | (-) | ACGT | S000415 | early responsive to dehydration |
| LEA 2 | ACGTATERD1 | 527 | (+) | ACGT | S000415 | early responsive to dehydration |
| LEA 2 | ACGTATERD1 | 580 | (-) | ACGT | S000415 | early responsive to dehydration |
| LEA 2 | ACGTATERD1 | 580 | (+) | ACGT | S000415 | early responsive to dehydration |
| LEA 2 | ACGTATERD1 | 234 | (-) | ACGT | S000415 | early responsive to dehydration |
| LEA 2 | ACGTATERD1 | 234 | (+) | ACGT | S000415 | early responsive to dehydration |
| LEA 2 | ACGTATERD1 | 319 | (-) | ACGT | S000415 | early responsive to dehydration |
| LEA 2 | ACGTATERD1 | 319 | (+) | ACGT | S000415 | early responsive to dehydration |
| LEA 2 | ACGTATERD1 | 341 | (-) | ACGT | S000415 | early responsive to dehydration |
| LEA 2 | ACGTATERD1 | 341 | (+) | ACGT | S000415 | early responsive to dehydration |
| LEA 2 | ACGTATERD1 | 372 | (-) | ACGT | S000415 | early responsive to dehydration |
| LEA 2 | ACGTATERD1 | 372 | (+) | ACGT | S000415 | early responsive to dehydration |
| LEA 2 | ACGTATERD1 | 409 | (-) | ACGT | S000415 | early responsive to dehydration |
| LEA 2 | ACGTATERD1 | 409 | (+) | ACGT | S000415 | early responsive to dehydration |
| LEA 2 | ACGTATERD1 | 414 | (-) | ACGT | S000415 | early responsive to dehydration |
| LEA 2 | ACGTATERD1 | 414 | (+) | ACGT | S000415 | early responsive to dehydration |
| LEA 2 | ACGTATERD1 | 430 | (-) | ACGT | S000415 | early responsive to dehydration |
| LEA 2 | ACGTATERD1 | 430 | (+) | ACGT | S000415 | early responsive to dehydration |
| LEA 2 | ACGTATERD1 | 500 | (-) | ACGT | S000415 | early responsive to dehydration |
| LEA 2 | ACGTATERD1 | 500 | (+) | ACGT | S000415 | early responsive to dehydration |
| LEA 2 | ACGTATERD1 | 112 | (-) | ACGT | S000415 | early responsive to dehydration |
| LEA 2 | ACGTATERD1 | 112 | (+) | ACGT | S000415 | early responsive to dehydration |
| LEA 2 | ACGTATERD1 | 365 | (-) | ACGT | S000415 | early responsive to dehydration |
| LEA 2 | ACGTATERD1 | 365 | (+) | ACGT | S000415 | early responsive to dehydration |
| LEA 2 | ACGTATERD1 | 396 | (-) | ACGT | S000415 | early responsive to dehydration |
| LEA 2 | ACGTATERD1 | 396 | (+) | ACGT | S000415 | early responsive to dehydration |
| LEA 2 | ACGTATERD1 | 534 | (-) | ACGT | S000415 | early responsive to dehydration |
| LEA 2 | ACGTATERD1 | 534 | (+) | ACGT | S000415 | early responsive to dehydration |
| LEA 2 | ACGTATERD1 | 539 | (-) | ACGT | S000415 | early responsive to dehydration |
| LEA 2 | ACGTATERD1 | 539 | (+) | ACGT | S000415 | early responsive to dehydration |
| LEA 2 | ACGTATERD1 | 689 | (-) | ACGT | S000415 | early responsive to dehydration |
| LEA 2 | ACGTATERD1 | 689 | (+) | ACGT | S000415 | early responsive to dehydration |
| LEA 2 | ACGTATERD1 | 452 | (-) | ACGT | S000415 | early responsive to dehydration |
| LEA 2 | ACGTATERD1 | 452 | (+) | ACGT | S000415 | early responsive to dehydration |
| LEA 2 | ACGTATERD1 | 552 | (-) | ACGT | S000415 | early responsive to dehydration |
| LEA 2 | ACGTATERD1 | 552 | (+) | ACGT | S000415 | early responsive to dehydration |
| LEA 2 | ACGTATERD1 | 552 | (-) | ACGT | S000415 | early responsive to dehydration |
| LEA 2 | ACGTATERD1 | 552 | (+) | ACGT | S000415 | early responsive to dehydration |
| LEA 2 | ACGTATERD1 | 666 | (-) | ACGT | S000415 | early responsive to dehydration |
| LEA 2 | ACGTATERD1 | 666 | (+) | ACGT | S000415 | early responsive to dehydration |
| LEA 2 | ACGTATERD1 | 81 | (-) | ACGT | S000415 | early responsive to dehydration |
| LEA 2 | ACGTATERD1 | 81 | (+) | ACGT | S000415 | early responsive to dehydration |
| LEA 2 | ACGTATERD1 | 97 | (-) | ACGT | S000415 | early responsive to dehydration |
| LEA 2 | ACGTATERD1 | 97 | (+) | ACGT | S000415 | early responsive to dehydration |
| LEA 2 | ACGTATERD1 | 129 | (-) | ACGT | S000415 | early responsive to dehydration |
| LEA 2 | ACGTATERD1 | 129 | (+) | ACGT | S000415 | early responsive to dehydration |
| LEA 2 | ACGTATERD1 | 14 | (-) | ACGT | S000415 | early responsive to dehydration |
| LEA 2 | ACGTATERD1 | 14 | (+) | ACGT | S000415 | early responsive to dehydration |
| LEA 2 | ACGTATERD1 | 47 | (-) | ACGT | S000415 | early responsive to dehydration |
| LEA 2 | ACGTATERD1 | 47 | (+) | ACGT | S000415 | early responsive to dehydration |
| LEA 2 | ACGTATERD1 | 135 | (-) | ACGT | S000415 | early responsive to dehydration |
| LEA 2 | ACGTATERD1 | 135 | (+) | ACGT | S000415 | early responsive to dehydration |
| LEA 2 | ACGTATERD1 | 206 | (-) | ACGT | S000415 | early responsive to dehydration |
| LEA 2 | ACGTATERD1 | 206 | (+) | ACGT | S000415 | early responsive to dehydration |
| LEA 2 | ACGTATERD1 | 213 | (-) | ACGT | S000415 | early responsive to dehydration |
| LEA 2 | ACGTATERD1 | 213 | (+) | ACGT | S000415 | early responsive to dehydration |
| LEA 2 | ACGTATERD1 | 463 | (-) | ACGT | S000415 | early responsive to dehydration |
| LEA 2 | ACGTATERD1 | 463 | (+) | ACGT | S000415 | early responsive to dehydration |
| LEA 2 | ACGTATERD1 | 244 | (-) | ACGT | S000415 | early responsive to dehydration |
| LEA 2 | ACGTATERD1 | 244 | (+) | ACGT | S000415 | early responsive to dehydration |
| LEA 2 | ACGTATERD1 | 331 | (-) | ACGT | S000415 | early responsive to dehydration |
| LEA 2 | ACGTATERD1 | 331 | (+) | ACGT | S000415 | early responsive to dehydration |
| LEA 2 | ACGTATERD1 | 490 | (-) | ACGT | S000415 | early responsive to dehydration |
| LEA 2 | ACGTATERD1 | 490 | (+) | ACGT | S000415 | early responsive to dehydration |
| LEA 2 | ACGTATERD1 | 505 | (-) | ACGT | S000415 | early responsive to dehydration |
| LEA 2 | ACGTATERD1 | 505 | (+) | ACGT | S000415 | early responsive to dehydration |
| LEA 2 | ACGTATERD1 | 588 | (-) | ACGT | S000415 | early responsive to dehydration |
| LEA 2 | ACGTATERD1 | 588 | (+) | ACGT | S000415 | early responsive to dehydration |
| LEA 2 | ACGTATERD1 | 616 | (-) | ACGT | S000415 | early responsive to dehydration |
| LEA 2 | ACGTATERD1 | 616 | (+) | ACGT | S000415 | early responsive to dehydration |
| LEA 2 | ACGTATERD1 | 243 | (-) | ACGT | S000415 | early responsive to dehydration |
| LEA 2 | ACGTATERD1 | 243 | (+) | ACGT | S000415 | early responsive to dehydration |
| LEA 2 | ACGTATERD1 | 91 | (-) | ACGT | S000415 | early responsive to dehydration |
| LEA 2 | ACGTATERD1 | 91 | (+) | ACGT | S000415 | early responsive to dehydration |
| LEA 2 | ACGTATERD1 | 526 | (-) | ACGT | S000415 | early responsive to dehydration |
| LEA 2 | ACGTATERD1 | 526 | (+) | ACGT | S000415 | early responsive to dehydration |
| LEA 2 | ACGTATERD1 | 161 | (-) | ACGT | S000415 | early responsive to dehydration |
| LEA 2 | ACGTATERD1 | 161 | (+) | ACGT | S000415 | early responsive to dehydration |
| LEA 2 | ACGTATERD1 | 24 | (-) | ACGT | S000415 | early responsive to dehydration |
| LEA 2 | ACGTATERD1 | 24 | (+) | ACGT | S000415 | early responsive to dehydration |
| LEA 2 | ACGTATERD1 | 112 | (-) | ACGT | S000415 | early responsive to dehydration |
| LEA 2 | ACGTATERD1 | 112 | (+) | ACGT | S000415 | early responsive to dehydration |
| LEA 2 | ACGTATERD1 | 365 | (-) | ACGT | S000415 | early responsive to dehydration |
| LEA 2 | ACGTATERD1 | 365 | (+) | ACGT | S000415 | early responsive to dehydration |
| LEA 2 | ACGTATERD1 | 396 | (-) | ACGT | S000415 | early responsive to dehydration |
| LEA 2 | ACGTATERD1 | 396 | (+) | ACGT | S000415 | early responsive to dehydration |
| LEA 2 | ACGTATERD1 | 539 | (-) | ACGT | S000415 | early responsive to dehydration |
| LEA 2 | ACGTATERD1 | 539 | (+) | ACGT | S000415 | early responsive to dehydration |
| LEA 2 | ACGTATERD1 | 689 | (-) | ACGT | S000415 | early responsive to dehydration |
| LEA 2 | ACGTATERD1 | 689 | (+) | ACGT | S000415 | early responsive to dehydration |
| LEA 2 | ACGTATERD1 | 231 | (-) | ACGT | S000415 | early responsive to dehydration |
| LEA 2 | ACGTATERD1 | 231 | (+) | ACGT | S000415 | early responsive to dehydration |
| LEA 2 | ACGTATERD1 | 295 | (-) | ACGT | S000415 | early responsive to dehydration |
| LEA 2 | ACGTATERD1 | 295 | (+) | ACGT | S000415 | early responsive to dehydration |
| LEA 2 | ACGTATERD1 | 338 | (-) | ACGT | S000415 | early responsive to dehydration |
| LEA 2 | ACGTATERD1 | 338 | (+) | ACGT | S000415 | early responsive to dehydration |
| LEA 2 | ACGTATERD1 | 207 | (-) | ACGT | S000415 | early responsive to dehydration |
| LEA 2 | ACGTATERD1 | 207 | (+) | ACGT | S000415 | early responsive to dehydration |
| LEA 2 | ACGTATERD1 | 292 | (-) | ACGT | S000415 | early responsive to dehydration |
| LEA 2 | ACGTATERD1 | 292 | (+) | ACGT | S000415 | early responsive to dehydration |
| LEA 2 | ACGTATERD1 | 732 | (-) | ACGT | S000415 | early responsive to dehydration |
| LEA 2 | ACGTATERD1 | 732 | (+) | ACGT | S000415 | early responsive to dehydration |
| LEA 2 | ACGTATERD1 | 639 | (-) | ACGT | S000415 | early responsive to dehydration |
| LEA 2 | ACGTATERD1 | 639 | (+) | ACGT | S000415 | early responsive to dehydration |
| LEA 2 | ACGTATERD1 | 115 | (-) | ACGT | S000415 | early responsive to dehydration |
| LEA 2 | ACGTATERD1 | 115 | (+) | ACGT | S000415 | early responsive to dehydration |
| LEA 2 | ACGTATERD1 | 280 | (-) | ACGT | S000415 | early responsive to dehydration |
| LEA 2 | ACGTATERD1 | 280 | (+) | ACGT | S000415 | early responsive to dehydration |
| LEA 2 | ACGTATERD1 | 337 | (-) | ACGT | S000415 | early responsive to dehydration |
| LEA 2 | ACGTATERD1 | 337 | (+) | ACGT | S000415 | early responsive to dehydration |
| LEA 2 | ACGTATERD1 | 363 | (-) | ACGT | S000415 | early responsive to dehydration |
| LEA 2 | ACGTATERD1 | 363 | (+) | ACGT | S000415 | early responsive to dehydration |
| LEA 2 | ACGTATERD1 | 531 | (-) | ACGT | S000415 | early responsive to dehydration |
| LEA 2 | ACGTATERD1 | 531 | (+) | ACGT | S000415 | early responsive to dehydration |
| LEA 2 | ACGTATERD1 | 231 | (-) | ACGT | S000415 | early responsive to dehydration |
| LEA 2 | ACGTATERD1 | 231 | (+) | ACGT | S000415 | early responsive to dehydration |
| LEA 2 | ACGTATERD1 | 392 | (-) | ACGT | S000415 | early responsive to dehydration |
| LEA 2 | ACGTATERD1 | 392 | (+) | ACGT | S000415 | early responsive to dehydration |
| LEA 2 | ACGTATERD1 | 658 | (-) | ACGT | S000415 | early responsive to dehydration |
| LEA 2 | ACGTATERD1 | 658 | (+) | ACGT | S000415 | early responsive to dehydration |
| LEA 2 | ACGTATERD1 | 9 | (-) | ACGT | S000415 | early responsive to dehydration |
| LEA 2 | ACGTATERD1 | 9 | (+) | ACGT | S000415 | early responsive to dehydration |
| LEA 2 | ACGTATERD1 | 95 | (-) | ACGT | S000415 | early responsive to dehydration |
| LEA 2 | ACGTATERD1 | 95 | (+) | ACGT | S000415 | early responsive to dehydration |
| LEA 2 | ACGTATERD1 | 362 | (-) | ACGT | S000415 | early responsive to dehydration |
| LEA 2 | ACGTATERD1 | 362 | (+) | ACGT | S000415 | early responsive to dehydration |
| LEA 2 | ACGTATERD1 | 568 | (-) | ACGT | S000415 | early responsive to dehydration |
| LEA 2 | ACGTATERD1 | 568 | (+) | ACGT | S000415 | early responsive to dehydration |
| LEA 2 | ACGTATERD1 | 194 | (-) | ACGT | S000415 | early responsive to dehydration |
| LEA 2 | ACGTATERD1 | 194 | (+) | ACGT | S000415 | early responsive to dehydration |
| LEA 2 | ACGTATERD1 | 200 | (-) | ACGT | S000415 | early responsive to dehydration |
| LEA 2 | ACGTATERD1 | 200 | (+) | ACGT | S000415 | early responsive to dehydration |
| LEA 2 | ACGTATERD1 | 89 | (-) | ACGT | S000415 | early responsive to dehydration |
| LEA 2 | ACGTATERD1 | 89 | (+) | ACGT | S000415 | early responsive to dehydration |
| LEA 2 | ACGTATERD1 | 159 | (-) | ACGT | S000415 | early responsive to dehydration |
| LEA 2 | ACGTATERD1 | 159 | (+) | ACGT | S000415 | early responsive to dehydration |
| LEA 2 | ACGTATERD1 | 109 | (-) | ACGT | S000415 | early responsive to dehydration |
| LEA 2 | ACGTATERD1 | 109 | (+) | ACGT | S000415 | early responsive to dehydration |
| LEA 2 | ACGTATERD1 | 589 | (-) | ACGT | S000415 | early responsive to dehydration |
| LEA 2 | ACGTATERD1 | 589 | (+) | ACGT | S000415 | early responsive to dehydration |
| LEA 2 | ACGTATERD1 | 35 | (-) | ACGT | S000415 | early responsive to dehydration |
| LEA 2 | ACGTATERD1 | 35 | (+) | ACGT | S000415 | early responsive to dehydration |
| LEA 2 | ACGTATERD1 | 84 | (-) | ACGT | S000415 | early responsive to dehydration |
| LEA 2 | ACGTATERD1 | 84 | (+) | ACGT | S000415 | early responsive to dehydration |
| LEA 2 | ACGTATERD1 | 277 | (-) | ACGT | S000415 | early responsive to dehydration |
| LEA 2 | ACGTATERD1 | 277 | (+) | ACGT | S000415 | early responsive to dehydration |
| LEA 2 | ACGTATERD1 | 298 | (-) | ACGT | S000415 | early responsive to dehydration |
| LEA 2 | ACGTATERD1 | 298 | (+) | ACGT | S000415 | early responsive to dehydration |
| LEA 2 | ACGTATERD1 | 598 | (-) | ACGT | S000415 | early responsive to dehydration |
| LEA 2 | ACGTATERD1 | 598 | (+) | ACGT | S000415 | early responsive to dehydration |
| LEA 2 | ACGTATERD1 | 231 | (-) | ACGT | S000415 | early responsive to dehydration |
| LEA 2 | ACGTATERD1 | 231 | (+) | ACGT | S000415 | early responsive to dehydration |
| LEA 2 | ACGTATERD1 | 259 | (-) | ACGT | S000415 | early responsive to dehydration |
| LEA 2 | ACGTATERD1 | 259 | (+) | ACGT | S000415 | early responsive to dehydration |
| LEA 2 | ACGTATERD1 | 376 | (-) | ACGT | S000415 | early responsive to dehydration |
| LEA 2 | ACGTATERD1 | 376 | (+) | ACGT | S000415 | early responsive to dehydration |
| LEA 2 | ACGTATERD1 | 363 | (-) | ACGT | S000415 | early responsive to dehydration |
| LEA 2 | ACGTATERD1 | 363 | (+) | ACGT | S000415 | early responsive to dehydration |
| LEA 2 | ACGTATERD1 | 420 | (-) | ACGT | S000415 | early responsive to dehydration |
| LEA 2 | ACGTATERD1 | 420 | (+) | ACGT | S000415 | early responsive to dehydration |
| LEA 2 | ACGTATERD1 | 531 | (-) | ACGT | S000415 | early responsive to dehydration |
| LEA 2 | ACGTATERD1 | 531 | (+) | ACGT | S000415 | early responsive to dehydration |
| LEA 2 | ACGTATERD1 | 476 | (-) | ACGT | S000415 | early responsive to dehydration |
| LEA 2 | ACGTATERD1 | 476 | (+) | ACGT | S000415 | early responsive to dehydration |
| LEA 2 | ACGTATERD1 | 42 | (-) | ACGT | S000415 | early responsive to dehydration |
| LEA 2 | ACGTATERD1 | 42 | (+) | ACGT | S000415 | early responsive to dehydration |
| LEA 2 | ACGTATERD1 | 72 | (-) | ACGT | S000415 | early responsive to dehydration |
| LEA 2 | ACGTATERD1 | 72 | (+) | ACGT | S000415 | early responsive to dehydration |
| LEA 2 | ACGTATERD1 | 237 | (-) | ACGT | S000415 | early responsive to dehydration |
| LEA 2 | ACGTATERD1 | 237 | (+) | ACGT | S000415 | early responsive to dehydration |
| LEA 2 | ACGTATERD1 | 251 | (-) | ACGT | S000415 | early responsive to dehydration |
| LEA 2 | ACGTATERD1 | 251 | (+) | ACGT | S000415 | early responsive to dehydration |
| LEA 2 | ACGTATERD1 | 427 | (-) | ACGT | S000415 | early responsive to dehydration |
| LEA 2 | ACGTATERD1 | 427 | (+) | ACGT | S000415 | early responsive to dehydration |
| LEA 2 | ACGTATERD1 | 103 | (-) | ACGT | S000415 | early responsive to dehydration |
| LEA 2 | ACGTATERD1 | 103 | (+) | ACGT | S000415 | early responsive to dehydration |
| LEA 2 | ACGTATERD1 | 292 | (-) | ACGT | S000415 | early responsive to dehydration |
| LEA 2 | ACGTATERD1 | 292 | (+) | ACGT | S000415 | early responsive to dehydration |
| LEA 2 | ACGTATERD1 | 136 | (-) | ACGT | S000415 | early responsive to dehydration |
| LEA 2 | ACGTATERD1 | 136 | (+) | ACGT | S000415 | early responsive to dehydration |
| LEA 2 | ACGTATERD1 | 198 | (-) | ACGT | S000415 | early responsive to dehydration |
| LEA 2 | ACGTATERD1 | 198 | (+) | ACGT | S000415 | early responsive to dehydration |
| LEA 2 | ACGTATERD1 | 256 | (-) | ACGT | S000415 | early responsive to dehydration |
| LEA 2 | ACGTATERD1 | 256 | (+) | ACGT | S000415 | early responsive to dehydration |
| LEA 2 | ACGTATERD1 | 270 | (-) | ACGT | S000415 | early responsive to dehydration |
| LEA 2 | ACGTATERD1 | 270 | (+) | ACGT | S000415 | early responsive to dehydration |
| LEA 2 | ACGTATERD1 | 155 | (-) | ACGT | S000415 | early responsive to dehydration |
| LEA 2 | ACGTATERD1 | 155 | (+) | ACGT | S000415 | early responsive to dehydration |
| LEA 2 | ACGTATERD1 | 185 | (-) | ACGT | S000415 | early responsive to dehydration |
| LEA 2 | ACGTATERD1 | 185 | (+) | ACGT | S000415 | early responsive to dehydration |
| LEA 2 | ACGTATERD1 | 381 | (-) | ACGT | S000415 | early responsive to dehydration |
| LEA 2 | ACGTATERD1 | 381 | (+) | ACGT | S000415 | early responsive to dehydration |
| LEA 2 | ACGTATERD1 | 473 | (-) | ACGT | S000415 | early responsive to dehydration |
| LEA 2 | ACGTATERD1 | 473 | (+) | ACGT | S000415 | early responsive to dehydration |
| LEA 2 | ACGTATERD1 | 109 | (-) | ACGT | S000415 | early responsive to dehydration |
| LEA 2 | ACGTATERD1 | 109 | (+) | ACGT | S000415 | early responsive to dehydration |
| LEA 2 | ACGTATERD1 | 435 | (-) | ACGT | S000415 | early responsive to dehydration |
| LEA 2 | ACGTATERD1 | 435 | (+) | ACGT | S000415 | early responsive to dehydration |
| LEA 2 | ACGTATERD1 | 317 | (-) | ACGT | S000415 | early responsive to dehydration |
| LEA 2 | ACGTATERD1 | 317 | (+) | ACGT | S000415 | early responsive to dehydration |
| LEA 2 | ACGTATERD1 | 657 | (-) | ACGT | S000415 | early responsive to dehydration |
| LEA 2 | ACGTATERD1 | 657 | (+) | ACGT | S000415 | early responsive to dehydration |
| LEA 2 | ACGTATERD1 | 89 | (-) | ACGT | S000415 | early responsive to dehydration |
| LEA 2 | ACGTATERD1 | 89 | (+) | ACGT | S000415 | early responsive to dehydration |
| LEA 2 | ACGTATERD1 | 350 | (-) | ACGT | S000415 | early responsive to dehydration |
| LEA 2 | ACGTATERD1 | 350 | (+) | ACGT | S000415 | early responsive to dehydration |
| LEA 2 | ACGTATERD1 | 441 | (-) | ACGT | S000415 | early responsive to dehydration |
| LEA 2 | ACGTATERD1 | 441 | (+) | ACGT | S000415 | early responsive to dehydration |
| LEA 2 | ACGTATERD1 | 567 | (-) | ACGT | S000415 | early responsive to dehydration |
| LEA 2 | ACGTATERD1 | 567 | (+) | ACGT | S000415 | early responsive to dehydration |
| LEA 2 | ACGTATERD1 | 298 | (-) | ACGT | S000415 | early responsive to dehydration |
| LEA 2 | ACGTATERD1 | 298 | (+) | ACGT | S000415 | early responsive to dehydration |
| LEA 2 | ACGTATERD1 | 598 | (-) | ACGT | S000415 | early responsive to dehydration |
| LEA 2 | ACGTATERD1 | 598 | (+) | ACGT | S000415 | early responsive to dehydration |
| LEA 2 | ACGTATERD1 | 262 | (-) | ACGT | S000415 | early responsive to dehydration |
| LEA 2 | ACGTATERD1 | 262 | (+) | ACGT | S000415 | early responsive to dehydration |
| LEA 2 | ACGTATERD1 | 369 | (-) | ACGT | S000415 | early responsive to dehydration |
| LEA 2 | ACGTATERD1 | 369 | (+) | ACGT | S000415 | early responsive to dehydration |
| LEA 2 | ACGTATERD1 | 72 | (-) | ACGT | S000415 | early responsive to dehydration |
| LEA 2 | ACGTATERD1 | 72 | (+) | ACGT | S000415 | early responsive to dehydration |
| LEA 2 | ACGTATERD1 | 234 | (-) | ACGT | S000415 | early responsive to dehydration |
| LEA 2 | ACGTATERD1 | 234 | (+) | ACGT | S000415 | early responsive to dehydration |
| LEA 2 | ACGTATERD1 | 248 | (-) | ACGT | S000415 | early responsive to dehydration |
| LEA 2 | ACGTATERD1 | 248 | (+) | ACGT | S000415 | early responsive to dehydration |
| LEA 2 | ACGTATERD1 | 424 | (-) | ACGT | S000415 | early responsive to dehydration |
| LEA 2 | ACGTATERD1 | 424 | (+) | ACGT | S000415 | early responsive to dehydration |
| LEA 2 | ACGTATERD1 | 81 | (-) | ACGT | S000415 | early responsive to dehydration |
| LEA 2 | ACGTATERD1 | 81 | (+) | ACGT | S000415 | early responsive to dehydration |
| LEA 2 | ACGTATERD1 | 115 | (-) | ACGT | S000415 | early responsive to dehydration |
| LEA 2 | ACGTATERD1 | 115 | (+) | ACGT | S000415 | early responsive to dehydration |
| LEA 2 | ACGTATERD1 | 280 | (-) | ACGT | S000415 | early responsive to dehydration |
| LEA 2 | ACGTATERD1 | 280 | (+) | ACGT | S000415 | early responsive to dehydration |
| LEA 2 | ACGTATERD1 | 337 | (-) | ACGT | S000415 | early responsive to dehydration |
| LEA 2 | ACGTATERD1 | 337 | (+) | ACGT | S000415 | early responsive to dehydration |
| LEA 2 | ACGTATERD1 | 363 | (-) | ACGT | S000415 | early responsive to dehydration |
| LEA 2 | ACGTATERD1 | 363 | (+) | ACGT | S000415 | early responsive to dehydration |
| LEA 2 | ACGTATERD1 | 531 | (-) | ACGT | S000415 | early responsive to dehydration |
| LEA 2 | ACGTATERD1 | 531 | (+) | ACGT | S000415 | early responsive to dehydration |
| LEA 2 | ACGTATERD1 | 231 | (-) | ACGT | S000415 | early responsive to dehydration |
| LEA 2 | ACGTATERD1 | 231 | (+) | ACGT | S000415 | early responsive to dehydration |
| LEA 2 | ACGTATERD1 | 392 | (-) | ACGT | S000415 | early responsive to dehydration |
| LEA 2 | ACGTATERD1 | 392 | (+) | ACGT | S000415 | early responsive to dehydration |
| LEA 2 | ACGTATERD1 | 658 | (-) | ACGT | S000415 | early responsive to dehydration |
| LEA 2 | ACGTATERD1 | 658 | (+) | ACGT | S000415 | early responsive to dehydration |
| LEA 2 | ACGTATERD1 | 72 | (-) | ACGT | S000415 | early responsive to dehydration |
| LEA 2 | ACGTATERD1 | 72 | (+) | ACGT | S000415 | early responsive to dehydration |
| LEA 2 | ACGTATERD1 | 237 | (-) | ACGT | S000415 | early responsive to dehydration |
| LEA 2 | ACGTATERD1 | 237 | (+) | ACGT | S000415 | early responsive to dehydration |
| LEA 2 | ACGTATERD1 | 427 | (-) | ACGT | S000415 | early responsive to dehydration |
| LEA 2 | ACGTATERD1 | 427 | (+) | ACGT | S000415 | early responsive to dehydration |
| LEA 2 | ACGTATERD1 | 155 | (-) | ACGT | S000415 | early responsive to dehydration |
| LEA 2 | ACGTATERD1 | 155 | (+) | ACGT | S000415 | early responsive to dehydration |
| LEA 2 | ACGTATERD1 | 185 | (-) | ACGT | S000415 | early responsive to dehydration |
| LEA 2 | ACGTATERD1 | 185 | (+) | ACGT | S000415 | early responsive to dehydration |
| LEA 2 | ACGTATERD1 | 381 | (-) | ACGT | S000415 | early responsive to dehydration |
| LEA 2 | ACGTATERD1 | 381 | (+) | ACGT | S000415 | early responsive to dehydration |
| LEA 2 | ACGTATERD1 | 473 | (-) | ACGT | S000415 | early responsive to dehydration |
| LEA 2 | ACGTATERD1 | 473 | (+) | ACGT | S000415 | early responsive to dehydration |
| LEA 2 | ACGTATERD1 | 454 | (-) | ACGT | S000415 | early responsive to dehydration |
| LEA 2 | ACGTATERD1 | 454 | (+) | ACGT | S000415 | early responsive to dehydration |
| LEA 2 | ACGTATERD1 | 162 | (-) | ACGT | S000415 | early responsive to dehydration |
| LEA 2 | ACGTATERD1 | 162 | (+) | ACGT | S000415 | early responsive to dehydration |
| LEA 2 | ACGTATERD1 | 142 | (-) | ACGT | S000415 | early responsive to dehydration |
| LEA 2 | ACGTATERD1 | 142 | (+) | ACGT | S000415 | early responsive to dehydration |
| LEA 2 | ACGTATERD1 | 213 | (-) | ACGT | S000415 | early responsive to dehydration |
| LEA 2 | ACGTATERD1 | 213 | (+) | ACGT | S000415 | early responsive to dehydration |
| LEA 2 | ACGTATERD1 | 715 | (-) | ACGT | S000415 | early responsive to dehydration |
| LEA 2 | ACGTATERD1 | 715 | (+) | ACGT | S000415 | early responsive to dehydration |
| LEA 2 | ACGTATERD1 | 162 | (-) | ACGT | S000415 | early responsive to dehydration |
| LEA 2 | ACGTATERD1 | 162 | (+) | ACGT | S000415 | early responsive to dehydration |
| LEA 2 | ACGTATERD1 | 34 | (-) | ACGT | S000415 | early responsive to dehydration |
| LEA 2 | ACGTATERD1 | 34 | (+) | ACGT | S000415 | early responsive to dehydration |
| LEA 2 | ACGTATERD1 | 72 | (-) | ACGT | S000415 | early responsive to dehydration |
| LEA 2 | ACGTATERD1 | 72 | (+) | ACGT | S000415 | early responsive to dehydration |
| LEA 2 | ACGTATERD1 | 237 | (-) | ACGT | S000415 | early responsive to dehydration |
| LEA 2 | ACGTATERD1 | 237 | (+) | ACGT | S000415 | early responsive to dehydration |
| LEA 2 | ACGTATERD1 | 427 | (-) | ACGT | S000415 | early responsive to dehydration |
| LEA 2 | ACGTATERD1 | 427 | (+) | ACGT | S000415 | early responsive to dehydration |
| LEA 2 | ACGTATERD1 | 277 | (-) | ACGT | S000415 | early responsive to dehydration |
| LEA 2 | ACGTATERD1 | 277 | (+) | ACGT | S000415 | early responsive to dehydration |
| LEA 2 | ACGTATERD1 | 414 | (-) | ACGT | S000415 | early responsive to dehydration |
| LEA 2 | ACGTATERD1 | 414 | (+) | ACGT | S000415 | early responsive to dehydration |
| LEA 2 | ACGTATERD1 | 464 | (-) | ACGT | S000415 | early responsive to dehydration |
| LEA 2 | ACGTATERD1 | 464 | (+) | ACGT | S000415 | early responsive to dehydration |
| LEA 2 | ACGTATERD1 | 363 | (-) | ACGT | S000415 | early responsive to dehydration |
| LEA 2 | ACGTATERD1 | 363 | (+) | ACGT | S000415 | early responsive to dehydration |
| LEA 2 | ACGTATERD1 | 81 | (-) | ACGT | S000415 | early responsive to dehydration |
| LEA 2 | ACGTATERD1 | 81 | (+) | ACGT | S000415 | early responsive to dehydration |
| LEA 2 | ACGTATERD1 | 97 | (-) | ACGT | S000415 | early responsive to dehydration |
| LEA 2 | ACGTATERD1 | 97 | (+) | ACGT | S000415 | early responsive to dehydration |
| LEA 2 | ACGTATERD1 | 129 | (-) | ACGT | S000415 | early responsive to dehydration |
| LEA 2 | ACGTATERD1 | 129 | (+) | ACGT | S000415 | early responsive to dehydration |
| LEA 2 | ACGTATERD1 | 231 | (-) | ACGT | S000415 | early responsive to dehydration |
| LEA 2 | ACGTATERD1 | 231 | (+) | ACGT | S000415 | early responsive to dehydration |
| LEA 2 | ACGTATERD1 | 376 | (-) | ACGT | S000415 | early responsive to dehydration |
| LEA 2 | ACGTATERD1 | 376 | (+) | ACGT | S000415 | early responsive to dehydration |
| LEA 2 | ACGTATERD1 | 363 | (-) | ACGT | S000415 | early responsive to dehydration |
| LEA 2 | ACGTATERD1 | 363 | (+) | ACGT | S000415 | early responsive to dehydration |
| LEA 2 | ACGTATERD1 | 420 | (-) | ACGT | S000415 | early responsive to dehydration |
| LEA 2 | ACGTATERD1 | 420 | (+) | ACGT | S000415 | early responsive to dehydration |
| LEA 2 | ACGTATERD1 | 531 | (-) | ACGT | S000415 | early responsive to dehydration |
| LEA 2 | ACGTATERD1 | 531 | (+) | ACGT | S000415 | early responsive to dehydration |
| LEA 2 | ACGTATERD1 | 390 | (-) | ACGT | S000415 | early responsive to dehydration |
| LEA 2 | ACGTATERD1 | 390 | (+) | ACGT | S000415 | early responsive to dehydration |
| LEA 2 | ACGTATERD1 | 546 | (-) | ACGT | S000415 | early responsive to dehydration |
| LEA 2 | ACGTATERD1 | 546 | (+) | ACGT | S000415 | early responsive to dehydration |
| LEA 2 | ACGTATERD1 | 433 | (-) | ACGT | S000415 | early responsive to dehydration |
| LEA 2 | ACGTATERD1 | 433 | (+) | ACGT | S000415 | early responsive to dehydration |
| LEA 2 | ACGTATERD1 | 617 | (-) | ACGT | S000415 | early responsive to dehydration |
| LEA 2 | ACGTATERD1 | 617 | (+) | ACGT | S000415 | early responsive to dehydration |
| LEA 2 | ACGTATERD1 | 903 | (-) | ACGT | S000415 | early responsive to dehydration |
| LEA 2 | ACGTATERD1 | 903 | (+) | ACGT | S000415 | early responsive to dehydration |
| LEA 2 | ACGTATERD1 | 913 | (-) | ACGT | S000415 | early responsive to dehydration |
| LEA 2 | ACGTATERD1 | 913 | (+) | ACGT | S000415 | early responsive to dehydration |
| LEA 2 | ACGTATERD1 | 1029 | (-) | ACGT | S000415 | early responsive to dehydration |
| LEA 2 | ACGTATERD1 | 1029 | (+) | ACGT | S000415 | early responsive to dehydration |
| LEA 2 | ACGTATERD1 | 1169 | (-) | ACGT | S000415 | early responsive to dehydration |
| LEA 2 | ACGTATERD1 | 1169 | (+) | ACGT | S000415 | early responsive to dehydration |
| LEA 2 | ACGTATERD1 | 170 | (-) | ACGT | S000415 | early responsive to dehydration |
| LEA 2 | ACGTATERD1 | 170 | (+) | ACGT | S000415 | early responsive to dehydration |
| LEA 2 | ACGTATERD1 | 481 | (-) | ACGT | S000415 | early responsive to dehydration |
| LEA 2 | ACGTATERD1 | 481 | (+) | ACGT | S000415 | early responsive to dehydration |
| LEA 2 | ACGTATERD1 | 598 | (-) | ACGT | S000415 | early responsive to dehydration |
| LEA 2 | ACGTATERD1 | 598 | (+) | ACGT | S000415 | early responsive to dehydration |
| LEA 2 | ACGTATERD1 | 278 | (-) | ACGT | S000415 | early responsive to dehydration |
| LEA 2 | ACGTATERD1 | 278 | (+) | ACGT | S000415 | early responsive to dehydration |
| LEA 2 | ACGTATERD1 | 328 | (-) | ACGT | S000415 | early responsive to dehydration |
| LEA 2 | ACGTATERD1 | 328 | (+) | ACGT | S000415 | early responsive to dehydration |
| LEA 2 | ACGTATERD1 | 213 | (-) | ACGT | S000415 | early responsive to dehydration |
| LEA 2 | ACGTATERD1 | 213 | (+) | ACGT | S000415 | early responsive to dehydration |
| LEA 2 | ACGTATERD1 | 242 | (-) | ACGT | S000415 | early responsive to dehydration |
| LEA 2 | ACGTATERD1 | 242 | (+) | ACGT | S000415 | early responsive to dehydration |
| LEA 2 | ACGTATERD1 | 447 | (-) | ACGT | S000415 | early responsive to dehydration |
| LEA 2 | ACGTATERD1 | 447 | (+) | ACGT | S000415 | early responsive to dehydration |
| LEA 2 | ACGTATERD1 | 498 | (-) | ACGT | S000415 | early responsive to dehydration |
| LEA 2 | ACGTATERD1 | 498 | (+) | ACGT | S000415 | early responsive to dehydration |
| LEA 2 | ACGTATERD1 | 288 | (-) | ACGT | S000415 | early responsive to dehydration |
| LEA 2 | ACGTATERD1 | 288 | (+) | ACGT | S000415 | early responsive to dehydration |
| LEA 2 | ACGTATERD1 | 983 | (-) | ACGT | S000415 | early responsive to dehydration |
| LEA 2 | ACGTATERD1 | 983 | (+) | ACGT | S000415 | early responsive to dehydration |
| LEA 2 | ACGTATERD1 | 283 | (-) | ACGT | S000415 | early responsive to dehydration |
| LEA 2 | ACGTATERD1 | 283 | (+) | ACGT | S000415 | early responsive to dehydration |
| LEA 2 | ACGTATERD1 | 340 | (-) | ACGT | S000415 | early responsive to dehydration |
| LEA 2 | ACGTATERD1 | 340 | (+) | ACGT | S000415 | early responsive to dehydration |
| LEA 2 | ACGTATERD1 | 557 | (-) | ACGT | S000415 | early responsive to dehydration |
| LEA 2 | ACGTATERD1 | 557 | (+) | ACGT | S000415 | early responsive to dehydration |
| LEA 2 | ACGTATERD1 | 35 | (-) | ACGT | S000415 | early responsive to dehydration |
| LEA 2 | ACGTATERD1 | 35 | (+) | ACGT | S000415 | early responsive to dehydration |
| LEA 2 | ACGTATERD1 | 435 | (-) | ACGT | S000415 | early responsive to dehydration |
| LEA 2 | ACGTATERD1 | 435 | (+) | ACGT | S000415 | early responsive to dehydration |
| LEA 2 | ACGTATERD1 | 591 | (-) | ACGT | S000415 | early responsive to dehydration |
| LEA 2 | ACGTATERD1 | 591 | (+) | ACGT | S000415 | early responsive to dehydration |
| LEA 2 | ACGTATERD1 | 350 | (-) | ACGT | S000415 | early responsive to dehydration |
| LEA 2 | ACGTATERD1 | 350 | (+) | ACGT | S000415 | early responsive to dehydration |
| LEA 2 | ACGTATERD1 | 97 | (-) | ACGT | S000415 | early responsive to dehydration |
| LEA 2 | ACGTATERD1 | 97 | (+) | ACGT | S000415 | early responsive to dehydration |
| LEA 2 | ACGTATERD1 | 370 | (-) | ACGT | S000415 | early responsive to dehydration |
| LEA 2 | ACGTATERD1 | 370 | (+) | ACGT | S000415 | early responsive to dehydration |
| LEA 2 | ACGTATERD1 | 913 | (-) | ACGT | S000415 | early responsive to dehydration |
| LEA 2 | ACGTATERD1 | 913 | (+) | ACGT | S000415 | early responsive to dehydration |
| LEA 2 | ACGTATERD1 | 283 | (-) | ACGT | S000415 | early responsive to dehydration |
| LEA 2 | ACGTATERD1 | 283 | (+) | ACGT | S000415 | early responsive to dehydration |
| LEA 2 | ACGTATERD1 | 106 | (-) | ACGT | S000415 | early responsive to dehydration |
| LEA 2 | ACGTATERD1 | 106 | (+) | ACGT | S000415 | early responsive to dehydration |
| LEA 2 | ACGTATERD1 | 169 | (-) | ACGT | S000415 | early responsive to dehydration |
| LEA 2 | ACGTATERD1 | 169 | (+) | ACGT | S000415 | early responsive to dehydration |
| LEA 2 | ACGTATERD1 | 283 | (-) | ACGT | S000415 | early responsive to dehydration |
| LEA 2 | ACGTATERD1 | 283 | (+) | ACGT | S000415 | early responsive to dehydration |
| LEA 2 | ACGTATERD1 | 340 | (-) | ACGT | S000415 | early responsive to dehydration |
| LEA 2 | ACGTATERD1 | 340 | (+) | ACGT | S000415 | early responsive to dehydration |
| LEA 2 | ACGTATERD1 | 557 | (-) | ACGT | S000415 | early responsive to dehydration |
| LEA 2 | ACGTATERD1 | 557 | (+) | ACGT | S000415 | early responsive to dehydration |
| LEA 2 | ACGTATERD1 | 310 | (-) | ACGT | S000415 | early responsive to dehydration |
| LEA 2 | ACGTATERD1 | 310 | (+) | ACGT | S000415 | early responsive to dehydration |
| LEA 2 | ACGTATERD1 | 347 | (-) | ACGT | S000415 | early responsive to dehydration |
| LEA 2 | ACGTATERD1 | 347 | (+) | ACGT | S000415 | early responsive to dehydration |
| LEA 2 | ACGTATERD1 | 427 | (-) | ACGT | S000415 | early responsive to dehydration |
| LEA 2 | ACGTATERD1 | 427 | (+) | ACGT | S000415 | early responsive to dehydration |
| LEA 2 | ACGTATERD1 | 90 | (-) | ACGT | S000415 | early responsive to dehydration |
| LEA 2 | ACGTATERD1 | 90 | (+) | ACGT | S000415 | early responsive to dehydration |
| LEA 2 | ACGTATERD1 | 361 | (-) | ACGT | S000415 | early responsive to dehydration |
| LEA 2 | ACGTATERD1 | 361 | (+) | ACGT | S000415 | early responsive to dehydration |
| LEA 2 | ACGTATERD1 | 411 | (-) | ACGT | S000415 | early responsive to dehydration |
| LEA 2 | ACGTATERD1 | 411 | (+) | ACGT | S000415 | early responsive to dehydration |
| LEA 2 | ACGTATERD1 | 353 | (-) | ACGT | S000415 | early responsive to dehydration |
| LEA 2 | ACGTATERD1 | 353 | (+) | ACGT | S000415 | early responsive to dehydration |
| LEA 2 | ACGTATERD1 | 629 | (-) | ACGT | S000415 | early responsive to dehydration |
| LEA 2 | ACGTATERD1 | 629 | (+) | ACGT | S000415 | early responsive to dehydration |
| LEA 2 | ACGTATERD1 | 91 | (-) | ACGT | S000415 | early responsive to dehydration |
| LEA 2 | ACGTATERD1 | 91 | (+) | ACGT | S000415 | early responsive to dehydration |
| LEA 2 | ACGTATERD1 | 526 | (-) | ACGT | S000415 | early responsive to dehydration |
| LEA 2 | ACGTATERD1 | 526 | (+) | ACGT | S000415 | early responsive to dehydration |
| LEA 2 | ACGTATERD1 | 70 | (-) | ACGT | S000415 | early responsive to dehydration |
| LEA 2 | ACGTATERD1 | 70 | (+) | ACGT | S000415 | early responsive to dehydration |
| LEA 2 | ACGTATERD1 | 106 | (-) | ACGT | S000415 | early responsive to dehydration |
| LEA 2 | ACGTATERD1 | 106 | (+) | ACGT | S000415 | early responsive to dehydration |
| LEA 2 | ACGTATERD1 | 314 | (-) | ACGT | S000415 | early responsive to dehydration |
| LEA 2 | ACGTATERD1 | 314 | (+) | ACGT | S000415 | early responsive to dehydration |
| LEA 2 | ACGTATERD1 | 162 | (-) | ACGT | S000415 | early responsive to dehydration |
| LEA 2 | ACGTATERD1 | 162 | (+) | ACGT | S000415 | early responsive to dehydration |
| LEA 2 | ACGTATERD1 | 617 | (-) | ACGT | S000415 | early responsive to dehydration |
| LEA 2 | ACGTATERD1 | 617 | (+) | ACGT | S000415 | early responsive to dehydration |
| LEA 2 | ACGTATERD1 | 161 | (-) | ACGT | S000415 | early responsive to dehydration |
| LEA 2 | ACGTATERD1 | 161 | (+) | ACGT | S000415 | early responsive to dehydration |
| LEA 2 | ACGTATERD1 | 158 | (-) | ACGT | S000415 | early responsive to dehydration |
| LEA 2 | ACGTATERD1 | 158 | (+) | ACGT | S000415 | early responsive to dehydration |
| LEA 2 | ACGTATERD1 | 531 | (-) | ACGT | S000415 | early responsive to dehydration |
| LEA 2 | ACGTATERD1 | 531 | (+) | ACGT | S000415 | early responsive to dehydration |
| LEA 2 | ACGTATERD1 | 207 | (-) | ACGT | S000415 | early responsive to dehydration |
| LEA 2 | ACGTATERD1 | 207 | (+) | ACGT | S000415 | early responsive to dehydration |
| LEA 2 | ACGTATERD1 | 402 | (-) | ACGT | S000415 | early responsive to dehydration |
| LEA 2 | ACGTATERD1 | 402 | (+) | ACGT | S000415 | early responsive to dehydration |
| LEA 2 | ACGTATERD1 | 494 | (-) | ACGT | S000415 | early responsive to dehydration |
| LEA 2 | ACGTATERD1 | 494 | (+) | ACGT | S000415 | early responsive to dehydration |
| LEA 2 | ACGTATERD1 | 243 | (-) | ACGT | S000415 | early responsive to dehydration |
| LEA 2 | ACGTATERD1 | 243 | (+) | ACGT | S000415 | early responsive to dehydration |
| LEA 2 | ACGTATERD1 | 292 | (-) | ACGT | S000415 | early responsive to dehydration |
| LEA 2 | ACGTATERD1 | 292 | (+) | ACGT | S000415 | early responsive to dehydration |
| LEA 2 | ACGTATERD1 | 265 | (-) | ACGT | S000415 | early responsive to dehydration |
| LEA 2 | ACGTATERD1 | 265 | (+) | ACGT | S000415 | early responsive to dehydration |
| LEA 2 | ACGTATERD1 | 418 | (-) | ACGT | S000415 | early responsive to dehydration |
| LEA 2 | ACGTATERD1 | 418 | (+) | ACGT | S000415 | early responsive to dehydration |
| LEA 2 | ACGTATERD1 | 439 | (-) | ACGT | S000415 | early responsive to dehydration |
| LEA 2 | ACGTATERD1 | 439 | (+) | ACGT | S000415 | early responsive to dehydration |
| LEA 2 | ACGTATERD1 | 464 | (-) | ACGT | S000415 | early responsive to dehydration |
| LEA 2 | ACGTATERD1 | 464 | (+) | ACGT | S000415 | early responsive to dehydration |
| LEA 2 | ACGTATERD1 | 552 | (-) | ACGT | S000415 | early responsive to dehydration |
| LEA 2 | ACGTATERD1 | 552 | (+) | ACGT | S000415 | early responsive to dehydration |
| LEA 2 | ACGTATERD1 | 81 | (-) | ACGT | S000415 | early responsive to dehydration |
| LEA 2 | ACGTATERD1 | 81 | (+) | ACGT | S000415 | early responsive to dehydration |
| LEA 2 | ACGTATERD1 | 850 | (-) | ACGT | S000415 | early responsive to dehydration |
| LEA 2 | ACGTATERD1 | 850 | (+) | ACGT | S000415 | early responsive to dehydration |
| LEA 2 | ACGTATERD1 | 849 | (-) | ACGT | S000415 | early responsive to dehydration |
| LEA 2 | ACGTATERD1 | 849 | (+) | ACGT | S000415 | early responsive to dehydration |
| LEA 2 | ACGTATERD1 | 231 | (-) | ACGT | S000415 | early responsive to dehydration |
| LEA 2 | ACGTATERD1 | 231 | (+) | ACGT | S000415 | early responsive to dehydration |
| LEA 2 | ACGTATERD1 | 295 | (-) | ACGT | S000415 | early responsive to dehydration |
| LEA 2 | ACGTATERD1 | 295 | (+) | ACGT | S000415 | early responsive to dehydration |
| LEA 2 | ACGTATERD1 | 338 | (-) | ACGT | S000415 | early responsive to dehydration |
| LEA 2 | ACGTATERD1 | 338 | (+) | ACGT | S000415 | early responsive to dehydration |
| LEA 2 | ACGTATERD1 | 103 | (-) | ACGT | S000415 | early responsive to dehydration |
| LEA 2 | ACGTATERD1 | 103 | (+) | ACGT | S000415 | early responsive to dehydration |
| LEA 2 | ACGTATERD1 | 234 | (-) | ACGT | S000415 | early responsive to dehydration |
| LEA 2 | ACGTATERD1 | 234 | (+) | ACGT | S000415 | early responsive to dehydration |
| LEA 2 | ACGTATERD1 | 319 | (-) | ACGT | S000415 | early responsive to dehydration |
| LEA 2 | ACGTATERD1 | 319 | (+) | ACGT | S000415 | early responsive to dehydration |
| LEA 2 | ACGTATERD1 | 341 | (-) | ACGT | S000415 | early responsive to dehydration |
| LEA 2 | ACGTATERD1 | 341 | (+) | ACGT | S000415 | early responsive to dehydration |
| LEA 2 | ACGTATERD1 | 372 | (-) | ACGT | S000415 | early responsive to dehydration |
| LEA 2 | ACGTATERD1 | 372 | (+) | ACGT | S000415 | early responsive to dehydration |
| LEA 2 | ACGTATERD1 | 409 | (-) | ACGT | S000415 | early responsive to dehydration |
| LEA 2 | ACGTATERD1 | 409 | (+) | ACGT | S000415 | early responsive to dehydration |
| LEA 2 | ACGTATERD1 | 414 | (-) | ACGT | S000415 | early responsive to dehydration |
| LEA 2 | ACGTATERD1 | 414 | (+) | ACGT | S000415 | early responsive to dehydration |
| LEA 2 | ACGTATERD1 | 430 | (-) | ACGT | S000415 | early responsive to dehydration |
| LEA 2 | ACGTATERD1 | 430 | (+) | ACGT | S000415 | early responsive to dehydration |
| LEA 2 | ACGTATERD1 | 500 | (-) | ACGT | S000415 | early responsive to dehydration |
| LEA 2 | ACGTATERD1 | 500 | (+) | ACGT | S000415 | early responsive to dehydration |
| LEA 2 | ACGTATERD1 | 277 | (-) | ACGT | S000415 | early responsive to dehydration |
| LEA 2 | ACGTATERD1 | 277 | (+) | ACGT | S000415 | early responsive to dehydration |
| LEA 2 | ACGTATERD1 | 183 | (-) | ACGT | S000415 | early responsive to dehydration |
| LEA 2 | ACGTATERD1 | 183 | (+) | ACGT | S000415 | early responsive to dehydration |
| LEA 2 | ACGTATERD1 | 192 | (-) | ACGT | S000415 | early responsive to dehydration |
| LEA 2 | ACGTATERD1 | 192 | (+) | ACGT | S000415 | early responsive to dehydration |
| LEA 2 | ACGTATERD1 | 531 | (-) | ACGT | S000415 | early responsive to dehydration |
| LEA 2 | ACGTATERD1 | 531 | (+) | ACGT | S000415 | early responsive to dehydration |
| LEA 2 | ACGTATERD1 | 283 | (-) | ACGT | S000415 | early responsive to dehydration |
| LEA 2 | ACGTATERD1 | 283 | (+) | ACGT | S000415 | early responsive to dehydration |
| LEA 2 | ACGTATERD1 | 340 | (-) | ACGT | S000415 | early responsive to dehydration |
| LEA 2 | ACGTATERD1 | 340 | (+) | ACGT | S000415 | early responsive to dehydration |
| LEA 2 | ACGTATERD1 | 557 | (-) | ACGT | S000415 | early responsive to dehydration |
| LEA 2 | ACGTATERD1 | 557 | (+) | ACGT | S000415 | early responsive to dehydration |
| LEA 2 | ACGTATERD1 | 562 | (-) | ACGT | S000415 | early responsive to dehydration |
| LEA 2 | ACGTATERD1 | 562 | (+) | ACGT | S000415 | early responsive to dehydration |
| LEA 2 | ACGTATERD1 | 158 | (-) | ACGT | S000415 | early responsive to dehydration |
| LEA 2 | ACGTATERD1 | 158 | (+) | ACGT | S000415 | early responsive to dehydration |
| LEA 2 | ACGTATERD1 | 531 | (-) | ACGT | S000415 | early responsive to dehydration |
| LEA 2 | ACGTATERD1 | 531 | (+) | ACGT | S000415 | early responsive to dehydration |
| LEA 2 | ACGTATERD1 | 340 | (-) | ACGT | S000415 | early responsive to dehydration |
| LEA 2 | ACGTATERD1 | 340 | (+) | ACGT | S000415 | early responsive to dehydration |
| LEA 2 | ACGTATERD1 | 207 | (-) | ACGT | S000415 | early responsive to dehydration |
| LEA 2 | ACGTATERD1 | 207 | (+) | ACGT | S000415 | early responsive to dehydration |
| LEA 2 | ACGTATERD1 | 402 | (-) | ACGT | S000415 | early responsive to dehydration |
| LEA 2 | ACGTATERD1 | 402 | (+) | ACGT | S000415 | early responsive to dehydration |
| LEA 2 | ACGTATERD1 | 494 | (-) | ACGT | S000415 | early responsive to dehydration |
| LEA 2 | ACGTATERD1 | 494 | (+) | ACGT | S000415 | early responsive to dehydration |
| LEA 2 | ACGTATERD1 | 435 | (-) | ACGT | S000415 | early responsive to dehydration |
| LEA 2 | ACGTATERD1 | 435 | (+) | ACGT | S000415 | early responsive to dehydration |
| LEA 2 | ACGTATERD1 | 591 | (-) | ACGT | S000415 | early responsive to dehydration |
| LEA 2 | ACGTATERD1 | 591 | (+) | ACGT | S000415 | early responsive to dehydration |
| LEA 2 | ACGTATERD1 | 81 | (-) | ACGT | S000415 | early responsive to dehydration |
| LEA 2 | ACGTATERD1 | 81 | (+) | ACGT | S000415 | early responsive to dehydration |
| LEA 2 | ACGTATERD1 | 97 | (-) | ACGT | S000415 | early responsive to dehydration |
| LEA 2 | ACGTATERD1 | 97 | (+) | ACGT | S000415 | early responsive to dehydration |
| LEA 2 | ACGTATERD1 | 388 | (-) | ACGT | S000415 | early responsive to dehydration |
| LEA 2 | ACGTATERD1 | 388 | (+) | ACGT | S000415 | early responsive to dehydration |
| LEA 2 | ACGTATERD1 | 1036 | (-) | ACGT | S000415 | early responsive to dehydration |
| LEA 2 | ACGTATERD1 | 1036 | (+) | ACGT | S000415 | early responsive to dehydration |
| LEA 2 | ACGTATERD1 | 298 | (-) | ACGT | S000415 | early responsive to dehydration |
| LEA 2 | ACGTATERD1 | 298 | (+) | ACGT | S000415 | early responsive to dehydration |
| LEA 2 | ACGTATERD1 | 512 | (-) | ACGT | S000415 | early responsive to dehydration |
| LEA 2 | ACGTATERD1 | 512 | (+) | ACGT | S000415 | early responsive to dehydration |
| LEA 2 | ACGTATERD1 | 295 | (-) | ACGT | S000415 | early responsive to dehydration |
| LEA 2 | ACGTATERD1 | 295 | (+) | ACGT | S000415 | early responsive to dehydration |
| LEA 2 | ACGTATERD1 | 317 | (-) | ACGT | S000415 | early responsive to dehydration |
| LEA 2 | ACGTATERD1 | 317 | (+) | ACGT | S000415 | early responsive to dehydration |
| LEA 2 | ACGTATERD1 | 316 | (-) | ACGT | S000415 | early responsive to dehydration |
| LEA 2 | ACGTATERD1 | 316 | (+) | ACGT | S000415 | early responsive to dehydration |
| LEA 2 | ACGTATERD1 | 350 | (-) | ACGT | S000415 | early responsive to dehydration |
| LEA 2 | ACGTATERD1 | 350 | (+) | ACGT | S000415 | early responsive to dehydration |
| LEA 2 | ACGTATERD1 | 44 | (-) | ACGT | S000415 | early responsive to dehydration |
| LEA 2 | ACGTATERD1 | 44 | (+) | ACGT | S000415 | early responsive to dehydration |
| LEA 2 | ACGTATERD1 | 387 | (-) | ACGT | S000415 | early responsive to dehydration |
| LEA 2 | ACGTATERD1 | 387 | (+) | ACGT | S000415 | early responsive to dehydration |
| LEA 2 | ACGTATERD1 | 467 | (-) | ACGT | S000415 | early responsive to dehydration |
| LEA 2 | ACGTATERD1 | 467 | (+) | ACGT | S000415 | early responsive to dehydration |
| LEA 2 | ACGTATERD1 | 475 | (-) | ACGT | S000415 | early responsive to dehydration |
| LEA 2 | ACGTATERD1 | 475 | (+) | ACGT | S000415 | early responsive to dehydration |
| LEA 2 | ACGTATERD1 | 574 | (-) | ACGT | S000415 | early responsive to dehydration |
| LEA 2 | ACGTATERD1 | 574 | (+) | ACGT | S000415 | early responsive to dehydration |
| LEA 2 | ACGTATERD1 | 592 | (-) | ACGT | S000415 | early responsive to dehydration |
| LEA 2 | ACGTATERD1 | 592 | (+) | ACGT | S000415 | early responsive to dehydration |
| LEA 2 | ACGTATERD1 | 656 | (-) | ACGT | S000415 | early responsive to dehydration |
| LEA 2 | ACGTATERD1 | 656 | (+) | ACGT | S000415 | early responsive to dehydration |
| LEA 2 | ACGTATERD1 | 262 | (-) | ACGT | S000415 | early responsive to dehydration |
| LEA 2 | ACGTATERD1 | 262 | (+) | ACGT | S000415 | early responsive to dehydration |
| LEA 2 | ACGTATERD1 | 369 | (-) | ACGT | S000415 | early responsive to dehydration |
| LEA 2 | ACGTATERD1 | 369 | (+) | ACGT | S000415 | early responsive to dehydration |
| LEA 2 | ACGTATERD1 | 170 | (-) | ACGT | S000415 | early responsive to dehydration |
| LEA 2 | ACGTATERD1 | 170 | (+) | ACGT | S000415 | early responsive to dehydration |
| LEA 2 | ACGTATERD1 | 481 | (-) | ACGT | S000415 | early responsive to dehydration |
| LEA 2 | ACGTATERD1 | 481 | (+) | ACGT | S000415 | early responsive to dehydration |
| LEA 2 | ACGTATERD1 | 598 | (-) | ACGT | S000415 | early responsive to dehydration |
| LEA 2 | ACGTATERD1 | 598 | (+) | ACGT | S000415 | early responsive to dehydration |
| LEA 2 | ACGTATERD1 | 371 | (-) | ACGT | S000415 | early responsive to dehydration |
| LEA 2 | ACGTATERD1 | 371 | (+) | ACGT | S000415 | early responsive to dehydration |
| LEA 2 | ACGTATERD1 | 903 | (-) | ACGT | S000415 | early responsive to dehydration |
| LEA 2 | ACGTATERD1 | 903 | (+) | ACGT | S000415 | early responsive to dehydration |
| LEA 2 | ACGTATERD1 | 913 | (-) | ACGT | S000415 | early responsive to dehydration |
| LEA 2 | ACGTATERD1 | 913 | (+) | ACGT | S000415 | early responsive to dehydration |
| LEA 2 | ACGTATERD1 | 1029 | (-) | ACGT | S000415 | early responsive to dehydration |
| LEA 2 | ACGTATERD1 | 1029 | (+) | ACGT | S000415 | early responsive to dehydration |
| LEA 2 | ACGTATERD1 | 1169 | (-) | ACGT | S000415 | early responsive to dehydration |
| LEA 2 | ACGTATERD1 | 1169 | (+) | ACGT | S000415 | early responsive to dehydration |
| LEA 2 | ACGTATERD1 | 552 | (-) | ACGT | S000415 | early responsive to dehydration |
| LEA 2 | ACGTATERD1 | 552 | (+) | ACGT | S000415 | early responsive to dehydration |
| LEA 2 | ACGTATERD1 | 136 | (-) | ACGT | S000415 | early responsive to dehydration |
| LEA 2 | ACGTATERD1 | 136 | (+) | ACGT | S000415 | early responsive to dehydration |
| LEA 2 | ACGTATERD1 | 198 | (-) | ACGT | S000415 | early responsive to dehydration |
| LEA 2 | ACGTATERD1 | 198 | (+) | ACGT | S000415 | early responsive to dehydration |
| LEA 2 | ACGTATERD1 | 256 | (-) | ACGT | S000415 | early responsive to dehydration |
| LEA 2 | ACGTATERD1 | 256 | (+) | ACGT | S000415 | early responsive to dehydration |
| LEA 2 | ACGTATERD1 | 270 | (-) | ACGT | S000415 | early responsive to dehydration |
| LEA 2 | ACGTATERD1 | 270 | (+) | ACGT | S000415 | early responsive to dehydration |
| LEA 2 | ACGTATERD1 | 598 | (-) | ACGT | S000415 | early responsive to dehydration |
| LEA 2 | ACGTATERD1 | 598 | (+) | ACGT | S000415 | early responsive to dehydration |
| LEA 2 | ACGTATERD1 | 589 | (-) | ACGT | S000415 | early responsive to dehydration |
| LEA 2 | ACGTATERD1 | 589 | (+) | ACGT | S000415 | early responsive to dehydration |
| LEA 2 | ACGTATERD1 | 142 | (-) | ACGT | S000415 | early responsive to dehydration |
| LEA 2 | ACGTATERD1 | 142 | (+) | ACGT | S000415 | early responsive to dehydration |
| LEA 2 | ACGTATERD1 | 213 | (-) | ACGT | S000415 | early responsive to dehydration |
| LEA 2 | ACGTATERD1 | 213 | (+) | ACGT | S000415 | early responsive to dehydration |
| LEA 2 | ACGTATERD1 | 240 | (-) | ACGT | S000415 | early responsive to dehydration |
| LEA 2 | ACGTATERD1 | 240 | (+) | ACGT | S000415 | early responsive to dehydration |
| LEA 2 | ACGTATERD1 | 192 | (-) | ACGT | S000415 | early responsive to dehydration |
| LEA 2 | ACGTATERD1 | 192 | (+) | ACGT | S000415 | early responsive to dehydration |
| LEA 2 | ACGTATERD1 | 183 | (-) | ACGT | S000415 | early responsive to dehydration |
| LEA 2 | ACGTATERD1 | 183 | (+) | ACGT | S000415 | early responsive to dehydration |
| LEA 2 | ACGTATERD1 | 350 | (-) | ACGT | S000415 | early responsive to dehydration |
| LEA 2 | ACGTATERD1 | 350 | (+) | ACGT | S000415 | early responsive to dehydration |
| LEA 2 | ACGTATERD1 | 441 | (-) | ACGT | S000415 | early responsive to dehydration |
| LEA 2 | ACGTATERD1 | 441 | (+) | ACGT | S000415 | early responsive to dehydration |
| LEA 2 | ACGTATERD1 | 567 | (-) | ACGT | S000415 | early responsive to dehydration |
| LEA 2 | ACGTATERD1 | 567 | (+) | ACGT | S000415 | early responsive to dehydration |
| LEA 2 | ACGTATERD1 | 188 | (-) | ACGT | S000415 | early responsive to dehydration |
| LEA 2 | ACGTATERD1 | 188 | (+) | ACGT | S000415 | early responsive to dehydration |
| LEA 2 | ACGTATERD1 | 194 | (-) | ACGT | S000415 | early responsive to dehydration |
| LEA 2 | ASF1MOTIFCAMV | 73 | (-) | TGACG | S000024 | Abiotic and biotic stress |
| LEA 2 | ASF1MOTIFCAMV | 137 | (-) | TGACG | S000024 | Abiotic and biotic stress |
| LEA 2 | CBFHV | 446 | (+) | RYCGAC | S000497 | dehydration responsive element |
| LEA 2 | CBFHV | 78 | (-) | RYCGAC | S000497 | dehydration responsive element |
| LEA 2 | LTRECOREATCOR15 | 497 | (+) | CCGAC | S000153 | necessary for coldor drought |
| LEA 2 | LTRECOREATCOR15 | 818 | (-) | CCGAC | S000153 | necessary for coldor drought |
| LEA 2 | LTRECOREATCOR15 | 11 | (+) | CCGAC | S000153 | necessary for coldor drought |
| LEA 2 | LTRECOREATCOR15 | 313 | (-) | CCGAC | S000153 | necessary for coldor drought |
| LEA 2 | LTRECOREATCOR15 | 348 | (-) | CCGAC | S000153 | necessary for coldor drought |
| LEA 2 | LTRECOREATCOR15 | 681 | (-) | CCGAC | S000153 | necessary for coldor drought |
| LEA 2 | MYBAT | 733 | (+) | TAACTG | S000177 | MYB recognition site |
| LEA 2 | MYBAT | 1315 | (-) | TAACTG | S000177 | MYB recognition site |
| LEA 2 | MYBAT | 856 | (-) | TAACTG | S000177 | MYB recognition site |
| LEA 2 | MYBCOREATCYCB1 | 649 | (-) | AACGG | S000502 | Dehydratio/water stress |
| LEA 2 | MYBCOREATCYCB1 | 61 | (+) | AACGG | S000502 | Dehydratio/water stress |
| LEA 2 | MYBCOREATCYCB1 | 112 | (-) | AACGG | S000502 | Dehydratio/water stress |
| LEA 2 | MYBCOREATCYCB1 | 219 | (-) | AACGG | S000502 | Dehydratio/water stress |
| LEA 2 | MYBCOREATCYCB1 | 240 | (-) | AACGG | S000502 | Dehydratio/water stress |
| LEA 2 | MYBCOREATCYCB1 | 366 | (-) | AACGG | S000502 | Dehydratio/water stress |
| LEA 2 | MYBCOREATCYCB1 | 380 | (-) | AACGG | S000502 | Dehydratio/water stress |
| LEA 2 | MYBCOREATCYCB1 | 132 | (-) | AACGG | S000502 | Dehydratio/water stress |
| LEA 2 | MYBCOREATCYCB1 | 896 | (-) | AACGG | S000502 | Dehydratio/water stress |
| LEA 2 | MYBCOREATCYCB1 | 936 | (+) | AACGG | S000502 | Dehydratio/water stress |
| LEA 2 | MYBCOREATCYCB1 | 232 | (-) | AACGG | S000502 | Dehydratio/water stress |
| LEA 2 | MYBCOREATCYCB1 | 570 | (+) | AACGG | S000502 | Dehydratio/water stress |
| LEA 2 | MYBCOREATCYCB1 | 793 | (+) | AACGG | S000502 | Dehydratio/water stress |
| LEA 2 | MYBCOREATCYCB1 | 683 | (+) | AACGG | S000502 | Dehydratio/water stress |
| LEA 2 | MYBCOREATCYCB1 | 69 | (+) | AACGG | S000502 | Dehydratio/water stress |
| LEA 2 | MYBCOREATCYCB1 | 383 | (-) | AACGG | S000502 | Dehydratio/water stress |
| LEA 2 | MYBCOREATCYCB1 | 62 | (+) | AACGG | S000502 | Dehydratio/water stress |
| LEA 2 | MYBCOREATCYCB1 | 502 | (+) | AACGG | S000502 | Dehydratio/water stress |
| LEA 2 | MYBCOREATCYCB1 | 12 | (+) | AACGG | S000502 | Dehydratio/water stress |
| LEA 2 | MYBCOREATCYCB1 | 535 | (+) | AACGG | S000502 | Dehydratio/water stress |
| LEA 2 | MYBCOREATCYCB1 | 61 | (-) | AACGG | S000502 | Dehydratio/water stress |
| LEA 2 | MYBCOREATCYCB1 | 232 | (-) | AACGG | S000502 | Dehydratio/water stress |
| LEA 2 | MYBCOREATCYCB1 | 40 | (+) | AACGG | S000502 | Dehydratio/water stress |
| LEA 2 | MYBCOREATCYCB1 | 140 | (-) | AACGG | S000502 | Dehydratio/water stress |
| LEA 2 | MYBCOREATCYCB1 | 441 | (+) | AACGG | S000502 | Dehydratio/water stress |
| LEA 2 | MYBCOREATCYCB1 | 692 | (+) | AACGG | S000502 | Dehydratio/water stress |
| LEA 2 | MYBCOREATCYCB1 | 732 | (-) | AACGG | S000502 | Dehydratio/water stress |
| LEA 2 | MYBCOREATCYCB1 | 58 | (+) | AACGG | S000502 | Dehydratio/water stress |
| LEA 2 | MYBCOREATCYCB1 | 392 | (+) | AACGG | S000502 | Dehydratio/water stress |
| LEA 2 | MYBCOREATCYCB1 | 176 | (-) | AACGG | S000502 | Dehydratio/water stress |
| LEA 2 | MYBCOREATCYCB1 | 632 | (-) | AACGG | S000502 | Dehydratio/water stress |
| LEA 2 | MYBCOREATCYCB1 | 12 | (+) | AACGG | S000502 | Dehydratio/water stress |
| LEA 2 | MYBCOREATCYCB1 | 535 | (+) | AACGG | S000502 | Dehydratio/water stress |
| LEA 2 | MYBCOREATCYCB1 | 324 | (+) | AACGG | S000502 | Dehydratio/water stress |
| LEA 2 | MYBCOREATCYCB1 | 137 | (-) | AACGG | S000502 | Dehydratio/water stress |
| LEA 2 | MYBCOREATCYCB1 | 229 | (-) | AACGG | S000502 | Dehydratio/water stress |
| LEA 2 | MYBCOREATCYCB1 | 313 | (+) | AACGG | S000502 | Dehydratio/water stress |
| LEA 2 | MYBCOREATCYCB1 | 513 | (-) | AACGG | S000502 | Dehydratio/water stress |
| LEA 2 | MYBCOREATCYCB1 | 392 | (+) | AACGG | S000502 | Dehydratio/water stress |
| LEA 2 | MYBCOREATCYCB1 | 26 | (-) | AACGG | S000502 | Dehydratio/water stress |
| LEA 2 | MYBCOREATCYCB1 | 482 | (-) | AACGG | S000502 | Dehydratio/water stress |
| LEA 2 | MYBCOREATCYCB1 | 13 | (-) | AACGG | S000502 | Dehydratio/water stress |
| LEA 2 | MYBCOREATCYCB1 | 219 | (-) | AACGG | S000502 | Dehydratio/water stress |
| LEA 2 | MYBCOREATCYCB1 | 425 | (+) | AACGG | S000502 | Dehydratio/water stress |
| LEA 2 | MYBCOREATCYCB1 | 504 | (+) | AACGG | S000502 | Dehydratio/water stress |
| LEA 2 | MYBCOREATCYCB1 | 84 | (-) | AACGG | S000502 | Dehydratio/water stress |
| LEA 2 | MYBCOREATCYCB1 | 487 | (+) | AACGG | S000502 | Dehydratio/water stress |
| LEA 2 | MYBCOREATCYCB1 | 1809 | (-) | AACGG | S000502 | Dehydratio/water stress |
| LEA 2 | MYBCOREATCYCB1 | 860 | (-) | AACGG | S000502 | Dehydratio/water stress |
| LEA 2 | MYBCOREATCYCB1 | 139 | (-) | AACGG | S000502 | Dehydratio/water stress |
| LEA 2 | MYBCOREATCYCB1 | 385 | (+) | AACGG | S000502 | Dehydratio/water stress |
| LEA 2 | MYBCOREATCYCB1 | 542 | (-) | AACGG | S000502 | Dehydratio/water stress |
| LEA 2 | MYBCOREATCYCB1 | 292 | (-) | AACGG | S000502 | Dehydratio/water stress |
| LEA 2 | MYBCOREATCYCB1 | 301 | (-) | AACGG | S000502 | Dehydratio/water stress |
| LEA 2 | MYBCOREATCYCB1 | 573 | (+) | AACGG | S000502 | Dehydratio/water stress |
| LEA 2 | MYBCOREATCYCB1 | 232 | (-) | AACGG | S000502 | Dehydratio/water stress |
| LEA 2 | MYBCOREATCYCB1 | 683 | (+) | AACGG | S000502 | Dehydratio/water stress |
| LEA 2 | MYBCOREATCYCB1 | 242 | (+) | AACGG | S000502 | Dehydratio/water stress |
| LEA 2 | MYBCOREATCYCB1 | 464 | (-) | AACGG | S000502 | Dehydratio/water stress |
| LEA 2 | MYBCOREATCYCB1 | 513 | (-) | AACGG | S000502 | Dehydratio/water stress |
| LEA 2 | MYBCOREATCYCB1 | 793 | (+) | AACGG | S000502 | Dehydratio/water stress |
| LEA 2 | MYBCOREATCYCB1 | 72 | (-) | AACGG | S000502 | Dehydratio/water stress |
| LEA 2 | MYBCOREATCYCB1 | 445 | (-) | AACGG | S000502 | Dehydratio/water stress |
| LEA 2 | MYBCOREATCYCB1 | 124 | (-) | AACGG | S000502 | Dehydratio/water stress |
| LEA 2 | MYBCOREATCYCB1 | 215 | (+) | AACGG | S000502 | Dehydratio/water stress |
| LEA 2 | MYBCOREATCYCB1 | 669 | (-) | AACGG | S000502 | Dehydratio/water stress |
| LEA 2 | MYBCOREATCYCB1 | 162 | (-) | AACGG | S000502 | Dehydratio/water stress |
| LEA 2 | MYBCOREATCYCB1 | 221 | (+) | AACGG | S000502 | Dehydratio/water stress |
| LEA 2 | MYBCOREATCYCB1 | 184 | (-) | AACGG | S000502 | Dehydratio/water stress |
| LEA 2 | MYBCOREATCYCB1 | 1161 | (-) | AACGG | S000502 | Dehydratio/water stress |
| LEA 2 | MYBCOREATCYCB1 | 110 | (-) | AACGG | S000502 | Dehydratio/water stress |
| LEA 2 | MYBCOREATCYCB1 | 112 | (-) | AACGG | S000502 | Dehydratio/water stress |
| LEA 2 | MYBCOREATCYCB1 | 219 | (-) | AACGG | S000502 | Dehydratio/water stress |
| LEA 2 | MYBCOREATCYCB1 | 470 | (-) | AACGG | S000502 | Dehydratio/water stress |
| LEA 2 | MYBCOREATCYCB1 | 626 | (-) | AACGG | S000502 | Dehydratio/water stress |
| LEA 2 | MYBCOREATCYCB1 | 139 | (-) | AACGG | S000502 | Dehydratio/water stress |
| LEA 2 | MYBCOREATCYCB1 | 385 | (+) | AACGG | S000502 | Dehydratio/water stress |
| LEA 2 | MYBCOREATCYCB1 | 301 | (-) | AACGG | S000502 | Dehydratio/water stress |
| LEA 2 | MYBCOREATCYCB1 | 292 | (-) | AACGG | S000502 | Dehydratio/water stress |
| LEA 2 | MYBCOREATCYCB1 | 115 | (-) | AACGG | S000502 | Dehydratio/water stress |
| LEA 2 | MYBCOREATCYCB1 | 321 | (+) | AACGG | S000502 | Dehydratio/water stress |
| LEA 2 | MYBCOREATCYCB1 | 400 | (+) | AACGG | S000502 | Dehydratio/water stress |
| LEA 2 | MYBCOREATCYCB1 | 732 | (+) | AACGG | S000502 | Dehydratio/water stress |
| LEA 2 | MYBCOREATCYCB1 | 860 | (-) | AACGG | S000502 | Dehydratio/water stress |
| LEA 2 | MYBCOREATCYCB1 | 282 | (-) | AACGG | S000502 | Dehydratio/water stress |
| LEA 2 | MYBGAHV | 437 | (+) | TAACAAA | S000181 | MYB recognition site |
| LEA 2 | MYBGAHV | 275 | (+) | TAACAAA | S000181 | MYB recognition site |
| LEA 2 | MYBGAHV | 463 | (-) | TAACAAA | S000181 | MYB recognition site |
| LEA 2 | MYBGAHV | 166 | (-) | TAACAAA | S000181 | MYB recognition site |
| LEA 2 | MYBGAHV | 671 | (-) | TAACAAA | S000181 | MYB recognition site |
| LEA 2 | MYBGAHV | 463 | (-) | TAACAAA | S000181 | MYB recognition site |
| LEA 2 | MYBGAHV | 503 | (+) | TAACAAA | S000181 | MYB recognition site |
| LEA 2 | MYBGAHV | 338 | (+) | TAACAAA | S000181 | MYB recognition site |
| LEA 2 | MYBGAHV | 133 | (-) | TAACAAA | S000181 | MYB recognition site |
| LEA 2 | MYBGAHV | 388 | (+) | TAACAAA | S000181 | MYB recognition site |
| LEA 2 | MYBPLANT | 39 | (+) | MACCWAMC | S000167 | MYB recognition site |
| LEA 2 | MYBPLANT | 53 | (+) | MACCWAMC | S000167 | MYB recognition site |
| LEA 2 | MYBPLANT | 57 | (+) | MACCWAMC | S000167 | MYB recognition site |
| LEA 2 | MYBPLANT | 492 | (-) | MACCWAMC | S000167 | MYB recognition site |
| LEA 2 | MYBPLANT | 527 | (-) | MACCWAMC | S000167 | MYB recognition site |
| LEA 2 | MYBPLANT | 62 | (+) | MACCWAMC | S000167 | MYB recognition site |
| LEA 2 | MYBPLANT | 448 | (-) | MACCWAMC | S000167 | MYB recognition site |
| LEA 2 | MYBPLANT | 52 | (+) | MACCWAMC | S000167 | MYB recognition site |
| LEA 2 | MYBPLANT | 290 | (+) | MACCWAMC | S000167 | MYB recognition site |
| LEA 2 | MYBPLANT | 300 | (+) | MACCWAMC | S000167 | MYB recognition site |
| LEA 2 | MYBPLANT | 533 | (-) | MACCWAMC | S000167 | MYB recognition site |
| LEA 2 | MYBPLANT | 585 | (-) | MACCWAMC | S000167 | MYB recognition site |
| LEA 2 | MYBPLANT | 288 | (-) | MACCWAMC | S000167 | MYB recognition site |
| LEA 2 | MYBPLANT | 677 | (-) | MACCWAMC | S000167 | MYB recognition site |
| LEA 2 | MYBPLANT | 76 | (+) | MACCWAMC | S000167 | MYB recognition site |
| LEA 2 | MYBPLANT | 321 | (+) | MACCWAMC | S000167 | MYB recognition site |
| LEA 2 | MYBPLANT | 300 | (+) | MACCWAMC | S000167 | MYB recognition site |
| LEA 2 | MYBPLANT | 190 | (+) | MACCWAMC | S000167 | MYB recognition site |
| LEA 2 | MYBPLANT | 677 | (-) | MACCWAMC | S000167 | MYB recognition site |
| LEA 2 | MYBPLANT | 321 | (+) | MACCWAMC | S000167 | MYB recognition site |
| LEA 2 | MYBPLANT | 351 | (+) | MACCWAMC | S000167 | MYB recognition site |
| LEA 2 | MYBPLANT | 290 | (+) | MACCWAMC | S000167 | MYB recognition site |
| LEA 2 | MYBPLANT | 300 | (+) | MACCWAMC | S000167 | MYB recognition site |
| LEA 2 | MYBPLANT | 533 | (-) | MACCWAMC | S000167 | MYB recognition site |
| LEA 2 | MYBPLANT | 611 | (-) | MACCWAMC | S000167 | MYB recognition site |
| LEA 2 | MYBPLANT | 67 | (+) | MACCWAMC | S000167 | MYB recognition site |
| LEA 2 | MYBPLANT | 585 | (-) | MACCWAMC | S000167 | MYB recognition site |
| LEA 2 | MYBPLANT | 113 | (+) | MACCWAMC | S000167 | MYB recognition site |
| LEA 2 | MYBPLANT | 619 | (+) | MACCWAMC | S000167 | MYB recognition site |
| LEA 2 | MYBPLANT | 113 | (+) | MACCWAMC | S000167 | MYB recognition site |
| LEA 2 | MYBPLANT | 619 | (+) | MACCWAMC | S000167 | MYB recognition site |
| LEA 2 | MYBPLANT | 143 | (+) | MACCWAMC | S000167 | MYB recognition site |
| LEA 2 | MYBPLANT | 145 | (-) | MACCWAMC | S000167 | MYB recognition site |
| LEA 2 | MYBPLANT | 300 | (+) | MACCWAMC | S000167 | MYB recognition site |
| LEA 2 | MYBPLANT | 62 | (+) | MACCWAMC | S000167 | MYB recognition site |
| LEA 2 | MYBPLANT | 448 | (-) | MACCWAMC | S000167 | MYB recognition site |
| LEA 2 | MYBPLANT | 84 | (-) | MACCWAMC | S000167 | MYB recognition site |
| LEA 2 | MYBPLANT | 330 | (+) | MACCWAMC | S000167 | MYB recognition site |
| LEA 2 | MYBPLANT | 190 | (+) | MACCWAMC | S000167 | MYB recognition site |
| LEA 2 | MYBPLANT | 145 | (+) | MACCWAMC | S000167 | MYB recognition site |
| LEA 2 | MYBPLANT | 145 | (+) | MACCWAMC | S000167 | MYB recognition site |
| LEA 2 | MYBPLANT | 527 | (-) | MACCWAMC | S000167 | MYB recognition site |
| LEA 2 | MYBPLANT | 336 | (+) | MACCWAMC | S000167 | MYB recognition site |
| LEA 2 | MYBPLANT | 448 | (-) | MACCWAMC | S000167 | MYB recognition site |
| LEA 2 | MYBPLANT | 211 | (+) | MACCWAMC | S000167 | MYB recognition site |
| LEA 2 | MYBPLANT | 434 | (-) | MACCWAMC | S000167 | MYB recognition site |
| LEA 2 | MYBPLANT | 52 | (+) | MACCWAMC | S000167 | MYB recognition site |
| LEA 2 | MYBPLANT | 143 | (+) | MACCWAMC | S000167 | MYB recognition site |
| LEA 2 | MYBPLANT | 300 | (+) | MACCWAMC | S000167 | MYB recognition site |
| LEA 2 | MYBPLANT | 533 | (-) | MACCWAMC | S000167 | MYB recognition site |
| LEA 2 | MYBPLANT | 336 | (+) | MACCWAMC | S000167 | MYB recognition site |
| LEA 2 | MYBPLANT | 448 | (-) | MACCWAMC | S000167 | MYB recognition site |
| LEA 2 | MYBPLANT | 271 | (+) | MACCWAMC | S000167 | MYB recognition site |
| LEA 2 | MYBPLANT | 330 | (+) | MACCWAMC | S000167 | MYB recognition site |
| LEA 2 | MYBPLANT | 147 | (+) | MACCWAMC | S000167 | MYB recognition site |
| LEA 2 | MYBPLANT | 643 | (-) | MACCWAMC | S000167 | MYB recognition site |
| LEA 2 | MYBPLANT | 713 | (-) | MACCWAMC | S000167 | MYB recognition site |
| LEA 2 | MYBPLANT | 351 | (+) | MACCWAMC | S000167 | MYB recognition site |
| LEA 2 | MYBPLANT | 434 | (-) | MACCWAMC | S000167 | MYB recognition site |
| LEA 2 | MYBPLANT | 434 | (-) | MACCWAMC | S000167 | MYB recognition site |
| LEA 2 | MYBPZM | 267 | (-) | CCWACC | S000179 | myb homolog binding site |
| LEA 2 | MYBPZM | 314 | (+) | CCWACC | S000179 | myb homolog binding site |
| LEA 2 | MYBPZM | 77 | (+) | CCWACC | S000179 | myb homolog binding site |
| LEA 2 | MYBPZM | 380 | (+) | CCWACC | S000179 | myb homolog binding site |
| LEA 2 | MYBPZM | 271 | (-) | CCWACC | S000179 | myb homolog binding site |
| LEA 2 | MYBPZM | 55 | (+) | CCWACC | S000179 | myb homolog binding site |
| LEA 2 | MYBPZM | 59 | (+) | CCWACC | S000179 | myb homolog binding site |
| LEA 2 | MYBPZM | 172 | (+) | CCWACC | S000179 | myb homolog binding site |
| LEA 2 | MYBPZM | 207 | (+) | CCWACC | S000179 | myb homolog binding site |
| LEA 2 | MYBPZM | 339 | (-) | CCWACC | S000179 | myb homolog binding site |
| LEA 2 | MYBPZM | 480 | (-) | CCWACC | S000179 | myb homolog binding site |
| LEA 2 | MYBPZM | 64 | (+) | CCWACC | S000179 | myb homolog binding site |
| LEA 2 | MYBPZM | 448 | (-) | CCWACC | S000179 | myb homolog binding site |
| LEA 2 | MYBPZM | 28 | (+) | CCWACC | S000179 | myb homolog binding site |
| LEA 2 | MYBPZM | 54 | (+) | CCWACC | S000179 | myb homolog binding site |
| LEA 2 | MYBPZM | 515 | (-) | CCWACC | S000179 | myb homolog binding site |
| LEA 2 | MYBPZM | 292 | (+) | CCWACC | S000179 | myb homolog binding site |
| LEA 2 | MYBPZM | 314 | (+) | CCWACC | S000179 | myb homolog binding site |
| LEA 2 | MYBPZM | 585 | (-) | CCWACC | S000179 | myb homolog binding site |
| LEA 2 | MYBPZM | 17 | (+) | CCWACC | S000179 | myb homolog binding site |
| LEA 2 | MYBPZM | 288 | (-) | CCWACC | S000179 | myb homolog binding site |
| LEA 2 | MYBPZM | 299 | (-) | CCWACC | S000179 | myb homolog binding site |
| LEA 2 | MYBPZM | 78 | (+) | CCWACC | S000179 | myb homolog binding site |
| LEA 2 | MYBPZM | 260 | (+) | CCWACC | S000179 | myb homolog binding site |
| LEA 2 | MYBPZM | 261 | (-) | CCWACC | S000179 | myb homolog binding site |
| LEA 2 | MYBPZM | 145 | (+) | CCWACC | S000179 | myb homolog binding site |
| LEA 2 | MYBPZM | 323 | (+) | CCWACC | S000179 | myb homolog binding site |
| LEA 2 | MYBPZM | 547 | (-) | CCWACC | S000179 | myb homolog binding site |
| LEA 2 | MYBPZM | 566 | (-) | CCWACC | S000179 | myb homolog binding site |
| LEA 2 | MYBPZM | 351 | (-) | CCWACC | S000179 | myb homolog binding site |
| LEA 2 | MYBPZM | 498 | (-) | CCWACC | S000179 | myb homolog binding site |
| LEA 2 | MYBPZM | 261 | (-) | CCWACC | S000179 | myb homolog binding site |
| LEA 2 | MYBPZM | 207 | (-) | CCWACC | S000179 | myb homolog binding site |
| LEA 2 | MYBPZM | 384 | (-) | CCWACC | S000179 | myb homolog binding site |
| LEA 2 | MYBPZM | 323 | (+) | CCWACC | S000179 | myb homolog binding site |
| LEA 2 | MYBPZM | 547 | (-) | CCWACC | S000179 | myb homolog binding site |
| LEA 2 | MYBPZM | 81 | (+) | CCWACC | S000179 | myb homolog binding site |
| LEA 2 | MYBPZM | 670 | (-) | CCWACC | S000179 | myb homolog binding site |
| LEA 2 | MYBPZM | 348 | (-) | CCWACC | S000179 | myb homolog binding site |
| LEA 2 | MYBPZM | 292 | (+) | CCWACC | S000179 | myb homolog binding site |
| LEA 2 | MYBPZM | 69 | (+) | CCWACC | S000179 | myb homolog binding site |
| LEA 2 | MYBPZM | 314 | (+) | CCWACC | S000179 | myb homolog binding site |
| LEA 2 | MYBPZM | 585 | (-) | CCWACC | S000179 | myb homolog binding site |
| LEA 2 | MYBPZM | 115 | (+) | CCWACC | S000179 | myb homolog binding site |
| LEA 2 | MYBPZM | 351 | (-) | CCWACC | S000179 | myb homolog binding site |
| LEA 2 | MYBPZM | 22 | (+) | CCWACC | S000179 | myb homolog binding site |
| LEA 2 | MYBPZM | 115 | (+) | CCWACC | S000179 | myb homolog binding site |
| LEA 2 | MYBPZM | 351 | (-) | CCWACC | S000179 | myb homolog binding site |
| LEA 2 | MYBPZM | 145 | (+) | CCWACC | S000179 | myb homolog binding site |
| LEA 2 | MYBPZM | 145 | (-) | CCWACC | S000179 | myb homolog binding site |
| LEA 2 | MYBPZM | 495 | (-) | CCWACC | S000179 | myb homolog binding site |
| LEA 2 | MYBPZM | 566 | (-) | CCWACC | S000179 | myb homolog binding site |
| LEA 2 | MYBPZM | 64 | (+) | CCWACC | S000179 | myb homolog binding site |
| LEA 2 | MYBPZM | 448 | (-) | CCWACC | S000179 | myb homolog binding site |
| LEA 2 | MYBPZM | 314 | (+) | CCWACC | S000179 | myb homolog binding site |
| LEA 2 | MYBPZM | 360 | (+) | CCWACC | S000179 | myb homolog binding site |
| LEA 2 | MYBPZM | 598 | (-) | CCWACC | S000179 | myb homolog binding site |
| LEA 2 | MYBPZM | 686 | (-) | CCWACC | S000179 | myb homolog binding site |
| LEA 2 | MYBPZM | 126 | (+) | CCWACC | S000179 | myb homolog binding site |
| LEA 2 | MYBPZM | 332 | (+) | CCWACC | S000179 | myb homolog binding site |
| LEA 2 | MYBPZM | 413 | (-) | CCWACC | S000179 | myb homolog binding site |
| LEA 2 | MYBPZM | 345 | (+) | CCWACC | S000179 | myb homolog binding site |
| LEA 2 | MYBPZM | 498 | (-) | CCWACC | S000179 | myb homolog binding site |
| LEA 2 | MYBPZM | 575 | (-) | CCWACC | S000179 | myb homolog binding site |
| LEA 2 | MYBPZM | 147 | (+) | CCWACC | S000179 | myb homolog binding site |
| LEA 2 | MYBPZM | 645 | (-) | CCWACC | S000179 | myb homolog binding site |
| LEA 2 | MYBPZM | 480 | (-) | CCWACC | S000179 | myb homolog binding site |
| LEA 2 | MYBPZM | 242 | (-) | CCWACC | S000179 | myb homolog binding site |
| LEA 2 | MYBPZM | 147 | (+) | CCWACC | S000179 | myb homolog binding site |
| LEA 2 | MYBPZM | 15 | (+) | CCWACC | S000179 | myb homolog binding site |
| LEA 2 | MYBPZM | 278 | (+) | CCWACC | S000179 | myb homolog binding site |
| LEA 2 | MYBPZM | 210 | (+) | CCWACC | S000179 | myb homolog binding site |
| LEA 2 | MYBPZM | 265 | (+) | CCWACC | S000179 | myb homolog binding site |
| LEA 2 | MYBPZM | 327 | (+) | CCWACC | S000179 | myb homolog binding site |
| LEA 2 | MYBPZM | 338 | (+) | CCWACC | S000179 | myb homolog binding site |
| LEA 2 | MYBPZM | 448 | (-) | CCWACC | S000179 | myb homolog binding site |
| LEA 2 | MYBPZM | 213 | (+) | CCWACC | S000179 | myb homolog binding site |
| LEA 2 | MYBPZM | 312 | (+) | CCWACC | S000179 | myb homolog binding site |
| LEA 2 | MYBPZM | 172 | (+) | CCWACC | S000179 | myb homolog binding site |
| LEA 2 | MYBPZM | 92 | (-) | CCWACC | S000179 | myb homolog binding site |
| LEA 2 | MYBPZM | 845 | (+) | CCWACC | S000179 | myb homolog binding site |
| LEA 2 | MYBPZM | 1091 | (+) | CCWACC | S000179 | myb homolog binding site |
| LEA 2 | MYBPZM | 275 | (-) | CCWACC | S000179 | myb homolog binding site |
| LEA 2 | MYBPZM | 517 | (-) | CCWACC | S000179 | myb homolog binding site |
| LEA 2 | MYBPZM | 267 | (-) | CCWACC | S000179 | myb homolog binding site |
| LEA 2 | MYBPZM | 54 | (+) | CCWACC | S000179 | myb homolog binding site |
| LEA 2 | MYBPZM | 636 | (-) | CCWACC | S000179 | myb homolog binding site |
| LEA 2 | MYBPZM | 82 | (+) | CCWACC | S000179 | myb homolog binding site |
| LEA 2 | MYBPZM | 145 | (+) | CCWACC | S000179 | myb homolog binding site |
| LEA 2 | MYBPZM | 686 | (-) | CCWACC | S000179 | myb homolog binding site |
| LEA 2 | MYBPZM | 210 | (+) | CCWACC | S000179 | myb homolog binding site |
| LEA 2 | MYBPZM | 327 | (+) | CCWACC | S000179 | myb homolog binding site |
| LEA 2 | MYBPZM | 338 | (+) | CCWACC | S000179 | myb homolog binding site |
| LEA 2 | MYBPZM | 448 | (-) | CCWACC | S000179 | myb homolog binding site |
| LEA 2 | MYBPZM | 312 | (+) | CCWACC | S000179 | myb homolog binding site |
| LEA 2 | MYBPZM | 126 | (+) | CCWACC | S000179 | myb homolog binding site |
| LEA 2 | MYBPZM | 131 | (+) | CCWACC | S000179 | myb homolog binding site |
| LEA 2 | MYBPZM | 332 | (+) | CCWACC | S000179 | myb homolog binding site |
| LEA 2 | MYBPZM | 109 | (+) | CCWACC | S000179 | myb homolog binding site |
| LEA 2 | MYBPZM | 128 | (+) | CCWACC | S000179 | myb homolog binding site |
| LEA 2 | MYBPZM | 81 | (+) | CCWACC | S000179 | myb homolog binding site |
| LEA 2 | MYBPZM | 670 | (-) | CCWACC | S000179 | myb homolog binding site |
| LEA 2 | MYBPZM | 275 | (-) | CCWACC | S000179 | myb homolog binding site |
| LEA 2 | MYBPZM | 517 | (-) | CCWACC | S000179 | myb homolog binding site |
| LEA 2 | MYBPZM | 431 | (-) | CCWACC | S000179 | myb homolog binding site |
| LEA 2 | MYBPZM | 15 | (+) | CCWACC | S000179 | myb homolog binding site |
| LEA 2 | MYBPZM | 384 | (-) | CCWACC | S000179 | myb homolog binding site |
| LEA 2 | MYBPZM | 299 | (-) | CCWACC | S000179 | myb homolog binding site |
| LEA 2 | MYBPZM | 275 | (-) | CCWACC | S000179 | myb homolog binding site |
| LEA 2 | MYBPZM | 517 | (-) | CCWACC | S000179 | myb homolog binding site |
| LEA 2 | MYBPZM | 24 | (-) | CCWACC | S000179 | myb homolog binding site |
| LEA 2 | MYBPZM | 555 | (-) | CCWACC | S000179 | myb homolog binding site |
| LEA 2 | MYBST1 | 169 | (-) | GGATA | S000180 | Myb DNA binding domain |
| LEA 2 | MYBST1 | 226 | (-) | GGATA | S000180 | Myb DNA binding domain |
| LEA 2 | MYBST1 | 333 | (-) | GGATA | S000180 | Myb DNA binding domain |
| LEA 2 | MYBST1 | 737 | (-) | GGATA | S000180 | Myb DNA binding domain |
| LEA 2 | MYBST1 | 129 | (+) | GGATA | S000180 | Myb DNA binding domain |
| LEA 2 | MYBST1 | 192 | (-) | GGATA | S000180 | Myb DNA binding domain |
| LEA 2 | MYBST1 | 43 | (+) | GGATA | S000180 | Myb DNA binding domain |
| LEA 2 | MYBST1 | 520 | (+) | GGATA | S000180 | Myb DNA binding domain |
| LEA 2 | MYBST1 | 299 | (+) | GGATA | S000180 | Myb DNA binding domain |
| LEA 2 | MYBST1 | 185 | (-) | GGATA | S000180 | Myb DNA binding domain |
| LEA 2 | MYBST1 | 731 | (+) | GGATA | S000180 | Myb DNA binding domain |
| LEA 2 | MYBST1 | 438 | (+) | GGATA | S000180 | Myb DNA binding domain |
| LEA 2 | MYBST1 | 656 | (-) | GGATA | S000180 | Myb DNA binding domain |
| LEA 2 | MYBST1 | 17 | (-) | GGATA | S000180 | Myb DNA binding domain |
| LEA 2 | MYBST1 | 268 | (+) | GGATA | S000180 | Myb DNA binding domain |
| LEA 2 | MYBST1 | 621 | (+) | GGATA | S000180 | Myb DNA binding domain |
| LEA 2 | MYBST1 | 87 | (+) | GGATA | S000180 | Myb DNA binding domain |
| LEA 2 | MYBST1 | 127 | (-) | GGATA | S000180 | Myb DNA binding domain |
| LEA 2 | MYBST1 | 350 | (+) | GGATA | S000180 | Myb DNA binding domain |
| LEA 2 | MYBST1 | 129 | (+) | GGATA | S000180 | Myb DNA binding domain |
| LEA 2 | MYBST1 | 192 | (-) | GGATA | S000180 | Myb DNA binding domain |
| LEA 2 | MYBST1 | 89 | (-) | GGATA | S000180 | Myb DNA binding domain |
| LEA 2 | MYBST1 | 185 | (-) | GGATA | S000180 | Myb DNA binding domain |
| LEA 2 | MYBST1 | 731 | (+) | GGATA | S000180 | Myb DNA binding domain |
| LEA 2 | MYBST1 | 11 | (+) | GGATA | S000180 | Myb DNA binding domain |
| LEA 2 | MYBST1 | 183 | (-) | GGATA | S000180 | Myb DNA binding domain |
| LEA 2 | MYBST1 | 641 | (+) | GGATA | S000180 | Myb DNA binding domain |
| LEA 2 | MYBST1 | 345 | (-) | GGATA | S000180 | Myb DNA binding domain |
| LEA 2 | MYBST1 | 250 | (-) | GGATA | S000180 | Myb DNA binding domain |
| LEA 2 | MYBST1 | 670 | (+) | GGATA | S000180 | Myb DNA binding domain |
| LEA 2 | MYBST1 | 693 | (+) | GGATA | S000180 | Myb DNA binding domain |
| LEA 2 | MYBST1 | 84 | (-) | GGATA | S000180 | Myb DNA binding domain |
| LEA 2 | MYBST1 | 335 | (+) | GGATA | S000180 | Myb DNA binding domain |
| LEA 2 | MYBST1 | 538 | (-) | GGATA | S000180 | Myb DNA binding domain |
| LEA 2 | MYBST1 | 114 | (-) | GGATA | S000180 | Myb DNA binding domain |
| LEA 2 | MYBST1 | 135 | (-) | GGATA | S000180 | Myb DNA binding domain |
| LEA 2 | MYBST1 | 347 | (-) | GGATA | S000180 | Myb DNA binding domain |
| LEA 2 | MYBST1 | 733 | (+) | GGATA | S000180 | Myb DNA binding domain |
| LEA 2 | MYBST1 | 39 | (+) | GGATA | S000180 | Myb DNA binding domain |
| LEA 2 | MYBST1 | 213 | (+) | GGATA | S000180 | Myb DNA binding domain |
| LEA 2 | MYBST1 | 324 | (+) | GGATA | S000180 | Myb DNA binding domain |
| LEA 2 | MYBST1 | 43 | (+) | GGATA | S000180 | Myb DNA binding domain |
| LEA 2 | MYBST1 | 468 | (-) | GGATA | S000180 | Myb DNA binding domain |
| LEA 2 | MYBST1 | 552 | (+) | GGATA | S000180 | Myb DNA binding domain |
| LEA 2 | MYBST1 | 126 | (-) | GGATA | S000180 | Myb DNA binding domain |
| LEA 2 | MYBST1 | 193 | (+) | GGATA | S000180 | Myb DNA binding domain |
| LEA 2 | MYBST1 | 586 | (+) | GGATA | S000180 | Myb DNA binding domain |
| LEA 2 | MYBST1 | 250 | (-) | GGATA | S000180 | Myb DNA binding domain |
| LEA 2 | MYBST1 | 342 | (-) | GGATA | S000180 | Myb DNA binding domain |
| LEA 2 | MYBST1 | 670 | (+) | GGATA | S000180 | Myb DNA binding domain |
| LEA 2 | MYBST1 | 53 | (-) | GGATA | S000180 | Myb DNA binding domain |
| LEA 2 | MYBST1 | 419 | (+) | GGATA | S000180 | Myb DNA binding domain |
| LEA 2 | MYBST1 | 477 | (+) | GGATA | S000180 | Myb DNA binding domain |
| LEA 2 | MYBST1 | 653 | (+) | GGATA | S000180 | Myb DNA binding domain |
| LEA 2 | MYBST1 | 702 | (+) | GGATA | S000180 | Myb DNA binding domain |
| LEA 2 | MYBST1 | 122 | (+) | GGATA | S000180 | Myb DNA binding domain |
| LEA 2 | MYBST1 | 281 | (-) | GGATA | S000180 | Myb DNA binding domain |
| LEA 2 | MYBST1 | 642 | (-) | GGATA | S000180 | Myb DNA binding domain |
| LEA 2 | MYBST1 | 114 | (-) | GGATA | S000180 | Myb DNA binding domain |
| LEA 2 | MYBST1 | 135 | (-) | GGATA | S000180 | Myb DNA binding domain |
| LEA 2 | MYBST1 | 347 | (-) | GGATA | S000180 | Myb DNA binding domain |
| LEA 2 | MYBST1 | 535 | (-) | GGATA | S000180 | Myb DNA binding domain |
| LEA 2 | MYBST1 | 43 | (+) | GGATA | S000180 | Myb DNA binding domain |
| LEA 2 | MYBST1 | 214 | (-) | GGATA | S000180 | Myb DNA binding domain |
| LEA 2 | MYBST1 | 226 | (-) | GGATA | S000180 | Myb DNA binding domain |
| LEA 2 | MYBST1 | 187 | (-) | GGATA | S000180 | Myb DNA binding domain |
| LEA 2 | MYBST1 | 678 | (+) | GGATA | S000180 | Myb DNA binding domain |
| LEA 2 | MYBST1 | 84 | (-) | GGATA | S000180 | Myb DNA binding domain |
| LEA 2 | MYBST1 | 335 | (+) | GGATA | S000180 | Myb DNA binding domain |
| LEA 2 | MYBST1 | 39 | (-) | GGATA | S000180 | Myb DNA binding domain |
| LEA 2 | MYBST1 | 167 | (-) | GGATA | S000180 | Myb DNA binding domain |
| LEA 2 | MYBST1 | 419 | (+) | GGATA | S000180 | Myb DNA binding domain |
| LEA 2 | MYBST1 | 477 | (+) | GGATA | S000180 | Myb DNA binding domain |
| LEA 2 | MYBST1 | 94 | (-) | GGATA | S000180 | Myb DNA binding domain |
| LEA 2 | MYBST1 | 116 | (+) | GGATA | S000180 | Myb DNA binding domain |
| LEA 2 | MYBST1 | 119 | (-) | GGATA | S000180 | Myb DNA binding domain |
| LEA 2 | MYBST1 | 129 | (+) | GGATA | S000180 | Myb DNA binding domain |
| LEA 2 | MYBST1 | 412 | (+) | GGATA | S000180 | Myb DNA binding domain |
| LEA 2 | MYBST1 | 39 | (-) | GGATA | S000180 | Myb DNA binding domain |
| LEA 2 | MYBST1 | 165 | (-) | GGATA | S000180 | Myb DNA binding domain |
| LEA 2 | MYBST1 | 342 | (+) | GGATA | S000180 | Myb DNA binding domain |
| LEA 2 | MYBST1 | 402 | (+) | GGATA | S000180 | Myb DNA binding domain |
| LEA 2 | MYBST1 | 656 | (-) | GGATA | S000180 | Myb DNA binding domain |
| LEA 2 | MYBST1 | 552 | (+) | GGATA | S000180 | Myb DNA binding domain |
| LEA 2 | MYBST1 | 123 | (-) | GGATA | S000180 | Myb DNA binding domain |
| LEA 2 | MYBST1 | 190 | (+) | GGATA | S000180 | Myb DNA binding domain |
| LEA 2 | MYBST1 | 209 | (-) | GGATA | S000180 | Myb DNA binding domain |
| LEA 2 | MYBST1 | 656 | (+) | GGATA | S000180 | Myb DNA binding domain |
| LEA 2 | MYBST1 | 89 | (-) | GGATA | S000180 | Myb DNA binding domain |
| LEA 2 | MYBST1 | 272 | (+) | GGATA | S000180 | Myb DNA binding domain |
| LEA 2 | MYBST1 | 344 | (+) | GGATA | S000180 | Myb DNA binding domain |
| LEA 2 | MYBST1 | 492 | (+) | GGATA | S000180 | Myb DNA binding domain |
| LEA 2 | MYBST1 | 592 | (-) | GGATA | S000180 | Myb DNA binding domain |
| LEA 2 | MYBST1 | 281 | (-) | GGATA | S000180 | Myb DNA binding domain |
| LEA 2 | MYBST1 | 853 | (+) | GGATA | S000180 | Myb DNA binding domain |
| LEA 2 | MYBST1 | 480 | (+) | GGATA | S000180 | Myb DNA binding domain |
| LEA 2 | MYBST1 | 281 | (-) | GGATA | S000180 | Myb DNA binding domain |
| LEA 2 | MYBST1 | 226 | (-) | GGATA | S000180 | Myb DNA binding domain |
| LEA 2 | MYBST1 | 333 | (-) | GGATA | S000180 | Myb DNA binding domain |
| LEA 2 | MYBST1 | 727 | (+) | GGATA | S000180 | Myb DNA binding domain |
| LEA 2 | MYBST1 | 228 | (+) | GGATA | S000180 | Myb DNA binding domain |
| LEA 2 | MYBST1 | 24 | (+) | GGATA | S000180 | Myb DNA binding domain |
| LEA 2 | MYBST1 | 222 | (+) | GGATA | S000180 | Myb DNA binding domain |
| LEA 2 | MYBST1 | 330 | (+) | GGATA | S000180 | Myb DNA binding domain |
| LEA 2 | MYBST1 | 727 | (+) | GGATA | S000180 | Myb DNA binding domain |
| LEA 2 | MYBST1 | 129 | (+) | GGATA | S000180 | Myb DNA binding domain |
| LEA 2 | MYBST1 | 192 | (-) | GGATA | S000180 | Myb DNA binding domain |
| LEA 2 | MYBST1 | 87 | (+) | GGATA | S000180 | Myb DNA binding domain |
| LEA 2 | MYBST1 | 103 | (-) | GGATA | S000180 | Myb DNA binding domain |
| LEA 2 | MYBST1 | 72 | (+) | GGATA | S000180 | Myb DNA binding domain |
| LEA 2 | MYBST1 | 180 | (+) | GGATA | S000180 | Myb DNA binding domain |
| LEA 2 | MYBST1 | 228 | (-) | GGATA | S000180 | Myb DNA binding domain |
| LEA 2 | MYBST1 | 309 | (+) | GGATA | S000180 | Myb DNA binding domain |
| LEA 2 | MYBST1 | 364 | (+) | GGATA | S000180 | Myb DNA binding domain |
| LEA 2 | MYBST1 | 1539 | (+) | GGATA | S000180 | Myb DNA binding domain |
| LEA 2 | MYBST1 | 1924 | (+) | GGATA | S000180 | Myb DNA binding domain |
| LEA 2 | MYBST1 | 235 | (-) | GGATA | S000180 | Myb DNA binding domain |
| LEA 2 | MYBST1 | 188 | (-) | GGATA | S000180 | Myb DNA binding domain |
| LEA 2 | MYBST1 | 183 | (-) | GGATA | S000180 | Myb DNA binding domain |
| LEA 2 | MYBST1 | 641 | (+) | GGATA | S000180 | Myb DNA binding domain |
| LEA 2 | MYBST1 | 299 | (+) | GGATA | S000180 | Myb DNA binding domain |
| LEA 2 | MYBST1 | 165 | (-) | GGATA | S000180 | Myb DNA binding domain |
| LEA 2 | MYBST1 | 342 | (+) | GGATA | S000180 | Myb DNA binding domain |
| LEA 2 | MYBST1 | 75 | (+) | GGATA | S000180 | Myb DNA binding domain |
| LEA 2 | MYBST1 | 393 | (+) | GGATA | S000180 | Myb DNA binding domain |
| LEA 2 | MYBST1 | 228 | (-) | GGATA | S000180 | Myb DNA binding domain |
| LEA 2 | MYBST1 | 363 | (+) | GGATA | S000180 | Myb DNA binding domain |
| LEA 2 | MYBST1 | 907 | (-) | GGATA | S000180 | Myb DNA binding domain |
| LEA 2 | MYBST1 | 214 | (-) | GGATA | S000180 | Myb DNA binding domain |
| LEA 2 | MYBST1 | 226 | (-) | GGATA | S000180 | Myb DNA binding domain |
| LEA 2 | MYBST1 | 592 | (-) | GGATA | S000180 | Myb DNA binding domain |
| LEA 2 | MYBST1 | 272 | (+) | GGATA | S000180 | Myb DNA binding domain |
| LEA 2 | MYBST1 | 344 | (+) | GGATA | S000180 | Myb DNA binding domain |
| LEA 2 | MYBST1 | 492 | (+) | GGATA | S000180 | Myb DNA binding domain |
| LEA 2 | MYBST1 | 235 | (-) | GGATA | S000180 | Myb DNA binding domain |
| LEA 2 | MYBST1 | 53 | (-) | GGATA | S000180 | Myb DNA binding domain |
| LEA 2 | MYBST1 | 189 | (-) | GGATA | S000180 | Myb DNA binding domain |
| LEA 2 | MYBST1 | 125 | (-) | GGATA | S000180 | Myb DNA binding domain |
| LEA 2 | MYBST1 | 257 | (+) | GGATA | S000180 | Myb DNA binding domain |
| LEA 2 | MYBST1 | 427 | (-) | GGATA | S000180 | Myb DNA binding domain |
| LEA 2 | MYBST1 | 46 | (-) | GGATA | S000180 | Myb DNA binding domain |
| LEA 2 | MYBST1 | 75 | (+) | GGATA | S000180 | Myb DNA binding domain |
| LEA 2 | MYBST1 | 393 | (+) | GGATA | S000180 | Myb DNA binding domain |
| LEA 2 | MYBST1 | 535 | (-) | GGATA | S000180 | Myb DNA binding domain |
| LEA 2 | MYBST1 | 124 | (-) | GGATA | S000180 | Myb DNA binding domain |
| LEA 2 | MYBST1 | 538 | (-) | GGATA | S000180 | Myb DNA binding domain |
| LEA 2 | MYBST1 | 541 | (+) | GGATA | S000180 | Myb DNA binding domain |
| LEA 2 | MYBST1 | 235 | (-) | GGATA | S000180 | Myb DNA binding domain |
| LEA 2 | MYBST1 | 574 | (-) | GGATA | S000180 | Myb DNA binding domain |
| LEA 2 | MYBST1 | 615 | (-) | GGATA | S000180 | Myb DNA binding domain |
| LEA 2 | MYCATERD1 | 271 | (-) | CATGTG | S000413 | water stress |
| LEA 2 | MYCATERD1 | 76 | (+) | CATGTG | S000413 | water stress |
| LEA 2 | MYCATERD1 | 76 | (+) | CATGTG | S000413 | water stress |
| LEA 2 | MYCATERD1 | 271 | (-) | CATGTG | S000413 | water stress |
| LEA 2 | MYCATERD1 | 311 | (+) | CATGTG | S000413 | water stress |
| LEA 2 | MYCATERD1 | 338 | (-) | CATGTG | S000413 | water stress |
| LEA 2 | MYCATERD1 | 214 | (+) | CATGTG | S000413 | water stress |
| LEA 2 | MYCATERD1 | 221 | (+) | CATGTG | S000413 | water stress |
| LEA 2 | MYCATERD1 | 592 | (+) | CATGTG | S000413 | water stress |
| LEA 2 | MYCATERD1 | 536 | (+) | CATGTG | S000413 | water stress |
| LEA 2 | MYCATERD1 | 592 | (+) | CATGTG | S000413 | water stress |
| LEA 2 | MYCATERD1 | 221 | (+) | CATGTG | S000413 | water stress |
| LEA 2 | MYCATERD1 | 370 | (-) | CATGTG | S000413 | water stress |
| LEA 2 | MYCATERD1 | 283 | (-) | CATGTG | S000413 | water stress |
| LEA 2 | MYCATERD1 | 570 | (+) | CATGTG | S000413 | water stress |
| LEA 2 | MYCATERD1 | 217 | (+) | CATGTG | S000413 | water stress |
| LEA 2 | MYCATERD1 | 258 | (+) | CATGTG | S000413 | water stress |
| LEA 2 | MYCATERD1 | 76 | (+) | CATGTG | S000413 | water stress |
| LEA 2 | MYCATERD1 | 258 | (+) | CATGTG | S000413 | water stress |
| LEA 2 | MYCATERD1 | 271 | (-) | CATGTG | S000413 | water stress |
| LEA 2 | MYCATERD1 | 76 | (+) | CATGTG | S000413 | water stress |
| LEA 2 | MYCATERD1 | 768 | (-) | CATGTG | S000413 | water stress |
| LEA 2 | MYCATERD1 | 273 | (-) | CATGTG | S000413 | water stress |
| LEA 2 | MYCATERD1 | 629 | (+) | CATGTG | S000413 | water stress |
| LEA 2 | MYCATERD1 | 1080 | (+) | CATGTG | S000413 | water stress |
| LEA 2 | MYCATERD1 | 788 | (-) | CATGTG | S000413 | water stress |
| LEA 2 | MYCATERD1 | 283 | (-) | CATGTG | S000413 | water stress |
| LEA 2 | MYCATERD1 | 536 | (+) | CATGTG | S000413 | water stress |
| LEA 2 | MYCATERD1 | 288 | (+) | CATGTG | S000413 | water stress |
| LEA 2 | MYCATERD1 | 594 | (+) | CATGTG | S000413 | water stress |
| LEA 2 | MYCATERD1 | 338 | (-) | CATGTG | S000413 | water stress |
| LEA 2 | MYCATERD1 | 66 | (+) | CATGTG | S000413 | water stress |
| LEA 2 | MYCATERD1 | 96 | (+) | CATGTG | S000413 | water stress |
| LEA 2 | MYCATERD1 | 327 | (+) | CATGTG | S000413 | water stress |
| LEA 2 | MYCATERD1 | 521 | (+) | CATGTG | S000413 | water stress |
| LEA 2 | MYCATERD1 | 621 | (-) | CATGTG | S000413 | water stress |
| LEA 2 | MYCATRD | 271 | (+) | CACATG | S000174 | Dehydration |
| LEA 2 | MYCATRD | 76 | (-) | CACATG | S000174 | Dehydration |
| LEA 2 | MYCATRD | 76 | (-) | CACATG | S000174 | Dehydration |
| LEA 2 | MYCATRD | 271 | (+) | CACATG | S000174 | Dehydration |
| LEA 2 | MYCATRD | 311 | (-) | CACATG | S000174 | Dehydration |
| LEA 2 | MYCATRD | 338 | (+) | CACATG | S000174 | Dehydration |
| LEA 2 | MYCATRD | 214 | (-) | CACATG | S000174 | Dehydration |
| LEA 2 | MYCATRD | 221 | (-) | CACATG | S000174 | Dehydration |
| LEA 2 | MYCATRD | 592 | (-) | CACATG | S000174 | Dehydration |
| LEA 2 | MYCATRD | 536 | (-) | CACATG | S000174 | Dehydration |
| LEA 2 | MYCATRD | 592 | (-) | CACATG | S000174 | Dehydration |
| LEA 2 | MYCATRD | 221 | (-) | CACATG | S000174 | Dehydration |
| LEA 2 | MYCATRD | 370 | (+) | CACATG | S000174 | Dehydration |
| LEA 2 | MYCATRD | 283 | (+) | CACATG | S000174 | Dehydration |
| LEA 2 | MYCATRD | 570 | (-) | CACATG | S000174 | Dehydration |
| LEA 2 | MYCATRD | 217 | (-) | CACATG | S000174 | Dehydration |
| LEA 2 | MYCATRD | 258 | (-) | CACATG | S000174 | Dehydration |
| LEA 2 | MYCATRD | 76 | (-) | CACATG | S000174 | Dehydration |
| LEA 2 | MYCATRD | 258 | (-) | CACATG | S000174 | Dehydration |
| LEA 2 | MYCATRD | 271 | (+) | CACATG | S000174 | Dehydration |
| LEA 2 | MYCATRD | 76 | (-) | CACATG | S000174 | Dehydration |
| LEA 2 | MYCATRD | 768 | (+) | CACATG | S000174 | Dehydration |
| LEA 2 | MYCATRD | 273 | (+) | CACATG | S000174 | Dehydration |
| LEA 2 | MYCATRD | 629 | (-) | CACATG | S000174 | Dehydration |
| LEA 2 | MYCATRD | 1080 | (-) | CACATG | S000174 | Dehydration |
| LEA 2 | MYCATRD | 788 | (+) | CACATG | S000174 | Dehydration |
| LEA 2 | MYCATRD | 283 | (+) | CACATG | S000174 | Dehydration |
| LEA 2 | MYCATRD | 536 | (-) | CACATG | S000174 | Dehydration |
| LEA 2 | MYCATRD | 288 | (-) | CACATG | S000174 | Dehydration |
| LEA 2 | MYCATRD | 594 | (-) | CACATG | S000174 | Dehydration |
| LEA 2 | MYCATRD | 338 | (+) | CACATG | S000174 | Dehydration |
| LEA 2 | MYCATRD | 66 | (-) | CACATG | S000174 | Dehydration |
| LEA 2 | MYCATRD | 96 | (-) | CACATG | S000174 | Dehydration |
| LEA 2 | MYCATRD | 327 | (-) | CACATG | S000174 | Dehydration |
| LEA 2 | MYCATRD | 521 | (-) | CACATG | S000174 | Dehydration |
| LEA 2 | MYCATRD | 621 | (+) | CACATG | S000174 | Dehydration |
| LEA 2 | MYCCONSENSUSAT | 380 | (-) | CANNTG | S000407 | dehydration responsive |
| LEA 2 | MYCCONSENSUSAT | 380 | (+) | CANNTG | S000407 | dehydration responsive |
| LEA 2 | MYCCONSENSUSAT | 577 | (-) | CANNTG | S000407 | dehydration responsive |
| LEA 2 | MYCCONSENSUSAT | 577 | (+) | CANNTG | S000407 | dehydration responsive |
| LEA 2 | MYCCONSENSUSAT | 655 | (-) | CANNTG | S000407 | dehydration responsive |
| LEA 2 | MYCCONSENSUSAT | 655 | (+) | CANNTG | S000407 | dehydration responsive |
| LEA 2 | MYCCONSENSUSAT | 792 | (-) | CANNTG | S000407 | dehydration responsive |
| LEA 2 | MYCCONSENSUSAT | 792 | (+) | CANNTG | S000407 | dehydration responsive |
| LEA 2 | MYCCONSENSUSAT | 413 | (-) | CANNTG | S000407 | dehydration responsive |
| LEA 2 | MYCCONSENSUSAT | 413 | (+) | CANNTG | S000407 | dehydration responsive |
| LEA 2 | MYCCONSENSUSAT | 632 | (-) | CANNTG | S000407 | dehydration responsive |
| LEA 2 | MYCCONSENSUSAT | 632 | (+) | CANNTG | S000407 | dehydration responsive |
| LEA 2 | MYCCONSENSUSAT | 646 | (-) | CANNTG | S000407 | dehydration responsive |
| LEA 2 | MYCCONSENSUSAT | 646 | (+) | CANNTG | S000407 | dehydration responsive |
| LEA 2 | MYCCONSENSUSAT | 754 | (-) | CANNTG | S000407 | dehydration responsive |
| LEA 2 | MYCCONSENSUSAT | 754 | (+) | CANNTG | S000407 | dehydration responsive |
| LEA 2 | MYCCONSENSUSAT | 319 | (-) | CANNTG | S000407 | dehydration responsive |
| LEA 2 | MYCCONSENSUSAT | 319 | (+) | CANNTG | S000407 | dehydration responsive |
| LEA 2 | MYCCONSENSUSAT | 331 | (-) | CANNTG | S000407 | dehydration responsive |
| LEA 2 | MYCCONSENSUSAT | 331 | (+) | CANNTG | S000407 | dehydration responsive |
| LEA 2 | MYCCONSENSUSAT | 799 | (-) | CANNTG | S000407 | dehydration responsive |
| LEA 2 | MYCCONSENSUSAT | 799 | (+) | CANNTG | S000407 | dehydration responsive |
| LEA 2 | MYCCONSENSUSAT | 858 | (-) | CANNTG | S000407 | dehydration responsive |
| LEA 2 | MYCCONSENSUSAT | 858 | (+) | CANNTG | S000407 | dehydration responsive |
| LEA 2 | MYCCONSENSUSAT | 224 | (-) | CANNTG | S000407 | dehydration responsive |
| LEA 2 | MYCCONSENSUSAT | 224 | (+) | CANNTG | S000407 | dehydration responsive |
| LEA 2 | MYCCONSENSUSAT | 635 | (-) | CANNTG | S000407 | dehydration responsive |
| LEA 2 | MYCCONSENSUSAT | 635 | (+) | CANNTG | S000407 | dehydration responsive |
| LEA 2 | MYCCONSENSUSAT | 688 | (-) | CANNTG | S000407 | dehydration responsive |
| LEA 2 | MYCCONSENSUSAT | 688 | (+) | CANNTG | S000407 | dehydration responsive |
| LEA 2 | MYCCONSENSUSAT | 73 | (-) | CANNTG | S000407 | dehydration responsive |
| LEA 2 | MYCCONSENSUSAT | 73 | (+) | CANNTG | S000407 | dehydration responsive |
| LEA 2 | MYCCONSENSUSAT | 271 | (-) | CANNTG | S000407 | dehydration responsive |
| LEA 2 | MYCCONSENSUSAT | 271 | (+) | CANNTG | S000407 | dehydration responsive |
| LEA 2 | MYCCONSENSUSAT | 331 | (-) | CANNTG | S000407 | dehydration responsive |
| LEA 2 | MYCCONSENSUSAT | 331 | (+) | CANNTG | S000407 | dehydration responsive |
| LEA 2 | MYCCONSENSUSAT | 554 | (-) | CANNTG | S000407 | dehydration responsive |
| LEA 2 | MYCCONSENSUSAT | 554 | (+) | CANNTG | S000407 | dehydration responsive |
| LEA 2 | MYCCONSENSUSAT | 110 | (-) | CANNTG | S000407 | dehydration responsive |
| LEA 2 | MYCCONSENSUSAT | 110 | (+) | CANNTG | S000407 | dehydration responsive |
| LEA 2 | MYCCONSENSUSAT | 289 | (-) | CANNTG | S000407 | dehydration responsive |
| LEA 2 | MYCCONSENSUSAT | 289 | (+) | CANNTG | S000407 | dehydration responsive |
| LEA 2 | MYCCONSENSUSAT | 515 | (-) | CANNTG | S000407 | dehydration responsive |
| LEA 2 | MYCCONSENSUSAT | 515 | (+) | CANNTG | S000407 | dehydration responsive |
| LEA 2 | MYCCONSENSUSAT | 429 | (-) | CANNTG | S000407 | dehydration responsive |
| LEA 2 | MYCCONSENSUSAT | 429 | (+) | CANNTG | S000407 | dehydration responsive |
| LEA 2 | MYCCONSENSUSAT | 680 | (-) | CANNTG | S000407 | dehydration responsive |
| LEA 2 | MYCCONSENSUSAT | 680 | (+) | CANNTG | S000407 | dehydration responsive |
| LEA 2 | MYCCONSENSUSAT | 220 | (-) | CANNTG | S000407 | dehydration responsive |
| LEA 2 | MYCCONSENSUSAT | 220 | (+) | CANNTG | S000407 | dehydration responsive |
| LEA 2 | MYCCONSENSUSAT | 134 | (-) | CANNTG | S000407 | dehydration responsive |
| LEA 2 | MYCCONSENSUSAT | 134 | (+) | CANNTG | S000407 | dehydration responsive |
| LEA 2 | MYCCONSENSUSAT | 96 | (-) | CANNTG | S000407 | dehydration responsive |
| LEA 2 | MYCCONSENSUSAT | 96 | (+) | CANNTG | S000407 | dehydration responsive |
| LEA 2 | MYCCONSENSUSAT | 240 | (-) | CANNTG | S000407 | dehydration responsive |
| LEA 2 | MYCCONSENSUSAT | 240 | (+) | CANNTG | S000407 | dehydration responsive |
| LEA 2 | MYCCONSENSUSAT | 523 | (-) | CANNTG | S000407 | dehydration responsive |
| LEA 2 | MYCCONSENSUSAT | 523 | (+) | CANNTG | S000407 | dehydration responsive |
| LEA 2 | MYCCONSENSUSAT | 725 | (-) | CANNTG | S000407 | dehydration responsive |
| LEA 2 | MYCCONSENSUSAT | 725 | (+) | CANNTG | S000407 | dehydration responsive |
| LEA 2 | MYCCONSENSUSAT | 76 | (-) | CANNTG | S000407 | dehydration responsive |
| LEA 2 | MYCCONSENSUSAT | 76 | (+) | CANNTG | S000407 | dehydration responsive |
| LEA 2 | MYCCONSENSUSAT | 107 | (-) | CANNTG | S000407 | dehydration responsive |
| LEA 2 | MYCCONSENSUSAT | 107 | (+) | CANNTG | S000407 | dehydration responsive |
| LEA 2 | MYCCONSENSUSAT | 164 | (-) | CANNTG | S000407 | dehydration responsive |
| LEA 2 | MYCCONSENSUSAT | 164 | (+) | CANNTG | S000407 | dehydration responsive |
| LEA 2 | MYCCONSENSUSAT | 488 | (-) | CANNTG | S000407 | dehydration responsive |
| LEA 2 | MYCCONSENSUSAT | 488 | (+) | CANNTG | S000407 | dehydration responsive |
| LEA 2 | MYCCONSENSUSAT | 76 | (-) | CANNTG | S000407 | dehydration responsive |
| LEA 2 | MYCCONSENSUSAT | 76 | (+) | CANNTG | S000407 | dehydration responsive |
| LEA 2 | MYCCONSENSUSAT | 164 | (-) | CANNTG | S000407 | dehydration responsive |
| LEA 2 | MYCCONSENSUSAT | 164 | (+) | CANNTG | S000407 | dehydration responsive |
| LEA 2 | MYCCONSENSUSAT | 377 | (-) | CANNTG | S000407 | dehydration responsive |
| LEA 2 | MYCCONSENSUSAT | 377 | (+) | CANNTG | S000407 | dehydration responsive |
| LEA 2 | MYCCONSENSUSAT | 488 | (-) | CANNTG | S000407 | dehydration responsive |
| LEA 2 | MYCCONSENSUSAT | 488 | (+) | CANNTG | S000407 | dehydration responsive |
| LEA 2 | MYCCONSENSUSAT | 73 | (-) | CANNTG | S000407 | dehydration responsive |
| LEA 2 | MYCCONSENSUSAT | 73 | (+) | CANNTG | S000407 | dehydration responsive |
| LEA 2 | MYCCONSENSUSAT | 271 | (-) | CANNTG | S000407 | dehydration responsive |
| LEA 2 | MYCCONSENSUSAT | 271 | (+) | CANNTG | S000407 | dehydration responsive |
| LEA 2 | MYCCONSENSUSAT | 331 | (-) | CANNTG | S000407 | dehydration responsive |
| LEA 2 | MYCCONSENSUSAT | 331 | (+) | CANNTG | S000407 | dehydration responsive |
| LEA 2 | MYCCONSENSUSAT | 515 | (-) | CANNTG | S000407 | dehydration responsive |
| LEA 2 | MYCCONSENSUSAT | 515 | (+) | CANNTG | S000407 | dehydration responsive |
| LEA 2 | MYCCONSENSUSAT | 182 | (-) | CANNTG | S000407 | dehydration responsive |
| LEA 2 | MYCCONSENSUSAT | 182 | (+) | CANNTG | S000407 | dehydration responsive |
| LEA 2 | MYCCONSENSUSAT | 367 | (-) | CANNTG | S000407 | dehydration responsive |
| LEA 2 | MYCCONSENSUSAT | 367 | (+) | CANNTG | S000407 | dehydration responsive |
| LEA 2 | MYCCONSENSUSAT | 359 | (-) | CANNTG | S000407 | dehydration responsive |
| LEA 2 | MYCCONSENSUSAT | 359 | (+) | CANNTG | S000407 | dehydration responsive |
| LEA 2 | MYCCONSENSUSAT | 552 | (-) | CANNTG | S000407 | dehydration responsive |
| LEA 2 | MYCCONSENSUSAT | 552 | (+) | CANNTG | S000407 | dehydration responsive |
| LEA 2 | MYCCONSENSUSAT | 211 | (-) | CANNTG | S000407 | dehydration responsive |
| LEA 2 | MYCCONSENSUSAT | 211 | (+) | CANNTG | S000407 | dehydration responsive |
| LEA 2 | MYCCONSENSUSAT | 220 | (-) | CANNTG | S000407 | dehydration responsive |
| LEA 2 | MYCCONSENSUSAT | 220 | (+) | CANNTG | S000407 | dehydration responsive |
| LEA 2 | MYCCONSENSUSAT | 32 | (-) | CANNTG | S000407 | dehydration responsive |
| LEA 2 | MYCCONSENSUSAT | 32 | (+) | CANNTG | S000407 | dehydration responsive |
| LEA 2 | MYCCONSENSUSAT | 528 | (-) | CANNTG | S000407 | dehydration responsive |
| LEA 2 | MYCCONSENSUSAT | 528 | (+) | CANNTG | S000407 | dehydration responsive |
| LEA 2 | MYCCONSENSUSAT | 500 | (-) | CANNTG | S000407 | dehydration responsive |
| LEA 2 | MYCCONSENSUSAT | 500 | (+) | CANNTG | S000407 | dehydration responsive |
| LEA 2 | MYCCONSENSUSAT | 84 | (-) | CANNTG | S000407 | dehydration responsive |
| LEA 2 | MYCCONSENSUSAT | 84 | (+) | CANNTG | S000407 | dehydration responsive |
| LEA 2 | MYCCONSENSUSAT | 96 | (-) | CANNTG | S000407 | dehydration responsive |
| LEA 2 | MYCCONSENSUSAT | 96 | (+) | CANNTG | S000407 | dehydration responsive |
| LEA 2 | MYCCONSENSUSAT | 586 | (-) | CANNTG | S000407 | dehydration responsive |
| LEA 2 | MYCCONSENSUSAT | 586 | (+) | CANNTG | S000407 | dehydration responsive |
| LEA 2 | MYCCONSENSUSAT | 801 | (-) | CANNTG | S000407 | dehydration responsive |
| LEA 2 | MYCCONSENSUSAT | 801 | (+) | CANNTG | S000407 | dehydration responsive |
| LEA 2 | MYCCONSENSUSAT | 37 | (-) | CANNTG | S000407 | dehydration responsive |
| LEA 2 | MYCCONSENSUSAT | 37 | (+) | CANNTG | S000407 | dehydration responsive |
| LEA 2 | MYCCONSENSUSAT | 92 | (-) | CANNTG | S000407 | dehydration responsive |
| LEA 2 | MYCCONSENSUSAT | 92 | (+) | CANNTG | S000407 | dehydration responsive |
| LEA 2 | MYCCONSENSUSAT | 263 | (-) | CANNTG | S000407 | dehydration responsive |
| LEA 2 | MYCCONSENSUSAT | 263 | (+) | CANNTG | S000407 | dehydration responsive |
| LEA 2 | MYCCONSENSUSAT | 308 | (-) | CANNTG | S000407 | dehydration responsive |
| LEA 2 | MYCCONSENSUSAT | 308 | (+) | CANNTG | S000407 | dehydration responsive |
| LEA 2 | MYCCONSENSUSAT | 113 | (-) | CANNTG | S000407 | dehydration responsive |
| LEA 2 | MYCCONSENSUSAT | 113 | (+) | CANNTG | S000407 | dehydration responsive |
| LEA 2 | MYCCONSENSUSAT | 122 | (-) | CANNTG | S000407 | dehydration responsive |
| LEA 2 | MYCCONSENSUSAT | 122 | (+) | CANNTG | S000407 | dehydration responsive |
| LEA 2 | MYCCONSENSUSAT | 311 | (-) | CANNTG | S000407 | dehydration responsive |
| LEA 2 | MYCCONSENSUSAT | 311 | (+) | CANNTG | S000407 | dehydration responsive |
| LEA 2 | MYCCONSENSUSAT | 417 | (-) | CANNTG | S000407 | dehydration responsive |
| LEA 2 | MYCCONSENSUSAT | 417 | (+) | CANNTG | S000407 | dehydration responsive |
| LEA 2 | MYCCONSENSUSAT | 471 | (-) | CANNTG | S000407 | dehydration responsive |
| LEA 2 | MYCCONSENSUSAT | 471 | (+) | CANNTG | S000407 | dehydration responsive |
| LEA 2 | MYCCONSENSUSAT | 338 | (-) | CANNTG | S000407 | dehydration responsive |
| LEA 2 | MYCCONSENSUSAT | 338 | (+) | CANNTG | S000407 | dehydration responsive |
| LEA 2 | MYCCONSENSUSAT | 517 | (-) | CANNTG | S000407 | dehydration responsive |
| LEA 2 | MYCCONSENSUSAT | 517 | (+) | CANNTG | S000407 | dehydration responsive |
| LEA 2 | MYCCONSENSUSAT | 183 | (-) | CANNTG | S000407 | dehydration responsive |
| LEA 2 | MYCCONSENSUSAT | 183 | (+) | CANNTG | S000407 | dehydration responsive |
| LEA 2 | MYCCONSENSUSAT | 222 | (-) | CANNTG | S000407 | dehydration responsive |
| LEA 2 | MYCCONSENSUSAT | 222 | (+) | CANNTG | S000407 | dehydration responsive |
| LEA 2 | MYCCONSENSUSAT | 267 | (-) | CANNTG | S000407 | dehydration responsive |
| LEA 2 | MYCCONSENSUSAT | 267 | (+) | CANNTG | S000407 | dehydration responsive |
| LEA 2 | MYCCONSENSUSAT | 513 | (-) | CANNTG | S000407 | dehydration responsive |
| LEA 2 | MYCCONSENSUSAT | 513 | (+) | CANNTG | S000407 | dehydration responsive |
| LEA 2 | MYCCONSENSUSAT | 708 | (-) | CANNTG | S000407 | dehydration responsive |
| LEA 2 | MYCCONSENSUSAT | 708 | (+) | CANNTG | S000407 | dehydration responsive |
| LEA 2 | MYCCONSENSUSAT | 772 | (-) | CANNTG | S000407 | dehydration responsive |
| LEA 2 | MYCCONSENSUSAT | 772 | (+) | CANNTG | S000407 | dehydration responsive |
| LEA 2 | MYCCONSENSUSAT | 837 | (-) | CANNTG | S000407 | dehydration responsive |
| LEA 2 | MYCCONSENSUSAT | 837 | (+) | CANNTG | S000407 | dehydration responsive |
| LEA 2 | MYCCONSENSUSAT | 873 | (-) | CANNTG | S000407 | dehydration responsive |
| LEA 2 | MYCCONSENSUSAT | 873 | (+) | CANNTG | S000407 | dehydration responsive |
| LEA 2 | MYCCONSENSUSAT | 1060 | (-) | CANNTG | S000407 | dehydration responsive |
| LEA 2 | MYCCONSENSUSAT | 1060 | (+) | CANNTG | S000407 | dehydration responsive |
| LEA 2 | MYCCONSENSUSAT | 20 | (-) | CANNTG | S000407 | dehydration responsive |
| LEA 2 | MYCCONSENSUSAT | 20 | (+) | CANNTG | S000407 | dehydration responsive |
| LEA 2 | MYCCONSENSUSAT | 547 | (-) | CANNTG | S000407 | dehydration responsive |
| LEA 2 | MYCCONSENSUSAT | 547 | (+) | CANNTG | S000407 | dehydration responsive |
| LEA 2 | MYCCONSENSUSAT | 20 | (-) | CANNTG | S000407 | dehydration responsive |
| LEA 2 | MYCCONSENSUSAT | 20 | (+) | CANNTG | S000407 | dehydration responsive |
| LEA 2 | MYCCONSENSUSAT | 124 | (-) | CANNTG | S000407 | dehydration responsive |
| LEA 2 | MYCCONSENSUSAT | 124 | (+) | CANNTG | S000407 | dehydration responsive |
| LEA 2 | MYCCONSENSUSAT | 696 | (-) | CANNTG | S000407 | dehydration responsive |
| LEA 2 | MYCCONSENSUSAT | 696 | (+) | CANNTG | S000407 | dehydration responsive |
| LEA 2 | MYCCONSENSUSAT | 208 | (-) | CANNTG | S000407 | dehydration responsive |
| LEA 2 | MYCCONSENSUSAT | 208 | (+) | CANNTG | S000407 | dehydration responsive |
| LEA 2 | MYCCONSENSUSAT | 214 | (-) | CANNTG | S000407 | dehydration responsive |
| LEA 2 | MYCCONSENSUSAT | 214 | (+) | CANNTG | S000407 | dehydration responsive |
| LEA 2 | MYCCONSENSUSAT | 606 | (-) | CANNTG | S000407 | dehydration responsive |
| LEA 2 | MYCCONSENSUSAT | 606 | (+) | CANNTG | S000407 | dehydration responsive |
| LEA 2 | MYCCONSENSUSAT | 7 | (-) | CANNTG | S000407 | dehydration responsive |
| LEA 2 | MYCCONSENSUSAT | 7 | (+) | CANNTG | S000407 | dehydration responsive |
| LEA 2 | MYCCONSENSUSAT | 171 | (-) | CANNTG | S000407 | dehydration responsive |
| LEA 2 | MYCCONSENSUSAT | 171 | (+) | CANNTG | S000407 | dehydration responsive |
| LEA 2 | MYCCONSENSUSAT | 38 | (-) | CANNTG | S000407 | dehydration responsive |
| LEA 2 | MYCCONSENSUSAT | 38 | (+) | CANNTG | S000407 | dehydration responsive |
| LEA 2 | MYCCONSENSUSAT | 214 | (-) | CANNTG | S000407 | dehydration responsive |
| LEA 2 | MYCCONSENSUSAT | 214 | (+) | CANNTG | S000407 | dehydration responsive |
| LEA 2 | MYCCONSENSUSAT | 719 | (-) | CANNTG | S000407 | dehydration responsive |
| LEA 2 | MYCCONSENSUSAT | 719 | (+) | CANNTG | S000407 | dehydration responsive |
| LEA 2 | MYCCONSENSUSAT | 221 | (-) | CANNTG | S000407 | dehydration responsive |
| LEA 2 | MYCCONSENSUSAT | 221 | (+) | CANNTG | S000407 | dehydration responsive |
| LEA 2 | MYCCONSENSUSAT | 460 | (-) | CANNTG | S000407 | dehydration responsive |
| LEA 2 | MYCCONSENSUSAT | 460 | (+) | CANNTG | S000407 | dehydration responsive |
| LEA 2 | MYCCONSENSUSAT | 545 | (-) | CANNTG | S000407 | dehydration responsive |
| LEA 2 | MYCCONSENSUSAT | 545 | (+) | CANNTG | S000407 | dehydration responsive |
| LEA 2 | MYCCONSENSUSAT | 113 | (-) | CANNTG | S000407 | dehydration responsive |
| LEA 2 | MYCCONSENSUSAT | 113 | (+) | CANNTG | S000407 | dehydration responsive |
| LEA 2 | MYCCONSENSUSAT | 5 | (-) | CANNTG | S000407 | dehydration responsive |
| LEA 2 | MYCCONSENSUSAT | 5 | (+) | CANNTG | S000407 | dehydration responsive |
| LEA 2 | MYCCONSENSUSAT | 26 | (-) | CANNTG | S000407 | dehydration responsive |
| LEA 2 | MYCCONSENSUSAT | 26 | (+) | CANNTG | S000407 | dehydration responsive |
| LEA 2 | MYCCONSENSUSAT | 136 | (-) | CANNTG | S000407 | dehydration responsive |
| LEA 2 | MYCCONSENSUSAT | 136 | (+) | CANNTG | S000407 | dehydration responsive |
| LEA 2 | MYCCONSENSUSAT | 581 | (-) | CANNTG | S000407 | dehydration responsive |
| LEA 2 | MYCCONSENSUSAT | 581 | (+) | CANNTG | S000407 | dehydration responsive |
| LEA 2 | MYCCONSENSUSAT | 807 | (-) | CANNTG | S000407 | dehydration responsive |
| LEA 2 | MYCCONSENSUSAT | 807 | (+) | CANNTG | S000407 | dehydration responsive |
| LEA 2 | MYCCONSENSUSAT | 592 | (-) | CANNTG | S000407 | dehydration responsive |
| LEA 2 | MYCCONSENSUSAT | 592 | (+) | CANNTG | S000407 | dehydration responsive |
| LEA 2 | MYCCONSENSUSAT | 626 | (-) | CANNTG | S000407 | dehydration responsive |
| LEA 2 | MYCCONSENSUSAT | 626 | (+) | CANNTG | S000407 | dehydration responsive |
| LEA 2 | MYCCONSENSUSAT | 703 | (-) | CANNTG | S000407 | dehydration responsive |
| LEA 2 | MYCCONSENSUSAT | 703 | (+) | CANNTG | S000407 | dehydration responsive |
| LEA 2 | MYCCONSENSUSAT | 44 | (-) | CANNTG | S000407 | dehydration responsive |
| LEA 2 | MYCCONSENSUSAT | 44 | (+) | CANNTG | S000407 | dehydration responsive |
| LEA 2 | MYCCONSENSUSAT | 318 | (-) | CANNTG | S000407 | dehydration responsive |
| LEA 2 | MYCCONSENSUSAT | 318 | (+) | CANNTG | S000407 | dehydration responsive |
| LEA 2 | MYCCONSENSUSAT | 521 | (-) | CANNTG | S000407 | dehydration responsive |
| LEA 2 | MYCCONSENSUSAT | 521 | (+) | CANNTG | S000407 | dehydration responsive |
| LEA 2 | MYCCONSENSUSAT | 44 | (-) | CANNTG | S000407 | dehydration responsive |
| LEA 2 | MYCCONSENSUSAT | 44 | (+) | CANNTG | S000407 | dehydration responsive |
| LEA 2 | MYCCONSENSUSAT | 318 | (-) | CANNTG | S000407 | dehydration responsive |
| LEA 2 | MYCCONSENSUSAT | 318 | (+) | CANNTG | S000407 | dehydration responsive |
| LEA 2 | MYCCONSENSUSAT | 521 | (-) | CANNTG | S000407 | dehydration responsive |
| LEA 2 | MYCCONSENSUSAT | 521 | (+) | CANNTG | S000407 | dehydration responsive |
| LEA 2 | MYCCONSENSUSAT | 14 | (-) | CANNTG | S000407 | dehydration responsive |
| LEA 2 | MYCCONSENSUSAT | 14 | (+) | CANNTG | S000407 | dehydration responsive |
| LEA 2 | MYCCONSENSUSAT | 73 | (-) | CANNTG | S000407 | dehydration responsive |
| LEA 2 | MYCCONSENSUSAT | 73 | (+) | CANNTG | S000407 | dehydration responsive |
| LEA 2 | MYCCONSENSUSAT | 298 | (-) | CANNTG | S000407 | dehydration responsive |
| LEA 2 | MYCCONSENSUSAT | 298 | (+) | CANNTG | S000407 | dehydration responsive |
| LEA 2 | MYCCONSENSUSAT | 84 | (-) | CANNTG | S000407 | dehydration responsive |
| LEA 2 | MYCCONSENSUSAT | 84 | (+) | CANNTG | S000407 | dehydration responsive |
| LEA 2 | MYCCONSENSUSAT | 96 | (-) | CANNTG | S000407 | dehydration responsive |
| LEA 2 | MYCCONSENSUSAT | 96 | (+) | CANNTG | S000407 | dehydration responsive |
| LEA 2 | MYCCONSENSUSAT | 586 | (-) | CANNTG | S000407 | dehydration responsive |
| LEA 2 | MYCCONSENSUSAT | 586 | (+) | CANNTG | S000407 | dehydration responsive |
| LEA 2 | MYCCONSENSUSAT | 801 | (-) | CANNTG | S000407 | dehydration responsive |
| LEA 2 | MYCCONSENSUSAT | 801 | (+) | CANNTG | S000407 | dehydration responsive |
| LEA 2 | MYCCONSENSUSAT | 38 | (-) | CANNTG | S000407 | dehydration responsive |
| LEA 2 | MYCCONSENSUSAT | 38 | (+) | CANNTG | S000407 | dehydration responsive |
| LEA 2 | MYCCONSENSUSAT | 536 | (-) | CANNTG | S000407 | dehydration responsive |
| LEA 2 | MYCCONSENSUSAT | 536 | (+) | CANNTG | S000407 | dehydration responsive |
| LEA 2 | MYCCONSENSUSAT | 154 | (-) | CANNTG | S000407 | dehydration responsive |
| LEA 2 | MYCCONSENSUSAT | 154 | (+) | CANNTG | S000407 | dehydration responsive |
| LEA 2 | MYCCONSENSUSAT | 273 | (-) | CANNTG | S000407 | dehydration responsive |
| LEA 2 | MYCCONSENSUSAT | 273 | (+) | CANNTG | S000407 | dehydration responsive |
| LEA 2 | MYCCONSENSUSAT | 310 | (-) | CANNTG | S000407 | dehydration responsive |
| LEA 2 | MYCCONSENSUSAT | 310 | (+) | CANNTG | S000407 | dehydration responsive |
| LEA 2 | MYCCONSENSUSAT | 472 | (-) | CANNTG | S000407 | dehydration responsive |
| LEA 2 | MYCCONSENSUSAT | 472 | (+) | CANNTG | S000407 | dehydration responsive |
| LEA 2 | MYCCONSENSUSAT | 539 | (-) | CANNTG | S000407 | dehydration responsive |
| LEA 2 | MYCCONSENSUSAT | 539 | (+) | CANNTG | S000407 | dehydration responsive |
| LEA 2 | MYCCONSENSUSAT | 20 | (-) | CANNTG | S000407 | dehydration responsive |
| LEA 2 | MYCCONSENSUSAT | 20 | (+) | CANNTG | S000407 | dehydration responsive |
| LEA 2 | MYCCONSENSUSAT | 124 | (-) | CANNTG | S000407 | dehydration responsive |
| LEA 2 | MYCCONSENSUSAT | 124 | (+) | CANNTG | S000407 | dehydration responsive |
| LEA 2 | MYCCONSENSUSAT | 666 | (-) | CANNTG | S000407 | dehydration responsive |
| LEA 2 | MYCCONSENSUSAT | 666 | (+) | CANNTG | S000407 | dehydration responsive |
| LEA 2 | MYCCONSENSUSAT | 732 | (-) | CANNTG | S000407 | dehydration responsive |
| LEA 2 | MYCCONSENSUSAT | 732 | (+) | CANNTG | S000407 | dehydration responsive |
| LEA 2 | MYCCONSENSUSAT | 835 | (-) | CANNTG | S000407 | dehydration responsive |
| LEA 2 | MYCCONSENSUSAT | 835 | (+) | CANNTG | S000407 | dehydration responsive |
| LEA 2 | MYCCONSENSUSAT | 226 | (-) | CANNTG | S000407 | dehydration responsive |
| LEA 2 | MYCCONSENSUSAT | 226 | (+) | CANNTG | S000407 | dehydration responsive |
| LEA 2 | MYCCONSENSUSAT | 240 | (-) | CANNTG | S000407 | dehydration responsive |
| LEA 2 | MYCCONSENSUSAT | 240 | (+) | CANNTG | S000407 | dehydration responsive |
| LEA 2 | MYCCONSENSUSAT | 155 | (-) | CANNTG | S000407 | dehydration responsive |
| LEA 2 | MYCCONSENSUSAT | 155 | (+) | CANNTG | S000407 | dehydration responsive |
| LEA 2 | MYCCONSENSUSAT | 319 | (-) | CANNTG | S000407 | dehydration responsive |
| LEA 2 | MYCCONSENSUSAT | 319 | (+) | CANNTG | S000407 | dehydration responsive |
| LEA 2 | MYCCONSENSUSAT | 390 | (-) | CANNTG | S000407 | dehydration responsive |
| LEA 2 | MYCCONSENSUSAT | 390 | (+) | CANNTG | S000407 | dehydration responsive |
| LEA 2 | MYCCONSENSUSAT | 183 | (-) | CANNTG | S000407 | dehydration responsive |
| LEA 2 | MYCCONSENSUSAT | 183 | (+) | CANNTG | S000407 | dehydration responsive |
| LEA 2 | MYCCONSENSUSAT | 514 | (-) | CANNTG | S000407 | dehydration responsive |
| LEA 2 | MYCCONSENSUSAT | 514 | (+) | CANNTG | S000407 | dehydration responsive |
| LEA 2 | MYCCONSENSUSAT | 38 | (-) | CANNTG | S000407 | dehydration responsive |
| LEA 2 | MYCCONSENSUSAT | 38 | (+) | CANNTG | S000407 | dehydration responsive |
| LEA 2 | MYCCONSENSUSAT | 214 | (-) | CANNTG | S000407 | dehydration responsive |
| LEA 2 | MYCCONSENSUSAT | 214 | (+) | CANNTG | S000407 | dehydration responsive |
| LEA 2 | MYCCONSENSUSAT | 719 | (-) | CANNTG | S000407 | dehydration responsive |
| LEA 2 | MYCCONSENSUSAT | 719 | (+) | CANNTG | S000407 | dehydration responsive |
| LEA 2 | MYCCONSENSUSAT | 38 | (-) | CANNTG | S000407 | dehydration responsive |
| LEA 2 | MYCCONSENSUSAT | 38 | (+) | CANNTG | S000407 | dehydration responsive |
| LEA 2 | MYCCONSENSUSAT | 562 | (-) | CANNTG | S000407 | dehydration responsive |
| LEA 2 | MYCCONSENSUSAT | 562 | (+) | CANNTG | S000407 | dehydration responsive |
| LEA 2 | MYCCONSENSUSAT | 44 | (-) | CANNTG | S000407 | dehydration responsive |
| LEA 2 | MYCCONSENSUSAT | 44 | (+) | CANNTG | S000407 | dehydration responsive |
| LEA 2 | MYCCONSENSUSAT | 130 | (-) | CANNTG | S000407 | dehydration responsive |
| LEA 2 | MYCCONSENSUSAT | 130 | (+) | CANNTG | S000407 | dehydration responsive |
| LEA 2 | MYCCONSENSUSAT | 318 | (-) | CANNTG | S000407 | dehydration responsive |
| LEA 2 | MYCCONSENSUSAT | 318 | (+) | CANNTG | S000407 | dehydration responsive |
| LEA 2 | MYCCONSENSUSAT | 521 | (-) | CANNTG | S000407 | dehydration responsive |
| LEA 2 | MYCCONSENSUSAT | 521 | (+) | CANNTG | S000407 | dehydration responsive |
| LEA 2 | MYCCONSENSUSAT | 436 | (-) | CANNTG | S000407 | dehydration responsive |
| LEA 2 | MYCCONSENSUSAT | 436 | (+) | CANNTG | S000407 | dehydration responsive |
| LEA 2 | MYCCONSENSUSAT | 695 | (-) | CANNTG | S000407 | dehydration responsive |
| LEA 2 | MYCCONSENSUSAT | 695 | (+) | CANNTG | S000407 | dehydration responsive |
| LEA 2 | MYCCONSENSUSAT | 308 | (-) | CANNTG | S000407 | dehydration responsive |
| LEA 2 | MYCCONSENSUSAT | 308 | (+) | CANNTG | S000407 | dehydration responsive |
| LEA 2 | MYCCONSENSUSAT | 113 | (-) | CANNTG | S000407 | dehydration responsive |
| LEA 2 | MYCCONSENSUSAT | 113 | (+) | CANNTG | S000407 | dehydration responsive |
| LEA 2 | MYCCONSENSUSAT | 122 | (-) | CANNTG | S000407 | dehydration responsive |
| LEA 2 | MYCCONSENSUSAT | 122 | (+) | CANNTG | S000407 | dehydration responsive |
| LEA 2 | MYCCONSENSUSAT | 652 | (-) | CANNTG | S000407 | dehydration responsive |
| LEA 2 | MYCCONSENSUSAT | 652 | (+) | CANNTG | S000407 | dehydration responsive |
| LEA 2 | MYCCONSENSUSAT | 154 | (-) | CANNTG | S000407 | dehydration responsive |
| LEA 2 | MYCCONSENSUSAT | 154 | (+) | CANNTG | S000407 | dehydration responsive |
| LEA 2 | MYCCONSENSUSAT | 273 | (-) | CANNTG | S000407 | dehydration responsive |
| LEA 2 | MYCCONSENSUSAT | 273 | (+) | CANNTG | S000407 | dehydration responsive |
| LEA 2 | MYCCONSENSUSAT | 310 | (-) | CANNTG | S000407 | dehydration responsive |
| LEA 2 | MYCCONSENSUSAT | 310 | (+) | CANNTG | S000407 | dehydration responsive |
| LEA 2 | MYCCONSENSUSAT | 472 | (-) | CANNTG | S000407 | dehydration responsive |
| LEA 2 | MYCCONSENSUSAT | 472 | (+) | CANNTG | S000407 | dehydration responsive |
| LEA 2 | MYCCONSENSUSAT | 539 | (-) | CANNTG | S000407 | dehydration responsive |
| LEA 2 | MYCCONSENSUSAT | 539 | (+) | CANNTG | S000407 | dehydration responsive |
| LEA 2 | MYCCONSENSUSAT | 249 | (-) | CANNTG | S000407 | dehydration responsive |
| LEA 2 | MYCCONSENSUSAT | 249 | (+) | CANNTG | S000407 | dehydration responsive |
| LEA 2 | MYCCONSENSUSAT | 409 | (-) | CANNTG | S000407 | dehydration responsive |
| LEA 2 | MYCCONSENSUSAT | 409 | (+) | CANNTG | S000407 | dehydration responsive |
| LEA 2 | MYCCONSENSUSAT | 360 | (-) | CANNTG | S000407 | dehydration responsive |
| LEA 2 | MYCCONSENSUSAT | 360 | (+) | CANNTG | S000407 | dehydration responsive |
| LEA 2 | MYCCONSENSUSAT | 23 | (-) | CANNTG | S000407 | dehydration responsive |
| LEA 2 | MYCCONSENSUSAT | 23 | (+) | CANNTG | S000407 | dehydration responsive |
| LEA 2 | MYCCONSENSUSAT | 500 | (-) | CANNTG | S000407 | dehydration responsive |
| LEA 2 | MYCCONSENSUSAT | 500 | (+) | CANNTG | S000407 | dehydration responsive |
| LEA 2 | MYCCONSENSUSAT | 601 | (-) | CANNTG | S000407 | dehydration responsive |
| LEA 2 | MYCCONSENSUSAT | 601 | (+) | CANNTG | S000407 | dehydration responsive |
| LEA 2 | MYCCONSENSUSAT | 652 | (-) | CANNTG | S000407 | dehydration responsive |
| LEA 2 | MYCCONSENSUSAT | 652 | (+) | CANNTG | S000407 | dehydration responsive |
| LEA 2 | MYCCONSENSUSAT | 171 | (-) | CANNTG | S000407 | dehydration responsive |
| LEA 2 | MYCCONSENSUSAT | 171 | (+) | CANNTG | S000407 | dehydration responsive |
| LEA 2 | MYCCONSENSUSAT | 119 | (-) | CANNTG | S000407 | dehydration responsive |
| LEA 2 | MYCCONSENSUSAT | 119 | (+) | CANNTG | S000407 | dehydration responsive |
| LEA 2 | MYCCONSENSUSAT | 442 | (-) | CANNTG | S000407 | dehydration responsive |
| LEA 2 | MYCCONSENSUSAT | 442 | (+) | CANNTG | S000407 | dehydration responsive |
| LEA 2 | MYCCONSENSUSAT | 592 | (-) | CANNTG | S000407 | dehydration responsive |
| LEA 2 | MYCCONSENSUSAT | 592 | (+) | CANNTG | S000407 | dehydration responsive |
| LEA 2 | MYCCONSENSUSAT | 186 | (-) | CANNTG | S000407 | dehydration responsive |
| LEA 2 | MYCCONSENSUSAT | 186 | (+) | CANNTG | S000407 | dehydration responsive |
| LEA 2 | MYCCONSENSUSAT | 192 | (-) | CANNTG | S000407 | dehydration responsive |
| LEA 2 | MYCCONSENSUSAT | 192 | (+) | CANNTG | S000407 | dehydration responsive |
| LEA 2 | MYCCONSENSUSAT | 620 | (-) | CANNTG | S000407 | dehydration responsive |
| LEA 2 | MYCCONSENSUSAT | 620 | (+) | CANNTG | S000407 | dehydration responsive |
| LEA 2 | MYCCONSENSUSAT | 96 | (-) | CANNTG | S000407 | dehydration responsive |
| LEA 2 | MYCCONSENSUSAT | 96 | (+) | CANNTG | S000407 | dehydration responsive |
| LEA 2 | MYCCONSENSUSAT | 221 | (-) | CANNTG | S000407 | dehydration responsive |
| LEA 2 | MYCCONSENSUSAT | 221 | (+) | CANNTG | S000407 | dehydration responsive |
| LEA 2 | MYCCONSENSUSAT | 460 | (-) | CANNTG | S000407 | dehydration responsive |
| LEA 2 | MYCCONSENSUSAT | 460 | (+) | CANNTG | S000407 | dehydration responsive |
| LEA 2 | MYCCONSENSUSAT | 113 | (-) | CANNTG | S000407 | dehydration responsive |
| LEA 2 | MYCCONSENSUSAT | 113 | (+) | CANNTG | S000407 | dehydration responsive |
| LEA 2 | MYCCONSENSUSAT | 5 | (-) | CANNTG | S000407 | dehydration responsive |
| LEA 2 | MYCCONSENSUSAT | 5 | (+) | CANNTG | S000407 | dehydration responsive |
| LEA 2 | MYCCONSENSUSAT | 26 | (-) | CANNTG | S000407 | dehydration responsive |
| LEA 2 | MYCCONSENSUSAT | 26 | (+) | CANNTG | S000407 | dehydration responsive |
| LEA 2 | MYCCONSENSUSAT | 114 | (-) | CANNTG | S000407 | dehydration responsive |
| LEA 2 | MYCCONSENSUSAT | 114 | (+) | CANNTG | S000407 | dehydration responsive |
| LEA 2 | MYCCONSENSUSAT | 133 | (-) | CANNTG | S000407 | dehydration responsive |
| LEA 2 | MYCCONSENSUSAT | 133 | (+) | CANNTG | S000407 | dehydration responsive |
| LEA 2 | MYCCONSENSUSAT | 351 | (-) | CANNTG | S000407 | dehydration responsive |
| LEA 2 | MYCCONSENSUSAT | 351 | (+) | CANNTG | S000407 | dehydration responsive |
| LEA 2 | MYCCONSENSUSAT | 651 | (-) | CANNTG | S000407 | dehydration responsive |
| LEA 2 | MYCCONSENSUSAT | 651 | (+) | CANNTG | S000407 | dehydration responsive |
| LEA 2 | MYCCONSENSUSAT | 182 | (-) | CANNTG | S000407 | dehydration responsive |
| LEA 2 | MYCCONSENSUSAT | 182 | (+) | CANNTG | S000407 | dehydration responsive |
| LEA 2 | MYCCONSENSUSAT | 536 | (-) | CANNTG | S000407 | dehydration responsive |
| LEA 2 | MYCCONSENSUSAT | 536 | (+) | CANNTG | S000407 | dehydration responsive |
| LEA 2 | MYCCONSENSUSAT | 61 | (-) | CANNTG | S000407 | dehydration responsive |
| LEA 2 | MYCCONSENSUSAT | 61 | (+) | CANNTG | S000407 | dehydration responsive |
| LEA 2 | MYCCONSENSUSAT | 93 | (-) | CANNTG | S000407 | dehydration responsive |
| LEA 2 | MYCCONSENSUSAT | 93 | (+) | CANNTG | S000407 | dehydration responsive |
| LEA 2 | MYCCONSENSUSAT | 112 | (-) | CANNTG | S000407 | dehydration responsive |
| LEA 2 | MYCCONSENSUSAT | 112 | (+) | CANNTG | S000407 | dehydration responsive |
| LEA 2 | MYCCONSENSUSAT | 370 | (-) | CANNTG | S000407 | dehydration responsive |
| LEA 2 | MYCCONSENSUSAT | 370 | (+) | CANNTG | S000407 | dehydration responsive |
| LEA 2 | MYCCONSENSUSAT | 529 | (-) | CANNTG | S000407 | dehydration responsive |
| LEA 2 | MYCCONSENSUSAT | 529 | (+) | CANNTG | S000407 | dehydration responsive |
| LEA 2 | MYCCONSENSUSAT | 690 | (-) | CANNTG | S000407 | dehydration responsive |
| LEA 2 | MYCCONSENSUSAT | 690 | (+) | CANNTG | S000407 | dehydration responsive |
| LEA 2 | MYCCONSENSUSAT | 753 | (-) | CANNTG | S000407 | dehydration responsive |
| LEA 2 | MYCCONSENSUSAT | 753 | (+) | CANNTG | S000407 | dehydration responsive |
| LEA 2 | MYCCONSENSUSAT | 1082 | (-) | CANNTG | S000407 | dehydration responsive |
| LEA 2 | MYCCONSENSUSAT | 1082 | (+) | CANNTG | S000407 | dehydration responsive |
| LEA 2 | MYCCONSENSUSAT | 1237 | (-) | CANNTG | S000407 | dehydration responsive |
| LEA 2 | MYCCONSENSUSAT | 1237 | (+) | CANNTG | S000407 | dehydration responsive |
| LEA 2 | MYCCONSENSUSAT | 1298 | (-) | CANNTG | S000407 | dehydration responsive |
| LEA 2 | MYCCONSENSUSAT | 1298 | (+) | CANNTG | S000407 | dehydration responsive |
| LEA 2 | MYCCONSENSUSAT | 1354 | (-) | CANNTG | S000407 | dehydration responsive |
| LEA 2 | MYCCONSENSUSAT | 1354 | (+) | CANNTG | S000407 | dehydration responsive |
| LEA 2 | MYCCONSENSUSAT | 60 | (-) | CANNTG | S000407 | dehydration responsive |
| LEA 2 | MYCCONSENSUSAT | 60 | (+) | CANNTG | S000407 | dehydration responsive |
| LEA 2 | MYCCONSENSUSAT | 82 | (-) | CANNTG | S000407 | dehydration responsive |
| LEA 2 | MYCCONSENSUSAT | 82 | (+) | CANNTG | S000407 | dehydration responsive |
| LEA 2 | MYCCONSENSUSAT | 283 | (-) | CANNTG | S000407 | dehydration responsive |
| LEA 2 | MYCCONSENSUSAT | 283 | (+) | CANNTG | S000407 | dehydration responsive |
| LEA 2 | MYCCONSENSUSAT | 343 | (-) | CANNTG | S000407 | dehydration responsive |
| LEA 2 | MYCCONSENSUSAT | 343 | (+) | CANNTG | S000407 | dehydration responsive |
| LEA 2 | MYCCONSENSUSAT | 447 | (-) | CANNTG | S000407 | dehydration responsive |
| LEA 2 | MYCCONSENSUSAT | 447 | (+) | CANNTG | S000407 | dehydration responsive |
| LEA 2 | MYCCONSENSUSAT | 627 | (-) | CANNTG | S000407 | dehydration responsive |
| LEA 2 | MYCCONSENSUSAT | 627 | (+) | CANNTG | S000407 | dehydration responsive |
| LEA 2 | MYCCONSENSUSAT | 718 | (-) | CANNTG | S000407 | dehydration responsive |
| LEA 2 | MYCCONSENSUSAT | 718 | (+) | CANNTG | S000407 | dehydration responsive |
| LEA 2 | MYCCONSENSUSAT | 850 | (-) | CANNTG | S000407 | dehydration responsive |
| LEA 2 | MYCCONSENSUSAT | 850 | (+) | CANNTG | S000407 | dehydration responsive |
| LEA 2 | MYCCONSENSUSAT | 23 | (-) | CANNTG | S000407 | dehydration responsive |
| LEA 2 | MYCCONSENSUSAT | 23 | (+) | CANNTG | S000407 | dehydration responsive |
| LEA 2 | MYCCONSENSUSAT | 270 | (-) | CANNTG | S000407 | dehydration responsive |
| LEA 2 | MYCCONSENSUSAT | 270 | (+) | CANNTG | S000407 | dehydration responsive |
| LEA 2 | MYCCONSENSUSAT | 570 | (-) | CANNTG | S000407 | dehydration responsive |
| LEA 2 | MYCCONSENSUSAT | 570 | (+) | CANNTG | S000407 | dehydration responsive |
| LEA 2 | MYCCONSENSUSAT | 343 | (-) | CANNTG | S000407 | dehydration responsive |
| LEA 2 | MYCCONSENSUSAT | 343 | (+) | CANNTG | S000407 | dehydration responsive |
| LEA 2 | MYCCONSENSUSAT | 402 | (-) | CANNTG | S000407 | dehydration responsive |
| LEA 2 | MYCCONSENSUSAT | 402 | (+) | CANNTG | S000407 | dehydration responsive |
| LEA 2 | MYCCONSENSUSAT | 224 | (-) | CANNTG | S000407 | dehydration responsive |
| LEA 2 | MYCCONSENSUSAT | 224 | (+) | CANNTG | S000407 | dehydration responsive |
| LEA 2 | MYCCONSENSUSAT | 879 | (-) | CANNTG | S000407 | dehydration responsive |
| LEA 2 | MYCCONSENSUSAT | 879 | (+) | CANNTG | S000407 | dehydration responsive |
| LEA 2 | MYCCONSENSUSAT | 116 | (-) | CANNTG | S000407 | dehydration responsive |
| LEA 2 | MYCCONSENSUSAT | 116 | (+) | CANNTG | S000407 | dehydration responsive |
| LEA 2 | MYCCONSENSUSAT | 441 | (-) | CANNTG | S000407 | dehydration responsive |
| LEA 2 | MYCCONSENSUSAT | 441 | (+) | CANNTG | S000407 | dehydration responsive |
| LEA 2 | MYCCONSENSUSAT | 211 | (-) | CANNTG | S000407 | dehydration responsive |
| LEA 2 | MYCCONSENSUSAT | 211 | (+) | CANNTG | S000407 | dehydration responsive |
| LEA 2 | MYCCONSENSUSAT | 217 | (-) | CANNTG | S000407 | dehydration responsive |
| LEA 2 | MYCCONSENSUSAT | 217 | (+) | CANNTG | S000407 | dehydration responsive |
| LEA 2 | MYCCONSENSUSAT | 402 | (-) | CANNTG | S000407 | dehydration responsive |
| LEA 2 | MYCCONSENSUSAT | 402 | (+) | CANNTG | S000407 | dehydration responsive |
| LEA 2 | MYCCONSENSUSAT | 957 | (-) | CANNTG | S000407 | dehydration responsive |
| LEA 2 | MYCCONSENSUSAT | 957 | (+) | CANNTG | S000407 | dehydration responsive |
| LEA 2 | MYCCONSENSUSAT | 412 | (-) | CANNTG | S000407 | dehydration responsive |
| LEA 2 | MYCCONSENSUSAT | 412 | (+) | CANNTG | S000407 | dehydration responsive |
| LEA 2 | MYCCONSENSUSAT | 584 | (-) | CANNTG | S000407 | dehydration responsive |
| LEA 2 | MYCCONSENSUSAT | 584 | (+) | CANNTG | S000407 | dehydration responsive |
| LEA 2 | MYCCONSENSUSAT | 598 | (-) | CANNTG | S000407 | dehydration responsive |
| LEA 2 | MYCCONSENSUSAT | 598 | (+) | CANNTG | S000407 | dehydration responsive |
| LEA 2 | MYCCONSENSUSAT | 155 | (-) | CANNTG | S000407 | dehydration responsive |
| LEA 2 | MYCCONSENSUSAT | 155 | (+) | CANNTG | S000407 | dehydration responsive |
| LEA 2 | MYCCONSENSUSAT | 319 | (-) | CANNTG | S000407 | dehydration responsive |
| LEA 2 | MYCCONSENSUSAT | 319 | (+) | CANNTG | S000407 | dehydration responsive |
| LEA 2 | MYCCONSENSUSAT | 390 | (-) | CANNTG | S000407 | dehydration responsive |
| LEA 2 | MYCCONSENSUSAT | 390 | (+) | CANNTG | S000407 | dehydration responsive |
| LEA 2 | MYCCONSENSUSAT | 37 | (-) | CANNTG | S000407 | dehydration responsive |
| LEA 2 | MYCCONSENSUSAT | 37 | (+) | CANNTG | S000407 | dehydration responsive |
| LEA 2 | MYCCONSENSUSAT | 475 | (-) | CANNTG | S000407 | dehydration responsive |
| LEA 2 | MYCCONSENSUSAT | 475 | (+) | CANNTG | S000407 | dehydration responsive |
| LEA 2 | MYCCONSENSUSAT | 529 | (-) | CANNTG | S000407 | dehydration responsive |
| LEA 2 | MYCCONSENSUSAT | 529 | (+) | CANNTG | S000407 | dehydration responsive |
| LEA 2 | MYCCONSENSUSAT | 14 | (-) | CANNTG | S000407 | dehydration responsive |
| LEA 2 | MYCCONSENSUSAT | 14 | (+) | CANNTG | S000407 | dehydration responsive |
| LEA 2 | MYCCONSENSUSAT | 73 | (-) | CANNTG | S000407 | dehydration responsive |
| LEA 2 | MYCCONSENSUSAT | 73 | (+) | CANNTG | S000407 | dehydration responsive |
| LEA 2 | MYCCONSENSUSAT | 161 | (-) | CANNTG | S000407 | dehydration responsive |
| LEA 2 | MYCCONSENSUSAT | 161 | (+) | CANNTG | S000407 | dehydration responsive |
| LEA 2 | MYCCONSENSUSAT | 96 | (-) | CANNTG | S000407 | dehydration responsive |
| LEA 2 | MYCCONSENSUSAT | 96 | (+) | CANNTG | S000407 | dehydration responsive |
| LEA 2 | MYCCONSENSUSAT | 549 | (-) | CANNTG | S000407 | dehydration responsive |
| LEA 2 | MYCCONSENSUSAT | 549 | (+) | CANNTG | S000407 | dehydration responsive |
| LEA 2 | MYCCONSENSUSAT | 972 | (-) | CANNTG | S000407 | dehydration responsive |
| LEA 2 | MYCCONSENSUSAT | 972 | (+) | CANNTG | S000407 | dehydration responsive |
| LEA 2 | MYCCONSENSUSAT | 109 | (-) | CANNTG | S000407 | dehydration responsive |
| LEA 2 | MYCCONSENSUSAT | 109 | (+) | CANNTG | S000407 | dehydration responsive |
| LEA 2 | MYCCONSENSUSAT | 380 | (-) | CANNTG | S000407 | dehydration responsive |
| LEA 2 | MYCCONSENSUSAT | 380 | (+) | CANNTG | S000407 | dehydration responsive |
| LEA 2 | MYCCONSENSUSAT | 577 | (-) | CANNTG | S000407 | dehydration responsive |
| LEA 2 | MYCCONSENSUSAT | 577 | (+) | CANNTG | S000407 | dehydration responsive |
| LEA 2 | MYCCONSENSUSAT | 655 | (-) | CANNTG | S000407 | dehydration responsive |
| LEA 2 | MYCCONSENSUSAT | 655 | (+) | CANNTG | S000407 | dehydration responsive |
| LEA 2 | MYCCONSENSUSAT | 792 | (-) | CANNTG | S000407 | dehydration responsive |
| LEA 2 | MYCCONSENSUSAT | 792 | (+) | CANNTG | S000407 | dehydration responsive |
| LEA 2 | MYCCONSENSUSAT | 258 | (-) | CANNTG | S000407 | dehydration responsive |
| LEA 2 | MYCCONSENSUSAT | 258 | (+) | CANNTG | S000407 | dehydration responsive |
| LEA 2 | MYCCONSENSUSAT | 640 | (-) | CANNTG | S000407 | dehydration responsive |
| LEA 2 | MYCCONSENSUSAT | 640 | (+) | CANNTG | S000407 | dehydration responsive |
| LEA 2 | MYCCONSENSUSAT | 756 | (-) | CANNTG | S000407 | dehydration responsive |
| LEA 2 | MYCCONSENSUSAT | 756 | (+) | CANNTG | S000407 | dehydration responsive |
| LEA 2 | MYCCONSENSUSAT | 791 | (-) | CANNTG | S000407 | dehydration responsive |
| LEA 2 | MYCCONSENSUSAT | 791 | (+) | CANNTG | S000407 | dehydration responsive |
| LEA 2 | MYCCONSENSUSAT | 75 | (-) | CANNTG | S000407 | dehydration responsive |
| LEA 2 | MYCCONSENSUSAT | 75 | (+) | CANNTG | S000407 | dehydration responsive |
| LEA 2 | MYCCONSENSUSAT | 267 | (-) | CANNTG | S000407 | dehydration responsive |
| LEA 2 | MYCCONSENSUSAT | 267 | (+) | CANNTG | S000407 | dehydration responsive |
| LEA 2 | MYCCONSENSUSAT | 67 | (-) | CANNTG | S000407 | dehydration responsive |
| LEA 2 | MYCCONSENSUSAT | 67 | (+) | CANNTG | S000407 | dehydration responsive |
| LEA 2 | MYCCONSENSUSAT | 116 | (-) | CANNTG | S000407 | dehydration responsive |
| LEA 2 | MYCCONSENSUSAT | 116 | (+) | CANNTG | S000407 | dehydration responsive |
| LEA 2 | MYCCONSENSUSAT | 441 | (-) | CANNTG | S000407 | dehydration responsive |
| LEA 2 | MYCCONSENSUSAT | 441 | (+) | CANNTG | S000407 | dehydration responsive |
| LEA 2 | MYCCONSENSUSAT | 174 | (-) | CANNTG | S000407 | dehydration responsive |
| LEA 2 | MYCCONSENSUSAT | 174 | (+) | CANNTG | S000407 | dehydration responsive |
| LEA 2 | MYCCONSENSUSAT | 80 | (-) | CANNTG | S000407 | dehydration responsive |
| LEA 2 | MYCCONSENSUSAT | 80 | (+) | CANNTG | S000407 | dehydration responsive |
| LEA 2 | MYCCONSENSUSAT | 800 | (-) | CANNTG | S000407 | dehydration responsive |
| LEA 2 | MYCCONSENSUSAT | 800 | (+) | CANNTG | S000407 | dehydration responsive |
| LEA 2 | MYCCONSENSUSAT | 933 | (-) | CANNTG | S000407 | dehydration responsive |
| LEA 2 | MYCCONSENSUSAT | 933 | (+) | CANNTG | S000407 | dehydration responsive |
| LEA 2 | MYCCONSENSUSAT | 478 | (-) | CANNTG | S000407 | dehydration responsive |
| LEA 2 | MYCCONSENSUSAT | 478 | (+) | CANNTG | S000407 | dehydration responsive |
| LEA 2 | MYCCONSENSUSAT | 532 | (-) | CANNTG | S000407 | dehydration responsive |
| LEA 2 | MYCCONSENSUSAT | 532 | (+) | CANNTG | S000407 | dehydration responsive |
| LEA 2 | MYCCONSENSUSAT | 76 | (-) | CANNTG | S000407 | dehydration responsive |
| LEA 2 | MYCCONSENSUSAT | 76 | (+) | CANNTG | S000407 | dehydration responsive |
| LEA 2 | MYCCONSENSUSAT | 107 | (-) | CANNTG | S000407 | dehydration responsive |
| LEA 2 | MYCCONSENSUSAT | 107 | (+) | CANNTG | S000407 | dehydration responsive |
| LEA 2 | MYCCONSENSUSAT | 377 | (-) | CANNTG | S000407 | dehydration responsive |
| LEA 2 | MYCCONSENSUSAT | 377 | (+) | CANNTG | S000407 | dehydration responsive |
| LEA 2 | MYCCONSENSUSAT | 646 | (-) | CANNTG | S000407 | dehydration responsive |
| LEA 2 | MYCCONSENSUSAT | 646 | (+) | CANNTG | S000407 | dehydration responsive |
| LEA 2 | MYCCONSENSUSAT | 258 | (-) | CANNTG | S000407 | dehydration responsive |
| LEA 2 | MYCCONSENSUSAT | 258 | (+) | CANNTG | S000407 | dehydration responsive |
| LEA 2 | MYCCONSENSUSAT | 640 | (-) | CANNTG | S000407 | dehydration responsive |
| LEA 2 | MYCCONSENSUSAT | 640 | (+) | CANNTG | S000407 | dehydration responsive |
| LEA 2 | MYCCONSENSUSAT | 756 | (-) | CANNTG | S000407 | dehydration responsive |
| LEA 2 | MYCCONSENSUSAT | 756 | (+) | CANNTG | S000407 | dehydration responsive |
| LEA 2 | MYCCONSENSUSAT | 791 | (-) | CANNTG | S000407 | dehydration responsive |
| LEA 2 | MYCCONSENSUSAT | 791 | (+) | CANNTG | S000407 | dehydration responsive |
| LEA 2 | MYCCONSENSUSAT | 73 | (-) | CANNTG | S000407 | dehydration responsive |
| LEA 2 | MYCCONSENSUSAT | 73 | (+) | CANNTG | S000407 | dehydration responsive |
| LEA 2 | MYCCONSENSUSAT | 271 | (-) | CANNTG | S000407 | dehydration responsive |
| LEA 2 | MYCCONSENSUSAT | 271 | (+) | CANNTG | S000407 | dehydration responsive |
| LEA 2 | MYCCONSENSUSAT | 331 | (-) | CANNTG | S000407 | dehydration responsive |
| LEA 2 | MYCCONSENSUSAT | 331 | (+) | CANNTG | S000407 | dehydration responsive |
| LEA 2 | MYCCONSENSUSAT | 515 | (-) | CANNTG | S000407 | dehydration responsive |
| LEA 2 | MYCCONSENSUSAT | 515 | (+) | CANNTG | S000407 | dehydration responsive |
| LEA 2 | MYCCONSENSUSAT | 196 | (-) | CANNTG | S000407 | dehydration responsive |
| LEA 2 | MYCCONSENSUSAT | 196 | (+) | CANNTG | S000407 | dehydration responsive |
| LEA 2 | MYCCONSENSUSAT | 364 | (-) | CANNTG | S000407 | dehydration responsive |
| LEA 2 | MYCCONSENSUSAT | 364 | (+) | CANNTG | S000407 | dehydration responsive |
| LEA 2 | MYCCONSENSUSAT | 493 | (-) | CANNTG | S000407 | dehydration responsive |
| LEA 2 | MYCCONSENSUSAT | 493 | (+) | CANNTG | S000407 | dehydration responsive |
| LEA 2 | MYCCONSENSUSAT | 76 | (-) | CANNTG | S000407 | dehydration responsive |
| LEA 2 | MYCCONSENSUSAT | 76 | (+) | CANNTG | S000407 | dehydration responsive |
| LEA 2 | MYCCONSENSUSAT | 107 | (-) | CANNTG | S000407 | dehydration responsive |
| LEA 2 | MYCCONSENSUSAT | 107 | (+) | CANNTG | S000407 | dehydration responsive |
| LEA 2 | MYCCONSENSUSAT | 164 | (-) | CANNTG | S000407 | dehydration responsive |
| LEA 2 | MYCCONSENSUSAT | 164 | (+) | CANNTG | S000407 | dehydration responsive |
| LEA 2 | MYCCONSENSUSAT | 488 | (-) | CANNTG | S000407 | dehydration responsive |
| LEA 2 | MYCCONSENSUSAT | 488 | (+) | CANNTG | S000407 | dehydration responsive |
| LEA 2 | MYCCONSENSUSAT | 210 | (-) | CANNTG | S000407 | dehydration responsive |
| LEA 2 | MYCCONSENSUSAT | 210 | (+) | CANNTG | S000407 | dehydration responsive |
| LEA 2 | MYCCONSENSUSAT | 284 | (-) | CANNTG | S000407 | dehydration responsive |
| LEA 2 | MYCCONSENSUSAT | 284 | (+) | CANNTG | S000407 | dehydration responsive |
| LEA 2 | MYCCONSENSUSAT | 384 | (-) | CANNTG | S000407 | dehydration responsive |
| LEA 2 | MYCCONSENSUSAT | 384 | (+) | CANNTG | S000407 | dehydration responsive |
| LEA 2 | MYCCONSENSUSAT | 717 | (-) | CANNTG | S000407 | dehydration responsive |
| LEA 2 | MYCCONSENSUSAT | 717 | (+) | CANNTG | S000407 | dehydration responsive |
| LEA 2 | MYCCONSENSUSAT | 785 | (-) | CANNTG | S000407 | dehydration responsive |
| LEA 2 | MYCCONSENSUSAT | 785 | (+) | CANNTG | S000407 | dehydration responsive |
| LEA 2 | MYCCONSENSUSAT | 496 | (-) | CANNTG | S000407 | dehydration responsive |
| LEA 2 | MYCCONSENSUSAT | 496 | (+) | CANNTG | S000407 | dehydration responsive |
| LEA 2 | MYCCONSENSUSAT | 768 | (-) | CANNTG | S000407 | dehydration responsive |
| LEA 2 | MYCCONSENSUSAT | 768 | (+) | CANNTG | S000407 | dehydration responsive |
| LEA 2 | MYCCONSENSUSAT | 113 | (-) | CANNTG | S000407 | dehydration responsive |
| LEA 2 | MYCCONSENSUSAT | 113 | (+) | CANNTG | S000407 | dehydration responsive |
| LEA 2 | MYCCONSENSUSAT | 141 | (-) | CANNTG | S000407 | dehydration responsive |
| LEA 2 | MYCCONSENSUSAT | 141 | (+) | CANNTG | S000407 | dehydration responsive |
| LEA 2 | MYCCONSENSUSAT | 273 | (-) | CANNTG | S000407 | dehydration responsive |
| LEA 2 | MYCCONSENSUSAT | 273 | (+) | CANNTG | S000407 | dehydration responsive |
| LEA 2 | MYCCONSENSUSAT | 417 | (-) | CANNTG | S000407 | dehydration responsive |
| LEA 2 | MYCCONSENSUSAT | 417 | (+) | CANNTG | S000407 | dehydration responsive |
| LEA 2 | MYCCONSENSUSAT | 438 | (-) | CANNTG | S000407 | dehydration responsive |
| LEA 2 | MYCCONSENSUSAT | 438 | (+) | CANNTG | S000407 | dehydration responsive |
| LEA 2 | MYCCONSENSUSAT | 629 | (-) | CANNTG | S000407 | dehydration responsive |
| LEA 2 | MYCCONSENSUSAT | 629 | (+) | CANNTG | S000407 | dehydration responsive |
| LEA 2 | MYCCONSENSUSAT | 983 | (-) | CANNTG | S000407 | dehydration responsive |
| LEA 2 | MYCCONSENSUSAT | 983 | (+) | CANNTG | S000407 | dehydration responsive |
| LEA 2 | MYCCONSENSUSAT | 1080 | (-) | CANNTG | S000407 | dehydration responsive |
| LEA 2 | MYCCONSENSUSAT | 1080 | (+) | CANNTG | S000407 | dehydration responsive |
| LEA 2 | MYCCONSENSUSAT | 1684 | (-) | CANNTG | S000407 | dehydration responsive |
| LEA 2 | MYCCONSENSUSAT | 1684 | (+) | CANNTG | S000407 | dehydration responsive |
| LEA 2 | MYCCONSENSUSAT | 134 | (-) | CANNTG | S000407 | dehydration responsive |
| LEA 2 | MYCCONSENSUSAT | 134 | (+) | CANNTG | S000407 | dehydration responsive |
| LEA 2 | MYCCONSENSUSAT | 413 | (-) | CANNTG | S000407 | dehydration responsive |
| LEA 2 | MYCCONSENSUSAT | 413 | (+) | CANNTG | S000407 | dehydration responsive |
| LEA 2 | MYCCONSENSUSAT | 632 | (-) | CANNTG | S000407 | dehydration responsive |
| LEA 2 | MYCCONSENSUSAT | 632 | (+) | CANNTG | S000407 | dehydration responsive |
| LEA 2 | MYCCONSENSUSAT | 646 | (-) | CANNTG | S000407 | dehydration responsive |
| LEA 2 | MYCCONSENSUSAT | 646 | (+) | CANNTG | S000407 | dehydration responsive |
| LEA 2 | MYCCONSENSUSAT | 754 | (-) | CANNTG | S000407 | dehydration responsive |
| LEA 2 | MYCCONSENSUSAT | 754 | (+) | CANNTG | S000407 | dehydration responsive |
| LEA 2 | MYCCONSENSUSAT | 161 | (-) | CANNTG | S000407 | dehydration responsive |
| LEA 2 | MYCCONSENSUSAT | 161 | (+) | CANNTG | S000407 | dehydration responsive |
| LEA 2 | MYCCONSENSUSAT | 323 | (-) | CANNTG | S000407 | dehydration responsive |
| LEA 2 | MYCCONSENSUSAT | 323 | (+) | CANNTG | S000407 | dehydration responsive |
| LEA 2 | MYCCONSENSUSAT | 606 | (-) | CANNTG | S000407 | dehydration responsive |
| LEA 2 | MYCCONSENSUSAT | 606 | (+) | CANNTG | S000407 | dehydration responsive |
| LEA 2 | MYCCONSENSUSAT | 73 | (-) | CANNTG | S000407 | dehydration responsive |
| LEA 2 | MYCCONSENSUSAT | 73 | (+) | CANNTG | S000407 | dehydration responsive |
| LEA 2 | MYCCONSENSUSAT | 227 | (-) | CANNTG | S000407 | dehydration responsive |
| LEA 2 | MYCCONSENSUSAT | 227 | (+) | CANNTG | S000407 | dehydration responsive |
| LEA 2 | MYCCONSENSUSAT | 589 | (-) | CANNTG | S000407 | dehydration responsive |
| LEA 2 | MYCCONSENSUSAT | 589 | (+) | CANNTG | S000407 | dehydration responsive |
| LEA 2 | MYCCONSENSUSAT | 32 | (-) | CANNTG | S000407 | dehydration responsive |
| LEA 2 | MYCCONSENSUSAT | 32 | (+) | CANNTG | S000407 | dehydration responsive |
| LEA 2 | MYCCONSENSUSAT | 528 | (-) | CANNTG | S000407 | dehydration responsive |
| LEA 2 | MYCCONSENSUSAT | 528 | (+) | CANNTG | S000407 | dehydration responsive |
| LEA 2 | MYCCONSENSUSAT | 429 | (-) | CANNTG | S000407 | dehydration responsive |
| LEA 2 | MYCCONSENSUSAT | 429 | (+) | CANNTG | S000407 | dehydration responsive |
| LEA 2 | MYCCONSENSUSAT | 680 | (-) | CANNTG | S000407 | dehydration responsive |
| LEA 2 | MYCCONSENSUSAT | 680 | (+) | CANNTG | S000407 | dehydration responsive |
| LEA 2 | MYCCONSENSUSAT | 171 | (-) | CANNTG | S000407 | dehydration responsive |
| LEA 2 | MYCCONSENSUSAT | 171 | (+) | CANNTG | S000407 | dehydration responsive |
| LEA 2 | MYCCONSENSUSAT | 107 | (-) | CANNTG | S000407 | dehydration responsive |
| LEA 2 | MYCCONSENSUSAT | 107 | (+) | CANNTG | S000407 | dehydration responsive |
| LEA 2 | MYCCONSENSUSAT | 412 | (-) | CANNTG | S000407 | dehydration responsive |
| LEA 2 | MYCCONSENSUSAT | 412 | (+) | CANNTG | S000407 | dehydration responsive |
| LEA 2 | MYCCONSENSUSAT | 116 | (-) | CANNTG | S000407 | dehydration responsive |
| LEA 2 | MYCCONSENSUSAT | 116 | (+) | CANNTG | S000407 | dehydration responsive |
| LEA 2 | MYCCONSENSUSAT | 421 | (-) | CANNTG | S000407 | dehydration responsive |
| LEA 2 | MYCCONSENSUSAT | 421 | (+) | CANNTG | S000407 | dehydration responsive |
| LEA 2 | MYCCONSENSUSAT | 447 | (-) | CANNTG | S000407 | dehydration responsive |
| LEA 2 | MYCCONSENSUSAT | 447 | (+) | CANNTG | S000407 | dehydration responsive |
| LEA 2 | MYCCONSENSUSAT | 113 | (-) | CANNTG | S000407 | dehydration responsive |
| LEA 2 | MYCCONSENSUSAT | 113 | (+) | CANNTG | S000407 | dehydration responsive |
| LEA 2 | MYCCONSENSUSAT | 621 | (-) | CANNTG | S000407 | dehydration responsive |
| LEA 2 | MYCCONSENSUSAT | 621 | (+) | CANNTG | S000407 | dehydration responsive |
| LEA 2 | MYCCONSENSUSAT | 67 | (-) | CANNTG | S000407 | dehydration responsive |
| LEA 2 | MYCCONSENSUSAT | 67 | (+) | CANNTG | S000407 | dehydration responsive |
| LEA 2 | MYCCONSENSUSAT | 116 | (-) | CANNTG | S000407 | dehydration responsive |
| LEA 2 | MYCCONSENSUSAT | 116 | (+) | CANNTG | S000407 | dehydration responsive |
| LEA 2 | MYCCONSENSUSAT | 441 | (-) | CANNTG | S000407 | dehydration responsive |
| LEA 2 | MYCCONSENSUSAT | 441 | (+) | CANNTG | S000407 | dehydration responsive |
| LEA 2 | MYCCONSENSUSAT | 196 | (-) | CANNTG | S000407 | dehydration responsive |
| LEA 2 | MYCCONSENSUSAT | 196 | (+) | CANNTG | S000407 | dehydration responsive |
| LEA 2 | MYCCONSENSUSAT | 364 | (-) | CANNTG | S000407 | dehydration responsive |
| LEA 2 | MYCCONSENSUSAT | 364 | (+) | CANNTG | S000407 | dehydration responsive |
| LEA 2 | MYCCONSENSUSAT | 74 | (-) | CANNTG | S000407 | dehydration responsive |
| LEA 2 | MYCCONSENSUSAT | 74 | (+) | CANNTG | S000407 | dehydration responsive |
| LEA 2 | MYCCONSENSUSAT | 493 | (-) | CANNTG | S000407 | dehydration responsive |
| LEA 2 | MYCCONSENSUSAT | 493 | (+) | CANNTG | S000407 | dehydration responsive |
| LEA 2 | MYCCONSENSUSAT | 402 | (-) | CANNTG | S000407 | dehydration responsive |
| LEA 2 | MYCCONSENSUSAT | 402 | (+) | CANNTG | S000407 | dehydration responsive |
| LEA 2 | MYCCONSENSUSAT | 957 | (-) | CANNTG | S000407 | dehydration responsive |
| LEA 2 | MYCCONSENSUSAT | 957 | (+) | CANNTG | S000407 | dehydration responsive |
| LEA 2 | MYCCONSENSUSAT | 96 | (-) | CANNTG | S000407 | dehydration responsive |
| LEA 2 | MYCCONSENSUSAT | 96 | (+) | CANNTG | S000407 | dehydration responsive |
| LEA 2 | MYCCONSENSUSAT | 577 | (-) | CANNTG | S000407 | dehydration responsive |
| LEA 2 | MYCCONSENSUSAT | 577 | (+) | CANNTG | S000407 | dehydration responsive |
| LEA 2 | MYCCONSENSUSAT | 774 | (-) | CANNTG | S000407 | dehydration responsive |
| LEA 2 | MYCCONSENSUSAT | 774 | (+) | CANNTG | S000407 | dehydration responsive |
| LEA 2 | MYCCONSENSUSAT | 788 | (-) | CANNTG | S000407 | dehydration responsive |
| LEA 2 | MYCCONSENSUSAT | 788 | (+) | CANNTG | S000407 | dehydration responsive |
| LEA 2 | MYCCONSENSUSAT | 840 | (-) | CANNTG | S000407 | dehydration responsive |
| LEA 2 | MYCCONSENSUSAT | 840 | (+) | CANNTG | S000407 | dehydration responsive |
| LEA 2 | MYCCONSENSUSAT | 856 | (-) | CANNTG | S000407 | dehydration responsive |
| LEA 2 | MYCCONSENSUSAT | 856 | (+) | CANNTG | S000407 | dehydration responsive |
| LEA 2 | MYCCONSENSUSAT | 268 | (-) | CANNTG | S000407 | dehydration responsive |
| LEA 2 | MYCCONSENSUSAT | 268 | (+) | CANNTG | S000407 | dehydration responsive |
| LEA 2 | MYCCONSENSUSAT | 432 | (-) | CANNTG | S000407 | dehydration responsive |
| LEA 2 | MYCCONSENSUSAT | 432 | (+) | CANNTG | S000407 | dehydration responsive |
| LEA 2 | MYCCONSENSUSAT | 110 | (-) | CANNTG | S000407 | dehydration responsive |
| LEA 2 | MYCCONSENSUSAT | 110 | (+) | CANNTG | S000407 | dehydration responsive |
| LEA 2 | MYCCONSENSUSAT | 475 | (-) | CANNTG | S000407 | dehydration responsive |
| LEA 2 | MYCCONSENSUSAT | 475 | (+) | CANNTG | S000407 | dehydration responsive |
| LEA 2 | MYCCONSENSUSAT | 324 | (-) | CANNTG | S000407 | dehydration responsive |
| LEA 2 | MYCCONSENSUSAT | 324 | (+) | CANNTG | S000407 | dehydration responsive |
| LEA 2 | MYCCONSENSUSAT | 38 | (-) | CANNTG | S000407 | dehydration responsive |
| LEA 2 | MYCCONSENSUSAT | 38 | (+) | CANNTG | S000407 | dehydration responsive |
| LEA 2 | MYCCONSENSUSAT | 562 | (-) | CANNTG | S000407 | dehydration responsive |
| LEA 2 | MYCCONSENSUSAT | 562 | (+) | CANNTG | S000407 | dehydration responsive |
| LEA 2 | MYCCONSENSUSAT | 82 | (-) | CANNTG | S000407 | dehydration responsive |
| LEA 2 | MYCCONSENSUSAT | 82 | (+) | CANNTG | S000407 | dehydration responsive |
| LEA 2 | MYCCONSENSUSAT | 283 | (-) | CANNTG | S000407 | dehydration responsive |
| LEA 2 | MYCCONSENSUSAT | 283 | (+) | CANNTG | S000407 | dehydration responsive |
| LEA 2 | MYCCONSENSUSAT | 343 | (-) | CANNTG | S000407 | dehydration responsive |
| LEA 2 | MYCCONSENSUSAT | 343 | (+) | CANNTG | S000407 | dehydration responsive |
| LEA 2 | MYCCONSENSUSAT | 447 | (-) | CANNTG | S000407 | dehydration responsive |
| LEA 2 | MYCCONSENSUSAT | 447 | (+) | CANNTG | S000407 | dehydration responsive |
| LEA 2 | MYCCONSENSUSAT | 627 | (-) | CANNTG | S000407 | dehydration responsive |
| LEA 2 | MYCCONSENSUSAT | 627 | (+) | CANNTG | S000407 | dehydration responsive |
| LEA 2 | MYCCONSENSUSAT | 61 | (-) | CANNTG | S000407 | dehydration responsive |
| LEA 2 | MYCCONSENSUSAT | 61 | (+) | CANNTG | S000407 | dehydration responsive |
| LEA 2 | MYCCONSENSUSAT | 93 | (-) | CANNTG | S000407 | dehydration responsive |
| LEA 2 | MYCCONSENSUSAT | 93 | (+) | CANNTG | S000407 | dehydration responsive |
| LEA 2 | MYCCONSENSUSAT | 112 | (-) | CANNTG | S000407 | dehydration responsive |
| LEA 2 | MYCCONSENSUSAT | 112 | (+) | CANNTG | S000407 | dehydration responsive |
| LEA 2 | MYCCONSENSUSAT | 370 | (-) | CANNTG | S000407 | dehydration responsive |
| LEA 2 | MYCCONSENSUSAT | 370 | (+) | CANNTG | S000407 | dehydration responsive |
| LEA 2 | MYCCONSENSUSAT | 529 | (-) | CANNTG | S000407 | dehydration responsive |
| LEA 2 | MYCCONSENSUSAT | 529 | (+) | CANNTG | S000407 | dehydration responsive |
| LEA 2 | MYCCONSENSUSAT | 690 | (-) | CANNTG | S000407 | dehydration responsive |
| LEA 2 | MYCCONSENSUSAT | 690 | (+) | CANNTG | S000407 | dehydration responsive |
| LEA 2 | MYCCONSENSUSAT | 753 | (-) | CANNTG | S000407 | dehydration responsive |
| LEA 2 | MYCCONSENSUSAT | 753 | (+) | CANNTG | S000407 | dehydration responsive |
| LEA 2 | MYCCONSENSUSAT | 1237 | (-) | CANNTG | S000407 | dehydration responsive |
| LEA 2 | MYCCONSENSUSAT | 1237 | (+) | CANNTG | S000407 | dehydration responsive |
| LEA 2 | MYCCONSENSUSAT | 1298 | (-) | CANNTG | S000407 | dehydration responsive |
| LEA 2 | MYCCONSENSUSAT | 1298 | (+) | CANNTG | S000407 | dehydration responsive |
| LEA 2 | MYCCONSENSUSAT | 1354 | (-) | CANNTG | S000407 | dehydration responsive |
| LEA 2 | MYCCONSENSUSAT | 1354 | (+) | CANNTG | S000407 | dehydration responsive |
| LEA 2 | MYCCONSENSUSAT | 134 | (-) | CANNTG | S000407 | dehydration responsive |
| LEA 2 | MYCCONSENSUSAT | 134 | (+) | CANNTG | S000407 | dehydration responsive |
| LEA 2 | MYCCONSENSUSAT | 29 | (-) | CANNTG | S000407 | dehydration responsive |
| LEA 2 | MYCCONSENSUSAT | 29 | (+) | CANNTG | S000407 | dehydration responsive |
| LEA 2 | MYCCONSENSUSAT | 163 | (-) | CANNTG | S000407 | dehydration responsive |
| LEA 2 | MYCCONSENSUSAT | 163 | (+) | CANNTG | S000407 | dehydration responsive |
| LEA 2 | MYCCONSENSUSAT | 513 | (-) | CANNTG | S000407 | dehydration responsive |
| LEA 2 | MYCCONSENSUSAT | 513 | (+) | CANNTG | S000407 | dehydration responsive |
| LEA 2 | MYCCONSENSUSAT | 568 | (-) | CANNTG | S000407 | dehydration responsive |
| LEA 2 | MYCCONSENSUSAT | 568 | (+) | CANNTG | S000407 | dehydration responsive |
| LEA 2 | MYCCONSENSUSAT | 652 | (-) | CANNTG | S000407 | dehydration responsive |
| LEA 2 | MYCCONSENSUSAT | 652 | (+) | CANNTG | S000407 | dehydration responsive |
| LEA 2 | MYCCONSENSUSAT | 32 | (-) | CANNTG | S000407 | dehydration responsive |
| LEA 2 | MYCCONSENSUSAT | 32 | (+) | CANNTG | S000407 | dehydration responsive |
| LEA 2 | MYCCONSENSUSAT | 38 | (-) | CANNTG | S000407 | dehydration responsive |
| LEA 2 | MYCCONSENSUSAT | 38 | (+) | CANNTG | S000407 | dehydration responsive |
| LEA 2 | MYCCONSENSUSAT | 210 | (-) | CANNTG | S000407 | dehydration responsive |
| LEA 2 | MYCCONSENSUSAT | 210 | (+) | CANNTG | S000407 | dehydration responsive |
| LEA 2 | MYCCONSENSUSAT | 536 | (-) | CANNTG | S000407 | dehydration responsive |
| LEA 2 | MYCCONSENSUSAT | 536 | (+) | CANNTG | S000407 | dehydration responsive |
| LEA 2 | MYCCONSENSUSAT | 105 | (-) | CANNTG | S000407 | dehydration responsive |
| LEA 2 | MYCCONSENSUSAT | 105 | (+) | CANNTG | S000407 | dehydration responsive |
| LEA 2 | MYCCONSENSUSAT | 288 | (-) | CANNTG | S000407 | dehydration responsive |
| LEA 2 | MYCCONSENSUSAT | 288 | (+) | CANNTG | S000407 | dehydration responsive |
| LEA 2 | MYCCONSENSUSAT | 615 | (-) | CANNTG | S000407 | dehydration responsive |
| LEA 2 | MYCCONSENSUSAT | 615 | (+) | CANNTG | S000407 | dehydration responsive |
| LEA 2 | MYCCONSENSUSAT | 23 | (-) | CANNTG | S000407 | dehydration responsive |
| LEA 2 | MYCCONSENSUSAT | 23 | (+) | CANNTG | S000407 | dehydration responsive |
| LEA 2 | MYCCONSENSUSAT | 482 | (-) | CANNTG | S000407 | dehydration responsive |
| LEA 2 | MYCCONSENSUSAT | 482 | (+) | CANNTG | S000407 | dehydration responsive |
| LEA 2 | MYCCONSENSUSAT | 594 | (-) | CANNTG | S000407 | dehydration responsive |
| LEA 2 | MYCCONSENSUSAT | 594 | (+) | CANNTG | S000407 | dehydration responsive |
| LEA 2 | MYCCONSENSUSAT | 73 | (-) | CANNTG | S000407 | dehydration responsive |
| LEA 2 | MYCCONSENSUSAT | 73 | (+) | CANNTG | S000407 | dehydration responsive |
| LEA 2 | MYCCONSENSUSAT | 227 | (-) | CANNTG | S000407 | dehydration responsive |
| LEA 2 | MYCCONSENSUSAT | 227 | (+) | CANNTG | S000407 | dehydration responsive |
| LEA 2 | MYCCONSENSUSAT | 589 | (-) | CANNTG | S000407 | dehydration responsive |
| LEA 2 | MYCCONSENSUSAT | 589 | (+) | CANNTG | S000407 | dehydration responsive |
| LEA 2 | MYCCONSENSUSAT | 116 | (-) | CANNTG | S000407 | dehydration responsive |
| LEA 2 | MYCCONSENSUSAT | 116 | (+) | CANNTG | S000407 | dehydration responsive |
| LEA 2 | MYCCONSENSUSAT | 421 | (-) | CANNTG | S000407 | dehydration responsive |
| LEA 2 | MYCCONSENSUSAT | 421 | (+) | CANNTG | S000407 | dehydration responsive |
| LEA 2 | MYCCONSENSUSAT | 447 | (-) | CANNTG | S000407 | dehydration responsive |
| LEA 2 | MYCCONSENSUSAT | 447 | (+) | CANNTG | S000407 | dehydration responsive |
| LEA 2 | MYCCONSENSUSAT | 107 | (-) | CANNTG | S000407 | dehydration responsive |
| LEA 2 | MYCCONSENSUSAT | 107 | (+) | CANNTG | S000407 | dehydration responsive |
| LEA 2 | MYCCONSENSUSAT | 194 | (-) | CANNTG | S000407 | dehydration responsive |
| LEA 2 | MYCCONSENSUSAT | 194 | (+) | CANNTG | S000407 | dehydration responsive |
| LEA 2 | MYCCONSENSUSAT | 412 | (-) | CANNTG | S000407 | dehydration responsive |
| LEA 2 | MYCCONSENSUSAT | 412 | (+) | CANNTG | S000407 | dehydration responsive |
| LEA 2 | MYCCONSENSUSAT | 514 | (-) | CANNTG | S000407 | dehydration responsive |
| LEA 2 | MYCCONSENSUSAT | 514 | (+) | CANNTG | S000407 | dehydration responsive |
| LEA 2 | MYCCONSENSUSAT | 549 | (-) | CANNTG | S000407 | dehydration responsive |
| LEA 2 | MYCCONSENSUSAT | 549 | (+) | CANNTG | S000407 | dehydration responsive |
| LEA 2 | MYCCONSENSUSAT | 289 | (-) | CANNTG | S000407 | dehydration responsive |
| LEA 2 | MYCCONSENSUSAT | 289 | (+) | CANNTG | S000407 | dehydration responsive |
| LEA 2 | MYCCONSENSUSAT | 574 | (-) | CANNTG | S000407 | dehydration responsive |
| LEA 2 | MYCCONSENSUSAT | 574 | (+) | CANNTG | S000407 | dehydration responsive |
| LEA 2 | MYCCONSENSUSAT | 736 | (-) | CANNTG | S000407 | dehydration responsive |
| LEA 2 | MYCCONSENSUSAT | 736 | (+) | CANNTG | S000407 | dehydration responsive |
| LEA 2 | MYCCONSENSUSAT | 873 | (-) | CANNTG | S000407 | dehydration responsive |
| LEA 2 | MYCCONSENSUSAT | 873 | (+) | CANNTG | S000407 | dehydration responsive |
| LEA 2 | MYCCONSENSUSAT | 920 | (-) | CANNTG | S000407 | dehydration responsive |
| LEA 2 | MYCCONSENSUSAT | 920 | (+) | CANNTG | S000407 | dehydration responsive |
| LEA 2 | MYCCONSENSUSAT | 1143 | (-) | CANNTG | S000407 | dehydration responsive |
| LEA 2 | MYCCONSENSUSAT | 1143 | (+) | CANNTG | S000407 | dehydration responsive |
| LEA 2 | MYCCONSENSUSAT | 1289 | (-) | CANNTG | S000407 | dehydration responsive |
| LEA 2 | MYCCONSENSUSAT | 1289 | (+) | CANNTG | S000407 | dehydration responsive |
| LEA 2 | MYCCONSENSUSAT | 136 | (-) | CANNTG | S000407 | dehydration responsive |
| LEA 2 | MYCCONSENSUSAT | 136 | (+) | CANNTG | S000407 | dehydration responsive |
| LEA 2 | MYCCONSENSUSAT | 161 | (-) | CANNTG | S000407 | dehydration responsive |
| LEA 2 | MYCCONSENSUSAT | 161 | (+) | CANNTG | S000407 | dehydration responsive |
| LEA 2 | MYCCONSENSUSAT | 323 | (-) | CANNTG | S000407 | dehydration responsive |
| LEA 2 | MYCCONSENSUSAT | 323 | (+) | CANNTG | S000407 | dehydration responsive |
| LEA 2 | MYCCONSENSUSAT | 338 | (-) | CANNTG | S000407 | dehydration responsive |
| LEA 2 | MYCCONSENSUSAT | 338 | (+) | CANNTG | S000407 | dehydration responsive |
| LEA 2 | MYCCONSENSUSAT | 517 | (-) | CANNTG | S000407 | dehydration responsive |
| LEA 2 | MYCCONSENSUSAT | 517 | (+) | CANNTG | S000407 | dehydration responsive |
| LEA 2 | MYCCONSENSUSAT | 161 | (-) | CANNTG | S000407 | dehydration responsive |
| LEA 2 | MYCCONSENSUSAT | 161 | (+) | CANNTG | S000407 | dehydration responsive |
| LEA 2 | MYCCONSENSUSAT | 323 | (-) | CANNTG | S000407 | dehydration responsive |
| LEA 2 | MYCCONSENSUSAT | 323 | (+) | CANNTG | S000407 | dehydration responsive |
| LEA 2 | MYCCONSENSUSAT | 606 | (-) | CANNTG | S000407 | dehydration responsive |
| LEA 2 | MYCCONSENSUSAT | 606 | (+) | CANNTG | S000407 | dehydration responsive |
| LEA 2 | MYCCONSENSUSAT | 96 | (-) | CANNTG | S000407 | dehydration responsive |
| LEA 2 | MYCCONSENSUSAT | 96 | (+) | CANNTG | S000407 | dehydration responsive |
| LEA 2 | MYCCONSENSUSAT | 549 | (-) | CANNTG | S000407 | dehydration responsive |
| LEA 2 | MYCCONSENSUSAT | 549 | (+) | CANNTG | S000407 | dehydration responsive |
| LEA 2 | MYCCONSENSUSAT | 972 | (-) | CANNTG | S000407 | dehydration responsive |
| LEA 2 | MYCCONSENSUSAT | 972 | (+) | CANNTG | S000407 | dehydration responsive |
| LEA 2 | MYCCONSENSUSAT | 134 | (-) | CANNTG | S000407 | dehydration responsive |
| LEA 2 | MYCCONSENSUSAT | 134 | (+) | CANNTG | S000407 | dehydration responsive |
| LEA 2 | MYCCONSENSUSAT | 58 | (-) | CANNTG | S000407 | dehydration responsive |
| LEA 2 | MYCCONSENSUSAT | 58 | (+) | CANNTG | S000407 | dehydration responsive |
| LEA 2 | MYCCONSENSUSAT | 66 | (-) | CANNTG | S000407 | dehydration responsive |
| LEA 2 | MYCCONSENSUSAT | 66 | (+) | CANNTG | S000407 | dehydration responsive |
| LEA 2 | MYCCONSENSUSAT | 96 | (-) | CANNTG | S000407 | dehydration responsive |
| LEA 2 | MYCCONSENSUSAT | 96 | (+) | CANNTG | S000407 | dehydration responsive |
| LEA 2 | MYCCONSENSUSAT | 327 | (-) | CANNTG | S000407 | dehydration responsive |
| LEA 2 | MYCCONSENSUSAT | 327 | (+) | CANNTG | S000407 | dehydration responsive |
| LEA 2 | MYCCONSENSUSAT | 521 | (-) | CANNTG | S000407 | dehydration responsive |
| LEA 2 | MYCCONSENSUSAT | 521 | (+) | CANNTG | S000407 | dehydration responsive |
| LEA 2 | MYCCONSENSUSAT | 621 | (-) | CANNTG | S000407 | dehydration responsive |
| LEA 2 | MYCCONSENSUSAT | 621 | (+) | CANNTG | S000407 | dehydration responsive |
| LEA 3 | ABRELATERD1 | 97 | (-) | ACGTG | S000414 | ABA responsive elements |
| LEA 3 | ABRELATERD1 | 425 | (+) | ACGTG | S000414 | ABA responsive elements |
| LEA 3 | ABRELATERD1 | 80 | (+) | ACGTG | S000414 | ABA responsive elements |
| LEA 3 | ABRELATERD1 | 95 | (-) | ACGTG | S000414 | ABA responsive elements |
| LEA 3 | ABRELATERD1 | 96 | (+) | ACGTG | S000414 | ABA responsive elements |
| LEA 3 | ACGTATERD1 | 98 | (-) | ACGT | S000415 | early responsive to dehydration |
| LEA 3 | ACGTATERD1 | 98 | (+) | ACGT | S000415 | early responsive to dehydration |
| LEA 3 | ACGTATERD1 | 428 | (-) | ACGT | S000415 | early responsive to dehydration |
| LEA 3 | ACGTATERD1 | 428 | (+) | ACGT | S000415 | early responsive to dehydration |
| LEA 3 | ACGTATERD1 | 425 | (-) | ACGT | S000415 | early responsive to dehydration |
| LEA 3 | ACGTATERD1 | 425 | (+) | ACGT | S000415 | early responsive to dehydration |
| LEA 3 | ACGTATERD1 | 605 | (-) | ACGT | S000415 | early responsive to dehydration |
| LEA 3 | ACGTATERD1 | 605 | (+) | ACGT | S000415 | early responsive to dehydration |
| LEA 3 | ACGTATERD1 | 80 | (-) | ACGT | S000415 | early responsive to dehydration |
| LEA 3 | ACGTATERD1 | 80 | (+) | ACGT | S000415 | early responsive to dehydration |
| LEA 3 | ACGTATERD1 | 96 | (-) | ACGT | S000415 | early responsive to dehydration |
| LEA 3 | ACGTATERD1 | 96 | (+) | ACGT | S000415 | early responsive to dehydration |
| LEA 3 | ACGTATERD1 | 127 | (-) | ACGT | S000415 | early responsive to dehydration |
| LEA 3 | ACGTATERD1 | 127 | (+) | ACGT | S000415 | early responsive to dehydration |
| LEA 3 | ACGTATERD1 | 140 | (-) | ACGT | S000415 | early responsive to dehydration |
| LEA 3 | ACGTATERD1 | 140 | (+) | ACGT | S000415 | early responsive to dehydration |
| LEA 3 | ACGTATERD1 | 209 | (-) | ACGT | S000415 | early responsive to dehydration |
| LEA 3 | ACGTATERD1 | 209 | (+) | ACGT | S000415 | early responsive to dehydration |
| LEA 3 | ACGTATERD1 | 521 | (-) | ACGT | S000415 | early responsive to dehydration |
| LEA 3 | ACGTATERD1 | 521 | (+) | ACGT | S000415 | early responsive to dehydration |
| LEA 3 | ASF1MOTIFCAMV | 99 | (-) | TGACG | S000024 | Abiotic and biotic stress |
| LEA 3 | ASF1MOTIFCAMV | 354 | (+) | TGACG | S000024 | Abiotic and biotic stress |
| LEA 3 | ASF1MOTIFCAMV | 196 | (-) | TGACG | S000024 | Abiotic and biotic stress |
| LEA 3 | ASF1MOTIFCAMV | 186 | (-) | TGACG | S000024 | Abiotic and biotic stress |
| LEA 3 | ASF1MOTIFCAMV | 210 | (-) | TGACG | S000024 | Abiotic and biotic stress |
| LEA 3 | ASF1MOTIFCAMV | 2 | (+) | TGACG | S000024 | Abiotic and biotic stress |
| LEA 3 | ASF1MOTIFCAMV | 273 | (-) | TGACG | S000024 | Abiotic and biotic stress |
| LEA 3 | ASF1MOTIFCAMV | 603 | (-) | TGACG | S000024 | Abiotic and biotic stress |
| LEA 3 | ASF1MOTIFCAMV | 618 | (-) | TGACG | S000024 | Abiotic and biotic stress |
| LEA 3 | LTRE1HVBLT49 | 435 | (-) | CCGAAA | S000250 | necessary for coldor drought |
| LEA 3 | LTRE1HVBLT49 | 227 | (-) | CCGAAA | S000250 | necessary for coldor drought |
| LEA 3 | LTRE1HVBLT49 | 231 | (+) | CCGAAA | S000250 | necessary for coldor drought |
| LEA 3 | LTRE1HVBLT49 | 546 | (-) | CCGAAA | S000250 | necessary for coldor drought |
| LEA 3 | LTRECOREATCOR15 | 172 | (-) | CCGAC | S000153 | necessary for coldor drought |
| LEA 3 | LTRECOREATCOR15 | 289 | (-) | CCGAC | S000153 | necessary for coldor drought |
| LEA 3 | LTRECOREATCOR15 | 324 | (-) | CCGAC | S000153 | necessary for coldor drought |
| LEA 3 | LTRECOREATCOR15 | 551 | (-) | CCGAC | S000153 | necessary for coldor drought |
| LEA 3 | LTRECOREATCOR15 | 574 | (-) | CCGAC | S000153 | necessary for coldor drought |
| LEA 3 | MYB1AT | 11 | (+) | WAACCA | S000408 | MYB recognition site |
| LEA 3 | MYB1AT | 53 | (+) | WAACCA | S000408 | MYB recognition site |
| LEA 3 | MYB1AT | 143 | (+) | WAACCA | S000408 | MYB recognition site |
| LEA 3 | MYB1AT | 192 | (-) | WAACCA | S000408 | MYB recognition site |
| LEA 3 | MYB1AT | 728 | (-) | WAACCA | S000408 | MYB recognition site |
| LEA 3 | MYB1AT | 302 | (+) | WAACCA | S000408 | MYB recognition site |
| LEA 3 | MYB1AT | 297 | (+) | WAACCA | S000408 | MYB recognition site |
| LEA 3 | MYB1LEPR | 369 | (-) | GTTAGTT | S000443 | MYB recognition site |
| LEA 3 | MYB2AT | 719 | (+) | TAACTG | S000177 | MYB recognition site |
| LEA 3 | MYB2CONSENSUSAT | 243 | (+) | YAACKG | S000409 | Dehydratio/water stress |
| LEA 3 | MYB2CONSENSUSAT | 719 | (+) | YAACKG | S000409 | Dehydratio/water stress |
| LEA 3 | MYB2CONSENSUSAT | 215 | (-) | YAACKG | S000409 | Dehydratio/water stress |
| LEA 3 | MYB2CONSENSUSAT | 461 | (-) | YAACKG | S000409 | Dehydratio/water stress |
| LEA 3 | MYB2CONSENSUSAT | 614 | (-) | YAACKG | S000409 | Dehydratio/water stress |
| LEA 3 | MYB2CONSENSUSAT | 561 | (+) | YAACKG | S000409 | Dehydratio/water stress |
| LEA 3 | MYB2CONSENSUSAT | 107 | (-) | YAACKG | S000409 | Dehydratio/water stress |
| LEA 3 | MYBCORE | 11 | (-) | CNGTTR | S000176 | Dehydratio/water stress |
| LEA 3 | MYBCORE | 172 | (-) | CNGTTR | S000176 | Dehydratio/water stress |
| LEA 3 | MYBCORE | 243 | (-) | CNGTTR | S000176 | Dehydratio/water stress |
| LEA 3 | MYBCORE | 418 | (-) | CNGTTR | S000176 | Dehydratio/water stress |
| LEA 3 | MYBCORE | 380 | (+) | CNGTTR | S000176 | Dehydratio/water stress |
| LEA 3 | MYBCORE | 548 | (-) | CNGTTR | S000176 | Dehydratio/water stress |
| LEA 3 | MYBCORE | 719 | (-) | CNGTTR | S000176 | Dehydratio/water stress |
| LEA 3 | MYBCORE | 782 | (-) | CNGTTR | S000176 | Dehydratio/water stress |
| LEA 3 | MYBCORE | 215 | (+) | CNGTTR | S000176 | Dehydratio/water stress |
| LEA 3 | MYBCORE | 228 | (-) | CNGTTR | S000176 | Dehydratio/water stress |
| LEA 3 | MYBCORE | 382 | (-) | CNGTTR | S000176 | Dehydratio/water stress |
| LEA 3 | MYBCORE | 461 | (+) | CNGTTR | S000176 | Dehydratio/water stress |
| LEA 3 | MYBCORE | 614 | (+) | CNGTTR | S000176 | Dehydratio/water stress |
| LEA 3 | MYBCORE | 63 | (+) | CNGTTR | S000176 | Dehydratio/water stress |
| LEA 3 | MYBCORE | 561 | (-) | CNGTTR | S000176 | Dehydratio/water stress |
| LEA 3 | MYBCORE | 587 | (+) | CNGTTR | S000176 | Dehydratio/water stress |
| LEA 3 | MYBCORE | 107 | (+) | CNGTTR | S000176 | Dehydratio/water stress |
| LEA 3 | MYBCORE | 243 | (-) | CNGTTR | S000176 | Dehydratio/water stress |
| LEA 3 | MYBCOREATCYCB1 | 244 | (+) | AACGG | S000502 | Dehydratio/water stress |
| LEA 3 | MYBCOREATCYCB1 | 406 | (+) | AACGG | S000502 | Dehydratio/water stress |
| LEA 3 | MYBCOREATCYCB1 | 110 | (-) | AACGG | S000502 | Dehydratio/water stress |
| LEA 3 | MYBCOREATCYCB1 | 215 | (-) | AACGG | S000502 | Dehydratio/water stress |
| LEA 3 | MYBCOREATCYCB1 | 461 | (-) | AACGG | S000502 | Dehydratio/water stress |
| LEA 3 | MYBCOREATCYCB1 | 614 | (-) | AACGG | S000502 | Dehydratio/water stress |
| LEA 3 | MYBCOREATCYCB1 | 562 | (+) | AACGG | S000502 | Dehydratio/water stress |
| LEA 3 | MYBPLANT | 61 | (+) | MACCWAMC | S000167 | MYB recognition site |
| LEA 3 | MYBPLANT | 440 | (-) | MACCWAMC | S000167 | MYB recognition site |
| LEA 3 | MYBPLANT | 523 | (-) | MACCWAMC | S000167 | MYB recognition site |
| LEA 3 | MYBPZM | 63 | (+) | CCWACC | S000179 | MYB recognition site |
| LEA 3 | MYBPZM | 440 | (-) | CCWACC | S000179 | MYB recognition site |
| LEA 3 | MYBPZM | 564 | (-) | CCWACC | S000179 | MYB recognition site |
| LEA 3 | MYBST1 | 303 | (+) | GGATA | S000180 | MYB responsive |
| LEA 3 | MYBST1 | 88 | (-) | GGATA | S000180 | MYB responsive |
| LEA 3 | MYBST1 | 644 | (-) | GGATA | S000180 | MYB responsive |
| LEA 3 | MYBST1 | 224 | (-) | GGATA | S000180 | MYB responsive |
| LEA 3 | MYCATERD1 | 583 | (+) | CATGTG | S000413 | Dehydratio/water stress |
| LEA 3 | MYCATRD22 | 583 | (-) | CACATG | S000174 | Dehydratio/water stress |
| LEA 3 | MYCCONSENSUSAT | 14 | (-) | CANNTG | S000407 | Dehydratio/water stress |
| LEA 3 | MYCCONSENSUSAT | 14 | (+) | CANNTG | S000407 | Dehydratio/water stress |
| LEA 3 | MYCCONSENSUSAT | 67 | (-) | CANNTG | S000407 | Dehydratio/water stress |
| LEA 3 | MYCCONSENSUSAT | 67 | (+) | CANNTG | S000407 | Dehydratio/water stress |
| LEA 3 | MYCCONSENSUSAT | 179 | (-) | CANNTG | S000407 | Dehydratio/water stress |
| LEA 3 | MYCCONSENSUSAT | 179 | (+) | CANNTG | S000407 | Dehydratio/water stress |
| LEA 3 | MYCCONSENSUSAT | 526 | (-) | CANNTG | S000407 | Dehydratio/water stress |
| LEA 3 | MYCCONSENSUSAT | 526 | (+) | CANNTG | S000407 | Dehydratio/water stress |
| LEA 3 | MYCCONSENSUSAT | 95 | (-) | CANNTG | S000407 | Dehydratio/water stress |
| LEA 3 | MYCCONSENSUSAT | 95 | (+) | CANNTG | S000407 | Dehydratio/water stress |
| LEA 3 | MYCCONSENSUSAT | 23 | (-) | CANNTG | S000407 | Dehydratio/water stress |
| LEA 3 | MYCCONSENSUSAT | 23 | (+) | CANNTG | S000407 | Dehydratio/water stress |
| LEA 3 | MYCCONSENSUSAT | 473 | (-) | CANNTG | S000407 | Dehydratio/water stress |
| LEA 3 | MYCCONSENSUSAT | 473 | (+) | CANNTG | S000407 | Dehydratio/water stress |
| LEA 3 | MYCCONSENSUSAT | 583 | (-) | CANNTG | S000407 | Dehydratio/water stress |
| LEA 3 | MYCCONSENSUSAT | 583 | (+) | CANNTG | S000407 | Dehydratio/water stress |
| LEA 3 | MYCCONSENSUSAT | 111 | (-) | CANNTG | S000407 | Dehydratio/water stress |
| LEA 3 | MYCCONSENSUSAT | 111 | (+) | CANNTG | S000407 | Dehydratio/water stress |
| LEA 3 | MYCCONSENSUSAT | 609 | (-) | CANNTG | S000407 | Dehydratio/water stress |
| LEA 3 | MYCCONSENSUSAT | 609 | (+) | CANNTG | S000407 | Dehydratio/water stress |
| LEA 3 | MYCCONSENSUSAT | 107 | (-) | CANNTG | S000407 | Dehydratio/water stress |
| LEA 3 | MYCCONSENSUSAT | 107 | (+) | CANNTG | S000407 | Dehydratio/water stress |
| LEA 5 | ABRECE1HVA22 | 74 | (-) | TGCCACCGG | S000014 | ABA responsive elements |
| LEA 5 | ABRERATCAL | 296 | (+) | MACGYGB | S000507 | ABA responsive elements |
| LEA 5 | ABRERATCAL | 251 | (+) | MACGYGB | S000507 | ABA responsive elements |
| LEA 5 | ABRERATCAL | 1227 | (-) | MACGYGB | S000507 | ABA responsive elements |
| LEA 5 | ABRERATCAL | 221 | (+) | MACGYGB | S000507 | ABA responsive elements |
| LEA 5 | ACGTATERD1 | 29 | (-) | ACGT | S000415 | early responsive to dehydration |
| LEA 5 | ACGTATERD1 | 29 | (+) | ACGT | S000415 | early responsive to dehydration |
| LEA 5 | ACGTATERD1 | 297 | (-) | ACGT | S000415 | early responsive to dehydration |
| LEA 5 | ACGTATERD1 | 297 | (+) | ACGT | S000415 | early responsive to dehydration |
| LEA 5 | ACGTATERD1 | 193 | (-) | ACGT | S000415 | early responsive to dehydration |
| LEA 5 | ACGTATERD1 | 193 | (+) | ACGT | S000415 | early responsive to dehydration |
| LEA 5 | ACGTATERD1 | 278 | (-) | ACGT | S000415 | early responsive to dehydration |
| LEA 5 | ACGTATERD1 | 278 | (+) | ACGT | S000415 | early responsive to dehydration |
| LEA 5 | ACGTATERD1 | 252 | (-) | ACGT | S000415 | early responsive to dehydration |
| LEA 5 | ACGTATERD1 | 252 | (+) | ACGT | S000415 | early responsive to dehydration |
| LEA 5 | ACGTATERD1 | 80 | (-) | ACGT | S000415 | early responsive to dehydration |
| LEA 5 | ACGTATERD1 | 80 | (+) | ACGT | S000415 | early responsive to dehydration |
| LEA 5 | ACGTATERD1 | 834 | (-) | ACGT | S000415 | early responsive to dehydration |
| LEA 5 | ACGTATERD1 | 834 | (+) | ACGT | S000415 | early responsive to dehydration |
| LEA 5 | ACGTATERD1 | 1229 | (-) | ACGT | S000415 | early responsive to dehydration |
| LEA 5 | ACGTATERD1 | 1229 | (+) | ACGT | S000415 | early responsive to dehydration |
| LEA 5 | ACGTATERD1 | 222 | (-) | ACGT | S000415 | early responsive to dehydration |
| LEA 5 | ACGTATERD1 | 222 | (+) | ACGT | S000415 | early responsive to dehydration |
| LEA 5 | AGCBOXNPGLB | 546 | (-) | AGCCGCC | S000232 | early responsive to dehydration |
| LEA 5 | LTRE1HVBLT49 | 704 | (+) | CCGAAA | S000250 | necessary for coldor drought |
| LEA 5 | LTRE1HVBLT49 | 956 | (+) | CCGAAA | S000250 | necessary for coldor drought |
| LEA 5 | LTRE1HVBLT49 | 611 | (+) | CCGAAA | S000250 | necessary for coldor drought |
| LEA 5 | LTREATLTI78 | 1035 | (+) | ACCGACA | S000157 | necessary for coldor drought |
| LEA 5 | LTRECOREATCOR15 | 327 | (-) | CCGAC | S000153 | necessary for coldor drought |
| LEA 5 | LTRECOREATCOR15 | 357 | (+) | CCGAC | S000153 | necessary for coldor drought |
| LEA 5 | LTRECOREATCOR15 | 1036 | (+) | CCGAC | S000153 | necessary for coldor drought |
| LEA 5 | LTRECOREATCOR15 | 3 | (-) | CCGAC | S000153 | necessary for coldor drought |
| LEA 5 | LTRECOREATCOR15 | 3 | (-) | CCGAC | S000153 | necessary for coldor drought |
| LEA 5 | LTRECOREATCOR15 | 488 | (+) | CCGAC | S000153 | necessary for coldor drought |
| LEA 5 | LTRECOREATCOR15 | 802 | (-) | CCGAC | S000153 | necessary for coldor drought |
| LEA 5 | LTRECOREATCOR15 | 1552 | (-) | CCGAC | S000153 | necessary for coldor drought |
| LEA 5 | LTRECOREATCOR15 | 210 | (-) | CCGAC | S000153 | necessary for coldor drought |
| LEA 5 | MYB1AT | 422 | (+) | WAACCA | S000408 | MYB recognition site |
| LEA 5 | MYB1AT | 1205 | (+) | WAACCA | S000408 | MYB recognition site |
| LEA 5 | MYB1AT | 159 | (-) | WAACCA | S000408 | MYB recognition site |
| LEA 5 | MYB1AT | 216 | (-) | WAACCA | S000408 | MYB recognition site |
| LEA 5 | MYB1AT | 851 | (-) | WAACCA | S000408 | MYB recognition site |
| LEA 5 | MYB1AT | 871 | (-) | WAACCA | S000408 | MYB recognition site |
| LEA 5 | MYB2AT | 489 | (-) | TAACTG | S000177 | MYB recognition site |
| LEA 5 | MYB2AT | 479 | (-) | TAACTG | S000177 | MYB recognition site |
| LEA 5 | MYB2AT | 842 | (+) | TAACTG | S000177 | MYB recognition site |
| LEA 5 | MYB2CONSENSUSAT | 239 | (+) | YAACKG | S000409 | Dehydratio/water stress |
| LEA 5 | MYB2CONSENSUSAT | 263 | (+) | YAACKG | S000409 | Dehydratio/water stress |
| LEA 5 | MYB2CONSENSUSAT | 489 | (-) | YAACKG | S000409 | Dehydratio/water stress |
| LEA 5 | MYB2CONSENSUSAT | 32 | (+) | YAACKG | S000409 | Dehydratio/water stress |
| LEA 5 | MYB2CONSENSUSAT | 479 | (-) | YAACKG | S000409 | Dehydratio/water stress |
| LEA 5 | MYB2CONSENSUSAT | 797 | (-) | YAACKG | S000409 | Dehydratio/water stress |
| LEA 5 | MYB2CONSENSUSAT | 317 | (+) | YAACKG | S000409 | Dehydratio/water stress |
| LEA 5 | MYB2CONSENSUSAT | 359 | (+) | YAACKG | S000409 | Dehydratio/water stress |
| LEA 5 | MYB2CONSENSUSAT | 995 | (+) | YAACKG | S000409 | Dehydratio/water stress |
| LEA 5 | MYB2CONSENSUSAT | 60 | (+) | YAACKG | S000409 | Dehydratio/water stress |
| LEA 5 | MYB2CONSENSUSAT | 99 | (+) | YAACKG | S000409 | Dehydratio/water stress |
| LEA 5 | MYB2CONSENSUSAT | 492 | (+) | YAACKG | S000409 | Dehydratio/water stress |
| LEA 5 | MYB2CONSENSUSAT | 620 | (-) | YAACKG | S000409 | Dehydratio/water stress |
| LEA 5 | MYB2CONSENSUSAT | 634 | (-) | YAACKG | S000409 | Dehydratio/water stress |
| LEA 5 | MYB2CONSENSUSAT | 581 | (-) | YAACKG | S000409 | Dehydratio/water stress |
| LEA 5 | MYB2CONSENSUSAT | 842 | (+) | YAACKG | S000409 | Dehydratio/water stress |
| LEA 5 | MYB2CONSENSUSAT | 1019 | (-) | YAACKG | S000409 | Dehydratio/water stress |
| LEA 5 | MYB2CONSENSUSAT | 56 | (-) | YAACKG | S000409 | Dehydratio/water stress |
| LEA 5 | MYBCORE | 239 | (-) | CNGTTR | S000176 | Dehydratio/water stress |
| LEA 5 | MYBCORE | 263 | (-) | CNGTTR | S000176 | Dehydratio/water stress |
| LEA 5 | MYBCORE | 489 | (+) | CNGTTR | S000176 | Dehydratio/water stress |
| LEA 5 | MYBCORE | 32 | (-) | CNGTTR | S000176 | Dehydratio/water stress |
| LEA 5 | MYBCORE | 479 | (+) | CNGTTR | S000176 | Dehydratio/water stress |
| LEA 5 | MYBCORE | 554 | (+) | CNGTTR | S000176 | Dehydratio/water stress |
| LEA 5 | MYBCORE | 797 | (+) | CNGTTR | S000176 | Dehydratio/water stress |
| LEA 5 | MYBCORE | 139 | (-) | CNGTTR | S000176 | Dehydratio/water stress |
| LEA 5 | MYBCORE | 145 | (-) | CNGTTR | S000176 | Dehydratio/water stress |
| LEA 5 | MYBCORE | 317 | (-) | CNGTTR | S000176 | Dehydratio/water stress |
| LEA 5 | MYBCORE | 359 | (-) | CNGTTR | S000176 | Dehydratio/water stress |
| LEA 5 | MYBCORE | 470 | (-) | CNGTTR | S000176 | Dehydratio/water stress |
| LEA 5 | MYBCORE | 995 | (-) | CNGTTR | S000176 | Dehydratio/water stress |
| LEA 5 | MYBCORE | 60 | (-) | CNGTTR | S000176 | Dehydratio/water stress |
| LEA 5 | MYBCORE | 92 | (-) | CNGTTR | S000176 | Dehydratio/water stress |
| LEA 5 | MYBCORE | 99 | (-) | CNGTTR | S000176 | Dehydratio/water stress |
| LEA 5 | MYBCORE | 471 | (-) | CNGTTR | S000176 | Dehydratio/water stress |
| LEA 5 | MYBCORE | 492 | (-) | CNGTTR | S000176 | Dehydratio/water stress |
| LEA 5 | MYBCORE | 519 | (-) | CNGTTR | S000176 | Dehydratio/water stress |
| LEA 5 | MYBCORE | 443 | (+) | CNGTTR | S000176 | Dehydratio/water stress |
| LEA 5 | MYBCORE | 620 | (+) | CNGTTR | S000176 | Dehydratio/water stress |
| LEA 5 | MYBCORE | 634 | (+) | CNGTTR | S000176 | Dehydratio/water stress |
| LEA 5 | MYBCORE | 581 | (+) | CNGTTR | S000176 | Dehydratio/water stress |
| LEA 5 | MYBCORE | 842 | (-) | CNGTTR | S000176 | Dehydratio/water stress |
| LEA 5 | MYBCORE | 998 | (+) | CNGTTR | S000176 | Dehydratio/water stress |
| LEA 5 | MYBCORE | 1019 | (+) | CNGTTR | S000176 | Dehydratio/water stress |
| LEA 5 | MYBCORE | 1439 | (+) | CNGTTR | S000176 | Dehydratio/water stress |
| LEA 5 | MYBCORE | 10 | (-) | CNGTTR | S000176 | Dehydratio/water stress |
| LEA 5 | MYBCORE | 56 | (+) | CNGTTR | S000176 | Dehydratio/water stress |
| LEA 5 | MYBCOREATCYCB1 | 240 | (+) | AACGG | S000502 | Dehydratio/water stress |
| LEA 5 | MYBCOREATCYCB1 | 33 | (+) | AACGG | S000502 | Dehydratio/water stress |
| LEA 5 | MYBCOREATCYCB1 | 135 | (+) | AACGG | S000502 | Dehydratio/water stress |
| LEA 5 | MYBCOREATCYCB1 | 797 | (-) | AACGG | S000502 | Dehydratio/water stress |
| LEA 5 | MYBCOREATCYCB1 | 1155 | (+) | AACGG | S000502 | Dehydratio/water stress |
| LEA 5 | MYBCOREATCYCB1 | 360 | (+) | AACGG | S000502 | Dehydratio/water stress |
| LEA 5 | MYBCOREATCYCB1 | 996 | (+) | AACGG | S000502 | Dehydratio/water stress |
| LEA 5 | MYBCOREATCYCB1 | 61 | (+) | AACGG | S000502 | Dehydratio/water stress |
| LEA 5 | MYBCOREATCYCB1 | 100 | (+) | AACGG | S000502 | Dehydratio/water stress |
| LEA 5 | MYBCOREATCYCB1 | 493 | (+) | AACGG | S000502 | Dehydratio/water stress |
| LEA 5 | MYBCOREATCYCB1 | 1019 | (-) | AACGG | S000502 | Dehydratio/water stress |
| LEA 5 | MYBCOREATCYCB1 | 56 | (-) | AACGG | S000502 | Dehydratio/water stress |
| LEA 5 | MYBGAHV | 171 | (+) | TAACAAA | S000181 | MYB recognition site |
| LEA 5 | MYBGAHV | 939 | (-) | TAACAAA | S000181 | MYB recognition site |
| LEA 5 | MYBPLANT | 942 | (-) | MACCWAMC | S000167 | MYB recognition site |
| LEA 5 | MYBPLANT | 1440 | (-) | MACCWAMC | S000167 | MYB recognition site |
| LEA 5 | MYBPZM | 53 | (-) | CCWACC | S000179 | MYB recognition site |
| LEA 5 | MYBPZM | 262 | (-) | CCWACC | S000179 | MYB recognition site |
| LEA 5 | MYBPZM | 501 | (-) | CCWACC | S000179 | MYB recognition site |
| LEA 5 | MYBPZM | 1440 | (-) | CCWACC | S000179 | MYB recognition site |
| LEA 5 | MYBST1 | 466 | (+) | GGATA | S000180 | MYB responsive |
| LEA 5 | MYBST1 | 464 | (+) | GGATA | S000180 | MYB responsive |
| LEA 5 | MYBST1 | 670 | (+) | GGATA | S000180 | MYB responsive |
| LEA 5 | MYBST1 | 1083 | (+) | GGATA | S000180 | MYB responsive |
| LEA 5 | MYBST1 | 1104 | (+) | GGATA | S000180 | MYB responsive |
| LEA 5 | MYBST1 | 627 | (+) | GGATA | S000180 | MYB responsive |
| LEA 5 | MYBST1 | 825 | (+) | GGATA | S000180 | MYB responsive |
| LEA 5 | MYBST1 | 846 | (+) | GGATA | S000180 | MYB responsive |
| LEA 5 | MYBST1 | 336 | (+) | GGATA | S000180 | MYB responsive |
| LEA 5 | MYBST1 | 222 | (-) | GGATA | S000180 | MYB responsive |
| LEA 5 | MYBST1 | 327 | (-) | GGATA | S000180 | MYB responsive |
| LEA 5 | MYBST1 | 648 | (+) | GGATA | S000180 | MYB responsive |
| LEA 5 | MYBST1 | 882 | (-) | GGATA | S000180 | MYB responsive |
| LEA 5 | MYCATERD1 | 37 | (-) | CATGTG | S000413 | Dehydratio/water stress |
| LEA 5 | MYCATRD22 | 37 | (+) | CACATG | S000174 | Dehydratio/water stress |
| LEA 5 | MYCCONSENSUSAT | 112 | (-) | CANNTG | S000407 | Dehydratio/water stress |
| LEA 5 | MYCCONSENSUSAT | 112 | (+) | CANNTG | S000407 | Dehydratio/water stress |
| LEA 5 | MYCCONSENSUSAT | 263 | (-) | CANNTG | S000407 | Dehydratio/water stress |
| LEA 5 | MYCCONSENSUSAT | 263 | (+) | CANNTG | S000407 | Dehydratio/water stress |
| LEA 5 | MYCCONSENSUSAT | 322 | (-) | CANNTG | S000407 | Dehydratio/water stress |
| LEA 5 | MYCCONSENSUSAT | 322 | (+) | CANNTG | S000407 | Dehydratio/water stress |
| LEA 5 | MYCCONSENSUSAT | 367 | (-) | CANNTG | S000407 | Dehydratio/water stress |
| LEA 5 | MYCCONSENSUSAT | 367 | (+) | CANNTG | S000407 | Dehydratio/water stress |
| LEA 5 | MYCCONSENSUSAT | 220 | (-) | CANNTG | S000407 | Dehydratio/water stress |
| LEA 5 | MYCCONSENSUSAT | 220 | (+) | CANNTG | S000407 | Dehydratio/water stress |
| LEA 5 | MYCCONSENSUSAT | 336 | (-) | CANNTG | S000407 | Dehydratio/water stress |
| LEA 5 | MYCCONSENSUSAT | 336 | (+) | CANNTG | S000407 | Dehydratio/water stress |
| LEA 5 | MYCCONSENSUSAT | 389 | (-) | CANNTG | S000407 | Dehydratio/water stress |
| LEA 5 | MYCCONSENSUSAT | 389 | (+) | CANNTG | S000407 | Dehydratio/water stress |
| LEA 5 | MYCCONSENSUSAT | 677 | (-) | CANNTG | S000407 | Dehydratio/water stress |
| LEA 5 | MYCCONSENSUSAT | 677 | (+) | CANNTG | S000407 | Dehydratio/water stress |
| LEA 5 | MYCCONSENSUSAT | 710 | (-) | CANNTG | S000407 | Dehydratio/water stress |
| LEA 5 | MYCCONSENSUSAT | 710 | (+) | CANNTG | S000407 | Dehydratio/water stress |
| LEA 5 | MYCCONSENSUSAT | 1249 | (-) | CANNTG | S000407 | Dehydratio/water stress |
| LEA 5 | MYCCONSENSUSAT | 1249 | (+) | CANNTG | S000407 | Dehydratio/water stress |
| LEA 5 | MYCCONSENSUSAT | 317 | (-) | CANNTG | S000407 | Dehydratio/water stress |
| LEA 5 | MYCCONSENSUSAT | 317 | (+) | CANNTG | S000407 | Dehydratio/water stress |
| LEA 5 | MYCCONSENSUSAT | 668 | (-) | CANNTG | S000407 | Dehydratio/water stress |
| LEA 5 | MYCCONSENSUSAT | 668 | (+) | CANNTG | S000407 | Dehydratio/water stress |
| LEA 5 | MYCCONSENSUSAT | 884 | (-) | CANNTG | S000407 | Dehydratio/water stress |
| LEA 5 | MYCCONSENSUSAT | 884 | (+) | CANNTG | S000407 | Dehydratio/water stress |
| LEA 5 | MYCCONSENSUSAT | 1127 | (-) | CANNTG | S000407 | Dehydratio/water stress |
| LEA 5 | MYCCONSENSUSAT | 1127 | (+) | CANNTG | S000407 | Dehydratio/water stress |
| LEA 5 | MYCCONSENSUSAT | 1135 | (-) | CANNTG | S000407 | Dehydratio/water stress |
| LEA 5 | MYCCONSENSUSAT | 1135 | (+) | CANNTG | S000407 | Dehydratio/water stress |
| LEA 5 | MYCCONSENSUSAT | 37 | (-) | CANNTG | S000407 | Dehydratio/water stress |
| LEA 5 | MYCCONSENSUSAT | 37 | (+) | CANNTG | S000407 | Dehydratio/water stress |
| LEA 5 | MYCCONSENSUSAT | 373 | (-) | CANNTG | S000407 | Dehydratio/water stress |
| LEA 5 | MYCCONSENSUSAT | 373 | (+) | CANNTG | S000407 | Dehydratio/water stress |
| LEA 5 | MYCCONSENSUSAT | 566 | (-) | CANNTG | S000407 | Dehydratio/water stress |
| LEA 5 | MYCCONSENSUSAT | 566 | (+) | CANNTG | S000407 | Dehydratio/water stress |
| LEA 5 | MYCCONSENSUSAT | 643 | (-) | CANNTG | S000407 | Dehydratio/water stress |
| LEA 5 | MYCCONSENSUSAT | 643 | (+) | CANNTG | S000407 | Dehydratio/water stress |
| LEA 5 | MYCCONSENSUSAT | 777 | (-) | CANNTG | S000407 | Dehydratio/water stress |
| LEA 5 | MYCCONSENSUSAT | 777 | (+) | CANNTG | S000407 | Dehydratio/water stress |
| LEA 5 | MYCCONSENSUSAT | 257 | (-) | CANNTG | S000407 | Dehydratio/water stress |
| LEA 5 | MYCCONSENSUSAT | 257 | (+) | CANNTG | S000407 | Dehydratio/water stress |
| LEA 5 | MYCCONSENSUSAT | 405 | (-) | CANNTG | S000407 | Dehydratio/water stress |
| LEA 5 | MYCCONSENSUSAT | 405 | (+) | CANNTG | S000407 | Dehydratio/water stress |
| LEA 5 | MYCCONSENSUSAT | 620 | (-) | CANNTG | S000407 | Dehydratio/water stress |
| LEA 5 | MYCCONSENSUSAT | 620 | (+) | CANNTG | S000407 | Dehydratio/water stress |
| LEA 5 | MYCCONSENSUSAT | 634 | (-) | CANNTG | S000407 | Dehydratio/water stress |
| LEA 5 | MYCCONSENSUSAT | 634 | (+) | CANNTG | S000407 | Dehydratio/water stress |
| LEA 5 | MYCCONSENSUSAT | 740 | (-) | CANNTG | S000407 | Dehydratio/water stress |
| LEA 5 | MYCCONSENSUSAT | 740 | (+) | CANNTG | S000407 | Dehydratio/water stress |
| LEA 5 | MYCCONSENSUSAT | 581 | (-) | CANNTG | S000407 | Dehydratio/water stress |
| LEA 5 | MYCCONSENSUSAT | 581 | (+) | CANNTG | S000407 | Dehydratio/water stress |
| LEA 5 | MYCCONSENSUSAT | 836 | (-) | CANNTG | S000407 | Dehydratio/water stress |
| LEA 5 | MYCCONSENSUSAT | 836 | (+) | CANNTG | S000407 | Dehydratio/water stress |
| LEA 5 | MYCCONSENSUSAT | 905 | (-) | CANNTG | S000407 | Dehydratio/water stress |
| LEA 5 | MYCCONSENSUSAT | 905 | (+) | CANNTG | S000407 | Dehydratio/water stress |
| LEA 5 | MYCCONSENSUSAT | 965 | (-) | CANNTG | S000407 | Dehydratio/water stress |
| LEA 5 | MYCCONSENSUSAT | 965 | (+) | CANNTG | S000407 | Dehydratio/water stress |
| LEA 5 | MYCCONSENSUSAT | 1262 | (-) | CANNTG | S000407 | Dehydratio/water stress |
| LEA 5 | MYCCONSENSUSAT | 1262 | (+) | CANNTG | S000407 | Dehydratio/water stress |
| LEA 5 | MYCCONSENSUSAT | 1271 | (-) | CANNTG | S000407 | Dehydratio/water stress |
| LEA 5 | MYCCONSENSUSAT | 1271 | (+) | CANNTG | S000407 | Dehydratio/water stress |
| LEA 5 | MYCCONSENSUSAT | 1451 | (-) | CANNTG | S000407 | Dehydratio/water stress |
| LEA 5 | MYCCONSENSUSAT | 1451 | (+) | CANNTG | S000407 | Dehydratio/water stress |
[truncated: 44,907 more chars]
